# Bismuth radical catalysis in the activation and coupling of redox-active electrophiles

**DOI:** 10.1038/s41557-023-01229-7

**Published:** 2023-06-01

**Authors:** Mauro Mato, Davide Spinnato, Markus Leutzsch, Hye Won Moon, Edward J. Reijerse, Josep Cornella

**Affiliations:** 1 Max-Planck-Institut für Kohlenforschung, Mülheim an der Ruhr, Germany; 2 Max-Planck-Institut für Chemische Energiekonversion, Mülheim an der Ruhr, Germany

**Keywords:** Homogeneous catalysis, Catalytic mechanisms

## Abstract

Radical cross-coupling reactions represent a revolutionary tool to make C(*sp*^3^)–C and C(*sp*^3^)–heteroatom bonds by means of transition metals and photoredox or electrochemical approaches. However, the use of main-group elements to harness this type of reactivity has been little explored. Here we show how a low-valency bismuth complex is able to undergo one-electron oxidative addition with redox-active alkyl-radical precursors, mimicking the behaviour of first-row transition metals. This reactivity paradigm for bismuth gives rise to well-defined oxidative addition complexes, which could be fully characterized in solution and in the solid state. The resulting Bi(III)–C(*sp*^3^) intermediates display divergent reactivity patterns depending on the α-substituents of the alkyl fragment. Mechanistic investigations of this reactivity led to the development of a bismuth-catalysed C(*sp*^3^)–N cross-coupling reaction that operates under mild conditions and accommodates synthetically relevant NH-heterocycles as coupling partners.

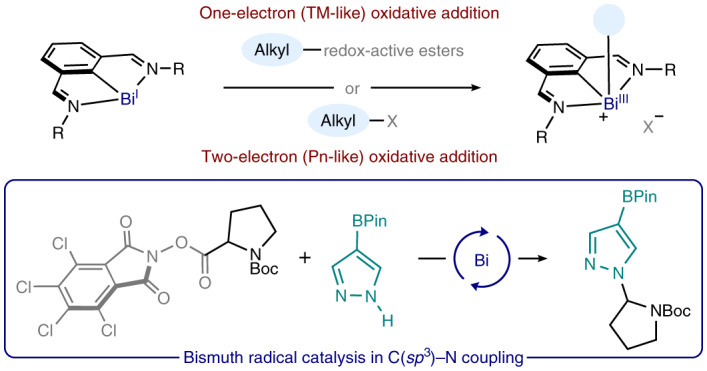

## Abstract (web summary)

The use of main-group elements in radical cross-coupling reactions has been little explored. Now, a low-valency bismuth complex has been shown to emulate the behaviour of first-row transition metals and undergo single-electron-transfer oxidative addition to redox-active electrophiles, leading to the development of a bismuth-catalysed C–N coupling reaction between amines and carboxylic acids.

## Main

**Fig. 1:**
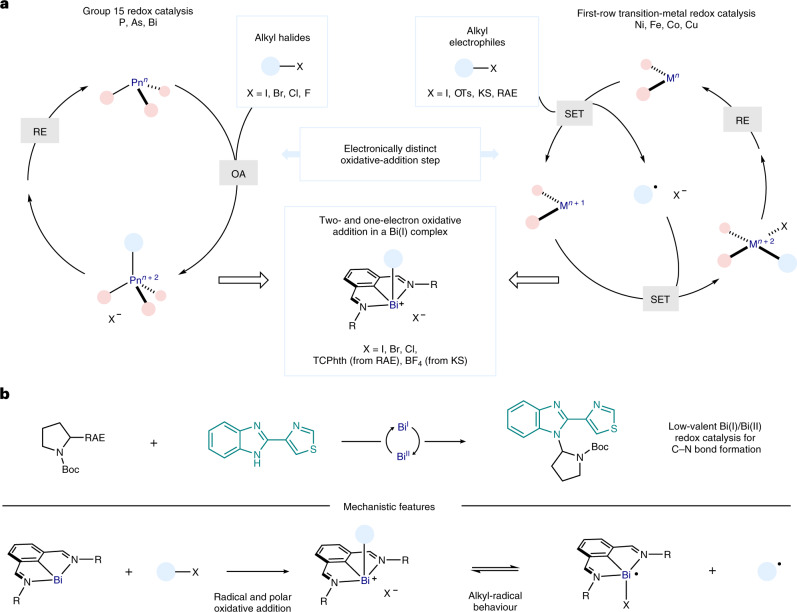
Unlocking single-electron oxidative-addition processes for bismuth. **a**, Merging pnictogen reactivity (left: polar, two-electron pathways dominate) with first-row transition-metal behaviour (right: radical, one-electron processes dominate) to unveil the oxidative addition of redox-active alkyl-radical precursors to bismuth(I) via SET. **b**, Development of a bismuth-catalysed C–N cross-coupling reaction through the study of the radical behaviour of alkyl-bismuth(III) complexes. OA, oxidative addition; RE, reductive elimination; Boc, *tert*-butoxycarbonyl; Ts, 4-toluenesulfonyl; Pn, pnictogen. R = *tert*-butyl.

Metal-catalysed radical cross-coupling reactions represent a conceptual paradigm shift from the historical two-electron polar disconnections^1^, resulting in a new approach for the synthesis of organic molecules^2^. Particularly, disconnections based on the coupling of alkyl-radical fragments have been shown to hold tremendous potential in making C(*sp*^3^)–C and C(*sp*^3^)–heteroatom bonds^3,4^. The evolution and application of such a synthetic strategy is linked to advances in the fields of photoredox catalysis^5–7^ and electrochemical synthesis^8,9^ and, especially, their combination with first-row transition-metal catalysis^10–13^. Indeed, elements such as Fe, Co, Ni or Cu hold a preferential place when one-electron processes are required in cross-coupling cycles, resulting in redox events occurring via (*n*)/(*n* + 1)/(*n* + 2) oxidation states (Fig. 1a, right). This particular chemical behaviour leads to the facile generation of alkyl-radical fragments through single-electron transfer (SET) oxidative addition from precursors such as redox-active esters (RAEs) or Katritzky salts (KSs).

Recent years have witnessed significant efforts towards mimicking the redox behaviour of transition metals by main-group elements^14^. For instance, pnictogens can take part in S_N_2-type polar oxidative additions resulting in two-electron manoeuvering throughout (*n*)/(*n* + 2) redox catalytic cycles, emulating those of late transition metals (Fig. 1a, left)^15^. However, radical oxidative additions of redox-active electrophiles have generally been restricted to first-row transition metals and well-defined examples of this process with a main-group complex remain elusive^6,16^. Very recently, bismuth redox catalysis has been established as an emerging platform for organic synthesis^17^ and our group has shown how Bi(III/V) or Bi(I/III) catalytic cycles can lead to the development of C–F (ref. ^18^), C–O (ref. ^19^) or C–H (ref. ^20^) bond-forming reactions, among others^21^. Nevertheless, despite the fact that persistent and stable radicals of heavier main-group elements are known^22^, bismuth radical catalysis has been significantly underexplored^23^. Bi(II/III) catalytic cycles have been postulated for the living radical polymerization of alkenes^24^ or the cycloisomerization of 4-iodoalkenes^25^. This, together with further reports probing the existence of bismuth(II)-centred radicals^26^, prompted us to explore the behaviour of the Bi(I/II) pair in SET-based oxidative additions of redox-active alkyl electrophiles. In this Article, we show how a well-defined bismuthinidene (**1**) reacts with alkyl phthalimide esters and alkyl KSs to give alkyl-bismuth(III) adducts, which were found to behave as Bi–C radical-equilibrium complexes (Fig. 1b, bottom). Additionally, we discovered that α-amino alkyl-radical fragments resulting from this process can be easily oxidized by Bi(II), giving rise to iminium ions^27,28^ that can be trapped by N-nucleophiles. This observation led to the development of a Bi-catalysed radical C–N cross-coupling reaction with a wide scope of both coupling partners (Fig. 1b, top). In spite of the vast number of alkyl-radical couplings developed during the past decade, only a few examples of C(*sp*^3^)–N bond formation from redox-active radical precursors have been reported^29^, mainly relying on photoredox set-ups^30–33^, electrochemical synthesis^34,35^ or the use of an excess of chemical oxidant^36^. In this Article, we demonstrate that catalytic amounts of a Bi(I) complex can promote this type of transformation in an autonomous manner, without the need for a photoredox system, a chemical oxidant, an external base or an electrochemical set-up.

## Results and discussion

**Fig. 2:**
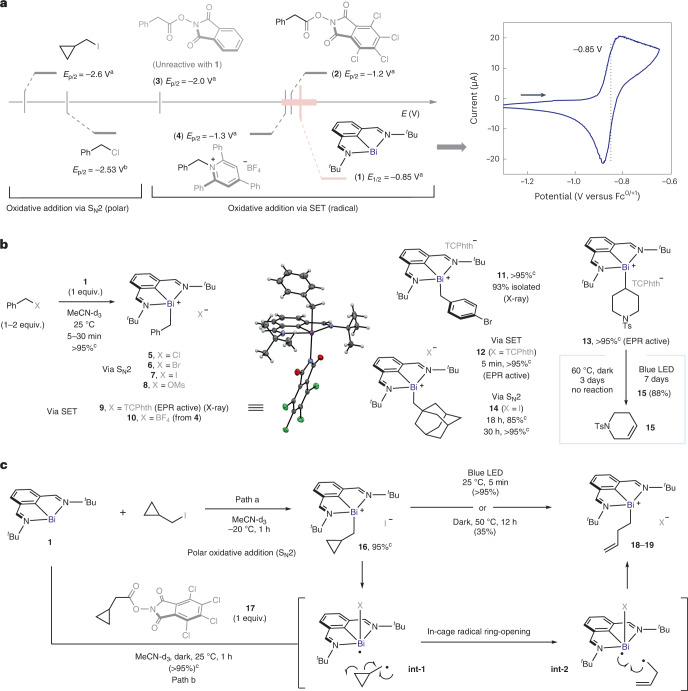
Oxidative additions to bismuth(I). **a**, Evaluating electronically different (polar, E_p/2_ < –2.0 V, versus radical, E_p/2_ > –2.0 V) oxidative additions to bismuthinidene **1** (left). Cyclic voltammetry of **1** (right). **b**, Stable oxidative-addition complexes accessed via S_N_2 (**5–8** and **14**) or SET (**9–13**) mechanisms. **c**, Evidence for alkyl-radical formation after oxidative addition. ^a^ Cyclic voltammetry recorded in MeCN, potential in V versus Fc^0/+^. ^b^ Cyclic voltammetry recorded in MeCN (ref. ^47^); potential in V versus Fc^0/+^ converted from V versus saturated calomel electrode (−2.13 V). ^c^ Yields and conversions determined by ^1^H NMR, unless noted otherwise. Ts, 4-toluenesulfonyl; MsO, mesylate.

As a result of the high nucleophilicity of the 6*p*^2^ lone pair on the Bi(I) centre, bismuthinidene **1** (refs. ^37,38^) has recently been shown to engage in polar S_N_2-type reactions with alkyl halides and triflates^39^. Similarly, **1** reacted quantitatively with a range of benzyl (pseudo)halides (Cl, Br, I, mesylate) to give benzyl bismuth(III) complexes **5–8** (Fig. 2b). Cyclic-voltammetry analysis of **1** (*E*_1/2_ = −0.85 versus Fc^0/+^, the ferrocene/ferrocenium couple) provides evidence that C‒X (X = halide) cleavage should proceed through a classical S_N_2 pathway (*E*_p/2_ < −2.0 V versus Fc^0/+^). On the other hand, the electrochemical behaviour suggested that **1** could potentially engage in SET oxidative-addition processes with alkyl redox-active electrophiles (Fig. 2a). Accordingly, reaction of **1** with 1 equiv. of tetrachlorophthalimide (TCPhth) ester **2** (*E*_p/2_ = −1.2 V versus Fc^0/+^) cleanly afforded benzyl bismuth(III) complex **9** after SET, fragmentation, release of CO_2_ and radical recombination (given that the potential difference between **1** and **2** is approximately 0.35 V, SET between **1** and **2** can be estimated to be approimately 8 kcal mol^−1^ uphill, but subsequent release of CO_2_ can drive the oxidative-addition process)^40^. The resulting alkyl-bismuth(III) adduct could be fully characterized by NMR, high-resolution mass spectrometry (HRMS) and single-crystal X-ray diffraction. Furthermore, KS **4** (*E*_p/2_ = −1.3 V versus Fc^0/+^) also underwent radical oxidative addition with **1** to give **10**. As expected, non-chlorinated phthalimide ester **3** (*E*_p/2_ = −2.0 V versus Fc^0/+^) remained unreacted when mixed with **1**. Besides benzyl groups, the same process occurs with primary (**12**) or secondary (**13**) RAEs, leading to stable alkyl-bismuth(III) complexes. Tertiary RAEs such as the one derived from 1-adamantanecarboxylic acid did also react with **1**, but the resulting adducts were found to be unstable and could not be characterized under standard conditions. Interestingly, the process is orthogonal to classical polar transition-metal oxidative additions, as it could be performed in the presence of an aryl bromide, giving **11** as the sole product in 93% yield (Fig. 2b). This reactivity is a rare example where bismuth, besides emulating the redox behaviour of first-row transition metals during oxidative addition, allows the isolation and characterization of the corresponding alkyl‒metal species resulting from radical recombination. We also found that, whereas classical S_N_2 reactivity is sensitive to steric effects (>24 h for **14**), single-electron oxidative addition of the corresponding RAE led to quantitative formation of complex **12** in <5 min. Furthermore, we found complexes **9**, **12** and **13** to be active by electron paramagnetic resonance (EPR) spectroscopy, especially upon light irradiation. Low-temperature EPR analysis of **12** suggests the formation of two radical species that decay at different rates. This is consistent with the homolysis of the C–Bi bond (see Supplementary Information for details)^41,42^. To investigate this behaviour further, the reaction of bismuthinidene **1** with cyclopropylmethyl iodide was monitored by NMR at low temperature in the dark (Fig. 2c). Complete conversion into cyclopropylmethyl adduct **16** was observed within 1 h at −20 °C. When the mixture was warmed to 50 °C, a slow but steady conversion to ring-opening compound **18** was observed (35% after 12 h).

**Fig. 3:**
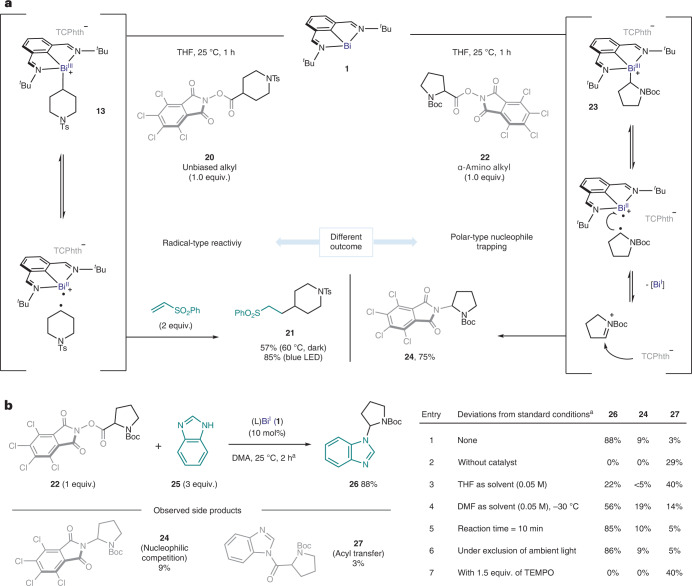
Divergent reactivity of an unbiased alkyl-bismuth complex and an α-amino alkyl-bismuth complex. **a**, Stable unbiased alkyl-bismuth(III) intermediates displaying typical alkyl-radical reactivity (left) and unstable α-amino alkyl-bismuth(III) complexes that evolve into iminium ion intermediates upon release of bismuth(I) (right). **b**, Development of a bismuth-catalysed C–N cross-coupling reaction based on the oxidation of α-amino alkyl radicals. ^a^Standard reaction conditions: **22** (1 equiv.) and **25** (3 equiv.) in the presence of bismuthinidene **1** (10 mol%) in DMA (0.033 M) at 25 °C for 2 h. Yields determined by ^1^H NMR using diphenylmethane as internal standard. Ts, 4-toluenesulfonyl; Boc, *tert*-butoxycarbonyl.

**Fig. 4:**
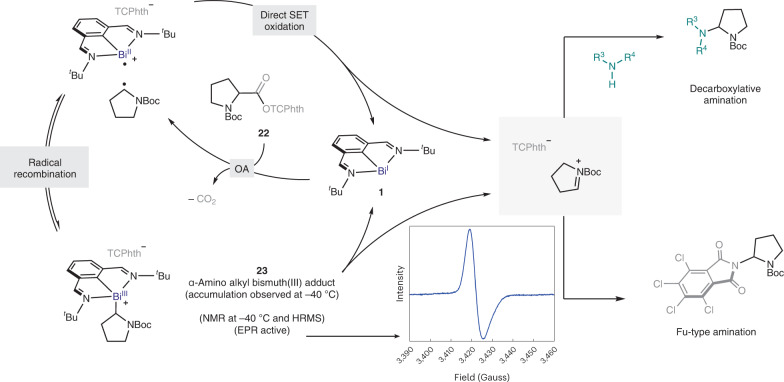
Proposed mechanistic rationale. The C–N cross-coupling reaction of α-amino and α-oxo acids via bismuth(I)-catalysed SET. The graph shows EPR analysis of **23**. R^3^R^4^NH, NH-heterocycle; OA, oxidative addition; Boc, *tert*-butoxycarbonyl.

This indicates that homolysis of the Bi‒C bond in **16** takes place, leading to an in-cage radical pair (**int-1**). Furthermore, subjecting a solution of **16** to blue light-emitting diode (LED) irradiation resulted in complete conversion into open product **18** within 5 min, showing that light can accelerate the radical ring-opening process^25^. Conversely, when cyclopropylmethyl RAE **17** was reacted with bismuthinidene **1**, a complex analogous to **16** was not observed; instead, radical ring-opening product **19** was immediately obtained, even in the dark. This is consistent with the two distinct mechanistic scenarios for the oxidative addition. On one hand, polar S_N_2-type reaction of cyclopropylmethyl iodide with **1** initially leads to **16**, which eventually ring-opens via alkyl-radical formation. On the other hand, SET and fragmentation of RAE **17** lead to an in-cage bismuth(II)/alkyl radical pair (**int-1**), for which cyclopropane ring-opening is faster than radical recombination, resulting in the formation of **19** (Fig. 2b). This alkyl radical-type reactivity is consistent with the behaviour displayed by these complexes: the secondary alkyl radical derived from **13** reacts with Michael acceptors such as phenylvinylsulfone giving Giese addition product **21**, either in the dark (57%) or under blue-light irradiation (85%) (Fig. 3a, left). Additionally, the alkyl fragment of several complexes reacted with (2,2,6,6-tetramethylpiperidin-1-yl)oxyl radical (TEMPO) leading to C(*sp*^3^)‒TEMPO adducts. Moreover, we observed that catalytic amounts of **1** can promote Giese-type reactions, among others, upon blue-light irradiation (Supplementary Information)^43^. When investigating the stability of the Bi(III)‒alkyl compounds in solution, it was found that benzyl bismuth(III) complex **9** was especially sensitive to light irradiation, resulting in decomposition mainly to benzyl–benzyl dimers and unselective benzylation of the N,C,N ligand. On the other hand, complex **13** was stable in solution, even after 3 days at 60 °C (Fig. 2b)^44^. However, under blue-LED irradiation, **13** underwent slow but clean conversion into elimination product **15** and Bi(I), in a radical-type elimination reminiscent of that of alkylcobaloximes^45^. Furthermore, scrambling experiments confirm that the exchange of alkyl fragments between two different Bi(III) adducts is also possible (Supplementary Information). Interestingly, when attempting the isolation of α-amino alkyl-bismuth(III) adduct **23** derived from proline, we observed the exclusive formation of the product of decarboxylative amination **24**, with recovery of bismuthinidene **1** (Fig. 3a, right)^30,33^. It was speculated that product **24** would arise from the oxidation of the corresponding α-amino alkyl radical by a highly reactive bismuth(II) species. This would lead to the formation of an electrophilic iminium ion^28^, which ultimately reacts with the TCPhth anion to make the C‒N bond (Fig. 4 and Supplementary Information).

At this point, it was envisaged that this reactivity could exploit the resulting iminium intermediates with external N-nucleophiles, leading to a formal C–N cross-coupling reaction. After optimization of the conditions, we found that the reaction of RAE **22** with 3 equiv. of benzimidazole (**25**) in the presence of 10 mol% of **1** in dimethylacetamide (DMA) at 25 °C afforded the product of C–N cross coupling (**26**) in 88% yield within 2 h (Fig. 3b). Under these conditions, the only observed side products were **24** (nucleophilic competition by TCPhth) and the expected amide bond-formation product **27**, which could be minimized by controlling the stoichiometry and selecting the appropriate solvent (Fig. 3b, entry 1 versus entry 3) (see Supplementary Information for optimization details). Control experiments without the Bi catalyst led exclusively to an acyl-transfer product (Fig, 3b, entry 2). The high efficiency of the optimized reaction relies on the faster kinetics of the Bi-catalysed radical reaction compared to the background amide formation. For example, the reaction could be carried out at −30 °C in DMF (Fig. 3b, entry 4) or at room temperature in DMA in only 10 min (Fig. 3b, entry 5), giving the desired product in 56 and 85% yield, respectively. To exclude completely the requirement of photoexcitation for any of the steps of the transformation to proceed, the reaction was carried out under exclusion of ambient light, giving comparable results (Fig. 3b, entry 6). As expected, the addition of TEMPO completely inhibited the reaction (Fig. 3b, entry 7)^41^. Other bismuth(I) complexes and different redox couples tested led to decreased yields or inactivity, thus highlighting the importance of the finely tuned redox properties of **1** (Supplementary Information).

This methodology led us to the assembly of a wide variety of products containing an aminal- or a hemiaminal-ether structural motif, which would be challenging to construct from the parent halide (Table 1). RAEs of readily available natural and non-natural α-amino acids were investigated (either fully protected or with free N–H bonds) as electrophilic partners. C–N coupling products derived from proline (**26**), phenylalanine (**29**), valine (**30** and **34**), leucine (**35**), glutamic acid (**36**) or pipecolic acid (**32** and **33**) were successfully obtained in good to excellent yields. Synthetically relevant N-heterocycles bearing free N‒H bonds were evaluated. Using proline-derived RAE **22**, the corresponding C–N products of benzimidazoles (**26**, **28** and **45**), triazole (**37**), imidazoles (**38**, **44** and **46**) and pyrazoles (**32**, **33** and **40**–**43**) were obtained.

**Table 1:**
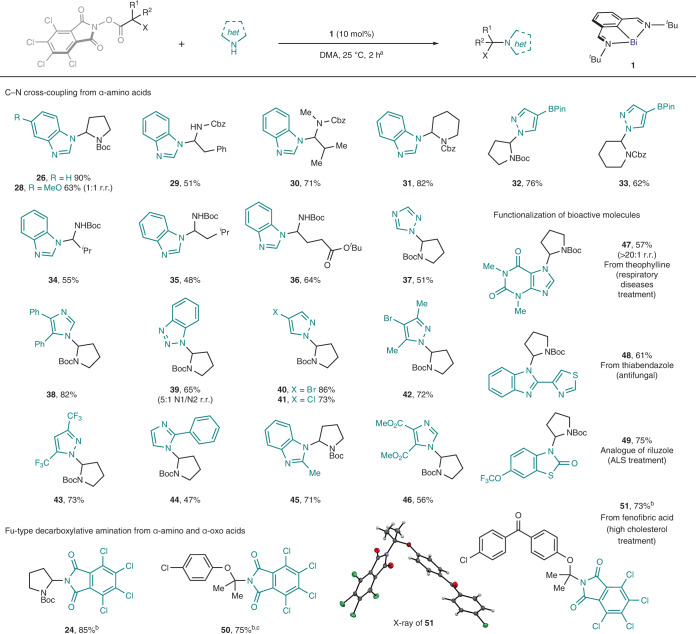
Scope of the C–N coupling reaction

^a^Reaction conditions: RAE (1 equiv., usually 0.2 mmol) and N-nucleophile (3 equiv.) in the presence of bismuthinidene **1** (10 mol%) in DMA (0.033 M) at 25 °C for 2 h. ^b^In the absence of an external N-nucleophile. ^c^DMA/MeCN 1:1 (0.05 M) as solvent. r.r., ratio of regioisomers. R^1^ and R^2^, alkyl groups; X, N or O; Boc, *tert*-butoxycarbonyl; Cbz, benzyloxycarbonyl; BPin, pinacolboryl; ALS, amyotrophic lateral sclerosis.

Non-symmetrical heterocycles such as benzotriazole (to give **39**) could also be accommodated, providing the product in a 5:1 N1/N2 ratio of regioisomers. A range of functional groups with different electronic properties were also tolerated (to give **43**, **46** and **50**). Since the radical process is orthogonal to classical transition-metal-catalysed cross-coupling reactions, different heteroaryl halides (to give **40**–**42**) and heteroaryl boronic esters (to give **32** and **33**) could be well tolerated. The strategy was successfully applied in the modification of bioactive molecules, such as theophylline (to give **47**, 57%, single regioisomer). The successful coupling using thiabendazole (to give **48**, 61%) provides another illustrative example of the orthogonal reactivity to transition metals, as the Lewis-basic sites on both starting material and product could inhibit catalysis by binding to a metal centre. Interestingly, carbamate-like N‒H bonds could also be accommodated as demonstrated by the preparation of **49**, a hydroxylated analogue of riluzole. In the absence of external nucleophiles, the product of decarboxylative amination via formal CO_2_ extrusion was obtained. For this process, both α-amino RAEs and α-oxo RAEs reacted, giving hemiaminal-ether structures such as **50** and **51** in good yields. Overall, this strategy is complementary to the photochemical protocol reported by Fu and co-workers^30^, allowing the use of α-heteroatom RAEs instead of unbiased alkyl substrates.

To shed light on the mechanism, we monitored the catalytic reaction of α-amino RAE **22** by NMR with 10 mol% of **1** at −40 °C, using DMF-d_7_ as the solvent. In this scenario, the pair of rotamers of α-amino alkyl-bismuth(III) intermediate **23** accumulated upon consumption of the RAE, coexisting with bismuthinidene **1**. It is important to mention that complex **23** was characterized by reaction of **1** with **22** in a separate stoichiometric experiment (see Supplementary Information for details). The accumulated **23** decays into **1** after 1 h at −20 °C (Fig. 4, bottom, Bi(I/II/III) pathway). However, we observed that the consumption of RAE **22** to give decarboxylative-amination product **24** occurs at a higher rate than that of the former process (see Supplementary Information for details of kinetic analysis). Thus, an alternative pathway should be considered in which the corresponding in-cage radical pair reacts directly through SET, leading to the iminium cation upon regeneration of Bi(I) (Fig. 4, bottom, Bi(I/II) pathway)^46^. Alternatively, radical recombination of the aforementioned radical pair leads to some accumulation of **23**, which eventually collapses into the reaction product (**24**) and **1**. Overall, the radical oxidative addition appears to be the rate-limiting step of the dominant pathway, as suggested by the continuous presence of **1** throughout the entire course of the reaction. Importantly, low-temperature EPR-spectroscopy analysis allowed us to detect an intense single-line signal, in agreement with the presence of the corresponding α-amino alkyl-radical fragment. This strong EPR signal was observed even in the dark. This is consistent with the fact that the Bi(I/II) pair can promote this reactivity in the absence of external light irradiation.

## Conclusions

In summary, we have developed a radical oxidative addition of redox-active carbon electrophiles to low-valency bismuth, based on the SET from a well-defined Bi(I) complex to alkyl RAEs and KSs, allowing us to merge one- and two-electron reactivity in a single main-group element platform. This process led to a family of alkyl-bismuth(III) compounds, which were found to behave as equilibrium complexes with the corresponding in-cage radical pair formed by bismuth(II) and a free alkyl radical. Unbiased alkyl-bismuth(III) complexes are stable and can be characterized both in solution and in the solid state. On the other hand, α-amino alkyl-bismuth(III) intermediates collapse back into bismuth(I) upon releasing iminium cations, which can be trapped by external N-nucleophiles. This led to the development of a bismuth-catalysed C–N cross-coupling reaction, using complex N-heterocyclic compounds. This new type of radical catalysis is promoted by bismuth in an autonomous manner, through a radical Bi(I/II) or Bi(I/II/III) redox cycle, without the need for a photoredox system, a chemical oxidant, an external base or an electrochemical set-up. Overall, these findings open up a field of radical couplings by a main-group element and pave the way for the design of synthetically relevant transformations based on Bi radical catalysis.

## Methods

### General procedure for the stoichiometric oxidative additions

Unless otherwise specified, a Schlenk flask with a magnetic stirring bar was charged, in an argon-filled glovebox, with bismuth complex **1** (1 equiv.) and the corresponding electrophile (1*–*2 equiv.). Both materials were dissolved in dry and degassed MeCN or THF (0.05 M) and the mixture was stirred until full discolouration of the characteristic dark green colour of bismuth complex **1** was observed, giving a homogeneous yellow/orange solution. After removal of the solvent in high vacuum, the corresponding oxidative-addition adduct was obtained as a pale yellow/orange air-sensitive solid. For characterization purposes, the same reactions can also be conducted directly in dry and degassed MeCN-d_3_ or THF-d_8_.

### General procedure for the C–N coupling reaction

Unless otherwise specified, a 10 ml screw-cap vial with a magnetic stirring bar was charged with an RAE (1 equiv., usually 0.2 mmol) and the corresponding nucleophile (3 equiv.). The vial was placed in an argon-filled glovebox, where bismuth complex **1** (10 mol%) was added. Finally, everything was dissolved in anhydrous DMA (0.033 M). Then the vial was closed, taken outside the glovebox and stirred for 2 h at room temperature. After this time, the mixture was diluted in water and EtOAc. Then the organic fraction was washed twice with water and once with brine, dried over anhydrous Na_2_SO_4_, filtered and concentrated in vacuum. Finally, the product was purified using flash column chromatography or preparative thin-layer chromatography in silica gel.

## Online content

Any methods, additional references, Nature Portfolio reporting summaries, source data, extended data, supplementary information, acknowledgements, peer review information; details of author contributions and competing interests; and statements of data and code availability are available at 10.1038/s41557-023-01229-7.

### Supplementary information

Supplementary Information This file includes all experimental data, details of the procedures, synthesis and characterization of all new compounds, NMR spectra, HRMS data, EPR data, electrochemical data, X-ray crystallographic data, mechanistic experiments, kinetic data, optimization details and further complementary reactivity studies.

Supplementary Data 1 Crystallographic data for compound 9; CCDC reference 2178464.

Supplementary Data 2 Crystallographic data for compound 11; CCDC reference 2178465.

Supplementary Data 3 Crystallographic data for compound 51; CCDC reference 2178503.

## Data Availability

The Supplementary Information contains all experimental procedures and analytical data (^1^H NMR, ^19^F NMR, ^13^C NMR, HRMS and crystallographic data) for all new compounds. Electrochemical, EPR and kinetic data are also included, together with details of the optimization and mechanistic investigations. Crystallographic data for compounds **9** (CCDC 2178464), **11** (CCDC 2178465) and **51** (CCDC 2178503) can be downloaded free of charge from the Cambridge Crystallographic Data Centre www.ccdc.cam.ac.uk.
