# Supplementary material for: Bismuth radical catalysis in the activation and coupling of redox-active electrophiles
Source: Nat Chem. 2023 Jun 1;15(8):1138–45. doi: 10.1038/s41557-023-01229-7 (PMC10396954; doi:10.1038/s41557-023-01229-7)
Supplement: Supplementary file 1 — This file includes all experimental data, details of the procedures, synthesis and characterization of all new compounds, NMR spectra, HRMS data, EPR data, electrochemical data, X-ray crystallographic data, mechanistic experiments, kinetic data, optimization details and further complementary reactivity studies. [file 41557_2023_1229_MOESM1_ESM.pdf]

nature chemistry

Article

https://doi.org/10.1038/s41557-023-01229-7

Bismuth radical catalysis in the activation and coupling of redox-active electrophiles

In the format provided by the authors and unedited

Supporting Information

1. General considerations 2
2. Gram-scale synthesis of bismuthinidene 3
3. Preparation of starting materials 6
   3.1. General procedure A for the preparation of redox-active esters 6
   3.2. Characterization of new redox-active esters 7
4. Stoichiometric studies and reactivity of the alkyl-bismuth(III) complexes 17
   4.1. Synthesis and characterization of the alkyl-bismuth(III) complexes 17
   4.2. Solid-state structure of alkyl bismuth(III) complexes 24
   4.3. Electrochemical data 26
   4.4. EPR spectroscopy 31
   4.5. Other reaction pathways of alkyl-bismuth(III) adducts 38
   4.6. Stoichiometric reactivity of the alkyl-bismuth(III) complexes 45
   4.7. Radical reactions promoted by catalytic bismuth and light 52
5. Development and optimization of the C–N coupling reaction 54
6. Bismuth-catalyzed C–N coupling reaction 61
   6.1. General procedure B for the C–N coupling reactions 61
   6.2. Characterization data for the C–N coupling products 62
   6.3. Variable temperature NMR of representative products 76
7. Mechanistic experiments for the C–N coupling reaction 78
8. Crystal data and structure refinement 84
9. NMR Spectra 99
10. References 183

1. General considerations

Unless otherwise stated, all manipulations were performed under argon using standard Schlenk-line techniques or in an argon-filled glovebox.

Instruments

NMR data were recorded using a Bruker AVIII HD 300 MHz, Bruker AVIII HD 400 MHz, Bruker AVIII 500 MHz or Bruker AVNeo 600 MHz NMR spectrometer (at 298-300 K, unless stated otherwise). ^{1}H and ^{13}C chemical shifts are reported in ppm relative to the solvent residual peaks as an internal reference. For ^{1}H NMR the following residual proton peaks of the deuterated solvents were used: $CDCl_3$, $\delta_H(CHCl_3)$ 7.260; THF-d_8, $\delta_H((CD_2)_3CHDO)$ 3.580; CD_3CN, $\delta_H(CHD_2CN)$ 1.940; DMSO-d_6, $\delta_H(CD_2HSOCD_3)$ 2.500; DMF-d_7, $\delta_H(CHON(CD_3)_2)$ 8.03. For ^{13}C NMR: $CDCl_3$, δ 77.16; THF-d_8, δ 67.57; CD_3CN, δ 1.32; DMSO-d_6, δ 39.52; DMF-d_7, δ 163.15/34.89/29.76. ^{13}C spectra were acquired with broadband ^{1}H decoupling unless mentioned otherwise. ^{19}F NMR shifts are reported relative to the ^{19}F resonances of $CFCl_3$. Chemical shifts (δ) are given in ppm, relative to deuterated solvent residual peak, and coupling constants (*J*) provided in Hz. Mass spectra were acquired using the following instruments: (EI): Finnigan MAT 8200 (70 eV), ESI-MS: ESQ 3000 (Bruker). Accurate mass determinations: Bruker APEX III FT-MS (7 T magnet) or MAT 95 (Finnigan). Melting points were recorded on a Büchi melting point apparatus, Model B-540 (Büchi, Switzerland). FT-IR spectra were recorded on an Alpha Platinum ATR (Bruker) instrument; wavenumbers (ṽ) are given in cm^{-1}; most medium and weak resonances were omitted. Chromatographic purifications were performed by flash column chromatography using Merck silica gel 60 (40-63 µm) or by preparative TLC using PLC Silica gel 60 F_{254}, 1 mm, 20x20 cm (Sigma-Aldrich). Blue-light irradiation was performed with a 465 nm LED strip.

Solvents and reagents

Anhydrous solvents were distilled from appropriate drying agents (THF and THF-d_8, Na/benzophenone, MeCN and MeCN-d_3, CaH_2 or P_2O_5), and stored over 3 or 4 Å molecular sieves under argon prior to use. MeCN and MeCN-d_3 were degassed through 3 freeze-pump-thaw cycles prior to use. 3 and 4 Å molecular sieves were activated at 200 °C under high vacuum for 3 days. Anhydrous DMA and DMF-d_7 were purchased from Sigma-Aldrich, stored inside the glovebox and used as received without further treatment. Unless otherwise stated, solvents used for the preparation of the ligands, catalysts or starting materials were also anhydrous, but not degassed nor stored over molecular sieves. Anhydrous $BiCl_3$ (99.9%, trace metal basis) was purchased from Alfa Aesar and stored in the glovebox.

Unless otherwise noted, all reagents were obtained from commercial suppliers and used without further purification.

2. Gram-scale synthesis of bismuthinidene

The key bismuthinidene used in this study (**1**) was prepared by scaling up a reported procedure, in two steps from the corresponding aryl bromide **S1**.[1,2]

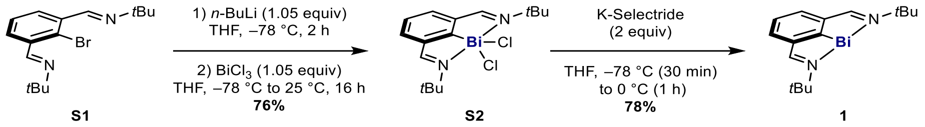

Dichlorobismuthine **S2**: A heat gun-dried 100 mL Schlenk flask with a magnetic stirring bar was charged with (1*E*,1'*E*)-1,1'-(2-bromo-1,3-phenylene)bis(*N*-tert-butylmethanimine) (**S1**) (1.10 g, 3.40 mmol, 1 equiv), and it was dissolved in dry THF (50 mL, 0.07 M). The mixture was cooled down to –78 °C in an acetone/dry ice bath, and to this mixture was added *n*-BuLi dropwise (2.5 M in hexanes, 1.43 mL, 1.05 equiv). The resulting solution was stirred at –78 °C for 2 h. After this time, this solution was transferred in a single portion via cannula to a 250 mL Schlenk flask containing a solution of $BiCl_3$ (1.13 g, 3.57 mmol, 1.05 equiv) in dry THF (30 mL, 0.12 M) under Ar, also pre-cooled to –78 °C. After stirring for 5 min at -78 °C, the cooling bath was removed and the mixture was allowed to stir at room temperature for 16 h. After this time a yellow suspension was obtained, and the rest of the procedure was carried out under air. THF was removed in vacuum, and the obtained residue was re-suspended in 100 mL of HPLC-grade dichloromethane. This was filtered through a plug of Celite, which was washed with 2x100 mL of dichloromethane. The volume of dichloromethane was reduced to ca. 50 mL in vacuum, and then, 50 mL of hexanes were added. A precipitate was obtained, which was filtered through a filtering plate, washed twice (2x25 mL) with pentane, and dried in high vacuum, to afford pure **S2** (1.35 g, 76%) as a white solid. **^{1}H NMR** (300 MHz, $CDCl_3$) δ 9.61 (s, 2H), 8.17 (d, *J* = 7.5 Hz, 2H), 7.85 (dd, *J* = 7.8, 7.2 Hz, 1H), 1.60 (s, 18H).[1,2]

Bismuthinidene **1**: A heat gun-dried 100 mL Schlenk flask with a magnetic stirring bar was charged with **S2** (1.20 g, 2.29 mmol, 1 equiv), which was dissolved under argon in anhydrous THF (30 mL, 0.075 M), and cooled down to –78 °C in an acetone/dry ice bath. To this mixture was added dropwise K-Selectride (1.0 M in THF, 4.59 mL, 2 equiv) and the resulting dark green mixture was stirred at –78 °C for 30 min and then warmed to 0 °C and stirred for 1 h. Then, the flask was taken out of the bath and THF was removed in vacuum. The crude was redissolved in anhydrous pentane, and filtered through a heat gun-dried glass funnel under argon, into another Schlenk flask. The volume of the filtrate was reduced to ca. 15 mL in vacuum, and the solution was cooled to –20 °C overnight. After this time, **1** was obtained as dark green crystalline needles, from which the mother liquor was removed via cannula before drying in high vacuum. The product (0.81 g, 78%) was stored in an argon-filled glovebox. **^{1}H NMR** (300 MHz, THF) δ 9.81 (s, 2H), 7.94 (d, *J* = 7.4 Hz, 2H), 7.08 (t, *J* = 7.4 Hz, 1H), 1.57 (s, 18H).[1,2]

Phebox and MeO-Phebox bismuthinidenes (used for comparative purposes in the optimization) were prepared according to a reported procedure.[3]

New bismuthinidene **S5** (employed for comparison purposes, electrochemical analysis, and cross-over experiments) was prepared following a similar procedure from the corresponding bis-imine aryl bromide, prepared from condensation of the corresponding bis-aldehyde with cyclohexylamine.

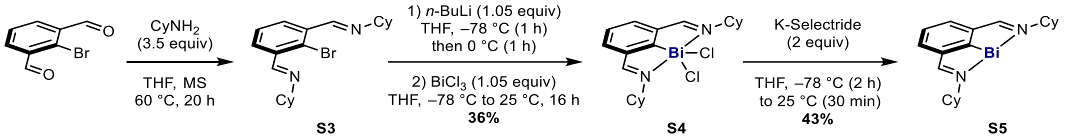

(*E*)-1-(2-Bromo-3-((*E*)-(cyclohexylimino)methyl)phenyl)-N-cyclohexylmethanimine (**S3**). A 250 mL two-necked round-bottomed flask with a magnetic stirring bar and a reflux condenser was charged, under argon, with 2-bromoisophthalaldehyde (3.0 g, 14.1 mmol, 1 equiv), cyclohexylamine (4.9 g, 49 mmol, 3.5 equiv) and activated 3 Å molecular sieves. To this were added 80 mL of dry THF, and the resulting mixture was stirred at 60 °C for 20 h. After cooling down to room temperature, the mixture was filtered through a pad of celite, which was washed with another 100 mL of THF. After removing the solvent and drying the resulting solid in high vacuum for 2 days (to remove residual cyclohexylamine), 5.05 g of the desired bisimine **S3** (95%) was obtained as a pale orange solid. The product was used in the next step without further purification. **^{1}H NMR** (300 MHz, $CDCl_3$) δ 8.74 (s, 2H), 8.01 (d, *J* = 7.7 Hz, 2H), 7.32 (t, *J* = 7.7 Hz, 1H), 3.30 (tt, *J* = 10.3, 4.1 Hz, 2H), 1.90 – 1.51 (m, 16H), 1.45 – 1.24 (m, 6H). **^{13}C NMR** (75 MHz, $CDCl_3$) δ 157.63, 135.60, 130.67, 127.42, 126.75, 69.80, 34.28, 25.58, 24.66.

Biscyclohexyl dichlorobismuthine **S4**: A heat gun-dried 100 mL Schlenk flask with a magnetic stirring bar was charged with (*E*)-1-(2-Bromo-3-((*E*)-(cyclohexylimino)methyl)phenyl)-N-cyclohexylmethanimine (**S3**) (1.60 g, 4.26 mmol, 1 equiv), and it was dissolved in dry THF (50 mL). The mixture was cooled down to –78 °C in an acetone/dry ice bath, and to this mixture was added *n*-BuLi dropwise (2.5 M in hexanes, 1.79 mL, 1.05 equiv). The resulting solution was stirred at –78 °C for 1 h. After this time, the solution was warmed up to 0 °C, and stirred at this temperature for 1 h. Then, this solution was transferred in a single portion via cannula to a 250 mL Schlenk flask containing a solution of $BiCl_3$ (1.41 g, 4.48 mmol, 1.05 equiv) in dry THF (30 mL, 0.12 M) under Ar at –78 °C. After stirring for 30 min at –78 °C, the cooling bath was removed and the mixture was allowed to stir at room temperature for 20 h. After this time a yellow suspension was obtained, and the rest of the procedure was carried out under air. THF was removed in vacuum, and the obtained residue was redisolved in 100 mL of HPLC-grade dichloromethane. This was filtered through a plug of Celite, which was washed with 2x100 mL of dichloromethane. The volume of dichloromethane was reduced to ca. 30 mL in vacuum, and then, 30 mL of hexanes were added. A precipitate was obtained, which was filtered through a filtering plate, washed twice (2x25 mL) with pentane, and dried in high vacuum, to afford pure **S4** (0.90 g, 36%) as a white solid. **^{1}H NMR** (600 MHz, THF) δ 9.70 (d, *J* = 1.0 Hz, 2H), 8.15 (d, *J* = 7.5 Hz, 2H), 7.76 (t, *J* = 7.5 Hz, 1H), 3.63 (ttd, *J* = 11.2, 4.2, 1.1 Hz, 2H), 2.13 – 2.05 (m, 4H), 1.93 – 1.85 (m, 4H), 1.73 – 1.70 (m, 6H), 1.42 (tdd, *J* = 16.4, 9.7, 3.4 Hz, 4H), 1.32 (tt, *J* = 12.6, 3.5 Hz, 2H). **^{13}C NMR** (151 MHz, THF) δ 209.82, 170.91, 147.98, 135.98, 129.10, 68.79, 36.17, 26.02, 25.55. **HRMS (ESI)**: calculated for $C_{20}H_{27}BiClN_2$ [M-Cl]$^+$: 539.16613; found: 539.16586.

Biscyclohexyl bismuthinidene **S5**: A heat gun-dried 100 mL Schlenk flask with a magnetic stirring bar was charged with **S4** (0.53 g, 0.92 mmol, 1 equiv), which was dissolved under argon in anhydrous THF (15 mL, 0.075 M), and cooled down to –78 °C in an acetone/dry ice bath. To this mixture was added dropwise K-Selectride (1.0 M in THF, 1.84 mL, 2 equiv) and the resulting dark green mixture was stirred at –78 °C for 30 min and then stirred at room temperature for 1 h. Then, THF was removed in vacuum. The crude was redissolved in ca. 20 mL of anhydrous pentane, and filtered through a heat gun-dried glass funnel under argon, into another Schlenk flask. The volume of the filtrate was reduced to ca. 5 mL in vacuum, and the solution was cooled to –20 °C overnight. After this time, **S5** was obtained as very dark green crystals, from which the mother liquor was removed via cannula before drying in high vacuum. The product (200 mg, 43%) was stored in an argon-filled glovebox. **^{1}H NMR** (600 MHz, THF) δ 9.76 (d, *J* = 0.9 Hz, 2H), 7.92 (d, *J* = 7.4 Hz, 2H), 7.07 (t, *J* = 7.4 Hz, 1H), 3.74 – 3.67 (m, 2H), 2.14 – 2.05 (m, 4H), 1.91 – 1.83 (m, 4H), 1.73 – 1.67 (m, 6H), 1.54 – 1.46 (m, 4H), 1.37 – 1.30 (m, 2H). **^{13}C NMR** (151 MHz, THF) δ 198.14, 166.49, 145.75, 133.82, 122.47, 69.54, 40.89, 26.48, 26.18. **HRMS (ESI)**: calculated for $C_{20}H_{27}BiN_2$ $[M]^+$: 504.19727; found: 504.19735.

3. Preparation of starting materials

3.1. General procedure A for the preparation of redox-active esters

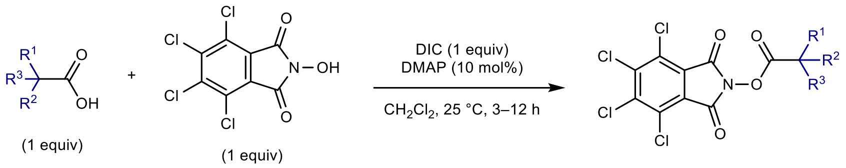

A two-necked round-bottom flask with a magnetic stirring bar was charged with the corresponding carboxylic acid (1.0 equiv), *N*-hydroxytetrachlorophthalimide (1.0 equiv) and 4-dimethylaminopyridine (DMAP, 0.1 equiv). The atmosphere was exchanged to argon through 3 vacuum/argon cycles, before anhydrous dichloromethane (ca. 0.1–0.2 M) was added. Finally, *N*,*N'*-diisopropylcarbodiimide (DIC, 1.0 equiv) was added dropwise via syringe. The mixture was stirred at room temperature until full consumption of the starting acid (usually left 3–12 h). After filtration through Celite, the products were purified by quick flash column chromatography in silica gel, using dichloromethane or dichloromethane/EtOAc 9:1 as eluent. In most cases, a quick filtration through a silica gel plug using dichloromethane or 9:1 dichloromethane/EtOAc afforded the product in >95% purity.

3.2. Characterization of new redox-active esters

The following redox-active esters were employed in this study. Most of the products are known compounds, and characterization data matched the reported ones.[4,5] Characterization data for the new and also for most of the known compounds is reported below.

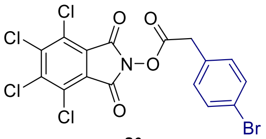
S6

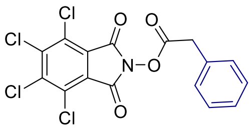
2

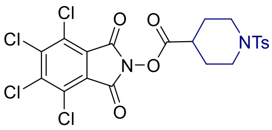
20

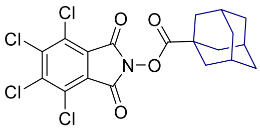
S7

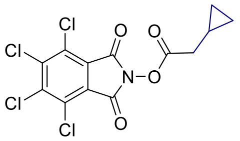
17

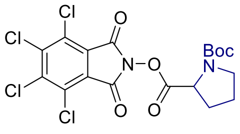
22

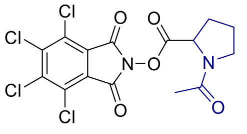
S8

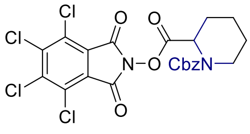
S9

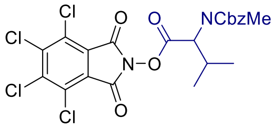
S10

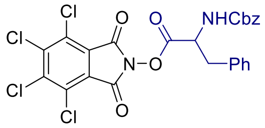
S11

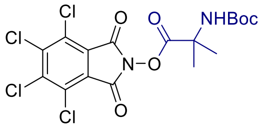
S12

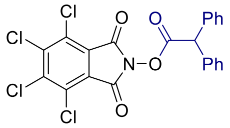
S13

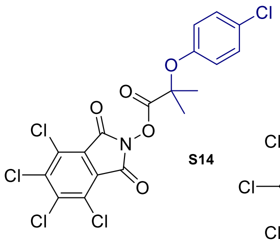
S14

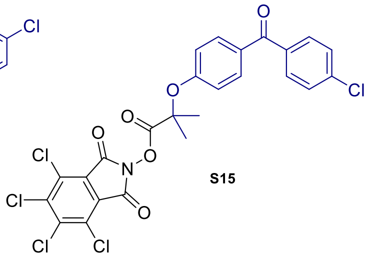
S15

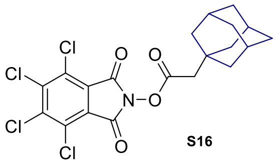
S16

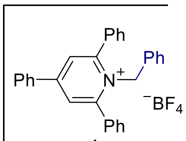
4

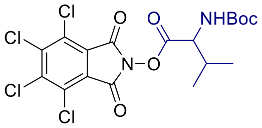
S17

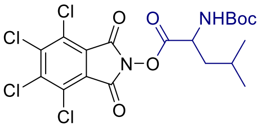
S18

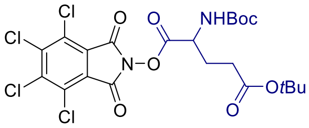
S19

RAE **S14**[5] and benzyl pyridinium salt **4**[6] were prepared according to reported procedures.

4,5,6,7-Tetrachloro-1,3-dioxoisoindolin-2-yl 2-phenylacetate (**2**)

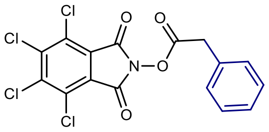

The title product was obtained as a white solid in 51% yield from the corresponding carboxylic acid (3 mmol), following General Procedure A, using dichloromethane as eluent.

^{1}H NMR (400 MHz, $CDCl_3$) δ 7.45 – 7.27 (m, 5H), 3.99 (s, 2H).

^{13}C NMR (101 MHz, $CDCl_3$) δ 167.2, 157.4, 141.0, 131.1, 130.5, 129.2, 128.9, 127.9, 124.6, 37.6.

HRMS (ESI): calculated for $C_{16}H_8NO_4Cl_4$ $[M+H]^+$: 417.92019; found: 417.91951.

FTIR (ATR): $\tilde{\nu}$ [cm^{-1}] = 1817, 1791, 1739, 1376, 1348, 1198, 1150, 1060, 797, 726, 704, 447.

Non-chlorinated analog 1,3-dioxoisoindolin-2-yl 2-phenylacetate (**3**) was prepared according to a similar reported procedure.[7]

4,5,6,7-Tetrachloro-2-(3-cyclopropyl-2-oxopropyl)isoindoline-1,3-dione (**17**)

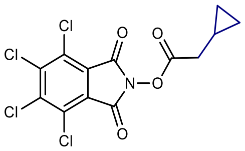

The title product was obtained as a white solid in 45% yield from the corresponding carboxylic acid (1 mmol), following General Procedure A, using dichloromethane as eluent.

^{1}H NMR (600 MHz, $CDCl_3$) δ 2.58 (d, *J* = 7.1 Hz, 2H), 1.21 – 1.14 (m, 1H), 0.72 – 0.65 (m, 2H), 0.33 (dt, *J* = 6.1, 4.9 Hz, 2H).

^{13}C NMR (151 MHz, $CDCl_3$) δ 168.6, 157.7, 141.1, 130.6, 124.8, 36.0, 6.7, 4.8.

HRMS (ESI): calculated for $C_{13}H_7NO_4Cl_4Na$ $[M+Na]^+$: 403.90214; found: 403.90215.

Characterization data matched the ones previously reported for this compound.[4]

4,5,6,7-Tetrachloro-1,3-dioxoisoindolin-2-yl 1-tosylpiperidine-4-carboxylate (**20**)

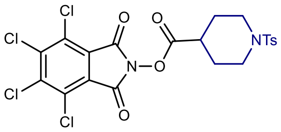

The title product was obtained as a white solid in 85% yield from the corresponding carboxylic acid (5 mmol), following General Procedure A, using dichloromethane/EtOAc (95:5 to 9:1) as eluent.

^{1}H NMR (400 MHz, $CDCl_3$) δ 7.67 – 7.62 (m, 2H), 7.37 – 7.31 (m, 2H), 3.62 (dt, *J* = 12.3, 4.4 Hz, 2H), 2.74 (tt, *J* = 9.9, 4.2 Hz, 1H), 2.65 (ddd, *J* = 12.5, 10.1, 3.1 Hz, 2H), 2.44 (s, 3H), 2.19 – 2.10 (m, 2H), 2.01 (dtd, *J* = 13.8, 10.0, 3.9 Hz, 2H).

^{13}C NMR (101 MHz, $CDCl_3$) δ 169.7, 157.4, 143.8, 141.1, 133.0, 130.5, 129.8, 127.6, 124.6, 44.8, 37.4, 27.2, 21.5.

HRMS (ESI): calculated for $C_{21}H_{16}N_2O_6Cl_4SNa$ $[M+Na]^+$: 586.93754; found: 586.93714.

FTIR (ATR): $\tilde{\nu}$ [cm^{-1}] = 1797, 1744, 1379, 1332, 1159, 1116, 1103, 1036, 923, 865, 802, 730, 651, 580, 552, 471.

1-(*tert*-Butyl) 2-(4,5,6,7-tetrachloro-1,3-dioxoisoindolin-2-yl) pyrrolidine-1,2-dicarboxylate (**22**)

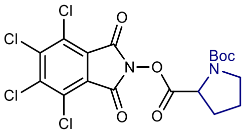

The title product was obtained as an off-white solid in 84% yield from the corresponding carboxylic acid (5 mmol), following General Procedure A, using dichloromethane/EtOAc (95:5) as eluent.

^{1}H NMR (400 MHz, $CDCl_3$, mixture of rotamers) δ 4.74 – 4.59 (m, 1H), 3.66 – 3.54 (m, 1H), 3.53 – 3.40 (m, 1H), 2.51 – 2.30 (m, 2H), 2.14 – 1.94 (m, 2H), 1.56 – 1.48 (m, 9H).

^{13}C NMR (101 MHz, $CDCl_3$, major rotamer) δ 169.2, 157.3, 153.3, 141.0, 130.4, 124.7, 81.2, 57.1, 46.2, 31.4, 28.1, 23.5.

HRMS (ESI): calculated for $C_{18}H_{16}N_2O_6Cl_4Na$ $[M+Na]^+$: 518.96546; found: 518.96532.

FTIR (ATR): $\tilde{\nu}$ [cm^{-1}] = 2976, 1825, 1793, 1744, 1687, 1395, 1366, 1347, 1300, 1197, 1159, 1095, 1057, 1036, 897, 836, 790, 728, 547, 451.

4,5,6,7-Tetrachloro-1,3-dioxoisoindolin-2-yl 2-(4-bromophenyl)acetate (**S6**)

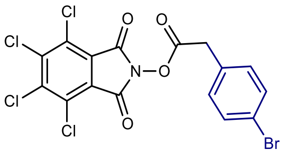

The title product was obtained as a white solid in 77% yield from the corresponding carboxylic acid (3 mmol), following General Procedure A, using dichloromethane as eluent.

^{1}H NMR (400 MHz, $CDCl_3$) δ 7.55 – 7.48 (m, 2H), 7.25 (d, *J* = 6.7 Hz, 2H), 3.95 (s, 2H).

^{13}C NMR (101 MHz, $CDCl_3$) δ 166.7, 157.3, 141.1, 132.1, 130.9, 130.5, 130.1, 124.6, 122.2, 37.1.

HRMS (ESI): calculated for $C_{16}H_6NO_4Cl_4BrNa$ $[M+Na]^+$: 517.81266; found: 517.81191.

FTIR (ATR): $\tilde{\nu}$ [cm^{-1}] = 1817, 1791, 1743, 1488, 1376, 1351, 1299, 1197, 1157, 1076, 1067, 1006, 898, 817, 721, 496, 456.

4,5,6,7-Tetrachloro-1,3-dioxoisoindolin-2-yl adamantane-1-carboxylate (**S7**)

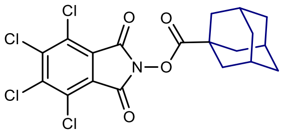

The title product was obtained as a white solid in 77% yield from the corresponding carboxylic acid (3.5 mmol), following General Procedure A, using dichloromethane as eluent.

^{1}H NMR (400 MHz, $CDCl_3$) δ 2.16 – 2.07 (m, 9H), 1.84 – 1.72 (m, 6H).

^{13}C NMR (101 MHz, $CDCl_3$) δ 172.7, 157.7, 140.9, 130.3, 124.8, 40.5, 38.4, 36.1, 27.6.

HRMS (ESI): calculated for $C_{19}H_{15}NO_4Cl_4Na$ $[M+Na]^+$: 483.96474; found: 483.96457.

FTIR (ATR): $\tilde{\nu}$ [cm^{-1}] = 2916, 2856, 1780, 1744, 1364, 1294, 1193, 1167, 1131, 1031, 1005, 965, 857, 726, 695, 602, 472, 409.

4,5,6,7-Tetrachloro-1,3-dioxoisoindolin-2-yl acetylprolinate (**S8**)

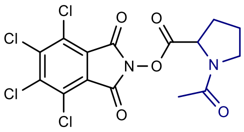

The title product was obtained as an off-white solid in 62% yield from the corresponding carboxylic acid (2 mmol), following General Procedure A, using dichloromethane/EtOAc (8:2) as eluent.

^{1}H NMR (400 MHz, $CDCl_3$, mixture of rotamers) δ 4.86 – 4.75 (m, 1H), 3.76 – 3.65 (m, 1H), 3.64 – 3.53 (m, 1H), 2.54 – 2.30 (m, 2H), 2.26 – 2.05 (m, 5H).

^{13}C NMR (101 MHz, $CDCl_3$, mixture of rotamers) δ 169.7, 169.7, 168.4, 168.3, 159.7, 157.3, 157.1, 141.3, 141.0, 139.6, 130.6, 130.4, 129.4, 125.4, 124.7, 124.5, 58.1, 56.4, 47.5, 46.5, 32.1, 29.6, 24.9, 22.8, 22.3, 22.0.

HRMS (ESI): calculated for $C_{15}H_{11}N_2O_5Cl_4$ $[M+H]^+$: 438.94165; found: 438.94108.

FTIR (ATR): $\tilde{\nu}$ [cm^{-1}] = 2975, 2931, 2889, 1825, 1743, 1687, 1378, 1346, 1300, 1158, 1122, 1094, 1057, 921, 835, 789, 728, 611, 547, 450.

1-Benzyl 2-(4,5,6,7-tetrachloro-1,3-dioxoisoindolin-2-yl) piperidine-1,2-dicarboxylate (**S9**)

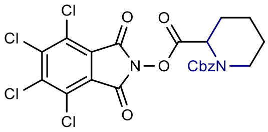

The title product was obtained as a pale-yellow solid in 93% yield from the corresponding carboxylic acid (2 mmol), following General Procedure A, using dichloromethane/EtOAc (9:1) as eluent.

^{1}H NMR (400 MHz, $CDCl_3$, mixture of rotamers) δ 7.42 – 7.26 (m, 5H), 5.50 – 5.25 (m, 1H), 5.23 – 5.12 (m, 2H), 4.23 – 4.09 (m, 1H), 3.22 – 3.03 (m, 1H), 2.37 (t, *J* = 15.6 Hz, 1H), 1.91 – 1.47 (m, 5H).

^{13}C NMR (101 MHz, $CDCl_3$, mixture of rotamers) δ 167.9, 157.3, 156.0, 155.4, 141.1, 141.1 136.3, 136.1, 130.5, 128.5, 128.5, 128.1, 128.1, 127.9, 124.6, 67.8, 53.3, 53.1, 41.9, 41.8, 27.2, 24.6, 24.3, 20.4, 20.3.

HRMS (ESI): calculated for $C_{22}H_{16}N_2O_6Cl_4Na$ $[M+Na]^+$: 566.96546; found: 566.96529.

FTIR (ATR): $\tilde{\nu}$ [cm^{-1}] = 2972, 2945, 2866, 1814, 1792, 1745, 1705, 1404, 1375, 1354, 1237, 1158, 1066, 1032, 921, 725, 697, 606, 464, 421.

4,5,6,7-Tetrachloro-1,3-dioxoisoindolin-2-yl *N*-((benzyloxy)carbonyl)-*N*-methylvalinate (**S10**)

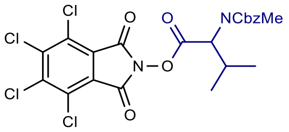

The title product was obtained as a pale-yellow solid in 95% yield from the corresponding carboxylic acid (1 mmol), following General Procedure A, using dichloromethane/EtOAc (95:5) as eluent.

^{1}H NMR (400 MHz, $CDCl_3$, mixture of rotamers) δ 7.40 – 7.28 (m, 5H), 5.29 – 5.16 (m, 2H), 5.00 – 4.66 (m, 1H), 3.00 (s, 3H), 2.31 (dp, *J* = 10.7, 6.6 Hz, 1H), 1.17 – 1.10 (m, 3H), 1.01 – 0.94 (m, 3H).

^{13}C NMR (101 MHz, $CDCl_3$, mixture of rotamers) δ 166.9, 166.7, 157.2, 156.8, 155.8, 141.1, 136.3, 130.5, 128.5, 128.1, 127.9, 127.7, 124.6, 67.9, 62.4, 61.9, 30.5, 30.0, 28.0, 27.7, 19.4, 19.3, 18.8, 18.5.

HRMS (ESI): calculated for $C_{22}H_{18}N_2O_6Cl_4Na$ [M+Na]$^+$: 568.98111; found: 568.98058.

FTIR (ATR): $\tilde{\nu}$ [cm^{-1}] = 2965, 2939, 1817, 1793, 1740, 1705, 1693, 1453, 1379, 1302, 1156, 1037, 982, 923, 792, 746, 727, 697, 589, 448.

4,5,6,7-Tetrachloro-1,3-dioxoisoindolin-2-yl ((benzyloxy)carbonyl)phenylalaninate (**S11**)

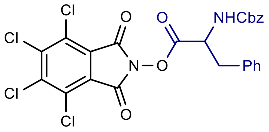

The title product was obtained as a white solid in 62% yield from the corresponding carboxylic acid (1 mmol), following General Procedure A, using dichloromethane/EtOAc (9:1) as eluent.

^{1}H NMR (300 MHz, $CDCl_3$, mixture of rotamers) δ 7.40 – 7.25 (m, 10H), 5.20 – 5.09 (m, 3H), 5.09 – 4.82 (m, 1H), 3.48 – 3.19 (m, 2H).

^{13}C NMR (75 MHz, $CDCl_3$, mixture of rotamers) δ 168.0, 157.0, 155.3, 141.1, 135.8, 134.1, 130.5, 129.5, 128.9, 128.5, 128.3, 128.2, 127.6, 124.6, 67.4, 53.0, 38.0.

HRMS (ESI): calculated for $C_{23}H_{14}N_5O_5Cl_4Na$ [M+Na]$^+$: 602.96412; found: 602.96471.

FTIR (ATR): $\tilde{\nu}$ [cm^{-1}] = 3309, 2949, 1795, 1745, 1699, 1543, 1365, 1266, 1198, 1158, 1046, 730, 699, 618, 495, 409.

4,5,6,7-Tetrachloro-1,3-dioxoisoindolin-2-yl 2-phenylacetate (**S12**)

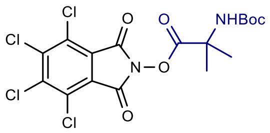

The title product was obtained as an off-white solid in 92% yield from the corresponding carboxylic acid (2 mmol), following General Procedure A, using dichloromethane/EtOAc (9:1) as eluent.

^{1}H NMR (400 MHz, $CDCl_3$) δ 5.14 (s, 1H), 1.67 (s, 6H), 1.51 (s, 9H).

^{13}C NMR (101 MHz, $CDCl_3$) δ 170.7, 157.2, 154.2, 140.9, 130.3, 124.7, 80.8, 55.7, 28.2, 25.6.

HRMS (ESI): calculated for $C_{17}H_{16}N_2O_6Cl_4Na$ $[M+Na]^+$: 506.96547; found: 506.96515.

FTIR (ATR): $\tilde{\nu}$ [cm^{-1}] = 3303, 2981, 1789, 1743, 1688, 1529, 1366, 1288, 1153, 1096, 1070, 1035, 840, 782, 730, 720, 617, 448.

4,5,6,7-Tetrachloro-1,3-dioxoisoindolin-2-yl 2,2-diphenylacetate (**S13**)

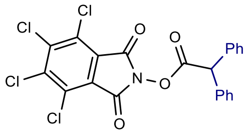

The title product was obtained as a white solid in 91% yield from the corresponding carboxylic acid (5 mmol), following General Procedure A, using dichloromethane as eluent.

^{1}H NMR (300 MHz, $CDCl_3$) δ 7.44 – 7.29 (m, 10H), 5.41 (s, 1H).

^{13}C NMR (75 MHz, $CDCl_3$) δ 168.5, 157.3, 141.0, 136.3, 130.4, 128.9, 128.6, 127.9, 124.6, 53.9.

HRMS (ESI): calculated for $C_{22}H_{11}NO_4Cl_4Na$ $[M+Na]^+$: 515.93344; found: 515.93316.

FTIR (ATR): $\tilde{\nu}$ [cm^{-1}] = 1813, 1790, 1767, 1740, 380, 1352, 1298, 1200, 1157, 1065, 1034, 899, 867, 751, 732, 722, 692, 597, 558, 498, 443.

4,5,6,7-Tetrachloro-1,3-dioxoisoindolin-2-yl 2-(4-(4-chlorobenzoyl)phenoxy)-2-methylpropanoate (**S15**)

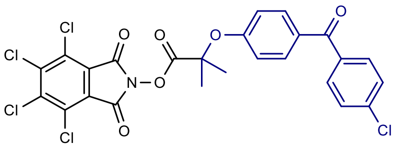

The title product was obtained as a white solid in 83% yield from the corresponding carboxylic acid (5 mmol), following General Procedure A, using dichloromethane/EtOAc (95:5 to 9:1) as eluent.

^{1}H NMR (400 MHz, $CDCl_3$) δ 7.85 – 7.80 (m, 2H), 7.76 – 7.72 (m, 2H), 7.50 – 7.43 (m, 2H), 7.12 – 7.06 (m, 2H), 1.86 (s, 6H).

^{13}C NMR (101 MHz, $CDCl_3$) δ 194.2, 170.0, 158.4, 157.3, 141.3, 138.5, 136.2, 132.1, 131.6, 131.3, 130.6, 128.6, 124.6, 118.4, 78.6, 25.7.

HRMS (ESI): calculated for $C_{25}H_{14}N_1O_6Cl_5Na$ $[M+Na]^+$: 621.91560; found: 621.91462.

FTIR (ATR): $\tilde{\nu}$ [cm^{-1}] = 2991, 1812, 1790, 1742, 1650, 1597, 1573, 1485, 1366, 1285, 1245, 1146, 1067, 924, 842, 762, 725, 658, 590, 522, 480, 432.

4,5,6,7-Tetrachloro-1,3-dioxoisoindolin-2-yl 2-(adamantan-1-yl)acetate (**S16**)

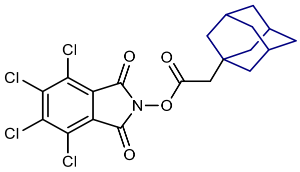

The title product was obtained as a white solid in 86% yield from the corresponding carboxylic acid (3 mmol), following General Procedure A, using dichloromethane as eluent.

^{1}H NMR (400 MHz, $CDCl_3$) δ 2.32 (s, 2H), 1.99 – 1.93 (m, 3H), 1.70 – 1.58 (m, 12H).

^{13}C NMR (101 MHz, $CDCl_3$) δ 165.7, 156.5, 139.9, 129.3, 123.7, 44.2, 41.0, 35.4, 32.3, 27.5.

HRMS (ESI): calculated for $C_{20}H_{17}NO_4Cl_4Na$ $[M+Na]^+$: 497.98039; found: 497.98035.

FTIR (ATR): $\tilde{\nu}$ [cm^{-1}] = 2971, 2909, 2850, 1787, 1746, 1368, 1332, 1188, 1110, 1073, 1028, 888, 793, 732, 722, 691, 588, 458.

4,5,6,7-Tetrachloro-1,3-dioxoisoindolin-2-yl (*tert*-butoxycarbonyl)valinate (S17)

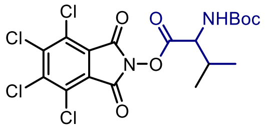

The title product was obtained as a white solid in 87% yield from the corresponding carboxylic acid (1.5 mmol), following General Procedure A, using dichloromethane/EtOAc (9:1) as eluent.

^{1}H NMR (300 MHz, $CDCl_3$, mixture of rotamers) δ 5.14 – 4.72 (m, 1H), 4.70 – 4.29 (m, 1H), 2.33 (tt, *J* = 12.1, 6.1 Hz, 1H), 1.48 (s, 9H), 1.18 – 1.03 (m, 6H).

^{13}C NMR (75 MHz, $CDCl_3$, mixture of rotamers) δ 168.5, 157.1, 155.0, 141.0, 130.5, 124.6, 80.5, 57.1, 31.6, 28.2, 18.7, 17.4.

HRMS (ESI): calculated for $C_{18}H_{18}Cl_4N_2NaO_6$ $[M+Na]^+$: 520.98112; found: 520.98112.

FTIR (ATR): $\tilde{\nu}$ [cm^{-1}] = 3366, 2966, 2933, 1794, 1742, 1697, 1529, 1362, 1277, 1176, 1153, 1070, 1032, 903, 792, 726, 615, 447.

4,5,6,7-Tetrachloro-1,3-dioxoisoindolin-2-yl (*tert*-butoxycarbonyl)leucinate (S18)

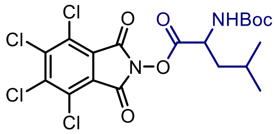

The title product was obtained as a white solid in 78% yield from the corresponding carboxylic acid (1.5 mmol), following General Procedure A, using dichloromethane/EtOAc (9:1) as eluent.

^{1}H NMR (300 MHz, $CDCl_3$, mixture of rotamers) δ 5.10 – 4.86 (m, 1H), 4.81 – 4.41 (m, 1H), 1.98 – 1.78 (m, 2H), 1.77 – 1.67 (m, 1H), 1.07 – 0.97 (m, 6H).

^{13}C NMR (75 MHz, $CDCl_3$, mixture of rotamers) δ 169.5, 157.1, 154.8, 141.0, 130.4, 124.6, 80.6, 60.3, 50.5, 41.6, 28.2, 24.7, 22.6, 21.8, 21.0, 14.1.

HRMS (ESI): calculated for $C_{19}H_{20}Cl_4N_2NaO_6$ $[M+Na]^+$: 534.99677; found: 534.99669.

FTIR (ATR): $\tilde{\nu}$ [cm^{-1}] = 3256, 2962, 2873, 1794, 1746, 1708, 1688, 1472, 1379, 1365, 1153, 1076, 1038, 1009, 788, 722, 582, 436.

5-(*tert*-Butyl) 1-(4,5,6,7-tetrachloro-1,3-dioxoisoindolin-2-yl) (*tert*-butoxycarbonyl)glutamate (S19)

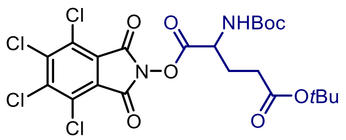

The title product was obtained as a white solid in 72% yield from the corresponding carboxylic acid (1.5 mmol), following General Procedure A, using dichloromethane/EtOAc (9:1) as eluent.

^{1}H NMR (300 MHz, $CDCl_3$) δ 5.26 (d, *J* = 8.3 Hz, 1H), 4.81 – 4.51 (m, 1H), 2.56 – 2.41 (m, 2H), 2.37 – 2.25 (m, 1H), 2.22 – 2.08 (m, 1H), 1.47 (s, 18H).

^{13}C NMR (75 MHz, $CDCl_3$) δ 171.6, 168.6, 157.0, 154.8, 141.0, 130.4, 124.6, 81.1, 80.6, 51.5, 31.1, 28.2, 28.0, 27.4.

HRMS (ESI): calculated for $C_{22}H_{24}Cl_4N_2NaO_8$ $[M+Na]^+$: 607.01790; found: 607.01786.

FTIR (ATR): $\tilde{\nu}$ [cm^{-1}] = 3402, 2980, 2937, 1841, 1798, 1444, 1720, 1698, 1509, 1451, 1379, 1366, 1248, 1145, 1046, 876, 729, 720, 536, 440, 416.

4. Stoichiometric studies and reactivity of the alkyl-bismuth(III) complexes

4.1. Synthesis and characterization of the alkyl-bismuth(III) complexes

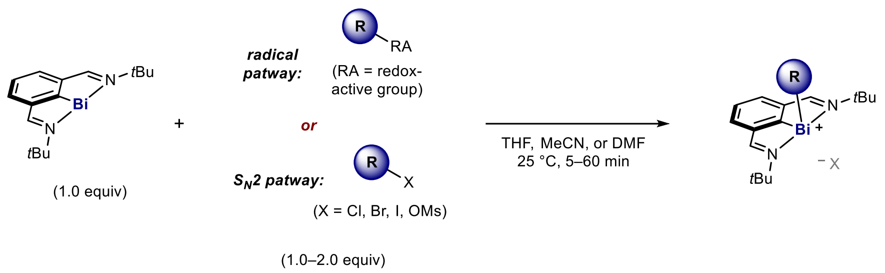

In an argon-filled glovebox, an oven-dried Schlenk flask or culture tube with a magnetic stirring bar was charged with bismuthinidene **1** (1 equiv) and the corresponding electrophile (1.0–2.0 equiv). Both reagents were dissolved in anhydrous MeCN-d_3, THF-d_8 or DMF-d_7 (0.025–0.05 M) for characterization, or in non-deuterated THF (0.035–0.1 M) for preparative scale. The resulting homogeneous solution was stirred at room temperature until full conversion of the starting bismuth(I) complex (which can happen instantly or after ca. 5–60 min, depending on the substrate). Full conversion of bismuth(I) was determined visually, by the disappearance of its dark green color to give yellow or light orange solutions. The resulting solution was analyzed by NMR for characterization purposes. Unless otherwise stated, conversion higher than 95% was observed for the Bi(I) towards the corresponding bismuth(III).

In preparative-scale reactions, the solvent was then removed in vacuum, to give the corresponding alkyl-bismuth(III) complex as a yellow–orange solid, which could be stored under argon without significant decomposition.

Characterization data of the different bismuth(III) complexes

[(2,6-(*t*BuNCH)$_2$C$_6$H$_3$)Bi(benzyl)(chloride)] (**5**)

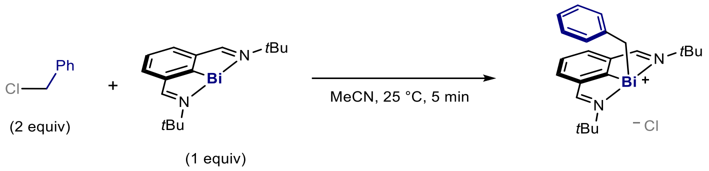

The title compound was obtained from bismuthinidene **1** (0.035 mmol, 1 equiv) and benzyl chloride (0.070 mmol, 2 equiv) in 0.6 mL of MeCN-d_3 (0.058 M) after stirring for 5 min at room temperature (>95% conversion by NMR, together with excess electrophile).

^{1}H NMR (600 MHz, CD$_3$CN) δ 9.72 (s, 2H), 7.97 (d, *J* = 7.5 Hz, 2H), 7.74 (dd, *J* = 7.8, 7.3 Hz, 1H), 6.92 – 6.88 (m, 2H), 6.44 (tt, *J* = 7.4, 1.2 Hz, 1H), 6.10 – 6.06 (m, 2H), 3.00 (s, 2H), 1.50 (s, 18H).

^{13}C NMR (151 MHz, CD$_3$CN) δ 194.9, 169.2, 148.8, 138.4, 136.7, 130.1, 129.9, 127.2, 126.8, 62.0, 59.6, 31.2.

HRMS (ESI Positive): calculated for $C_{23}H_{30}BiN_2$ [M-Cl]$^+$: 543.22075; found: 543.22155.

[(2,6-(*t*BuNCH)$_2$C$_6$H$_3$)Bi(benzyl)(bromide)] (**6**)

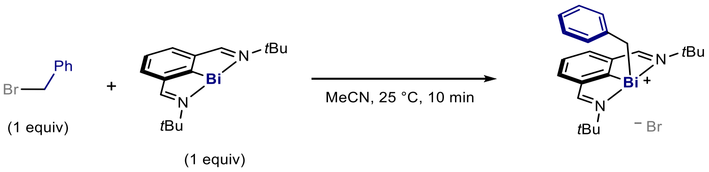

The title compound was obtained from bismuthinidene **1** (0.022 mmol, 1 equiv) and benzyl bromide (0.022 mmol, 1 equiv) in 0.6 mL of MeCN-d_3 (0.04 M) after stirring for 10 min at room temperature (>95% conversion by NMR).

^{1}H NMR (600 MHz, CD$_3$CN) δ 9.71 (s, 2H), 7.97 (d, *J* = 7.5 Hz, 2H), 7.75 (m, 1H), 6.93 – 6.88 (m, 2H), 6.45 (m, 1H), 6.10 – 6.07 (m, 2H), 3.01 (s, 2H), 1.50 (s, 18H).

^{13}C NMR (151 MHz, CD$_3$CN) δ 193.4, 168.8, 148.3, 137.9, 136.4, 129.8, 129.5, 126.8, 126.5, 61.6, 58.7, 30.8.

HRMS (ESI Positive): calculated for $C_{23}H_{30}BiN_2$ [M-Br]$^+$: 543.22075; found: 543.22101.

[(2,6-(*t*BuNCH)$_2$C$_6$H$_3$)Bi(benzyl)(iodide)] (**7**)

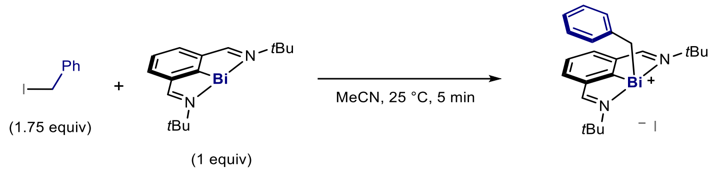

The title compound was obtained from bismuthinidene **1** (0.035 mmol, 1 equiv) and benzyl iodide (0.061 mmol, 1.75 equiv) in 0.6 mL of MeCN-*d*$_3$ (0.04 M) after stirring for 5 min at room temperature (>95% conversion by NMR, together with excess electrophile).

^{1}H NMR (600 MHz, CD$_3$CN) δ 9.71 (s, 2H), 7.97 (d, *J* = 7.5 Hz, 2H), 7.75 (dd, *J* = 7.8, 7.3 Hz, 1H), 6.94 – 6.88 (m, 2H), 6.44 (tt, *J* = 7.4, 1.2 Hz, 1H), 6.11 – 6.06 (m, 2H), 3.01 (s, 2H), 1.50 (s, 18H).

^{13}C NMR (151 MHz, CD$_3$CN) δ 193.7, 169.2, 148.7, 138.3, 136.8, 130.2, 129.9, 127.2, 126.9, 62.0, 59.1, 31.2.

HRMS (ESI Positive): calculated for $C_{23}H_{30}BiN_2$ [M-I]$^+$: 543.22075; found: 543.22093.

[(2,6-(*t*BuNCH)$_2$C$_6$H$_3$)Bi(benzyl)(mesylate)] (**8**)

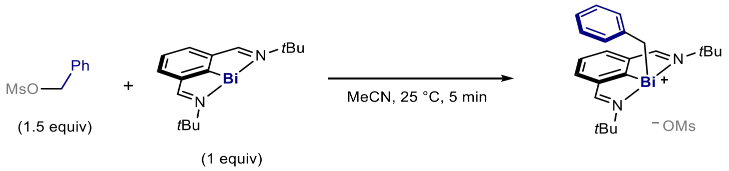

The title compound was obtained from bismuthinidene **1** (0.035 mmol, 1 equiv) and benzyl mesylate (0.053 mmol, 1.5 equiv) in 0.6 mL of MeCN-*d*$_3$ (0.04 M) after stirring for 5 min at room temperature (>95% conversion by NMR, together with excess electrophile).

^{1}H NMR (600 MHz, CD$_3$CN) δ 9.70 (s, 2H), 7.97 (d, *J* = 7.5 Hz, 2H), 7.76 (dd, *J* = 7.8, 7.3 Hz, 1H), 6.93 – 6.87 (m, 2H), 6.45 (tt, *J* = 7.4, 1.2 Hz, 1H), 6.11 – 6.06 (m, 2H), 3.01 (s, 2H), 2.41 (s, 3H), 1.49 (s, 18H).

^{13}C NMR (151 MHz, CD$_3$CN) δ 193.3, 169.3, 148.8, 138.2, 136.9, 130.3, 129.9, 127.3, 126.9, 62.0, 58.6, 39.9, 31.1.

HRMS (ESI Positive): calculated for $C_{23}H_{30}BiN_2$ [M-OMs]$^+$: 543.22075; found: 543.22095.

[(2,6-(*t*BuNCH)$_2$C$_6$H$_3$)Bi(benzyl)(tetrachlorophthalimide)] (**9**)

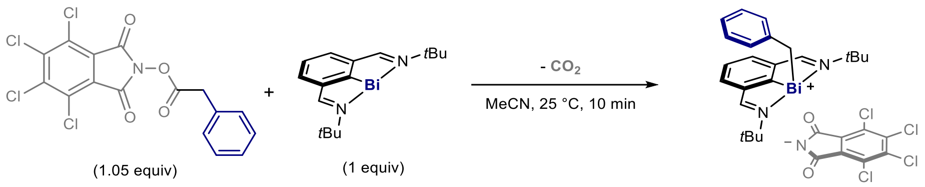

The title compound was obtained from bismuthinidene **1** (0.022 mmol, 1 equiv) and the corresponding redox-active ester **2** (0.023 mmol, 1.05 equiv) in 0.6 mL of MeCN-*d₃* (0.04 M, for characterization) or THF (0.04 M, for crystallization) after stirring for 10 min at room temperature (>95% conversion by NMR). X-ray quality single crystals were obtained by layering with pentane a solution of the product in THF in an NMR tube, which was left in the freezer (-30 °C) for 4 days.

^{1}H NMR (600 MHz, CD$_3$CN) δ 9.66 (s, 2H), 7.87 (d, *J* = 7.5 Hz, 2H), 7.65 (dd, *J* = 7.7, 7.3 Hz, 1H), 6.89 – 6.82 (m, 2H), 6.40 (tt, *J* = 7.4, 1.2 Hz, 1H), 6.06 – 6.00 (m, 2H), 2.95 (s, 2H), 1.48 (s, 18H).

^{13}C NMR (151 MHz, CD$_3$CN) δ 193.3, 168.9, 148.4, 138.2, 136.4, 129.9, 129.7, 127.0, 126.7, 61.8, 58.2, 31.0.

HRMS (ESI Positive): calculated for $C_{23}H_{30}BiN_2$ [M-TCPhth]$^+$: 543.22075; found: 543.22075.

[(2,6-(*t*BuNCH)$_2$C$_6$H$_3$)Bi(benzyl)(tetrafluoroborate)] (**10**)

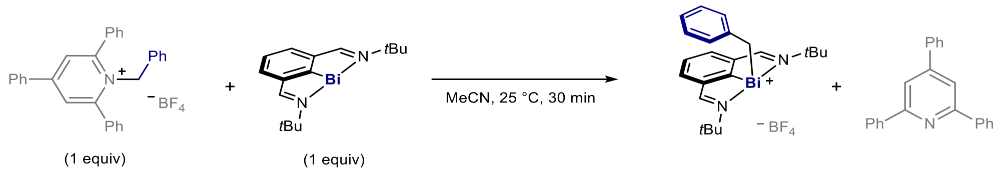

The title compound was obtained from bismuthinidene **1** (0.022 mmol, 1 equiv) and pyridinium salt **4** (0.022 mmol, 1 equiv) in 0.6 mL of MeCN-*d₃* (0.04 M) after stirring for 30 min at room temperature (>95% conversion by NMR). After drying in vacuum, most triphenylpyridine could be removed by washing with hexane/toluene (20:1), but the obtained residue still contained ca. 25% of this side product.

^{1}H NMR (600 MHz, CD$_3$CN) δ 9.68 (s, 2H), 7.96 (d, *J* = 7.5 Hz, 2H), 7.76 (dd, *J* = 7.8, 7.3 Hz, 1H), 6.99 – 6.84 (m, 2H), 6.53 – 6.40 (m, 1H), 6.12 – 6.02 (m, 2H), 3.01 (s, 2H), 1.48 (s, 18H).

^{13}C NMR (151 MHz, CD$_3$CN) δ 192.8, 169.3, 148.8, 138.2, 136.9, 130.4, 129.9, 127.4, 126.9, 62.0, 58.4, 31.2.

^{19}F NMR (565 MHz, CD$_3$CN) δ -151.68.

HRMS (ESI Positive): calculated for $C_{23}H_{30}BiN_2$ [M-BF$_4$]$^+$: 543.22075; found: 543.22073.

[(2,6-(*t*BuNCH)$_2$C$_6$H$_3$)Bi(1-adamantylmethyl)(tetrachlorophthalimide)] (**12**)

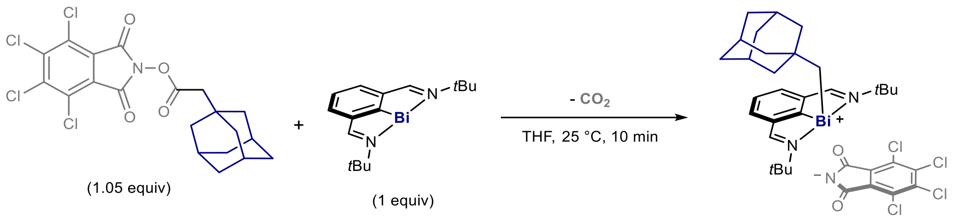

The title compound was obtained from bismuthinidene **1** (0.05 mmol, 1 equiv) and the corresponding redox-active ester **S16** (0.053 mmol, 1.05 equiv) in 1 mL of THF-d_8 (0.05 M) after stirring for 10 min at room temperature (>95% conversion by NMR).

^{1}H NMR (600 MHz, THF-d_8) δ 9.76 (s, 2H), 8.13 (d, *J* = 7.5 Hz, 2H), 7.66 (t, *J* = 7.5 Hz, 1H), 2.04 (s, 2H), 1.92 – 1.88 (m, 3H), 1.62 (s, 18H), 1.61 – 1.57 (m, 3H), 1.48 – 1.41 (m, 9H).

^{13}C NMR (151 MHz, THF-d_8) δ 190.8, 178.6, 169.8, 149.9, 136.5, 136.0, 134.9, 129.2, 126.0, 80.5, 62.2, 49.1, 37.1, 34.6, 31.6, 30.3.

HRMS (ESI Positive): calculated for $C_{27}H_{40}BiN_2$ [M-TCPhth]$^+$: 601.29900; found: 601.29949.

[(2,6-(*t*BuNCH)$_2$C$_6$H$_3$)Bi(1-adamantylmethyl)(iodide)] (**14**)

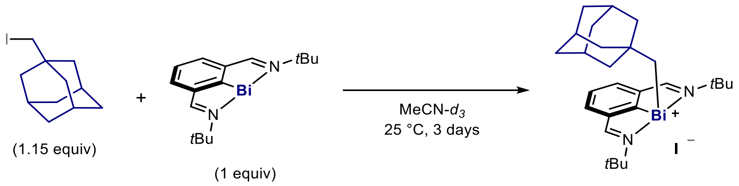

The title compound was obtained from bismuthinidene **1** (0.05 mmol, 1 equiv) and adamantylmethyl iodide (0.058 mmol, 1.15 equiv) in 1 mL of MeCN-d_3 (0.05 M) after stirring for 3 days at room temperature (>95% conversion by NMR). Alternatively, the reaction took 30 h to reach full conversion using THF as solvent.

^{1}H NMR (600 MHz, CD$_3$CN) δ 9.73 (s, 2H), 8.26 (d, *J* = 7.5 Hz, 2H), 7.96 (dd, *J* = 7.7, 7.4 Hz, 1H), 2.18 (s, 2H), 1.92 – 1.88 (m, 3H), 1.61 – 1.57 (m, 3H), 1.51 (s, 18H), 1.43 – 1.39 (m, 3H), 1.34 (dd, *J* = 2.4, 1.5 Hz, 6H).

^{13}C NMR (151 MHz, CD$_3$CN) δ 185.92, 170.32, 149.73, 137.41, 130.66, 118.25, 77.98, 62.30, 48.35, 36.76, 35.05, 31.65, 30.03.

HRMS (ESI Positive): calculated for $C_{27}H_{40}BiN_2$ [M-I]$^+$: 601.29900; found: 601.29922.
(ESI Negative), found: 126.9, iodide counteranion.

We found that for sterically challenging electrophiles (such as adamantylmethyl iodide), this new approach via SET results in drastically faster oxidative additions than classical polar reactivity via S$_N$2, which appears to be sensitive to steric effects.

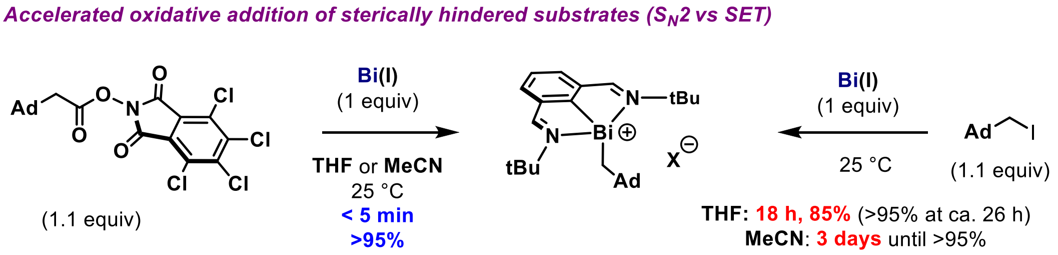

[(2,6-(*t*BuNCH)$_2$C$_6$H$_3$)Bi(*p*-bromobenzyl)(tetrachlorophthalimide)] (11)

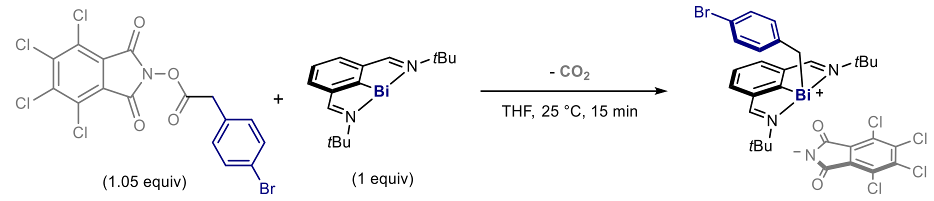

The title compound was obtained in preparative scale from bismuthinidene **1** (175 mg, 0.39 mmol, 1 equiv) and the corresponding redox-active ester **S6** (202 mg, 0.41 mmol, 1.05 equiv) in 6 mL of THF (0.06 M) after stirring for 15 min at room temperature, as a light orange solid (>95% conversion by NMR, 93% isolated product yield after drying and transferring, 326 mg).

^{1}H NMR (600 MHz, THF-*d$_8$*) δ 9.72 (s, 2H), 7.91 (d, *J* = 7.5 Hz, 2H), 7.66 – 7.60 (m, 1H), 6.99 – 6.94 (m, 2H), 6.08 – 5.94 (m, 2H), 2.88 (s, 2H), 1.55 (s, 18H).

^{13}C NMR (151 MHz, THF-*d$_8$*) δ 203.0, 174.2, 168.6, 149.4, 139.6, 136.6, 136.1, 134.0, 131.6, 129.2, 129.1, 127.3, 120.0, 62.7, 62.1, 31.1.

HRMS (ESI Positive): calculated for $C_{23}H_{29}BiBrN_2$ [M-TCPhth]$^+$: 621.13126; found: 621.13157.
(ESI Negative): calculated for $C_8Cl_4NO_2$ [TCPhth]$^-$: 281.86886; found: 281.86883.

[(2,6-(*t*BuNCH)$_2$C$_6$H$_3$)Bi(1-tosylpiperidin-4-yl)(tetrachlorophthalimide)] (13)

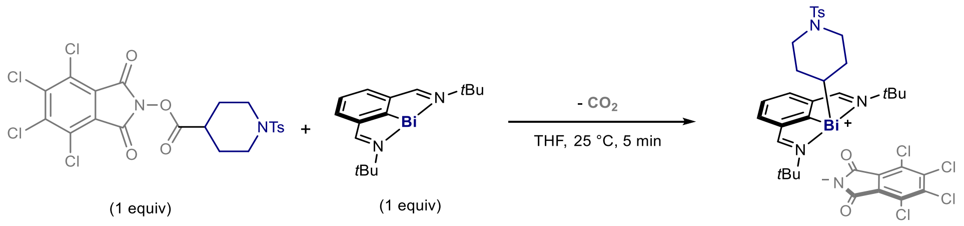

The title compound was obtained in preparative scale from bismuthinidene **1** (50 mg, 0.11 mmol, 1 equiv) and the corresponding redox-active ester **22** (63 mg, 0.11 mmol, 1 equiv) in 2 mL of THF (0.05 M) after stirring for 5 min at room temperature, as a yellow solid (>95% conversion by NMR, 87% isolated product yield after drying and transferring, 326 mg).

^{1}H NMR (400 MHz, THF-*d$_8$*) δ 9.85 (s, 2H), 8.14 (d, *J* = 7.5 Hz, 2H), 7.74 (t, *J* = 7.5 Hz, 1H), 7.51 – 7.46 (m, 2H), 7.31 – 7.25 (m, 2H), 3.93 – 3.86 (m, 2H), 2.42 – 2.29 (m, 6H), 2.07 (qd, *J* = 13.3, 4.5 Hz, 2H), 1.82 (td, *J* = 11.8, 2.1 Hz, 2H), 1.55 (s, 18H).

^{13}C NMR (101 MHz, THF-*d$_8$*) δ 196.7, 168.7, 149.4, 143.4, 136.2, 134.4, 129.7, 129.0, 128.0, 74.3, 61.7, 55.2, 31.9, 30.7, 20.9.

HRMS (ESI Positive): calculated for $C_{28}H_{39}BiN_3O_2S$ [M-TCPhth]$^+$: 690.25615; found: 690.25658.

4.2. Solid-state structure of alkyl bismuth(III) complexes

The structure of [(2,6-(*t*BuNCH)$_2$C$_6$H$_3$)Bi(benzyl)(tetrafluoroborate)] (**9**) was fully confirmed by x-ray crystal diffraction analysis.

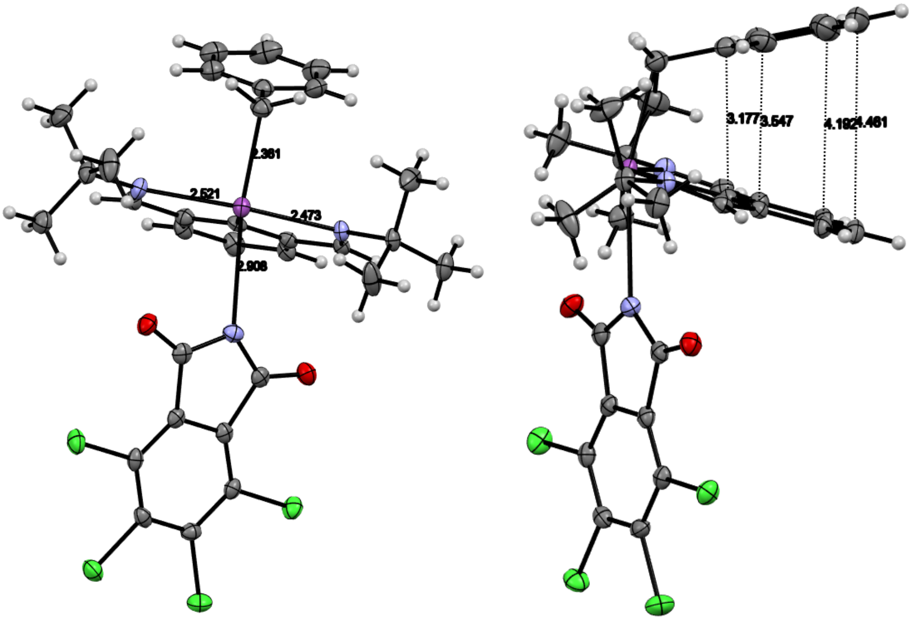

Some attractive interactions between the benzyl substituent and the aromatic backbone of the ligand are observed. These can be the cause of the significant shift upfield of the ^{1}H NMR aromatic signals of this substituent (between 6.0 and 6.9 ppm), when comparing it with a regular benzyl group (ca. 7.4 ppm).

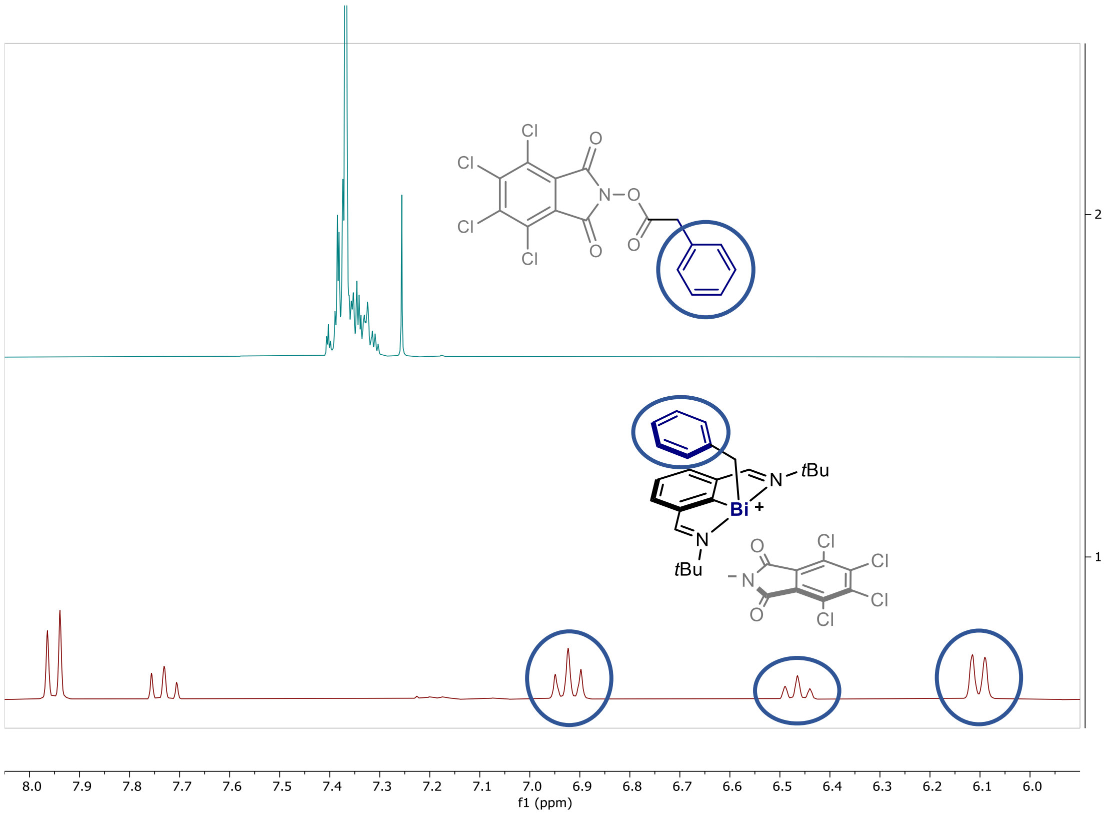

Furthermore, the structure of [(2,6-(tBuNCH)$_2$C$_6$H$_3$)Bi(p-bromobenzyl)(tetrachlorophthalimide)] (**11**) was also confirmed in solid state

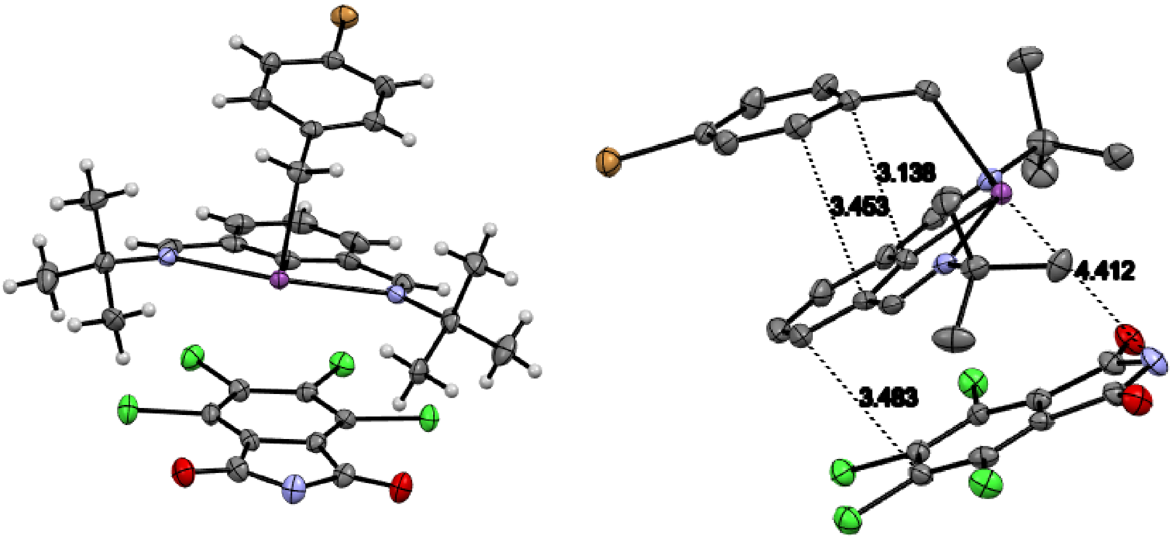

A similar type of π-stacking interactions between the 4-bromobenzyl group and the ligand backbone were observed (even stronger, considering the corresponding bond distances; also by ^{1}H NMR, two of the aromatic signals of this substituent go slightly below 6 ppm).

A different behavior of the tetrachlorophthalimide anion is observed: in this case, in solid state, it acts as external counteranion, not directly bound to the metal center, resulting in a cationic bismuth(III) complex.

In spite of this divergent behavior in the solid state, the broad/weak ^{13}C signals observed by NMR corresponding to the C atoms of the phthalimide for all these complexes suggest that, in solution, both the cationic and the N-coordinated species could be in equilibrium.

4.3. Electrochemical data

Cyclic voltammograms were collected using 3-electrode cell consisting of a 3 mm glassy carbon working electrode, platinum wire as the counter electrode, and silver wire as the reference at ambient temperature in an argon-filled glovebox equipped with electrochemical outlets. Sublimed ferrocene was added at the end of each experiment as the internal reference.

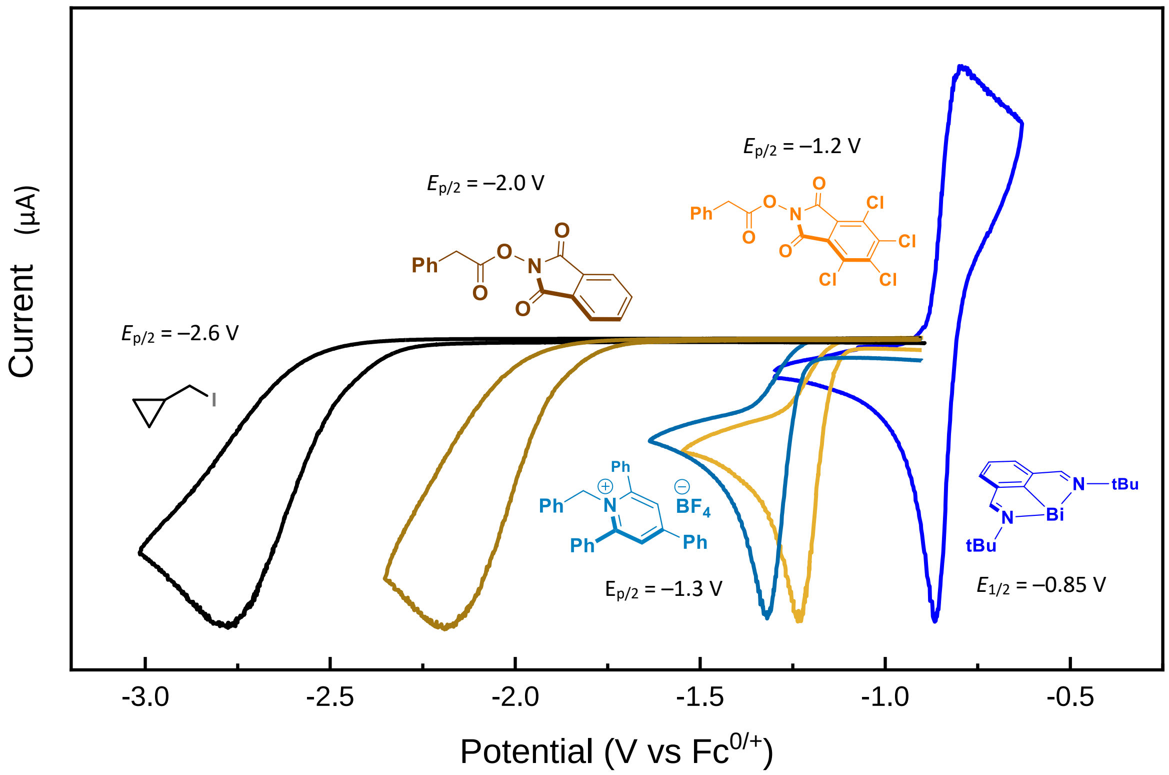

Cyclic voltammetry in CH_3CN, scan rate 100 mV/s vs Fc/Fc^+

A reversible oxidation peak corresponding to the Bi(I)/(II) redox step was observed at –0.85 V for bismuthinidene **1** vs Fc/Fc^+, with an onset potential at ca. –1.0 V. Due to the reversibility of the redox wave, and that the potential difference between **1** and **2** is ca. 0.25 V, SET between **1** and **2** can be estimated to be ca. 5 kcal/mol uphill, but subsequent release of CO_2 can drive the oxidative addition process. Thus, we consider **1** to be able to reduce electrophiles with a redox potential up to –1.2 V.

CVs of tetrachlorophthalimide derivative **2** and pyridinium salts **4** gave half-peak potentials of –1.2 V and –1.3 V vs Fc/Fc^+, respectively. This is consistent with the fact that these substrates undergo SET-oxidative addition into bismuth(I).

Non-chlorinated phthalimide derivative **3** showed a half-peak potential of –2.1 V vs Fc/Fc^+, consistent with it not being reactive with bismuthinidene **1**.

Furthermore, a non-SET-redox-active electrophile such as cyclopropylmethyl iodide shows half-peak potentials of –2.6 V vs Fc/Fc^+, supporting the fact that the fast oxidative addition of alkyl halides to Bi(I) occurs through a polar S_N2 mechanism.

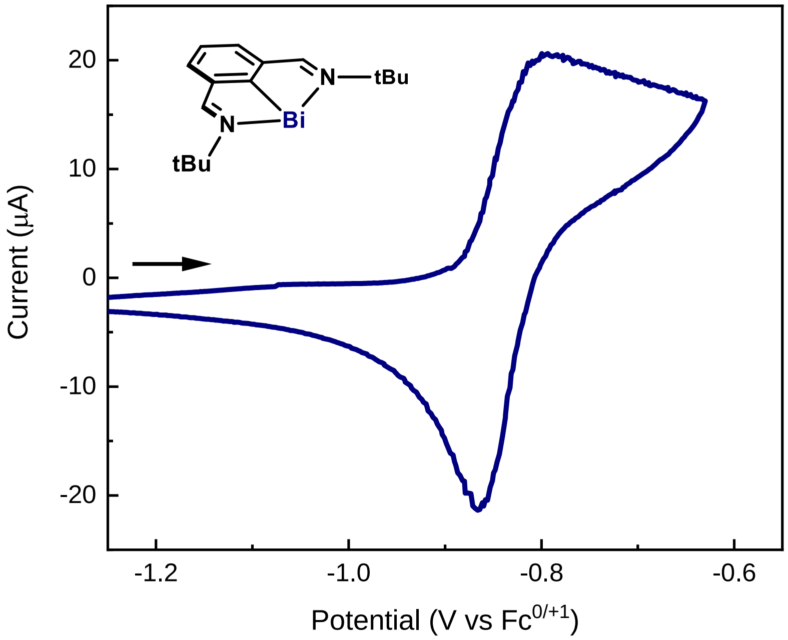

Cyclic voltammogram of bismuthinidene **1** in CH_3CN using 0.1 M [^{n}Bu_4N][PF_6] as supporting electrolyte at ambient temperature; scan rate: 100 mV/s, referenced to $Fc^{0/+}$.

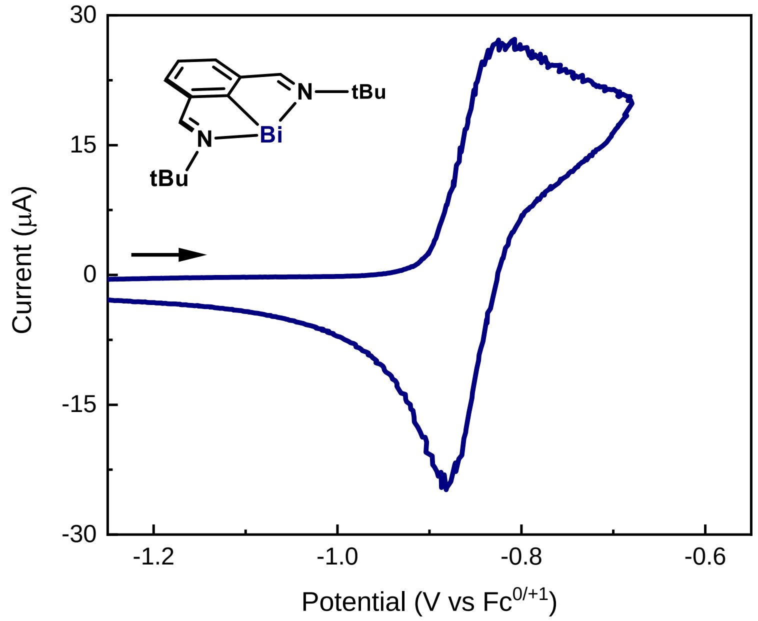

Cyclic voltammogram of bismuthinidene **1** in CH_3CN using 0.1 M [^{n}Bu_4N][BAr^F_4] as supporting electrolyte at ambient temperature; scan rate: 100 mV/s, referenced to $Fc^{0/+}$.

No significant counteranion effect on the redox potential was observed.

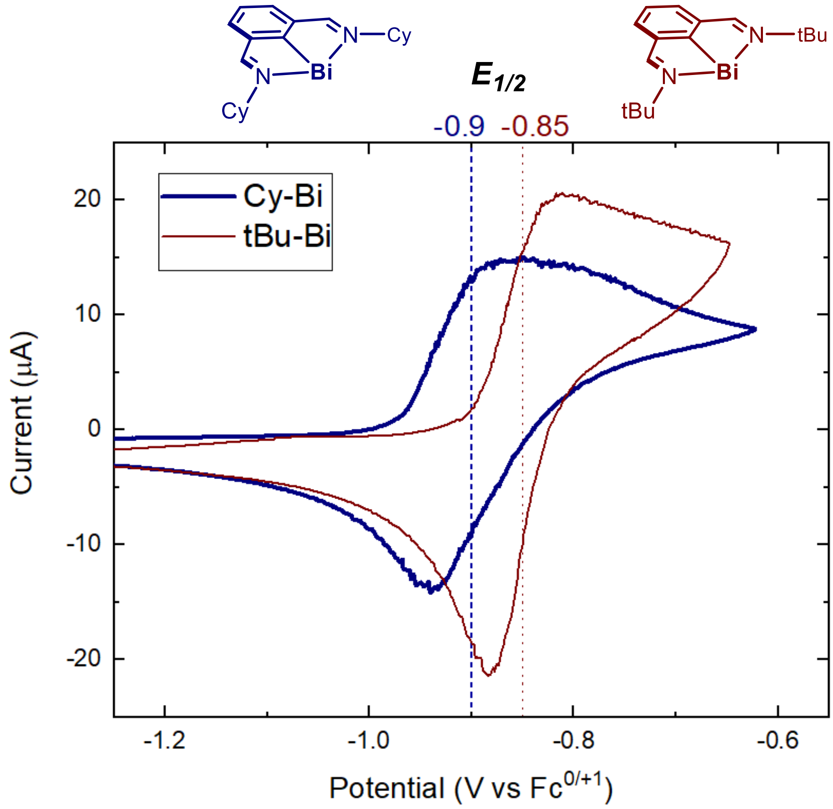

Cyclic voltammogram of bismuthinidene **S5** (blue line) compared to that of **1** (red line) in CH_3CN using 0.1 M $[^{n}Bu_4N][PF_6]$ as supporting electrolyte at ambient temperature; scan rate: 100 mV/s, referenced to $Fc^{0/+}$.

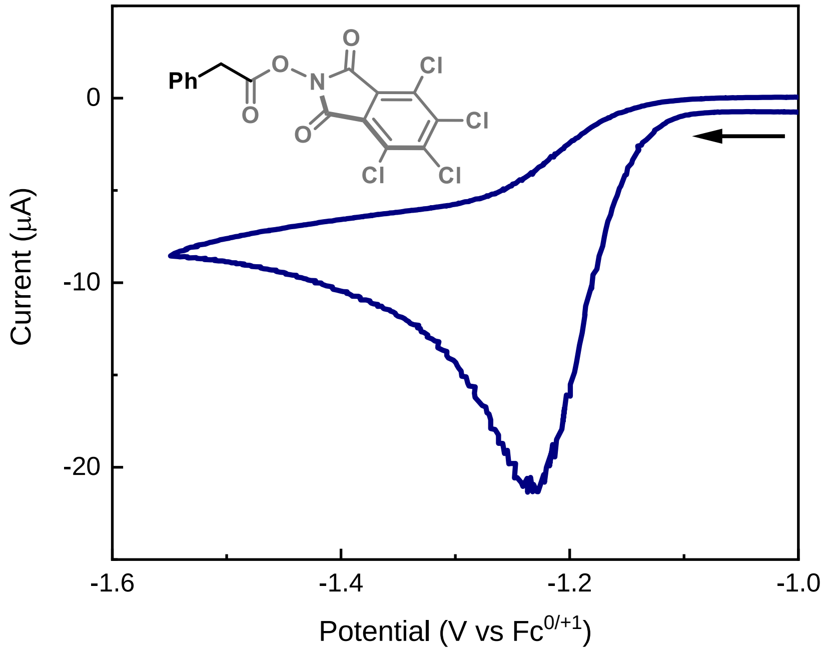

Cyclic voltammogram of tetrachlorophthalimide derivative **2** in CH_3CN using 0.1 M [^{n}Bu$_4$N][PF$_6$] as supporting electrolyte at ambient temperature; scan rate: 100 mV/s, referenced to $Fc^{0/+}$.

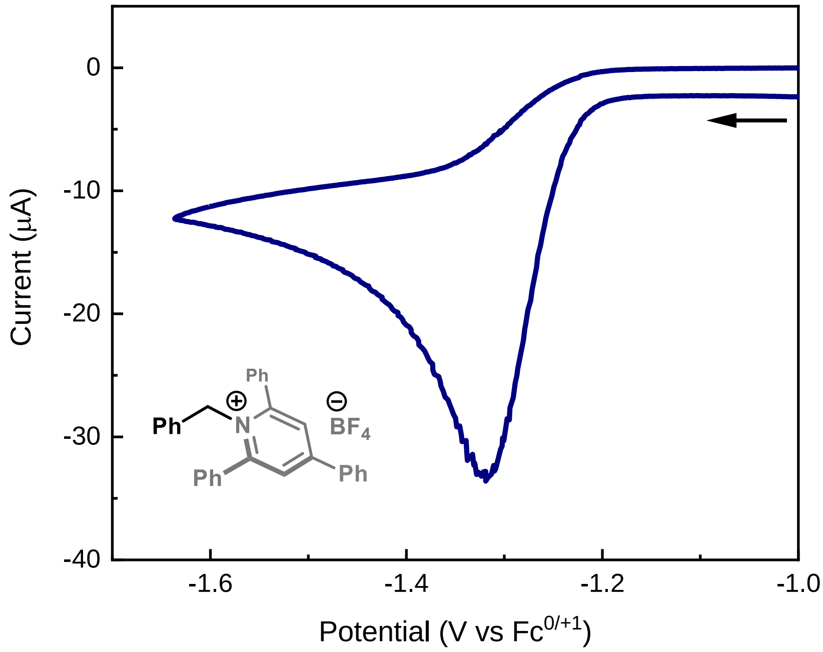

Cyclic voltammogram of pyridinium salt **4** in CH_3CN using 0.1 M [^{n}Bu$_4$N][PF$_6$] as supporting electrolyte at ambient temperature; scan rate: 100 mV/s, referenced to $Fc^{0/+}$.

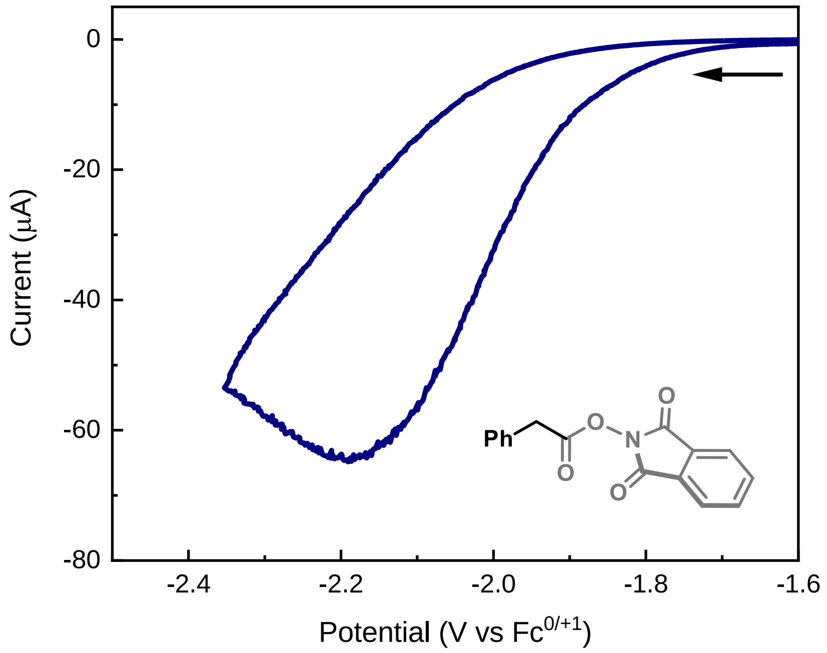

Cyclic voltammogram of non-chlorinated phthalimide derivative **3** in CH_3CN using 0.1 M [^{n}Bu$_4$N][PF$_6$] as supporting electrolyte at ambient temperature; scan rate: 100 mV/s, referenced to Fc$^{0/+}$.

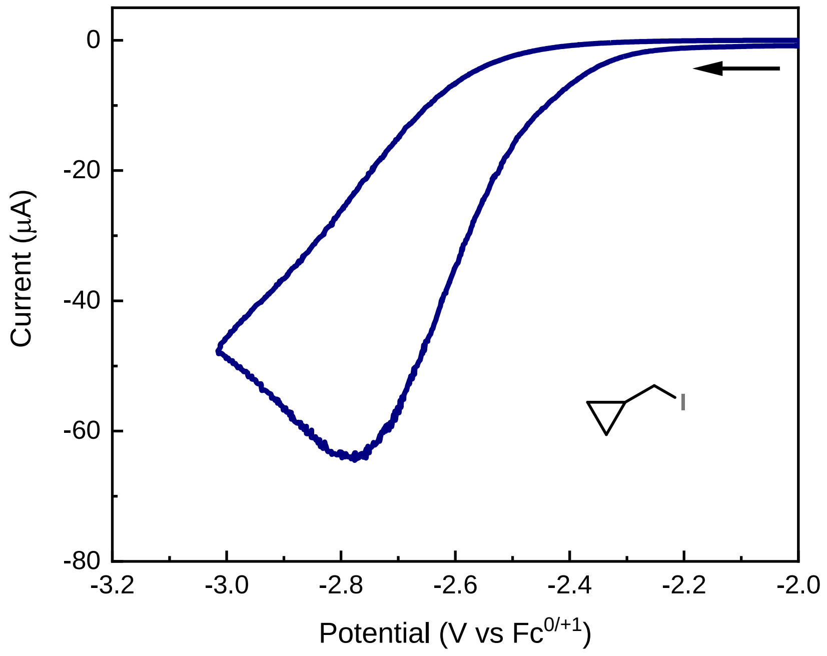

Cyclic voltammogram of cyclopropylmethyl iodide in CH_3CN using 0.1 M [^{n}Bu$_4$N][PF$_6$] as supporting electrolyte at ambient temperature; scan rate: 100 mV/s, referenced to Fc$^{0/+}$.

4.4. EPR spectroscopy

General considerations

EPR was recorded on a Bruker Elexsys E500 CW EPR spectrometer equipped with a ER4116DM resonator and an Oxford ESR900 cryostat at the corresponding temperature, or a Bruker Magnettech MS5000 desktop EPR instrument with a N_2 cooling system.

A laser-pumped white light source LS-WL1 (lightsource.tech, Göttingen) was connected to the optical port of the resonator using a 1 mm optical fiber and a 5 mm collimator and turned on for the experiments under illumination. The light intensity reaching the sample tube was estimated to be of the order of 50 mW.

General procedure for sample preparation

In an argon-filled glovebox, an oven-dried culture tube or glass vial was charged with bismuthinidene **1** (1 equiv) and the corresponding electrophile (1 equiv). The solids were dissolved in dry and degassed THF (0.2–0.3 M) and the reaction mixture was stirred until it turned yellow (ca. 5 min for redox-active esters). The solution of bismuth(III) complex was filtered through an oven-dried HPLC filter into an EPR tube (2.8 or 5 mm), the tube was closed, taken out of the glovebox, and introduced in the EPR instrument at the corresponding temperature.

Summary and aim of the EPR study

An EPR study of several unbiased alkyl-bismuth(III) complexes (**9**, **12** and **13**) was carried out at different temperatures, both in the absence of light or under white-light irradiation. Unless stated otherwise, the EPR spectra in the dark of these species were silent and are not included here. Under light irradiation, some accumulation of two different radical species can observed and recorded by EPR. Although very small structural information can be extracted from these spectra, they are included in the following pages, as a proof of light-induced formation of radicals from complexes **9**, **12** and **13**. This is in alignment with other experimental evidence gathered during the study of these compounds (e.g.: radical-clock or other radical-trapping experiments), which do seem to be more reactive under light irradiation.

On the other hand, when analyzing the catalytic reaction (in which α-amino-alkyl groups are employed instead), a very strong signal is observed in the dark, which does not increase in intensity upon illumination. This supports the formation and significant accumulation of α-amino-alkyl radical species, which are key intermediates in the catalytic CN coupling promoted by low valent bismuth.

In order to study the alkyl-radical character of these complexes, and to detect possible radical intermediates, we carried out an EPR study, analyzing five different alkyl-bismuth(III) complexes, at different temperatures, both in the dark and under light irradiation.

First, we analyzed four different complexes at room temperature under illumination. Two compounds showed a three line EPR spectrum which disappeared when the light was switched off. During repeated illumination experiments, the intensity of the triplet signal decreased. This indicates that the signal is associated with a radical intermediate that accumulates significantly to be detected under illumination. The three line spectrum is consistent with a carbon centered radical showing a hyperfine coupling with the ^{14}N nuclear spin (I = 1) of a neighboring nitrogen atom (splitting of 2.1 Gauss). This could be explained by a Bi–C homolysis, and localization of corresponding (L)(TCPhthal)Bi(II) radical into either the bisimine backbone or the phthalimide ligand in the Bi(II) radical complex.

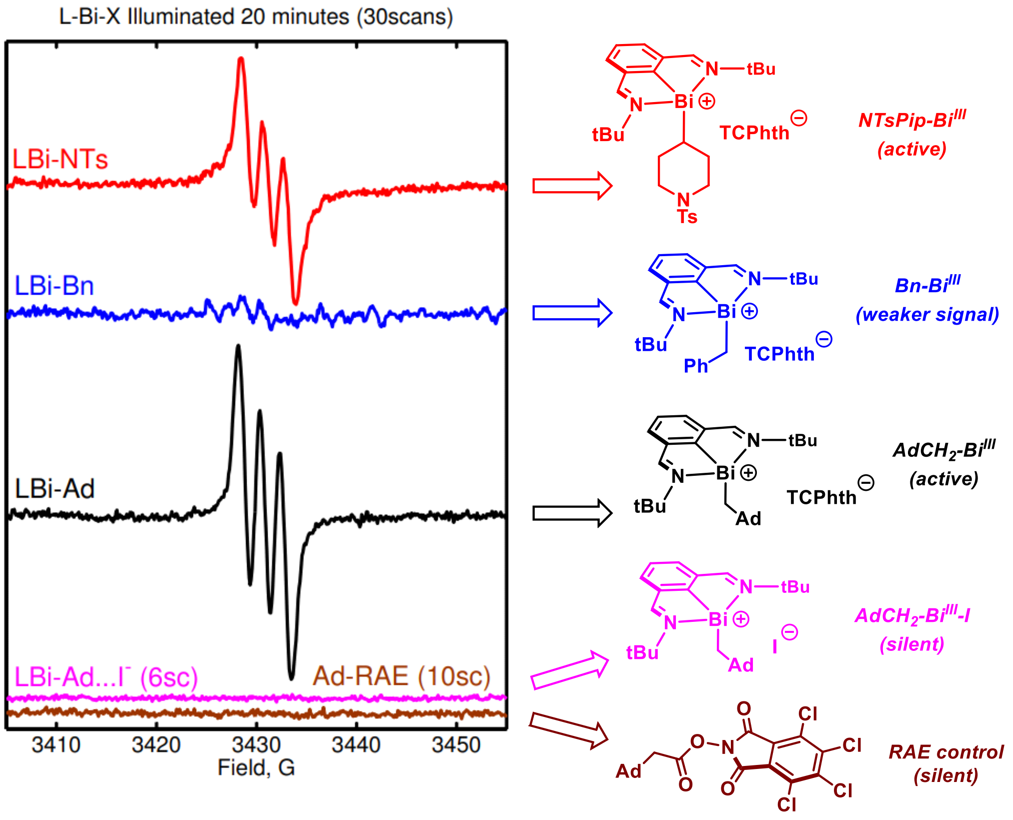

Compounds **13**, **9**, **12**, **14** and **S16** (from top to bottom) in THF under white light illumination. The spectra were recorded at room temperature with 2mW Power and 1 Gauss modulation amplitude, 30 scans (20 min).

Complexes **12** and **13** showed an intense signal, whereas benzyl derivative **9** gave a very weak response at room temperature (presumably due to the higher instability of this complex under light irradiation, see next section for details). Complex **14**, obtained by S_N2 reaction with adamantylmethyl iodide, was found to be EPR-silent, highlighting the importance of the counteranion in the homolysis/radical character of the complexes. Finally, a control experiment with the corresponding redox-active ester under illumination showed a silent EPR spectrum.

Then, we analyzed adamantylmethyl complex **12** in more detail, at lower temperatures, using a Bruker Magnettech MS5000 desktop EPR instrument with a N_2 cooling system allowing lowering the temperature slowly from room temperature down to –60 °C. Even though the signal/noise ratio was much smaller at this temperature, a triplet signal centered at 336.6 mT was still observed (splitting of 2.4 Gauss) but, in addition a single line at g=2.004 (centered at 337.1 mT) was formed under illumination suggesting the generation of a carbon centered radical. Under these conditions, the carbon-radical seems to be slightly more persistent than the N-coupled radical: it could be observed after switching off the light at 213 K, whereas the triplet disappears immediately at this temperature.

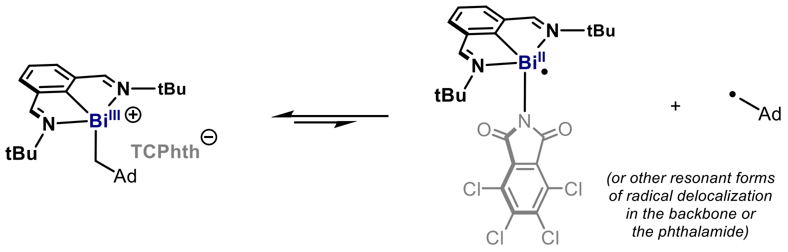

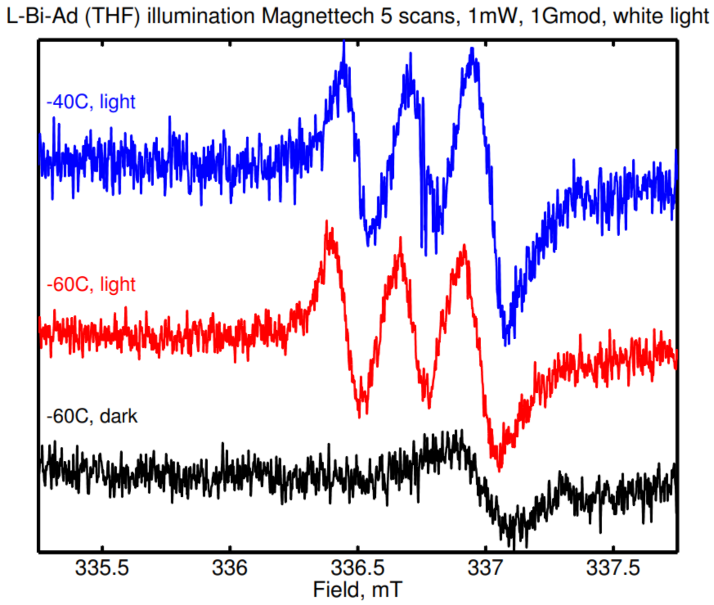

Compound **12** in THF under white light illumination. The spectra were recorded at 233 K (–40 °C) and 213 K (–60 °C) with 1mW Power and 1 Gauss modulation amplitude, 5 scans.

Note: The EPR experiments presented herein concluded that radical species can indeed be generated from alkyl-bismuth(III) complexes such as **9**, **12** and **13**. No further structural information could be extracted, and structures as the one proposed in the scheme above are purely speculative at this point.

Subsequently, we measured the same complex **12** at 123 K (–150 °C), as a frozen THF solution. Before illumination, no signal was detected. After light irradiation, two different radical species could be detected: an anisotropic multiline spectrum consistent with a ^{14}N hyperfine interaction is observed. A more intense, presumably C-centered single line radical signal is also observed at 337.5 mT.

Upon switching off the light a significant decay of the radical species was observed over the course of several minutes. Noteworthy, the two signals seem to decay at different rates, as judged by qualitative analysis of the spectra at different times, confirming the existence of two different radical species.

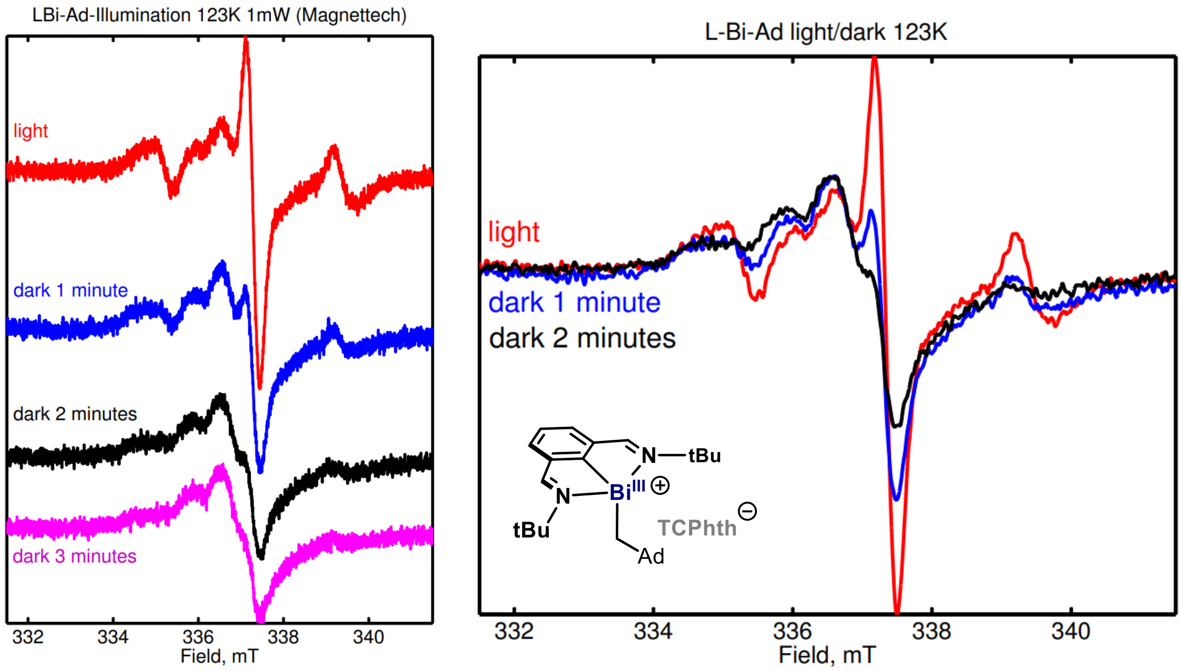

Compound **12** in frozen THF under white light illumination and decay of the radical species after switching off the light. The spectra were recorded at 123 K (–150 °C) with 1mW Power and 3 Gauss modulation amplitude, 5 scans.

Then, we analyzed by EPR compounds **12**, **13** and **9** in frozen THF under light illumination at 75 K, obtaining similar signals (though slightly shifted when changing the alkyl fragment, as one would expect). Even benzyl-bismuth(III) **9** gave a significant response at low temperature, whereas at room temperature almost no radical species could be detected.

Unlike at 123 K, after switching off the light at 75 K the signals remained unchanged for all samples over the course of >10 min, showing that the radical species are relatively persistent at this temperature in frozen solution.

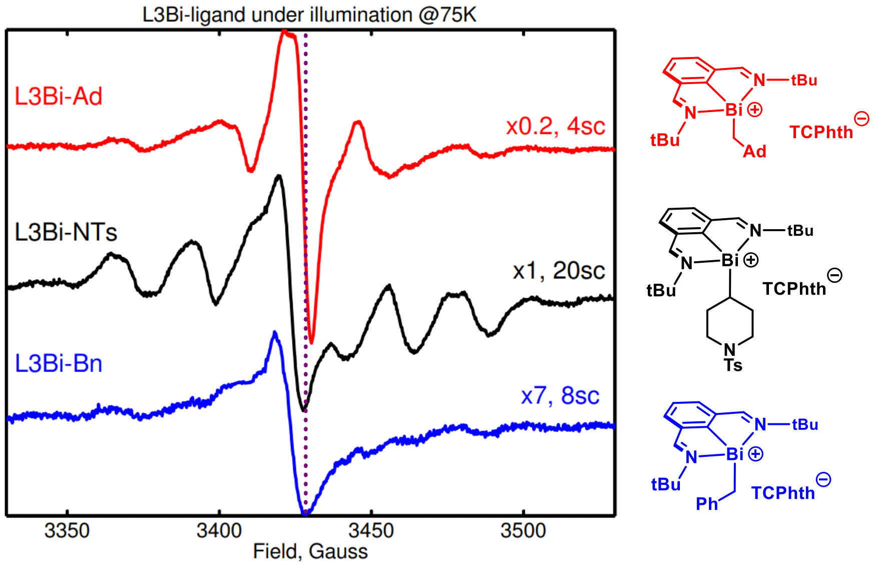

Compounds **12**, **13** and **9** (from top to bottom) in frozen THF under light illumination at 75 K (–198 °C) with 2mW Power and 5 Gauss modulation amplitude, multiple scans as indicated.

Finally, we studied by EPR the intermediate involved in the catalytic C–N coupling reaction (**25**/**25'**). We have only observed this species by NMR using DMF-d_7 as solvent, at temperatures below –40 °C (see Section 7 for details). Thus, we prepared this sample by slow addition of a solution of a solution of bismuth(I) **1** (1 equiv) in DMF to another solution of Boc-proline redox-active ester **22** (1 equiv) under argon, in a Schlenk flask at –50 °C. This mixture was stirred at this temperature for 10 min, before transferring it to a 5.0 mm EPR tube, also under Ar. This was immediately cooled down to –78 °C in a dry ice/acetone bath, and kept there before introducing it into the EPR instrument, were the sample was analyzed at 65 K (–208 °C).

Measurement in the dark already gave a very strong signal (orders of magnitude more intense than for complexes **9**, **12** and **13**), which presumably belongs to a carbon-centered radical. This is consistent with the additional stability provided by the α-N to the radical. Under light, an additional broader signal is formed similar to that observed for complexes unbiased alkyl-bismuth(III) complexes, which, as for those, is only persistent enough to be observed after light irradiation.

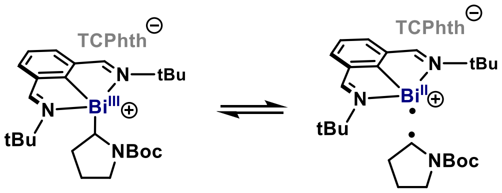

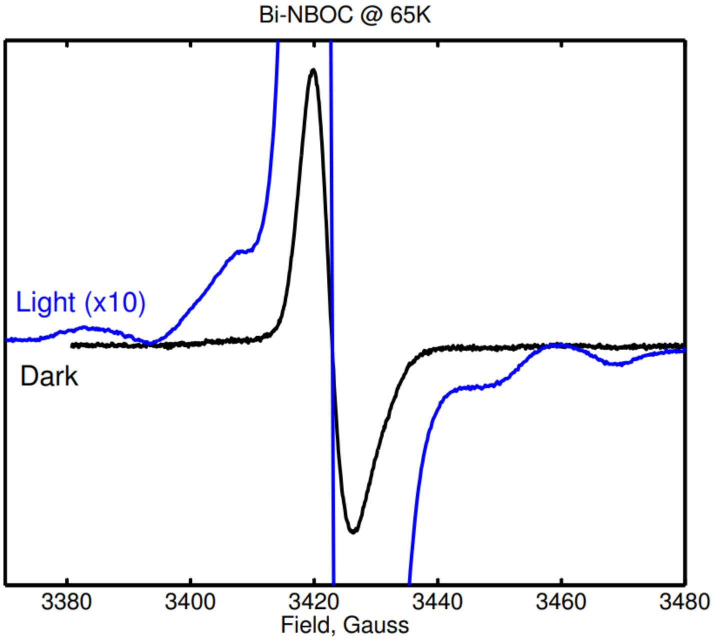

Intermediate **23** in frozen DMF in the dark (black plot, 1 scan) and under light illumination (blue plot, 48 scans; magnified x10 in order to appreciate the multiline signal of the second radical species. The overall intensity of the main signal **does not** increase upon illumination) at 65 K (–208 °C) with 2mW Power and 5 Gauss modulation amplitude.

The EPR spectrum of the α-amino-radical fragment was simulated (red dot line), showing a good fitting with the experimentally observed signal in the dark (blue line).

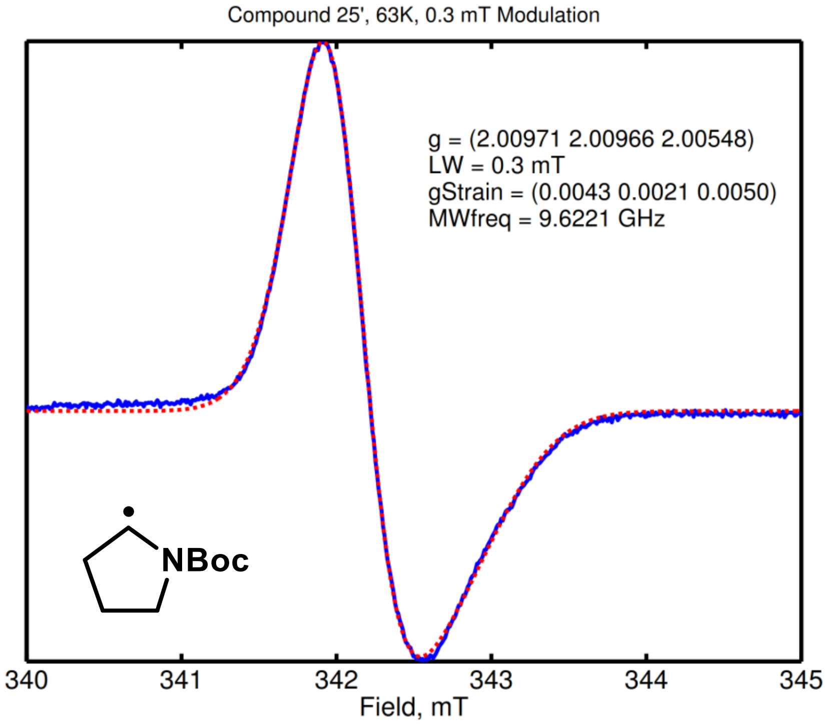

Simulation of spectrum of the α-amino-radical fragment in **23**.

In conclusion, we could observe the α-amino-radical radical intermediate of the radical-catalysis process. This species accumulates very significantly, even in the dark, consistent with α-amino-alkyl redox-active esters undergoing the catalytic reactivity disclosed in this work, leading to a formal CN cross-coupling reaction.

This comes in strong contrast with simple alkyl-bismuth(III) compounds (**9**, **12** and **13**), from which radicals can only be accumulated and detected in significant amount under light irradiation. This key difference in behavior is what led us to the discovery of the bismuth-catalyzed CN coupling reaction.

4.5. Other reaction pathways of alkyl-bismuth(III) adducts

Fate of a benzyl-bismuth(III) complex in solution

We studied the fate of benzyl bismuth(III) complex **9** (which previous EPR/NMR experiments showed to be particularly unstable, especially under light irradiation). We detected as main organic product dimer **S20**, which would arise from a radical-radical coupling of the corresponding alkyl fragment.

Under blue LED, full conversion of the starting adduct was observed already after 2 h. Under ambient light, the conversion of the complex was much slower (80% in 6 days). In both cases, a significant amount of dimer **S20** could be quantified by NMR.

Decomposition of benzyl-bismuth(III) adducts

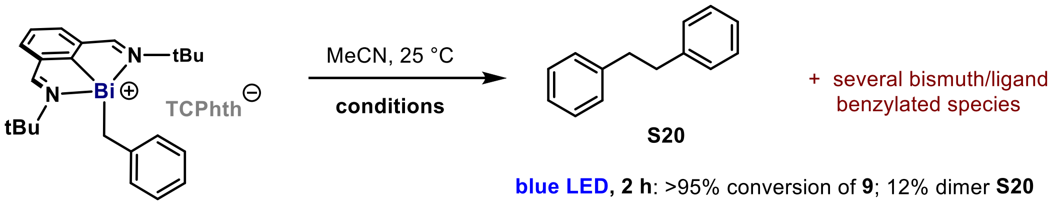

blue LED, 2 h: >95% conversion of **9**; 12% dimer **S20**

ambient light

total time	*conversion of **9***	*dimer **S20***
2 h	*7%*	*5%*
2 days	*45%*	*11%*
3 days	*66%*	*16%*
6 days	*80%*	*21%*

The fate of the rest of the complex was difficult to analyze by NMR, since a very complex mixture of bisimine ligand-containing species were observed. Therefore, when the reaction at room temperature reached full conversion (ca. 10 days), we analyzed it by MS/HRMS.

The results of the analysis are shown on the next page. As summary, several species resulting for the benzylation of the bisimine ligand scaffold were detected. Although an addition to the C atoms of the imines can be speculated, the position of the benzyl substituent in the ligands cannot be established by MS.

Fate of benzyl-bismuth(III) complex 9 in solution (after 12 days under ambient light)

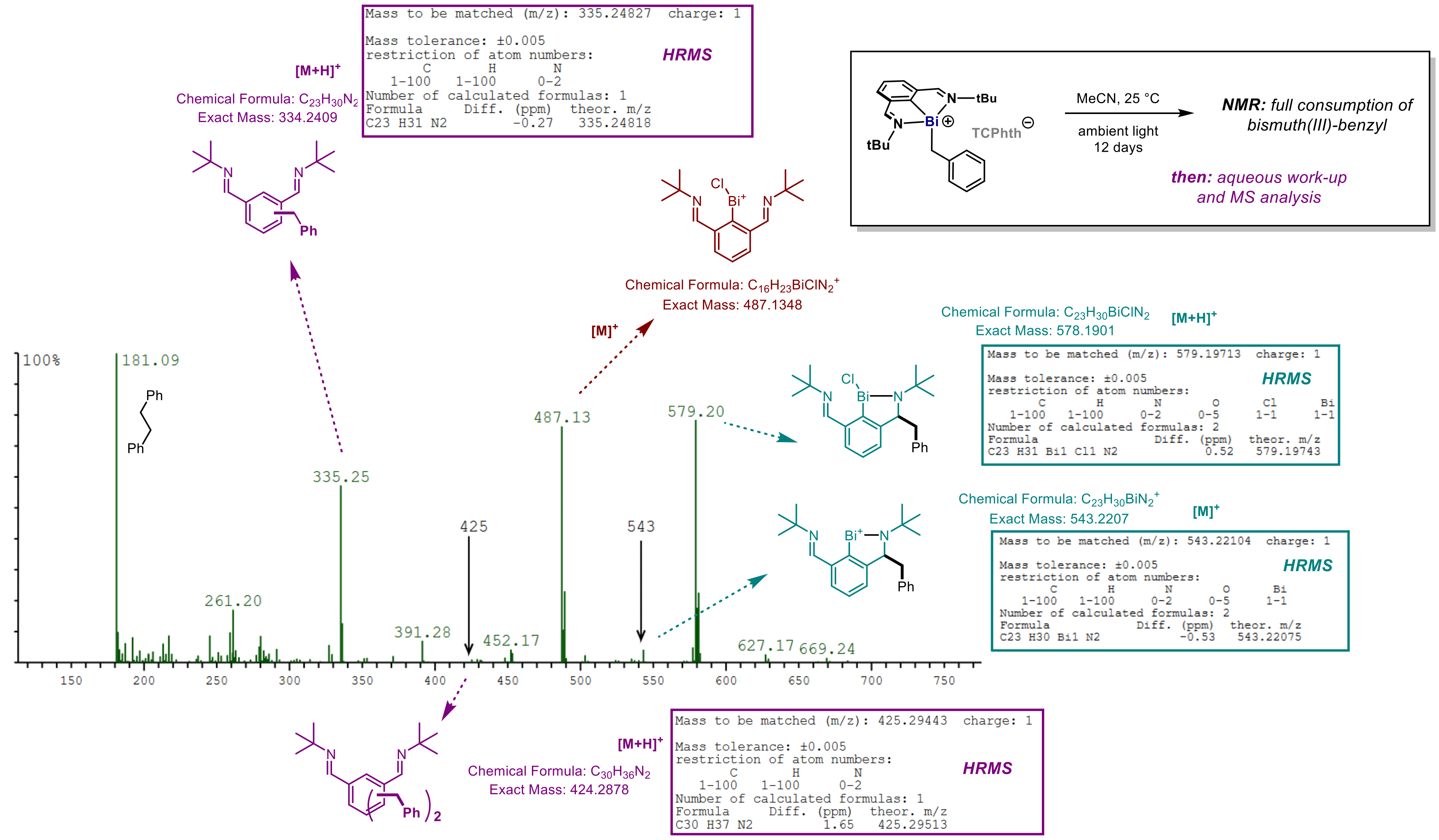

Fate of secondary alkyl bismuth(III) complex 13 in solution (after 7 days under blue LED irradiation)

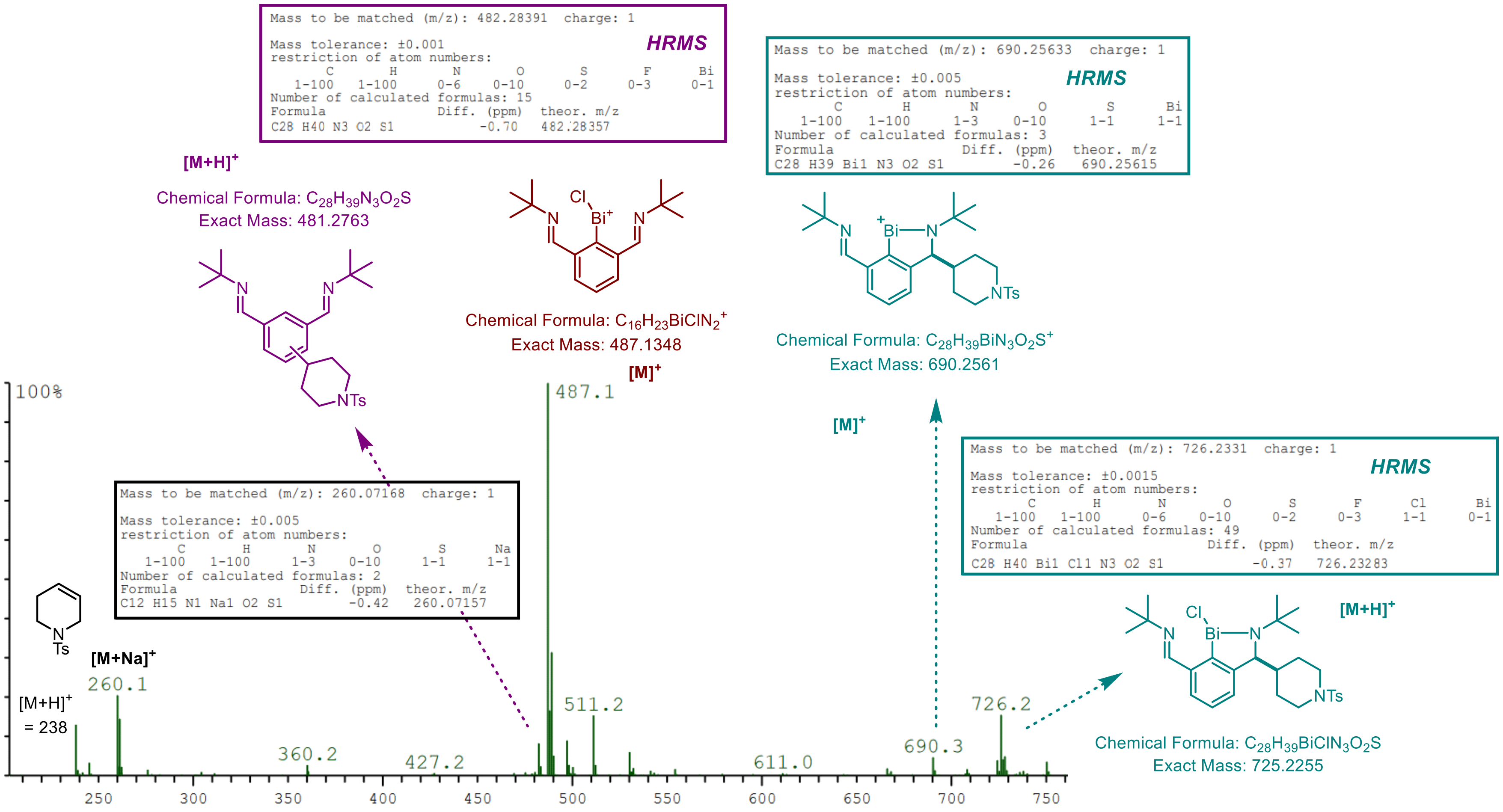

In this case, we also observed some alkylated-ligand products, but only trace amounts as judged by NMR. Conversely, we observed a rather clean formation to the product of beta-H elimination. Thus, we followed this reaction by NMR.

Stability of complex 13

Complex **13** was found to be stable under thermal conditions in the dark. This is in strong contrast with alkyl-transition metal complexes, which readily undergo β-hydride elimination. After stirring for 3 days in the dark, the initial complex was recovered, and neither Bi(I) or alkene **15** were detected (products of formal E1/E2 elimination). There is also no significant dimerization/ligand alkylation detected.

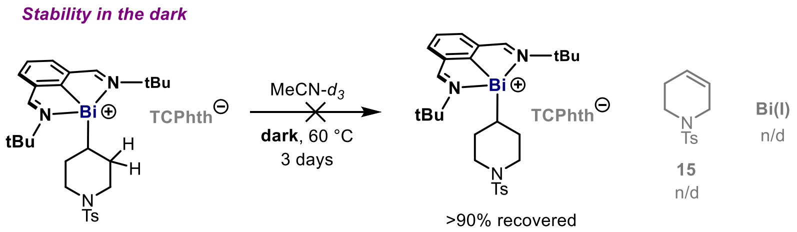

On the other hand, when following the same reaction under blue LED irradiation, a slow, but clean conversion to the corresponding product of elimination **15** was observed, together with the formation of some bismuth(I).

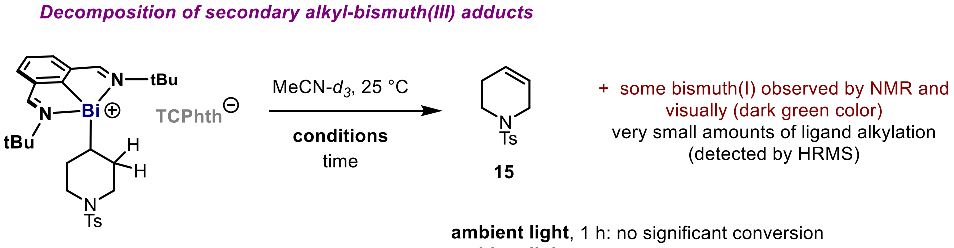

ambient light, 1 h: no significant conversion
ambient light, 12 h: no significant conversion

blue LED

total time	*complex **13***	*elimination product **15***
24 h	*66%*	*34%*
3 days	*35%*	*65%*
5 days	*22%*	*78%*
6 days	*16%*	*84%*
7 days	*12%*	*88%*
8 days	*9%*	*91%*

Thus, whereas benzyl complexes such as **9** as highly unstable under light, others such as **13** are rather robust, and even survive for long periods of time under blue LED irradiation. Further studies on the mechanism of this elimination, and attempts on speeding it up and rendering it catalytic are currently being carried out in our laboratories.

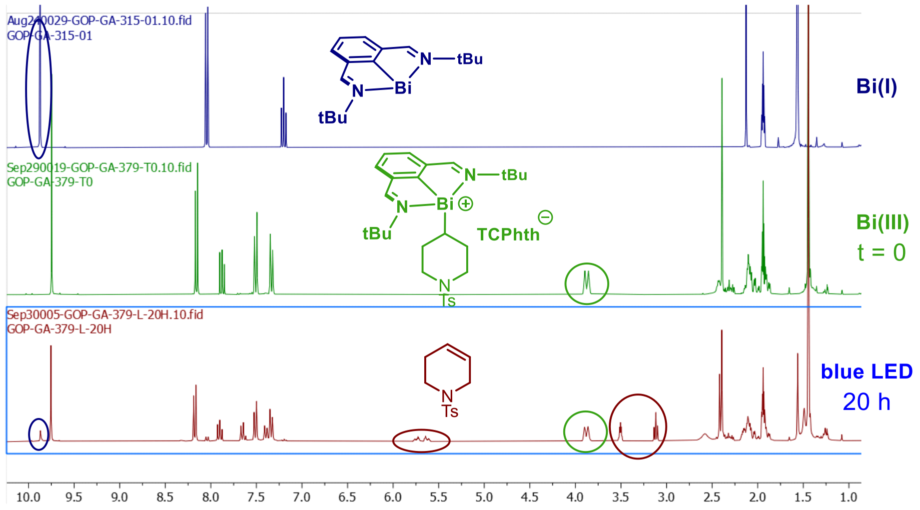

Kinetic profile of the reaction:

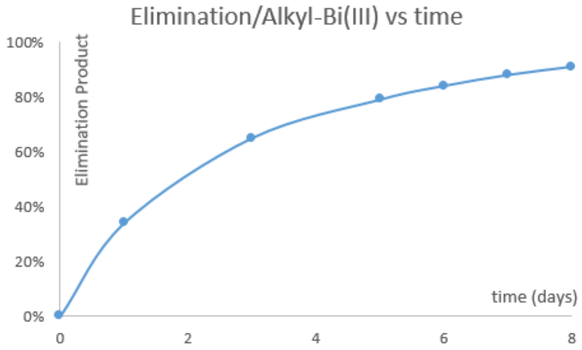

Elimination product **15** spectroscopic data fully matched the reported ones.[8]

^{1}H NMR (300 MHz, $CDCl_3$) δ 7.70 – 7.64 (m, 2H), 7.32 (d, *J* = 7.9 Hz, 2H), 5.75 (dtt, *J* = 9.9, 3.9, 2.3 Hz, 1H), 5.61 (dtt, *J* = 10.2, 3.3, 1.9 Hz, 1H), 3.58 (p, *J* = 2.8 Hz, 2H), 3.18 (t, *J* = 5.7 Hz, 2H), 2.43 (s, 3H), 2.22 (td, *J* = 5.9, 3.0 Hz, 2H).

HRMS (ESI Positive): calculated for $C_{12}H_{15}NNaO_2S$ $[M+Na]^+$: 260.07157; found: 260.07168.

Crude ^{1}H NMR analysis showing clean conversion to the elimination product **15**.

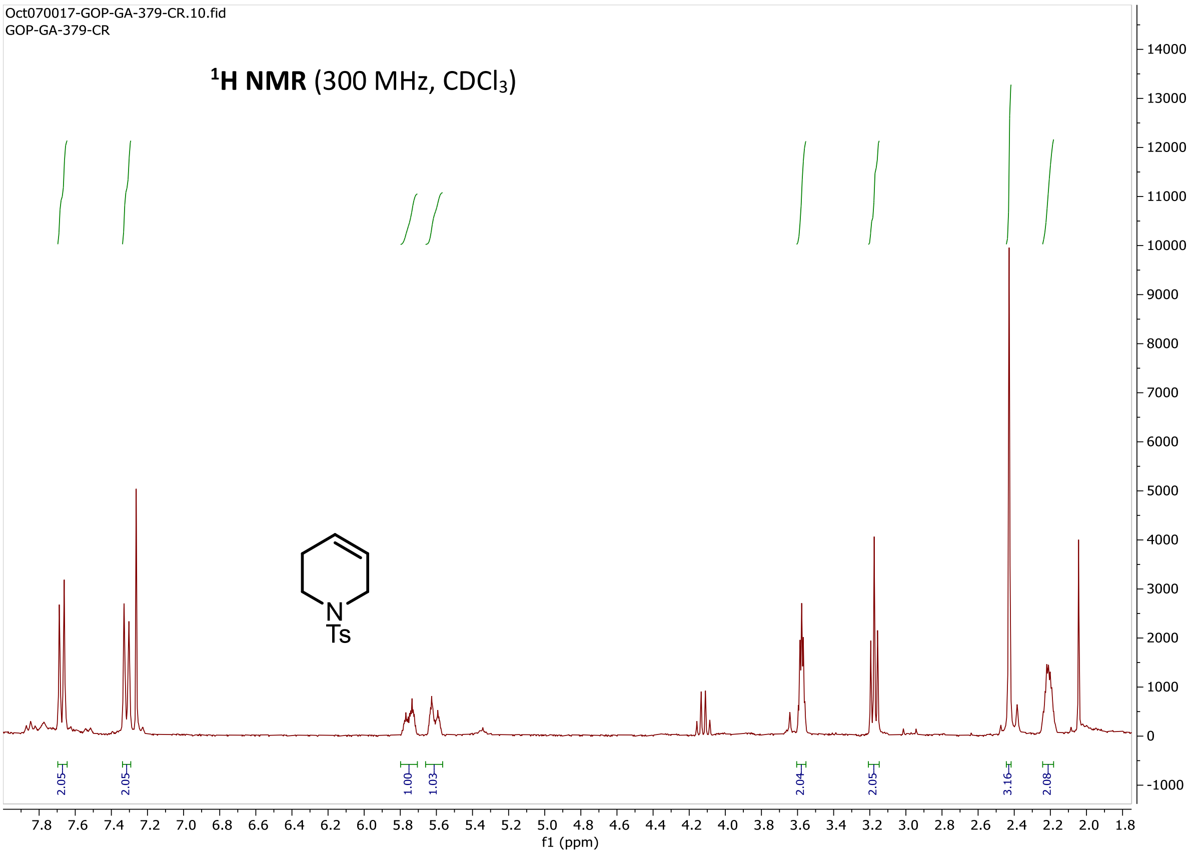

Alkyl scrambling experiments

Alkyl-bismuth(III) complexes A-D were prepared in-situ according to the general procedure (page SI-14), by mixing under argon the corresponding bismuth(I) complex (**1** or **S5**) and redox-active ester (**2** or **22**), and stirring in degassed acetonitrile for 15 min. They were obtained as solutions in MeCN-d_3 and analyzed by ^{1}H NMR, in order to identify all possible alkyl-bismuth(III) combinations.

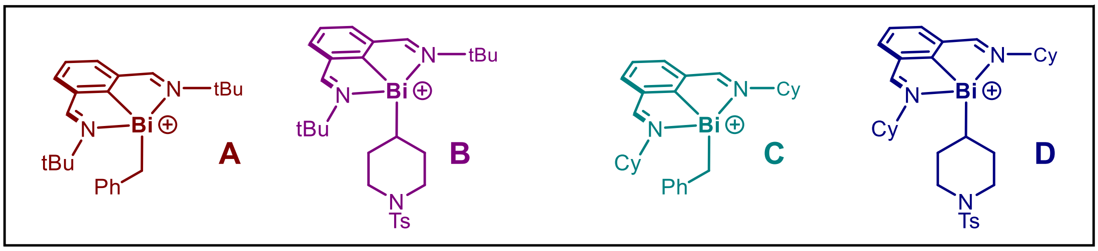

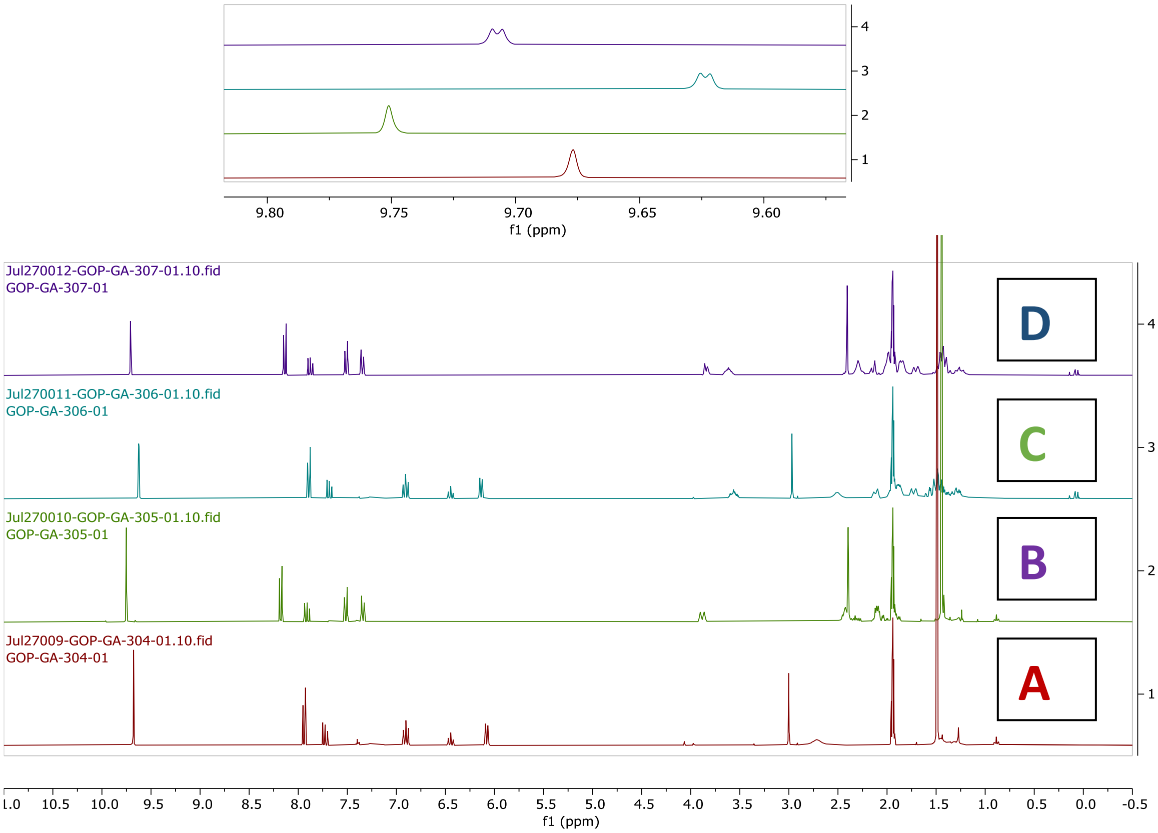

Then, they were mixed accordingly and put in another NMR tube under argon in order to follow the scrambling reactions (see next page). The experiment was carried out twice: one under blue LED irradiation, and the other under ambient light.

Equimolar solutions in MeCN-d_3 of complex A and D were mixed together under argon. The solution was split into two NMR tubes, one of each left under ambient light, and the other one under blue LED irradiation. ^{1}H NMR were recorded at different times to study the distribution of bismuth(III)-alkyl species.

Scrambling of the alkyl groups was detected, both under ambient (slower exchange) and under blue LED (faster exchange) light. A rather clear transfer of the secondary piperidyl group was observed, forming complex B until a ca. 3:1 D/B ratio is observed. Formation of complex C was also detected but, as shown in the previous section, benzyl-bismuth(III) complexes are not stable, and they undergo side reactivity such as dimerization or benzylation of the ligands. Thus, as expected, after enough time, both A and C are decomposed, but complex A survives enough time for some exchange of secondary alkyl to take place successfully.

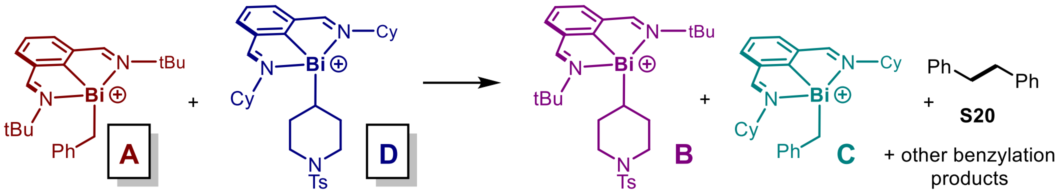

%composition of each species relative to all 4 together in the mixture at any given time/conditions

under ambient light, room temperature

time	amount of A	amount of D	amount of B	amount of C	S20	B/D ratio
t = 0	50%	50%	0%	0%	0%	**0**
30 min	38%	53%	5%	3%	2%	**0.09**
5 h	16%	60%	17%	8%	4%	**0.28**
18 h	n/d	71%	29%	0%	7%	**0.41**

under blue LED irradiation

time	amount of A	amount of D	amount of B	amount of C	S20	B/D ratio
t = 0	50%	50%	0%	0%	0%	**0**
5 min	21%	67%	8%	5%	3%	**0.12**
3 h	1%	71%	28%	0%	6%	**0.39**

This suggests a higher stability of complex B over D, which keeps forming for as long as some amount of complex A is available (which eventually decomposes via dimerization/benzylation pathways, see previous section). Indeed, when mixing B and C together as starting materials, no formation of D is observed: only the decomposition of C via benzylation/dimerization happens.

4.6. Stoichiometric reactivity of the alkyl-bismuth(III) complexes

Giese addition

4-(2-(Phenylsulfonyl)ethyl)-1-tosylpiperidine (21)

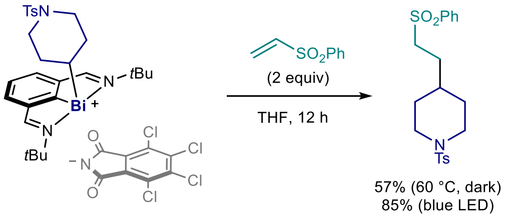

In an argon-filled glovebox, vinylphenyl sulfone (0.08 mmol, 2 equiv) was added to a solution of bismuth(III)-alkyl complex **13** (0.04 mmol, 1 equiv) in 1 mL of THF, in a previously oven-dried culture tube. The tube was closed, and taken out of the glovebox, before stirring it either in the dark at 60 °C (57% yield of product) or under blue LED irradiation (85% yield of product) for 12 h, and after work-up, the mixtures were analyzed by NMR spectroscopy. The product can be isolated by flash column chromatography in silica gel using a gradient from 9:1 to 6:4 hexane/EtOAc as eluent.

We found that, in contrast to the catalytic examples in Section 4.7, this stoichiometric reaction went to completion even in the absence of external H-donor sources. The source of H in the absence of external H-donor is unclear; however, we speculate that the solvent might be the origin of the H atom. In the presence of 2 equiv of γ-terpinene, the corresponding product was also obtained in 50% or 65% yield, in the dark at 60 °C or under blue LED irradiation, respectively.

^{1}H NMR (600 MHz, $CDCl_3$) δ 7.87 (d, 2H), 7.69 – 7.64 (m, 1H), 7.64 – 7.60 (m, 2H), 7.59 – 7.55 (m, 2H), 7.31 (d, 2H), 3.86 – 3.68 (m, 2H), 3.09 – 2.98 (m, 2H), 2.43 (s, 3H), 2.20 (td, *J* = 11.9, 2.6 Hz, 2H), 1.74 – 1.60 (m, 4H), 1.36 – 1.30 (m, 1H), 1.29 – 1.27 (m, 1H), 1.25 – 1.22 (m, 1H).

^{13}C NMR (151 MHz, $CDCl_3$) δ 143.7, 139.2, 133.9, 133.2, 129.8, 129.5, 128.1, 127.8, 53.8, 46.2, 34.1, 31.1, 28.7, 21.7.

HRMS (ESI Positive): calculated for $C_{20}H_{25}NO_4S_2Na$ $[M+Na]^+$: 430.11172; found: 430.11204.

TEMPO trapping

1-(Benzyloxy)-2,2,6,6-tetramethylpiperidine (S21)

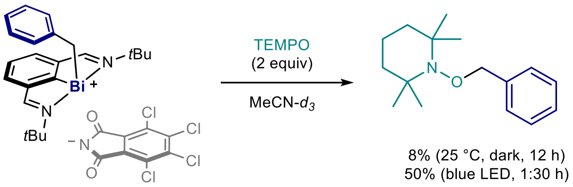

In an argon-filled glovebox, TEMPO (0.044 mmol, 2 equiv) was added to a solution of bismuth(III)-alkyl complex **9** (0.022 mmol, 1 equiv) in 0.6 mL of MeCN-d_3, in a previously oven-dried culture tube. The tube was closed, and taken out of the glovebox, before stirring it either in the dark at 25 °C for 12 h (8% NMR yield of product) or under blue LED irradiation for 1:30 h (50% yield of product). After crude-NMR analysis, the product was isolated by flash column chromatography in silica gel.

Characterization data matched previously reported ones.[9]

^{1}**H NMR** (600 MHz, $CDCl_3$) δ 7.40 – 7.32 (m, 4H), 7.31 – 7.26 (m, 1H), 4.83 (s, 2H), 1.68 – 1.55 (m, 2H), 1.55 – 1.46 (m, 4H), 1.26 (s, 6H), 1.16 (s, 6H).

1-((4-Bromobenzyl)oxy)-2,2,6,6-tetramethylpiperidine (S22)

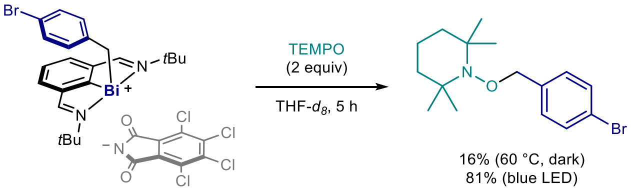

In an argon-filled glovebox, TEMPO (0.08 mmol, 2 equiv) was added to a solution of bismuth(III)-alkyl complex **11** (0.04 mmol, 1 equiv) in 1 mL of THF-d_8, in a previously oven-dried culture tube. The tube was closed, and taken out of the glovebox, before stirring it either in the dark at 60 °C (16% NMR yield of product) or under blue LED irradiation (81% NMR yield of product) for 5 h, and the mixtures were analyzed by NMR.

Characterization data matched previously reported ones for the TEMPO-adduct product.[10]

^{1}**H NMR** (300 MHz, $CDCl_3$) δ 7.47 – 7.44 (m, 2H), 7.25 – 7.20 (m, 2H), 4.76 (s, 2H), 1.56 – 1.48 (m, 6H), 1.22 (s, 6H), 1.14 (s, 6H).

HRMS (ESI Positive): calculated for $C_{16}H_{25}NOBr$ $[M+Na]^{+}$: 326.11141; found: 326.11172.

2,2,6,6-Tetramethyl-1-((1-tosylpiperidin-4-yl)oxy)piperidine (S23)

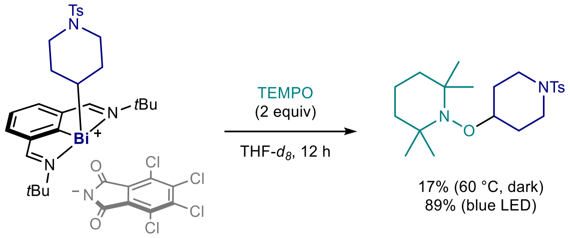

17% (60 °C, dark)
89% (blue LED)

In an argon-filled glovebox, TEMPO (0.08 mmol, 2 equiv) was added to a solution of bismuth(III)-alkyl complex **13** (0.04 mmol, 1 equiv) in 1 mL of THF-d_8, in a previously oven-dried culture tube. The tube was closed, and taken out of the glovebox, before stirring it either in the dark at 60 °C (17% NMR yield of product) or under blue LED irradiation (89% NMR yield of product) for 12 h, and the mixtures were analyzed by NMR.

Characterization data matched previously reported ones for the TEMPO-adduct product.[11]

^{1}**H NMR** (300 MHz, $CDCl_3$) δ 7.65 (d, *J* = 2.0 Hz, 2H), 7.32 (d, *J* = 8.0 Hz, 2H), 3.66 – 3.56 (m, 3H), 2.49 – 2.40 (m, 5H), 2.08 – 2.00 (m, 2H), 1.72 – 1.62 (m, 2H), 1.42 (s, 6H), 1.06 (s, 12H).

Radical-clock experiments: cyclopropane ring opening

Reaction of cylclopropylmetyhl redox-active ester with the bismuth(I) complex leads directly to the formation of the open product, due to an in-cage radical ring opening which takes place faster than the radical recombination, not allowing the detection of the unopened bismuth(III)-alkyl adduct.

[(2,6-(*t*BuNCH)$_2$C$_6$H$_3$)Bi(but-3-en-1-yl)(tetrachlorophthalimide)] (**19**)

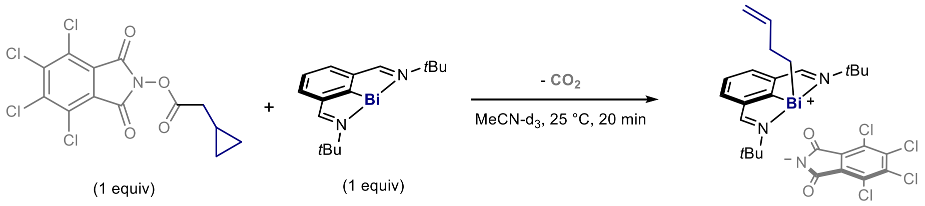

The title compound was obtained from bismuthinidene **1** (0.022 mmol, 1 equiv) and the corresponding redox-active ester **17** (0.022 mmol, 1 equiv) in 0.6 mL of MeCN-*d$_3$* (0.04 M) after stirring for 20 min at room temperature (>95% conversion by NMR).

^{1}H NMR (600 MHz, CD$_3$CN) δ 9.69 (s, 2H), 8.17 (d, *J* = 7.6 Hz, 2H), 7.91 (dd, *J* = 7.7, 7.4 Hz, 1H), 5.56 (ddt, *J* = 16.8, 10.1, 6.6 Hz, 1H), 4.90 – 4.80 (m, 1H), 4.79 – 4.67 (m, 1H), 2.71 (dtt, *J* = 9.2, 6.5, 1.3 Hz, 2H), 2.02 – 1.98 (m, 2H), 1.48 (s, 18H).

^{13}C NMR (151 MHz, CD$_3$CN) δ 185.7, 180.1, 169.2, 149.2, 146.1, 137.0, 136.0, 135.6, 130.8, 126.2, 115.4, 62.1, 51.6, 32.2, 31.1.

HRMS (ESI Positive): calculated for $C_{20}H_{30}BiN_2$ [M-TCPhth]$^+$: 507.22075; found: 507.22112.

On the other hand, the reaction of bismuthinidene **1** with the corresponding alkyl iodide, takes place through a polar S$_N$2 mechanism, allowing the observation and characterization of the corresponding unopened bismuth(III)-alkyl adduct at low temperature.

[(2,6-(*t*BuNCH)$_2$C$_6$H$_3$)Bi(1-cyclopropylmethyl)(iodide)] (**16**)

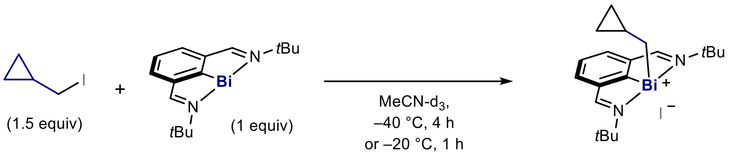

The title compound was obtained from bismuthinidene **1** (0.016 mmol, 1 equiv) and cyclopropylmethyl iodide (0.029 mmol, 1.8 equiv at –40 °C or 1.5 equiv at –20 °C) in 0.7 mL of MeCN-*d$_3$* , monitored in-situ by NMR, after 4 h at –40 °C or 1 h at -20 °C (>95% conversion by NMR).

^{1}H NMR (500 MHz, CD$_3$CN) δ 9.71 (s, 2H), 8.18 (d, *J* = 7.6 Hz, 2H), 7.92 (t, *J* = 7.5 Hz, 1H), 2.03 (d, *J* = 7.4 Hz, 2H), 1.44 (s, 18H), 0.79 – 0.85 (m, 1H), -0.04 0.05 – -0.04 (m, 2H), -0.42 – -0.52 (m, 2H).

^{13}C NMR (126 MHz, CD$_3$CN) δ 187.3, 167.0, 149.0, 136.3, 130.2, 61.7, 58.2, 30.6, 11.7, 11.4.

Kinetic profile of the S_N2 reaction to give unopened bismuth(III) adduct 16 at –40 °C by ^1H NMR

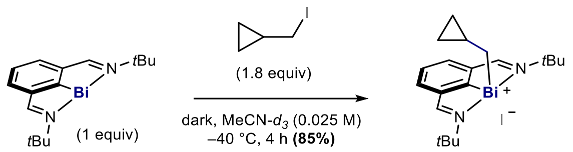

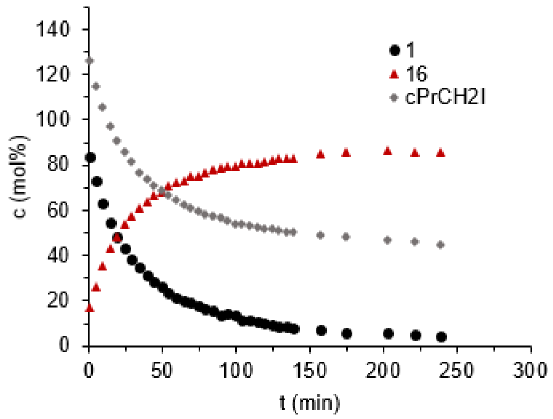

1= starting Bi(I); **16** = unopened bismuth(III) alkyl; **cPrCH2I** = cyclopropylmethyl iodide (1.8 equiv). The first spectrum was acquired 10 min after warming up the mixture to –40 °C

Kinetic profile of the S_N2 reaction to give unopened bismuth(III) adduct 16 at -20 °C by ^1H NMR

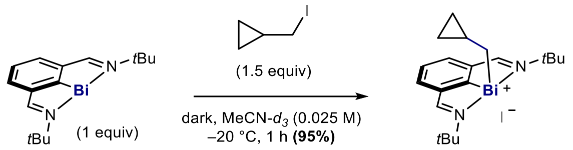

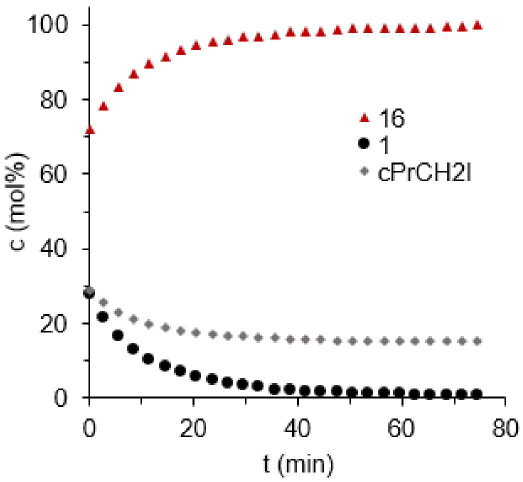

1= starting Bi(I); **16** = unopened bismuth(III) alkyl; **cPrCH2I** = cyclopropylmethyl iodide (1.5 equiv). The first spectrum was acquired 10 min after warming up the mixture to –20 °C (significant conversion already observed).

To further prove that alkyl-bismuth(III) compounds such as **16** are radical-equilibrium complexes, we demonstrated that unopened adduct **16** can open up to give the product of radical opening **18** both under photochemical and thermal conditions.

[(2,6-(*t*BuNCH)$_2$C$_6$H$_3$)Bi(but-3-en-1-yl)(iodide)] (**18**)

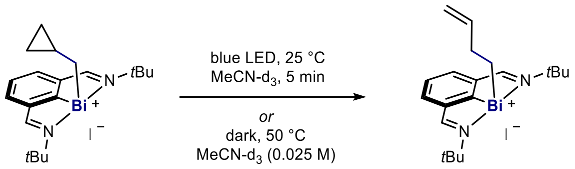

Taking the solution of the cylclopropylmethyl bismuth(III) out of the NMR instrument and exposing it to blue LED irradiation for 5 min led to quantitative opening of the cyclopropane ring.

Alternatively, without removing the tube from the NMR instrument, the open product was cleanly formed at a slower rate upon heating to 50 °C.

^{1}H NMR (300 MHz, CD$_3$CN) δ 9.58 (s, 2H), 8.07 (d, *J* = 7.5 Hz, 2H), 7.79 (dd, *J* = 7.9, 7.2 Hz, 1H), 5.40 (ddt, *J* = 16.8, 10.2, 6.6 Hz, 1H), 4.68 (ddt, *J* = 10.2, 2.2, 1.2 Hz, 1H), 4.57 (dq, *J* = 17.1, 1.6 Hz, 1H), 2.66 – 2.46 (m, 2H), 1.91 – 1.83 (m, 2H), 1.32 (s, 18H).

^{13}C NMR (151 MHz, CD$_3$CN) δ 186.2, 169.3, 149.2, 146.2, 137.1, 130.9, 115.4, 62.1, 51.9, 32.2, 31.1.

HRMS (ESI Positive): calculated for $C_{20}H_{30}BiN_2$ $[M-I]^+$: 507.22075; found: 507.22108.

Kinetic profile of the thermal cyclopropane opening to give bismuth(III) adduct 18 at 50 °C by ^{1}H NMR

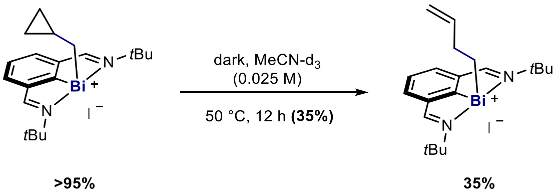

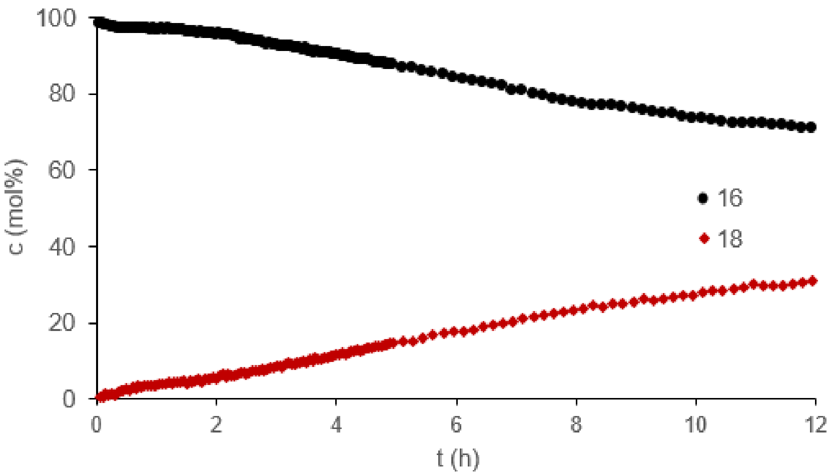

16 = unopened bismuth(III) alkyl; **18** = open bismuth(III) alkyl.
t = 0 is for the first recorded spectrum after warming up the solution of **16** to 50 °C.

The obtained mixture of opened and unopened bismuth(III) adducts can also be taken out of the NMR instrument and irradiated with blue LED to get full conversion to the corresponding open product.

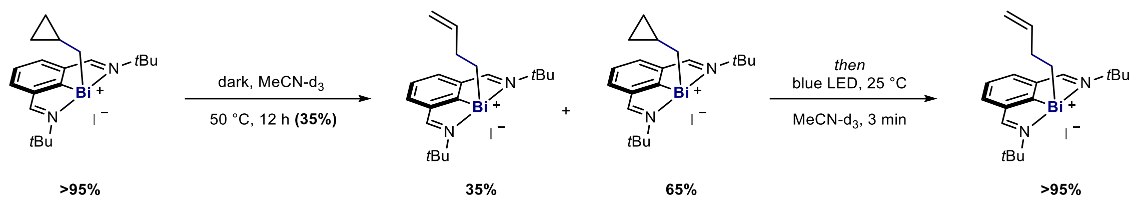

4.7. Radical reactions promoted by catalytic bismuth and light

A culture tube with a magnetic stirring bar was charged, in the glovebox, with a redox-active ester (0.1 mmol, 1 equiv), bismuthinidene **1** (0.01 mmol, 10 mol %) and the corresponding reactants for each case (i.e., radical acceptor, or hydrogen donor, when appropriate). All solids were dissolved in a 1:1 mixture of DMA and MeCN. The tube was closed, taken outside of the glovebox, and stirred under the corresponding set up (either in the dark or under blue LED irradiation) for 1-2 h. When appropriate, the reaction products were isolated by flash column chromatography.

4-(2-(Phenylsulfonyl)ethyl)-1-tosylpiperidine (21)

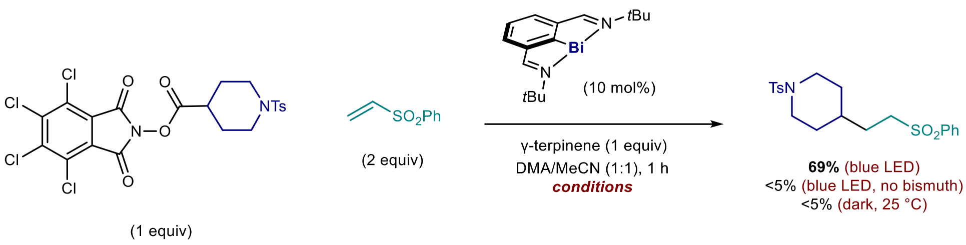

The title product was isolated in 69% yield as a white solid after flash column chromatography in silica gel, using hexane/EtOAc/dichloromethane 7:2:1 as eluent.

^{1}H NMR (600 MHz, $CDCl_3$) δ 7.87 (d, 2H), 7.69 – 7.64 (m, 1H), 7.64 – 7.60 (m, 2H), 7.59 – 7.55 (m, 2H), 7.31 (d, 2H), 3.86 – 3.68 (m, 2H), 3.09 – 2.98 (m, 2H), 2.43 (s, 3H), 2.20 (td, J = 11.9, 2.6 Hz, 2H), 1.74 – 1.60 (m, 4H), 1.36 – 1.30 (m, 1H), 1.29 – 1.27 (m, 1H), 1.25 – 1.22 (m, 1H).

^{13}C NMR (151 MHz, $CDCl_3$) δ 143.7, 139.2, 133.9, 133.2, 129.8, 129.5, 128.1, 127.8, 53.8, 46.2, 34.1, 31.1, 28.7, 21.7.

HRMS (ESI Positive): calculated for $C_{20}H_{25}NO_4S_2Na$ $[M+Na]^+$: 430.11172; found: 430.11204.

1-Tosylpiperidine (S24)

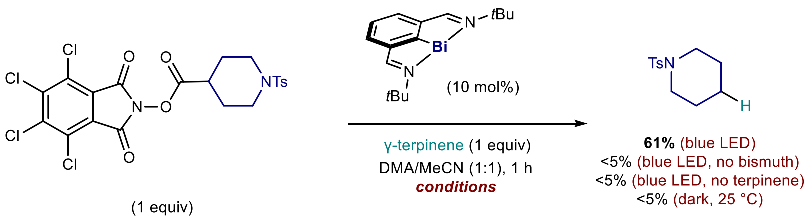

The title product was isolated in 61% yield as a white solid after flash column chromatography in silica gel, using hexane/EtOAc/dichloromethane 7:2:1 as eluent.

^{1}H NMR (600 MHz, $CDCl_3$): δ 7.64 (d, J = 8.2 Hz, 2H), 7.31 (d, J = 0.7 Hz, 2H), 3.01 – 2.92 (m, 4H), 2.43 (s, 3H), 1.66 – 1.61 (m, 4H), 1.47 – 1.38 (m, 2H).

^{13}C NMR (151 MHz, $CDCl_3$): δ 143.4, 133.5, 129.7, 127.9, 47.1, 25.3, 23.7, 21.7.

HRMS (ESI Positive): calculated for $C_{12}H_{17}NO_2S$ $[M]^+$: 239.09745; found: 239.09770.

4-(2,2-Diphenylvinyl)-1-tosylpiperidine (S25)

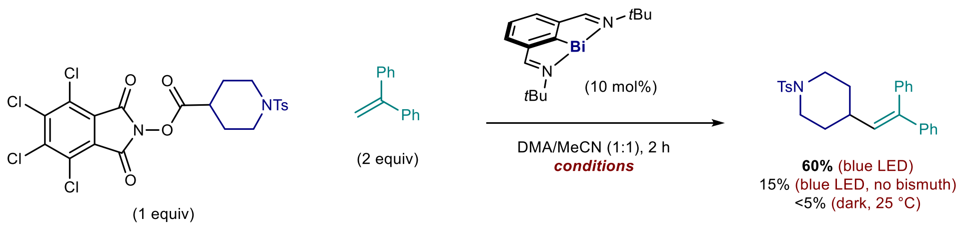

The title product was isolated in 60% yield as a white solid after flash column chromatography in silica gel, using hexane/EtOAc/dichloromethane 7:2:1 as eluent.

^{1}H NMR (600 MHz, $CDCl_3$): δ 7.61 – 7.58 (m, 2H), 7.34 – 7.30 (m, 2H), 7.30 – 7.26 (m, 3H), 7.26 – 7.18 (m, 3H), 7.19 – 7.14 (m, 2H), 7.11 – 7.07 (m, 2H), 5.83 (d, *J* = 9.8 Hz, 1H), 3.74 (d, *J* = 11.5 Hz, 2H), 2.41 (s, 3H), 2.11 (td, *J* = 11.9, 2.7 Hz, 2H), 2.06 – 1.99 (m, 1H), 1.73 – 1.66 (m, 2H), 1.65 – 1.57 (m, 2H).

^{13}C NMR (151 MHz, $CDCl_3$): δ 142.9, 142.7, 141.8, 140.4, 130.1, 128.5, 128.4, 128.3, 128.2, 127.3, 126.9, 126.9, 125.8, 35.8, 31.1, 29.7, 29.6.

HRMS (ESI Positive): calculated for $C_{26}H_{27}NO_2SNa$ $[M+Na]^+$: 440.16541; found: 440.16547.

Note: Even though the photocatalytic examples above only give significant yields in the presence of bismuth(I) complex, we found that for other substrates, and other combinations of solvent/reductant there was a very significant background reactivity that does not require catalyst.

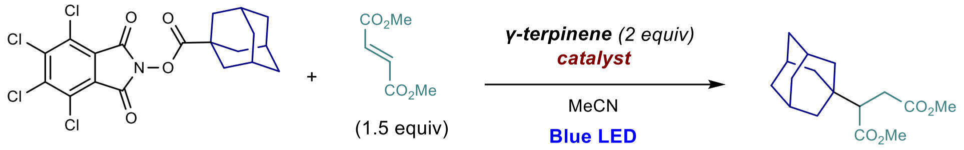

catalyst (time)	***Yield***
(*t*BuBisimine)Bi (10 mol %) *(36 h)*	34% with bismuth
no catalyst (20 h)	7% (no bismuth)

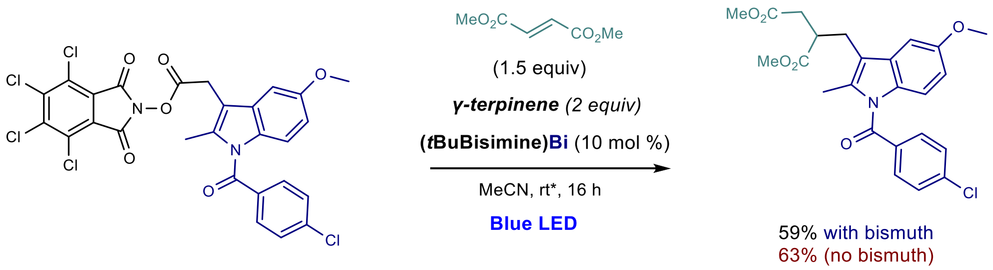

Due to these observations, this reactivity was not investigated further. The role and photochemical behavior of bismuth complexes is unclear and it is currently under investigation.

Thus, the mechanistic details behind these transformations promoted by substoichiometric bismuth and light are beyond the aim of this study.

5. Development and optimization of the C–N coupling reaction

Discovery and initial hit

While attempting the characterization of an α-amino alkyl bismuth(III) complex formed through oxidative addition we only observed a clean conversion of the starting redox-active ester into the product of formal decarboxylation. We hypothesized that it would be formed via further one-electron oxidation of the corresponding α-amino alkyl radical by a highly reactive Bi(II) species, which would regenerate a Bi(I) complex, upon release of a Shono-type iminium carbocation. This highly electrophilic species can react with the in-situ released tetrachlorophthalimide, giving the observed reaction product. Continuous observation of the Bi(I) complex suggests the oxidative addition as rate-limiting step.

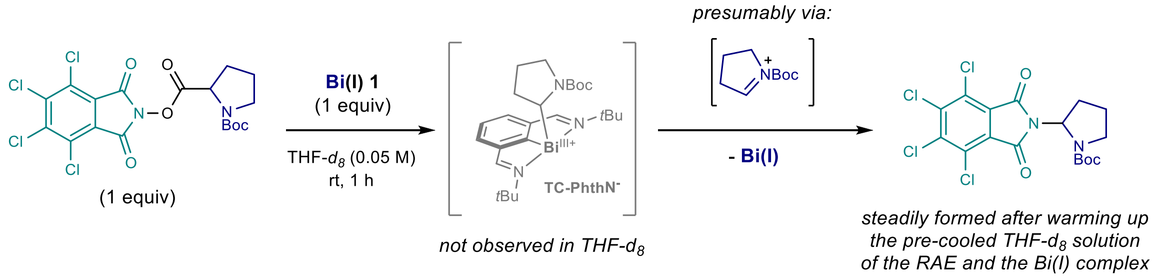

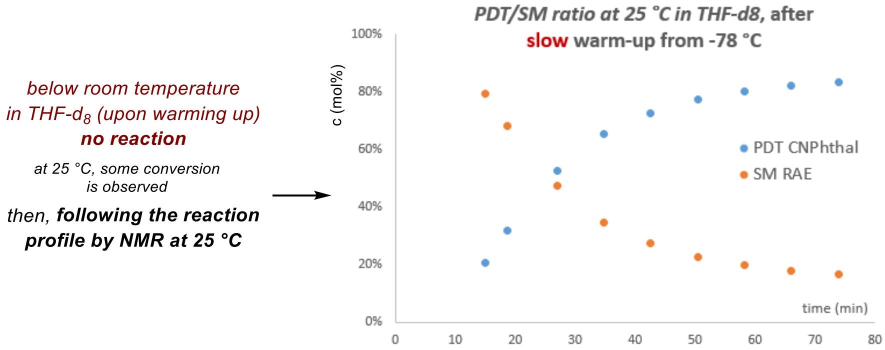

Following this finding, we extended this idea for the development of a C–N cross-coupling reaction, by addition of an external N-nucleophile.

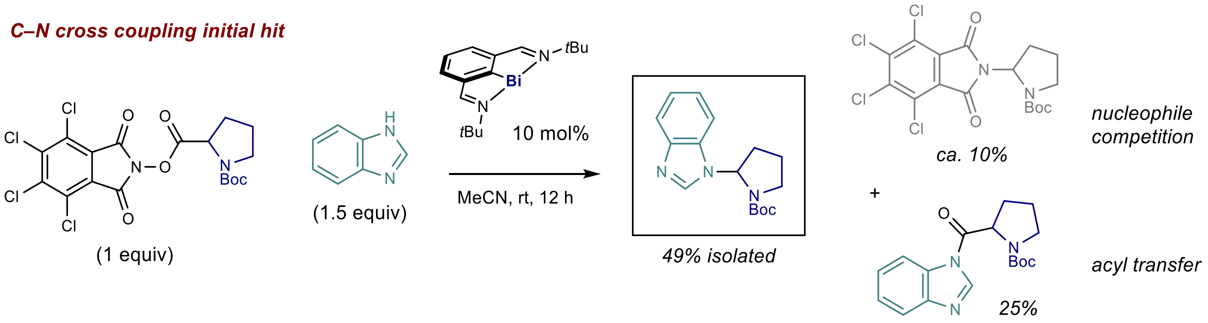

The main side-pathways were found to be, as expected, the competition of phthalimide as nucleophile, and the acyl-transfer reaction to the external N-nucleophile. Both could be minimized during the reaction optimization.

For all optimization and control experiments shown here, yields and conversions were determined by ^{1}H NMR using diphenylmethane as internal standard, in reactions carried out in a 0.05 mmol scale.

Solvent screening

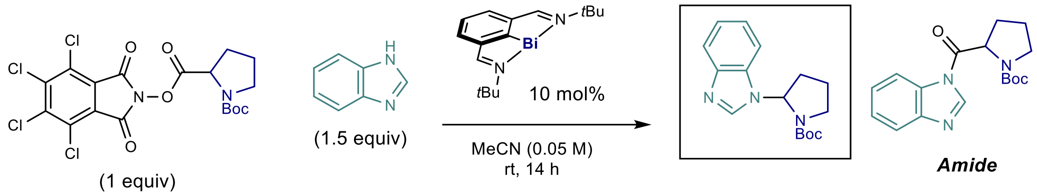

Deviations from Initial Conditions	*Yield C–N*	*Conv. RAE*	*Conv. Nuc (1.5 equiv)*	*Yield Amide*
none	**51%**	**70%**	**90%**	**15%**
MeCN as solvent (dry, not degassed)	28%	64%	73%	28%
PhMe as solvent	3%	60%	82%	41%
Pentane as solvent	11%	61%	64%	30%
Et_2O as solvent	43%	72%	55%	35%
**Dioxane as solvent**	**63%**	**100%**	**60%**	**14%**
THF as solvent	22%	65%	61%	40%
**DMA as solvent**	**83%**	**100%**	**80%**	**6%**
DCM as solvent (dry, not degassed)	8%	74%	n/d	31%

Optimization in DMA

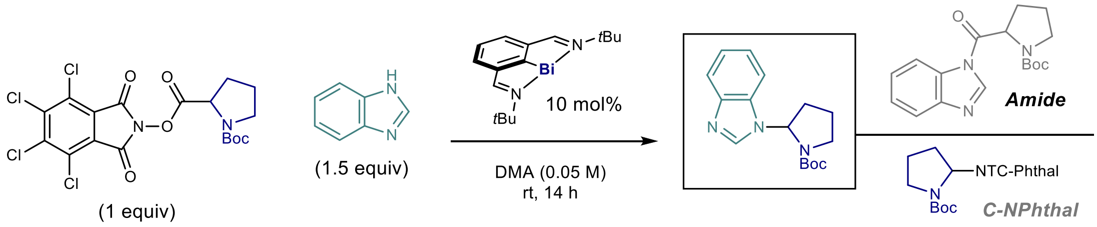

Switching to DMA (0.05 M) as solvent ** the rest of the conversion is some nuc. add of the NPhthal*

Deviations from Initial Conditions	*Yield C–N (C-NPhthal)*	*Conv. RAE*	*Yield Amide*
**initial conditions**	**83%**	**100%***	**6%**
without manually degassing DMA	**84%** (15%)	**100%***	**3%**
* Switch to using directly anhydrous solvent purchased from Sigma-Aldrich, opened and stored in the glovebox			
no catalyst (control)	0% (0%)	28%	24%
**1 equiv RAE + 2.5 equiv Nuc**	**86%** (10%)	**100%**	**6%**
1 equiv RAE + 1 equiv Nuc	58% (40%)	100%	7%
1.5 equiv RAE + 1 equiv Nuc	84% (50%)	85%	12%
0 °C instead of rt	41% (5%)	66%	20%
40 °C instead of rt	83% (16%)	100%	6%
reaction time = 1 h	83% (10%)	>95%	7%
**reaction time = 3 h**	**85%** (15%)	**100%**	**6%**

*Between parenthesis C-NPhthal product

Yields based on the limting reagents and conversions based on the total initial amount of each reagent

Further tuning of the conditions

Switching to 3 equiv Nuc and 2 h reaction time

Deviations from Initial Conditions	*Yield C–N (C-NPhthal)*	*Conv. RAE*	*Yield Amide*
initial conditions	86% (10%)	100%	7%
5 mol % of **Bi(I) 1**	56% (7%)	92%	24%
20 mol % of **Bi(I) 1**	75% (12%)	100%	9%
0.1 M in DMA	86% (10%)	100%	12%
0.025 M in DMA	88% (10%)	100%	3%

final std. conditions: 1 equiv RAE, 3 equiv Nuc, 10 mol % of **Bi(I) 1**, 0.033 M in DMA, 25 °C, 2 h

Final Standard Conditions for the C–N Cross Coupling

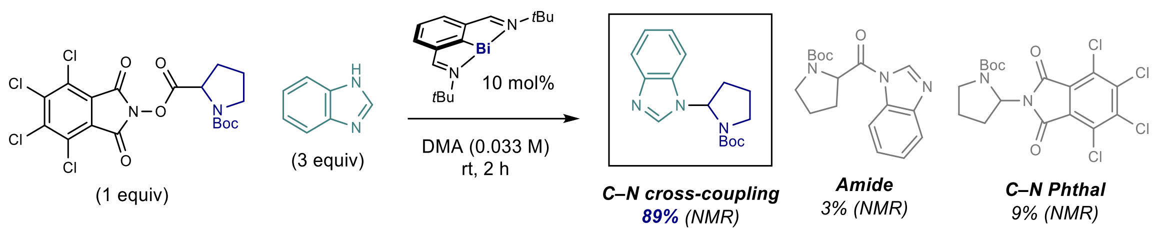

Additional control and optimization experiments

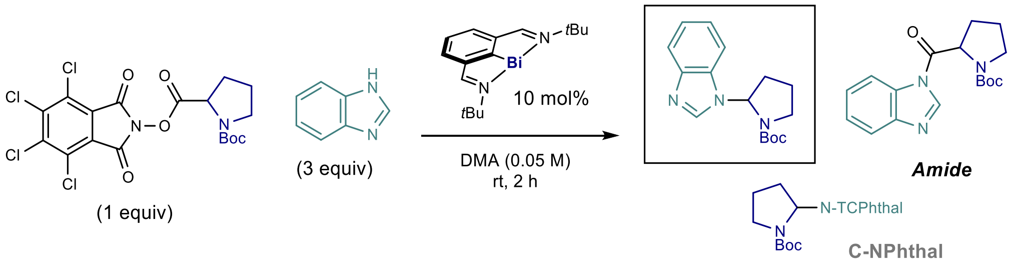

Deviations from Initial Conditions	*Yield C–N (C-NPhthal)*	*Conv. RAE*	*Yield Amide*
initial conditions	87% (10%)	100%	3%
reaction time = 10 min	85% (10%)	100%	5%
reaction time = 1 min	71% (9%)	94%	15%*
total exclusion of ambient light (DMA, rt, 2 h)	86% (9%)	100%	5%
DMF	73% (14%)	100%	13%
DMF, –30 °C, 2 h	56% (19%)	>95%	14%*
NAc instead of NBoc	40% (13%)	80%	20%*
with 2 equiv of vinylphenylsulfone as additive	<10%	70%	57%*

* Probably keeps forming upon quenching/workup from remaining RAE

Tempo inhibition

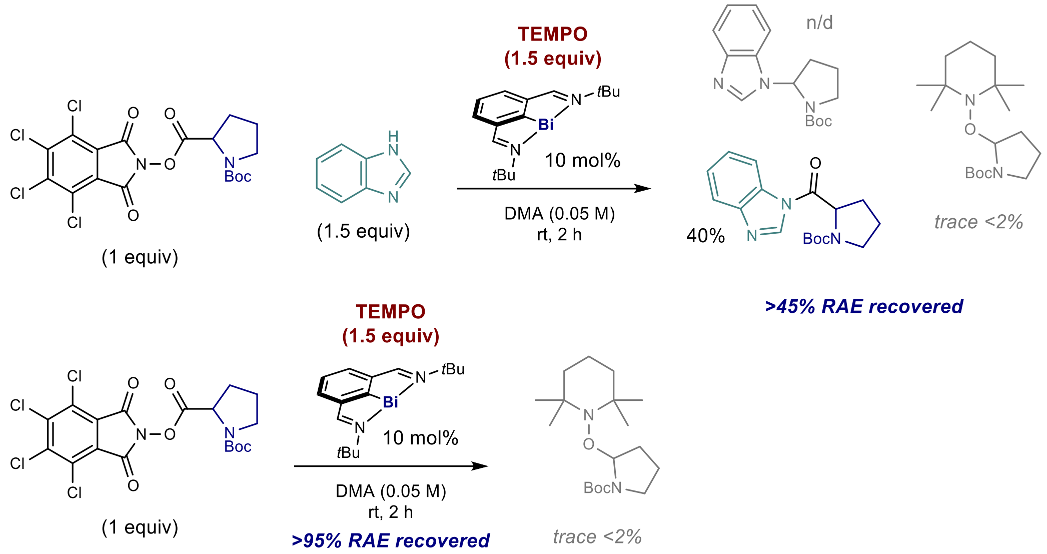

Scope limitations

Without α-heteroatoms, further oxidation of the alkyl radical to a carbocation that can be trapped by the N-nucleophiles is not favorable. Instead, only acyl-transfer (amide) and reduction or homo-coupling (A/B) of the corresponding alkyl radical is observed.

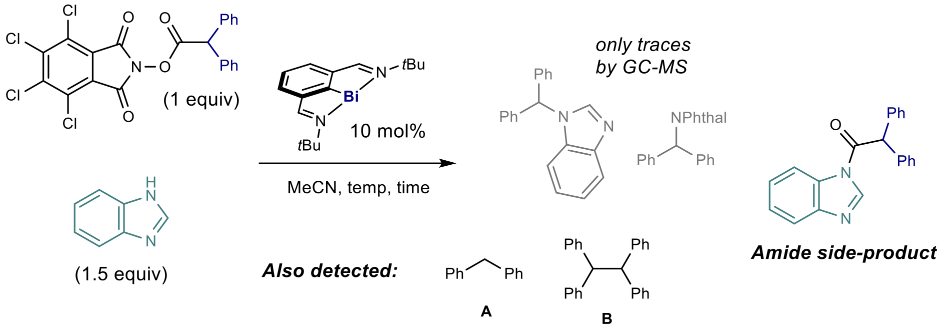

Conditions	*% Amide*	*% A*	*% B*	*Conv. RAE*	*Conv. Nuc*
25 °C, 3 h	62%	-	2%	67%	100%
50 °C, 3 h	60%	-	5%	71%	100%
50 °C, 12 h	58%	2%	4%	65%	100%
50 °C, 12 h, ***no nucleophile ctrl***	0%	3%	5%	13%	n/a
25 °C, 16 h, ***no bismuth ctrl***	67%	0%	0%	67%	100%

Acyl transfer product

1-(1*H*-Benzo[*d*]imidazol-1-yl)-2,2-diphenylethan-1-one (acyl transfer product)

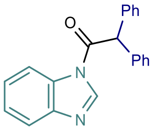

^{1}H NMR (600 MHz, $CDCl_3$) δ 8.29 (s, 1H), 8.24 (ddd, *J* = 8.0, 1.5, 0.7 Hz, 1H), 7.67 (ddd, *J* = 7.6, 1.5, 0.7 Hz, 1H), 7.35 – 7.31 (m, 1H), 7.31 – 7.26 (m, 5H), 7.25 – 7.21 (m, 6H), 5.70 (s, 1H).

^{13}C NMR (151 MHz, $CDCl_3$) δ 169.6, 143.6, 141.3, 137.2, 131.9, 129.3, 128.9, 128.2, 126.3, 125.4, 120.6, 116.1, 58.3.

HRMS (ESI Positive): calculated for $C_{16}H_{22}N_3O_3$ $[M+H]^+$: 312.1257; found: 312.1256.

Furthermore, some of the aminal products of this C–N coupling were found to be unstable. For instance, α-amino tertiary-alkyl coupling products could not be isolated successfully.

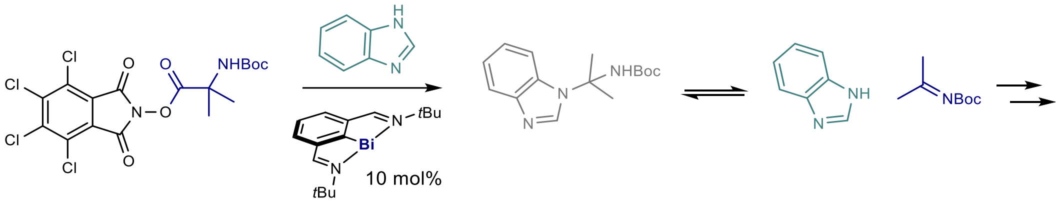

We also found less-nucleophilic amines to result in lower yield of the CN cross-coupling product, due to a more favorable nucleophilic trapping by the released tetrachlorophthalimide.

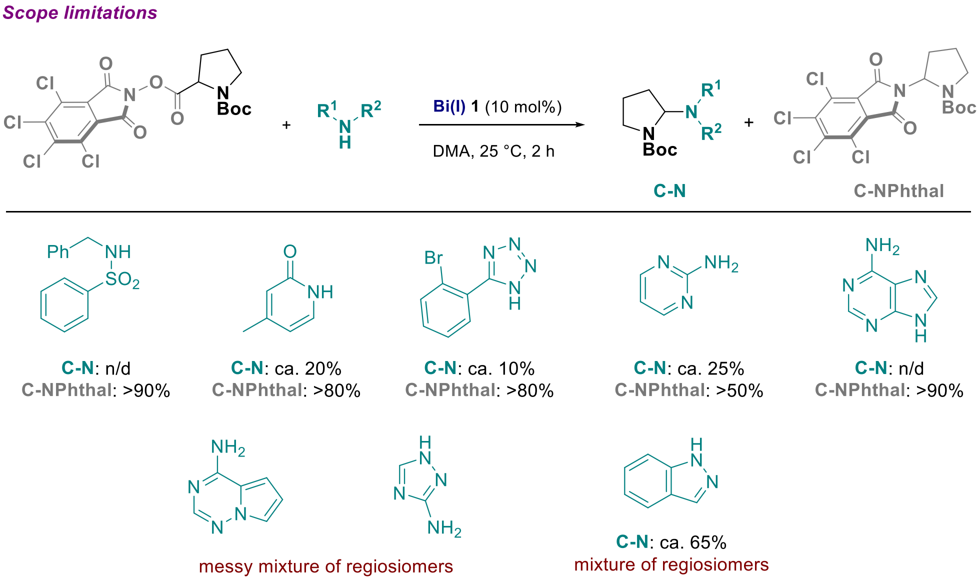

We also tried different non-symmetrical heterocycles, which did react favorably, but often gave mixtures of regioisomers.

Other Control/Mechanistic Experiments

Screening of other Bi(I) compounds as reversible radical transfer catalysts

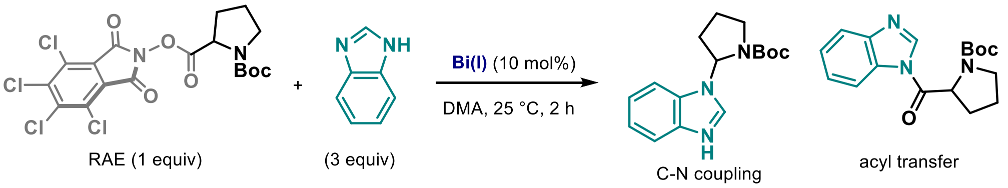

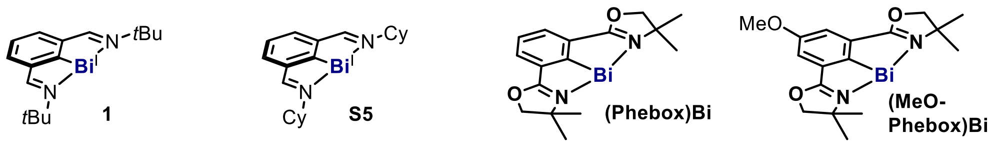

1	S5	(Phebox)Bi	(MeO-Phebox)Bi
**C-N coupling: 86%**	**C-N coupling: 70%**	**C-N coupling: 28%**	**C-N coupling: 10%**
C-NPhthal: 9%	C-NPhthal: 10%	C-NPhthal: <5%	C-NPhthal: <5%
acyl transfer: 5%	acyl transfer: 17%	acyl transfer: 40%	acyl transfer: 45%
conversion RAE: 100%	conversion RAE: 97%	conversion RAE: 73%	conversion RAE: 74%

Screening of some transition metal complexes as radical catalysts

$Co(acac)_2$ (10 mol%)	Co(salen) (10 mol%)	Decamethylferrocene (10 mol%)	Cobaltocene (10 mol%)
**C-N coupling: n/d**	**C-N coupling: n/d**	**C-N coupling: n/d**	**C-N coupling: n/d**
C-NPhthal: n/d	C-NPhthal: n/d	C-NPhthal: n/d	C-NPhthal: n/d
acyl transfer: 50%	acyl transfer: 39%	acyl transfer: 50%	acyl transfer: 36%
conversion RAE: 51%	conversion RAE: 42%	conversion RAE: 50%	conversion RAE: 53%

We tested other bismuth(I) complexes in the reaction (Phebox)Bi and (MeO-Phebox)Bi were prepared according to a reported procedure.[3] Although oxidative addition to these more reducing bismuthinidenes was found to be faster than with **1**, overall diminished yields for the CN coupling are consistent with the lower oxidizing ability of the resulting Bi(II) intermediates formed after SET/fragmentation. For an even closer comparison, we have prepared **S5**, a cyclohexyl derivative of **1**, which is only slightly more reducing than **1** (**S5** –0.9 vs **1** –0.85 V vs Fc0/+) and tested it in the reaction. A faster deactivation was observed, leading to decreased yield in favor of more acyl-transfer product.

All of this highlights the importance of the finely tuned redox properties of **1** as radical catalyst. Also, this catalyst effect supports the involvement of the Bi(II) intermediates in the oxidation of the α-amino radical to the corresponding iminium (see mechanistic studies section).

Finally, other reversible redox-pairs which could potentially promote this transformation were tested. The reaction was therefore attempted using some transition-metal-based redox pair which are either more reducing (cobaltocene) or less reducing (Co(salen), $Co(acac)_2$ or decamethylferrocene) than our bismuth(I) complexes, giving no formation of CN coupling product. This further highlights the properties of bismuth(I) in promoting this transformation: it has enough reducing power to activate the RAEs by SET, and the corresponding bismuth(II) can either stabilize the resulting alkyl radical or directly oxidize it to generate an electrophilic iminium ion that can engage in the CN coupling process (see Section 7 for details on the mechanistic proposal).

Furthermore, as control experiment, we also tested some bismuth(III) pre-catalysts often used in high valent bismuth catalysis. In both cases, the C–N coupling products were not detected, and only acyl transfer product was formed in significant amount.

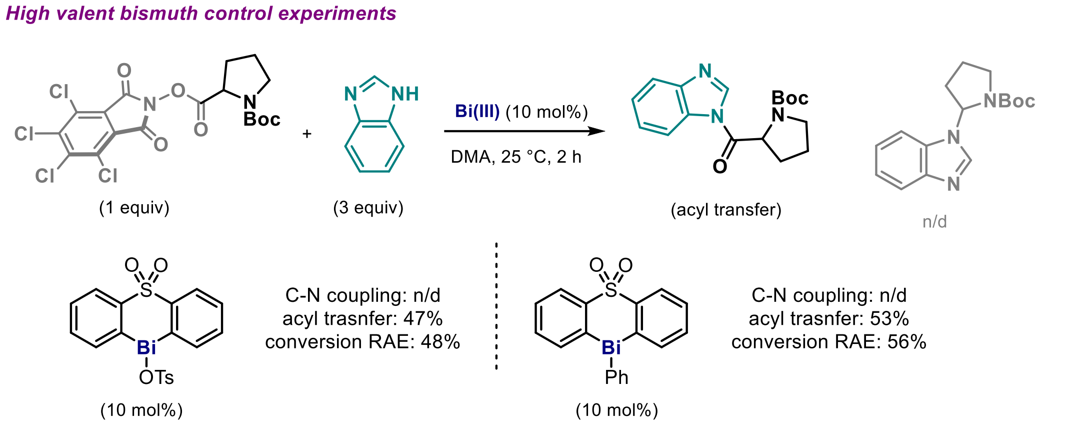

In-situ generation of the active Bi(I) species

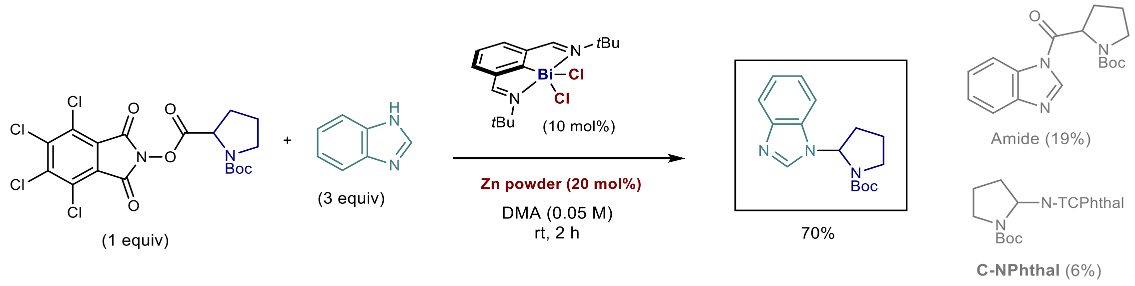

The reaction could also be carried out using bench-stable (L)$BiCl_2$ **S2** as precatalyst, in the presence of 20 mol% of Zn powder as in-situ reductant. After 10–15 min, the initially yellow reaction mixture turns dark green (the typical color of these Bi(I) complexes) and the reaction proceeds as usual, with slightly diminished yield.

6. Bismuth-catalyzed C–N coupling reaction

6.1. General procedure B for the C–N coupling reactions

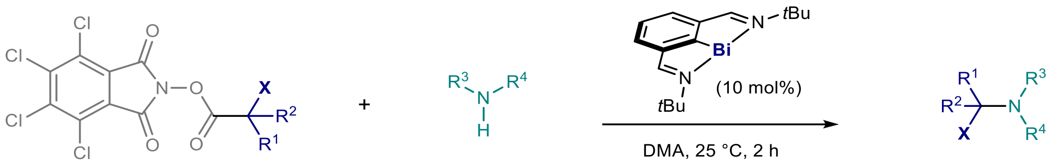

A culture tube with a magnetic stirring bar was charged with the corresponding RAE electrophile (1 equiv, usually 0.2 mmol) and nucleophile (3 equiv), and introduced in an argon-filled glovebox. Bismuth complex **1** (10 mol%) was added, and then everything was dissolved in anhydrous DMA (0.033 M). The vial was closed, taken out of the glovebox, and stirred for 2 h at room temperature. After this time, the mixture was diluted in water and EtOAc, and the organic fraction was washed twice with water and once with brine, dried over anhydrous Na_2SO_4, filtered and concentrated in vacuum. Finally, the product was purified by flash column chromatography or preparative TLC in silica gel.

Additional considerations

- The bismuth-catalyzed reactions were set up in an argon-filled glovebox, where both the catalyst and the solvent (DMA, anhydrous from Sigma-Aldrich) were stored for several months. Alternatively, the reactions can be set up with traditional Schlenk techniques under argon using a stock solution of **1**. A third approach is the in-situ reduction of 10 mol% of the (L)$BiCl_2$ complex, with 20 moL% of Zn powder; after an initial induction period, Bi(I) is formed in situ and the reaction takes place as usual (see previous section).
- Although optimization studies show that the model reaction is finished in <15 min at room temperature, for consistency and to ensure full conversion of other potentially slower substrates, all reactions of the scope were stirred at room temperature for 2 h.
- Unless otherwise stated, all yields refer to spectroscopically pure isolated material in a 0.15–0.30-mmol scale.
- A few products were found to get partially hydrolyzed under acidic conditions (e.g.: silica gel, $CDCl_3$), but this can be solved by neutralizing the silica gel with trimethylamine, or by using CD_2Cl_2 as solvent for NMR characterization.
- Due to the high rotational barrier around the newly formed C–N bond, most of the products are observed by NMR as two rotamers (which generally coalesce at ca. 50–60 °C). This was confirmed by comparison with reported characterization data,[12] and by recording variable-temperature NMR (see next section). Furthermore, for most of the products of the scope, NMR was recorded at lower temperature (usually 233-263 K) in order to sharpen the signals of each rotamer. However, many NMR signals are difficult to resolve and assign (especially in ^{13}C NMR, when rotameric mixtures are close to 1:1 ratio), so they are reported together for both rotamers in some products. Some pairs of ^{13}C signals could not be resolved and appear as a single one. In the cases where all signals could be resolved and assigned for each rotamer, the signals are reported for the major rotamer. For the signals of the minor rotamer, see the copy of the corresponding NMR spectra.

6.2. Characterization data for the C–N coupling products

***tert*-Butyl 2-(1*H*-benzo[*d*]imidazol-1-yl)pyrrolidine-1-carboxylate** (**26**)

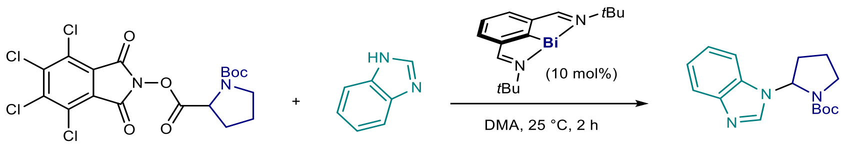

The title product was obtained as a white solid in 90% yield (52 mg) from redox-active ester **22** (100 mg, 0.20 mmol, 1 equiv) and benzimidazole (71 mg, 0.60 mmol, 3 equiv) using bismuth complex **1** as catalyst (9 mg, 0.020 mmol, 10 mol%) in DMA (0.033 M) following General Procedure B, after flash column chromatography in silica gel using hexanes/EtOAc (gradient from 7:3 to 2:8; R_f = 0.15 in hexanes/EtOAc 1:1). Characterization data matched the reported ones.[12]

^{1}H NMR (400 MHz, $CDCl_3$, mixture of rotamers) δ 7.91 (s, 1H), 7.85 – 7.76 (m, 1H), 7.40 (s, 1H), 7.32 – 7.26 (m, 2H), 6.39 – 6.03 (m, 1H), 3.89 – 3.51 (m, 2H), 2.49 – 2.15 (m, 2H), 2.15 – 2.01 (m, 2H), 1.16 (s, 9H).

^{13}C NMR (101 MHz, $CDCl_3$, major rotamer) δ 153.4, 144.2, 140.9, 122.9, 122.2, 120.5, 109.9, 81.1, 69.0, 46.6, 33.7, 28.0, 22.5.

FTIR (ATR): $\tilde{\nu}$ [cm^{-1}] = 3083, 2986, 2973, 1698, 1479, 1456, 1426, 1388, 1364, 1282, 1207, 1163, 1127, 1007, 912, 877, 774, 737, 535, 466, 421.

HRMS (ESI Positive): calculated for $C_{16}H_{22}N_3O_3$ [M+H]$^+$: 288.1705; found: 288.1707.

MP: 108–110 °C.

***tert*-Butyl 2-(N-methoxy-1*H*-benzo[*d*]imidazol-1-yl)pyrrolidine-1-carboxylate** (N = 5/6 ca. 1:1, **28**)

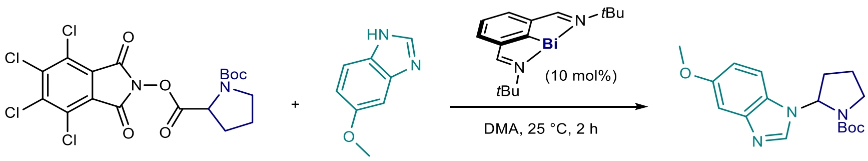

The title product was obtained as a yellow residue in 63% yield (40 mg, 1:1 mixture of *N*-regioisomers) from redox-active ester **22** (100 mg, 0.20 mmol, 1 equiv) and 5-methoxy-1*H*-benzo[*d*]imidazole (89 mg, 0.60 mmol, 3 equiv) using bismuth complex **1** as catalyst (9 mg, 0.020 mmol, 10 mol%) in DMA (0.033 M) following General Procedure B, after flash column chromatography in silica gel using hexanes/EtOAc (gradient from 7:3 to 1:9); R_f = 0.1 in hexanes/EtOAc 1:1).

^{1}H NMR (600 MHz, $CDCl_3$, 353 K, NMR measured at high temperature to converge the rotamers of each regioisomer; ca. 1:1 mixture of reg1/reg2 *N*-regioisomers) δ 7.82 (s, 1H), 7.77 (s, 1H), 7.65 (d, *J* = 8.8 Hz, 1H), 7.28 (d, *J* = 2.2 Hz, 1H), 7.25 (d, *J* = 9.0 Hz, 1H), 6.94 – 6.89 (m, 2H), 6.87 (s, 1H), 6.11 (s, 2H), 3.86 (s, 3H), 3.85 (s, 3H), 3.76 (ddt, *J* = 11.6, 7.9, 4.1 Hz, 2H), 3.63 (q, *J* = 8.7 Hz, 2H), 2.38 (dq, *J* = 16.1, 8.0 Hz, 2H), 2.21 (ddt, *J* = 13.3, 6.6, 3.4 Hz, 2H), 2.06 (pd, *J* = 12.9, 7.5 Hz, 4H), 1.29 (s, 19H).

^{13}C NMR (151 MHz, $CDCl_3$, 353 K, NMR measured at high temperature to converge the rotamers of each regioisomer; reg2 signals between parenthesis) δ 157.1, (156.6), 153.9, (153.9) 145.6, 141.2, (140.2), (139.4), 133.4, (127.4), 121.1, (113.3), 111.5, (110.4), (103.4), 94.7, 81.2 (81.2), (69.4), 69.2, 56.2, (56.1), 47.0, (46.9), 33.6, (33.5), 28.4, (28.4), 23.0, (23.0).

HRMS (EI): calculated for $C_{17}H_{23}N_3O_3$ [M]$^+$: 317.1734; found: 317.1732.

Benzyl (1-(1*H*-benzo[*d*]imidazol-1-yl)-2-phenylethyl)carbamate (29)

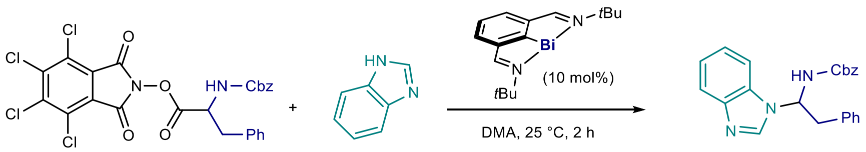

The title product was obtained as a white solid in 51% yield (38 mg) from redox-active ester **S11** (116 mg, 0.20 mmol, 1 equiv) and benzimidazole (71 mg, 0.60 mmol, 3 equiv) using bismuth complex **1** as catalyst (9 mg, 0.020 mmol, 10 mol%) in DMA (0.033 M) following General Procedure B, after preparative TLC in silica gel using hexanes/EtOAc 3:7; (R_f = 0.15 in hexanes/EtOAc 1:1).

^{1}H NMR (400 MHz, $CDCl_3$, mixture of rotamers) δ 7.93 – 7.75 (m, 2H), 7.62 – 7.46 (m, 1H), 7.34 – 7.14 (m, 10H), 7.02 – 6.88 (m, 2H), 6.35 – 6.24 (m, 1H), 5.11 – 4.92 (m, 2H), 3.48 – 3.35 (m, 2H).

^{13}C NMR (101 MHz, $CDCl_3$, mixture of rotamers) δ 155.1, 143.9, 141.2, 135.6, 134.3, 132.3, 128.9, 128.9, 128.6, 128.4, 128.2, 127.6, 123.2, 122.5, 120.5, 110.6, 67.5, 64.4, 40.4.

FTIR (ATR): $\tilde{\nu}$ [cm^{-1}] = 3189, 3097, 3031, 2952, 2920, 2850, 1715, 1553, 1495, 1454, 1282, 1245, 1048, 1022, 933, 743, 697, 652, 622, 528,483, 430.

HRMS (ESI Positive): calculated for $C_{23}H_{22}N_3O_2$ [M+H]$^+$: 372.17065; found: 372.17073.

MP: 132–134 °C.

Benzyl (1-(1*H*-benzo[*d*]imidazol-1-yl)-2-methylpropyl)(methyl)carbamate (30)

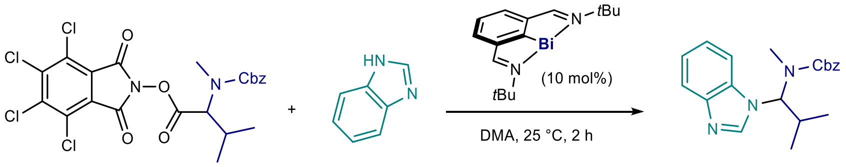

The title product was obtained as an off-white solid in 71% yield (48 mg) from redox-active ester **S10** (110 mg, 0.20 mmol, 1 equiv) and benzimidazole (71 mg, 0.60 mmol, 3 equiv) using bismuth complex **1** as catalyst (9 mg, 0.020 mmol, 10 mol%) in DMA (0.033 M) following General Procedure B, after flash column chromatography in silica gel using hexanes/EtOAc (gradient from 7:3 to 3:7; R_f = 0.30 in hexanes/EtOAc 3:7).

^{1}H NMR (600 MHz, $CDCl_3$, mixture of rotamers) δ 8.13 (s, 1H), 7.84 – 7.40 (m, 3H), 7.38 – 7.27 (m, 5H), 6.18 (d, *J* = 11.1 Hz, 1H), 5.42 – 5.11 (m, 2H), 2.79 – 2.75 (m, 1H), 2.77 – 2.70 (m, 3H), 1.16 – 0.89 (m, 6H).

^{13}C NMR (151 MHz, $CDCl_3$, major rotamer) δ 156.5, 143.1, 140.5, 136.2, 133.9, 128.6, 128.2, 127.8, 123.8, 122.9, 120.1, 111.3, 71.7, 67.8, 28.6, 27.8, 19.3.

FTIR (ATR): $\tilde{\nu}$ [cm^{-1}] = 3062, 2941, 2875, 1692, 1608, 1474, 1458, 1419, 1264, 1216, 1032, 955, 739, 693, 594, 497, 430.

HRMS (ESI Positive): calculated for $C_{20}H_{24}N_3O_2$ [M+H]$^+$: 338.18630; found: 338.18632.

MP: 102–106 °C.

Benzyl 2-(1*H*-benzo[*d*]imidazol-1-yl)piperidine-1-carboxylate (31)

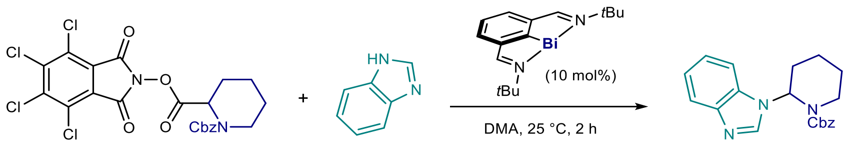

The title product was obtained as a colorless residue in 82% yield (55 mg) from redox-active ester **S9** (109 mg, 0.20 mmol, 1 equiv) and benzimidazole (71 mg, 0.60 mmol, 3 equiv) using bismuth complex **1** as catalyst (9 mg, 0.020 mmol, 10 mol%) in DMA (0.033 M) following General Procedure B, after flash column chromatography in silica gel using hexanes/EtOAc (gradient from 7:3 to 2:8; R_f = 0.20 in hexanes/EtOAc 3:7).

^{1}H NMR (600 MHz, $CDCl_3$, mixture of rotamers) δ 8.14 (s, 1H), 7.82 – 7.77 (m, 1H), 7.48 – 7.40 (m, 1H), 7.38 – 7.30 (m, 5H), 7.29 – 7.26 (m, 1H), 7.23 – 7.19 (m, 1H), 6.69 – 6.62 (m, 1H), 5.21 (s, 2H), 4.16 – 4.10 (m, 1H), 2.87 – 2.79 (m, 1H), 2.47 – 2.41 (m, 1H), 2.18 (ddt, *J* = 14.9, 12.7, 5.4 Hz, 1H), 1.95 – 1.76 (m, 3H), 1.65 (tdt, *J* = 13.2, 11.8, 4.8 Hz, 1H).

^{13}C NMR (151 MHz, $CDCl_3$, major rotamer) δ 154.9, 143.5, 141.0, 136.0, 133.4, 128.7, 128.4, 128.2, 123.4, 122.6, 120.2, 111.3, 68.0, 62.7, 40.0, 28.2, 24.4, 19.5.

FTIR (ATR): $\tilde{\nu}$ [cm^{-1}] = 3064, 3033, 2968, 1964, 1613, 1455, 1385, 1328, 1305, 1279, 1223, 1158, 1128, 1110, 974, 770, 742, 697,609, 499, 425.

HRMS (ESI Positive): calculated for $C_{20}H_{22}N_3O_2$ $[M+H]^+$: 336.17065; found: 336.17049.

***tert*-Butyl 2-(4-(4,4,5,5-tetramethyl-1,3,2-dioxaborolan-2-yl)-1*H*-pyrazol-1-yl)pyrrolidine-1-carboxylate (32)**

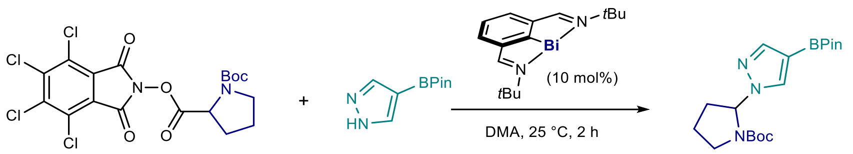

The title product was obtained as a colorless oil in 76% yield (55 mg) from redox-active ester **22** (100 mg, 0.20 mmol, 1 equiv) and 4-(4,4,5,5-tetramethyl-1,3,2-dioxaborolan-2-yl)-1*H*-pyrazole (116 mg, 0.60 mmol, 3 equiv) using bismuth complex **1** as catalyst (9 mg, 0.020 mmol, 10 mol%) in DMA (0.033 M) following General Procedure B, after flash column chromatography in silica gel using hexanes/EtOAc (gradient from 8:2 to 6:4; R_f = 0.45 in hexanes/EtOAc 6:4).

^{1}H NMR (400 MHz, $CDCl_3$, mixture of rotamers) δ 7.77 (s, 2H), 6.15 – 5.89 (m, 1H), 3.75 – 3.42 (m, 2H), 2.37 – 2.14 (m, 3H), 1.99 – 1.93 (m, 1H), 1.47 – 1.30 (m, 21H).

^{13}C NMR (101 MHz, $CDCl_3$, major rotamer) δ 153.5, 145.5, 134.7, 83.2, 80.6, 73.3, 46.4, 33.7, 28.1, 24.7, 23.2, 22.1.

FTIR (ATR): $\tilde{\nu}$ [cm^{-1}] = 2978, 2932, 1965, 1556, 1408, 1369, 1285, 1248, 1163, 1143, 1122, 1111, 1082, 988, 892, 857, 777, 692, 669, 552, 455.

HRMS (ESI Positive): calculated for $C_{18}H_{30}N_3O_4BNa$ $[M+Na]^+$: 386.22215; found: 386.22197.

Benzyl 2-(4-(4,4,5,5-tetramethyl-1,3,2-dioxaborolan-2-yl)-1*H*-pyrazol-1-yl)piperidine-1-carboxylate (33)

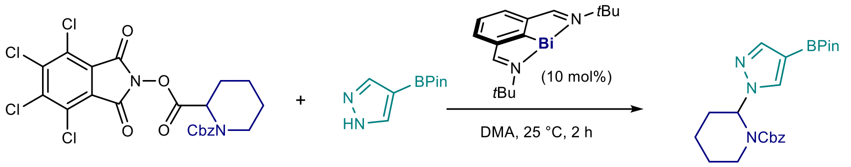

The title product was obtained as a pale-yellow oil in 62% yield (50 mg) from redox-active ester **S9** (109 mg, 0.20 mmol, 1 equiv) and 4-(4,4,5,5-tetramethyl-1,3,2-dioxaborolan-2-yl)-1*H*-pyrazole (116 mg, 0.60 mmol, 3 equiv) using bismuth complex **1** as catalyst (9 mg, 0.020 mmol, 10 mol%) in DMA (0.033 M) following General Procedure B, after flash column chromatography in silica gel using hexanes/EtOAc (gradient from 9:1 to 7:3; R_f = 0.60 in hexanes/EtOAc 6:4).

^{1}H NMR (500 MHz, CD_2Cl_2, 233 K, mixture of rotamers) δ 7.90 – 7.71 (m, 2H), 7.42 – 7.30 (m, 5H), 6.40 – 6.35 (m, 1H), 5.22 – 5.08 (m, 2H), 4.14 – 4.02 (m, 1H), 2.99 – 2.83 (m, 1H), 2.59 – 2.51 (m, 1H), 1.95 – 1.51 (m, 5H), 1.31 – 1.29 (m, 12H).

^{13}C NMR (126 MHz, CD_2Cl_2, 233 K, mixture of rotamers) δ 155.6, 154.9, 144.9, 144.8, 136.2, 136.1, 135.4, 134.8, 128.48, 128.45, 128.1, 128.0, 127.9, 127.8, 83.2, 67.5, 67.4, 66.6, 66.0, 40.2, 39.9, 28.2, 27.9, 24.5, 24.4, 24.2, 24.1, 18.5, 18.2.

FTIR (ATR): $\tilde{\nu}$ [cm^{-1}] = 2976, 1702, 1555, 1407, 1370, 1257, 1219, 1168, 1139, 1041, 984, 856, 735, 692, 668, 603, 450.

HRMS (ESI Positive): calculated for $C_{22}H_{30}N_3O_4BNa$ [M+Na]$^+$: 434.22215; found: 434.22243.

***tert*-Butyl (1-(1*H*-benzo[*d*]imidazol-1-yl)-2-methylpropyl)carbamate (34)**

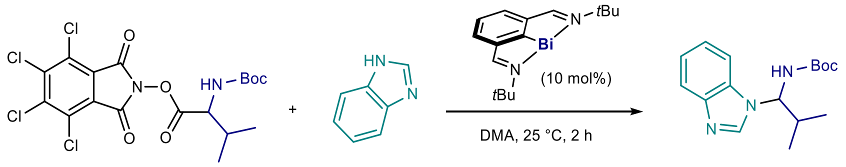

The title product was obtained as a-white foamy solid in 55% yield (33 mg) from redox-active ester **S17** (100 mg, 0.20 mmol, 1 equiv) and benzimidazole (71 mg, 0.60 mmol, 3 equiv) using bismuth complex **1** as catalyst (9 mg, 0.020 mmol, 10 mol%) in DMA (0.033 M) following General Procedure B, after flash column chromatography in silica gel using hexanes/EtOAc (gradient from 7:3 to 3:7; R_f = 0.45 in hexanes/EtOAc 4:6).

^{1}H NMR (600 MHz, $CDCl_3$, 263 K, ca. 2:1 mixture of rotamers *rot1*/*rot2*) δ 8.05 (s, 1H, *rot1*), 7.97 (s, 1H, *rot2*), 7.84 – 7.76 (m, 1H *rot1+2*), 7.59 – 7.47 (m, 1H *rot1+2*), 7.30 – 7.16 (m, 2H *rot1+2*), 6.02 (d, *J* = 8.9 Hz, 1H *rot1*), 5.66 (t, *J* = 9.2 Hz, 1H *rot1*), 5.41 – 5.30 (m, 1H *rot2*), 2.49 (dp, *J* = 9.4, 6.6 Hz, 1H *rot1*), 2.42 (dt, *J* = 9.3, 6.6 Hz, 1H *rot2*), 1.36 (s, 9H *rot1*), 1.28 (s, 9H *rot2*), 1.15 (d, *J* = 6.6 Hz, 3H *rot1*), 1.12 (d, *J* = 6.6 Hz, 3H *rot2*), 0.78 (d, *J* = 6.6 Hz, 3H *rot1*), 0.72 (d, *J* = 6.7 Hz, 3H *rot2*).

^{13}C NMR (151 MHz, $CDCl_3$, 263 K, ca. 2:1 mixture of rotamers, minor rotamer in parenthesis) δ (155.4), 154.8, 143.8, (142.2), 141.8, 132.5, (132.3), 123.1, (122.9), 122.4, (122.3), (120.4), (120.3), (110.9), 110.8, (81.6), 80.8, (70.4), 68.7, 32.5, (32.4), 28.3, (28.1), (19.4), 19.2, 19.1, (18.8).

FTIR (ATR): $\tilde{\nu}$ [cm^{-1}] = 3196, 2972, 2931, 2873, 1704, 1615, 1537, 1494, 1453, 1365, 1309, 1271, 1248, 1154, 1041, 1013, 898, 876, 800, 758, 740, 634, 488, 453, 433.

HRMS (ESI Positive): calculated for $C_{16}H_{24}N_3O_2$ [M+H]$^+$: 290.18630; found: 290.18622.

tert-Butyl (1-(1*H*-benzo[*d*]imidazol-1-yl)-3-methylbutyl)carbamate (**35**)

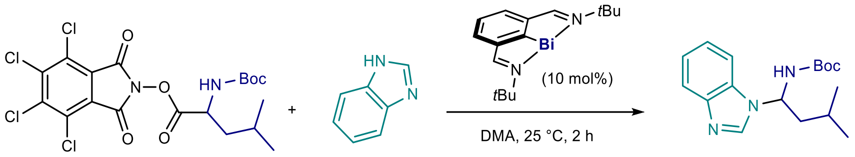

The title product was obtained as a-white foamy solid in 48% yield (29 mg) from redox-active ester **S18** (103 mg, 0.20 mmol, 1 equiv) and benzimidazole (71 mg, 0.60 mmol, 3 equiv) using bismuth complex **1** as catalyst (9 mg, 0.020 mmol, 10 mol%) in DMA (0.033 M) following General Procedure B, after flash column chromatography in silica gel using hexanes/EtOAc (gradient from 7:3 to 3:7; R_f = 0.4 in hexanes/EtOAc 4:6).

^{1}H NMR (600 MHz, $CDCl_3$, 263 K, major rotamer, ca. 4:1) δ 8.08 (s, 1H), 7.81 – 7.79 (m, 1H), 7.63 – 7.59 (m, 1H), 7.30 (tt, *J* = 7.1, 5.5 Hz, 2H), 6.13 – 6.08 (m, 1H), 5.76 (d, *J* = 8.8 Hz, 1H), 2.09 (dt, *J* = 14.3, 7.1 Hz, 1H), 2.02 – 1.97 (m, 1H), 1.53 – 1.48 (m, 1H), 1.37 (s, 9H), 0.95 (dd, *J* = 8.5, 6.6 Hz, 6H).

^{13}C NMR (151 MHz, $CDCl_3$, 263 K, major rotamer, ca. 4:1) δ 154.5, 143.6, 141.2, 132.5, 123.2, 122.5, 120.3, 110.9, 80.8, 61.3, 43.1, 28.2, 24.7, 22.4, 22.3.

FTIR (ATR): $\tilde{\nu}$ [cm^{-1}] = 3211, 2962, 2873, 1710, 1533, 1494, 1454, 1425, 1325, 1279, 1253, 1156, 1045, 1009, 906, 863, 748, 709, 538, 430.

HRMS (ESI Positive): calculated for $C_{17}H_{26}N_3O_2$ $[M+H]^+$: 304.20195; found: 304.20193.

tert-Butyl 4-(1*H*-benzo[*d*]imidazol-1-yl)-4-((*tert*-butoxycarbonyl)amino)butanoate (**36**)

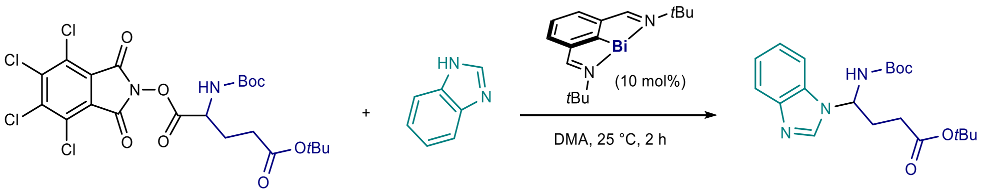

The title product was obtained as a white foamy solid in 64% yield (48 mg) from redox-active ester **S19** (117 mg, 0.20 mmol, 1 equiv) and benzimidazole (71 mg, 0.60 mmol, 3 equiv) using bismuth complex **1** as catalyst (9 mg, 0.020 mmol, 10 mol%) in DMA (0.033 M) following General Procedure B, after flash column chromatography in silica gel using hexanes/EtOAc (gradient from 7:3 to 3:7; R_f = 0.30 in hexanes/EtOAc 4:6).

^{1}H NMR (600 MHz, $CDCl_3$, 263 K, major rotamer, ca. 4:1) δ 8.09 (s, 1H), 7.82 – 7.79 (m, 1H), 7.65 – 7.57 (m, 1H), 7.31 – 7.26 (m, 2H), 6.19 (d, *J* = 8.5 Hz, 1H), 6.06 (qd, *J* = 7.4, 4.5 Hz, 1H), 2.57 – 2.50 (m, 1H), 2.37 – 2.28 (m, 3H), 1.43 (s, 9H), 1.36 (s, 9H).

^{13}C NMR (151 MHz, $CDCl_3$, 263 K, major rotamer, ca. 4:1) δ 172.18, 154.61, 143.73, 141.23, 132.26, 123.32, 122.59, 120.36, 110.88, 81.60, 80.86, 62.71, 31.24, 29.00, 28.24, 28.06.

FTIR (ATR): $\tilde{\nu}$ [cm^{-1}] = 3208, 2977, 2932, 1711, 1614, 1523, 1491, 1456, 1366, 1274, 1249, 1157, 1049, 896, 846, 740, 644, 426.

HRMS (ESI Positive): calculated for $C_{20}H_{30}N_3O_4$ $[M+H]^+$: 376.22308; found: 376.22336.

tert-Butyl 2-(1_H_-1,2,4-triazol-1-yl)pyrrolidine-1-carboxylate (37)

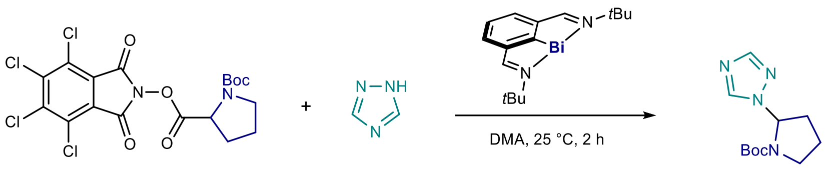

The title product was obtained as a colorless oil in 50% yield (24 mg) from redox-active ester **22** (100 mg, 0.20 mmol, 1 equiv) and 1*H*-1,2,4-triazole (42 mg, 0.60 mmol, 3 equiv) using bismuth complex **1** as catalyst (9 mg, 0.020 mmol, 10 mol%) in DMA (0.033 M) following General Procedure B, after flash column chromatography in silica gel using hexanes/EtOAc (gradient from 7:3 to 2:8; R_f = 0.3 in hexanes/EtOAc 3:7).

^{1}H NMR (600 MHz, $CDCl_3$, 263 K, 1:1 mixture of unassigned rotamers) δ 8.31 (s, 1H, rot1), 8.13 (s, 1H, rot2), 7.94 (s, 1H, rot1), 7.92 (s, 1H, rot2), 6.10 (d, *J* = 7.0 Hz, 1H, rot1), 6.00 (t, *J* = 4.1 Hz, 1H, rot2), 3.69 (ddd, *J* = 10.6, 8.2, 2.5 Hz, 1H, rot1), 3.63 – 3.58 (m, 1H, rot2), 3.48 (ddd, *J* = 10.5, 9.4, 7.2 Hz, 1H, rot1), 3.40 (td, *J* = 9.8, 7.0 Hz, 1H, rot2), 2.42 – 2.25 (m, 6H, rot1+2), 2.04 (dtd, *J* = 9.6, 7.6, 5.2 Hz, 2H, rot1+2), 1.41 (s, 9H, rot1), 1.36 (s, 9H, rot2).

^{13}C NMR (151 MHz, CDCl3, 263 K, 1:1 mixture of unassigned rotamers) δ 154.28, 153.25, 152.15, 151.82, 143.67, 142.60, 81.43, 80.98, 71.47, 71.07, 46.89, 46.57, 33.19, 31.70, 28.32, 28.25, 23.58, 22.38.

FTIR (ATR): $\tilde{\nu}$ [cm^{-1}] = 2977, 2933, 2890, 1696, 1504, 1378, 1367, 1256, 1160, 1117, 1007, 876, 774, 664, 563, 440.

HRMS (ESI Positive): calculated for $C_{11}H_{18}N_4NaO_2$ $[M+Na]^+$: 261.13220; found: 261.13226.

tert-Butyl 2-(4,5-diphenyl-1_H_-imidazol-1-yl)pyrrolidine-1-carboxylate (38)

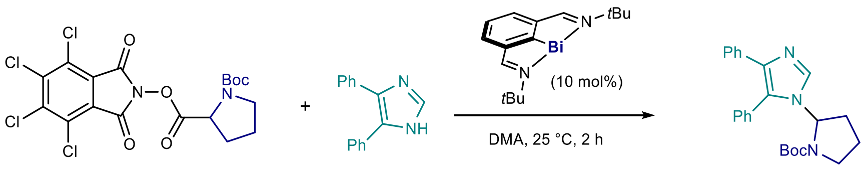

The title product was obtained as a white solid in 82% yield (65 mg) from redox-active ester **22** (100 mg, 0.20 mmol, 1 equiv) and 4,5-diphenyl-1*H*-imidazole (132 mg, 0.60 mmol, 3 equiv) using bismuth complex **1** as catalyst (9 mg, 0.020 mmol, 10 mol%) in DMA (0.033 M) following General Procedure B, after flash column chromatography in silica gel using hexanes/EtOAc (gradient from 6:4 to 2:8; R_f = 0.4 in hexanes/EtOAc 2:8).

^{1}H NMR (500 MHz, CD_2Cl_2, 233 K, mixture of rotamers) δ 7.90 – 7.65 (m, 2H), 7.60 – 7.42 (m, 5H), 7.31 – 7.21 (m, 1H), 7.10 – 7.05 (m, 1H), 7.04 – 6.99 (m, 1H), 6-98 – 6.85 (m, 1H), 6.08 – 5.87 (m, 1H), 3.78 – 3.65 (m, 1H), 3.45 – 3.35 (m, 1H), 2.46 – 2.21 (m, 1H), 2.21 – 1.92 (m, 3H), 1.24 (s, 9H).

^{13}C NMR (126 MHz, CD_2Cl_2, 233 K, mixture of rotamers) δ 153.9, 153.0, 146.59, 146.54, 130.5, 130.4, 129.0, 128.9, 128.69, 128.62, 128.5, 128.2, 128.0, 127.8, 117.0, 116.9, 80.3, 80.1, 69.3, 69.1, 47.0, 46.5, 35.4, 34.3, 27.8, 27.6, 22.8, 22.0.

HRMS (ESI Positive): calculated for $C_{24}H_{28}N_3O_2$ $[M+H]^+$: 390.21760; found: 390.21772.

FTIR (ATR): $\tilde{\nu}$ [cm^{-1}] = 3099, 2973, 2904, 2880, 1695, 1601, 1481, 1368, 1340, 1231, 1158, 1088, 891, 767, 596, 667, 585, 551, 449.

MP: 150–152 °C.

tert-Butyl 2-(1H-benzo[d][1,2,3]triazol-1-yl)pyrrolidine-1-carboxylate (39)

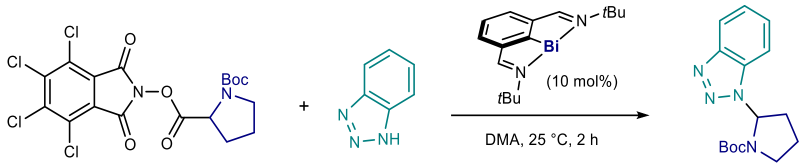

The title product was obtained as a pale-yellow oil in 65% yield (33 mg, 5:1 mixture of N1/N2 regioisomers, each as a pair of rotamers) from redox-active ester **22** (85 mg, 0.17 mmol, 1 equiv) and 1,2,3-benzotriazole (61 mg, 0.51 mmol, 3 equiv) using bismuth complex **1** as catalyst (8 mg, 0.017 mmol, 10 mol%) in DMA (0.033 M) following General Procedure B, after preparative TLC in silica gel using hexanes/EtOAc 1:1. Both the crude and isolated material were obtained as a 5:1 mixture of N1- and N2-alkylated products, each as a pair of rotamers (see NMR spectra). Major product is reported.[12]

^{1}H NMR (600 MHz, $CDCl_3$, 263 K, major regioisomer, mixture of rotamers) δ 8.10 – 8.03 (m, 1H), 7.84 – 7.45 (m, 2H), 7.39 – 7.35 (m, 1H), 6.63 – 6.56 (m, 1H), 3.94 – 3.75 (m, 1H), 3.68 – 3.55 (m, 1H), 2.73 – 2.54 (m, 1H), 2.47 – 2.30 (m, 2H), 2.19 – 2.10 (m, 1H), 1.40 (s, 4H, minor rotamer, major regioisomer), 1.07 (s, 5H, major rotamer, major regioisomer).

^{13}C NMR (151 MHz, $CDCl_3$, 263 K, major regioisomer, major rotamer) δ 153.3, 145.4, 131.8, 127.4, 124.1, 120.0, 109.8, 81.1, 71.4, 46.8, 34.6, 28.0, 22.8. **^{13}C NMR** (151 MHz, $CDCl_3$, major regioisomer, minor rotamer) δ 154.3, 145.0, 132.9, 127.6, 124.2, 119.4, 113.5, 110.6, 80.8, 70.4, 47.0, 33.1, 28.3, 27.3, 23.8.

FTIR (ATR): $\tilde{\nu}$ [cm^{-1}] = 3083, 3028, 2979, 2905, 1700, 1614, 1387, 1367, 1259, 1209, 1161, 1008, 777, 743, 701, 606, 430.

HRMS (ESI Positive): calculated for $C_{15}H_{20}N_4O_2Na$ $[M+Na]^+$: 311.14784; found: 311.14769.

tert-Butyl 2-(4-bromo-1H-pyrazol-1-yl)pyrrolidine-1-carboxylate (40)

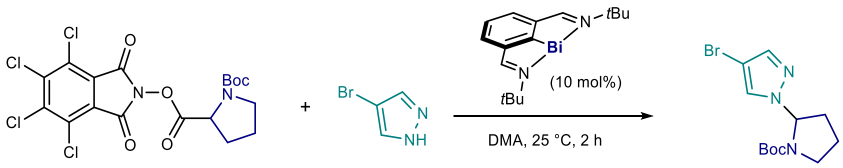

The title product was obtained as a pale yellow oil in 86% yield (54 mg) from redox-active ester **22** (100 mg, 0.20 mmol, 1 equiv) and 4-bromo-1*H*-pyrazole (89 mg, 0.60 mmol, 3 equiv) using bismuth complex **1** as catalyst (9 mg, 0.020 mmol, 10 mol%) in DMA (0.033 M) following General Procedure B, after flash column chromatography in silica gel using hexanes/EtOAc (gradient from 95:5 to 8:2; R_f = 0.50 in hexanes/EtOAc 8:2).

^{1}H NMR (400 MHz, $CDCl_3$, mixture of rotamers) δ 7.57 – 7.38 (m, 2H), 5.97 – 5.77 (m, 1H), 3.68 – 3.29 (m, 2H), 2.37 – 1.88 (m, 4H), 1.34 (s, 9H).

^{13}C NMR (101 MHz, $CDCl_3$, major rotamer) δ 153.4, 140.0, 128.1, 92.4, 80.9, 73.8, 46.5, 33.5, 28.2, 22.2.

FTIR (ATR): $\tilde{\nu}$ [cm^{-1}] = 3255, 3122, 2977, 2933, 2888, 1696, 1365, 1377, 1311, 1252, 1158, 1107, 952, 889, 844, 797, 773, 709, 614, 560, 437.

HRMS (ESI Positive): calculated for $C_{12}H_{18}N_3O_2BrNa$ $[M+Na]^+$: 338.04747; found: 338.04724.

tert-Butyl 2-(4-chloro-1_H_-pyrazol-1-yl)pyrrolidine-1-carboxylate (**41**)

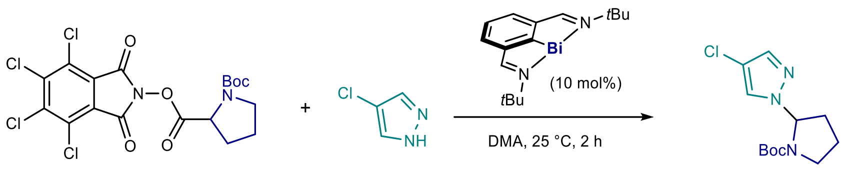

The title product was obtained as a pale-yellow oil in 73% yield (30 mg) from redox-active ester **22** (75 mg, 0.15 mmol, 1 equiv) and 4-chloro-1*H*-pyrazole (46 mg, 0.45 mmol, 3 equiv) using bismuth complex **1** as catalyst (7 mg, 0.015 mmol, 10 mol%) in DMA (0.033 M) following General Procedure B, after flash column chromatography in silica gel using hexanes/EtOAc (gradient from 95:5 to 8:2; R_f = 0.50 in hexanes/EtOAc 8:2).

^{1}**H NMR** (600 MHz, CD_2Cl_2, 253 K, mixture of rotamers) δ 7.59 – 7.46 (m, 1H), 7.43 – 7.38 (m, 1H), 5.92 – 5.90 (m, "0.5 H", *first rotamer*), 5.81 (dd, *J* = 6.4, 1.9 Hz, "0.5 H", *second rotamer*), 3.61 – 3.53 (m, 1H), 3.41 – 3.32 (m, 1H), 2.28 – 2.11 (m, 3H), 1.97 – 1.92 (m, 1H), 1.40 (s, "4.5 H", *first rotamer*).1.31 (s, "4.5 H", *second rotamer*).

^{13}**C NMR** (151 MHz, CD_2Cl_2, 253 K, mixture of rotamers, second signal of each pair in parenthesis) δ 154.3, (153.3), 137.67, (137.65), 126.9, (126.3), 108.9, (108.7), 80.5, (80.4), 73.8, (73.6), 47.0, (46.6), 33.5, (32.2), 28.0, (27.9), 23.4, (22.3).

FTIR (ATR): $\tilde{\nu}$ [cm^{-1}] = 3119, 2977, 2933, 2890, 1696, 1454, 1365, 1317, 1253, 1158, 1109, 966, 899, 841, 773, 712, 617, 561, 437.

HRMS (ESI Positive): calculated for $C_{12}H_{18}N_3O_2ClNa$ [M+Na]$^+$: 294.09797; found: 294.09793.

tert-Butyl 2-(4-bromo-3,5-dimethyl-1_H_-pyrazol-1-yl)pyrrolidine-1-carboxylate (**42**)

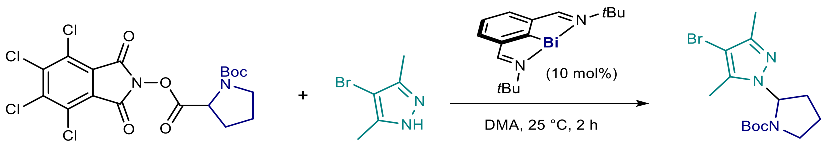

The title product was obtained as a white solid in 72% yield (50 mg) from redox-active ester **22** (100 mg, 0.20 mmol, 1 equiv) and 4-bromo-3,5-dimethyl-1*H*-pyrazole (105 mg, 0.60 mmol, 3 equiv) using bismuth complex **1** as catalyst (9 mg, 0.020 mmol, 10 mol%) in DMA (0.033 M) following General Procedure B, after flash column chromatography in silica gel using hexanes/EtOAc (gradient from 95:5 to 8:2; R_f = 0.50 in hexanes/EtOAc 8:2).

^{1}**H NMR** (500 MHz, $CDCl_3$, 253 K, mixture of rotamers) δ 5.90 (dd, *J* = 7.5, 1.8 Hz, "0.5 H", *first rotamer*), 5.76 (dd, *J* = 7.0, 3.1 Hz, "0.5 H", *second rotamer*), 3.72 (m, 1H), 3.54 – 3.36 (m, 1H), 2.58 – 2.17 (m, 8H), 2.08 – 1.88 (m, 2H), 1.40 (s, 5H, *first rotamer*), 1.27 (s, 4H, *second rotamer*).

^{13}**C NMR** (126 MHz, $CDCl_3$, 253 K, mixture of rotamers, second signal of each pair in parenthesis) δ 154.5, (153.5), 146.6, (146.3), 137.7, (136.6), 94.0, (93.6), 80.5, (80.1), 69.8, (69.6), 47.1, (46.8), 34.1, (32.9), 28.4, (28.2), 23.7, (22.5), 12.9, (12.7), 10.6, (10.5).

FTIR (ATR): $\tilde{\nu}$ [cm^{-1}] = 2980, 2931, 2887, 1691, 1546, 1377, 1366, 1348, 1253, 1161, 1122, 1085, 1068, 884, 775, 744, 685, 549, 448.

HRMS (ESI Positive): calculated for $C_{14}H_{22}N_3O_2BrNa$ [M+Na]$^+$: 366.07877; found: 366.07876.

MP: 85–87 °C.

tert-Butyl 2-(3,5-bis(trifluoromethyl)-1-pyrazol-1-yl)pyrrolidine-1-carboxylate (43)

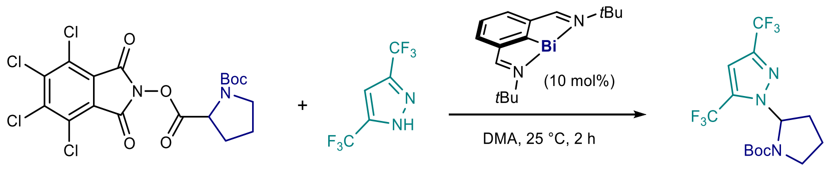

The title product was obtained as a colorless oil in 73% yield from redox-active ester **22** (100 mg, 0.20 mmol, 1 equiv) and 3,5-bis(trifluoromethyl)-1*H*-pyrazole (122 mg, 0.60 mmol, 3 equiv) using bismuth complex **1** as catalyst (9 mg, 0.020 mmol, 10 mol%) in DMA (0.033 M) following General Procedure B, after flash column chromatography in silica gel using hexanes/EtOAc +1% of NEt_3 (gradient from 98:2 to 9:1; R_f = 0.60 in hexanes/EtOAc 9:1).

^{1}H NMR (600 MHz, CD_2Cl_2, 253 K, mixture of rotamers) δ 6.93 – 6.89 (m, , "0.34 H", minor rotamer), 6.18 (d, *J* = 6.0 Hz, , "0.66 H", major rotamer), 6.09 – 6.06 (m, 1H), 3.81 – 3.72 (m, 1H), 3.48 – 3.41 (m, 1H), 2.41 – 2.13 (m, 2H), 2.02 – 1.91 (m, 2H), 1.38 (s, "2.7 H", minor rotamer), 1.18 (s, "5.3 H", major rotamer).

^{13}C NMR (151 MHz, CD_2Cl_2, 253 K, major rotamer) δ 152.9, 141.0 (q, *J* = 40 Hz), 131.9 (q, *J* = 41 Hz), 120.8 (q, *J* = 272 Hz), 118.9 (q, *J* = 272 Hz), 106.1 (q, *J* = 2.1 Hz), 80.8, 73.6 (q, *J* = 2.0 Hz), 47.0, 34.6, 27.7, 21.5.

^{19}F NMR (565 MHz, CD_2Cl_2, 253 K) δ -59.31 (major rotamer), -59.32 (minor rotamer), -62.55 (minor rotamer), -62.56 (major rotamer).

FTIR (ATR): $\tilde{\nu}$ [cm^{-1}] = 3155, 2983, 2894, 1711, 1563, 1388, 1368, 1264, 1202, 1159, 1128, 1090, 1045, 976, 898, 826, 699, 559, 440.

HRMS (ESI Positive): calculated for $C_{14}H_{17}N_3O_2F_6Na$ $[M+Na]^+$: 396.11171; found: 396.11197.

tert-Butyl 2-(2-phenyl-1*H*-imidazol-1-yl)pyrrolidine-1-carboxylate (44)

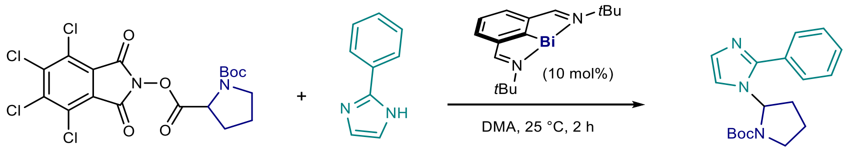

The title product was obtained as a colorless oil in 47% yield (22 mg) from redox-active ester **22** (75 mg, 0.15 mmol, 1 equiv) and 2-phenyl-1*H*-imidazole (71 mg, 0.45 mmol, 3 equiv) using bismuth complex **1** as catalyst (7 mg, 0.015 mmol, 10 mol%) in DMA (0.033 M) following General Procedure B, after flash column chromatography in silica gel using hexanes/EtOAc (gradient from 8:2 to 3:7; R_f = 0.25 in hexanes/EtOAc 3:7).

^{1}H NMR (500 MHz, CD_2Cl_2, 233 K, mixture of rotamers) δ 7.54 – 7.52 (m, 1H), 7.49 – 7.40 (m, 3H), 7.31 – 7.21 (m, 1H), 7.10 – 7.05 (m, 1H), 7.04 – 6.99 (m, 1H), 6.09 – 6.05 (m, "0.35 H", minor rotamer) 5.88 (dd, *J* = 6.9, 1.8 Hz, "0.65 H", major rotamer), 3.78 – 3.65 (m, 1H), 3.45 – 3.35 (m, 1H), 2.46 – 2.21 (m, 1H), 2.21 – 1.92 (m, 3H), 1.38 (s, "3H", minor rotamer), 1.11 (s, "6H", major rotamer).

^{13}C NMR (126 MHz, CD_2Cl_2, 233 K, mixture of rotamers) δ 153.9, 153.0, 146.9, 146.5, 130.5, 130.4, 129.0, 128.9, 128.69, 128.62, 128.5, 128.2, 128.0, 127.8, 117.0, 116.9, 80.3, 80.1, 69.3, 69.1, 47.0, 46.5, 35.4, 34.3, 27.8, 27.6, 22.8, 22.0.

FTIR (ATR): $\tilde{\nu}$ [cm^{-1}] = 3063, 2976, 2928, 2761, 1695, 1559, 1502, 1456, 1381, 1366, 1252, 1159, 1103, 947, 916, 869, 772, 746, 707, 693, 565, 461.

HRMS (ESI Positive): calculated for $C_{18}H_{24}N_3O_2$ $[M+H]^+$: 314.18630; found: 314.18618.

tert-Butyl 2-(2-methyl-1H-benzo[d]imidazol-1-yl)pyrrolidine-1-carboxylate (45)

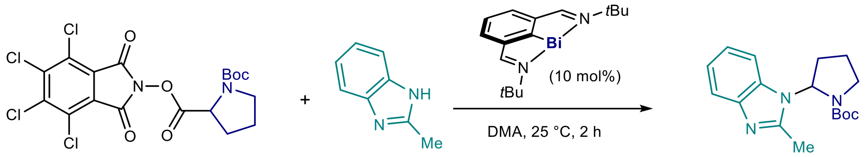

The title product was obtained as a colorless oil in 71% yield (32 mg) from redox-active ester **22** (75 mg, 0.15 mmol, 1 equiv) and 2-methylbenzimidazole (59 mg, 0.45 mmol, 3 equiv) using bismuth complex **1** as catalyst (7 mg, 0.015 mmol, 10 mol%) in DMA (0.033 M) following General Procedure B, after flash column chromatography in silica gel using hexanes/EtOAc (gradient from 7:3 to 2:8; R_f = 0.15 in hexanes/EtOAc 1:1).

^{1}H NMR (500 MHz, CD_2Cl_2, 233 K, mixture of rotamers) δ 7.63 – 7.55 (m, 1H), 7.26 – 7.21 (m, 1H), 7.18 – 7.13 (m, 2H), 6.11 – 6.08 (m, "0.28H", minor rot.), 5.97 – 5.92 (m, "0.72H", major rot.), 3.76 – 3.68 (m, 2H), 2.63 (s, "0.85H", minor rot.), 2.57 (s, "2.15H", major rot.), 2.46 – 2.34 (m, 1H), 2.31 – 2.17 (m, 1H), 2.16 – 2.06 (m, 1H), 2.02 – 1.92 (m, 1H), 1.33 (s, "2.4H", minor rot.), 0.92 (s, "6.6H", major rot.).

^{13}C NMR (126 MHz, CD_2Cl_2, 233 K, mixture of rotamers) δ 154.1, 153.2, 151.7, 151.5, 142.6, 132.5, 132.0, 121.7, 121.6, 121.3, 121.2, 118.78, 118.70, 110.3, 80.1, 79.9, 69.0, 68.5, 47.5, 46.9, 32.8, 31.9, 27.7, 27.3, 23.9, 23.1, 14.84, 14.80.

FTIR (ATR): $\tilde{\nu}$ [cm^{-1}] = 3058, 2975, 2930, 2881, 1694, 1523, 1453, 1381, 1364, 1283, 1158, 1121, 1099, 1014, 871, 767, 739, 661, 541, 430.

HRMS (ESI Positive): calculated for $C_{17}H_{23}N_3O_2$ [M+H]$^+$: 301.17848; found: 301.17849.

Dimethyl 1-(1-(tert-butoxycarbonyl)pyrrolidin-2-yl)-1H-imidazole-4,5-dicarboxylate (46)

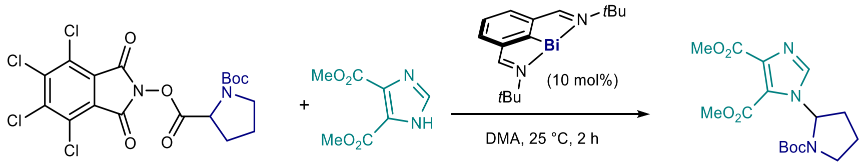

The title product was obtained as a white solid in 56% yield (30 mg) from redox-active ester **22** (75 mg, 0.15 mmol, 1 equiv) and dimethyl 1H-imidazole-4,5-dicarboxylate (83 mg, 0.45 mmol, 3 equiv) using bismuth complex **1** as catalyst (7 mg, 0.015 mmol, 10 mol%) in DMA (0.033 M) following General Procedure B, after flash column chromatography in silica gel using hexanes/EtOAc (gradient from 7:3 to 2:8; R_f = 0.25 in hexanes/EtOAc 3:7).

^{1}H NMR (500 MHz, CD_2Cl_2, 233 K, mixture of rotamers) δ 7.57 (s, "0.56H". major rot.), 7.55 (s, "0.44H", minor rot.) 6.31 (dd, *J* = 6.6, 1.2 Hz, "0.45H", minor rot.), 6.21 (dd, *J* = 6.7, 1.2 Hz, "0.54H", major rot.), 3.86 – 3.81 (m, 6H), 3.67 – 3.59 (m, 1H), 3.45 – 3.35 (m, 1H), 2.38 – 2.23 (m, 1H), 2.01 – 1.87 (m, 3H), 1.39 (s, "4.1H", minor rot.), 1.22 (s, "4.9H", major rot.).

^{13}C NMR (126 MHz, CD_2Cl_2, 233 K, mixture of rotamers) δ 163.2, 163.0, 160.5, 160.2, 153.6, 152.8, 137.6, 136.8, 136.5, 123.4, 122.9, 80.8, 80.7, 71.5, 71.2, 52.7, 52.3, 47.1, 46.5, 34.7, 34.0, 27.7, 27.5, 21.9, 21.3.

HRMS (ESI Positive): calculated for $C_{16}H_{23}N_3O_6Na$ [M+Na]$^+$: 376.14790; found: 376.14788.

FTIR (ATR): $\tilde{\nu}$ [cm^{-1}] = 2958, 2921, 2805, 2625, 2592, 2436, 1715, 1710, 1556, 1490, 1460, 1431, 1343, 1302, 1286, 1199, 1156, 1063, 970, 950, 865, 820, 796, 764, 664, 636, 588, 573, 481.

MP: 169–178 °C.

tert-Butyl 2-(1,3-dimethyl-2,6-dioxo-1,2,3,6-tetrahydro-7_H_-purin-7-yl)pyrrolidine-1-carboxylate (47)

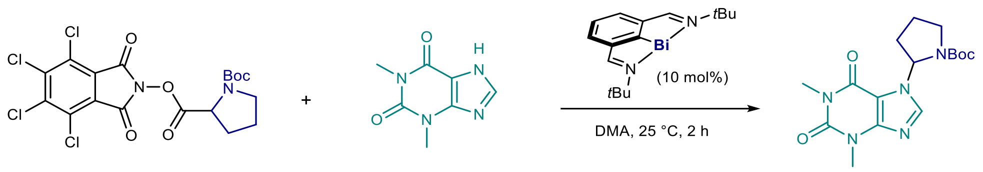

The title product was obtained as a white solid in 57% yield (40 mg, >20:1 rr) from redox-active ester **22** (100 mg, 0.20 mmol, 1 equiv) and theophylline (108 mg, 0.60 mmol, 3 equiv) using bismuth complex **1** as catalyst (9 mg, 0.020 mmol, 10 mol%) in DMA (0.033 M) following General Procedure B, after flash column chromatography in silica gel using a gradient from hexanes/EtOAc 1:1 + 1% of NEt_3 to pure EtOAc +1% of NEt_3 (product R_f = 0.40 in pure EtOAc; starting theophylline R_f = 0.1 in pure EtOAc).

Full assignment by NMR of both rotamers of the product was conducted.

^{1}H NMR (600 MHz, CD_2Cl_2, 253 K, ca. 1:1 mixture of *cis* –A– and *trans* –B– rotamers) δ 7.57 (d, *J* = 0.6 Hz, "0.5H", rotamer A), 7.55 (d, *J* = 0.6 Hz, "0.5H", rotamer B), 6.43 (dd, *J* = 6.5, 1.2 Hz, "0.5H", rotamer B), 6.40 (dd, *J* = 6.5, 1.4 Hz, "0.5H", rotamer A), 3.72 – 3.65 (m, 1H, A+B), 3.52 (s, "1.5H", rotamer A), 3.50 (s, 1.5H", rotamer B), 3.42 (m, 1H, A+B), 3.32 (s, "1.5H", rotamer A), 3.31 (s, "1.5H", rotamer B), 2.40 – 2.26 (m, 1H, A+B), 2.15 – 2.05 (m, 1H, A+B), 2.02 – 1.88 (m, 1H, A+B), 1.93 – 1.81 (m, 1H, A+B), 1.42 (s, "4.5H", rotamer B), 1.26 (s, "4.5H", rotamer A).

^{13}C NMR (151 MHz, CD_2Cl_2, 253 K, ca. 1:1 mixture of rotamers; rotamer B –*trans*– signals in parenthesis) δ (155.03), 155.02, (153.9), 153.3, 151.57, (151.56), (149.4), 149.3, 138.4, (138.2), (106.2), 106.1, (80.98), 80.91, (71.2), 71.0, (47.3), 46.8, 34.7, (33.9), 29.75, (29.71), (28.0), 27.91, (27.91), 27.8, (22.2), 21.4.

FTIR (ATR): $\tilde{\nu}$ [cm^{-1}] = 3475, 3107, 2975, 2891, 1697, 1659, 1601, 1541, 1414, 1383, 1366, 1207, 1158, 1121, 1018, 874, 746, 642, 504, 414.

HRMS (ESI Positive): calculated for $C_{16}H_{23}N_5O_4Na$ $[M+Na]^+$: 372.16422; found: 372.16441.

MP: 99–106 °C.

Key NOE correlations for the assignment of the *cis* rotamer of the product.

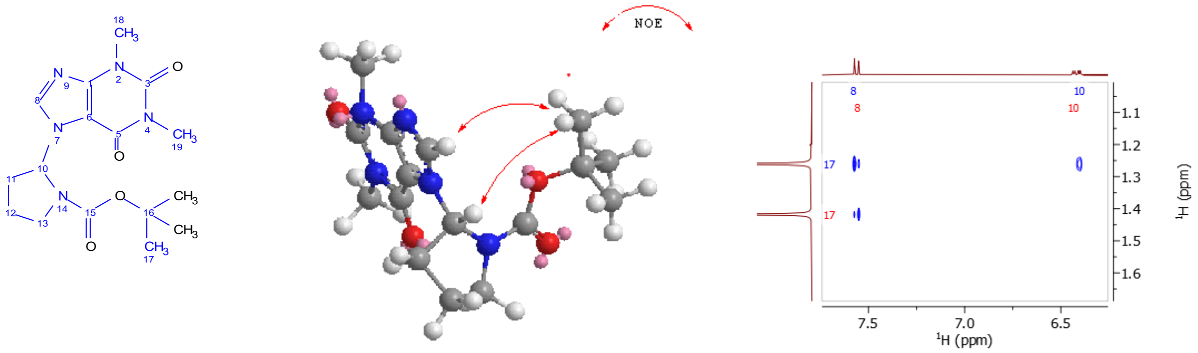

tert-Butyl 2-(2-(thiazol-4-yl)-1*H*-benzo[*d*]imidazol-1-yl)pyrrolidine-1-carboxylate (48)

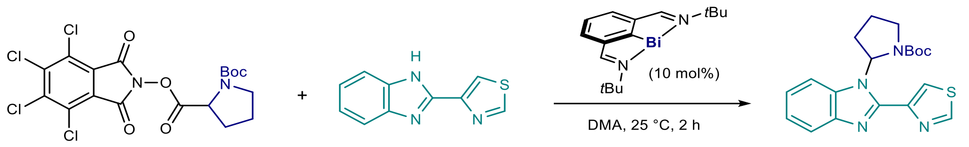

The title product was obtained as an amorphous white solid in 61% yield (45 mg) from redox-active ester **22** (100 mg, 0.20 mmol, 1 equiv) and thiabendazole (121 mg, 0.60 mmol, 3 equiv) using bismuth complex **1** as catalyst (9 mg, 0.020 mmol, 10 mol%) in DMA (0.033 M) following General Procedure B, after preparative TLC in silica gel with 25:75 hexane/EtOAc (R_f = 0.40 in hexanes/EtOAc 3:7).

^{1}H NMR (600 MHz, $CDCl_3$, mixture of rotamers, most signals coalesced) δ 8.92 – 8.86 (m, 1H), 8.30 (d, *J* = 19.3 Hz, 1H), 7.81 – 7.76 (m, 1H), 7.38 – 7.34 (m, 1H), 7.29 – 7.27 (m, 1H), 7.25 – 7.22 (m, 1H), 7.12 – 7.06 (m, 1H), 3.89 – 3.78 (m, 2H), 2.78 – 2.71 (m, 1H), 2.50 – 2.42 (m, 1H), 2.23 – 2.17 (m, 1H), 2.08 – 2.03 (m, 1H), 0.86 (s, 9H).

^{13}C NMR (151 MHz, $CDCl_3$, mixture of rotamers, most signals coalesced) δ 153.8, 153.6, 152.7, 148.2, 147.3, 143.2, 132.9, 123.3, 122.6, 121.6, 120.0, 111.6, 80.3, 70.6, 47.6, 34.2, 27.7, 23.3.

FTIR (ATR): $\tilde{\nu}$ [cm^{-1}] = 3080, 2980, 2909, 1701, 1614, 1479, 1427, 1364, 1322, 1248, 1223, 1162, 969, 925, 877, 774, 737, 569, 466.

HRMS (ESI Positive): calculated for $C_{16}H_{23}N_5O_4Na$ $[M+Na]^+$: 372.16422; found: 372.16441.

tert-Butyl 2-(2-oxo-6-(trifluoromethoxy)benzo[*d*]thiazol-3(2*H*)-yl)pyrrolidine-1-carboxylate (49)

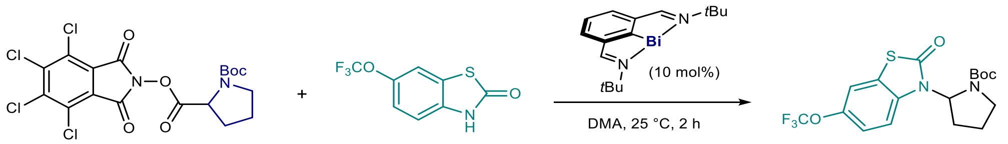

The title product was obtained as an amorphous solid in 74% yield (59 mg, with 10% of inseparable starting nucleophile) from redox-active ester **22** (100 mg, 0.20 mmol, 1 equiv) and 6-(trifluoromethoxy)benzo[*d*]thiazol-2(3*H*)-one (110 mg, 0.60 mmol, 3 equiv) using bismuth complex **1** as catalyst (9 mg, 0.020 mmol, 10 mol%) in DMA (0.033 M) following General Procedure B, after preparative TLC in silica gel with 7:3 hexane/EtOAc (three elutions, R_f = 0.40 in hexanes/EtOAc 7:3).

^{1}H NMR (600 MHz, $CDCl_3$, 263 K, major rotamer of the ca. 3:1 mixture) δ 7.34 (d, *J* = 2.1 Hz, 1H), 7.14 (dd, *J* = 8.8, 1.8 Hz, 1H), 7.04 (d, *J* = 8.9 Hz, 1H), 6.19 (t, *J* = 7.4 Hz, 1H), 3.82 (ddd, *J* = 10.9, 8.5, 2.6 Hz, 1H), 3.65 (m, 1H), 2.43 (dtt, *J* = 13.2, 8.9, 4.5 Hz, 1H), 2.32 – 2.23 (m, 1H), 2.16 (dtt, *J* = 13.0, 6.5, 2.7 Hz, 1H), 2.04 – 1.96 (m, 1H), 1.15 (s, 9H).

^{13}C NMR (151 MHz, $CDCl_3$, 263 K, ca. 3:1 mixture of rotamers, minor rotamer in parenthesis) δ 169.2, (169.1), (154.0), 153.6, 144.4, 144.38, 144.37, 144.36, (144.29), (144.27), (135.1), 133.8, (123.9), 123.5, 122.9, 121.2, 119.8, (119.7), 119.5, 117.8, 116.2, (116.1), 112.1, (111.6), 81.0, (80.7), (69.3), 68.4, (47.6), 47.1, 30.6, (30.5), (28.3), 28.0, (24.2), 23.2.

FTIR (ATR): $\tilde{\nu}$ [cm^{-1}] = 3156, 3082, 2972, 2902, 1698, 1456, 1389, 1282, 1163, 1127, 1098, 1007, 775, 738, 640, 535, 466, 421.

HRMS (ESI Positive): calculated for $C_{17}H_{19}F_3N_2NaO_4S$ $[M+Na]^+$: 427.09098; found: 427.09125.

tert-Butyl 2-(4,5,6,7-tetrachloro-1,3-dioxoisoindolin-2-yl)pyrrolidine-1-carboxylate (**24**)

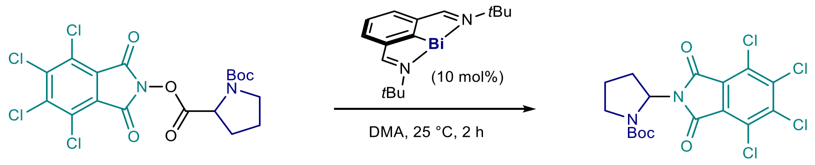

The title product was obtained as a white solid in 85% yield from redox-active ester **22** (100 mg, 0.20 mmol, 1 equiv), without external nucleophile, using bismuth complex **1** as catalyst (9 mg, 0.020 mmol, 10 mol%) in DMA (0.033 M) following General Procedure B, after flash column chromatography in silica gel using a hexanes/EtOAc gradient from 95:5 to 8:2; (R_f = 0.5 in hexanes/EtOAc 9:1).

^{1}H NMR (600 MHz, 253 K, acetone-d_6, mixture of rotamers): δ 5.95 (ddd, *J* = 7.8, 3.7, 1.7 Hz, 1H), 3.66 – 3.49 (m, 2H), 2.49 – 2.35 (m, 1H), 2.32 – 2.16 (m, 2H), 1.98 – 1.88 (m, 1H), 1.36 (s, 4H), 1.24 (s, 5H).

^{13}C NMR (151 MHz, 253 K, acetone-d_6, major rotamer): δ 163.3, 153.2, 139.8, 129.6, 128.7, 80.1, 64.9, 47.4, 31.8, 28.2, 23.8.

HRMS (ESI Positive): calculated for $C_{17}H_{16}N_2O_4Cl_4Na$ $[M+Na]^+$: 474.97563; found: 474.97585.

Characterization data fully matched the ones previously reported for this compound.[5]

4,5,6,7-Tetrachloro-2-(2-(4-chlorophenoxy)propan-2-yl)isoindoline-1,3-dione (**50**)

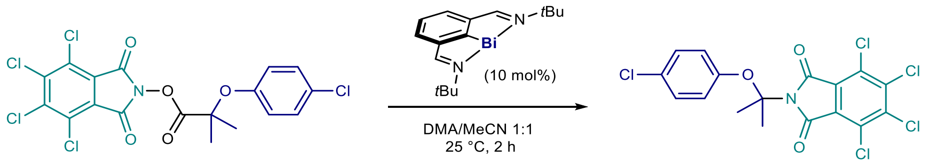

The title product was obtained as a white solid in 75% yield (71 mg) from redox-active ester **S14** (100 mg, 0.20 mmol, 1 equiv), without external nucleophile, using bismuth complex **1** as catalyst (9 mg, 0.020 mmol, 10 mol%) in DMA/MeCN (1:1, 0.033 M) following General Procedure B, after flash column chromatography in silica gel using pentane/dichloromethane (1:1) as eluent or by preparative TLC in hexane/EtOAc 95:5 (R_f = 0.50 in hexanes/EtOAc 95:5).

^{1}H NMR (600 MHz, $CDCl_3$): δ 7.19 (d, *J* = 8.9 Hz, 2H), 6.88 (d, *J* = 8.9 Hz, 2H), 2.05 (s, 6H).

^{13}C NMR (151 MHz, $CDCl_3$): δ 163.5, 152.6, 140.8, 130.0, 129.7, 129.6, 126.9, 123.2, 92.6, 27.6.

HRMS (ESI Positive): calculated for $C_{17}H_{10}NO_3Cl_5Na$ $[M+Na]^+$: 474.97563; found: 474.97585.

MP: 105–108 °C.

Characterization data fully matched the ones previously reported for this compound.[5]

4,5,6,7-Tetrachloro-2-(2-(4-(4-chlorobenzoyl)phenoxy)propan-2-yl)isoindoline-1,3-dione (**51**)

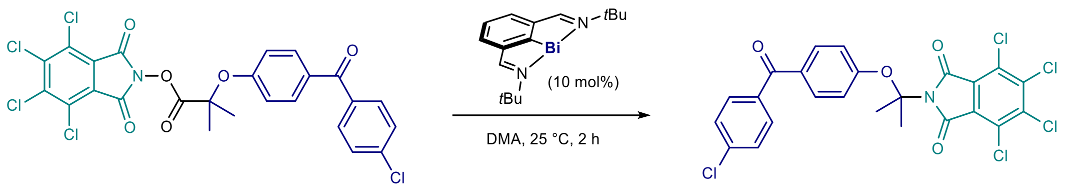

The title product was obtained as a white solid in 73% yield (62 mg) from redox-active ester **S15** (90 mg, 0.15 mmol, 1 equiv), without external nucleophile, using bismuth complex **1** as catalyst (7 mg, 0.015 mmol, 10 mol%) in DMA (0.033 M) following General Procedure B, after flash column chromatography in silica gel using hexanes/EtOAc (gradient from 95:5 to 8:2; R_f = 0.60 in hexanes/EtOAc 9:1, very close to the starting RAE). Slow gas-diffusion of pentane into a solution of the title compound in 1,2-DCE over 3 days in the fridge (5 °C) gave crystalline needles suitable for single-crystal x-ray diffraction analysis.

^{1}H NMR (400 MHz, $CDCl_3$) δ 7.72 – 7.65 (m, 4H), 7.45 – 7.41 (m, 2H), 7.05 – 6.99 (m, 2H), 2.12 (s, 6H).

^{13}C NMR (101 MHz, $CDCl_3$) δ 194.2, 163.1, 157.9, 140.7, 138.6, 136.0, 132.1, 132.0, 131.2, 129.9, 128.6, 126.7, 119.6, 92.2, 27.6.

FTIR (ATR): $\tilde{\nu}$ [cm^{-1}] = 2955, 1786, 1719, 1650, 1595, 1502, 1371, 1311, 1237, 1198, 1158, 1146, 1086, 1014, 927, 851, 833, 761, 734, 652, 592, 494, 472.

HRMS (ESI Positive): calculated for $C_{24}H_{14}NO_4ClNa$ $[M+Na]^+$: 577.92577; found: 577.92592.

MP: 159–164 °C.

6.3. Variable temperature NMR of representative products

40, heating in DMSO-d_6 (coalescence of rotamers)

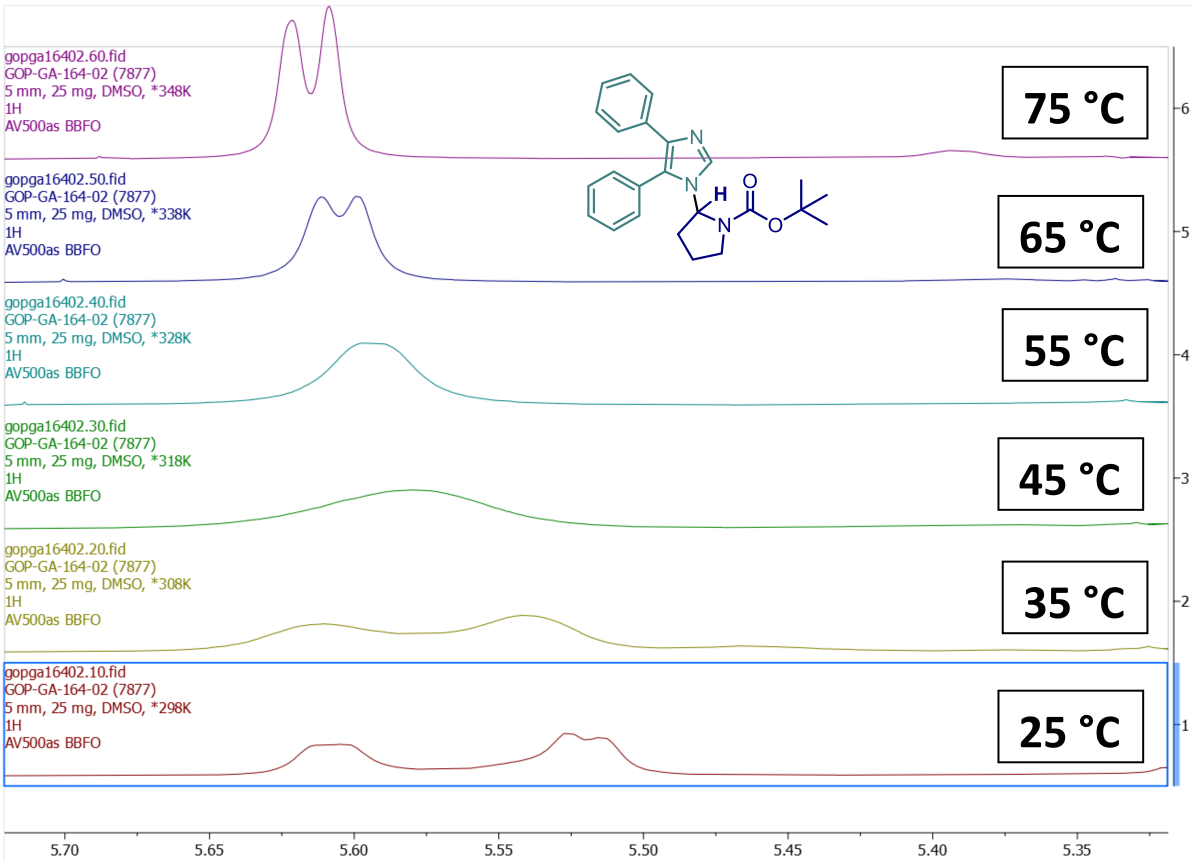

40, cooling down in $CDCl_3$ (better definition of rotamer peaks)

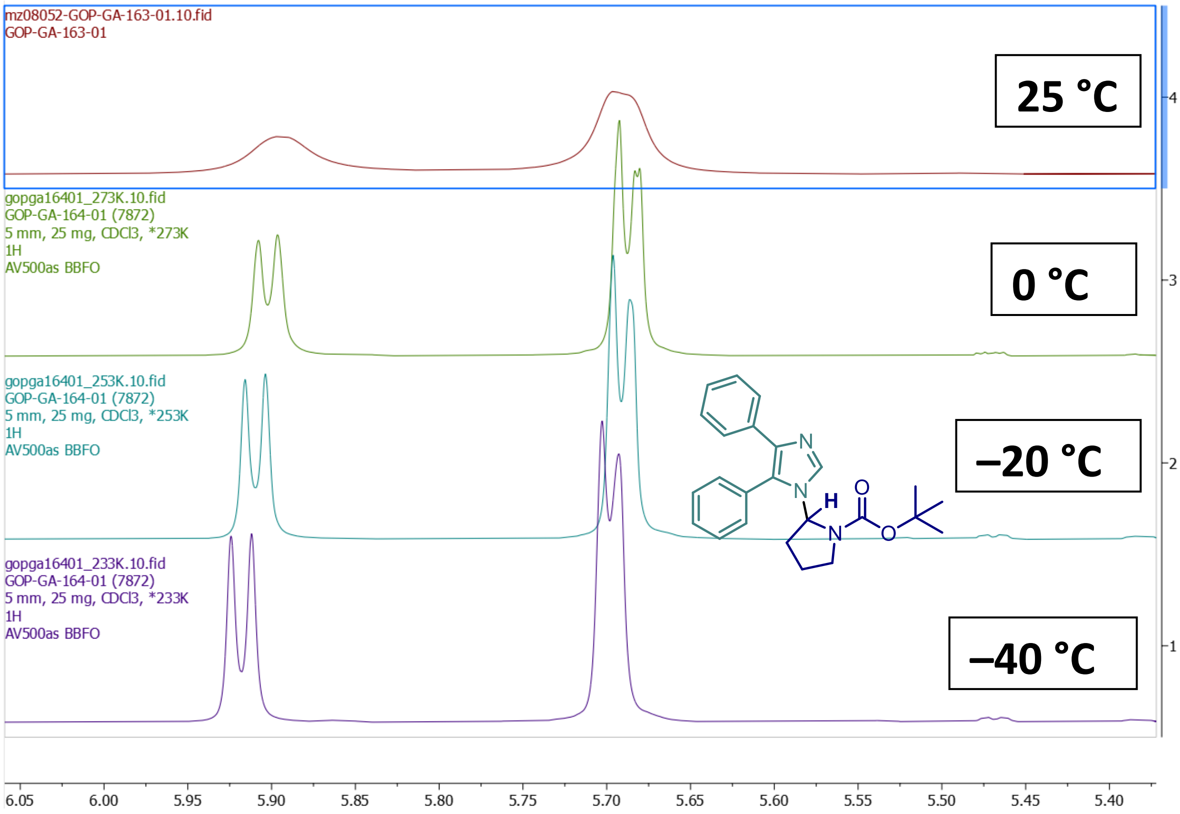

44, heating up in DMSO-d_6 (coalescence of rotamers)

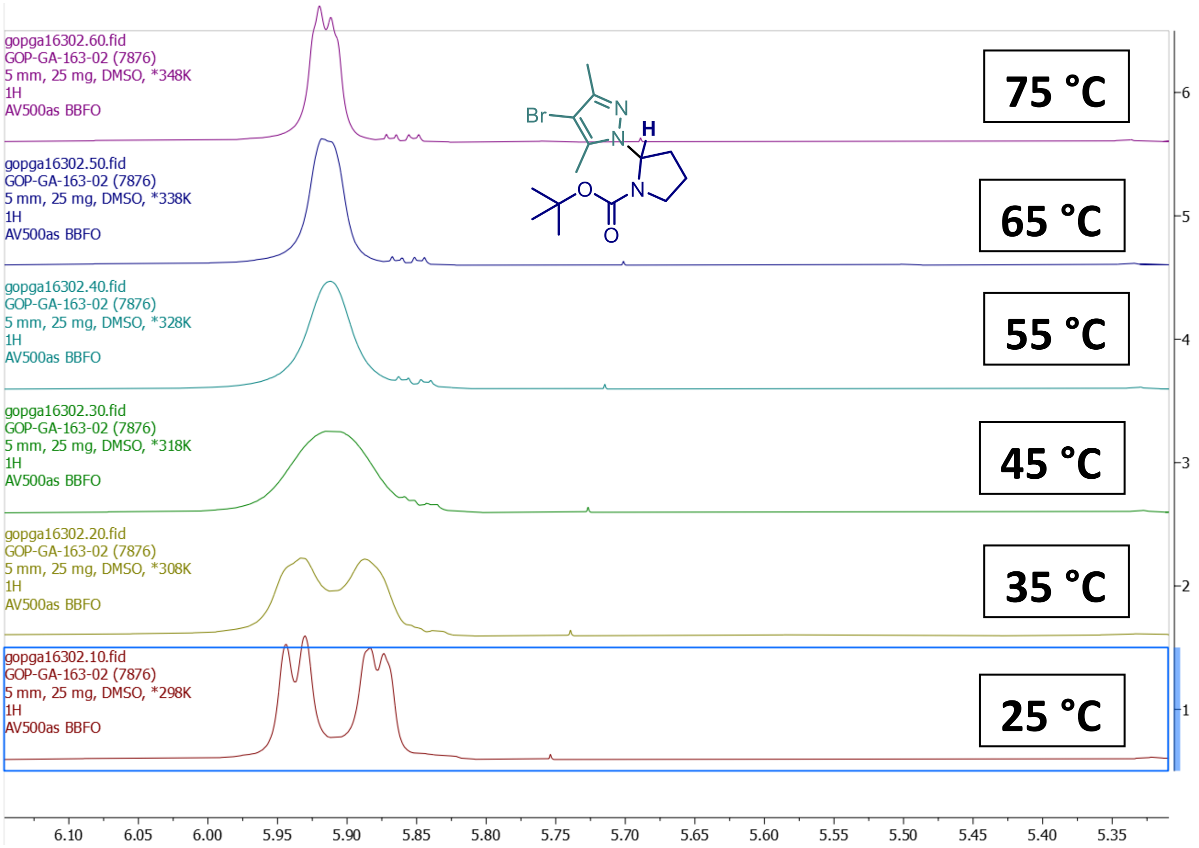

44, cooling down in $CDCl_3$ (better definition of rotamer peaks)

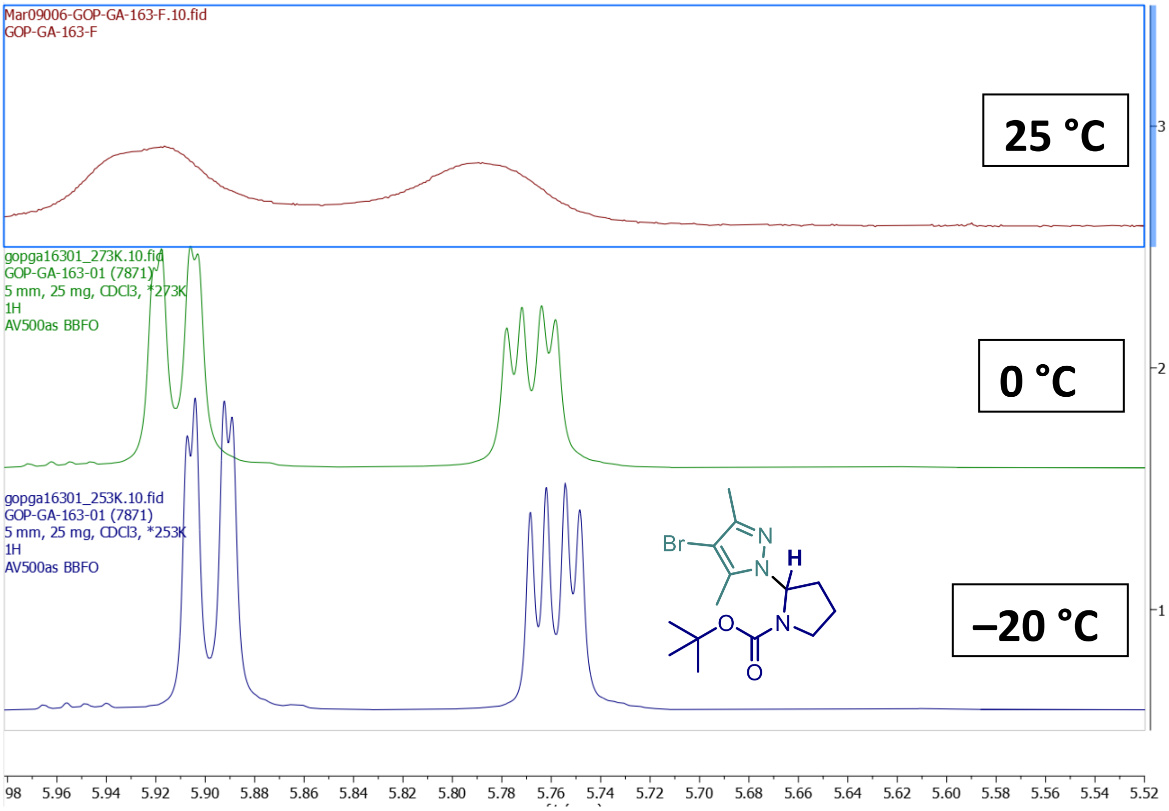

7. Mechanistic experiments for the C–N coupling reaction

Following the reaction by NMR in THF-d_8 (which only takes place above 0 °C) resulted only in observing the starting material and the product of the reaction, and no other species could be initially detected (see Section 5 of the SI).

Detection of α-amino alkylbismuth(III) intermediate 23

Switching to DMF-d_7 as solvent, we could observe and characterize the corresponding α-amino alkyl-bismuth(III) intermediate **23**, monitoring the reaction at –40 °C both in stoichiometric and catalytic conditions. Furthermore, HRMS could also be carried out after diluting a sample of **23** prepared in DMF with THF pre-cooled to –80 °C.

Stoichiometric experiment and characterization

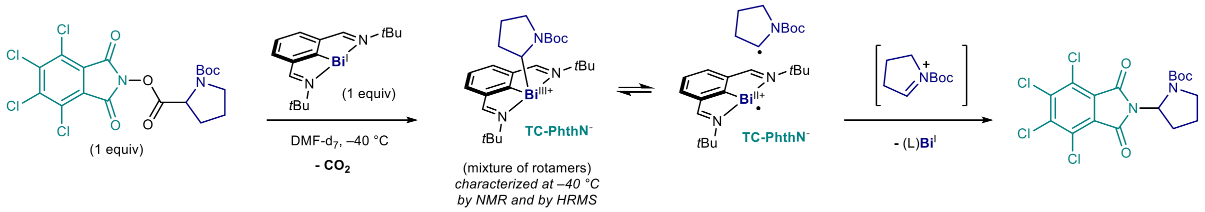

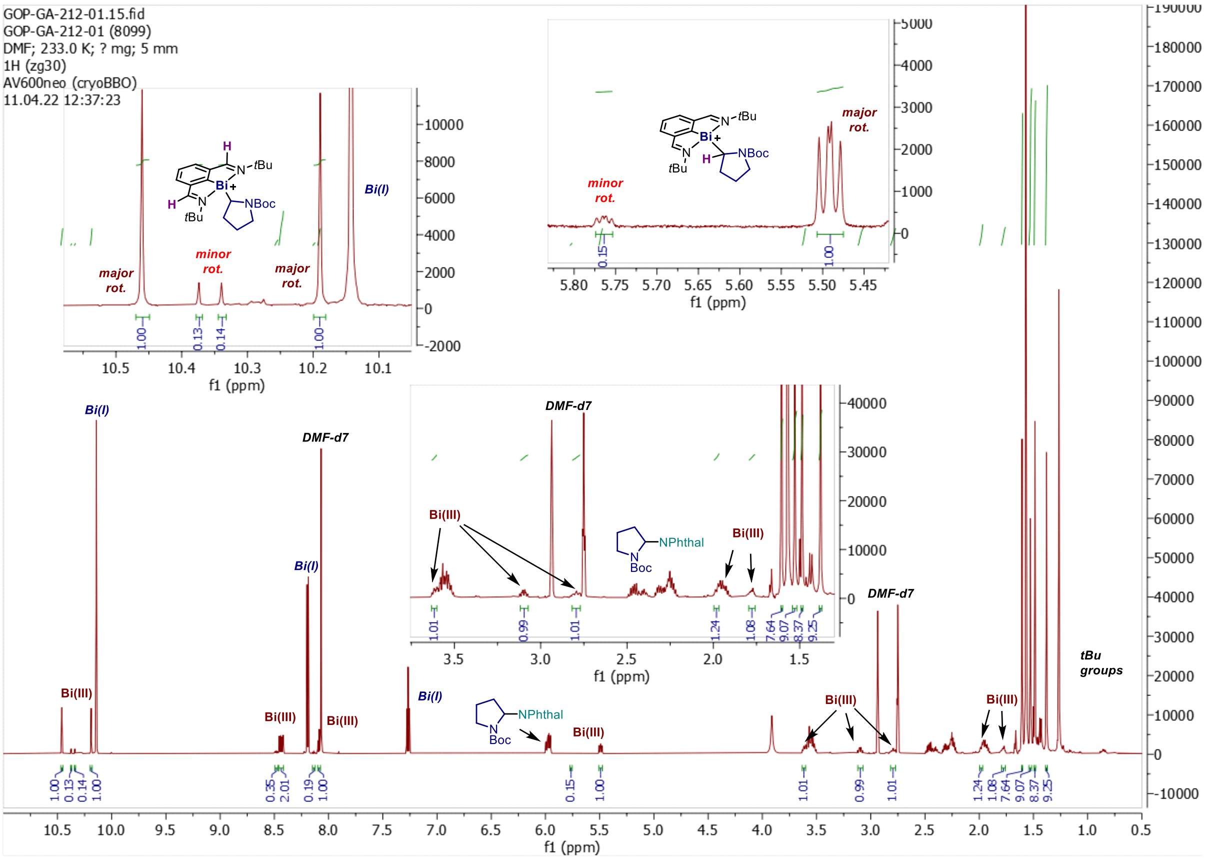

From the stoichiometric mixture of 1 equiv of bismuth(I) **1** and 1 equiv of BocPro-RAE **22** at –40 °C in DMF-*d*$_7$, a mixture of two new Bi-species was characterized, coexisting with some initial Bi(I) (already regenerated upon reaction product formation). The two Bi(III) species are likely rotamers, but the exchange between the two species could not be observed at low temperature from 2D NMR ROESY data. The ^{1}H and ^{13}C chemical shifts around the Bi-ligand part of the molecule are very similar for both Bi(III) species.

Assignments overview

[(2,6-(*t*BuNCH)$_2$C$_6$H$_3$)Bi(*N*-Boc-pirrolid-2-yl)(tetrachlorophthalimide)] (**23**)

Major rotamer (The signals of the major Bi(III) species could be fully assigned based on 1D and 2D NMR methods)

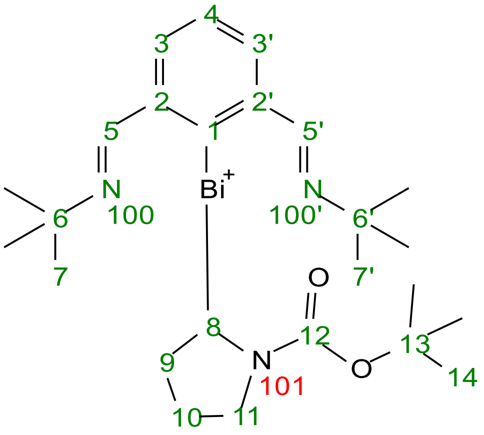

Atom	J	δ (ppm)	HSQC	COSY	HMBC	ROESY
1 C		190.862			3, 3', 5, 5'	
2 C		149.359			4, 5	
2' C		149.205			4, 5'	
3 C		137.088	3		3', 5	
H	7.60(4), 0.90(3')	8.450	3	4	1, 3', 5	4, 5
3' C		136.791	3'		3, 5'	
H	0.90(3), 7.60(4)	8.427	3'	4	1, 3, 5'	4, 5'
4 C			4			
H	7.60(3), 7.60(3')	8.090	4	3, 3'	2, 2'	3, 3'
5 C		170.696	5		3	
H		10.460	5		1, 2, 3, 6	3, 7
5' C		169.290	5'		3'	
H		10.188	5'		1, 2', 3', 6'	3', 7'
6 C		61.084			5, 7	
6' C		61.839			5', 7'	
7 C		30.676	7		7	
H3		1.485	7		6, 7	5
7' C		30.676	7'		7'	
H3		1.604	7'		6', 7'	5', 8

Atom	J	δ (ppm)	HSQC	COSY	HMBC	ROESY
8 C		91.172	8			
H	9.00(), 6.40()	5.493	8	9', 9"	9	7', 9', 9", 11"
9 C		30.676	9', 9"		8	
H'		2.795	9	8, 9", 10'		8
H"		1.977	9	8, 9'		8
10 C		29.901	10', 10"			
H'		1.782	10	9', 11'		
H"		1.517	10			
11 C		48.203	11', 11"			
H'		3.099	11	10', 11"		
H"		3.567	11	11'		8
12 C		155.285				
13 C		80.714			14	
14 C		28.207	14		14	
H3		1.527	14		13, 14	
100 N		-72.265				
100' N		-69.329				
101 N						

Minor rotamer (the assignment of the minor species could only be based on ^{1}H and 2D NMR data as its amount was too low and decreasing over time)

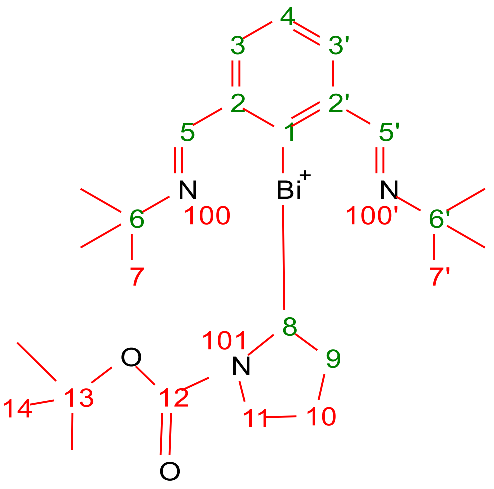

Atom	J	δ (ppm)	HSQC	HMBC
1 C		190.740		
2 C		149.148		
2' C		148.589		
3 C		137.241		
H	7.60(4), 0.90(3')	8.466		
3' C		137.667	3'	
H	0.90(3), 7.60(4)	8.485	3'	
4 C		130.456		
H	7.60(3), 7.60(3')	8.141		
5 C		170.995	5	
H		10.374	5	6
5' C		169.826		
H		10.340		
6 C		61.131		5
6' C		61.715		
7 C				
H3				
7' C				
H3				

Atom	J	δ (ppm)	HSQC	HMBC
8 C		94.984	8	
H	6.80(?), 4.30(?)	5.766	8	
9 C				
H'		2.795		
H''				
10 C				
H2				
11 C				
H2				
12 C				
13 C				
14 C				
H3				
100 N				
100' N				
101 N				

For ESI-HRMS measurement, a solution of the same intermediate was prepared in the same way in DMF at –40 °C under argon. Then, this solution was diluted with dry THF, precooled at –78 °C, and the resulting solution was immediately submitted to MS analysis.

HRMS (ESI Positive): calculated for $C_{25}H_{39}BiN_3O_2$ [M-TCPhth]$^{+}$: 622.28408; found: 622.28332.

For full NMR data and key correlations for the assignment, see NMR-spectra section.

Kinetic profile of the catalytic reaction at –40 °C monitored by ^{1}H NMR

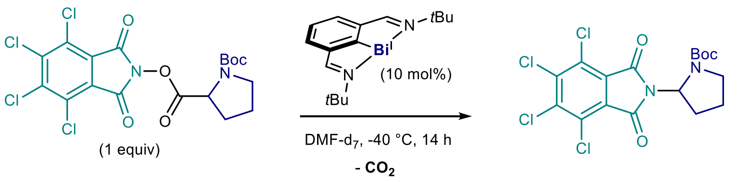

The reaction was followed by ^{1}H NMR in DMF-d_7. After ca. 5 h at –40 °C, full conversion of the starting redox-active ester **22** (blue) was observed, leading mostly to the reaction product of decarboxylative amination **24** (green). However, some of the corresponding α-amino alkyl-radical intermediate was trapped reductively by the Bi(II) intermediate, giving some accumulation of α-amino alkylbismuth(III) intermediate **23** (red).

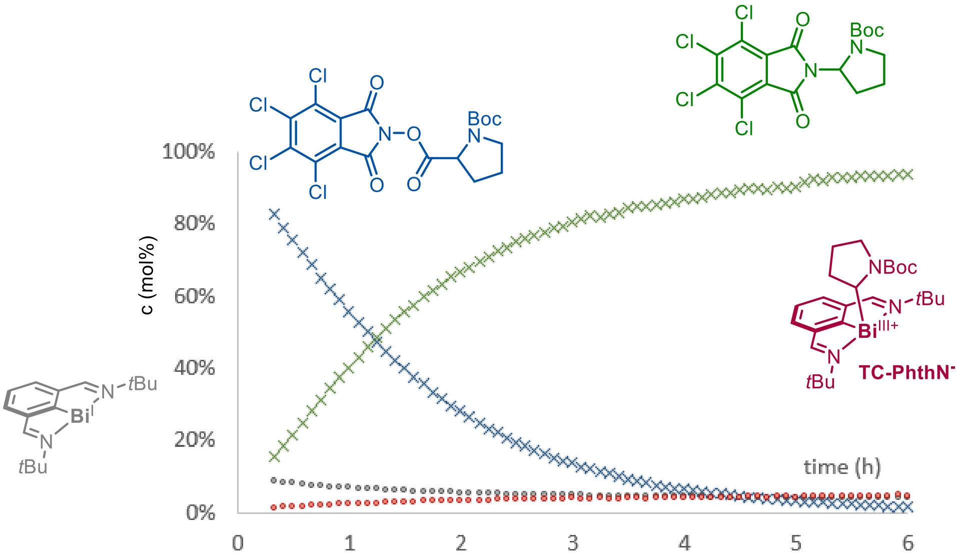

The first spectrum was acquired 20 min after mixing the components at –40 °C.

Further following this reaction at –40 °C allowed to observe that, eventually, the accumulated α-amino alkylbismuth(III) intermediate **23** can also transform back to bismuth(I) upon releasing iminium cation (that can then be trapped by phthalimide).

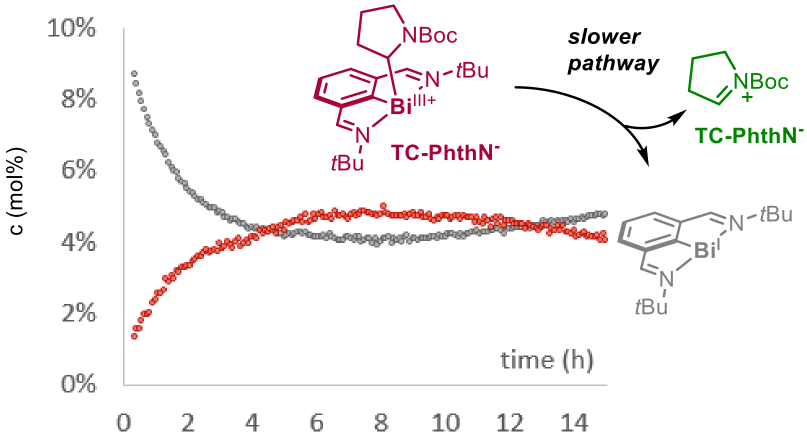

The first spectrum was acquired 20 min after mixing the components at –40 °C.

Kinetic profile of the catalytic reaction at -20 °C monitored by ^{1}H NMR

To fully confirm this hypothesis, the same reaction was followed at –20 °C. Here, we can see full conversion of the starting material **22** (red) in less than 2 h. As expected, most of it goes directly to the reaction product **24** (green) and some of the α-amino alkyl radical gets trapped as the alkyl bismuth(III) adduct **23** (blue), which gets accumulated over the first hour, and then starts decaying back to bismuth(I) **1** (turquoise) upon slow release of the remaining ca. 5% of the reaction product (slow increase of the amount of **24**, between 1 h and 3 h –green, left graph).

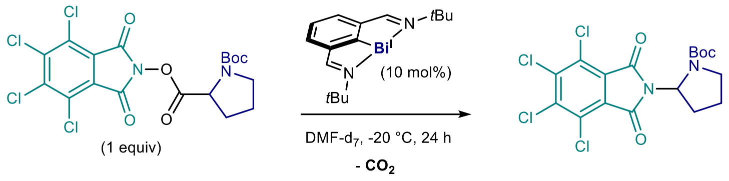

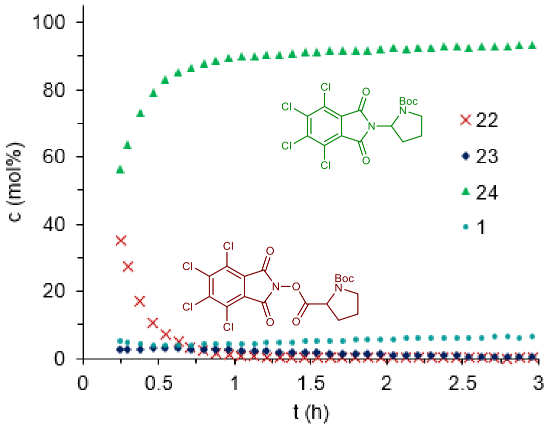

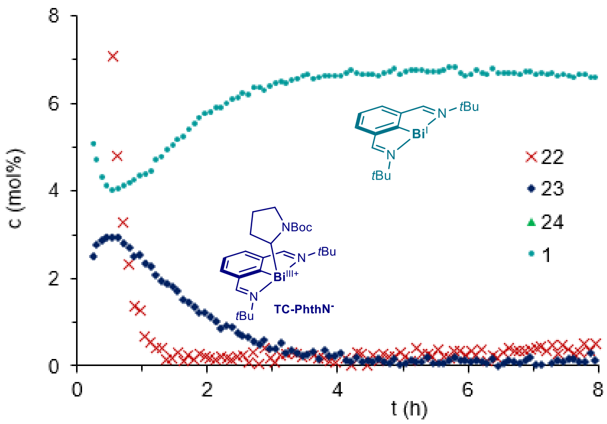

Left: full profile over 3 h. **Right**: Zoom into the detail of the transformations between Bi(I) and Bi(III) over 8 h.

The first spectrum was acquired 10 min after warming up the mixture to –20 °C in the NMR instrument (as expected, significant conversion already observed).

22 = starting redox-active ester; **23** = α-amino alkylbismuth(III) intermediate; **24** = reaction product; **1** = bismuth(I) complex.

Full mechanistic proposal with two alternative pathways

Therefore, since the consumption of the accumulated α-amino alkylbismuth(III) intermediate **23** is slow at –20 °C (and even slower at –40 °C), and when it starts being observed significantly, most starting material **22** has already been transformed into the reaction product **24**, we consider that this is a secondary, slower pathway.

When the α-amino alkyl-radical is generated upon SET from Bi(I), a highly reactive Bi(II) is formed. This can form α-amino alkylbismuth(III) intermediate **23** through radical recombination (which can also slowly convert back into Bi(I) **1** and the iminium cation that leads to the reaction product **24**). Alternatively, Bi(II) can immediately oxidize the α-amino alkyl radical intermediate, going back to Bi(I) through release of the iminium that leads to the reaction product. According to kinetics, this second "in-cage" SET is faster than the alternative Bi(I/III) pathway, that can also be observed.

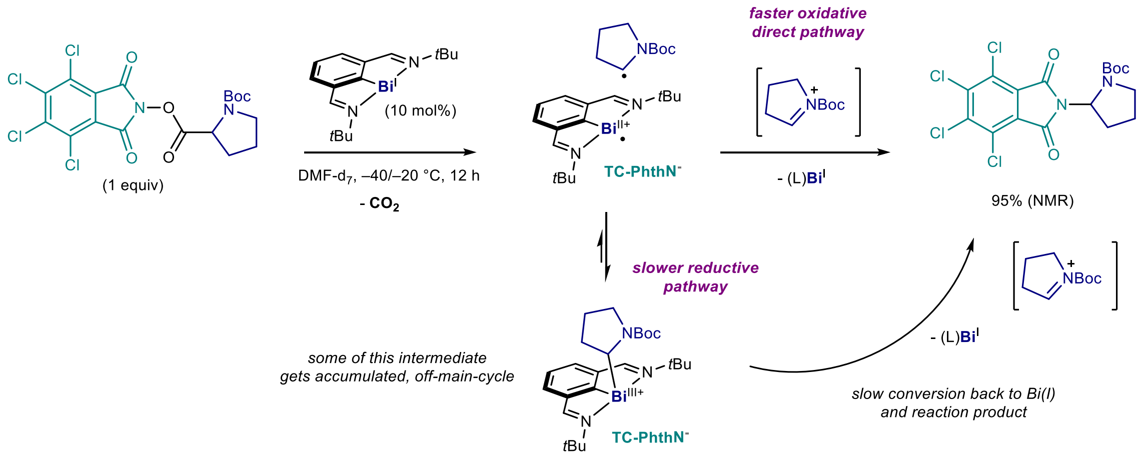

8. Crystal data and structure refinement

[(2,6-(*t*BuNCH)$_2$C$_6$H$_3$)Bi(benzyl)(tetrachlorophthalimide)] (**9**)

Single crystals of **9** were obtained by layering pentane on top of a solution of the compound in THF in an NMR tube, which was left over 4 days in the freezer (–30 °C). CCDC deposit number: 2178464.

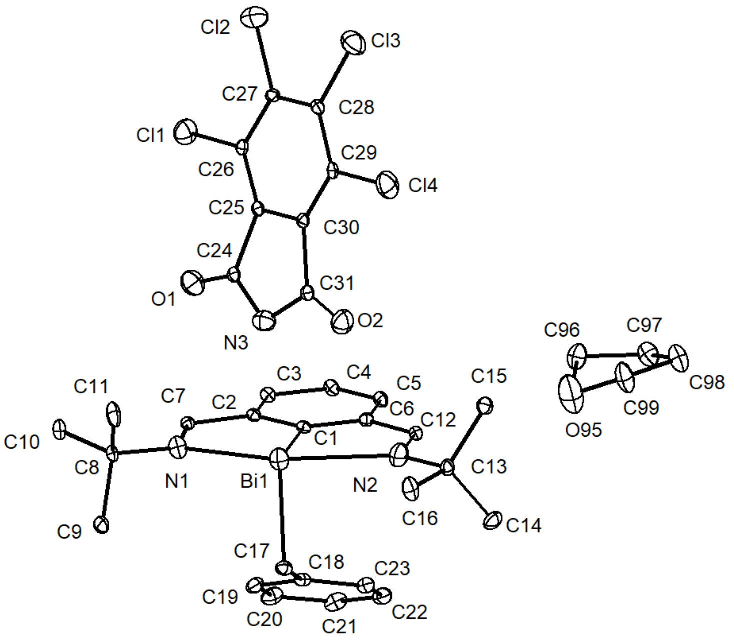

Molecular structure of **complex 9 · d$_8$-THF**. H and D atoms have been removed for clarity.

X-ray Crystal Structure Analysis of complex complex 9 · d$_8$-THF:

C_{35} H_{30} Bi Cl_4 D_8 N_3 O_3, M_r = 907.51 g mol^{-1}, yellow prism, crystal size 0.15 x 0.06 x 0.05 mm^3, triclinic, space group *P*-1 [2], *a* = 8.3615(11) Å, *b* = 13.0712(12) Å, *c* = 16.774(2) Å, *V* = 1771.9(4) Å^3, *T* = 100(2) K, *Z* = 2, D_{calc} = 1.701 g·cm^3, λ = 0.71073 Å, μ(*Mo-K*$_\alpha$) = 5.316 mm^{-1}, Gaussian absorption correction (T_{min} = 0.57672, T_{max} = 0.78122), Bruker AXS Enraf-Nonius KappaCCD diffractometer with a FR591 rotating Mo-anode X-ray source, 2.625 < θ < 29.130°, 49618 measured reflections, 9537 independent reflections, 8530 reflections with $I > 2\sigma(I)$, R_{int} = 0. 0554. The structure was solved by *SHELXT* and refined by full-matrix least-squares (*SHELXL*) against F^2 to R_1 = 0.0266 [$I > 2\sigma(I)$], wR_2 = 0.0557 [all data], 421 parameters and 0 restraints.

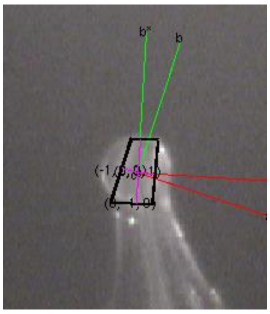

	h	k	l	distance (mm)
1.	0	0	1	0.025
2.	0	0	-1	0.025
3.	-1	0	0	0.03
4.	0	-1	0	0.055
5.	0	1	0	0.06
6.	3	1	0	0.03

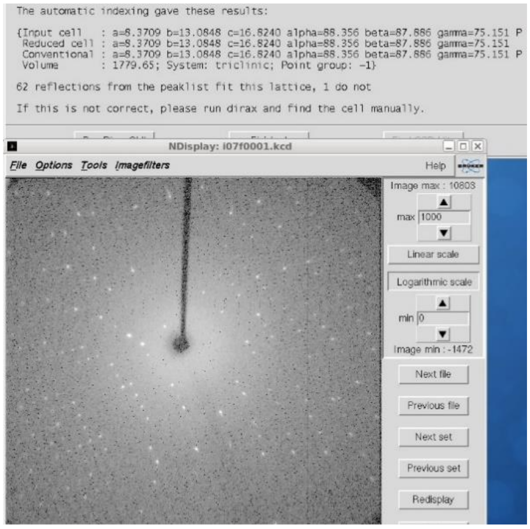

Crystal faces and unit cell determination/refinement of complex complex 9 · d_8-THF

INTENSITY STATISTICS FOR DATASET

Resolution	#Data	#Theory	%Complete	Redundancy	Mean I	Mean I/s	Rmerge	Rsigma
Inf - 2.59	205	211	97.2	10.06	109.23	56.49	0.0428	0.0162
2.59 - 1.76	472	472	100.0	7.23	72.50	44.09	0.0433	0.0198
1.76 - 1.39	690	690	100.0	6.48	51.72	37.85	0.0423	0.0221
1.39 - 1.22	668	668	100.0	6.06	37.58	33.38	0.0454	0.0248
1.22 - 1.11	683	683	100.0	5.79	31.02	29.55	0.0473	0.0269
1.11 - 1.03	660	660	100.0	5.59	26.05	27.52	0.0503	0.0296
1.03 - 0.97	688	688	100.0	5.33	22.17	24.68	0.0564	0.0331
0.97 - 0.92	694	694	100.0	5.13	18.48	21.82	0.0638	0.0374
0.92 - 0.88	688	688	100.0	4.85	16.52	19.66	0.0724	0.0422
0.88 - 0.84	785	785	100.0	4.66	13.56	16.98	0.0828	0.0502
0.84 - 0.81	745	745	100.0	4.42	12.46	15.13	0.0896	0.0574
0.81 - 0.79	548	548	100.0	4.30	11.25	13.41	0.0997	0.0658
0.79 - 0.77	572	572	100.0	4.16	10.09	11.65	0.1108	0.0779
0.77 - 0.75	708	708	100.0	4.01	8.92	9.87	0.1338	0.0959
0.75 - 0.73	731	731	100.0	3.86	8.20	8.37	0.1491	0.1167
0.73 - 0.71	831	831	100.0	3.74	7.32	6.75	0.1740	0.1459
0.71 - 0.70	429	429	100.0	3.62	6.86	5.66	0.1841	0.1801
0.70 - 0.68	991	991	100.0	3.50	5.61	4.26	0.2251	0.2413
0.68 - 0.67	564	564	100.0	3.32	4.90	3.20	0.2643	0.3272
0.67 - 0.66	553	553	100.0	3.31	4.60	2.83	0.2800	0.3791
0.66 - 0.65	601	652	92.2	3.02	4.44	2.46	0.3030	0.4350
0.75 - 0.65	4700	4751	98.9	3.50	6.08	4.94	0.2074	0.2272
Inf - 0.65	13506	13563	99.6	4.68	19.40	17.30	0.0636	0.0535

The high residual electron density could presumably be caused by anharmonic motion of the heavy atom. A resolution cut off (SHEL 99 0.73) was applied to the data set to exclude poorly determined intensities at higher diffraction angles.[13] Complete .cif-data of the compound are available under the CCDC number **CCDC-2178464**.

Table S1. Crystal data and structure refinement of **complex 9 · d_8-THF**.

Identification code	14474	
Empirical formula	$C_{35} H_{30} Bi Cl_4 D_8 N_3 O_3$	
Color	yellow	
Formula weight	907.51 g·mol^{-1}	
Temperature	100(2) K	
Wavelength	0.71073 Å	
Crystal system	Triclinic	
Space group	*P*-1, (no. 2)	
Unit cell dimensions	a = 8.3615(11) Å	α= 88.336(9)°.
	b = 13.0712(12) Å	β= 87.928(9)°.
	c = 16.774(2) Å	γ = 75.316(8)°.
Volume	1771.9(4) Å^3	
Z	2	
Density (calculated)	1.701 Mg·m^{-3}	
Absorption coefficient	5.316 mm^{-1}	
F(000)	888 e	
Crystal size	0.15 x 0.06 x 0.05 mm^3	
θ range for data collection	2.625 to 29.130°.	
Index ranges	$-11 \le h \le 11$, $-17 \le k \le 17$, $-22 \le l \le 22$	
Reflections collected	49618	
Independent reflections	9537 [R_{int} = 0.0554]	
Reflections with I>2σ(I)	8530	
Completeness to θ = 25.242°	99.9 %	
Absorption correction	Gaussian	
Max. and min. transmission	0.78122 and 0.57672	
Refinement method	Full-matrix least-squares on F^2	
Data / restraints / parameters	9537 / 0 / 421	
Goodness-of-fit on F^2	1.052	
Final R indices [I>2σ(I)]	R_1 = 0.0266	wR^2 = 0.0557
R indices (all data)	R_1 = 0.0343	wR^2 = 0.0582
Extinction coefficient	n/a	
Largest diff. peak and hole	2.588 and -1.633 e·Å^{-3}	

Table S2. Bond lengths [Å] and angles [°] **complex 9 · d_8-THF**.

Bi(1)-N(1)	2.473(2)	Bi(1)-N(2)	2.522(2)
Bi(1)-C(1)	2.183(3)	Bi(1)-C(17)	2.362(3)
Cl(1)-C(26)	1.732(3)	Cl(2)-C(27)	1.724(3)
Cl(3)-C(28)	1.722(3)	Cl(4)-C(29)	1.726(3)
O(1)-C(24)	1.227(3)	O(2)-C(31)	1.228(4)
N(1)-C(7)	1.283(4)	N(1)-C(8)	1.492(3)
N(2)-C(12)	1.286(4)	N(2)-C(13)	1.493(4)
N(3)-C(24)	1.365(4)	N(3)-C(31)	1.371(4)
C(1)-C(2)	1.386(4)	C(1)-C(6)	1.385(4)
C(2)-C(3)	1.402(4)	C(2)-C(7)	1.464(4)
C(3)-H(3)	0.9500	C(3)-C(4)	1.398(4)
C(4)-H(4)	0.9500	C(4)-C(5)	1.390(4)
C(5)-H(5)	0.9500	C(5)-C(6)	1.404(4)
C(6)-C(12)	1.474(4)	C(7)-H(7)	0.9500
C(8)-C(9)	1.527(5)	C(8)-C(10)	1.516(4)
C(8)-C(11)	1.519(4)	C(9)-H(9A)	0.9800
C(9)-H(9B)	0.9800	C(9)-H(9C)	0.9800
C(10)-H(10A)	0.9800	C(10)-H(10B)	0.9800
C(10)-H(10C)	0.9800	C(11)-H(11A)	0.9800
C(11)-H(11B)	0.9800	C(11)-H(11C)	0.9800
C(12)-H(12)	0.9500	C(13)-C(14)	1.525(5)
C(13)-C(15)	1.527(4)	C(13)-C(16)	1.525(5)
C(14)-H(14A)	0.9800	C(14)-H(14B)	0.9800
C(14)-H(14C)	0.9800	C(15)-H(15A)	0.9800
C(15)-H(15B)	0.9800	C(15)-H(15C)	0.9800
C(16)-H(16A)	0.9800	C(16)-H(16B)	0.9800
C(16)-H(16C)	0.9800	C(17)-H(17A)	0.9900
C(17)-H(17B)	0.9900	C(17)-C(18)	1.504(4)
C(18)-C(19)	1.410(4)	C(18)-C(23)	1.401(4)
C(19)-H(19)	0.9500	C(19)-C(20)	1.392(5)
C(20)-H(20)	0.9500	C(20)-C(21)	1.389(5)
C(21)-H(21)	0.9500	C(21)-C(22)	1.388(5)
C(22)-H(22)	0.9500	C(22)-C(23)	1.388(5)
C(23)-H(23)	0.9500	C(24)-C(25)	1.524(4)
C(25)-C(26)	1.373(4)	C(25)-C(30)	1.393(4)
C(26)-C(27)	1.410(4)	C(27)-C(28)	1.396(4)
C(28)-C(29)	1.405(4)	C(29)-C(30)	1.382(4)
C(30)-C(31)	1.514(4)	O(95)-C(96)	1.433(5)
O(95)-C(99)	1.427(5)	C(96)-D(96A)	0.9900
C(96)-D(96B)	0.9900	C(96)-C(97)	1.512(6)
C(97)-D(97A)	0.9900	C(97)-D(97B)	0.9900
C(97)-C(98)	1.528(6)	C(98)-D(98A)	0.9900

C(98)-D(98B)	0.9900	C(98)-C(99)	1.484(6)
C(99)-D(99A)	0.9900	C(99)-D(99B)	0.9900
N(1)-Bi(1)-N(2)	143.15(8)	C(1)-Bi(1)-N(1)	72.40(9)
C(1)-Bi(1)-N(2)	70.94(9)	C(1)-Bi(1)-C(17)	91.36(10)
C(17)-Bi(1)-N(1)	89.18(10)	C(17)-Bi(1)-N(2)	88.09(10)
C(7)-N(1)-Bi(1)	111.27(18)	C(7)-N(1)-C(8)	122.4(2)
C(8)-N(1)-Bi(1)	124.98(17)	C(12)-N(2)-Bi(1)	
111.63(18)	C(12)-N(2)-C(13)	120.8(2)	C(13)-
N(2)-Bi(1)	127.14(18)	C(24)-N(3)-C(31)	109.7(2)
C(2)-C(1)-Bi(1)	117.9(2)	C(6)-C(1)-Bi(1)	120.6(2)
C(6)-C(1)-C(2)	121.5(3)	C(1)-C(2)-C(3)	119.2(3)
C(1)-C(2)-C(7)	118.5(2)	C(3)-C(2)-C(7)	122.3(3)
C(2)-C(3)-H(3)	120.1	C(4)-C(3)-C(2)	119.7(3)
C(4)-C(3)-H(3)	120.1	C(3)-C(4)-H(4)	119.7
C(5)-C(4)-C(3)	120.5(3)	C(5)-C(4)-H(4)	119.7
C(4)-C(5)-H(5)	120.2	C(4)-C(5)-C(6)	119.6(3)
C(6)-C(5)-H(5)	120.2	C(1)-C(6)-C(5)	119.4(3)
C(1)-C(6)-C(12)	117.4(3)	C(5)-C(6)-C(12)	123.1(3)
N(1)-C(7)-C(2)	119.4(2)	N(1)-C(7)-H(7)	120.3
C(2)-C(7)-H(7)	120.3	N(1)-C(8)-C(9)	106.8(2)
N(1)-C(8)-C(10)	113.5(2)	N(1)-C(8)-C(11)	105.7(2)
C(10)-C(8)-C(9)	109.0(3)	C(10)-C(8)-C(11)	110.7(3)
C(11)-C(8)-C(9)	111.0(3)	C(8)-C(9)-H(9A)	109.5
C(8)-C(9)-H(9B)	109.5	C(8)-C(9)-H(9C)	109.5
H(9A)-C(9)-H(9B)	109.5	H(9A)-C(9)-H(9C)	109.5
H(9B)-C(9)-H(9C)	109.5	C(8)-C(10)-H(10A)	109.5
C(8)-C(10)-H(10B)	109.5	C(8)-C(10)-H(10C)	109.5
H(10A)-C(10)-H(10B)	109.5	H(10A)-C(10)-H(10C)	109.5
H(10B)-C(10)-H(10C)	109.5	C(8)-C(11)-H(11A)	109.5
C(8)-C(11)-H(11B)	109.5	C(8)-C(11)-H(11C)	109.5
H(11A)-C(11)-H(11B)	109.5	H(11A)-C(11)-H(11C)	109.5
H(11B)-C(11)-H(11C)	109.5	N(2)-C(12)-C(6)	119.3(3)
N(2)-C(12)-H(12)	120.4	C(6)-C(12)-H(12)	120.4
N(2)-C(13)-C(14)	109.3(3)	N(2)-C(13)-C(15)	109.8(3)
N(2)-C(13)-C(16)	106.9(2)	C(14)-C(13)-C(15)	110.4(3)
C(16)-C(13)-C(14)	110.4(3)	C(16)-C(13)-C(15)	110.0(3)
C(13)-C(14)-H(14A)	109.5	C(13)-C(14)-H(14B)	109.5
C(13)-C(14)-H(14C)	109.5	H(14A)-C(14)-H(14B)	109.5
H(14A)-C(14)-H(14C)	109.5	H(14B)-C(14)-H(14C)	109.5
C(13)-C(15)-H(15A)	109.5	C(13)-C(15)-H(15B)	109.5
C(13)-C(15)-H(15C)	109.5	H(15A)-C(15)-H(15B)	109.5
H(15A)-C(15)-H(15C)	109.5	H(15B)-C(15)-H(15C)	109.5
C(13)-C(16)-H(16A)	109.5	C(13)-C(16)-H(16B)	109.5

C(13)-C(16)-H(16C)	109.5	H(16A)-C(16)-H(16B)	109.5
H(16A)-C(16)-H(16C)	109.5	H(16B)-C(16)-H(16C)	109.5
Bi(1)-C(17)-H(17A)	108.4	Bi(1)-C(17)-H(17B)	108.4
H(17A)-C(17)-H(17B)	107.4	C(18)-C(17)-Bi(1)	115.7(2)
C(18)-C(17)-H(17A)	108.4	C(18)-C(17)-H(17B)	108.4
C(19)-C(18)-C(17)	120.8(3)	C(23)-C(18)-C(17)	121.7(3)
C(23)-C(18)-C(19)	117.4(3)	C(18)-C(19)-H(19)	119.6
C(20)-C(19)-C(18)	120.8(3)	C(20)-C(19)-H(19)	119.6
C(19)-C(20)-H(20)	119.6	C(21)-C(20)-C(19)	120.7(3)
C(21)-C(20)-H(20)	119.6	C(20)-C(21)-H(21)	120.5
C(22)-C(21)-C(20)	119.1(3)	C(22)-C(21)-H(21)	120.5
C(21)-C(22)-H(22)	119.7	C(21)-C(22)-C(23)	120.5(3)
C(23)-C(22)-H(22)	119.7	C(18)-C(23)-H(23)	119.3
C(22)-C(23)-C(18)	121.4(3)	C(22)-C(23)-H(23)	119.3
O(1)-C(24)-N(3)	126.8(3)	O(1)-C(24)-C(25)	124.3(3)
N(3)-C(24)-C(25)	108.9(2)	C(26)-C(25)-C(24)	132.6(3)
C(26)-C(25)-C(30)	121.3(3)	C(30)-C(25)-C(24)	106.1(2)
C(25)-C(26)-Cl(1)	121.7(2)	C(25)-C(26)-C(27)	118.5(3)
C(27)-C(26)-Cl(1)	119.8(2)	C(26)-C(27)-Cl(2)	119.8(2)
C(28)-C(27)-Cl(2)	119.8(2)	C(28)-C(27)-C(26)	120.4(3)
C(27)-C(28)-Cl(3)	119.4(2)	C(27)-C(28)-C(29)	120.3(3)
C(29)-C(28)-Cl(3)	120.3(2)	C(28)-C(29)-Cl(4)	120.3(2)
C(30)-C(29)-Cl(4)	121.1(2)	C(30)-C(29)-C(28)	118.6(3)
C(25)-C(30)-C(31)	106.2(2)	C(29)-C(30)-C(25)	121.0(3)
C(29)-C(30)-C(31)	132.8(3)	O(2)-C(31)-N(3)	125.5(3)
O(2)-C(31)-C(30)	125.3(3)	N(3)-C(31)-C(30)	109.1(2)
C(99)-O(95)-C(96)	105.4(3)	O(95)-C(96)-D(96A)	110.7
O(95)-C(96)-D(96B)	110.7	O(95)-C(96)-C(97)	105.1(3)
D(96A)-C(96)-D(96B)	108.8	C(97)-C(96)-D(96A)	110.7
C(97)-C(96)-D(96B)	110.7	C(96)-C(97)-D(97A)	111.1
C(96)-C(97)-D(97B)	111.1	C(96)-C(97)-C(98)	103.3(3)
D(97A)-C(97)-D(97B)	109.1	C(98)-C(97)-D(97A)	111.1
C(98)-C(97)-D(97B)	111.1	C(97)-C(98)-D(98A)	110.8
C(97)-C(98)-D(98B)	110.8	D(98A)-C(98)-D(98B)	108.9
C(99)-C(98)-C(97)	104.6(3)	C(99)-C(98)-D(98A)	110.8
C(99)-C(98)-D(98B)	110.8	O(95)-C(99)-C(98)	108.6(4)
O(95)-C(99)-D(99A)	110.0	O(95)-C(99)-D(99B)	110.0
C(98)-C(99)-D(99A)	110.0	C(98)-C(99)-D(99B)	110.0
D(99A)-C(99)-D(99B)	108.3		

[(2,6-(*t*BuNCH)$_2$C$_6$H$_3$)Bi(4-bromobenzyl)(tetrachlorophthalimide)] (**11**)

Single crystals of **11** were obtained by layering pentane on top of a solution of the compound in THF in an NMR tube, which was left over 4 days in the freezer (-30 °C). CCDC deposit number: 2178465.

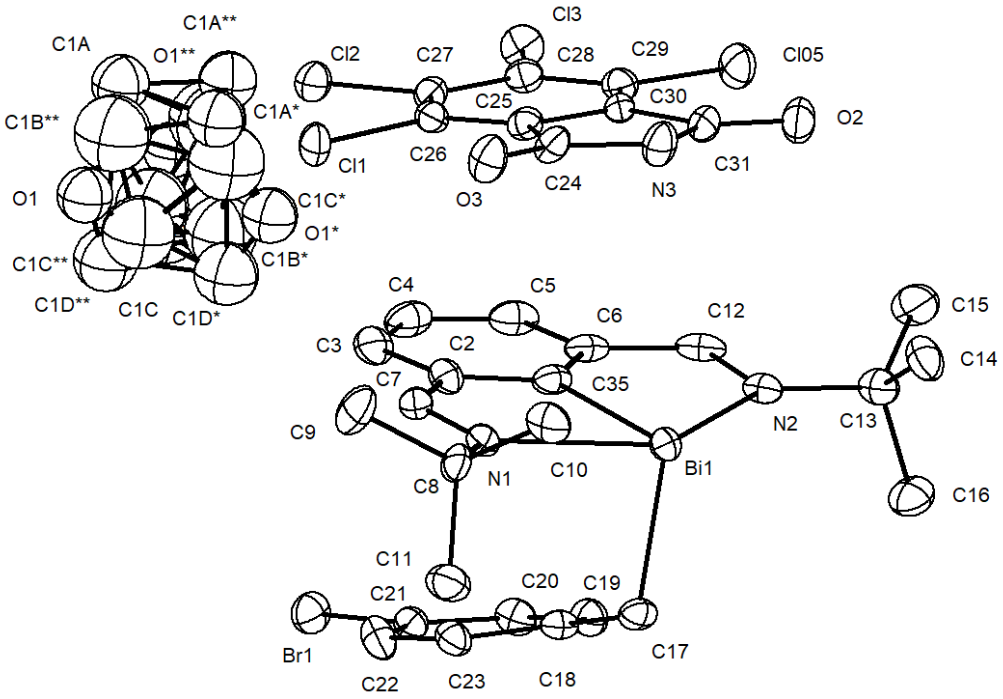

Molecular structure of **complex 11 · THF**. H and D atoms have been removed for clarity.

X-ray Crystal Structure Analysis of complex complex 11 · THF:

$C_{31.67}$ $H_{30.33}$ Bi Br Cl_4 N_3 $O_{2.17}$, M_r = 918.28 g mol^{-1}, yellow prism, crystal size 0.12 x 0.11 x 0.07 mm^3, hexagonal, space group $P6_3$ [173], a = 23.139(4) Å, c = 10.8913(11) Å, V = 5050.2(17) Å^3, T = 100(2) K, Z = 6, D_{calc} = 1.812 g·cm^3, λ = 0.71073 Å, $\mu(Mo\text{-}K_\alpha)$ = 6.775 mm^{-1}, Gaussian absorption correction (T_{min} = 0.49704, T_{max} = 0.67124), Bruker AXS Enraf-Nonius KappaCCD diffractometer with a FR591 rotating Mo-anode X-ray source, 2.689 < θ < 33.078°, 109427 measured reflections, 12769 independent reflections, 11529 reflections with $I > 2\sigma(I)$, R_{int} = 0.0572. The structure was solved by *SHELXS* and refined by full-matrix least-squares (*SHELXL*) against F^2 to R_1 = 0.0351 [$I > 2\sigma(I)$], wR_2 = 0.0780 [all data], 406 parameters, 13 restraints, absolute structure parameter Flack (x) = -0.054(5).

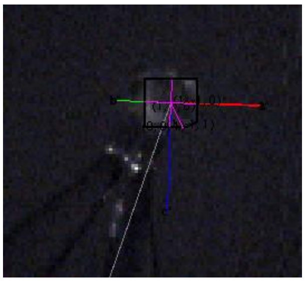

	h	k	l	distance (mm)
1.	-1	-1	0	0.035
2.	1	1	0	0.035
3.	-1	1	0	0.06
4.	1	-1	0	0.06
5.	0	0	-1	0.055
6.	0	0	1	0.055
7.	1	-1	1	0.065

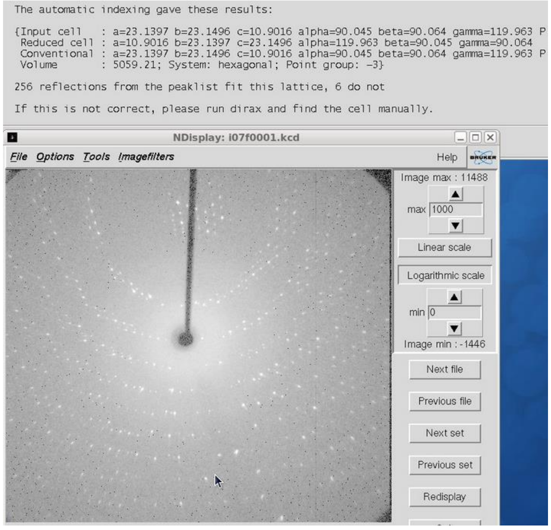

Crystal faces and unit cell determination/refinement of complex 11 · THF.

INTENSITY STATISTICS FOR DATASET

Resolution	#Data	#Theory	%Complete	Redundancy	Mean I	Mean I/s	Rmerge	Rsigma
Inf - 2.62	195	205	95.1	12.20	89.73	85.97	0.0302	0.0115
2.62 - 1.76	450	450	100.0	12.92	56.55	77.56	0.0307	0.0114
1.76 - 1.40	647	647	100.0	12.75	38.76	64.71	0.0340	0.0123
1.40 - 1.22	639	639	100.0	12.16	30.35	54.60	0.0375	0.0151
1.22 - 1.11	645	645	100.0	11.31	24.04	46.10	0.0417	0.0173
1.11 - 1.03	661	662	99.8	10.56	18.26	36.71	0.0489	0.0217
1.03 - 0.97	623	627	99.4	9.93	17.59	34.00	0.0529	0.0235
0.97 - 0.92	680	680	100.0	9.43	13.92	28.17	0.0624	0.0282
0.92 - 0.88	628	634	99.1	8.79	11.72	24.23	0.0700	0.0346
0.88 - 0.84	793	793	100.0	8.51	10.44	20.39	0.0814	0.0386
0.84 - 0.81	694	698	99.4	7.93	9.15	18.38	0.0916	0.0465
0.81 - 0.79	493	493	100.0	7.75	8.24	16.37	0.1007	0.0520
0.79 - 0.77	567	570	99.5	7.37	7.46	14.54	0.1094	0.0592
0.77 - 0.75	646	646	100.0	7.11	7.37	14.03	0.1220	0.0629
0.75 - 0.73	665	665	100.0	6.88	6.35	11.70	0.1425	0.0751
0.73 - 0.71	833	834	99.9	6.60	5.63	10.08	0.1571	0.0892
0.71 - 0.70	424	424	100.0	6.34	4.62	8.18	0.1939	0.1110
0.70 - 0.68	904	904	100.0	6.28	4.72	7.98	0.1904	0.1166
0.68 - 0.67	530	530	100.0	5.97	3.99	6.50	0.2307	0.1480
0.67 - 0.66	537	537	100.0	5.93	4.33	6.46	0.2284	0.1487
0.66 - 0.65	526	530	99.2	5.50	3.60	5.07	0.2555	0.2061
0.75 - 0.65	4419	4424	99.9	6.26	4.86	8.25	0.1853	0.1164
Inf - 0.65	12780	12813	99.7	8.54	15.02	25.72	0.0561	0.0327

The investigated crystal was twinned (TWIN 0 1 0 1 0 0 0 0 -1) with a BASF of 0.21768. One solute molecule is disordered and located on a crystallographic special position. DSR Tool as a plugin in Olex2 was used to model the disordered parts. The occupancy was fixed to be 0.1667 and isotropic displacement parameters have been used. Complete .cif-data of the compound are available under the CCDC number **CCDC-2178465**.

Table S3. Crystal data and structure refinement.

Identification code	14298	
Empirical formula	$C_{31.67}$ $H_{30.33}$ Bi Br Cl_4 N_3 $O_{2.17}$	
Color	yellow	
Formula weight	918.28 g·mol^{-1}	
Temperature	100(2) K	
Wavelength	0.71073 Å	
Crystal system	Hexagonal	
Space group	$P6_3$, (no. 173)	
Unit cell dimensions	a = 23.139(4) Å	α= 90°.
	b = 23.139(4) Å	β= 90°.
	c = 10.8913(11) Å	γ = 120°.
Volume	5050.2(17) Å^3	
Z	6	
Density (calculated)	1.812 Mg·m^{-3}	
Absorption coefficient	6.775 mm^{-1}	
F(000)	2668 e	
Crystal size	0.12 x 0.11 x 0.07 mm^3	
θ range for data collection	2.689 to 33.078°.	
Index ranges	-35 ≤ h ≤ 35, -35≤ k ≤ 35, -16 ≤ l ≤ 16	
Reflections collected	109427	
Independent reflections	12769 [R_{int} = 0.0572]	
Reflections with I>2σ(I)	11529	
Completeness to θ = 25.242°	99.8 %	
Absorption correction	Gaussian	
Max. and min. transmission	0.67124 and 0.49704	
Refinement method	Full-matrix least-squares on F^2	
Data / restraints / parameters	12769 / 13 / 406	
Goodness-of-fit on F^2	1.056	
Final R indices [I>2σ(I)]	R_1 = 0.0351	wR^2 = 0.0745
R indices (all data)	R_1 = 0.0437	wR^2 = 0.0780
Absolute structure parameter	-0.054(5)	
Extinction coefficient	n/a	
Largest diff. peak and hole	1.272 and -0.747 e·Å^{-3}	

Table S4. Bond lengths [Å] and angles [°] **complex 11 · THF**.

Bi(1)-N(1)	2.543(6)	Bi(1)-N(2)	2.546(6)
Bi(1)-C(35)	2.223(6)	Bi(1)-C(17)	2.316(7)
Br(1)-C(21)	1.904(8)	N(1)-C(7)	1.284(9)
N(1)-C(8)	1.491(10)	N(2)-C(12)	1.278(10)
N(2)-C(13)	1.490(10)	C(35)-C(2)	1.394(10)
C(35)-C(6)	1.390(10)	C(2)-C(3)	1.401(9)
C(2)-C(7)	1.466(11)	C(3)-H(3)	0.9500
C(3)-C(4)	1.386(12)	C(4)-H(4)	0.9500
C(4)-C(5)	1.387(11)	C(5)-H(5)	0.9500
C(5)-C(6)	1.402(9)	C(6)-C(12)	1.470(12)
C(7)-H(7)	0.9500	C(8)-C(9)	1.517(11)
C(8)-C(10)	1.521(12)	C(8)-C(11)	1.536(12)
C(9)-H(9A)	0.9800	C(9)-H(9B)	0.9800
C(9)-H(9C)	0.9800	C(10)-H(10A)	0.9800
C(10)-H(10B)	0.9800	C(10)-H(10C)	0.9800
C(11)-H(11A)	0.9800	C(11)-H(11B)	0.9800
C(11)-H(11C)	0.9800	C(12)-H(12)	0.9500
C(13)-C(14)	1.541(12)	C(13)-C(15)	1.537(11)
C(13)-C(16)	1.513(11)	C(14)-H(14A)	0.9800
C(14)-H(14B)	0.9800	C(14)-H(14C)	0.9800
C(15)-H(15A)	0.9800	C(15)-H(15B)	0.9800
C(15)-H(15C)	0.9800	C(16)-H(16A)	0.9800
C(16)-H(16B)	0.9800	C(16)-H(16C)	0.9800
C(17)-H(17A)	0.9900	C(17)-H(17B)	0.9900
C(17)-C(18)	1.479(10)	C(18)-C(19)	1.396(11)
C(18)-C(23)	1.396(11)	C(19)-H(19)	0.9500
C(19)-C(20)	1.390(12)	C(20)-H(20)	0.9500
C(20)-C(21)	1.392(11)	C(21)-C(22)	1.376(12)
C(22)-H(22)	0.9500	C(22)-C(23)	1.395(12)
C(23)-H(23)	0.9500	Cl(1)-C(26)	1.733(8)
Cl(2)-C(27)	1.720(7)	Cl(3)-C(28)	1.711(8)
Cl(05)-C(29)	1.737(8)	O(3)-C(24)	1.219(10)
O(2)-C(31)	1.229(9)	N(3)-C(24)	1.376(10)
N(3)-C(31)	1.352(10)	C(24)-C(25)	1.516(11)
C(25)-C(26)	1.373(11)	C(25)-C(30)	1.390(11)
C(26)-C(27)	1.402(10)	C(27)-C(28)	1.399(11)
C(28)-C(29)	1.414(11)	C(29)-C(30)	1.365(11)

C(30)-C(31)	1.528(10)	O(1)-C(1A)	1.54(10)
O(1)-C(1D)	1.04(11)	C(1A)-H(1AA)	0.9900
C(1A)-H(1AB)	0.9900	C(1A)-C(1B)	1.53(2)
C(1B)-H(1BA)	0.9900	C(1B)-H(1BB)	0.9900
C(1B)-C(1C)	1.53(2)	C(1C)-H(1CA)	0.9900
C(1C)-H(1CB)	0.9900	C(1C)-C(1D)	1.53(2)
C(1D)-H(1DA)	0.9900	C(1D)-H(1DB)	0.9900

N(1)-Bi(1)-N(2)	140.62(19)	C(35)-Bi(1)-N(1)	70.5(2)
C(35)-Bi(1)-N(2)	70.3(2)	C(35)-Bi(1)-C(17)	91.7(3)
C(17)-Bi(1)-N(1)	90.6(3)	C(17)-Bi(1)-N(2)	93.8(3)
C(7)-N(1)-Bi(1)	111.5(5)	C(7)-N(1)-C(8)	121.7(6)
C(8)-N(1)-Bi(1)	126.3(4)	C(12)-N(2)-Bi(1)	111.7(5)
C(12)-N(2)-C(13)	121.7(7)	C(13)-N(2)-Bi(1)	126.0(5)
C(2)-C(35)-Bi(1)	119.9(5)	C(6)-C(35)-Bi(1)	120.0(5)
C(6)-C(35)-C(2)	120.0(6)	C(35)-C(2)-C(3)	119.9(7)
C(35)-C(2)-C(7)	117.2(6)	C(3)-C(2)-C(7)	122.8(7)
C(2)-C(3)-H(3)	120.1	C(4)-C(3)-C(2)	119.7(7)
C(4)-C(3)-H(3)	120.1	C(3)-C(4)-H(4)	119.7
C(3)-C(4)-C(5)	120.5(6)	C(5)-C(4)-H(4)	119.7
C(4)-C(5)-H(5)	120.1	C(4)-C(5)-C(6)	119.8(7)
C(6)-C(5)-H(5)	120.1	C(35)-C(6)-C(5)	119.9(7)
C(35)-C(6)-C(12)	117.4(6)	C(5)-C(6)-C(12)	122.7(7)
N(1)-C(7)-C(2)	120.4(6)	N(1)-C(7)-H(7)	119.8
C(2)-C(7)-H(7)	119.8	N(1)-C(8)-C(9)	113.2(7)
N(1)-C(8)-C(10)	105.2(6)	N(1)-C(8)-C(11)	106.8(7)
C(9)-C(8)-C(10)	110.4(7)	C(9)-C(8)-C(11)	111.1(7)
C(10)-C(8)-C(11)	110.0(7)	C(8)-C(9)-H(9A)	109.5
C(8)-C(9)-H(9B)	109.5	C(8)-C(9)-H(9C)	109.5
H(9A)-C(9)-H(9B)	109.5	H(9A)-C(9)-H(9C)	109.5
H(9B)-C(9)-H(9C)	109.5	C(8)-C(10)-H(10A)	109.5
C(8)-C(10)-H(10B)	109.5	C(8)-C(10)-H(10C)	109.5
H(10A)-C(10)-H(10B)	109.5	H(10A)-C(10)-H(10C)	109.5
H(10B)-C(10)-H(10C)	109.5	C(8)-C(11)-H(11A)	109.5
C(8)-C(11)-H(11B)	109.5	C(8)-C(11)-H(11C)	109.5
H(11A)-C(11)-H(11B)	109.5	H(11A)-C(11)-H(11C)	109.5
H(11B)-C(11)-H(11C)	109.5	N(2)-C(12)-C(6)	120.0(7)
N(2)-C(12)-H(12)	120.0	C(6)-C(12)-H(12)	120.0
N(2)-C(13)-C(14)	105.7(6)	N(2)-C(13)-C(15)	113.1(7)
N(2)-C(13)-C(16)	107.2(7)	C(15)-C(13)-C(14)	108.7(7)
C(16)-C(13)-C(14)	111.1(7)	C(16)-C(13)-C(15)	110.9(7)
C(13)-C(14)-H(14A)	109.5	C(13)-C(14)-H(14B)	109.5
C(13)-C(14)-H(14C)	109.5	H(14A)-C(14)-H(14B)	109.5
H(14A)-C(14)-H(14C)	109.5	H(14B)-C(14)-H(14C)	109.5
C(13)-C(15)-H(15A)	109.5	C(13)-C(15)-H(15B)	109.5
C(13)-C(15)-H(15C)	109.5	H(15A)-C(15)-H(15B)	109.5

H(15A)-C(15)-H(15C)	109.5	H(15B)-C(15)-H(15C)	109.5
C(13)-C(16)-H(16A)	109.5	C(13)-C(16)-H(16B)	109.5
C(13)-C(16)-H(16C)	109.5	H(16A)-C(16)-H(16B)	109.5
H(16A)-C(16)-H(16C)	109.5	H(16B)-C(16)-H(16C)	109.5
Bi(1)-C(17)-H(17A)	108.5	Bi(1)-C(17)-H(17B)	108.5
H(17A)-C(17)-H(17B)	107.5	C(18)-C(17)-Bi(1)	115.1(4)
C(18)-C(17)-H(17A)	108.5	C(18)-C(17)-H(17B)	108.5
C(19)-C(18)-C(17)	121.0(7)	C(19)-C(18)-C(23)	117.7(7)
C(23)-C(18)-C(17)	121.3(7)	C(18)-C(19)-H(19)	119.0
C(20)-C(19)-C(18)	122.0(8)	C(20)-C(19)-H(19)	119.0
C(19)-C(20)-H(20)	121.0	C(19)-C(20)-C(21)	118.1(7)
C(21)-C(20)-H(20)	121.0	C(20)-C(21)-Br(1)	117.8(6)
C(22)-C(21)-Br(1)	120.5(6)	C(22)-C(21)-C(20)	121.7(7)
C(21)-C(22)-H(22)	120.6	C(21)-C(22)-C(23)	118.8(8)
C(23)-C(22)-H(22)	120.6	C(18)-C(23)-H(23)	119.3
C(22)-C(23)-C(18)	121.4(8)	C(22)-C(23)-H(23)	119.3
C(31)-N(3)-C(24)	109.1(6)	O(3)-C(24)-N(3)	126.4(7)
O(3)-C(24)-C(25)	124.7(7)	N(3)-C(24)-C(25)	108.8(6)
C(26)-C(25)-C(24)	131.9(7)	C(26)-C(25)-C(30)	121.1(7)
C(30)-C(25)-C(24)	107.0(6)	C(25)-C(26)-Cl(1)	121.0(6)
C(25)-C(26)-C(27)	119.2(7)	C(27)-C(26)-Cl(1)	119.7(6)
C(26)-C(27)-Cl(2)	119.9(6)	C(28)-C(27)-Cl(2)	120.0(6)
C(28)-C(27)-C(26)	120.1(7)	C(27)-C(28)-Cl(3)	119.9(6)
C(27)-C(28)-C(29)	119.2(7)	C(29)-C(28)-Cl(3)	120.9(6)
C(28)-C(29)-Cl(05)	119.1(6)	C(30)-C(29)-Cl(05)	121.0(6)
C(30)-C(29)-C(28)	119.9(7)	C(25)-C(30)-C(31)	104.8(6)
C(29)-C(30)-C(25)	120.5(7)	C(29)-C(30)-C(31)	134.7(7)
O(2)-C(31)-N(3)	126.6(7)	O(2)-C(31)-C(30)	123.1(7)
N(3)-C(31)-C(30)	110.3(6)	C(1D)-O(1)-C(1A)	121(7)
O(1)-C(1A)-H(1AA)	112.7	O(1)-C(1A)-H(1AB)	112.7
H(1AA)-C(1A)-H(1AB)	110.2	C(1B)-C(1A)-O(1)	95(4)
C(1B)-C(1A)-H(1AA)	112.7	C(1B)-C(1A)-H(1AB)	112.7
C(1A)-C(1B)-H(1BA)	112.0	C(1A)-C(1B)-H(1BB)	112.0
H(1BA)-C(1B)-H(1BB)	109.6	C(1C)-C(1B)-C(1A)	99(3)
C(1C)-C(1B)-H(1BA)	112.0	C(1C)-C(1B)-H(1BB)	112.0
C(1B)-C(1C)-H(1CA)	112.0	C(1B)-C(1C)-H(1CB)	112.0
C(1B)-C(1C)-C(1D)	99(3)	H(1CA)-C(1C)-H(1CB)	109.7
C(1D)-C(1C)-H(1CA)	112.0	C(1D)-C(1C)-H(1CB)	112.0
O(1)-C(1D)-C(1C)	99(7)	O(1)-C(1D)-H(1DA)	112.0
O(1)-C(1D)-H(1DB)	112.0	C(1C)-C(1D)-H(1DA)	112.0
C(1C)-C(1D)-H(1DB)	112.0	H(1DA)-C(1D)-H(1DB)	109.7

4,5,6,7-Tetrachloro-2-(2-(4-(4-chlorobenzoyl)phenoxy)propan-2-yl)isoindoline-1,3-dione (**51**)

Single crystals of **51** were obtained by slow gas-diffusion of pentane into a solution of the title compound in 1,2-dichloroethane over 3 days in the fridge (5 °C). CCDC deposit number: 2178503

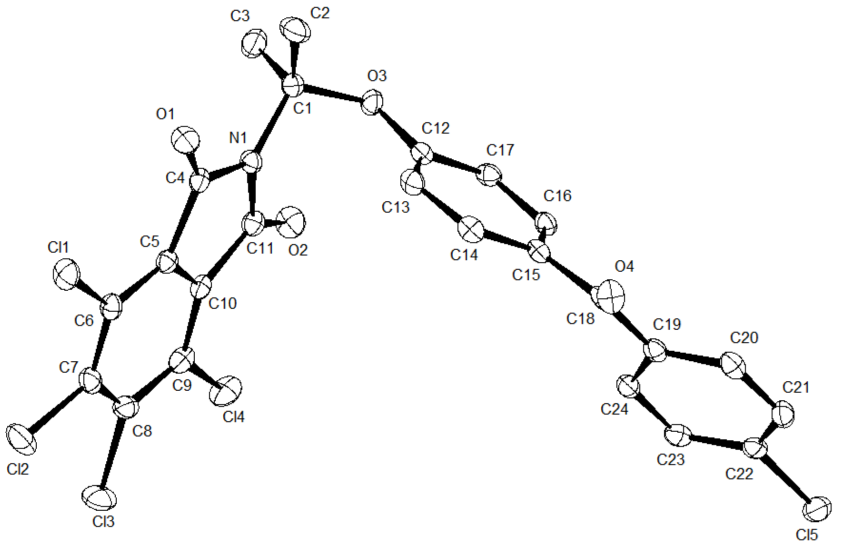

Table S5. Crystal data and structure refinement.

Identification code	14395	
Empirical formula	$C_{24}H_{14}Cl_5NO_4$	
Color	colourless	
Formula weight	557.61 $g \cdot mol^{-1}$	
Temperature	100(2) K	
Wavelength	0.71073 Å	
Crystal system	MONOCLINIC	
Space group	**$P2_1/c$, (no. 14)**	
Unit cell dimensions	a = 6.1390(2) Å	α= 90°.
	b = 15.5177(7) Å	β= 90.163(2)°.
	c = 24.2634(10) Å	γ = 90°.
Volume	2311.40(16) $Å^3$	
Z	4	
Density (calculated)	1.602 $Mg \cdot m^{-3}$	
Absorption coefficient	0.662 mm^{-1}	
F(000)	1128 e	
Crystal size	0.121 x 0.033 x 0.021 mm^3	
θ range for data collection	1.558 to 31.013°.	
Index ranges	-8 ≤ h ≤ 8, -22≤ k ≤ 22, -35 ≤ l ≤ 35	
Reflections collected	69195	
Independent reflections	7336 [R_{int} = 0.0824]	
Reflections with I>2σ(I)	4830	
Completeness to θ = 25.242°	100.0 %	
Absorption correction	Gaussian	
Max. and min. transmission	0.99and 0.95	
Refinement method	Full-matrix least-squares on F^2	
Data / restraints / parameters	7336 / 0 / 309	

Goodness-of-fit on F^2	1.005	
Final R indices [I>2σ(I)]	R_1 = 0.0408	wR^2 = 0.0760
R indices (all data)	R_1 = 0.0864	wR^2 = 0.0894
Largest diff. peak and hole	0.4 and -0.4 e · Å^{-3}	

Table S6. Bond lengths [Å] and angles [°] for 51.

Cl(1)-C(6)	1.7177(18)	Cl(2)-C(7)	1.710(2)
Cl(3)-C(8)	1.7183(19)	Cl(4)-C(9)	1.7128(18)
Cl(5)-C(22)	1.7454(18)	O(1)-C(4)	1.199(2)
O(2)-C(11)	1.200(2)	O(3)-C(1)	1.434(2)
O(3)-C(12)	1.368(2)	O(4)-C(18)	1.226(2)
N(1)-C(1)	1.482(2)	N(1)-C(4)	1.405(2)
N(1)-C(11)	1.410(2)	C(1)-C(2)	1.520(3)
C(1)-C(3)	1.518(3)	C(4)-C(5)	1.500(2)
C(5)-C(6)	1.374(3)	C(5)-C(10)	1.384(2)
C(6)-C(7)	1.401(3)	C(7)-C(8)	1.397(3)
C(8)-C(9)	1.397(3)	C(9)-C(10)	1.379(2)
C(10)-C(11)	1.482(3)	C(12)-C(13)	1.395(3)
C(12)-C(17)	1.393(2)	C(13)-C(14)	1.380(3)
C(14)-C(15)	1.396(2)	C(15)-C(16)	1.395(3)
C(15)-C(18)	1.484(3)	C(16)-C(17)	1.374(3)
C(18)-C(19)	1.496(3)	C(19)-C(20)	1.397(3)
C(19)-C(24)	1.391(2)	C(20)-C(21)	1.384(3)
C(21)-C(22)	1.385(3)	C(22)-C(23)	1.378(3)
C(23)-C(24)	1.382(3)		
C(12)-O(3)-C(1)	122.97(14)	C(4)-N(1)-C(1)	127.58(14)
C(4)-N(1)-C(11)	111.58(14)	C(11)-N(1)-C(1)	120.50(15)
O(3)-C(1)-N(1)	108.28(14)	O(3)-C(1)-C(2)	110.32(16)
O(3)-C(1)-C(3)	104.30(15)	N(1)-C(1)-C(2)	114.10(16)
N(1)-C(1)-C(3)	109.11(16)	C(3)-C(1)-C(2)	110.24(16)
O(1)-C(4)-N(1)	127.05(17)	O(1)-C(4)-C(5)	127.43(17)
N(1)-C(4)-C(5)	105.52(14)	C(6)-C(5)-C(4)	130.60(16)
C(6)-C(5)-C(10)	121.11(16)	C(10)-C(5)-C(4)	108.25(15)
C(5)-C(6)-Cl(1)	121.53(14)	C(5)-C(6)-C(7)	118.16(16)
C(7)-C(6)-Cl(1)	120.30(14)	C(6)-C(7)-Cl(2)	119.82(15)
C(8)-C(7)-Cl(2)	119.50(14)	C(8)-C(7)-C(6)	120.68(17)
C(7)-C(8)-Cl(3)	119.85(15)	C(9)-C(8)-Cl(3)	119.80(14)
C(9)-C(8)-C(7)	120.35(17)	C(8)-C(9)-Cl(4)	120.96(14)
C(10)-C(9)-Cl(4)	121.01(15)	C(10)-C(9)-C(8)	118.03(16)
C(5)-C(10)-C(11)	108.63(15)	C(9)-C(10)-C(5)	121.66(17)
C(9)-C(10)-C(11)	129.64(16)	O(2)-C(11)-N(1)	126.07(17)
O(2)-C(11)-C(10)	127.96(16)	N(1)-C(11)-C(10)	105.96(14)
O(3)-C(12)-C(13)	126.83(16)	O(3)-C(12)-C(17)	113.40(16)
C(17)-C(12)-C(13)	119.77(17)	C(14)-C(13)-C(12)	119.48(16)

C(13)-C(14)-C(15)	121.30(17)	C(14)-C(15)-C(18)	119.42(16)
C(16)-C(15)-C(14)	118.25(17)	C(16)-C(15)-C(18)	122.27(16)
C(17)-C(16)-C(15)	121.10(17)	C(16)-C(17)-C(12)	120.04(17)
O(4)-C(18)-C(15)	120.66(16)	O(4)-C(18)-C(19)	119.64(17)
C(15)-C(18)-C(19)	119.69(15)	C(20)-C(19)-C(18)	118.94(16)
C(24)-C(19)-C(18)	121.66(16)	C(24)-C(19)-C(20)	119.35(17)
C(21)-C(20)-C(19)	120.36(17)	C(20)-C(21)-C(22)	118.86(18)
C(21)-C(22)-Cl(5)	119.85(15)	C(23)-C(22)-Cl(5)	118.30(14)
C(23)-C(22)-C(21)	121.84(17)	C(22)-C(23)-C(24)	118.97(17)
C(23)-C(24)-C(19)	120.60(17)		

9. NMR Spectra

4,5,6,7-Tetrachloro-1,3-dioxoisoindolin-2-yl 2-phenylacetate (2)

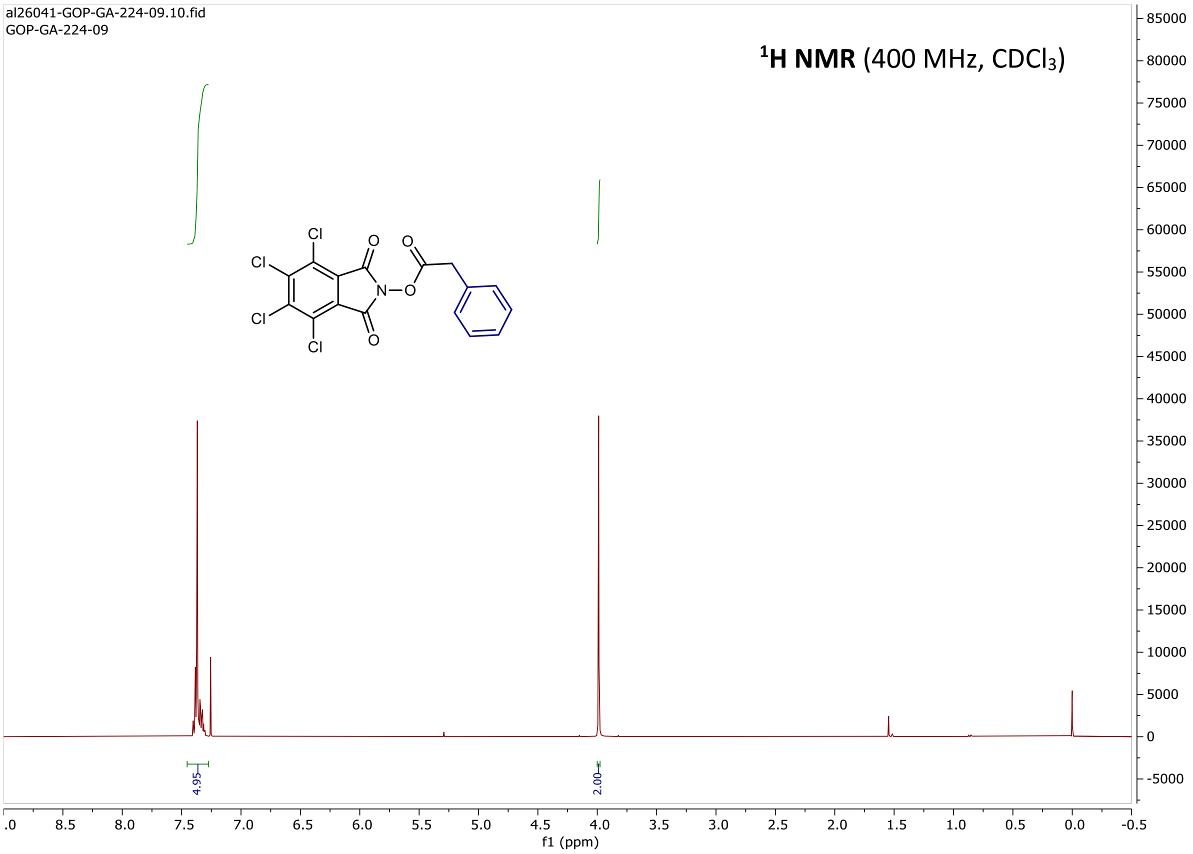

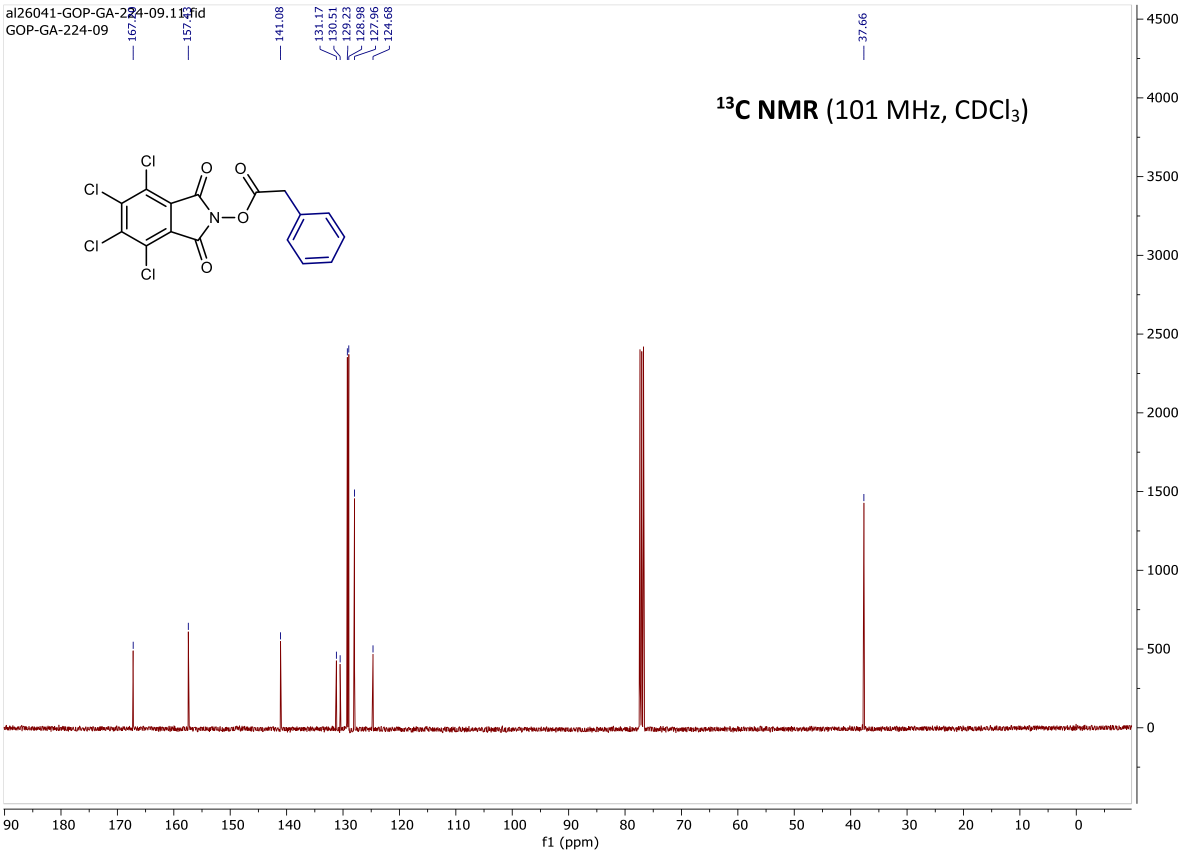

4,5,6,7-Tetrachloro-2-(3-cyclopropyl-2-oxopropyl)isoindoline-1,3-dione (**17**)

[1]**H NMR** (600 MHz, $CDCl_3$)

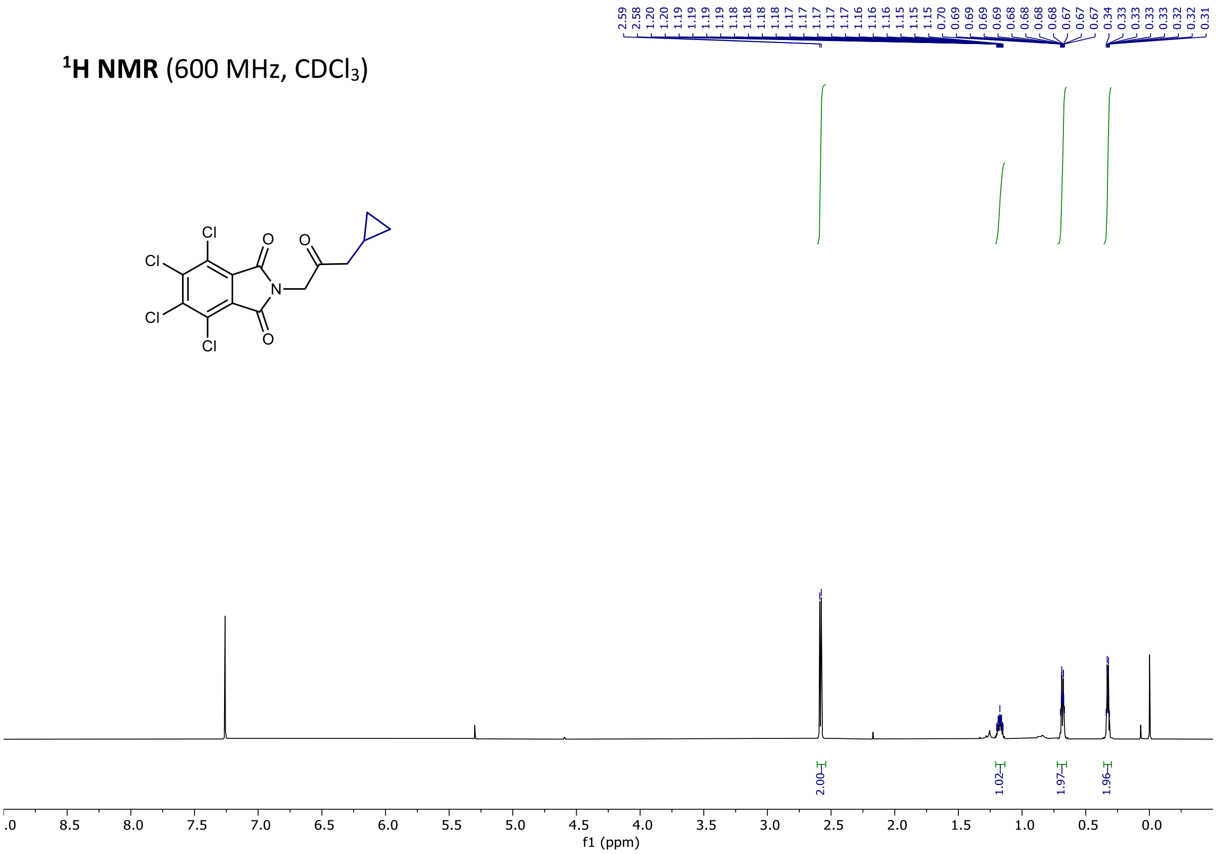

[13]**C NMR** (151 MHz, $CDCl_3$)

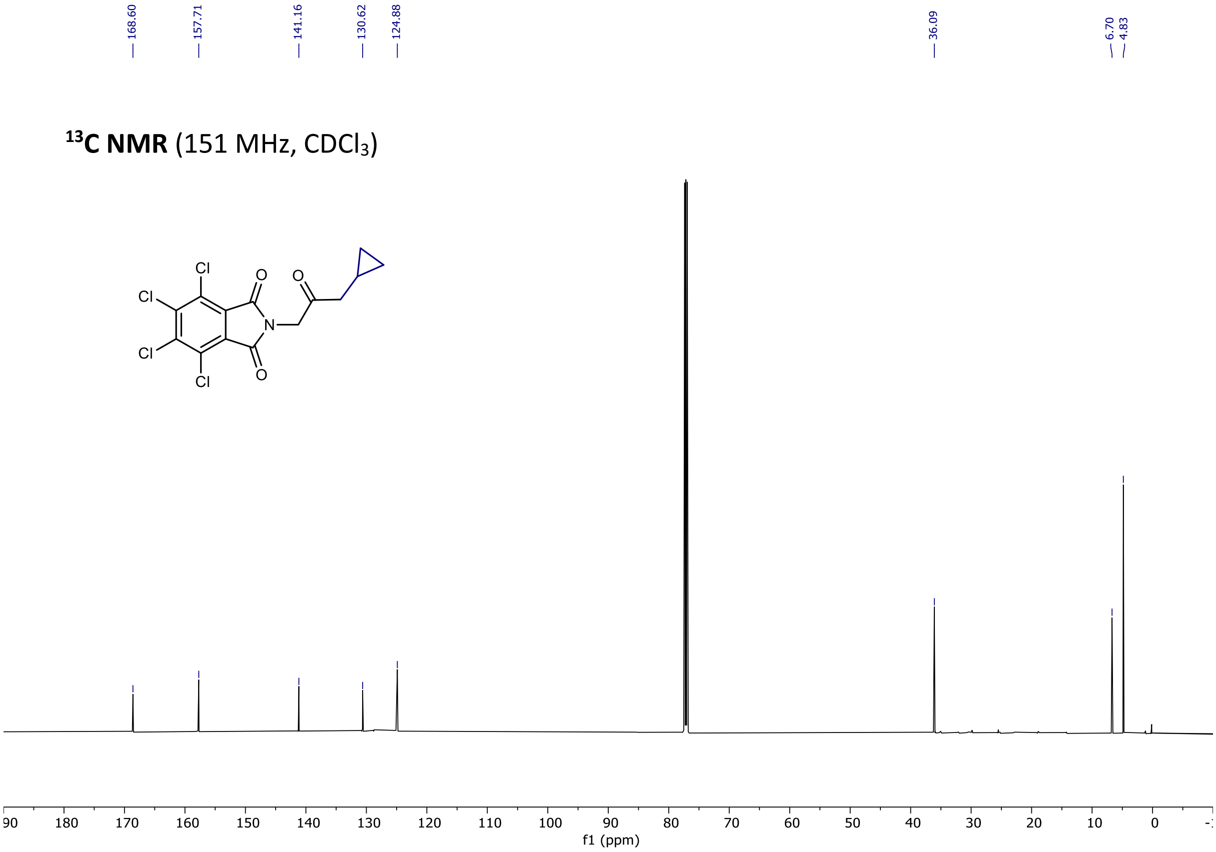

4,5,6,7-Tetrachloro-1,3-dioxoisoindolin-2-yl 1-tosylpiperidine-4-carboxylate (20)

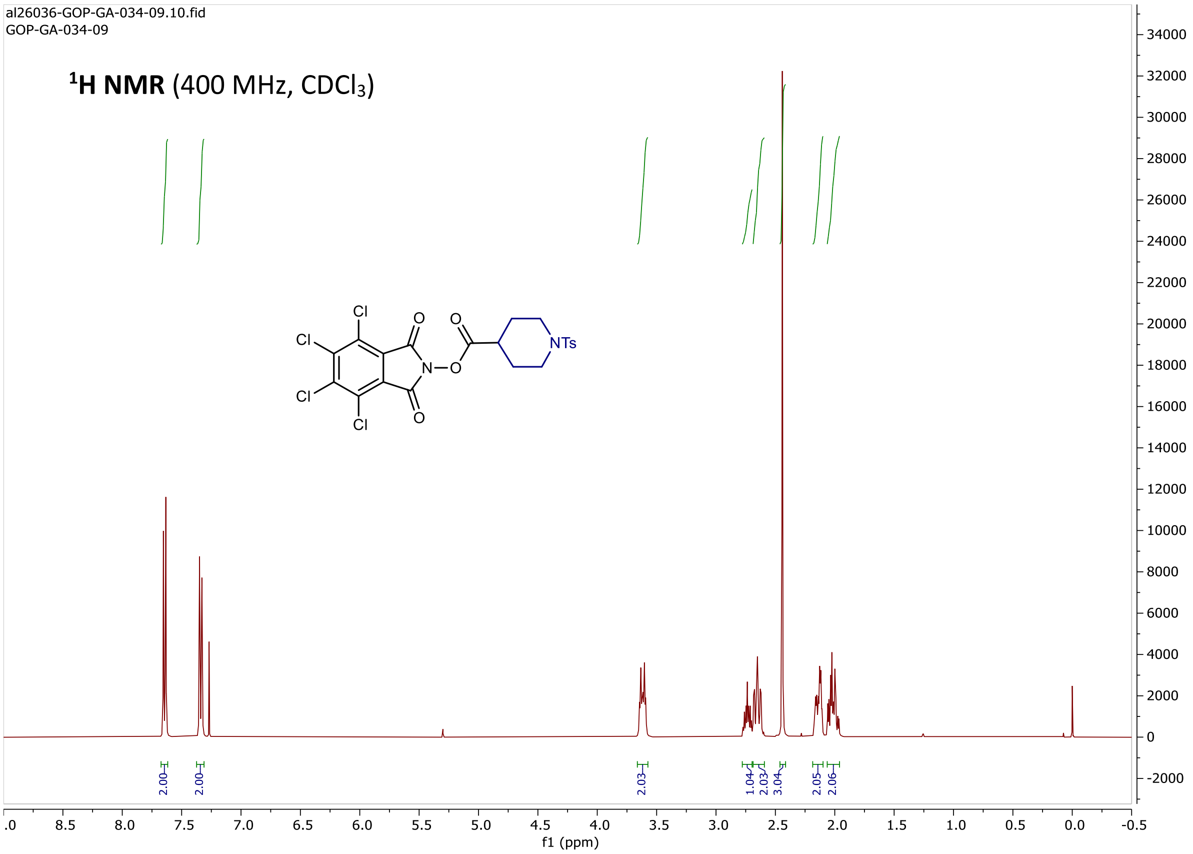

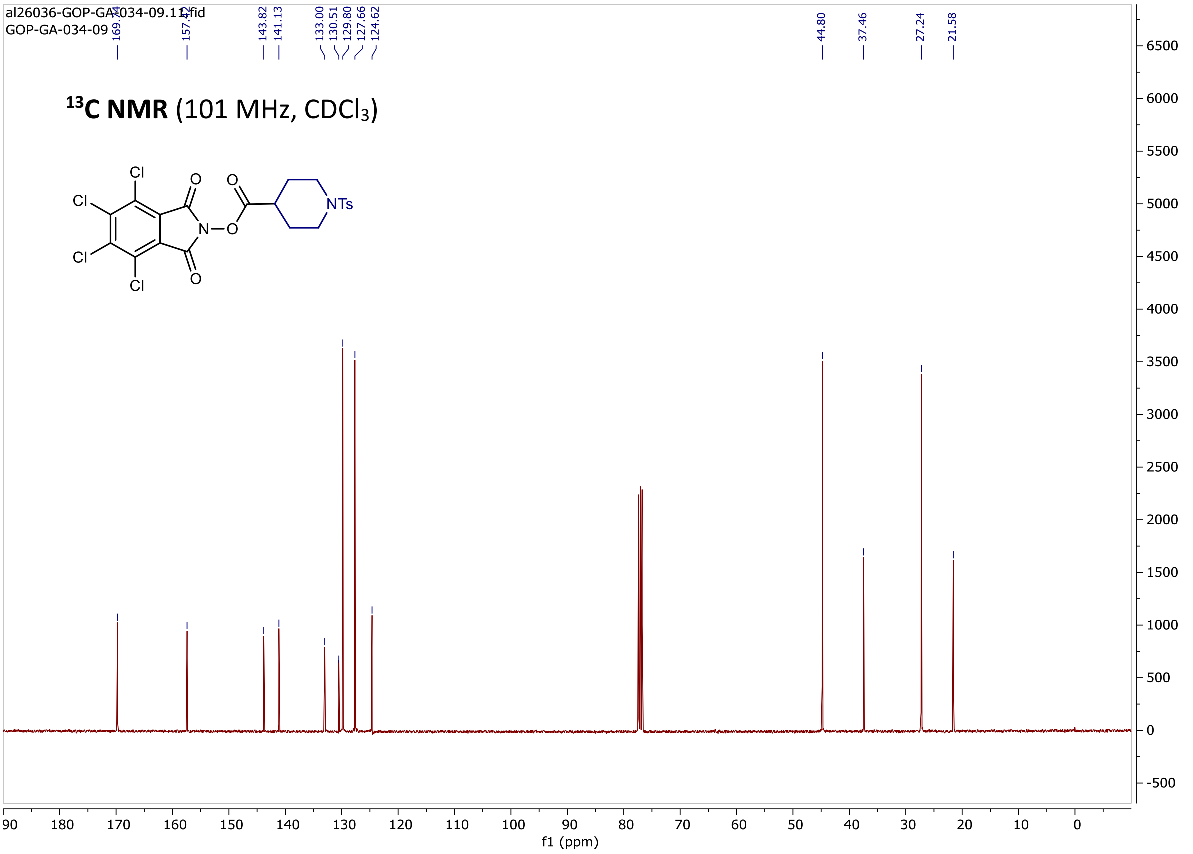

1-(*tert*-Butyl) 2-(4,5,6,7-tetrachloro-1,3-dioxoisoindolin-2-yl) pyrrolidine-1,2-dicarboxylate (22)

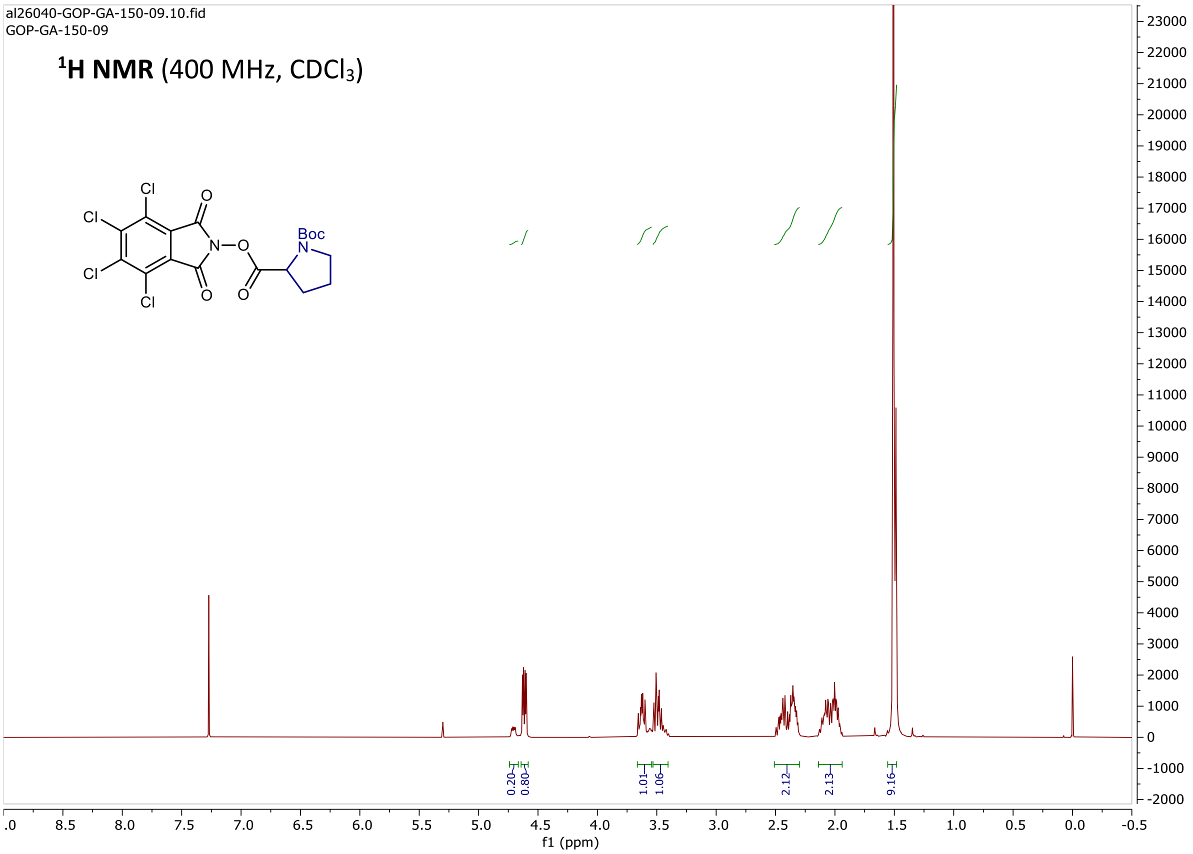

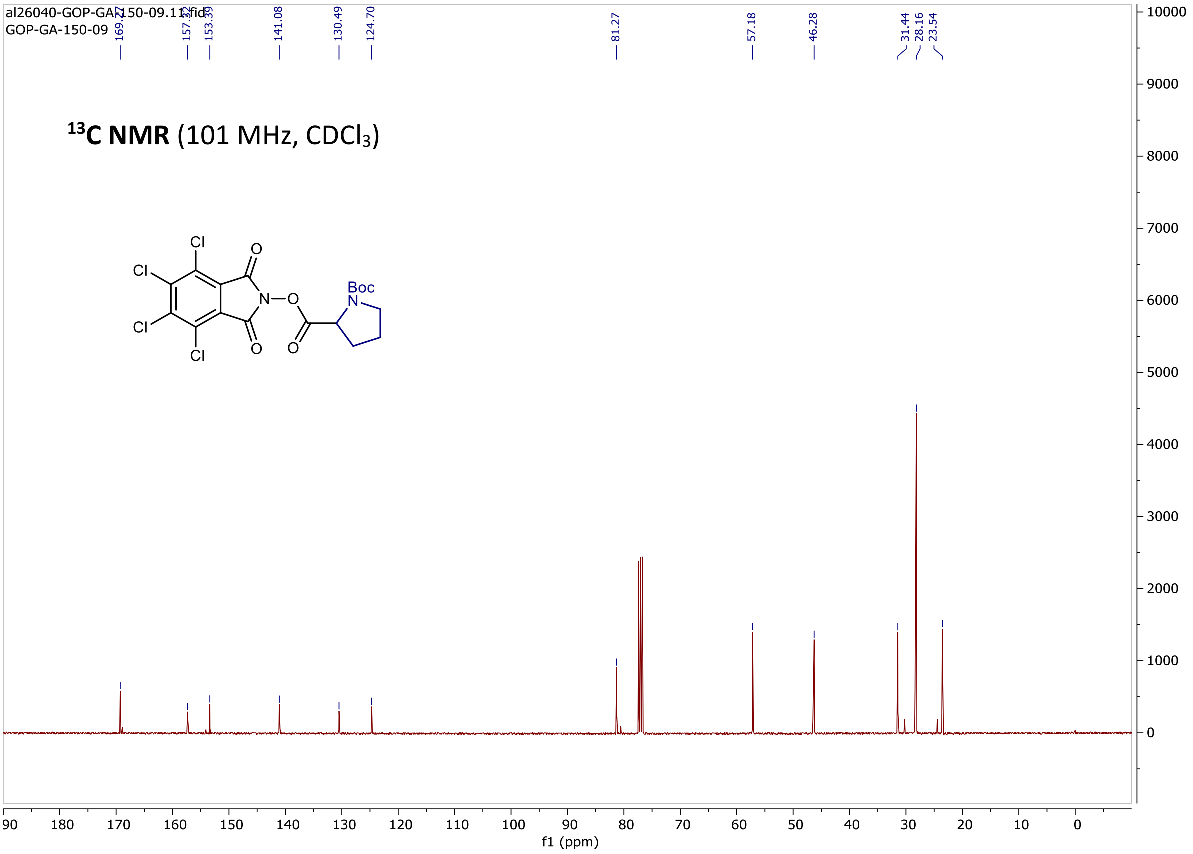

4,5,6,7-Tetrachloro-1,3-dioxoisoindolin-2-yl 2-(4-bromophenyl)acetate (S6)

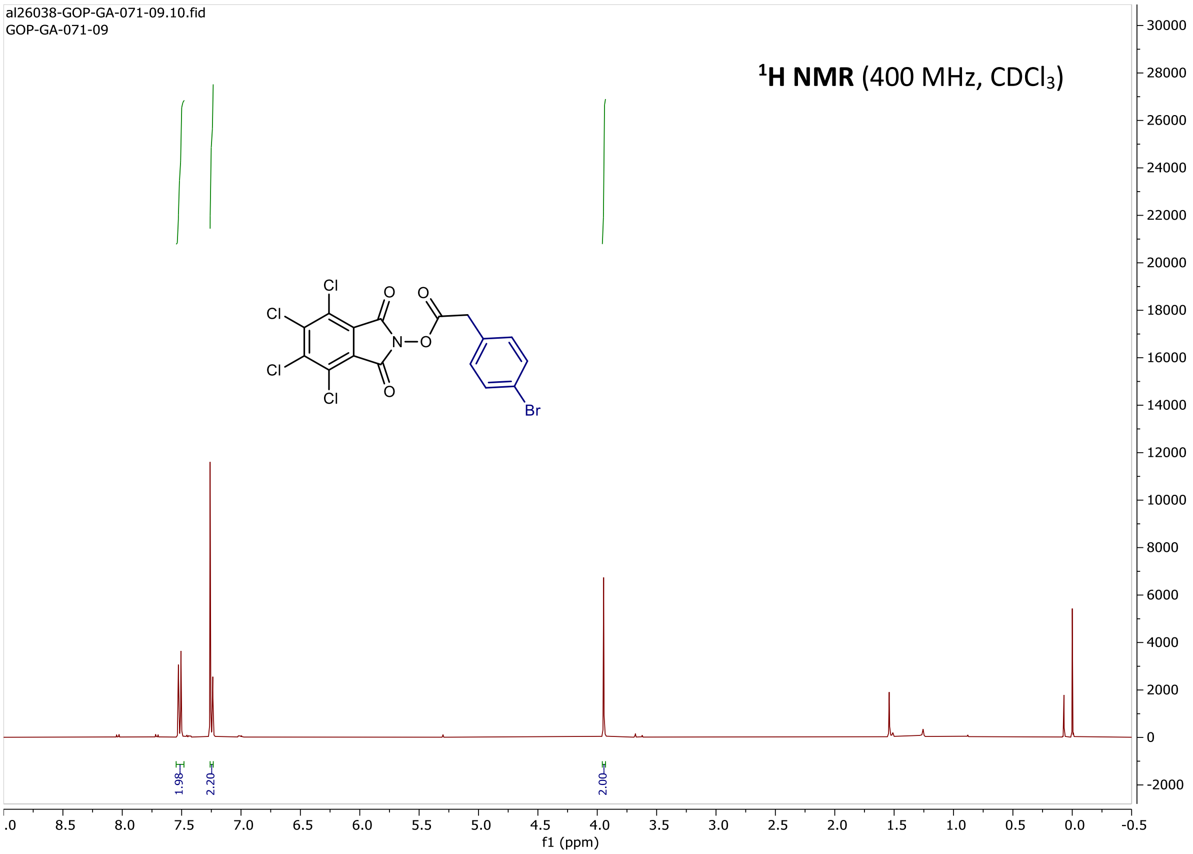

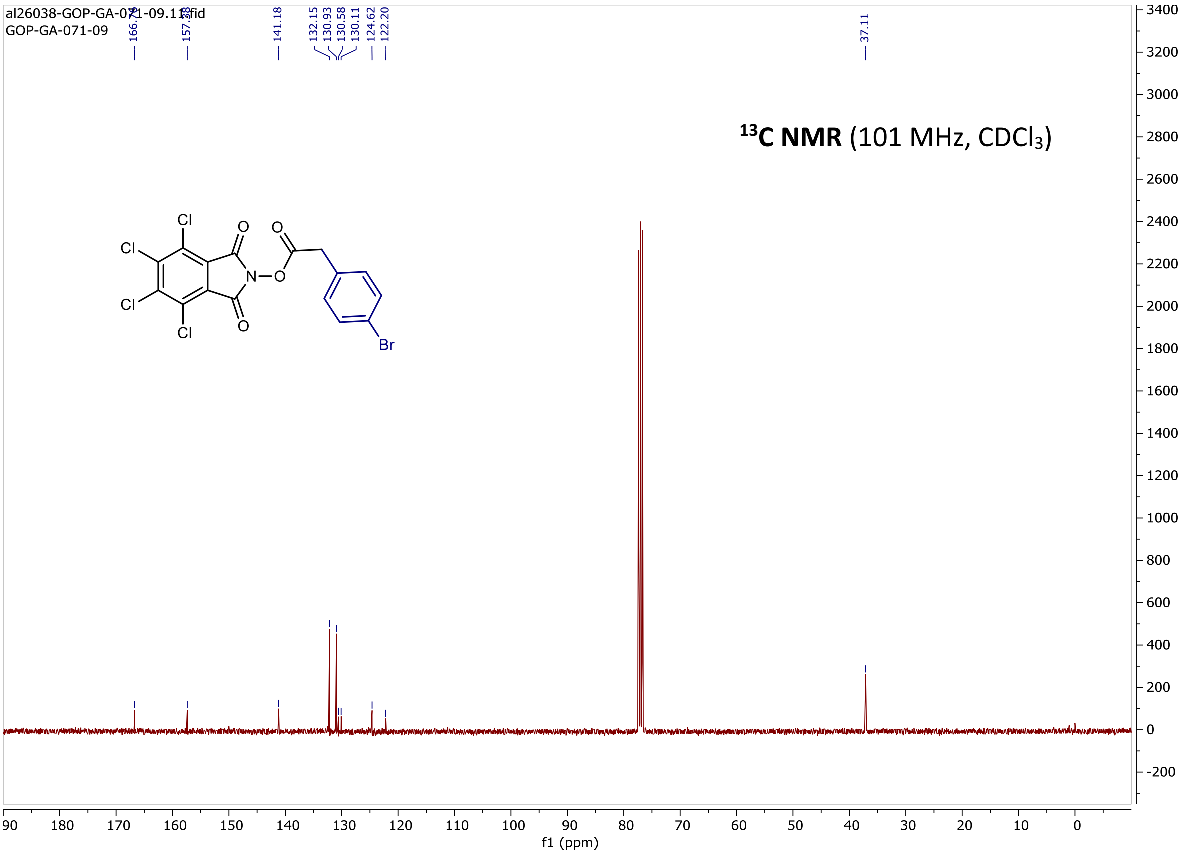

4,5,6,7-Tetrachloro-1,3-dioxoisoindolin-2-yl adamantane-1-carboxylate (S7)

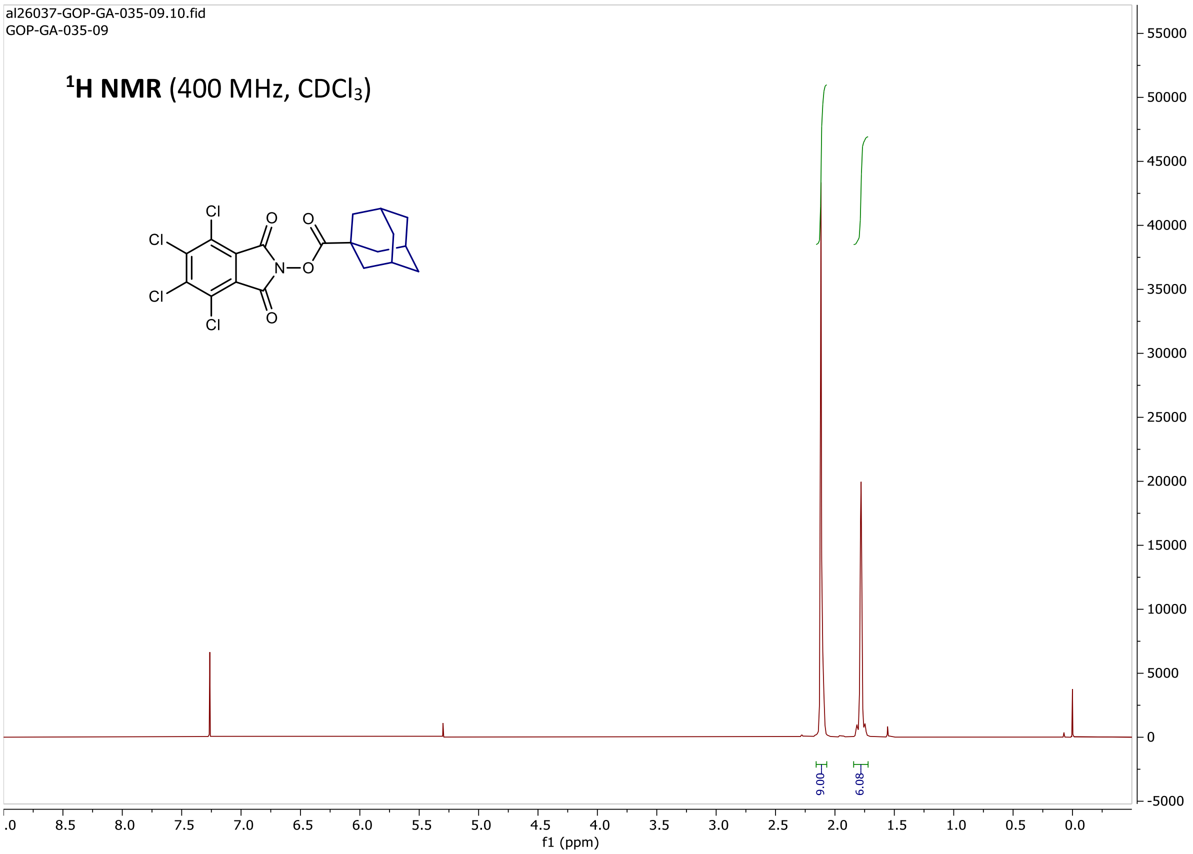

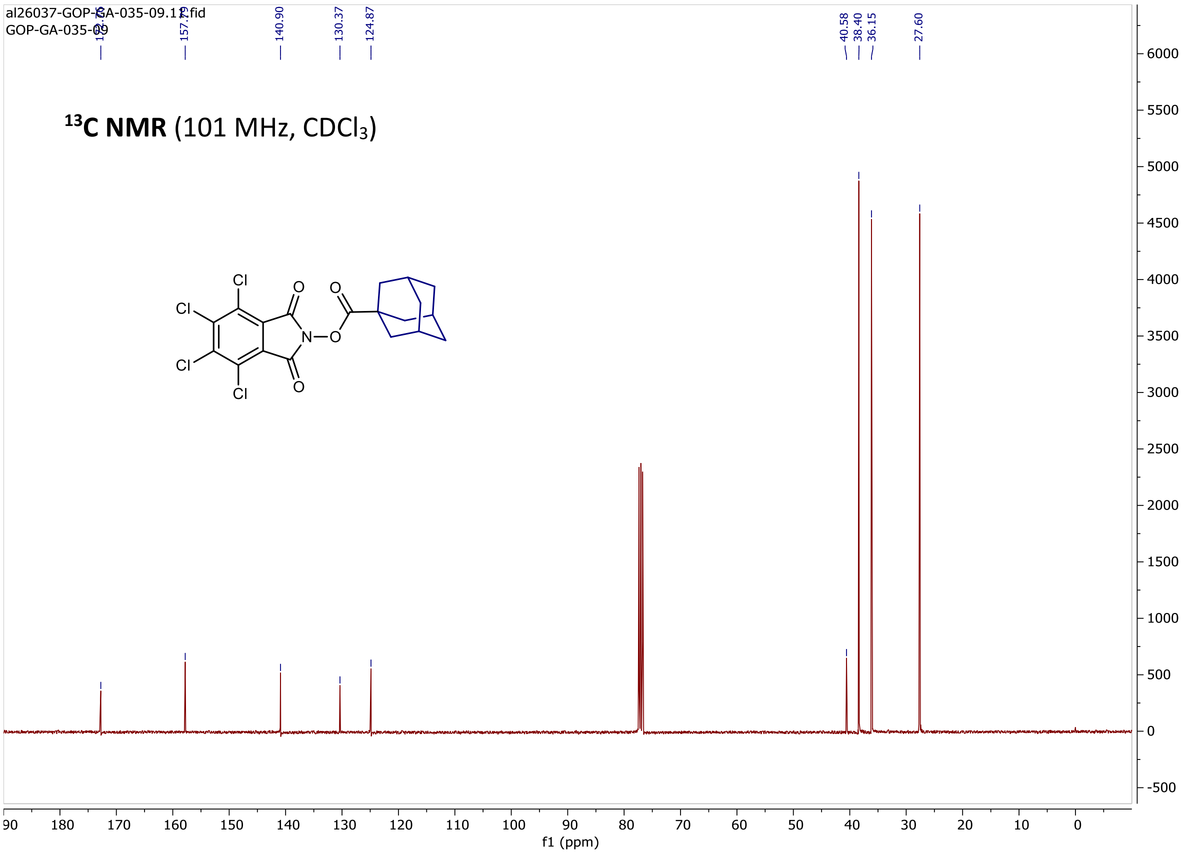

4,5,6,7-Tetrachloro-1,3-dioxoisoindolin-2-yl acetylprolinate (S8)

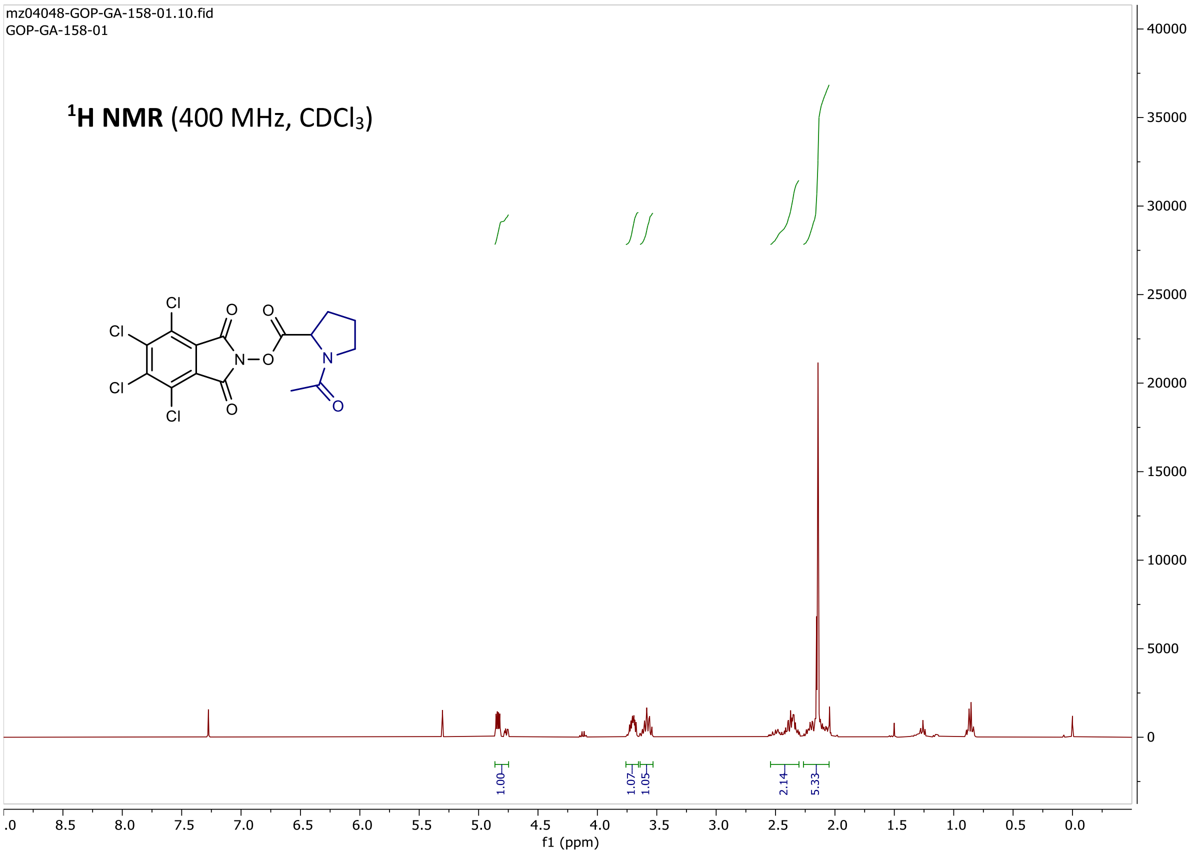

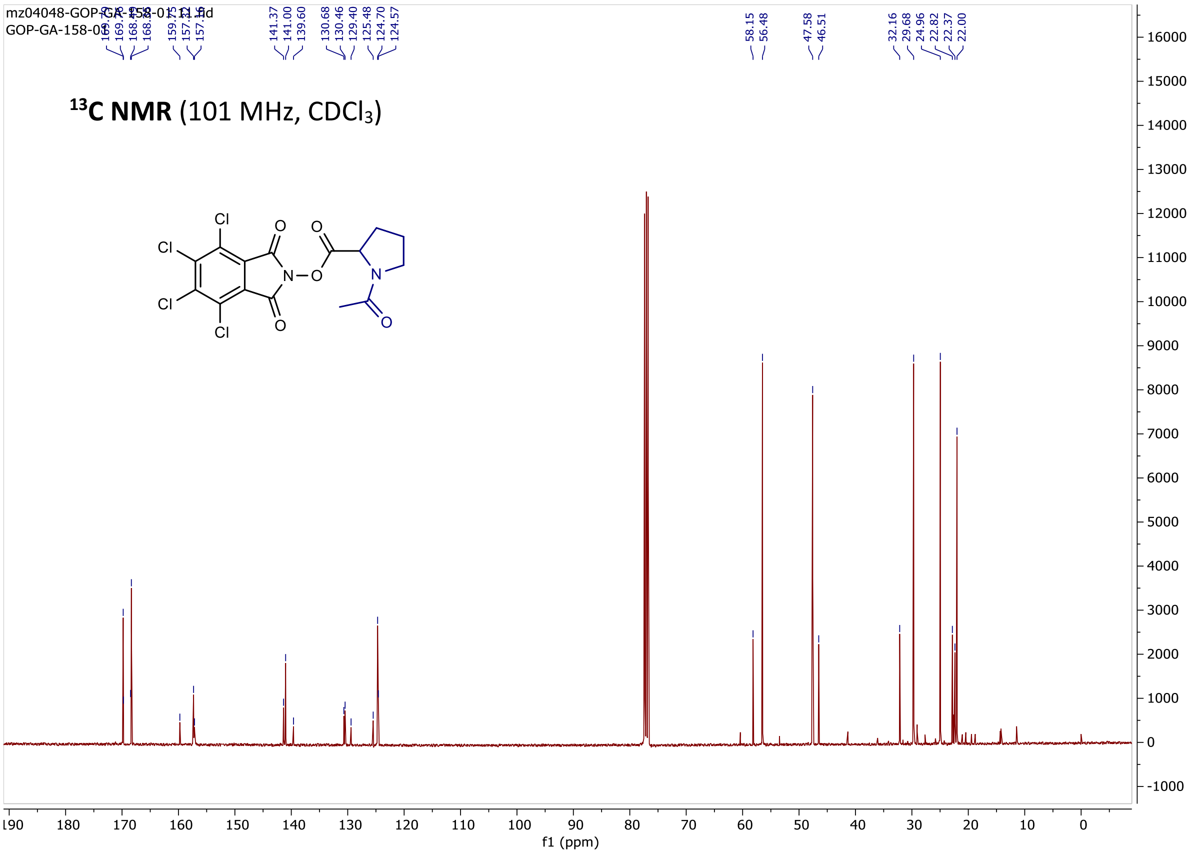

1-Benzyl 2-(4,5,6,7-tetrachloro-1,3-dioxoisoindolin-2-yl) piperidine-1,2-dicarboxylate (S9)

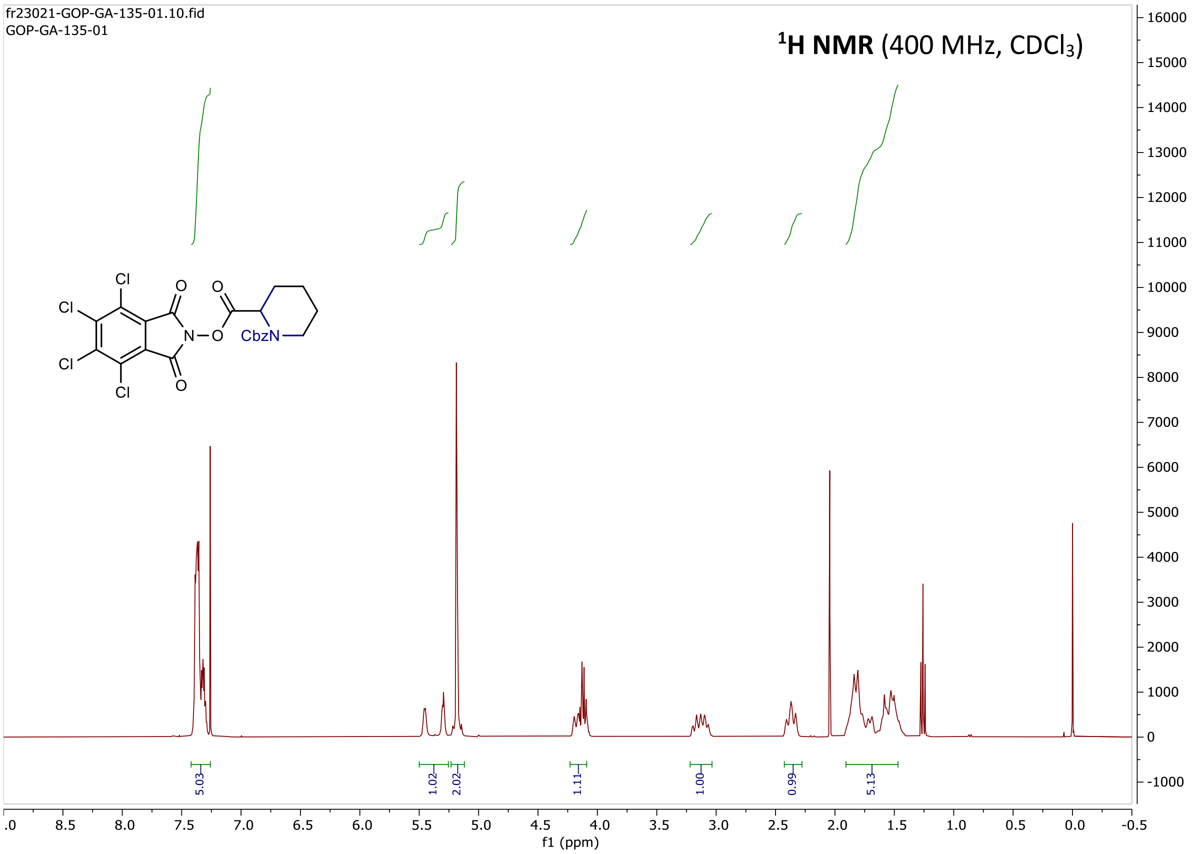

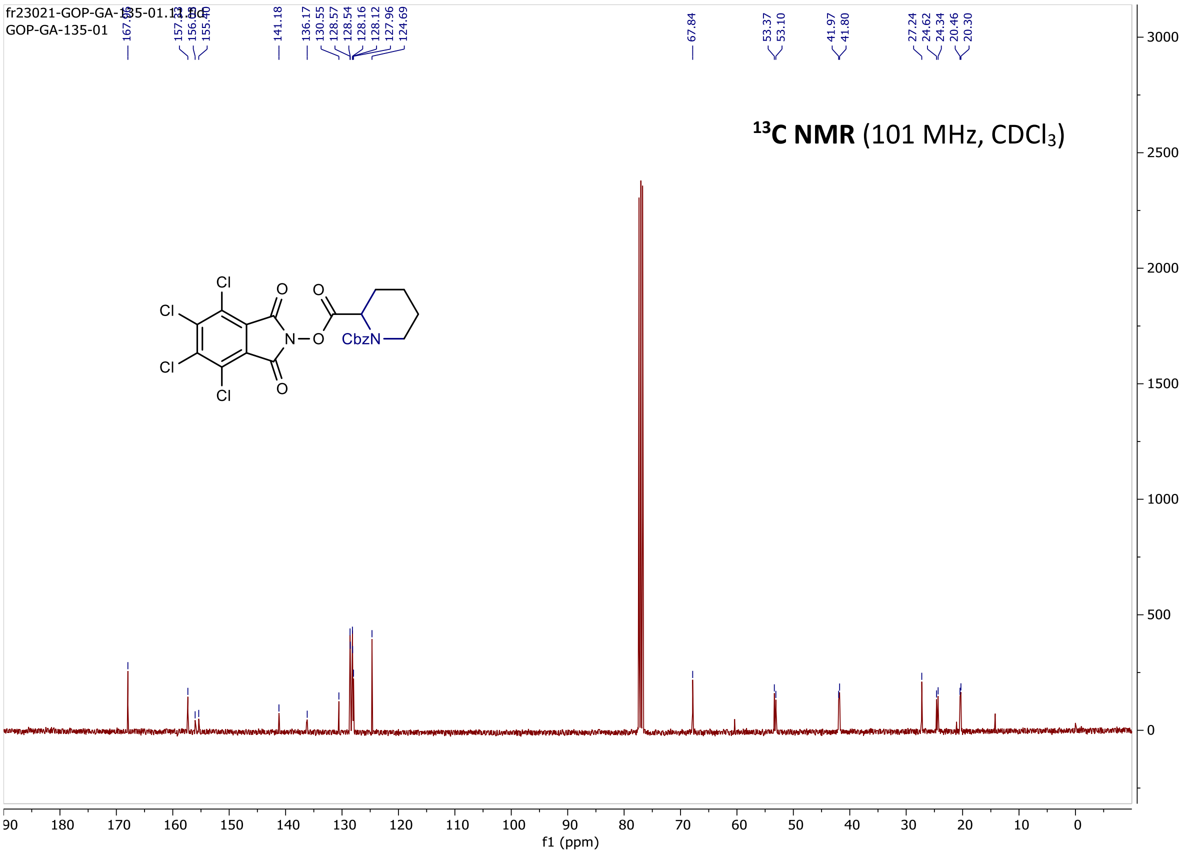

4,5,6,7-Tetrachloro-1,3-dioxoisoindolin-2-yl *N*-((benzyloxy)carbonyl)-*N*-methylvalinate (S10)

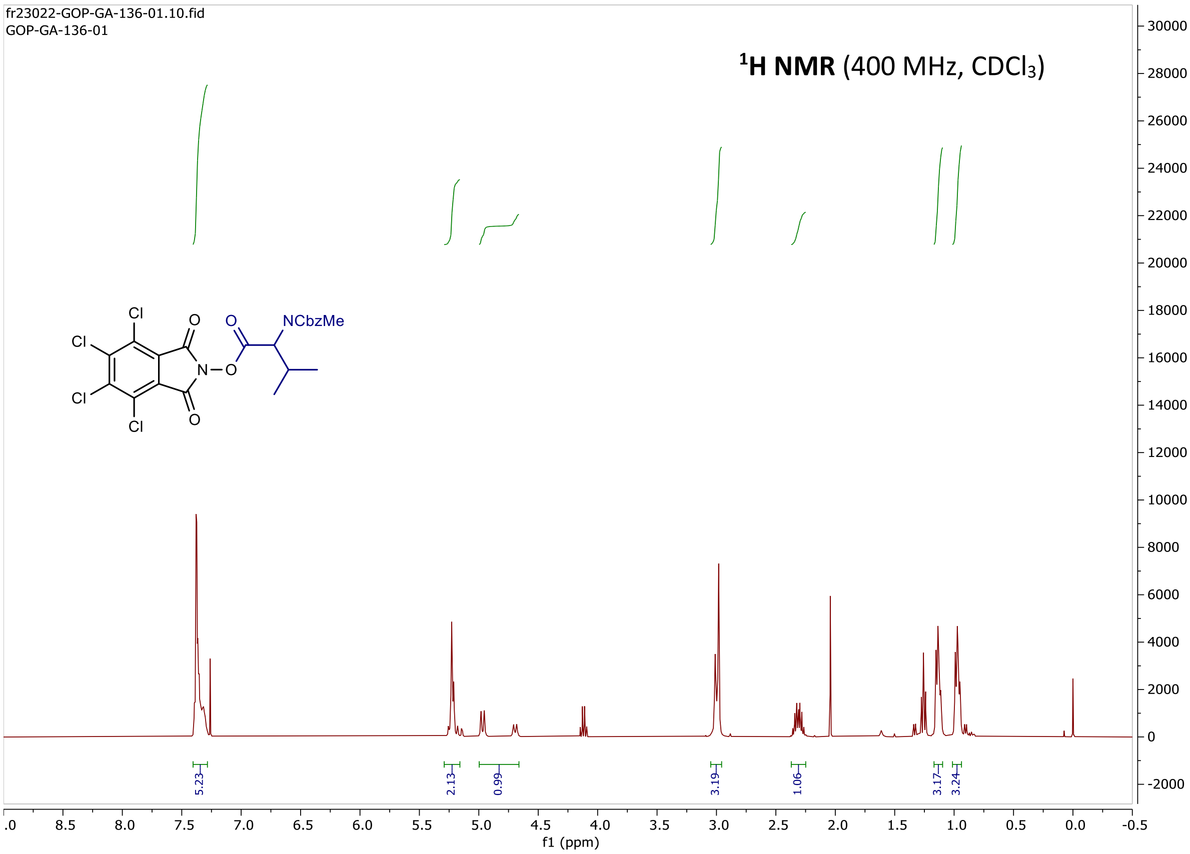

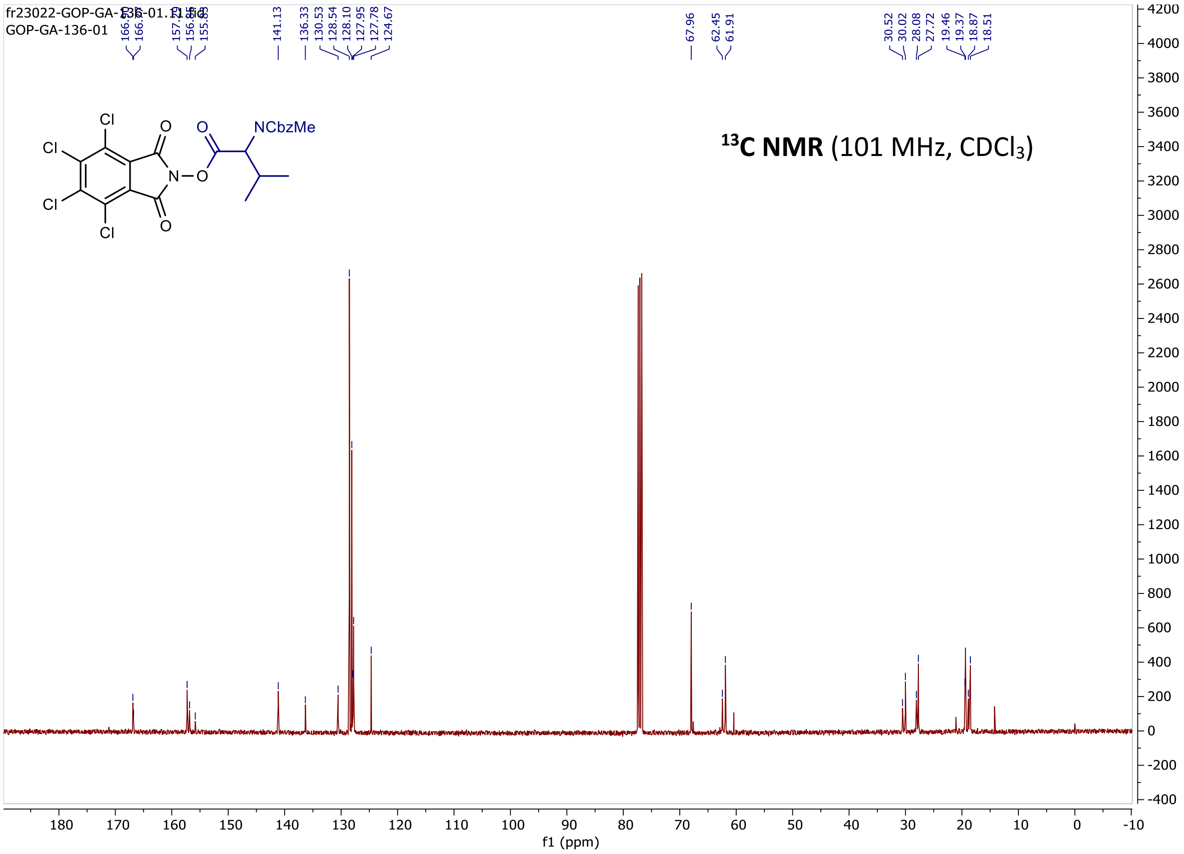

4,5,6,7-Tetrachloro-1,3-dioxoisoindolin-2-yl ((benzyloxy)carbonyl)phenylalaninate (S11)

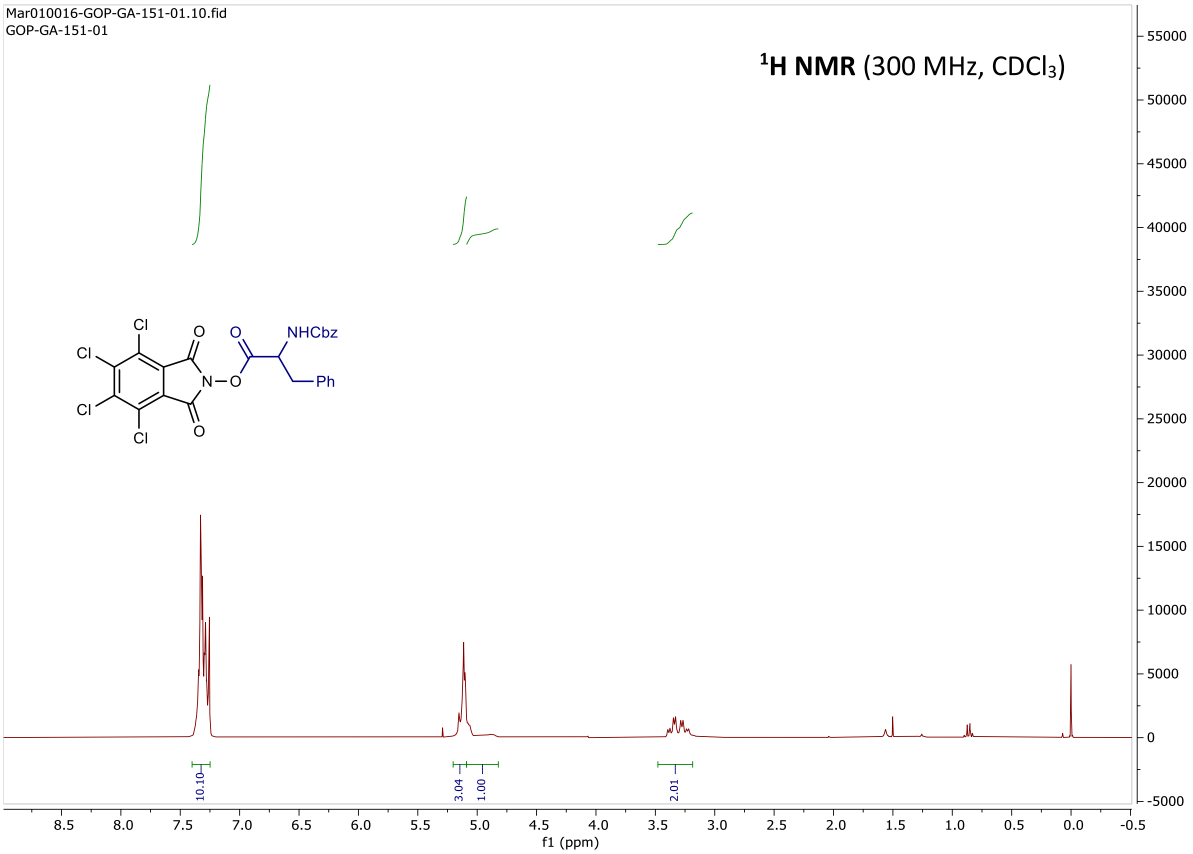

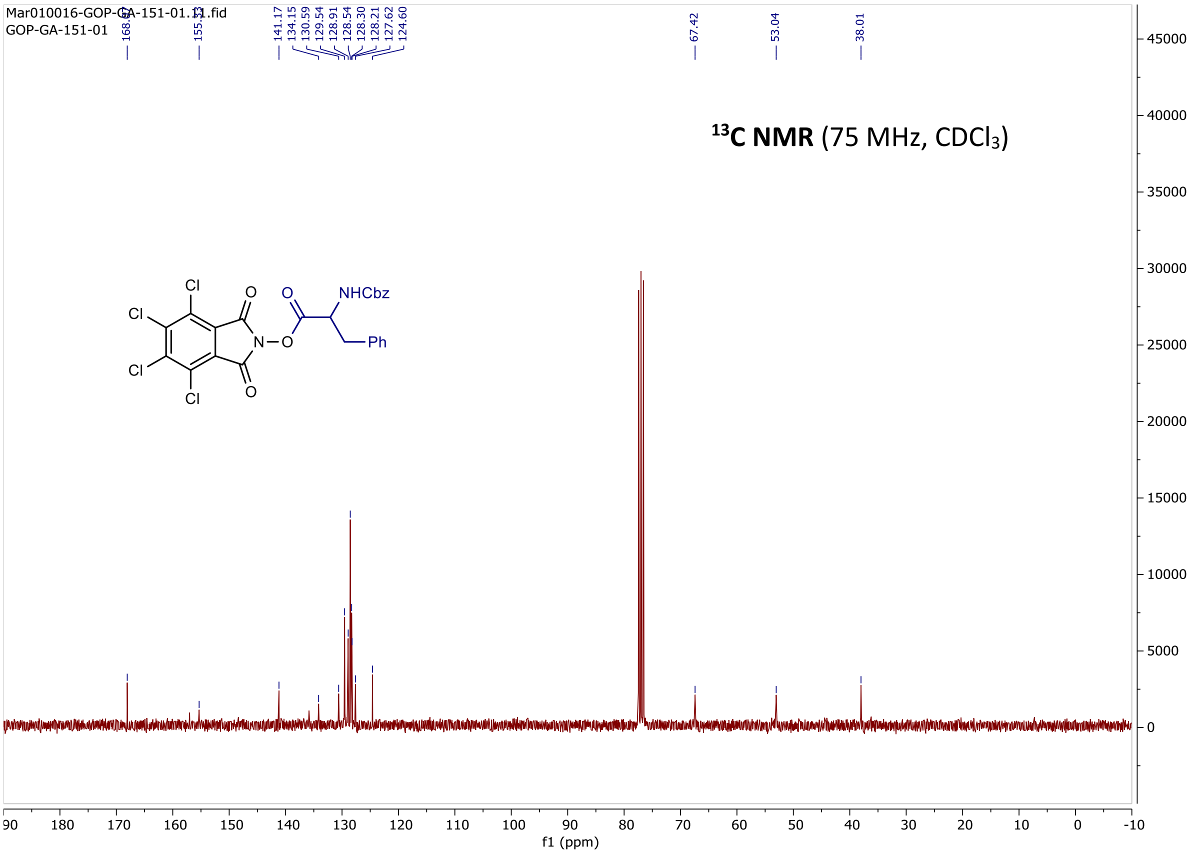

4,5,6,7-Tetrachloro-1,3-dioxoisoindolin-2-yl 2-phenylacetate (S12)

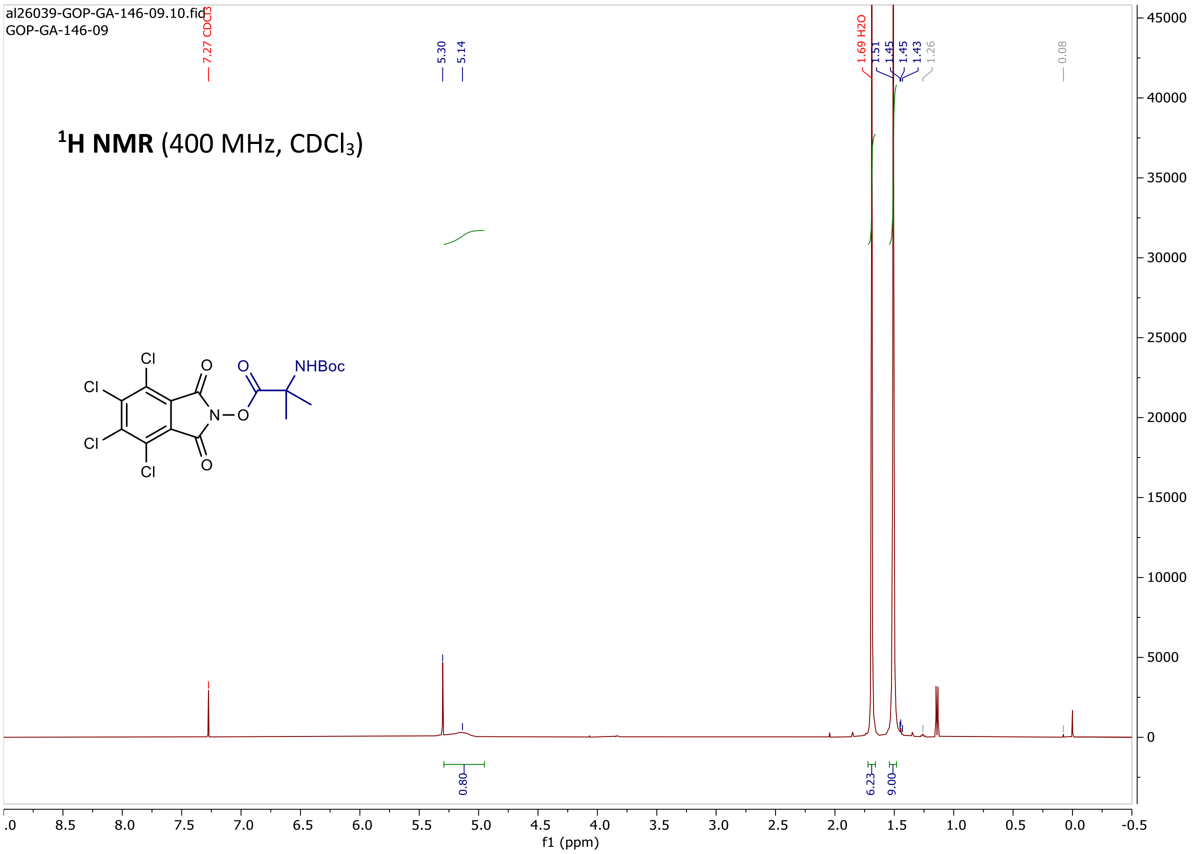

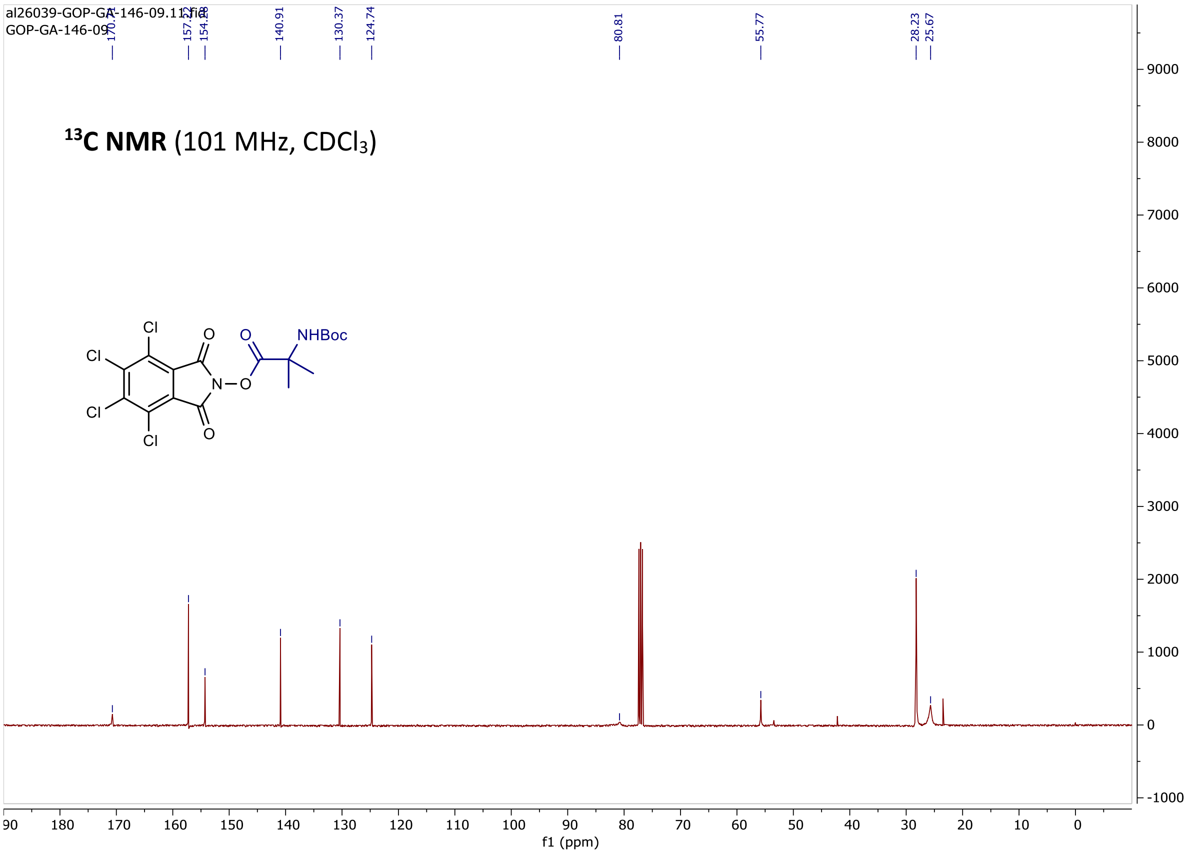

4,5,6,7-Tetrachloro-1,3-dioxoisoindolin-2-yl 2,2-diphenylacetate (S13)

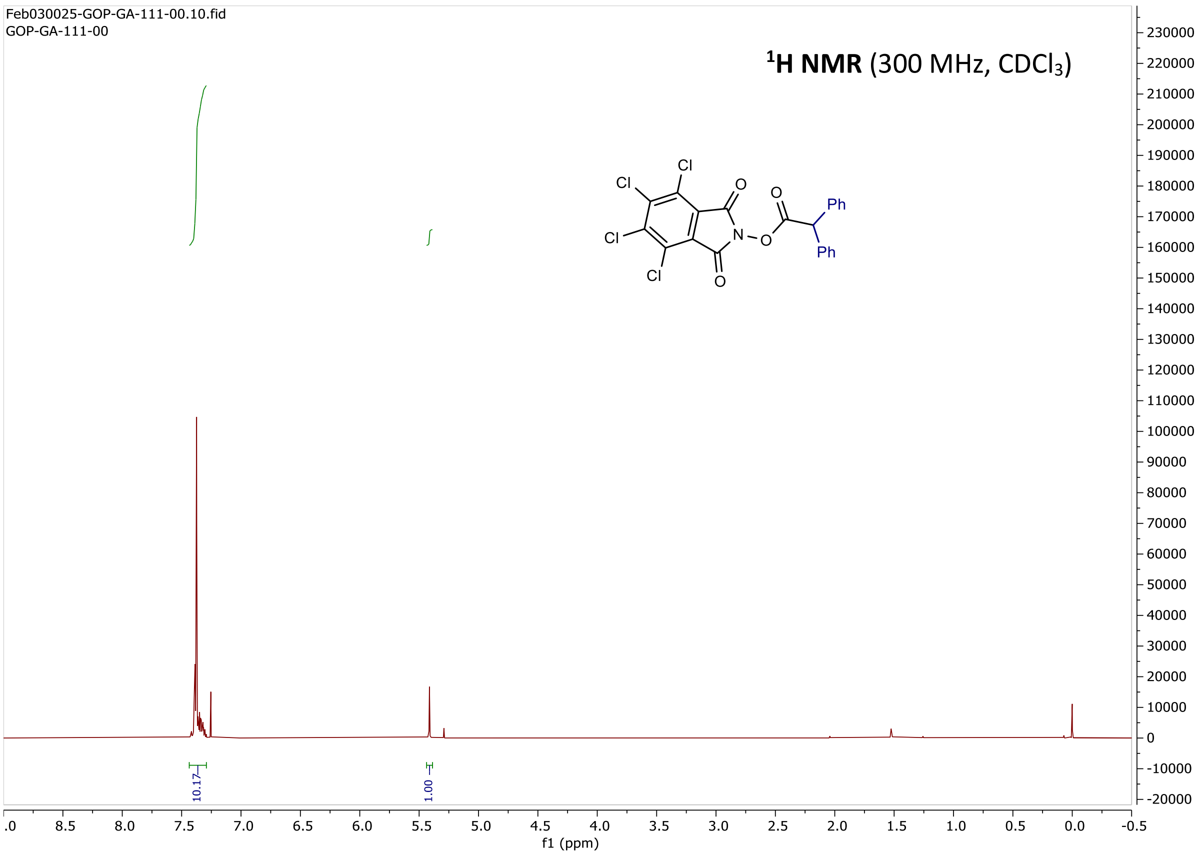

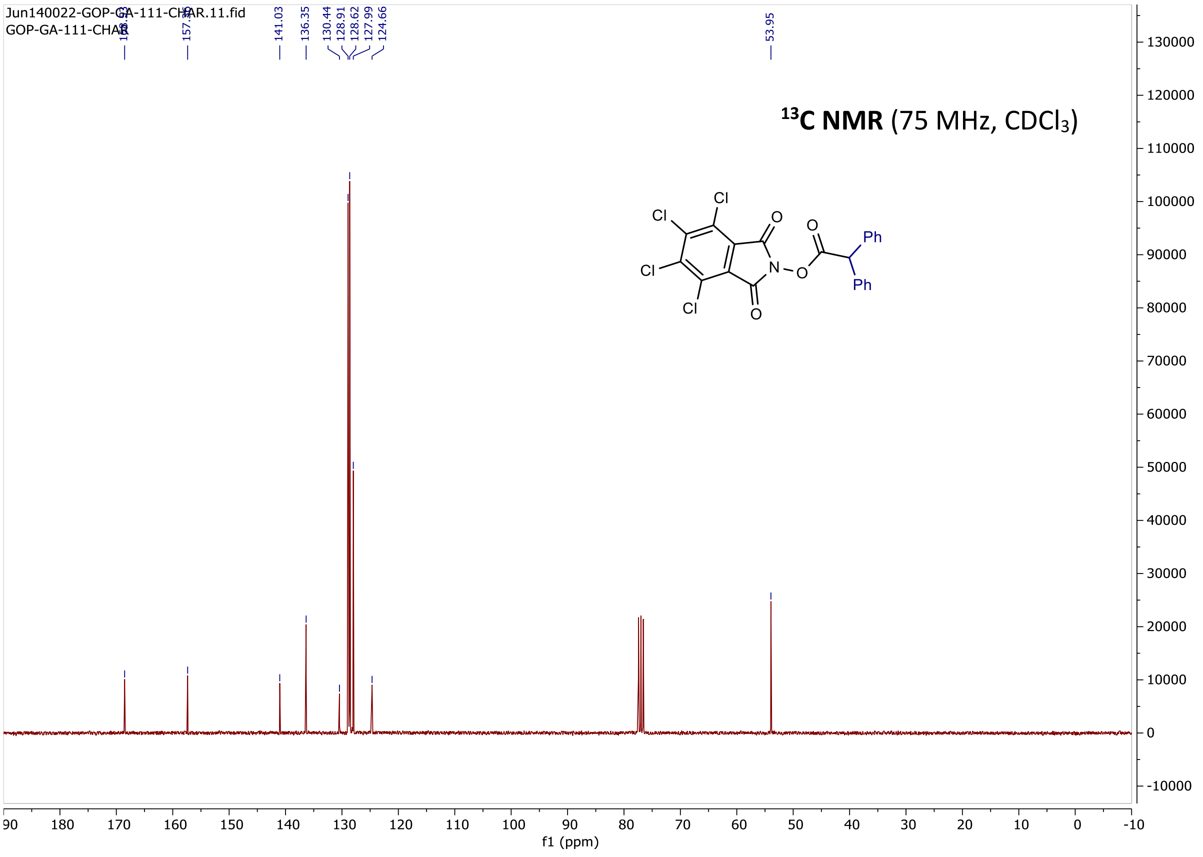

4,5,6,7-Tetrachloro-1,3-dioxoisoindolin-2-yl 2-(4-(4-chlorobenzoyl)phenoxy)-2-methylpropanoate (S15)

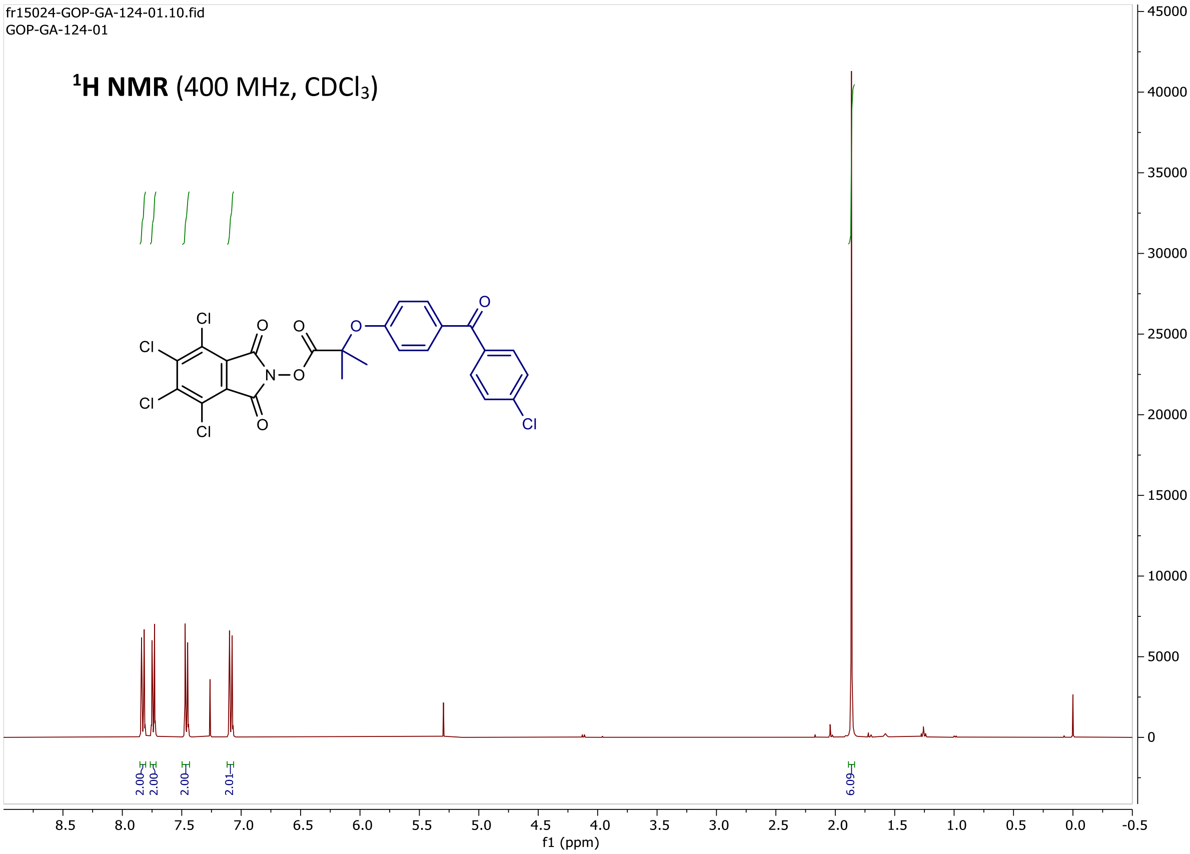

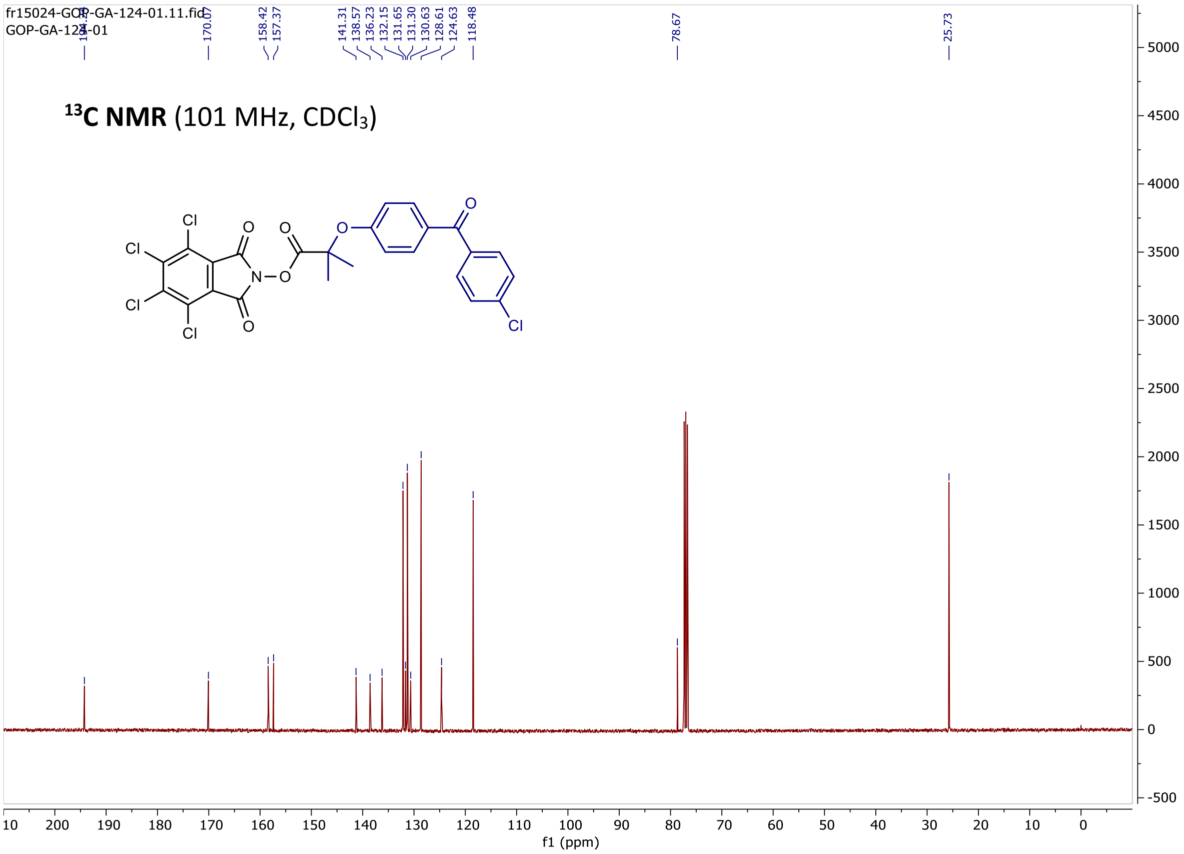

4,5,6,7-Tetrachloro-1,3-dioxoisoindolin-2-yl 2-(adamantan-1-yl)acetate (S16)

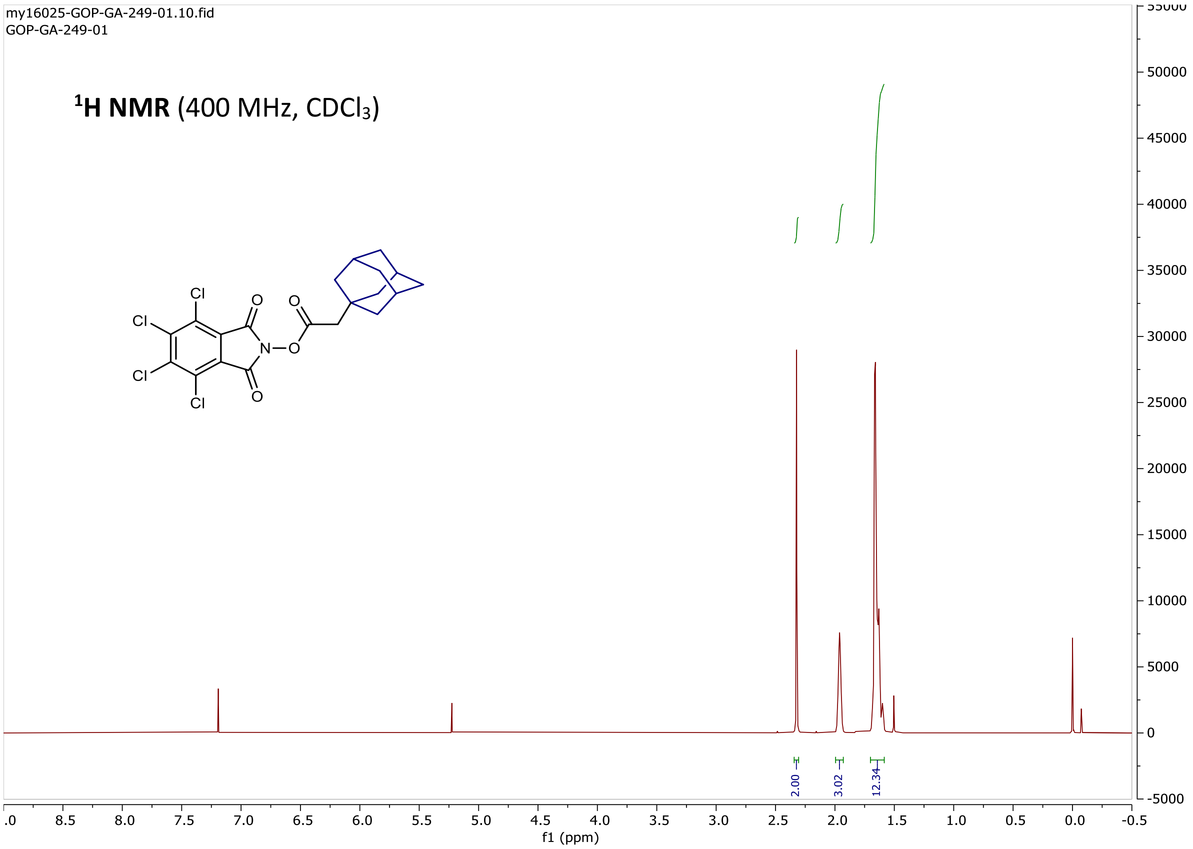

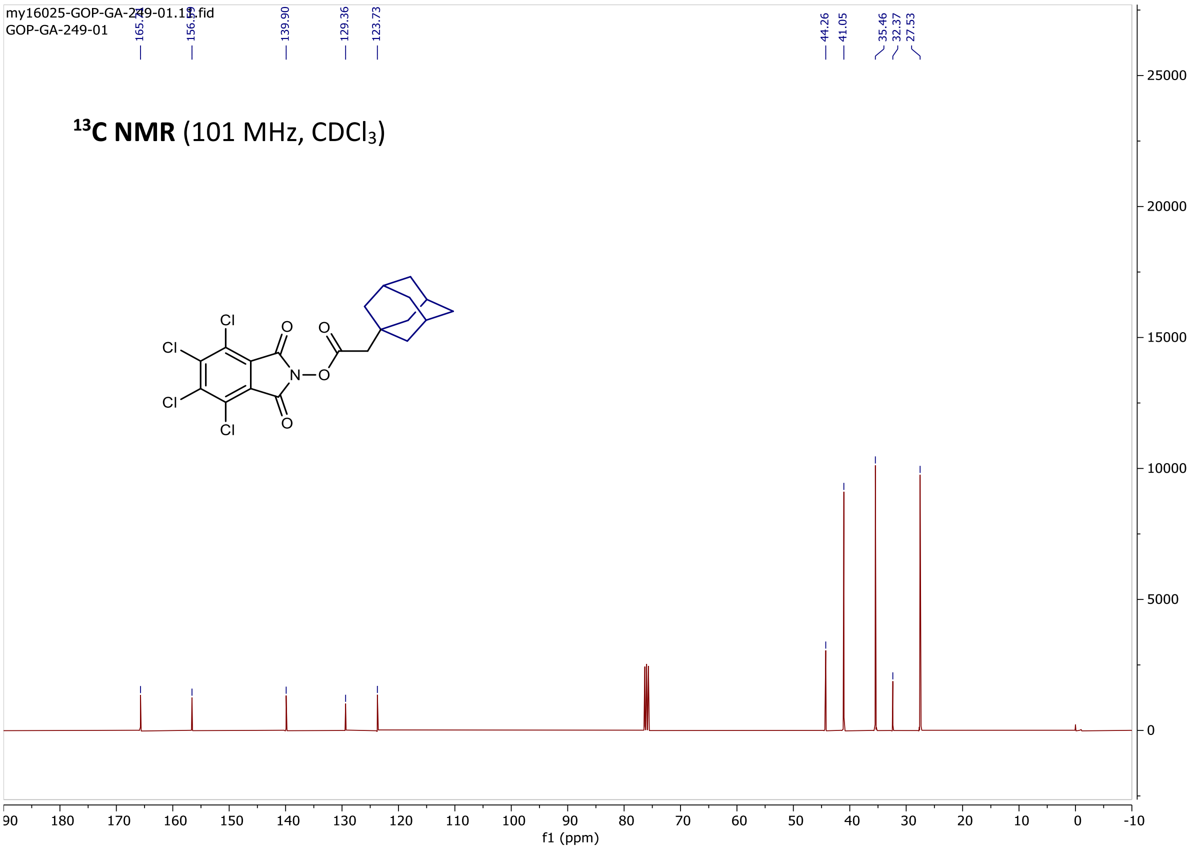

4,5,6,7-Tetrachloro-1,3-dioxoisoindolin-2-yl (*tert*-butoxycarbonyl)valinate (S17)

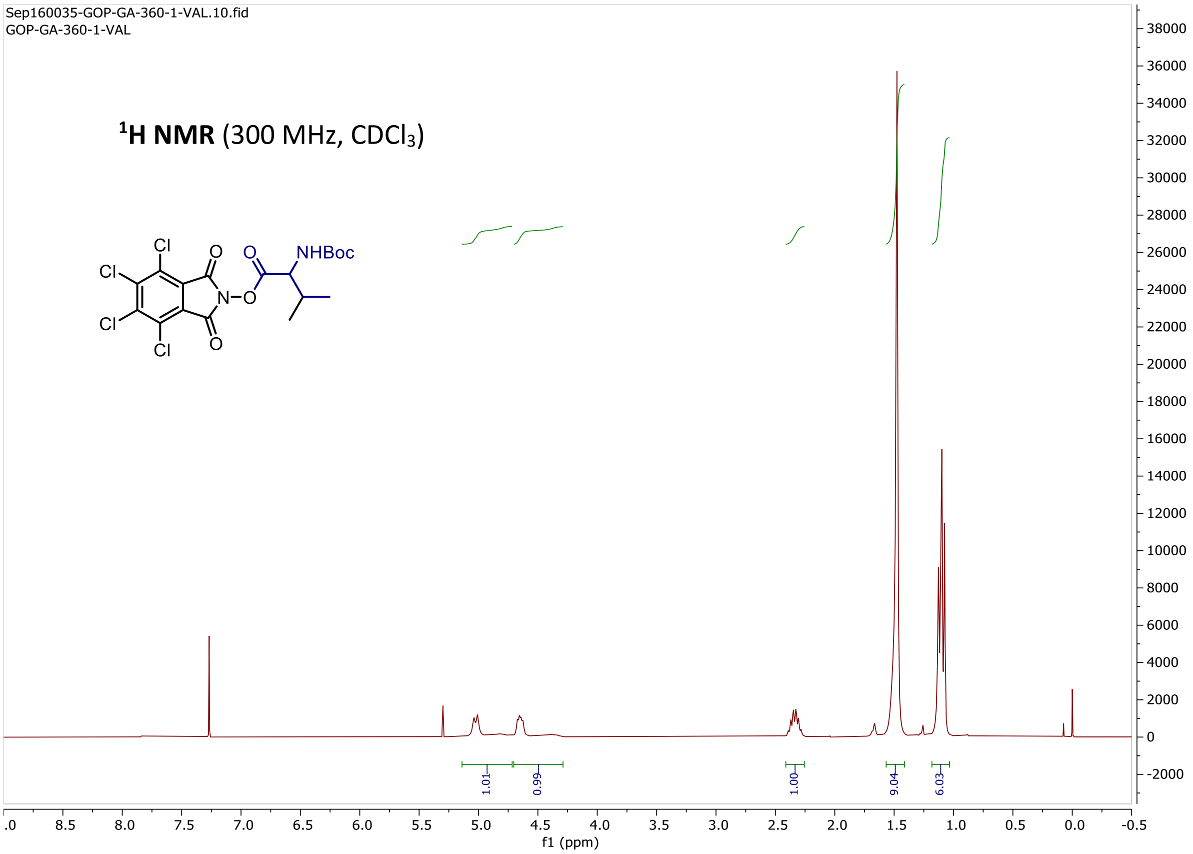

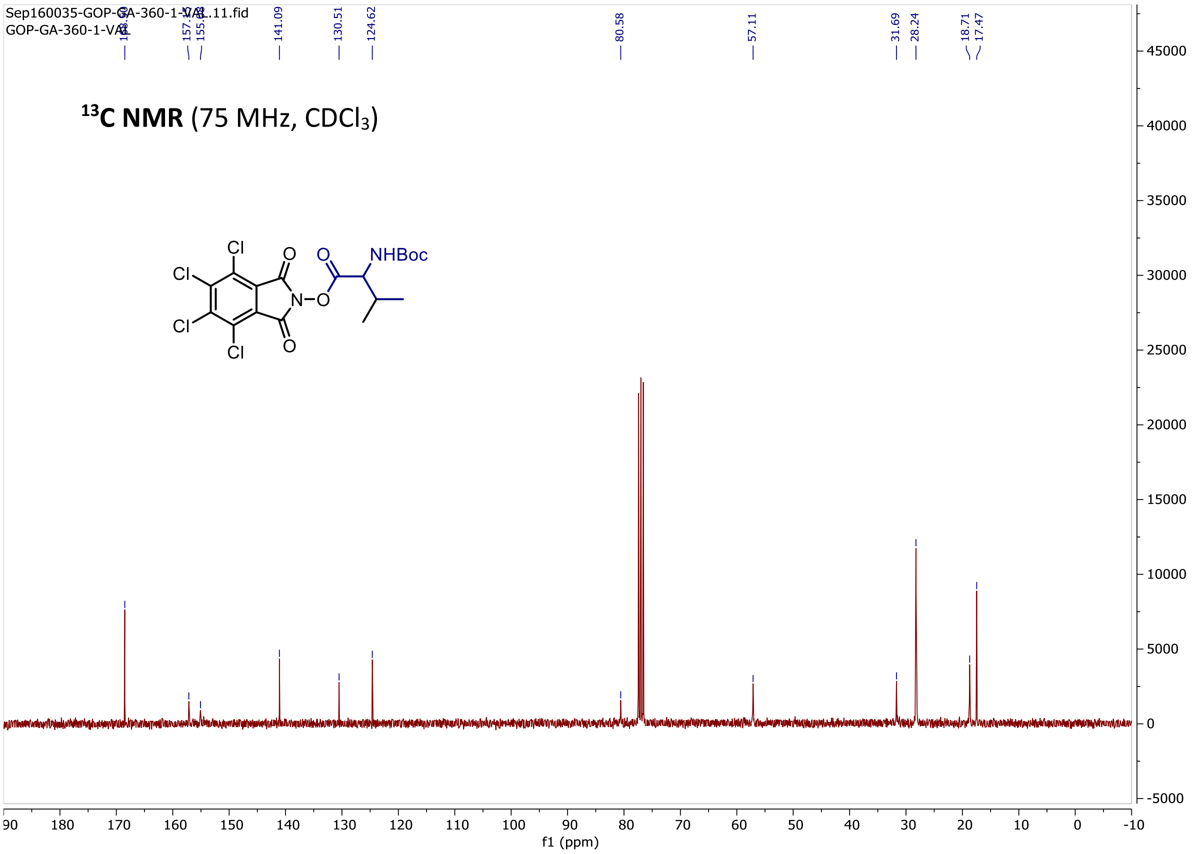

4,5,6,7-Tetrachloro-1,3-dioxoisoindolin-2-yl (*tert*-butoxycarbonyl)leucinate (S18)

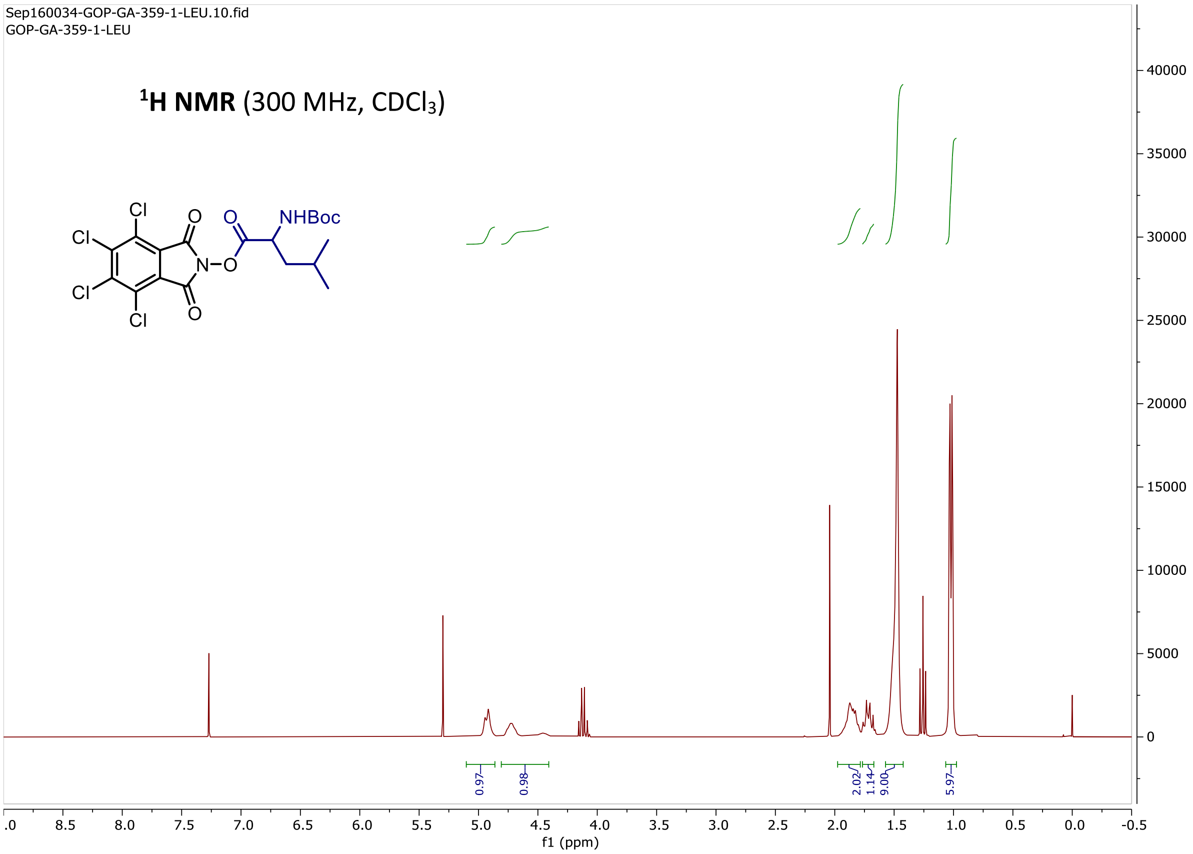

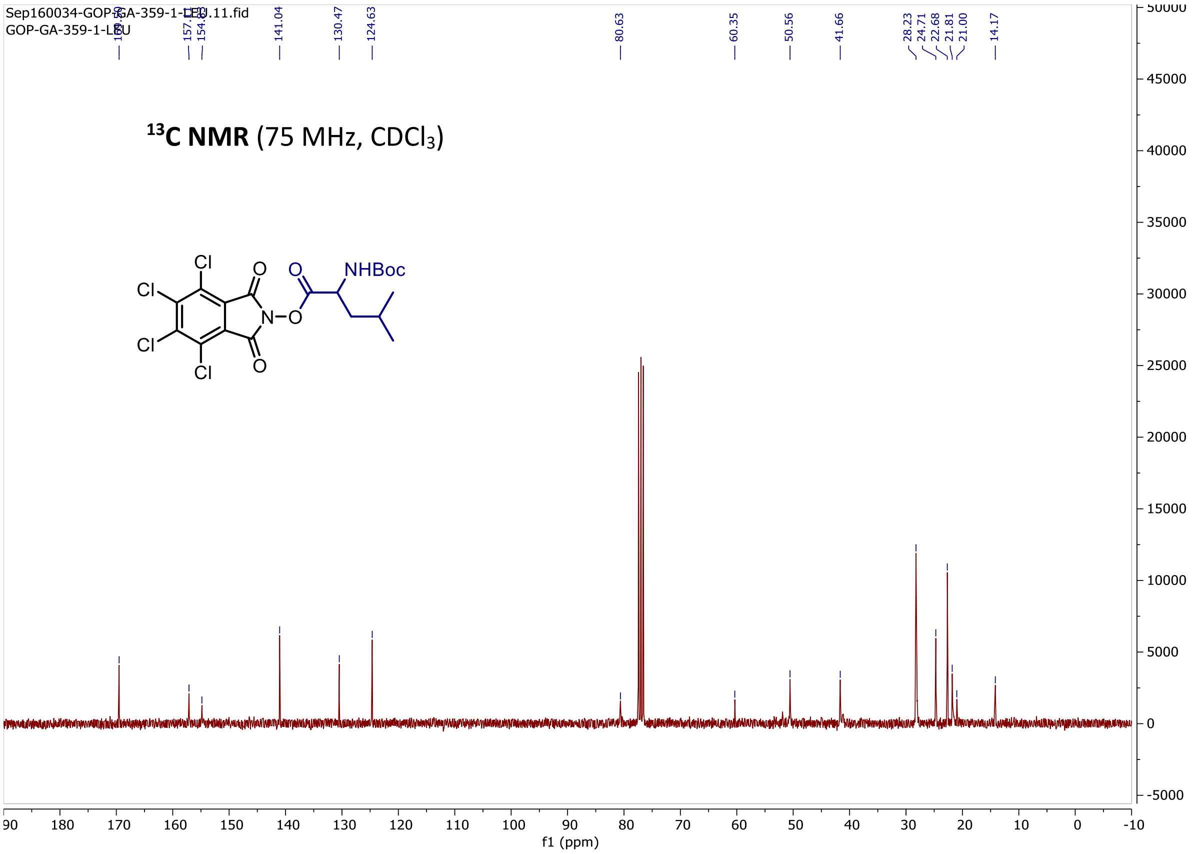

5-(*tert*-Butyl) 1-(4,5,6,7-tetrachloro-1,3-dioxoisoindolin-2-yl) (*tert*-butoxycarbonyl)glutamate (S19)

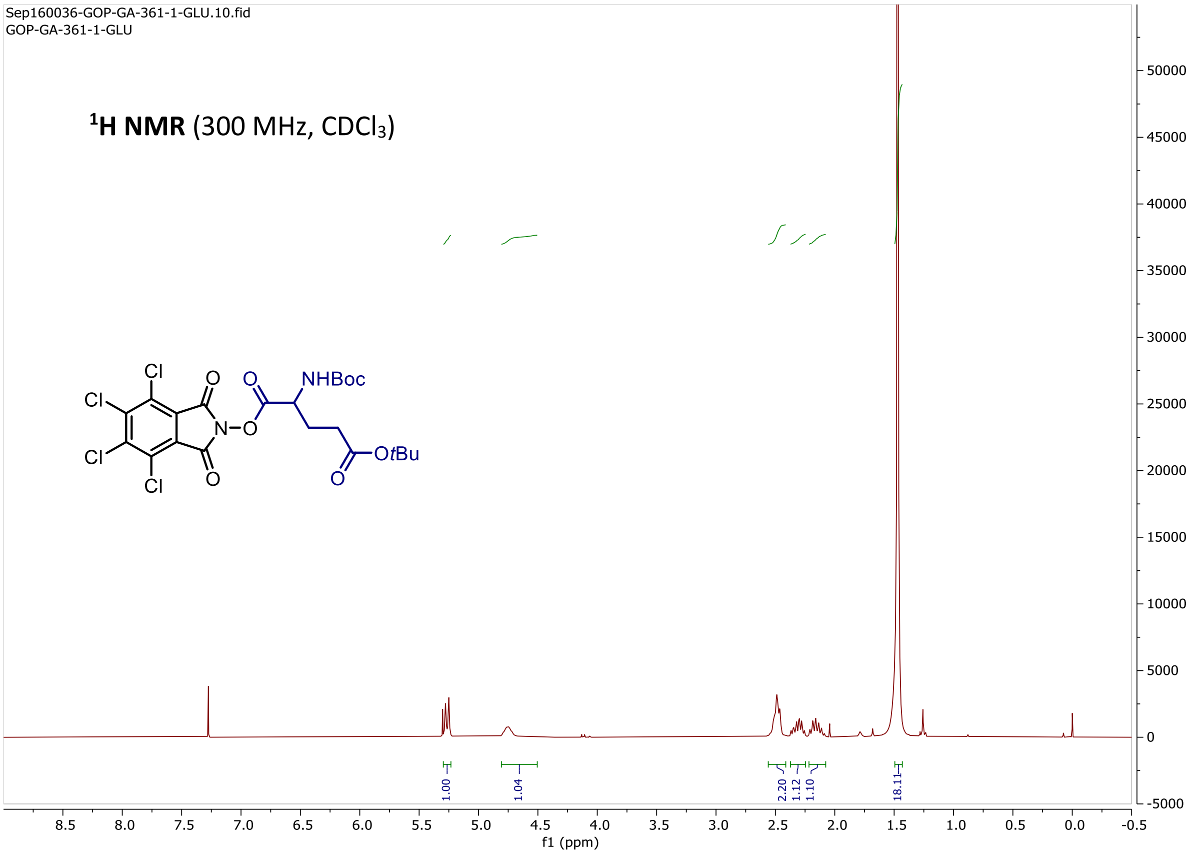

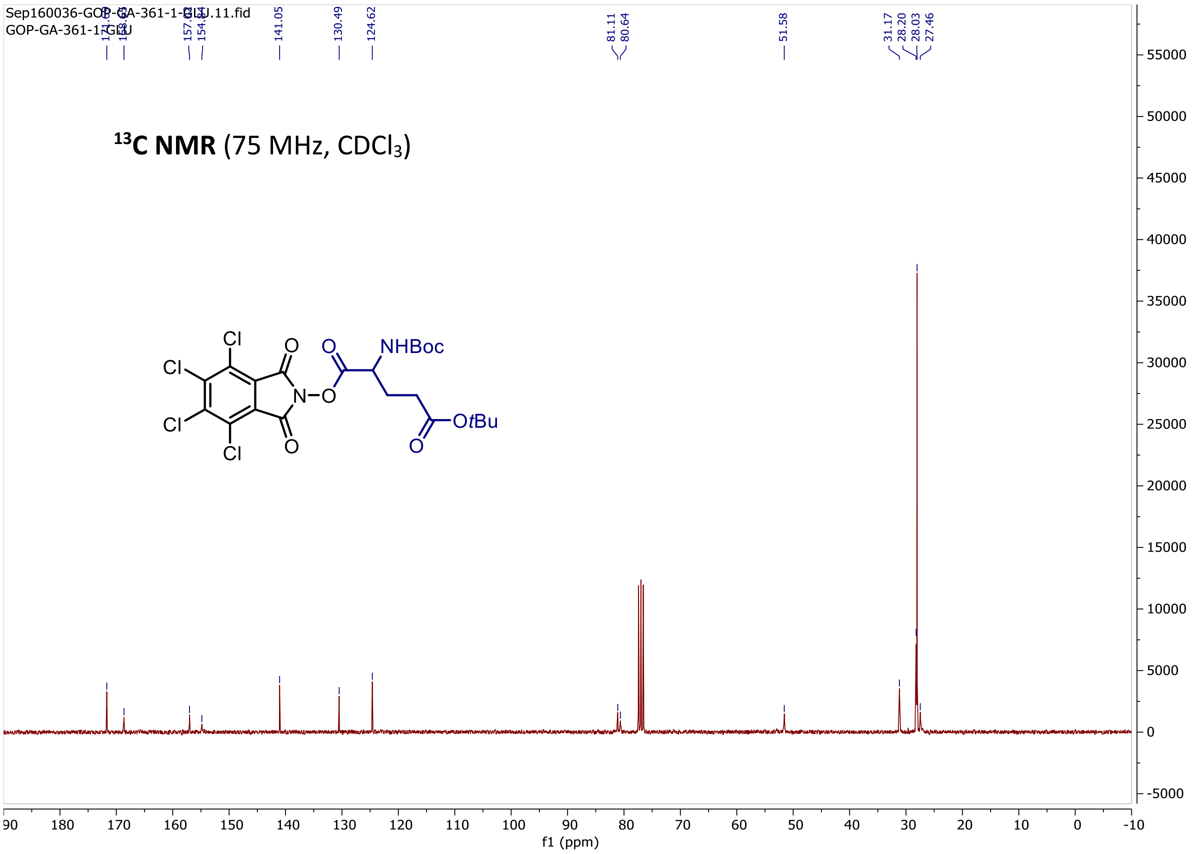

(*E*)-1-(2-Bromo-3-((*E*)-(cyclohexylimino)methyl)phenyl)-N-cyclohexylmethanimine (S3)

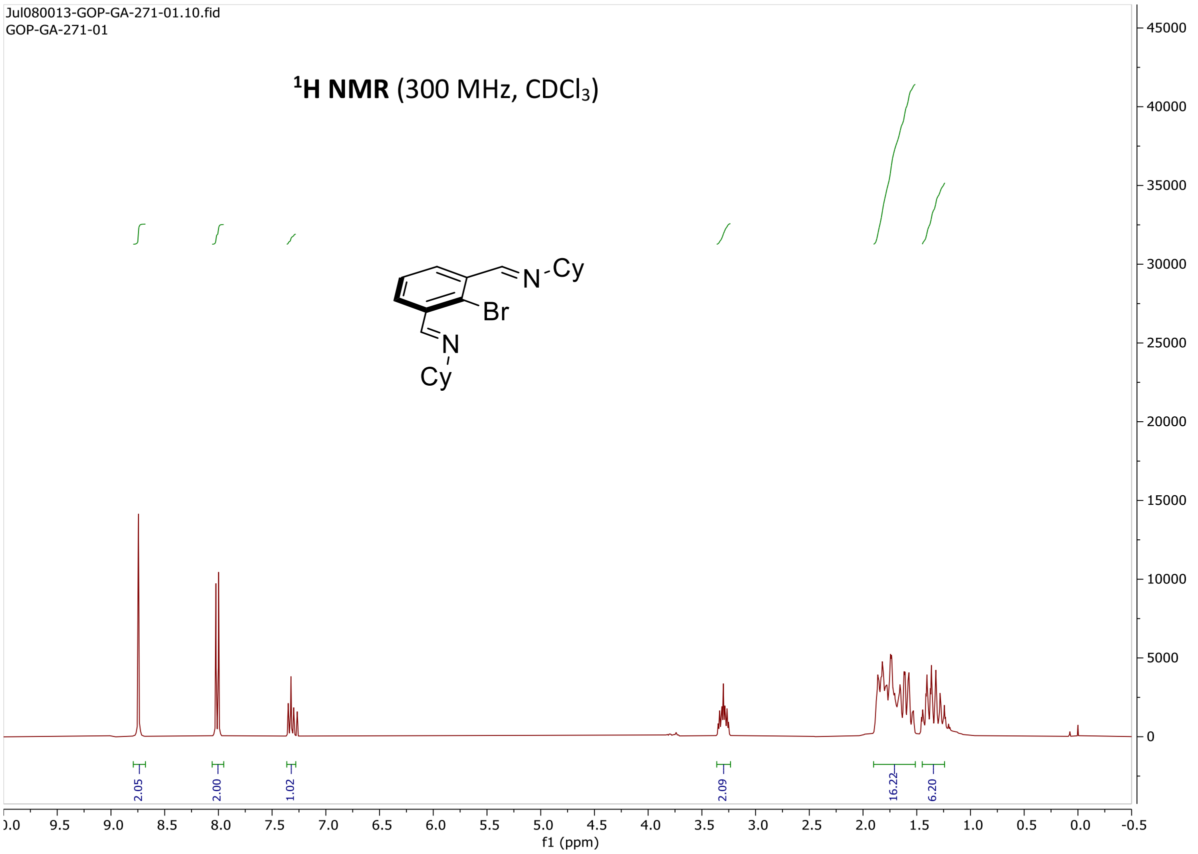

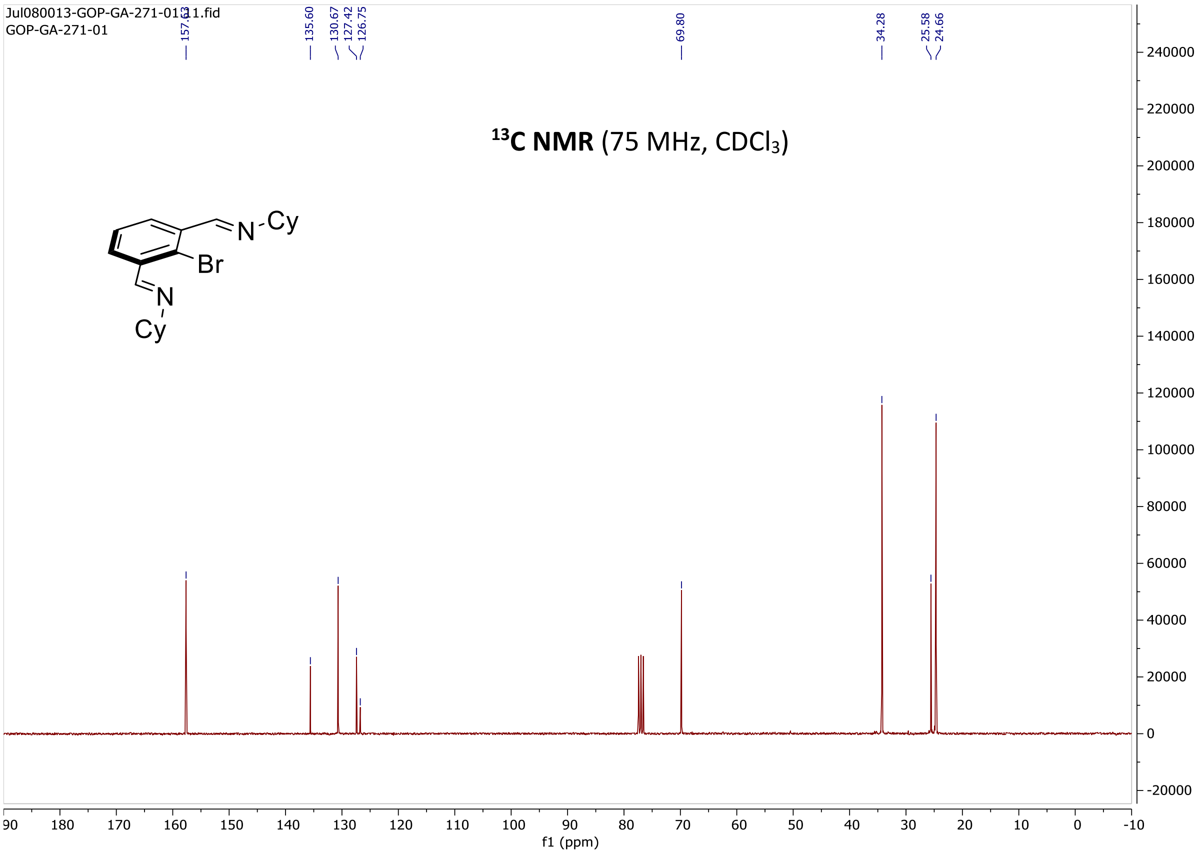

Bis-cyclohexyl dichlorobismuthine (S4)

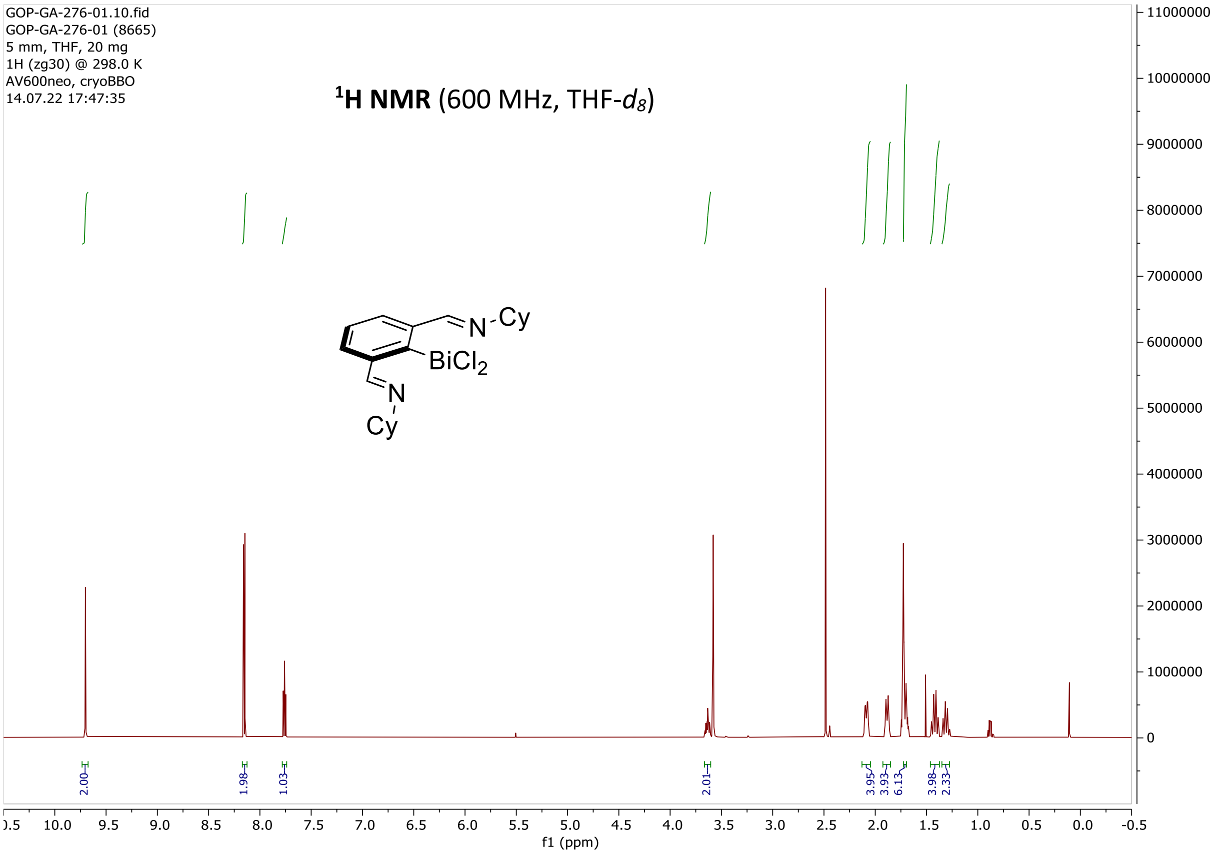

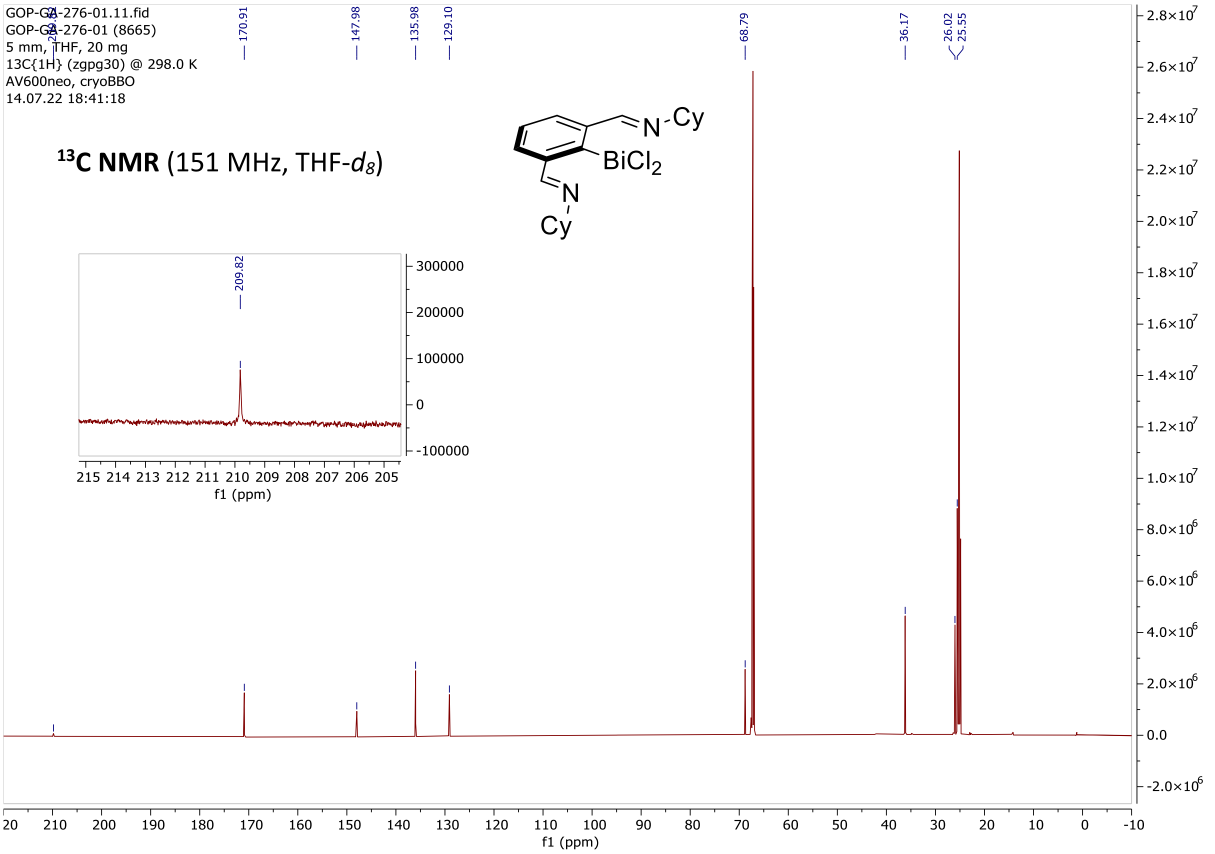

Bis-cyclohexyl bismuthinidine (S5)

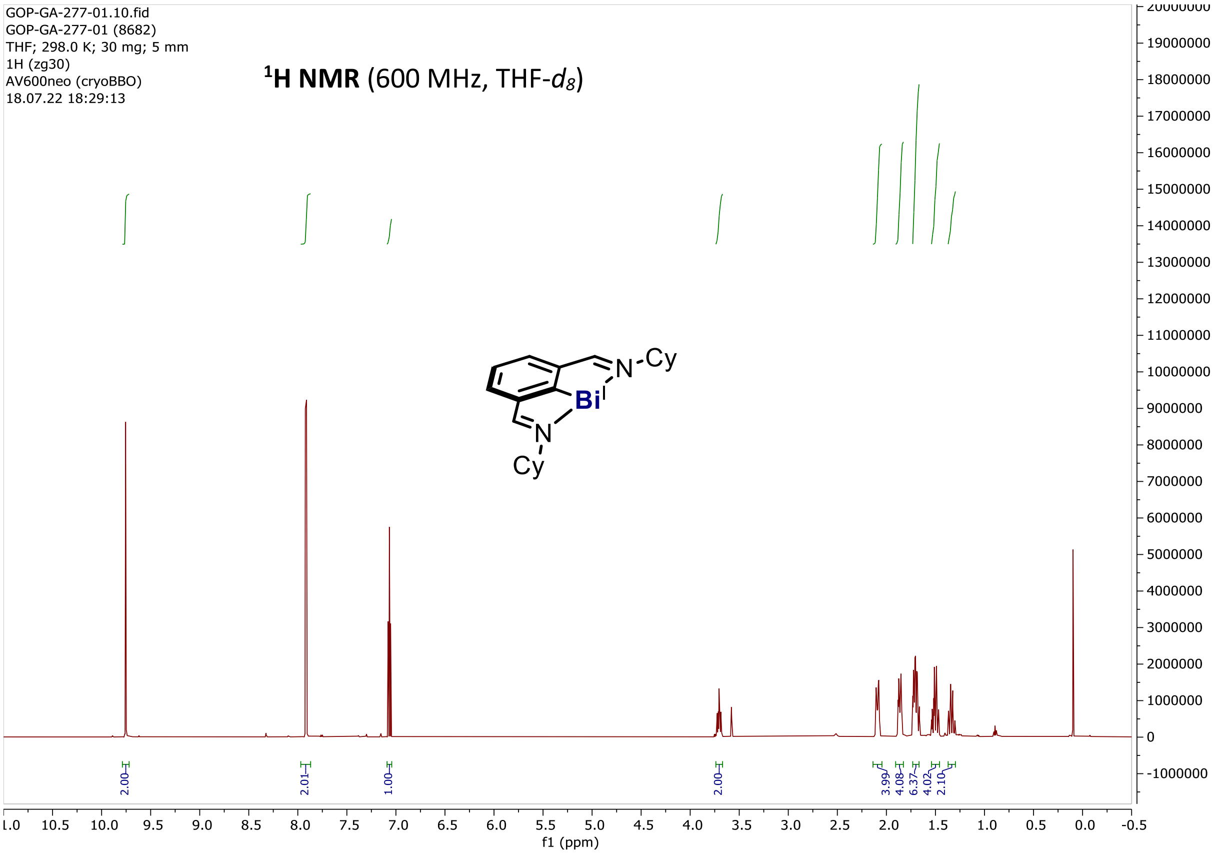

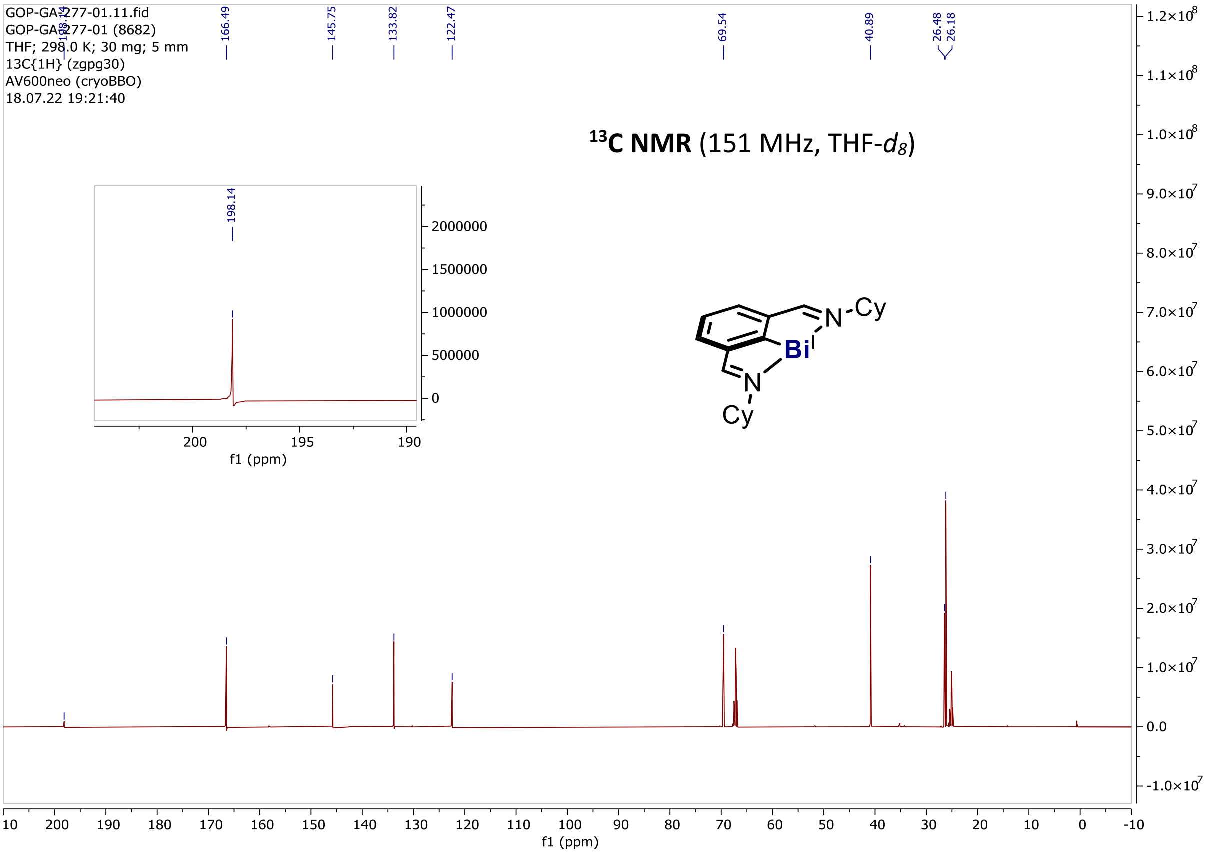

[(2,6-(*t*BuNCH)$_2$C$_6$H$_3$)Bi(benzyl)(chloride)] (5)

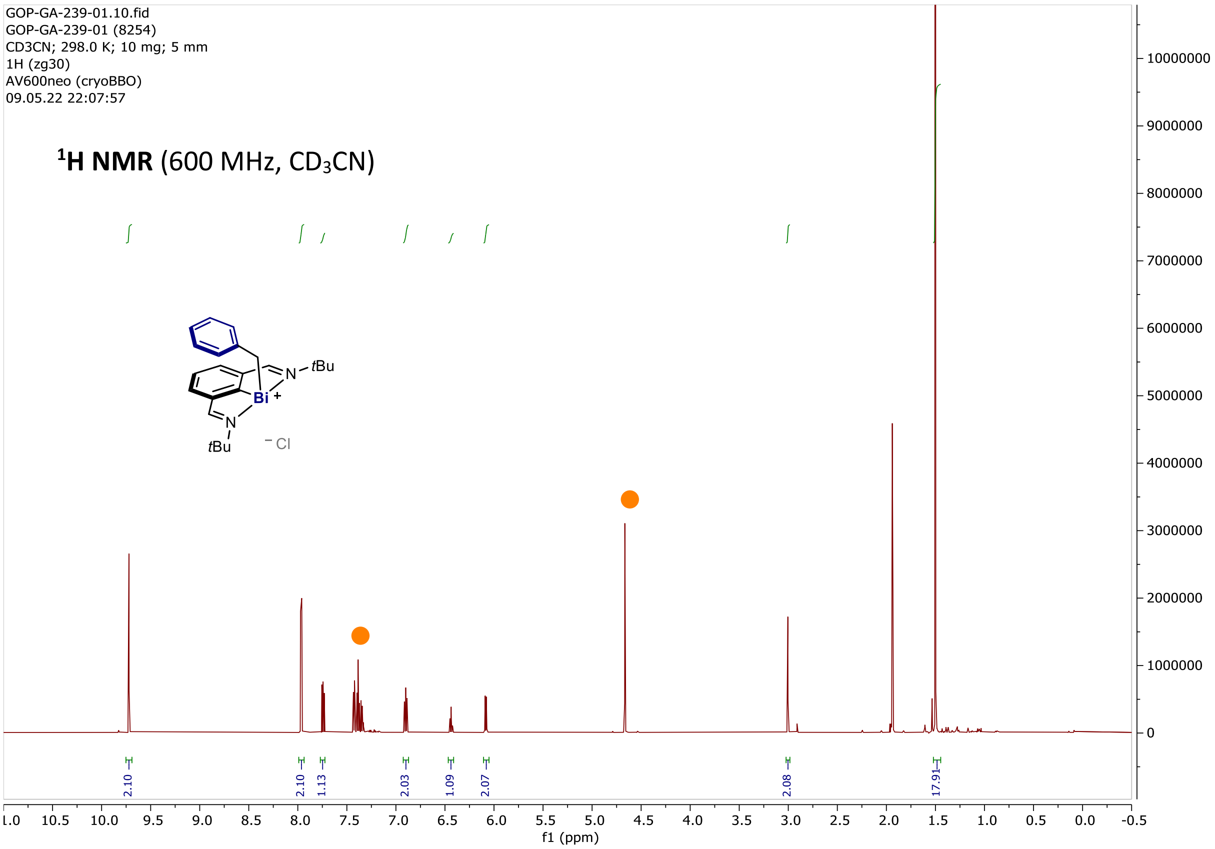

● Excess unreacted benzyl chloride

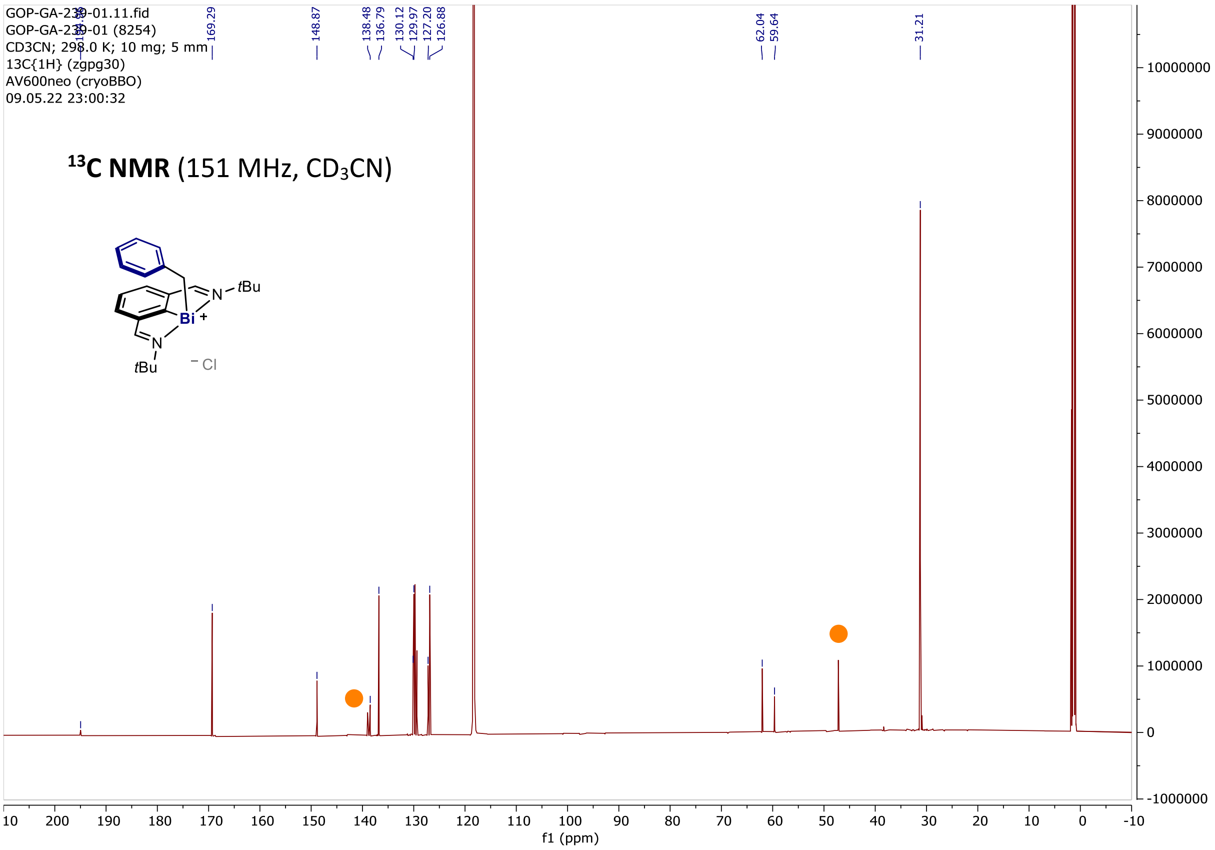

[(2,6-(*t*BuNCH)$_2$C$_6$H$_3$)Bi(benzyl)(bromide)] (6)

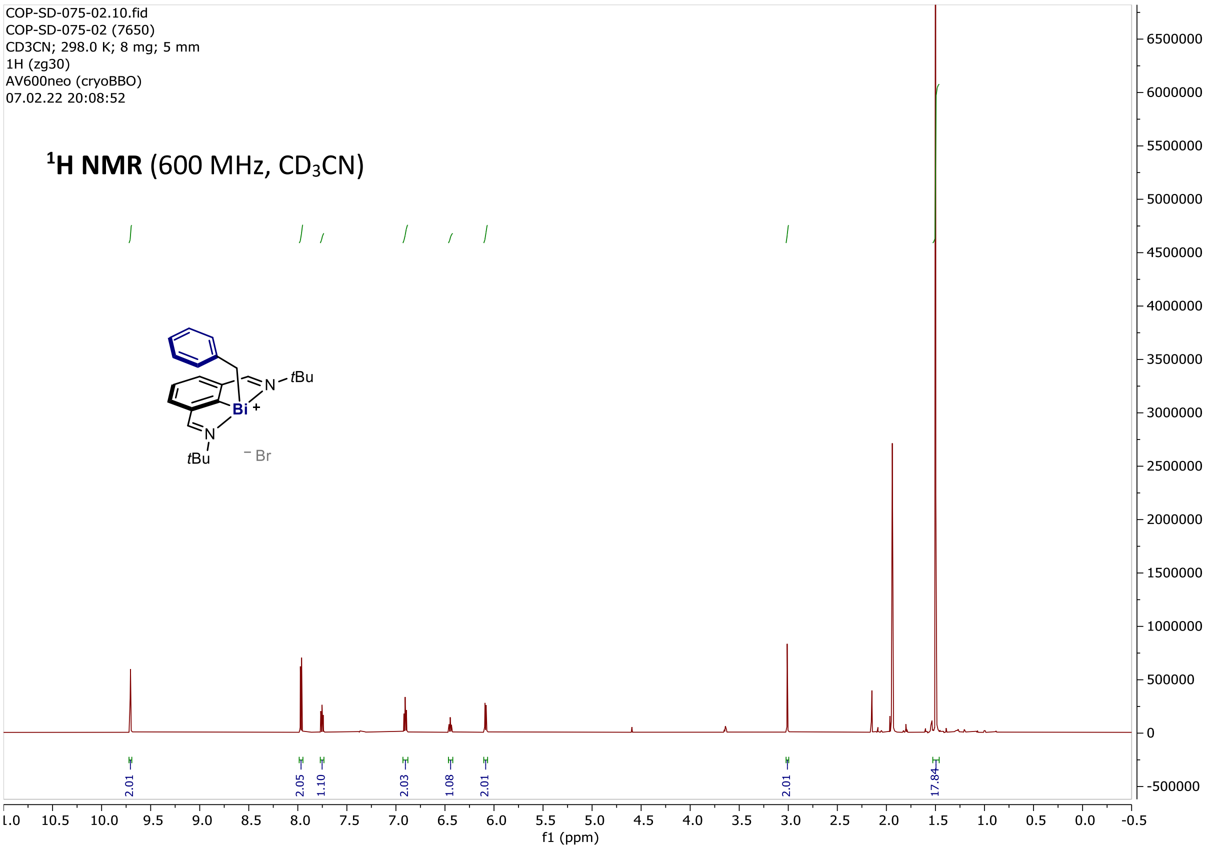

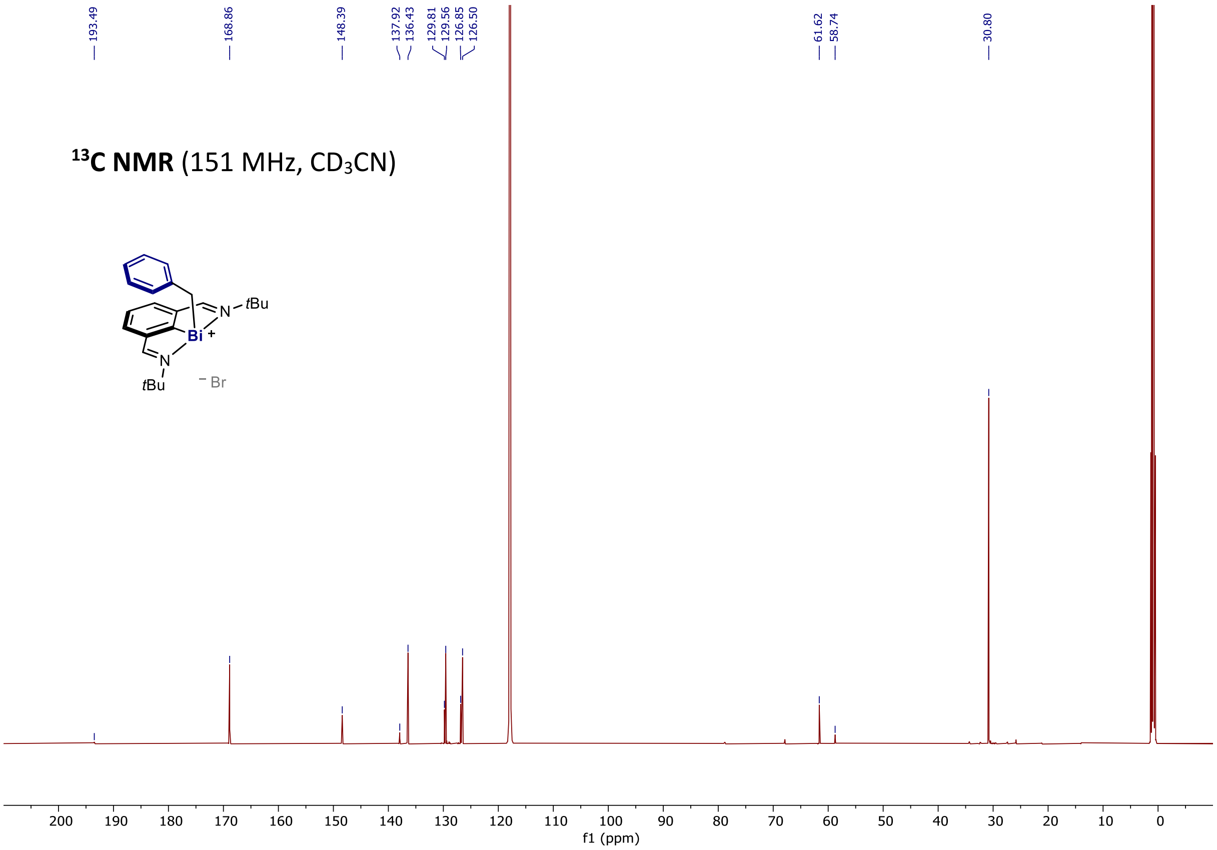

[(2,6-(*t*BuNCH)$_2$C$_6$H$_3$)Bi(benzyl)(iodide)] (7)

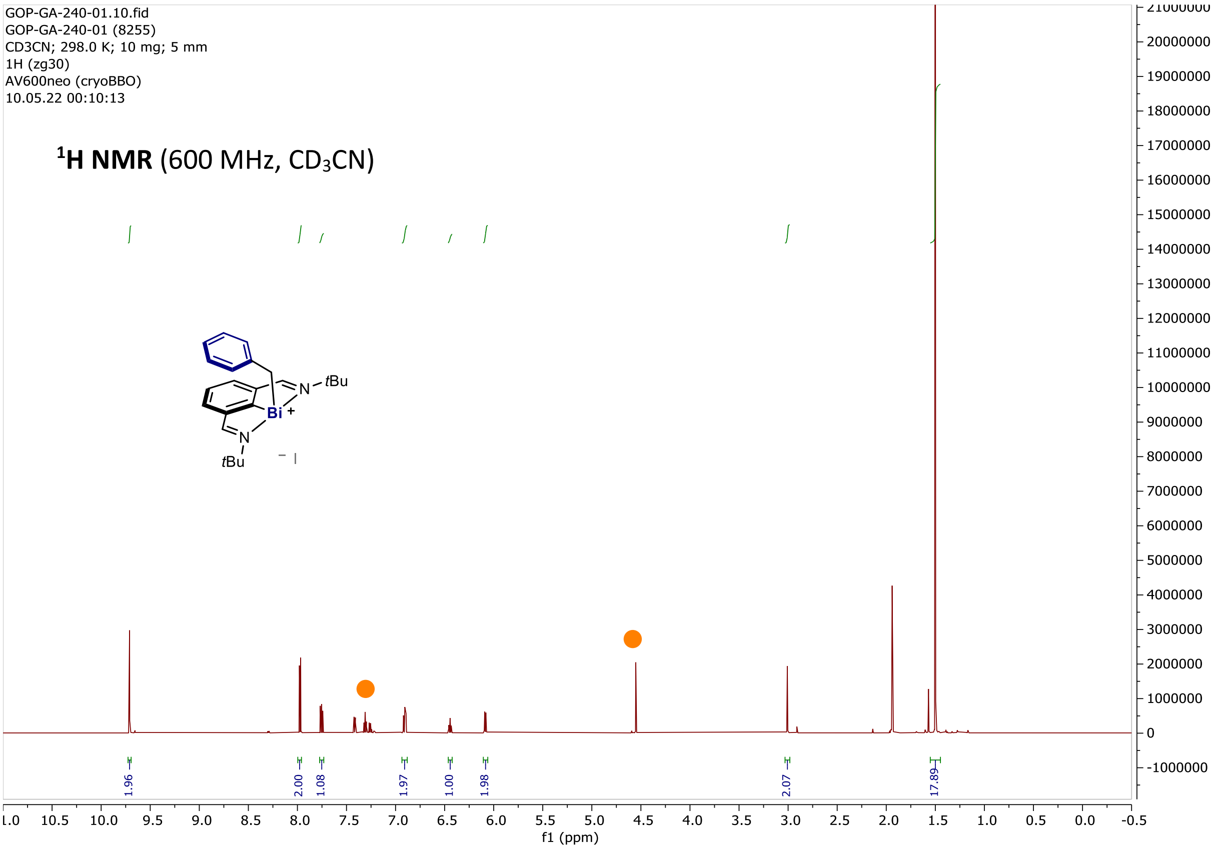

● Excess unreacted benzyl iodide

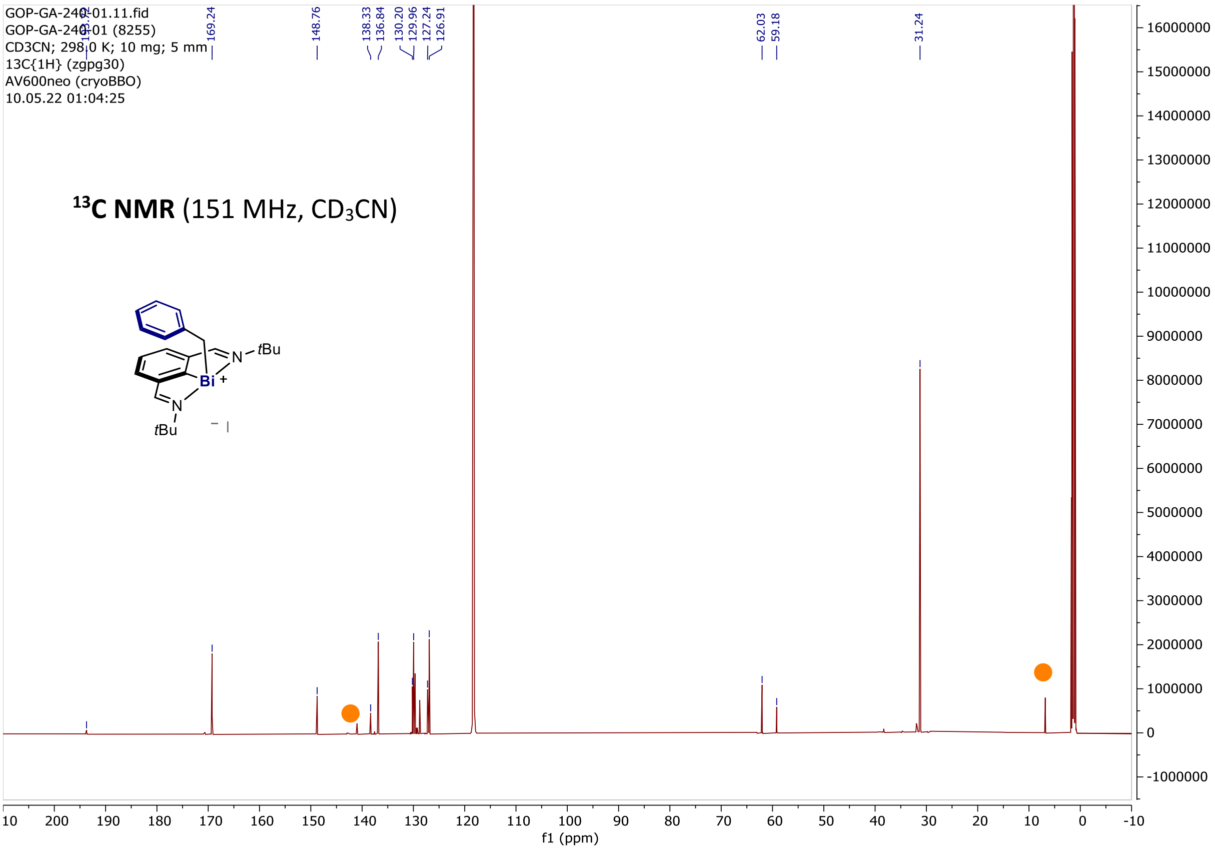

[(2,6-(tBuNCH)$_2$C$_6$H$_3$)Bi(benzyl)(mesylate)] (**8**)

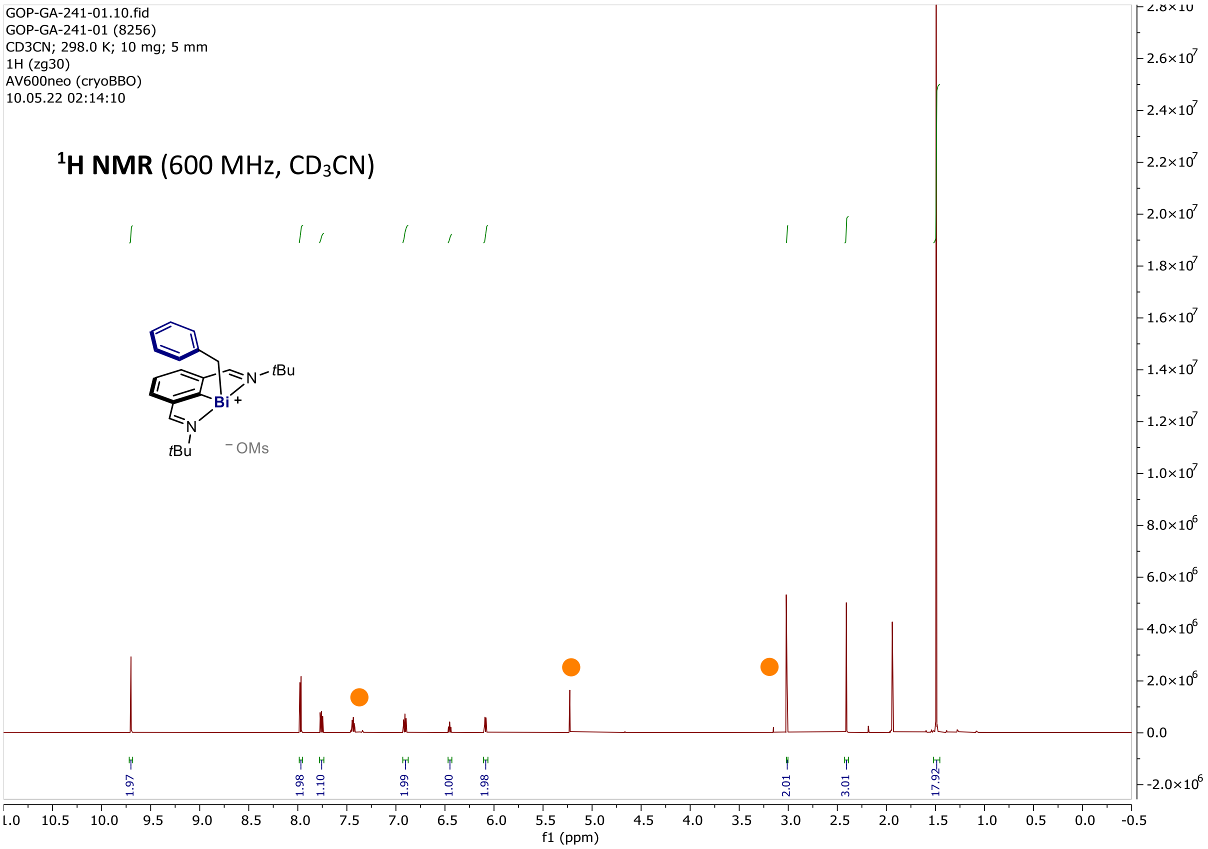

● Excess unreacted benzyl mesylate

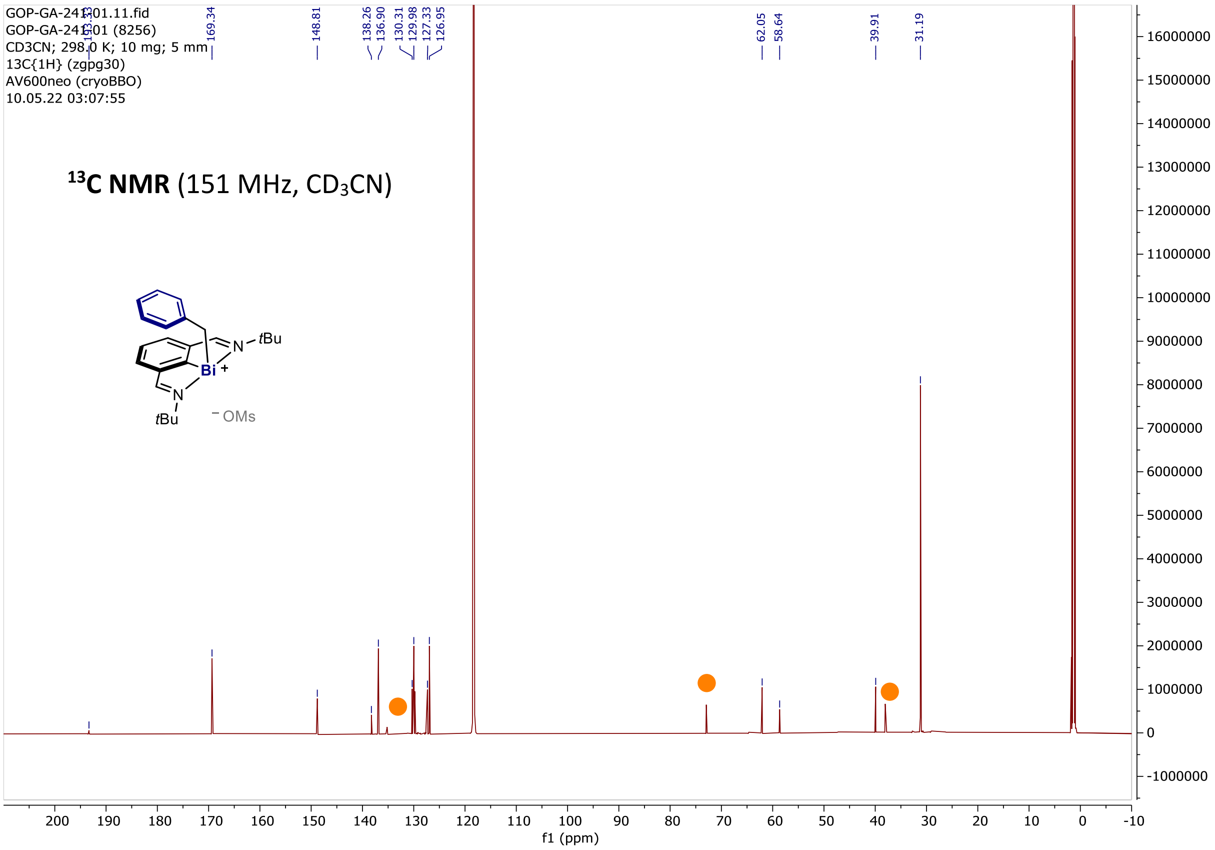

[(2,6-(*t*BuNCH)$_2$C$_6$H$_3$)Bi(benzyl)(tetrachlorophthalimide)] (9)

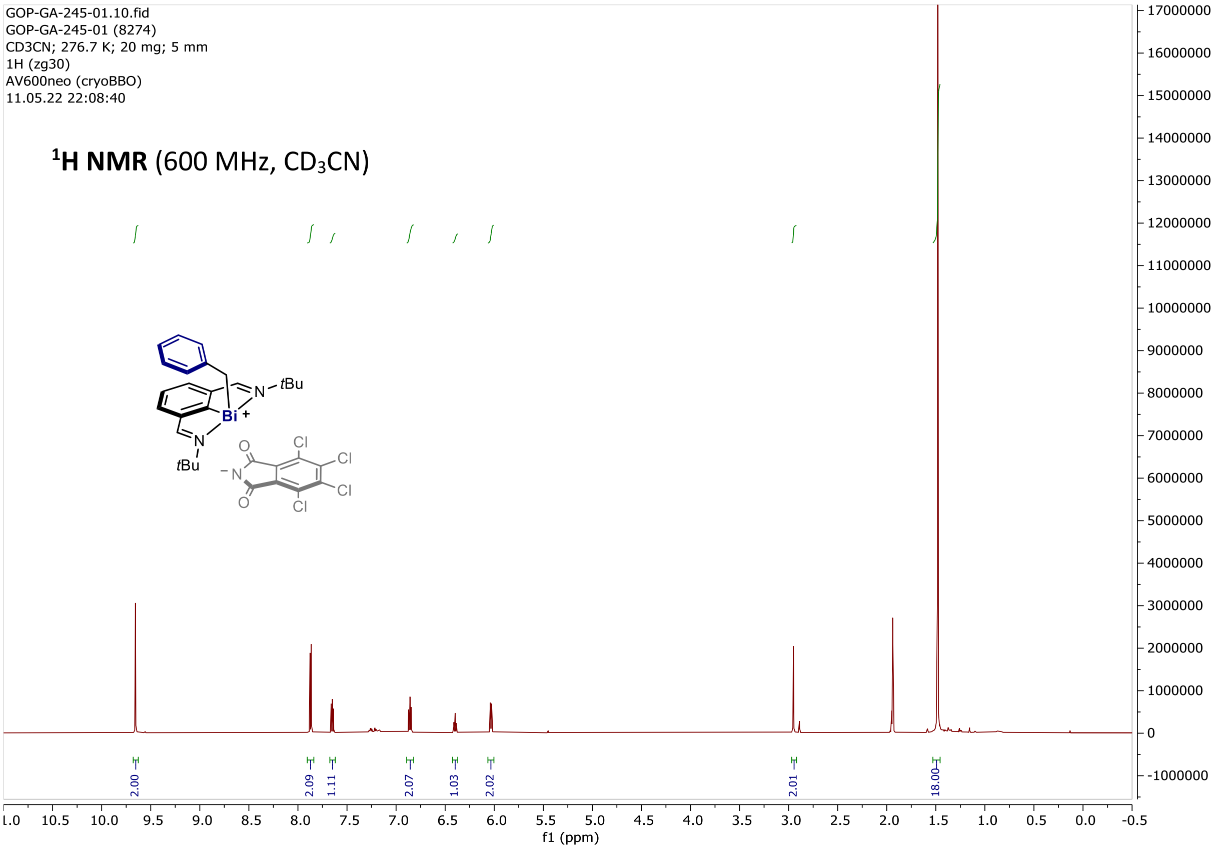

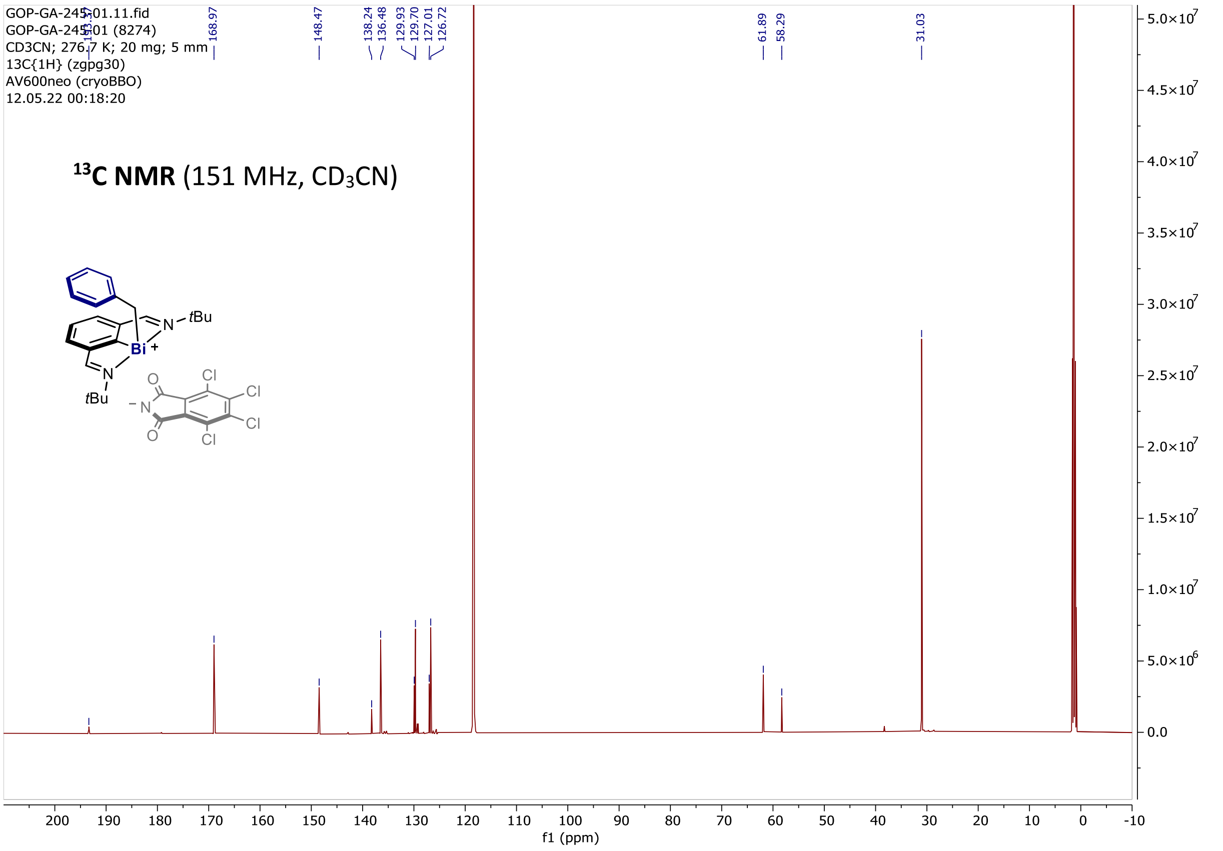

[(2,6-(*t*BuNCH)$_2$C$_6$H$_3$)Bi(benzyl)(tetrafluoroborate)] (10)

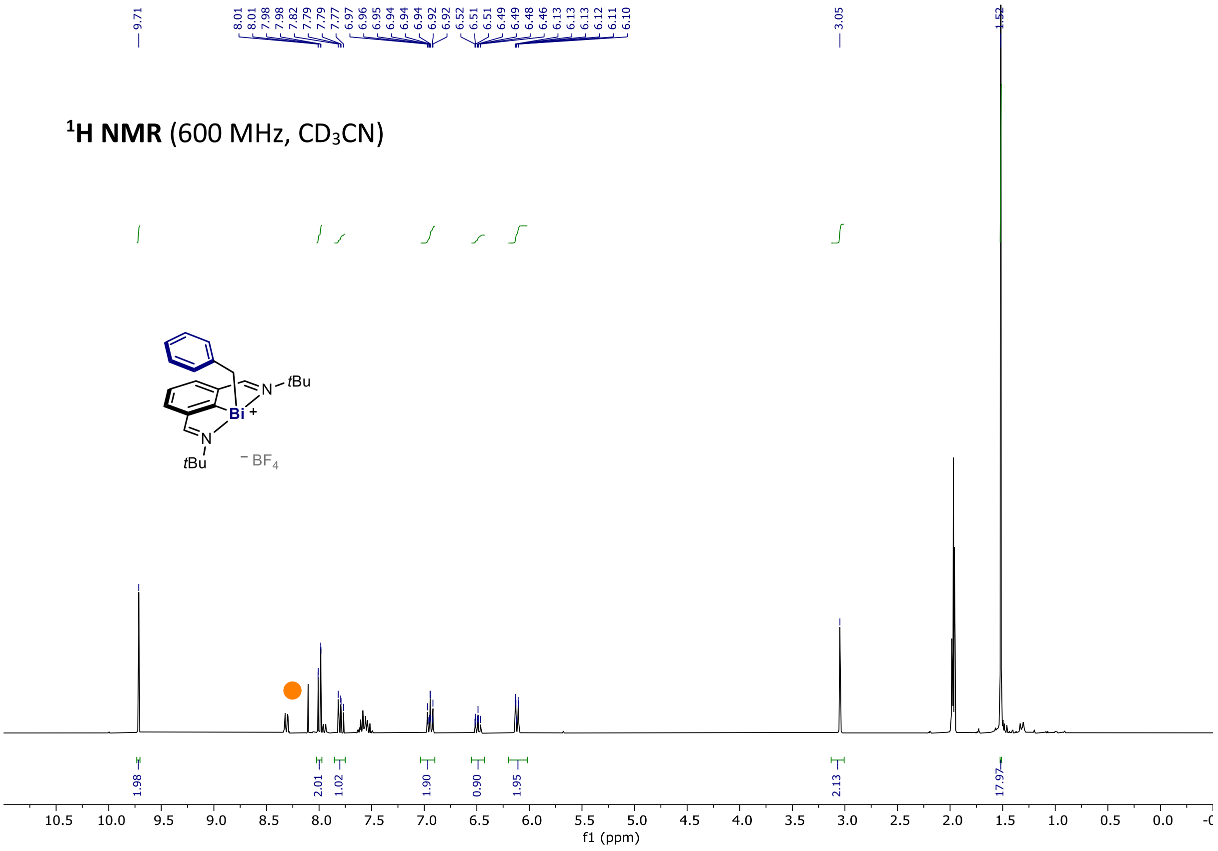

● Residual triphenylpyridine

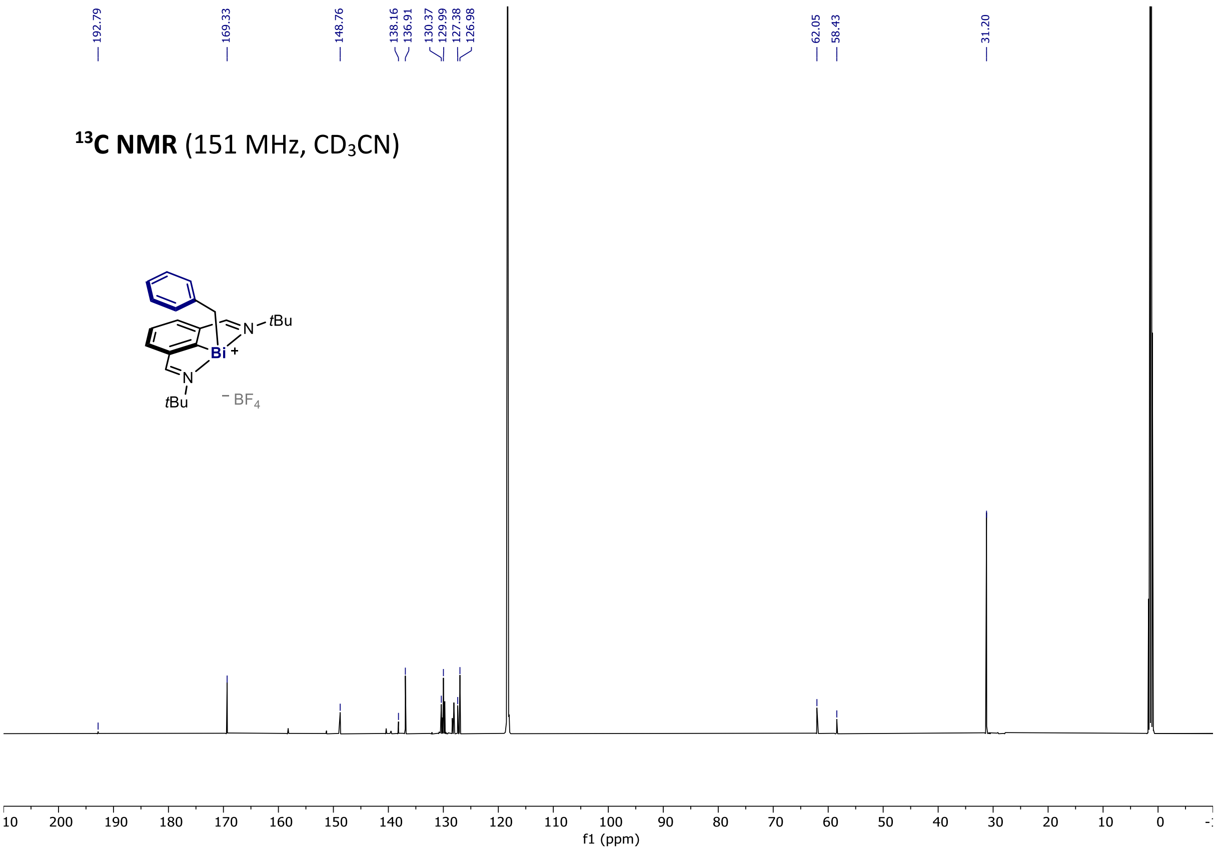

[(2,6-(*t*BuNCH)$_2$C$_6$H$_3$)Bi(1-adamantylmethyl)(tetrachlorophthalimide)] (12)

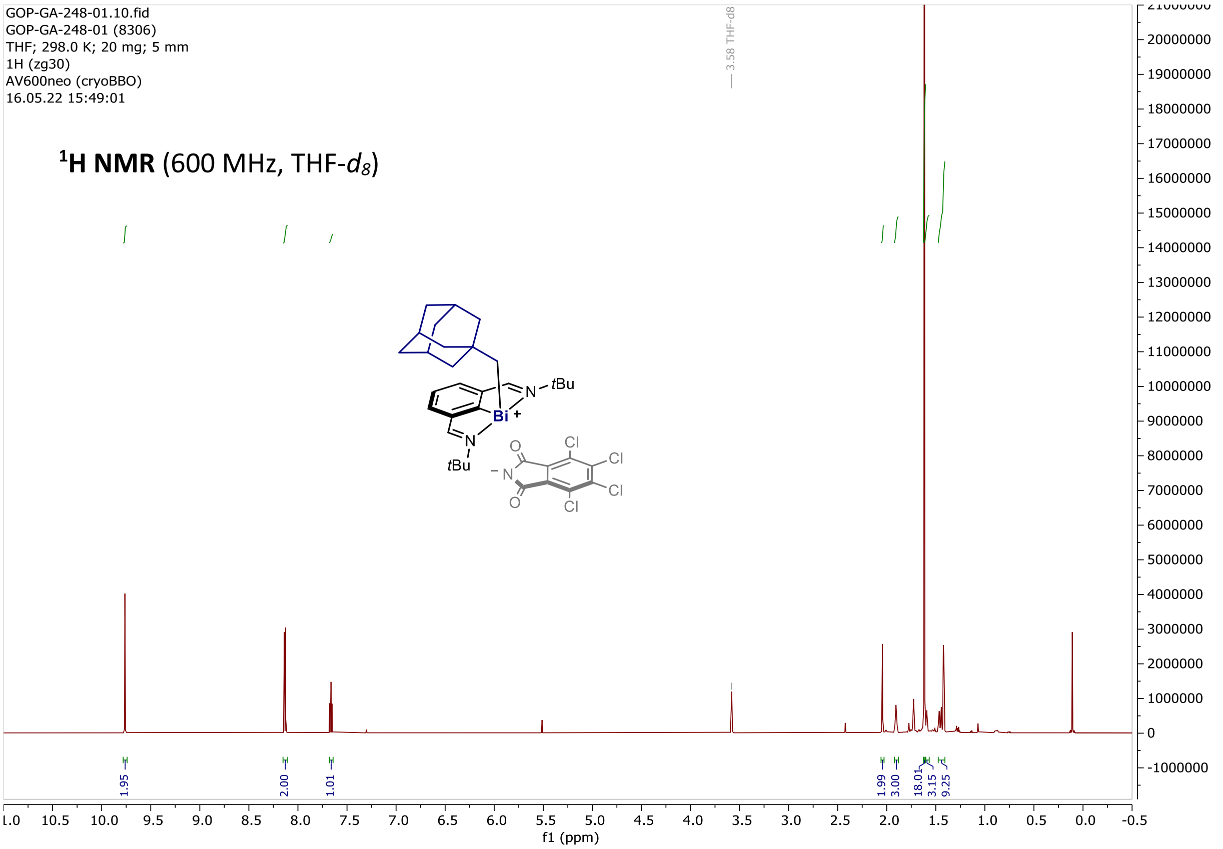

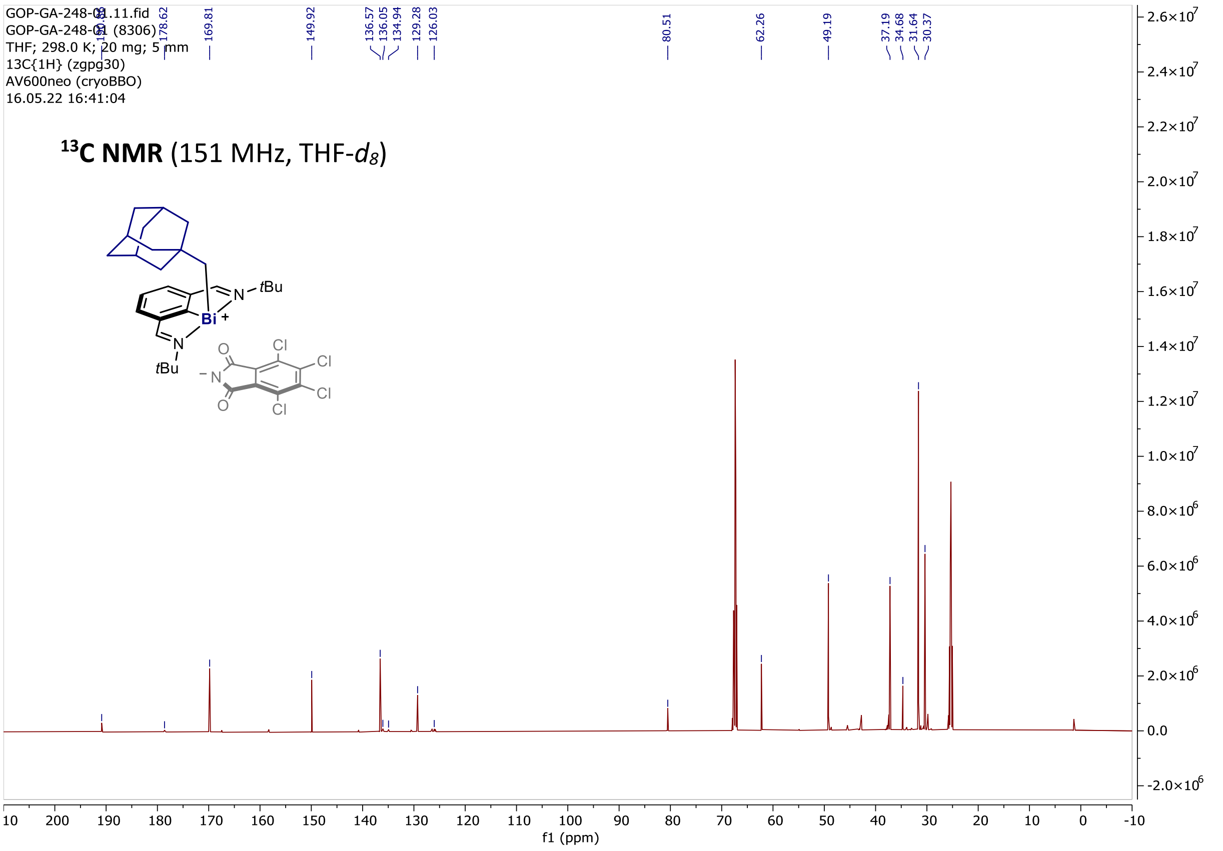

[(2,6-(tBuNCH)$_2$C$_6$H$_3$)Bi(1-adamantylmethyl)(iodide)] (14)

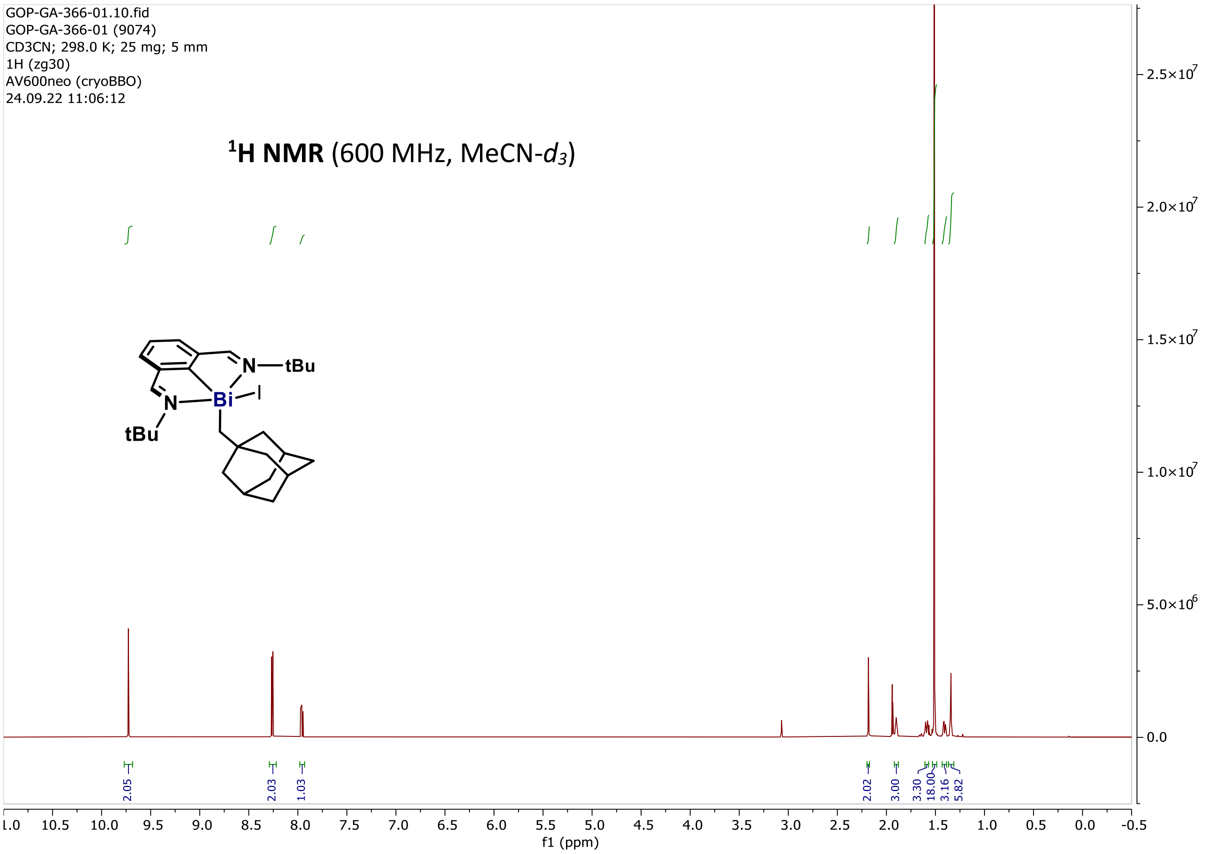

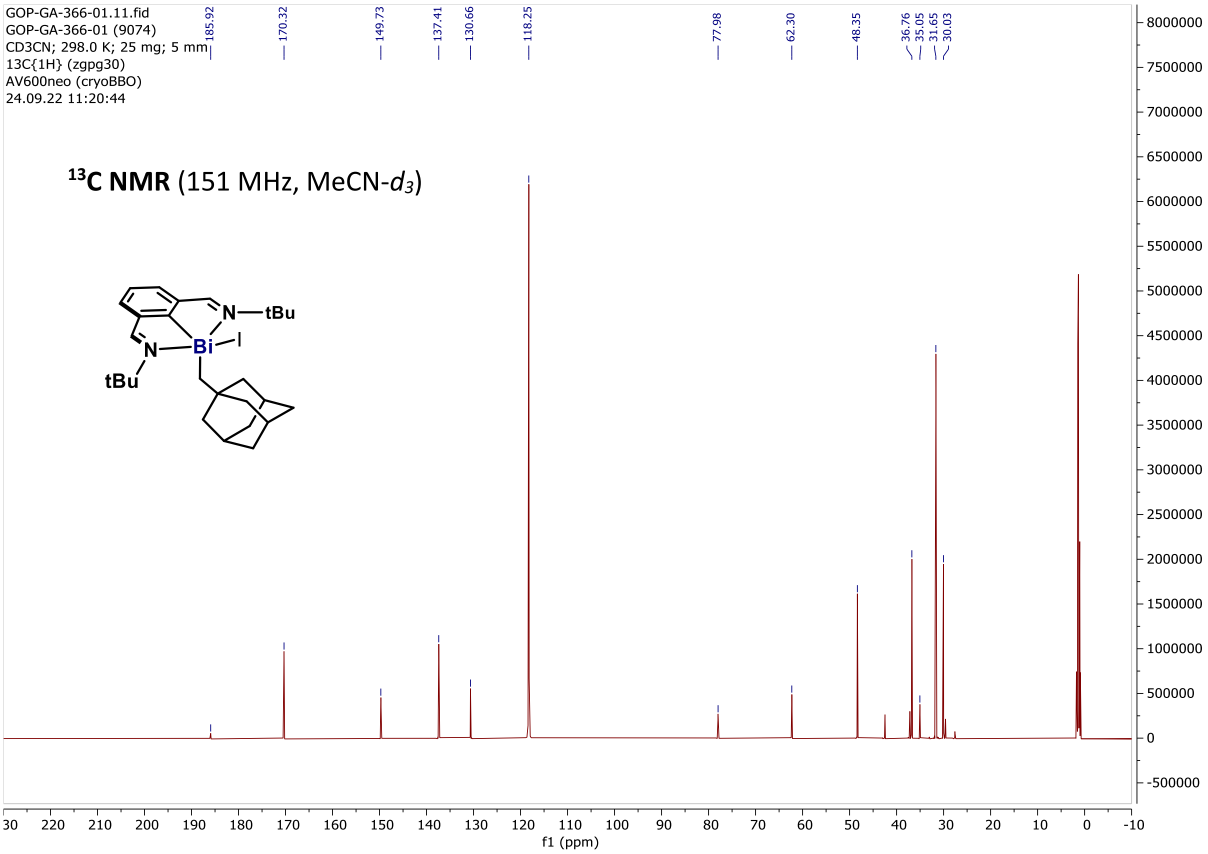

[(2,6-(*t*BuNCH)$_2$C$_6$H$_3$)Bi(*p*-bromobenzyl)(tetrachlorophthalimide)] (11)

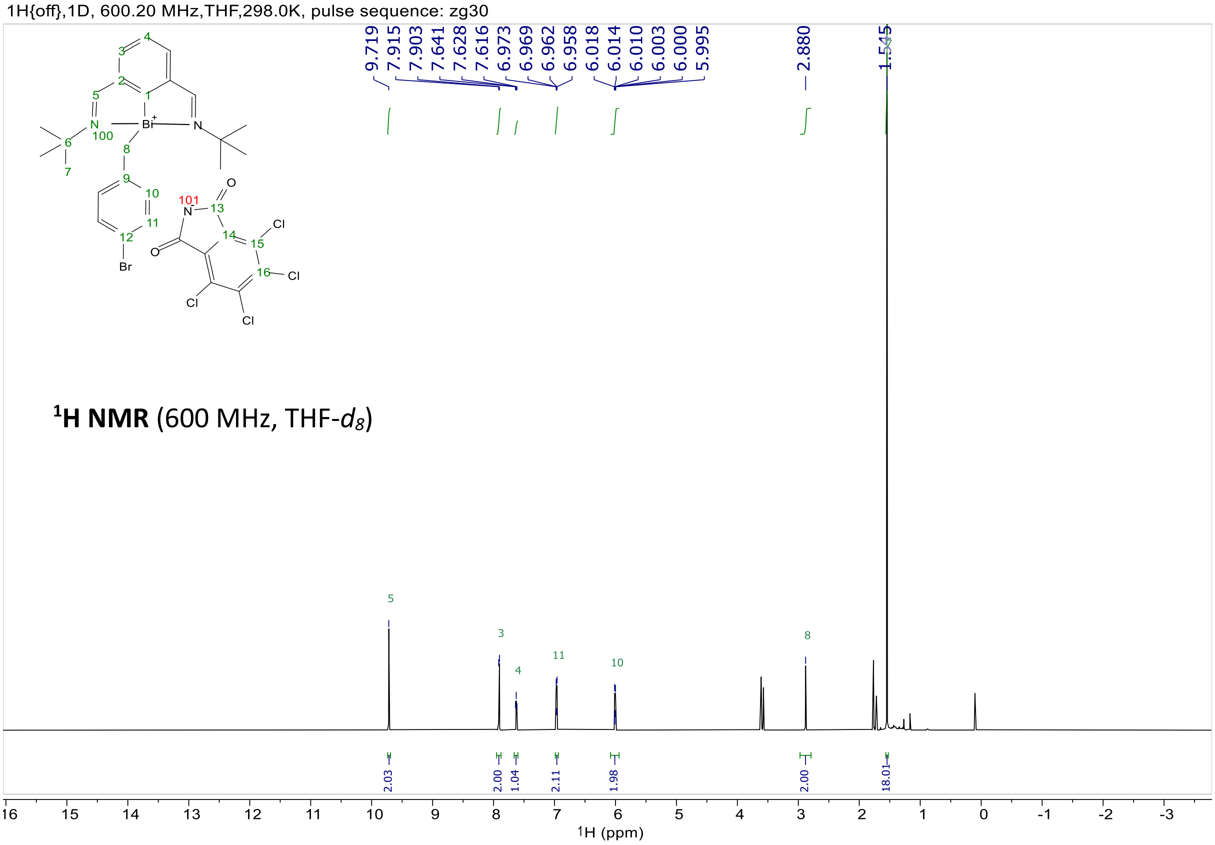

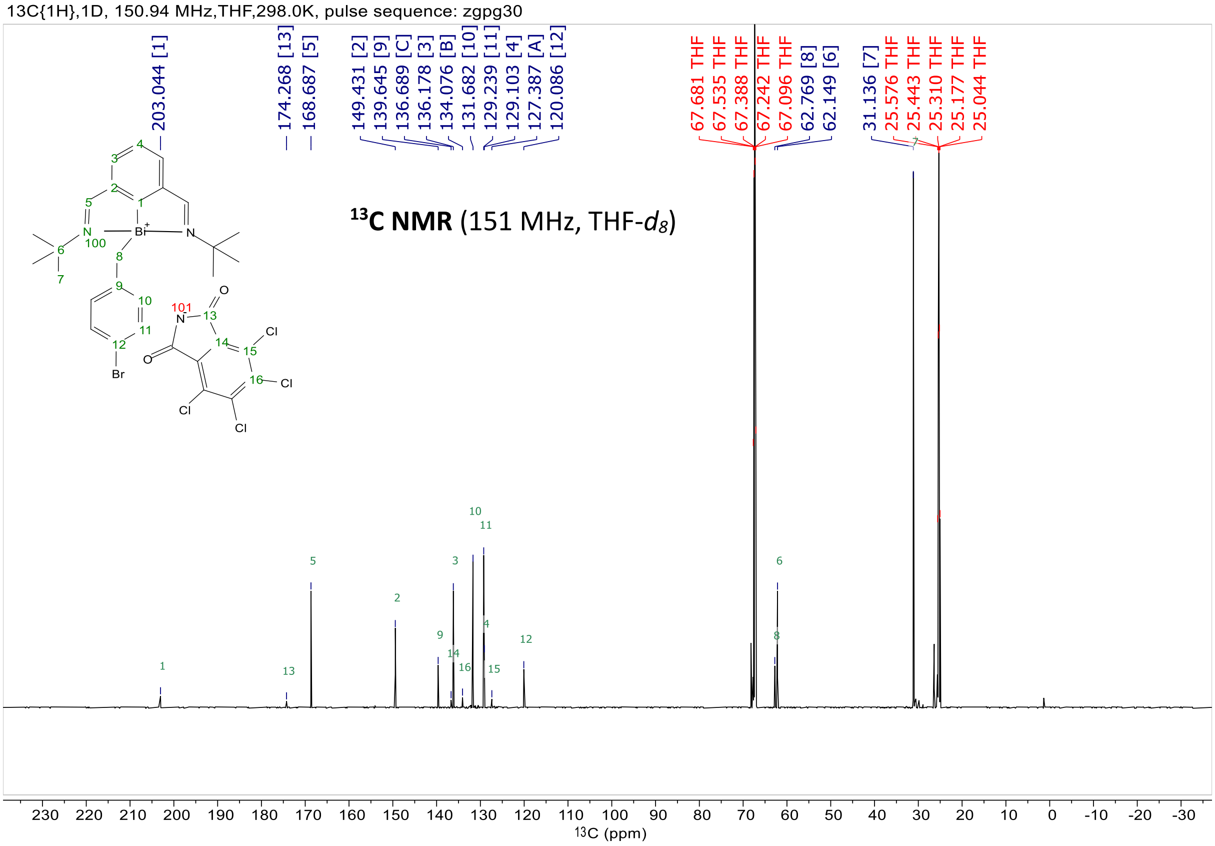

Variable-temperature NMR of (11) (edited)

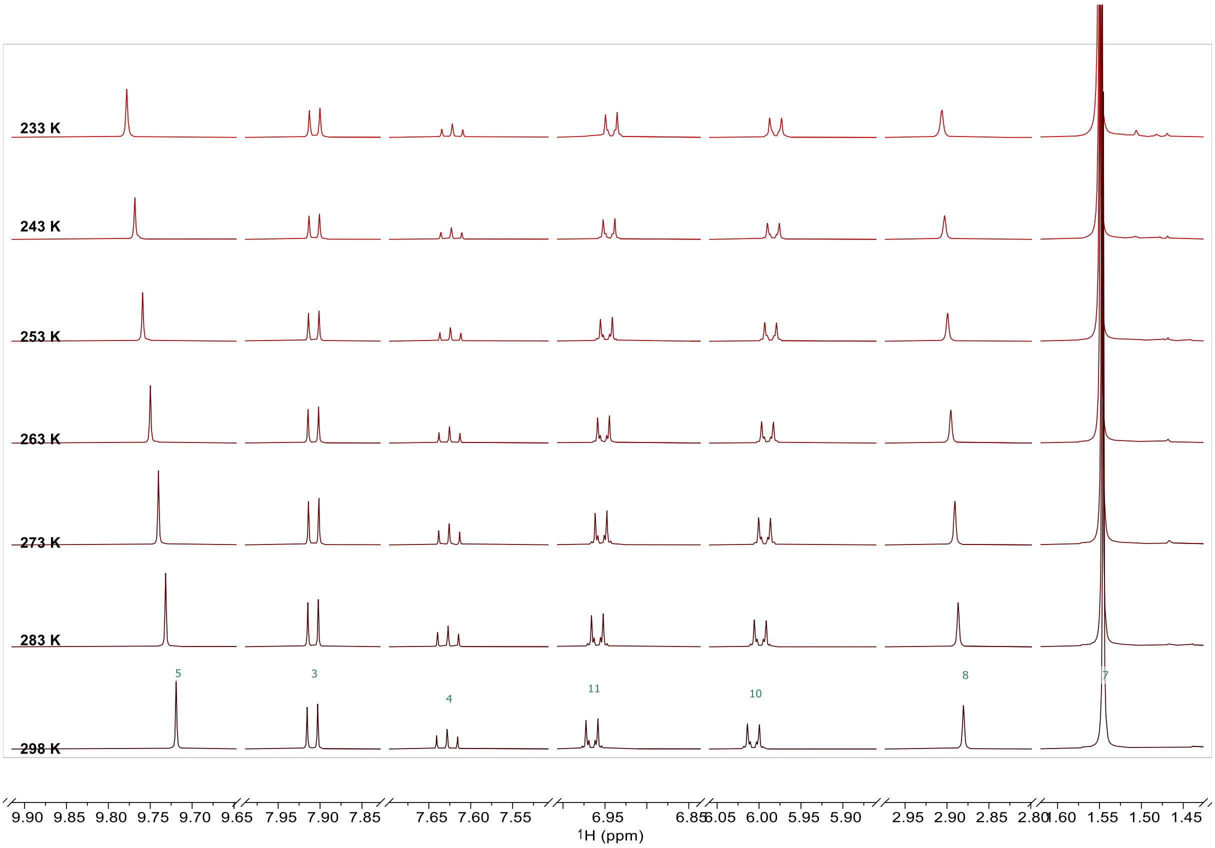

13C{1H},1D, 150.94 MHz,THF,233.0K, pulse sequence: zgpg30

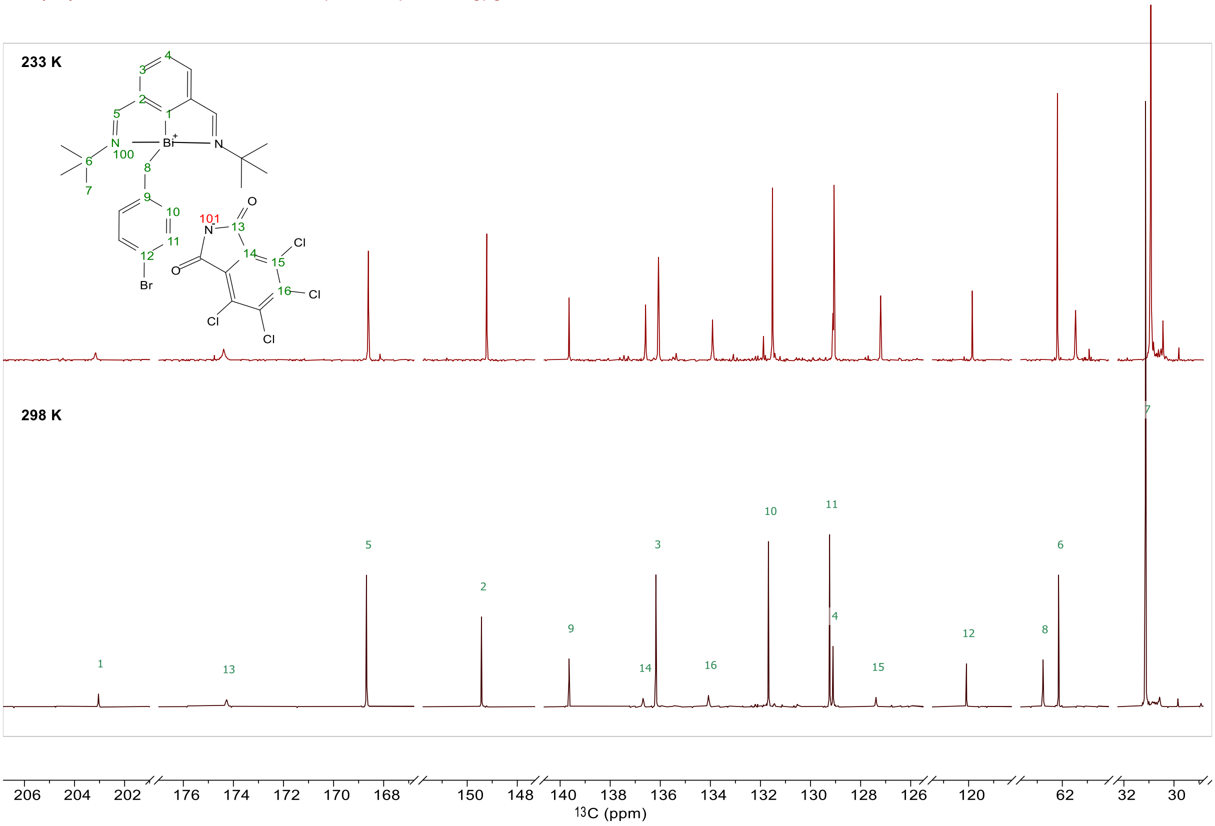

* ^{13}C signals of the phthalimide are broad, suggesting a equilibrium of the tetrachlorophthalimide counteranion in solution.

$^1H\{^{13}C\}$ HSQC of **11**

1H{13C},HSQC-EDITED, 600.20 MHz,THF,298.0K, pulse sequence: hsqcedetgpsisp2.3

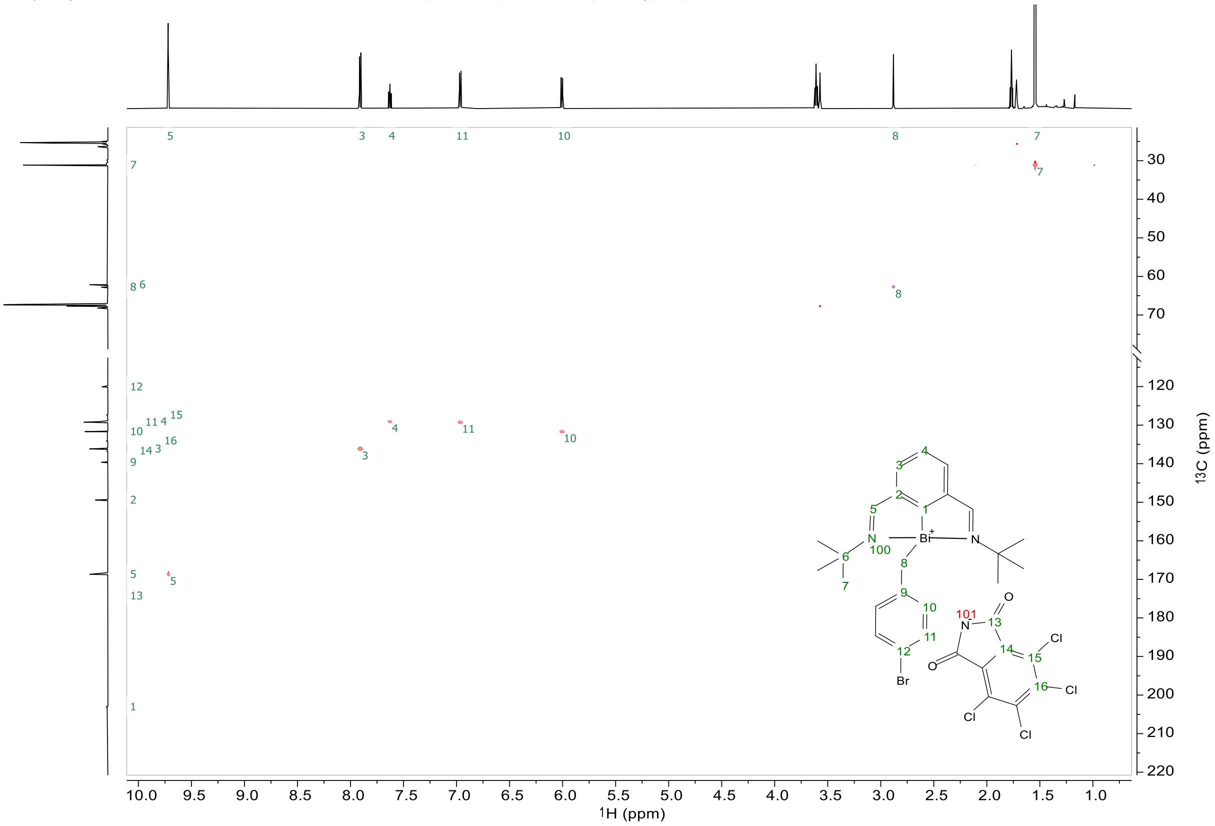

$^1H\{^{13}C\}$ HMBC of **11**

1H{13C},HMBC, 600.20 MHz,THF,298.0K, pulse sequence: hmbcetgpl3nd

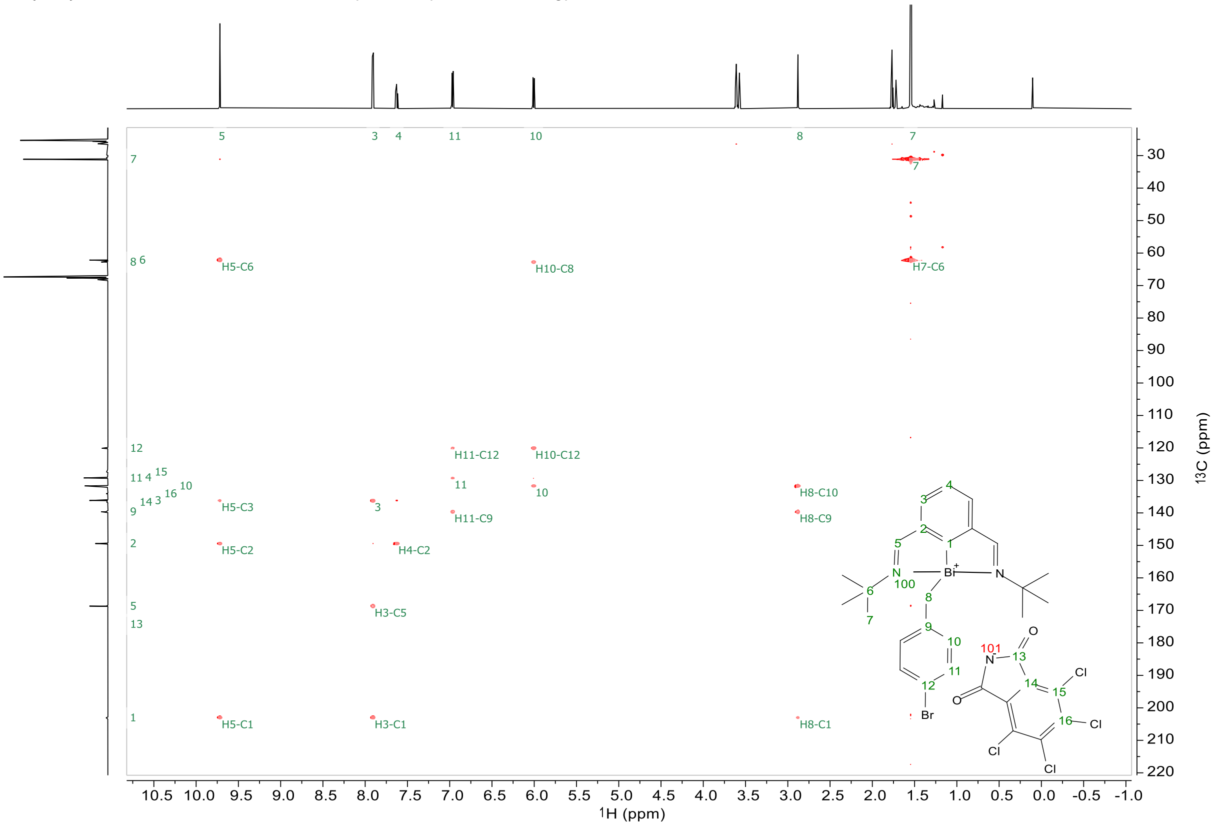

^{1}H COSY of **11**

1H{off},COSY, 600.20 MHz,THF,298.0K, pulse sequence: cosygpppqf

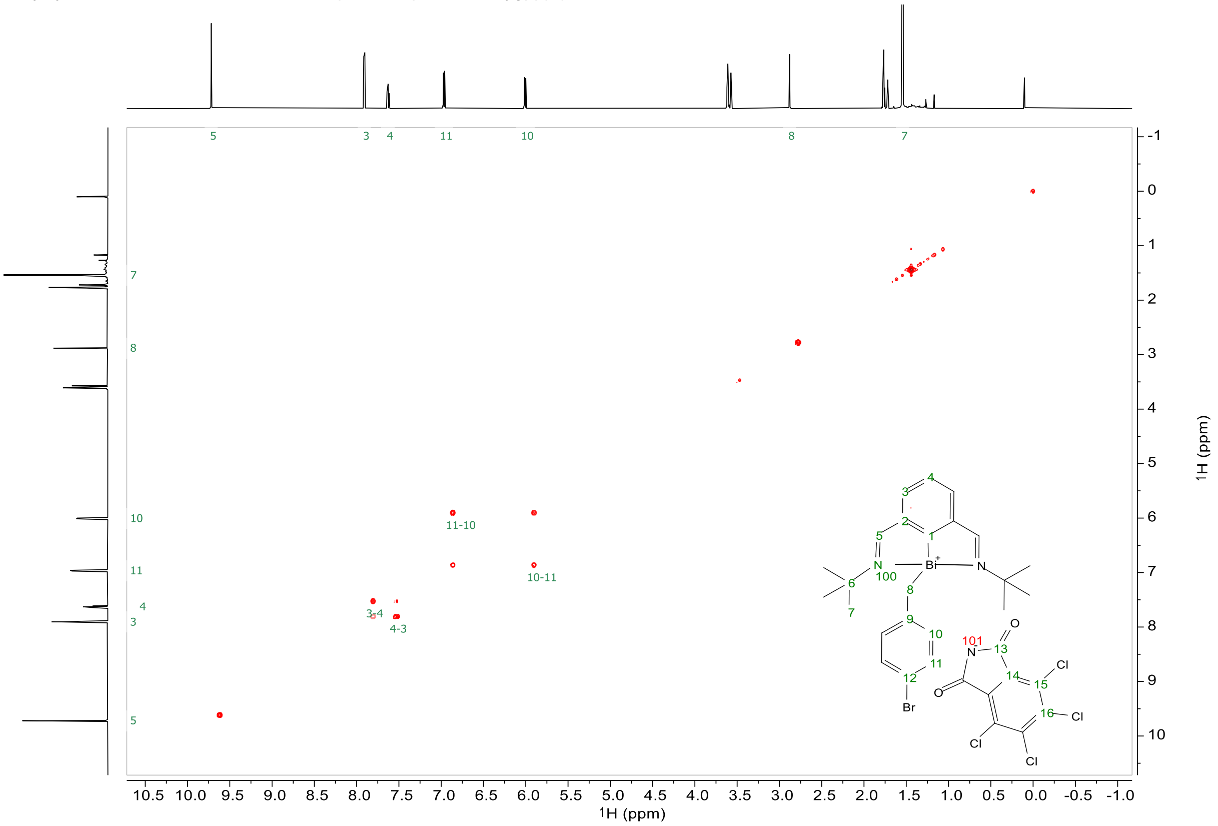

^{1}H NOESY of **11**

1H{off},NOESY, 600.20 MHz,THF,298.0K, pulse sequence: noesygpphpp

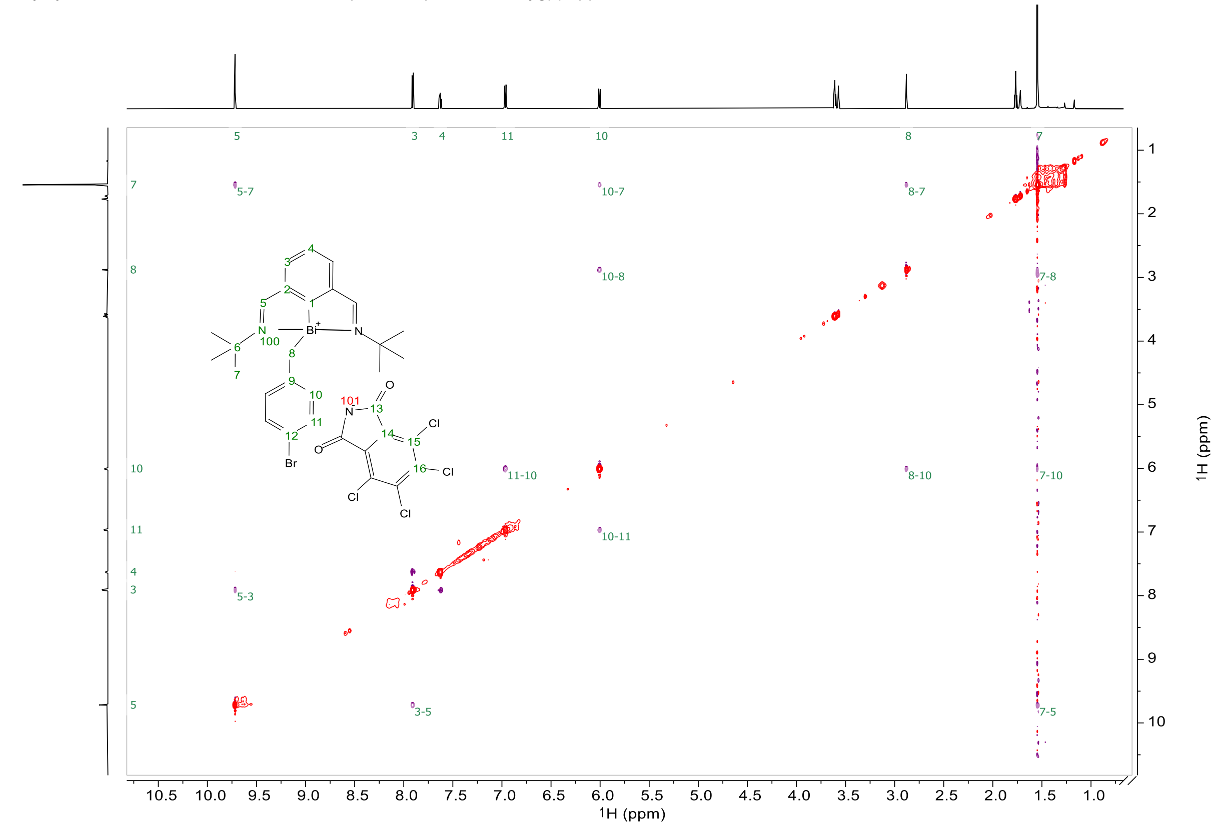

$^1H\{^{15}N\}$ HMBC of **11**

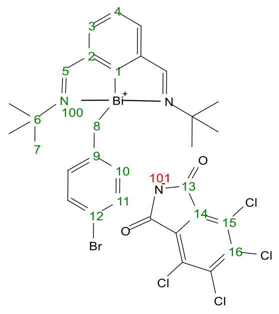

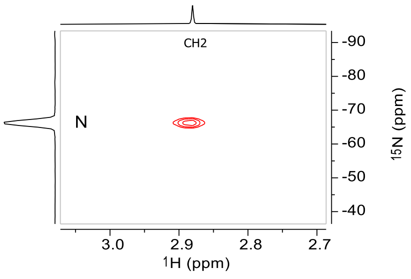

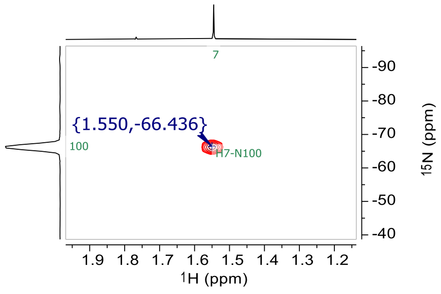

1H{15N},HMBC, 600.20 MHz,THF,298.0K, pulse sequence: hmbcgpndqf

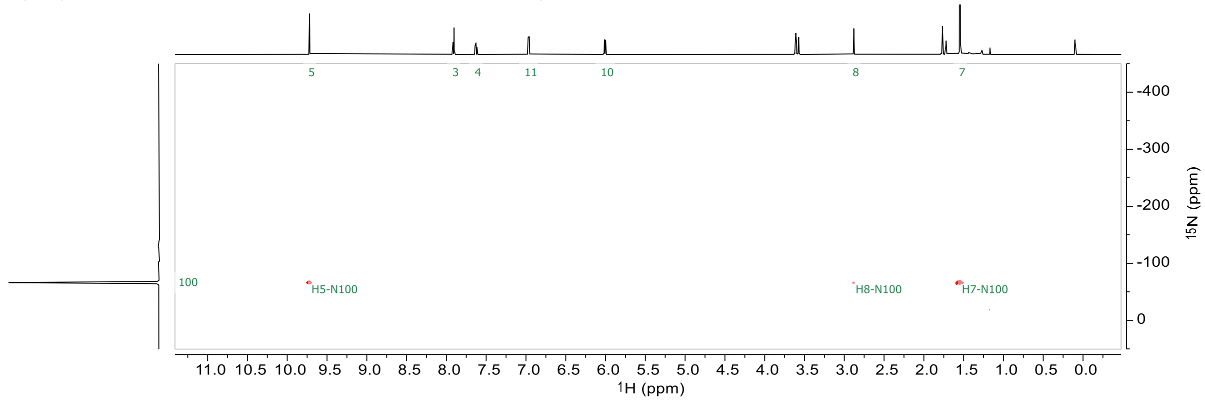

^1H DOSY of **11**

1H{},DOSY, 600.20 MHz,THF,298.002593994140625K, pulse sequence:

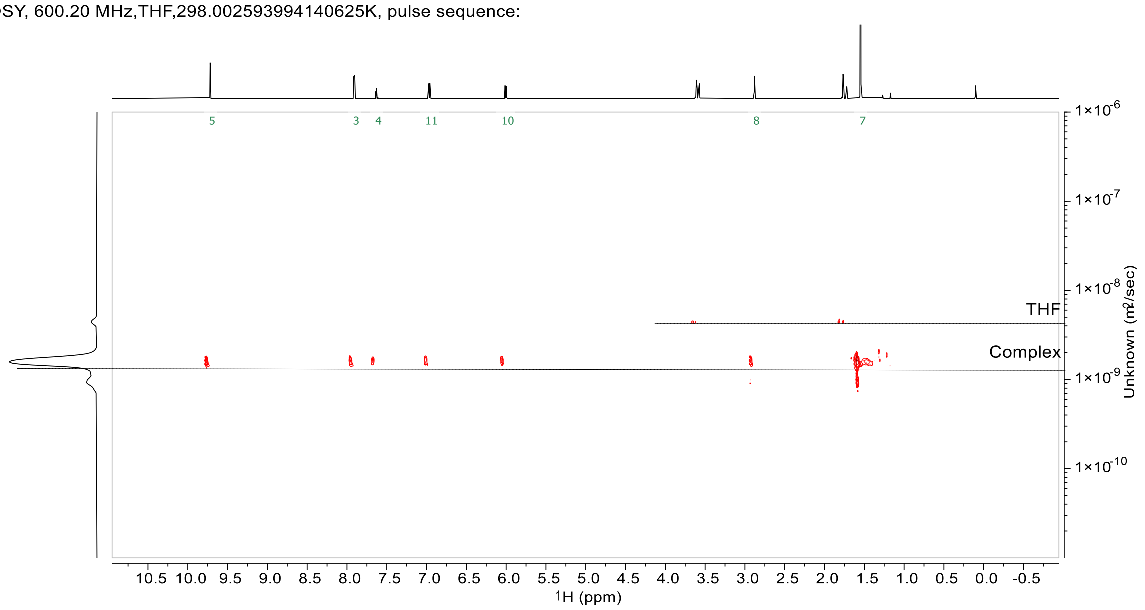

[(2,6-(*t*BuNCH)$_2$C$_6$H$_3$)Bi(1-tosylpiperidin-4-yl)(tetrachlorophthalimide)] (**13**)

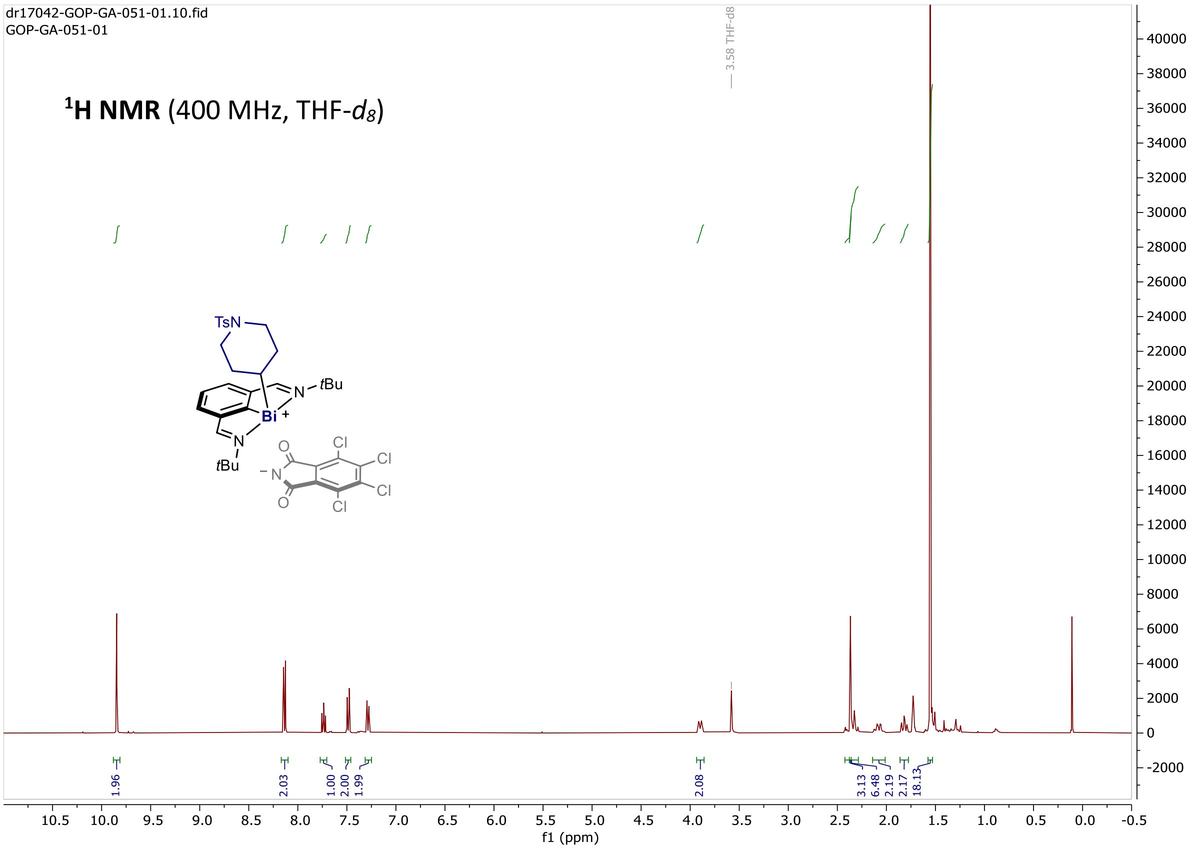

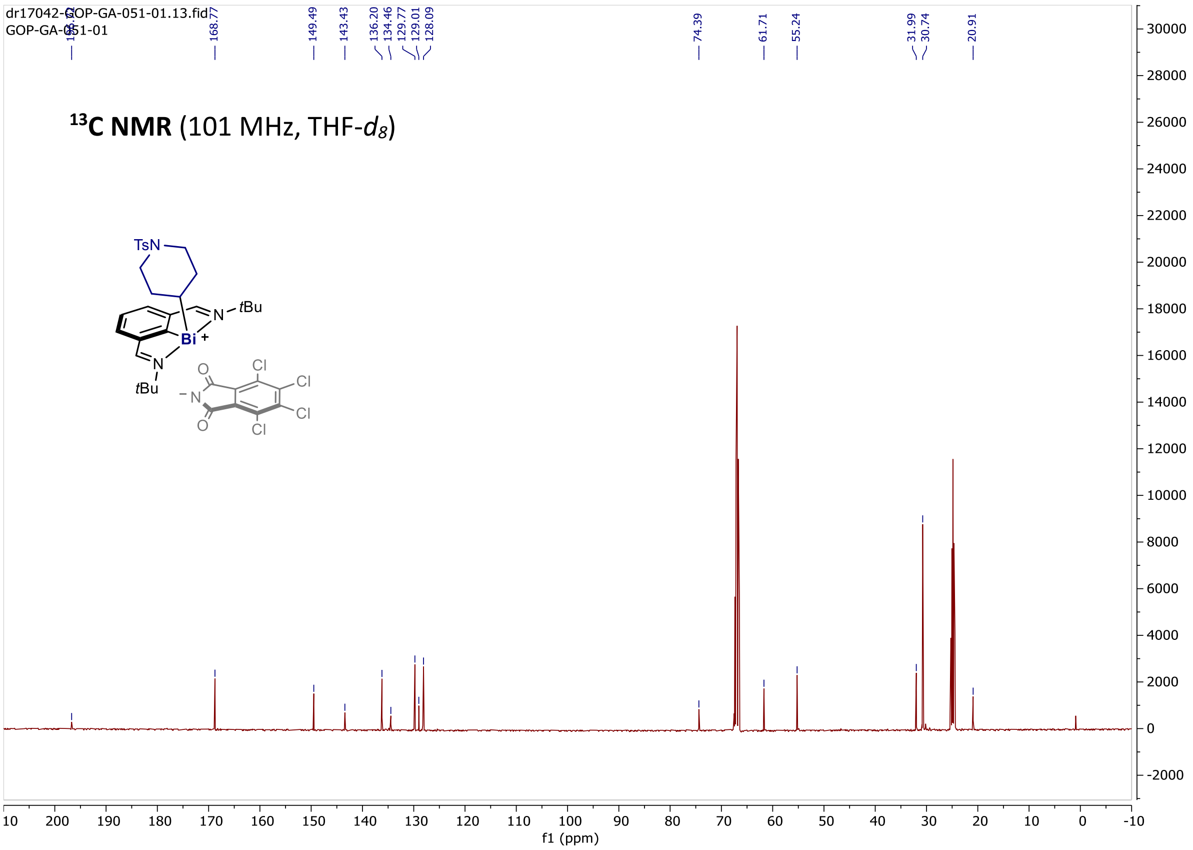

[(2,6-(*t*BuNCH)$_2$C$_6$H$_3$)Bi(but-3-en-1-yl)(tetrachlorophthalimide)] (19)

^{1}H NMR (600 MHz, CD$_3$CN)

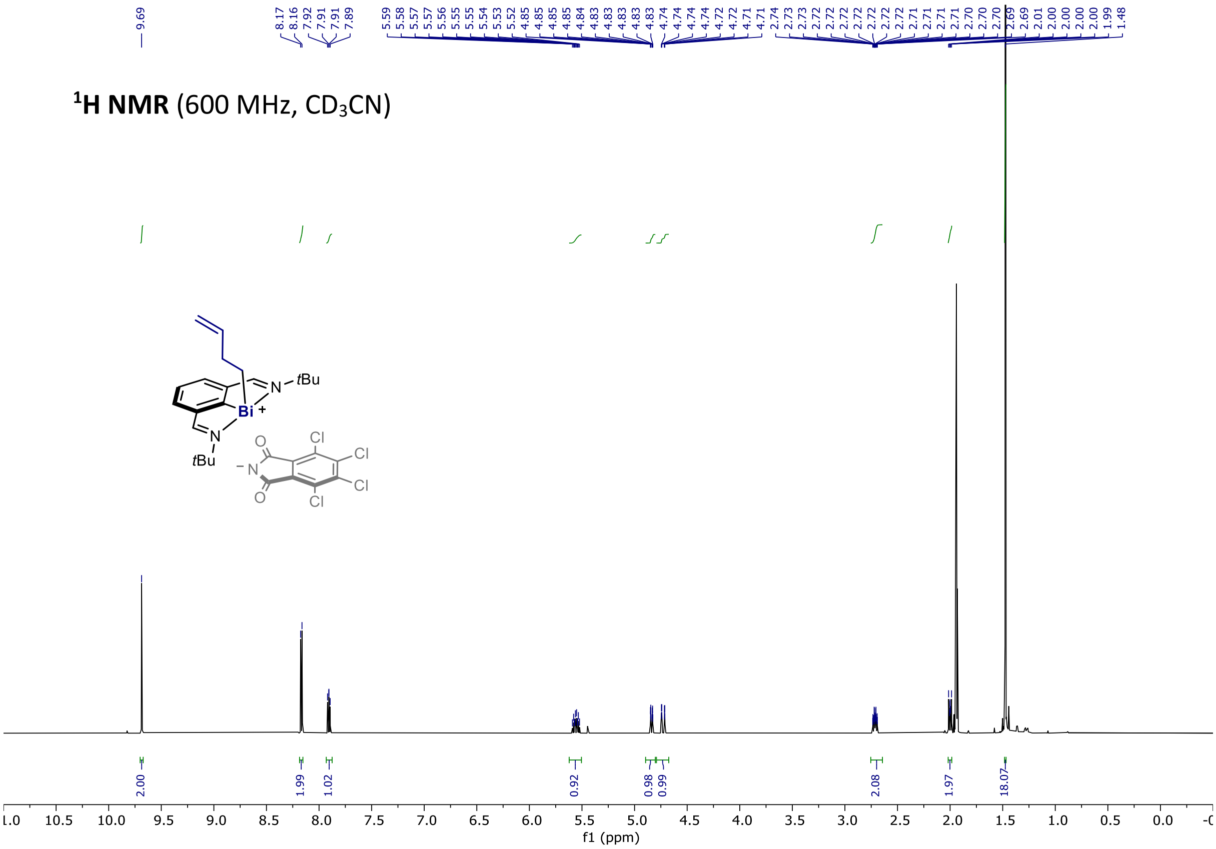

^{13}C NMR (151 MHz, CD$_3$CN)

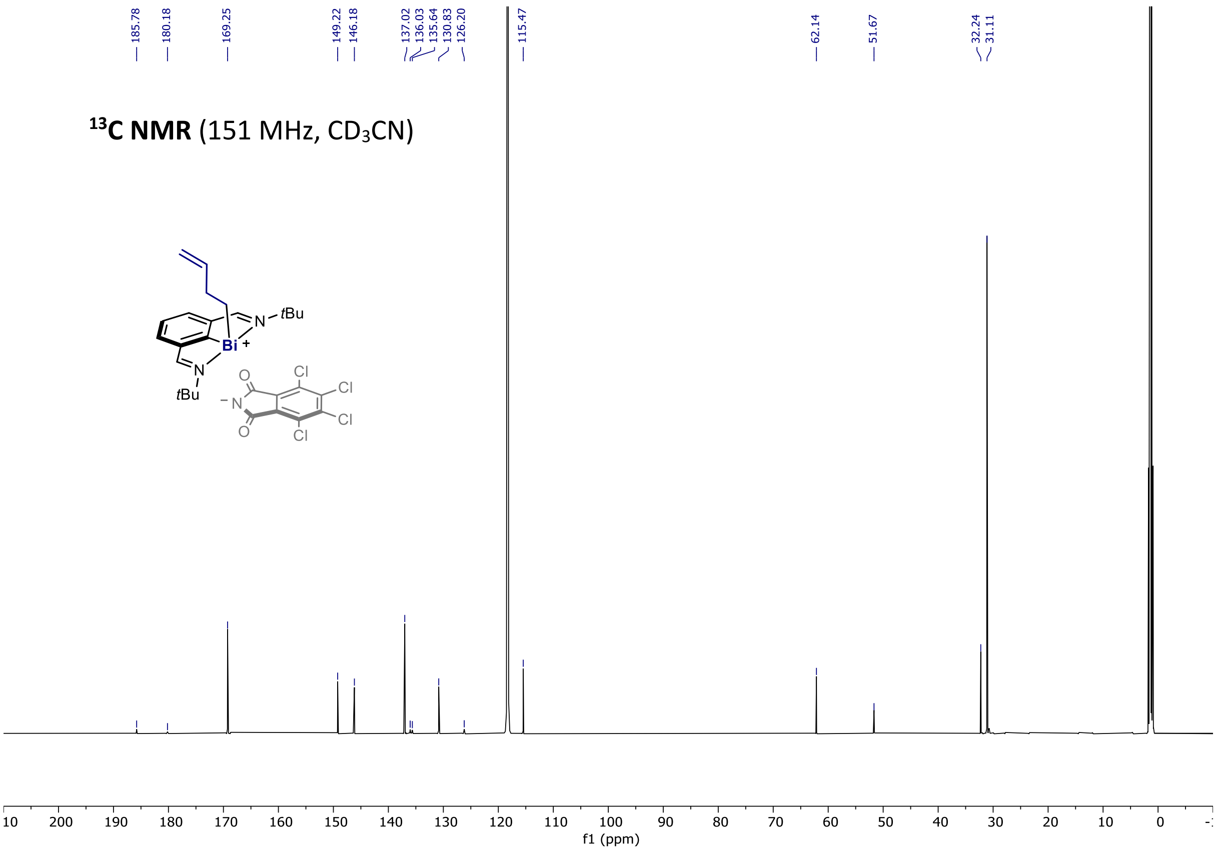

[(2,6-(*t*BuNCH)$_2$C$_6$H$_3$)Bi(2-cyclopropylmethyl)(iodide)] (16)

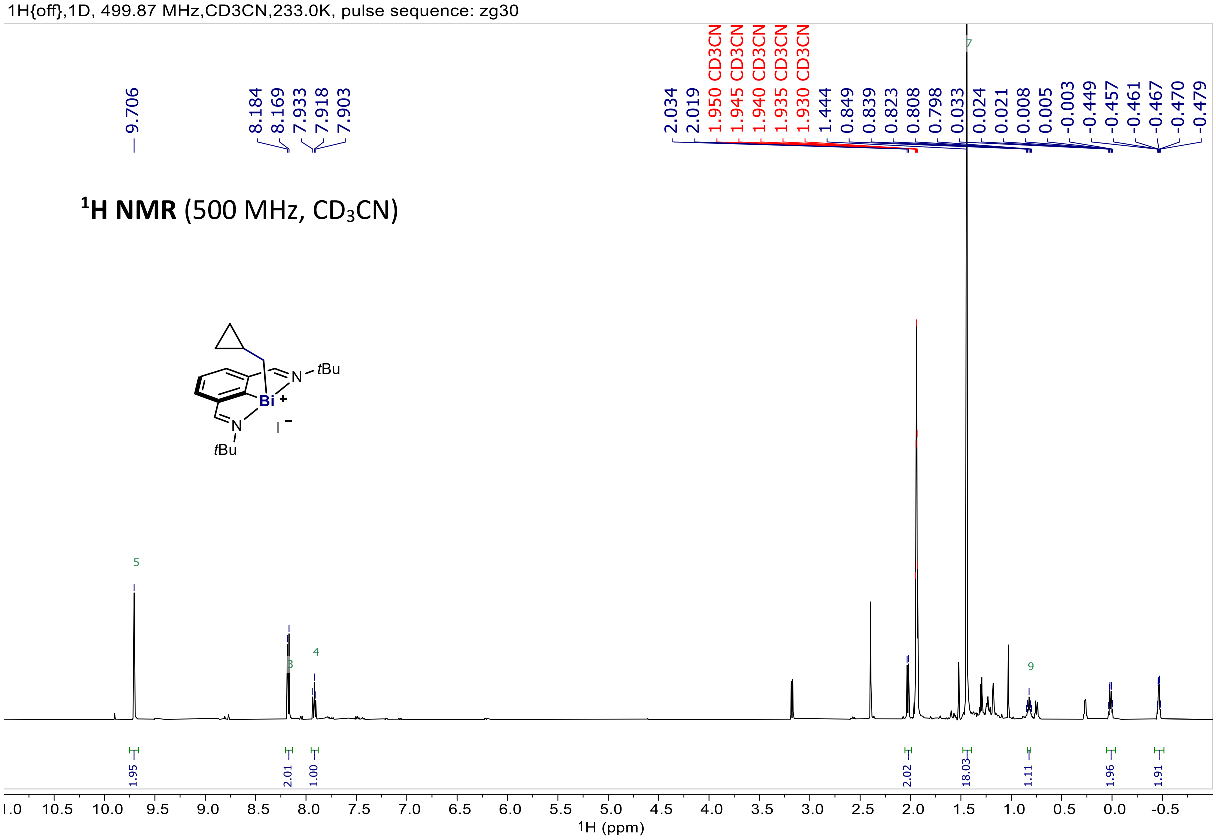

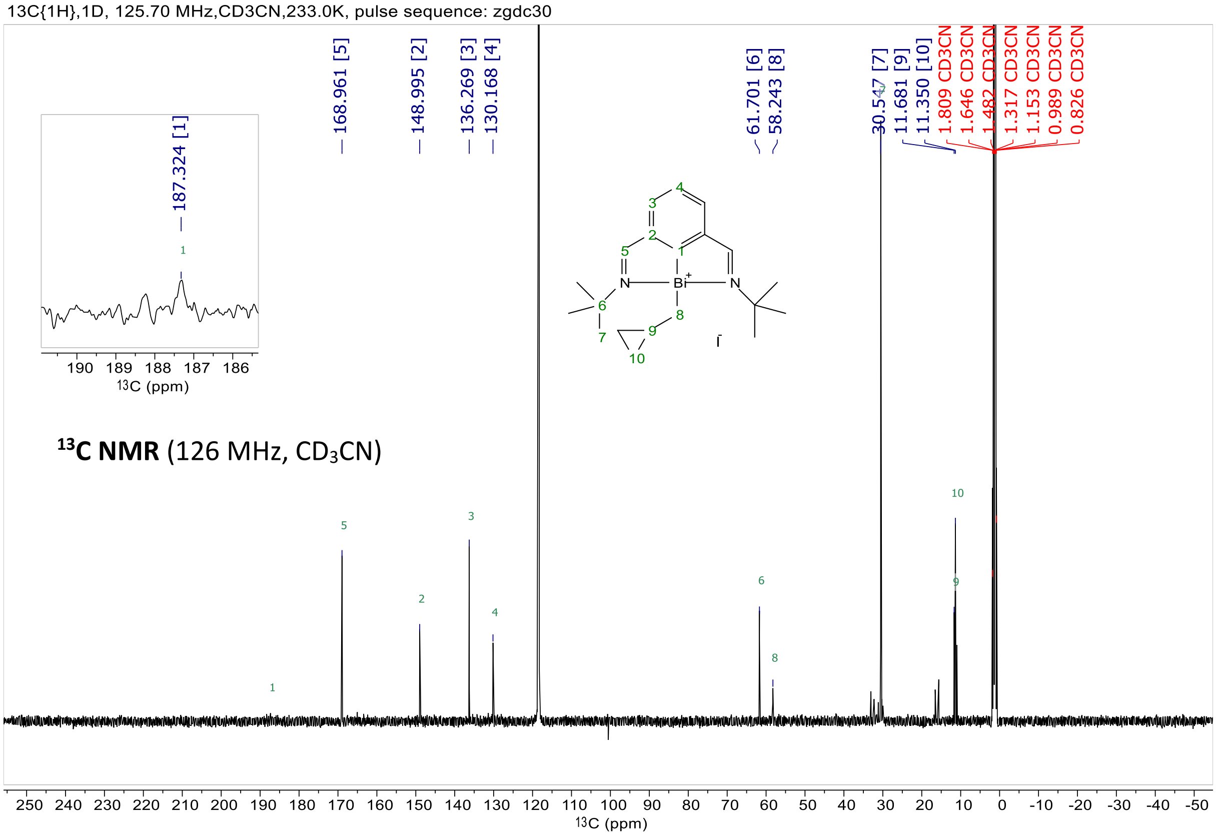

HSQC of 16 (edited)

1H{13C},HSQC-EDITED, 499.87 MHz,CD3CN,233.0K, pulse sequence: hsqcedetgpsisp2

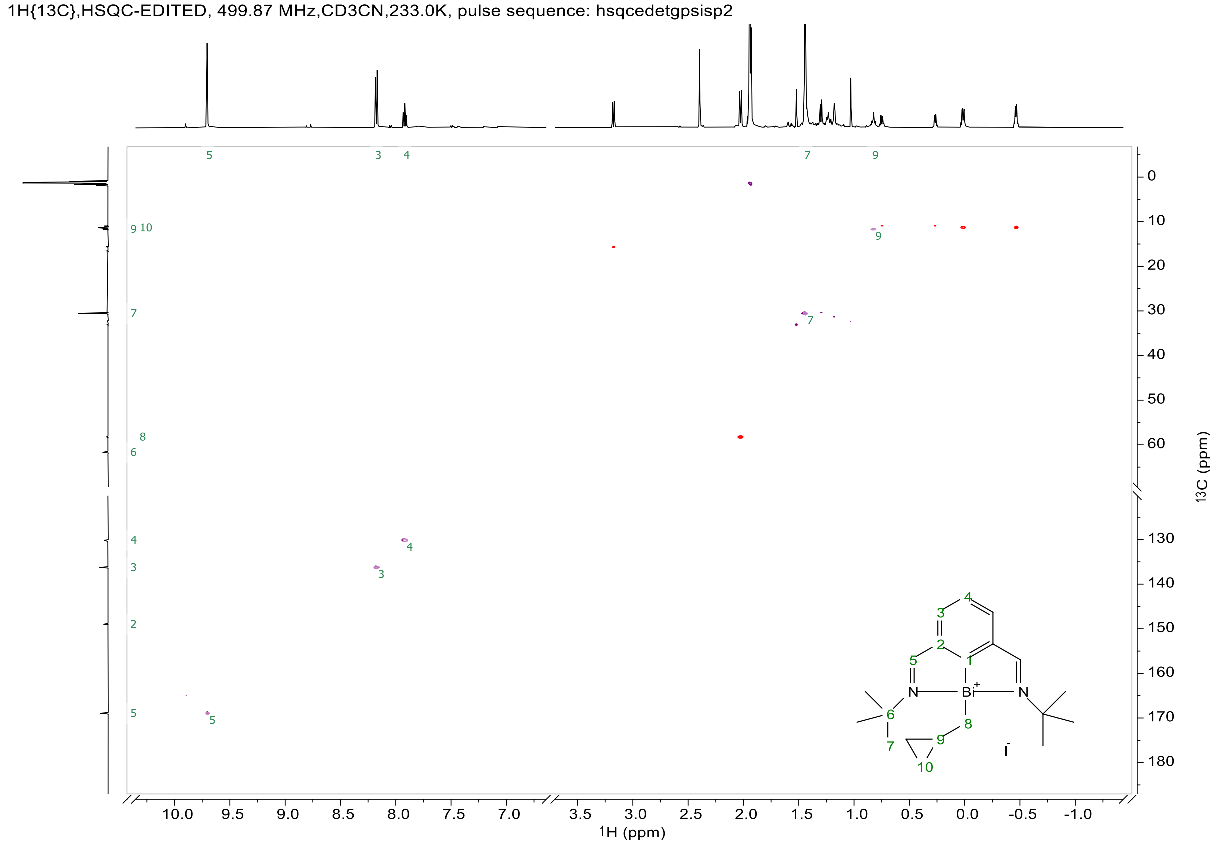

HMBC of 16

1H{13C},HMBC, 499.87 MHz,CD3CN,233.0K, pulse sequence: hmbcetgpl3nd

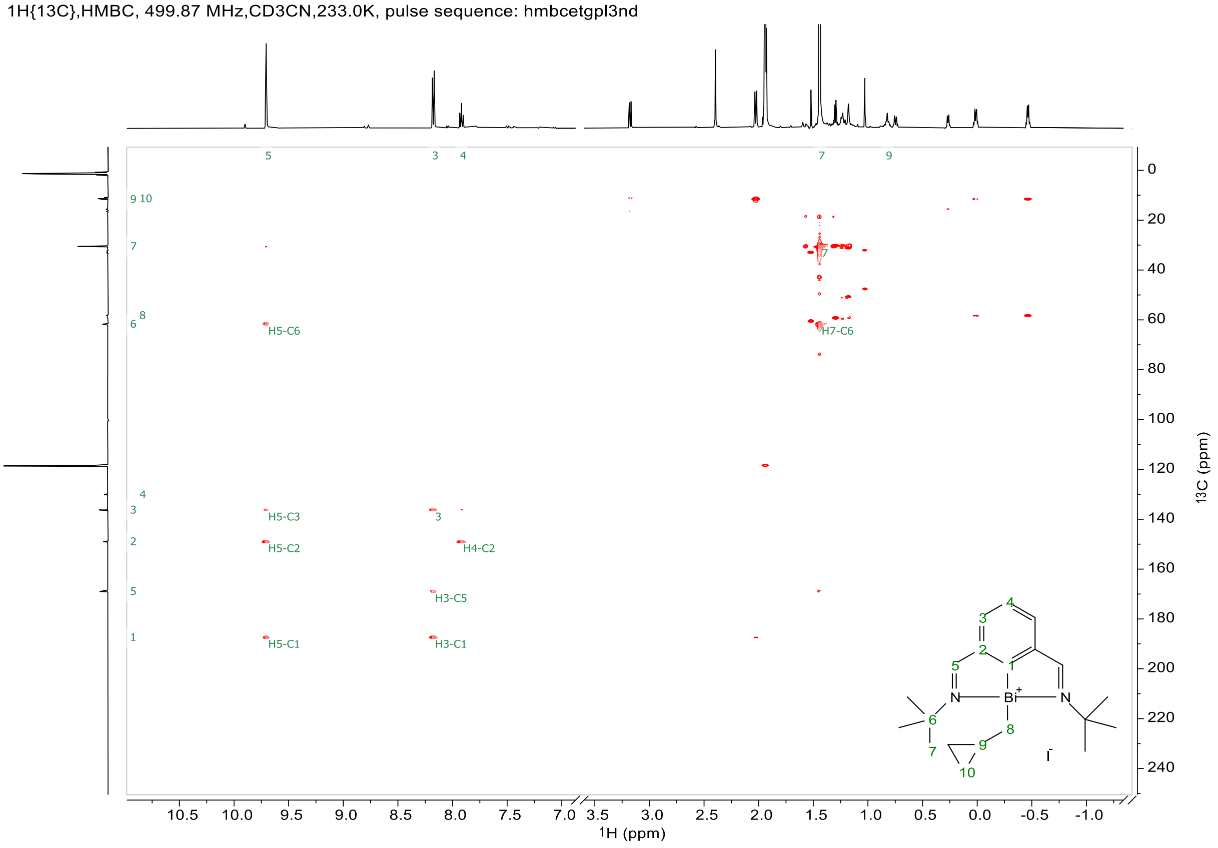

[(2,6-(*t*BuNCH)$_2$C$_6$H$_3$)Bi(but-3-en-1-yl)(iodide)] (18)

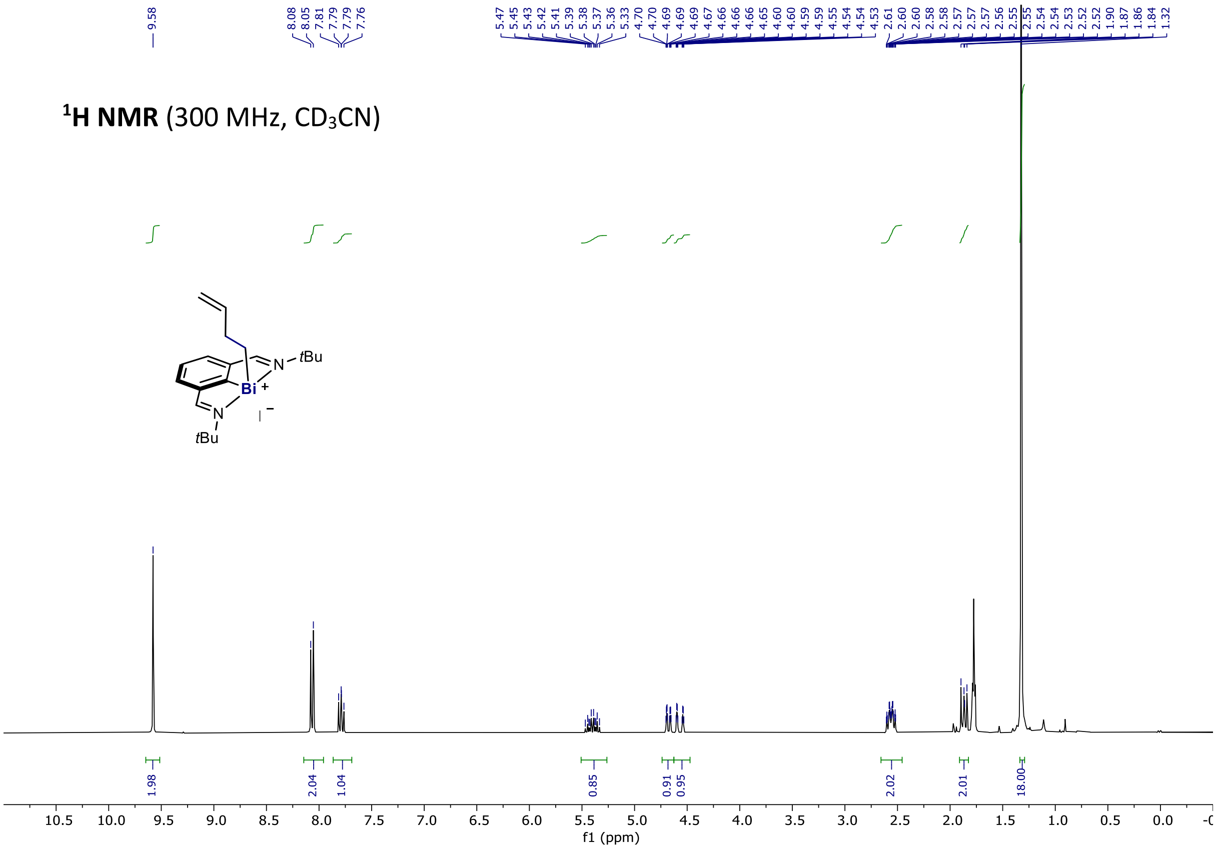

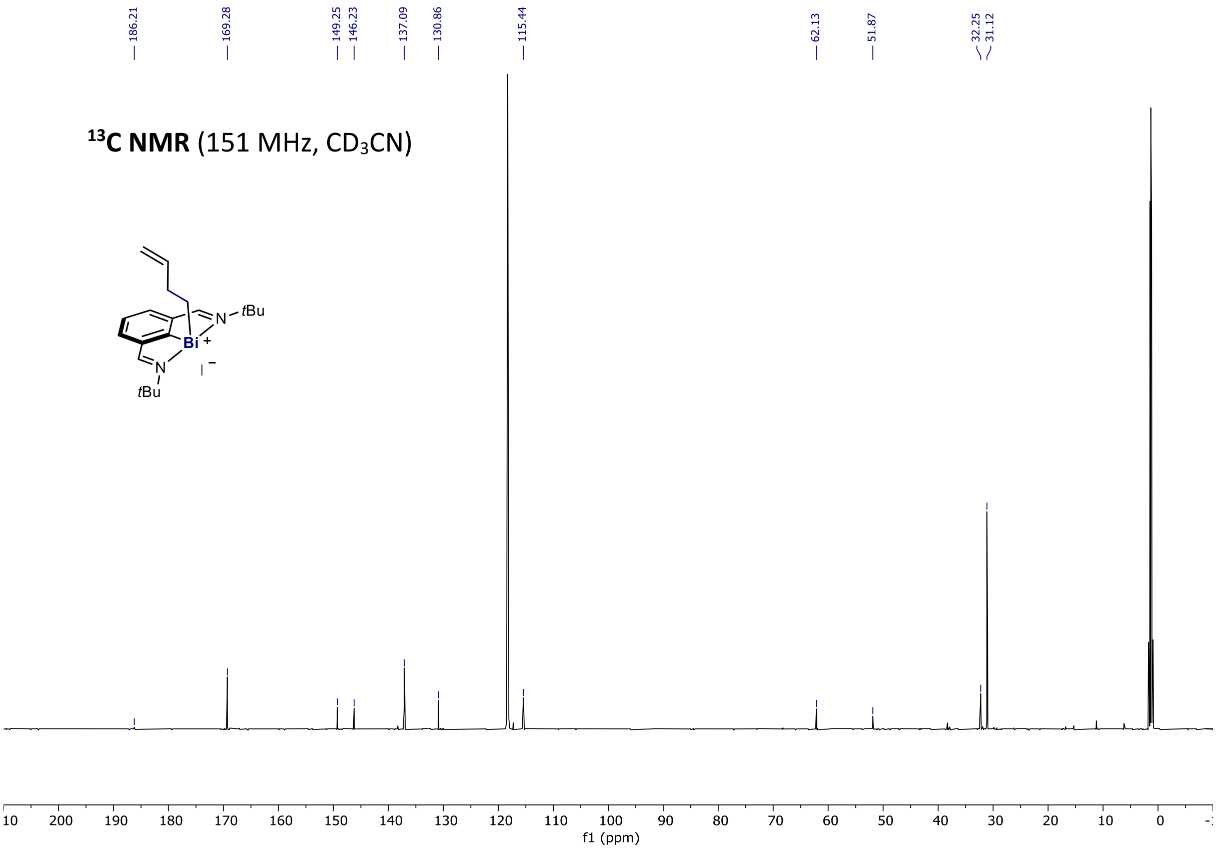

1-(Benzyloxy)-2,2,6,6-tetramethylpiperidine (S21), isolated product

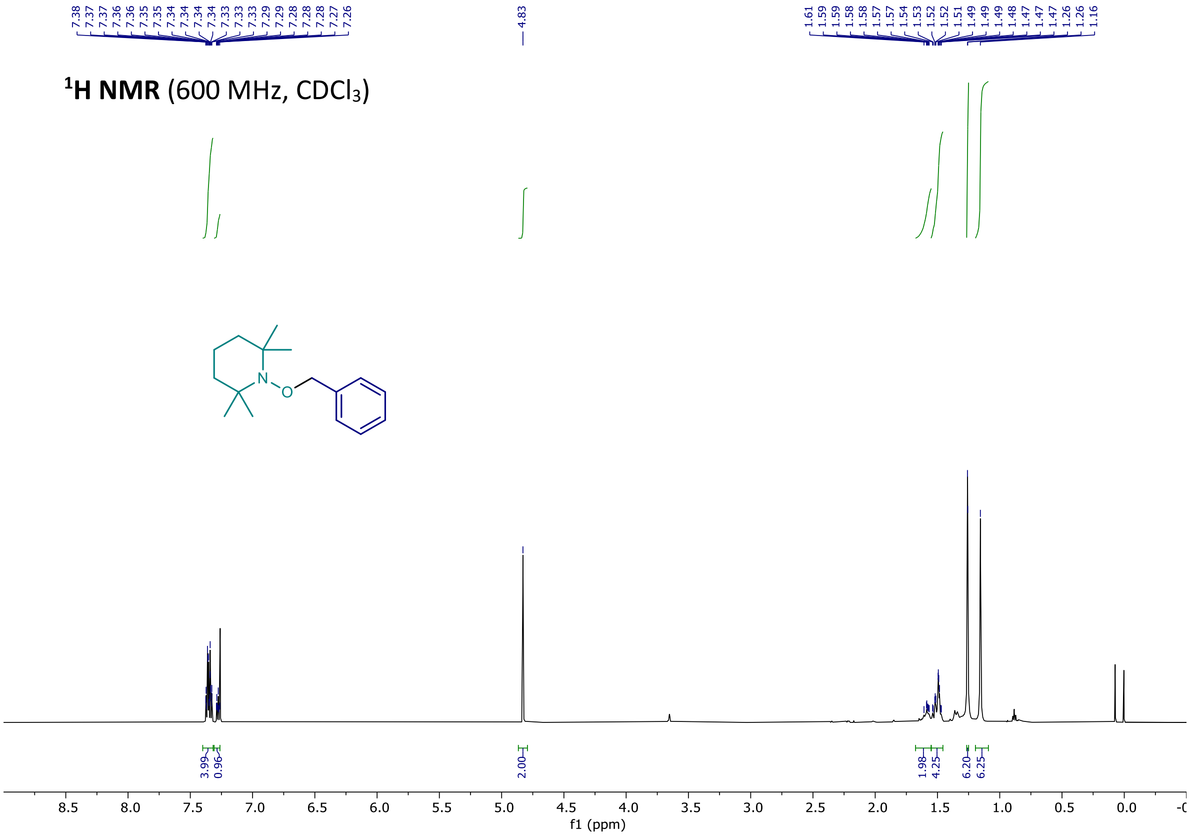

1-((4-bromobenzyl)oxy)-2,2,6,6-tetramethylpiperidine (S22),crude reaction mixture

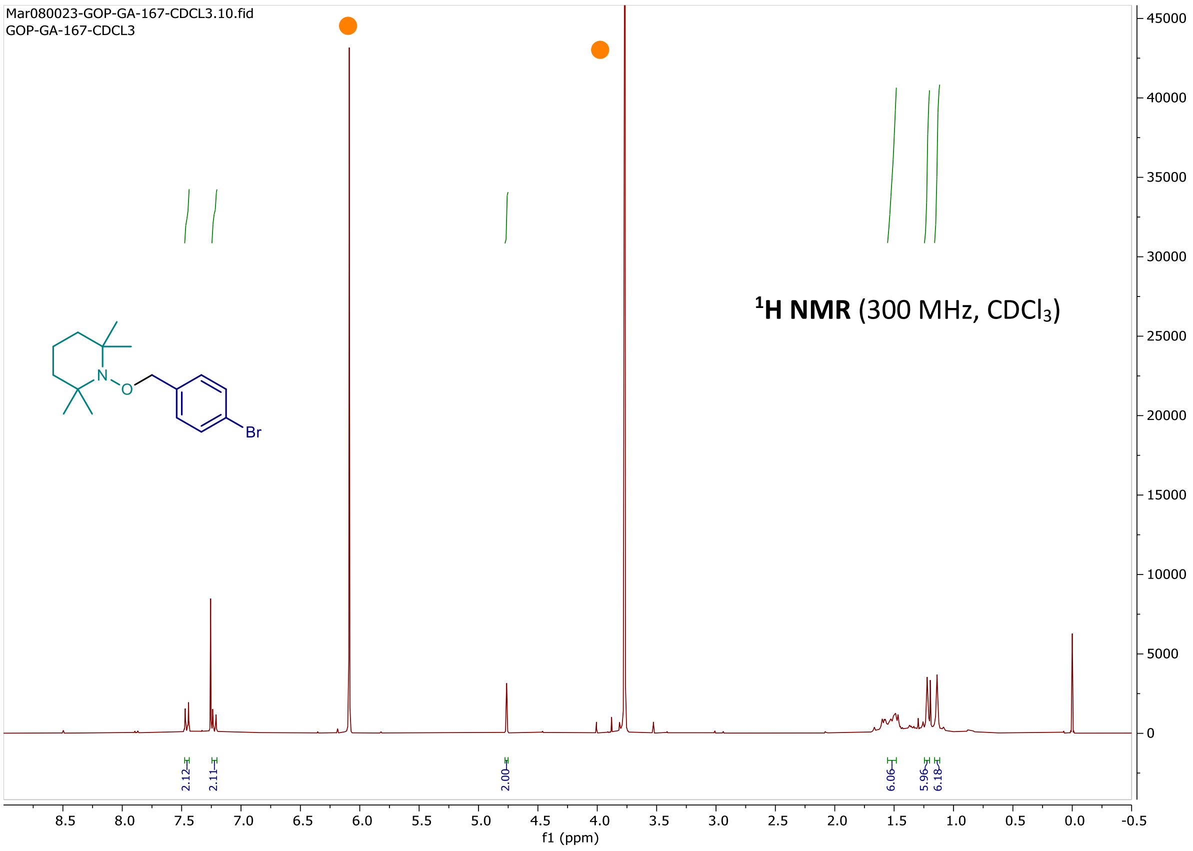

● 2 equiv of 1,3,5-trimethoxybenzene as internal standard

2,2,6,6-Tetramethyl-1-((1-tosylpiperidin-4-yl)oxy)piperidine (S23) ,crude reaction mixture

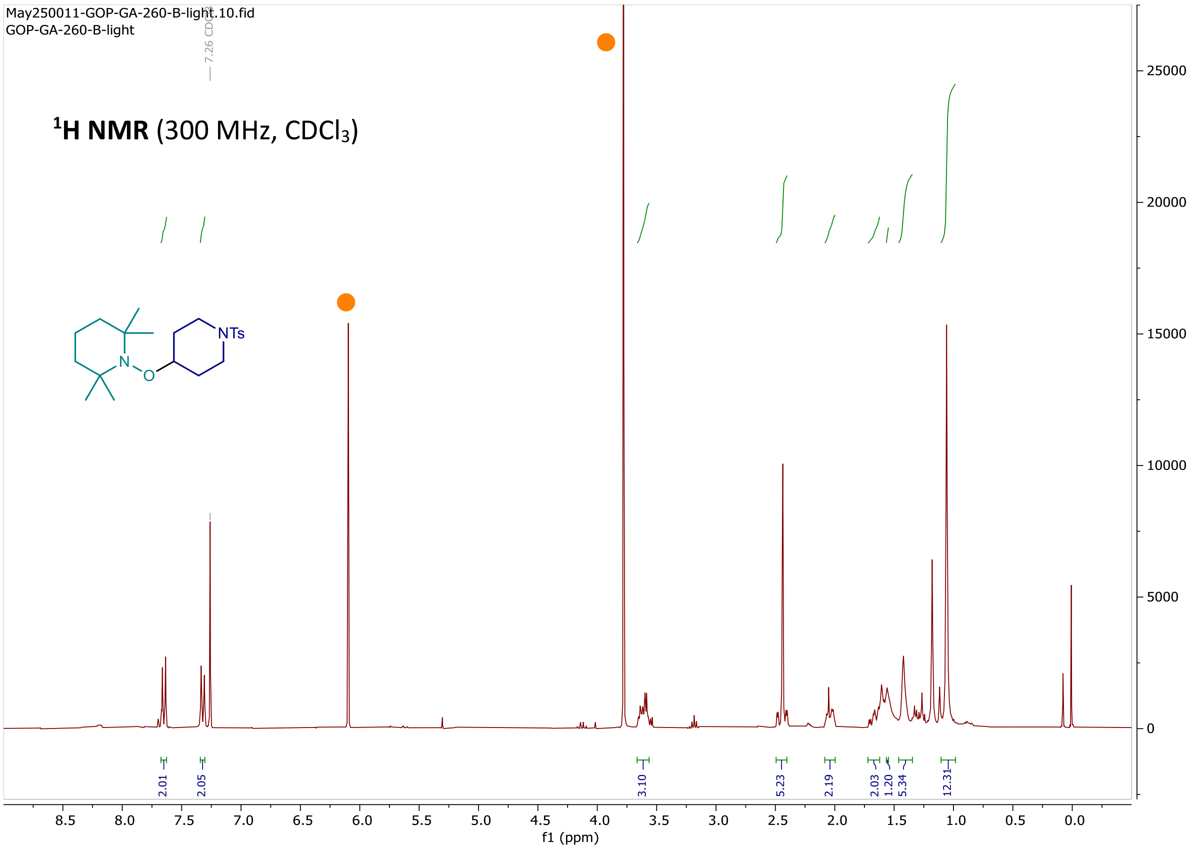

● 1 equiv of 1,3,5-trimethoxybenzene as internal standard

4-(2-(Phenylsulfonyl)ethyl)-1-tosylpiperidine (21)

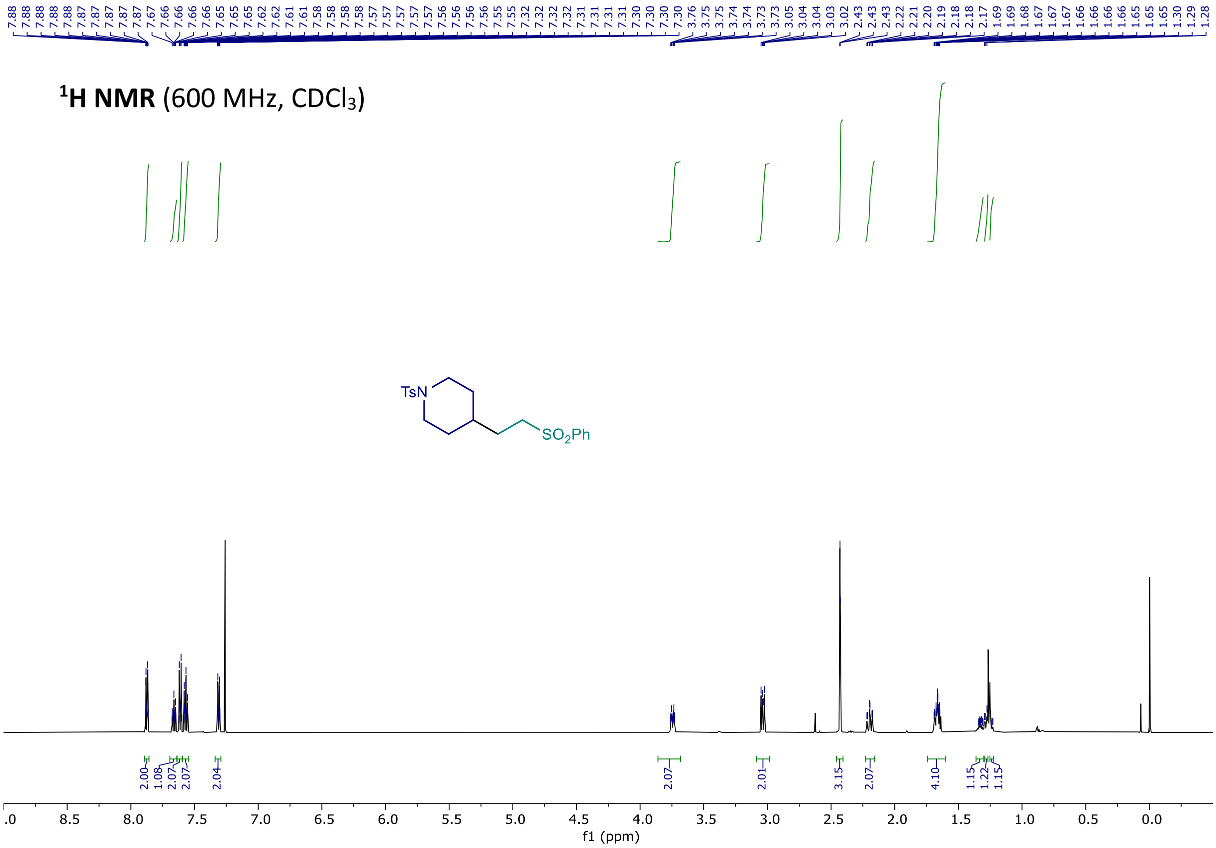

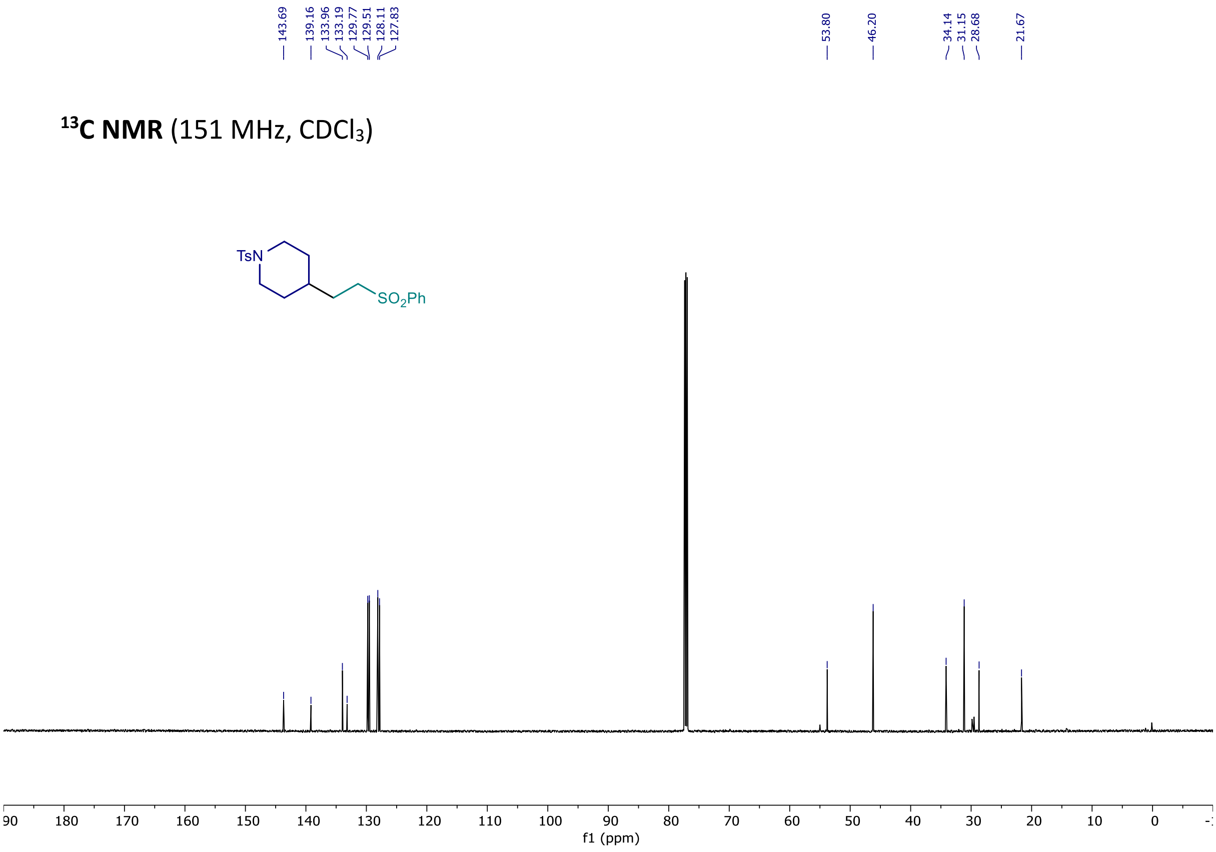

1-Tosylpiperidine (S24)

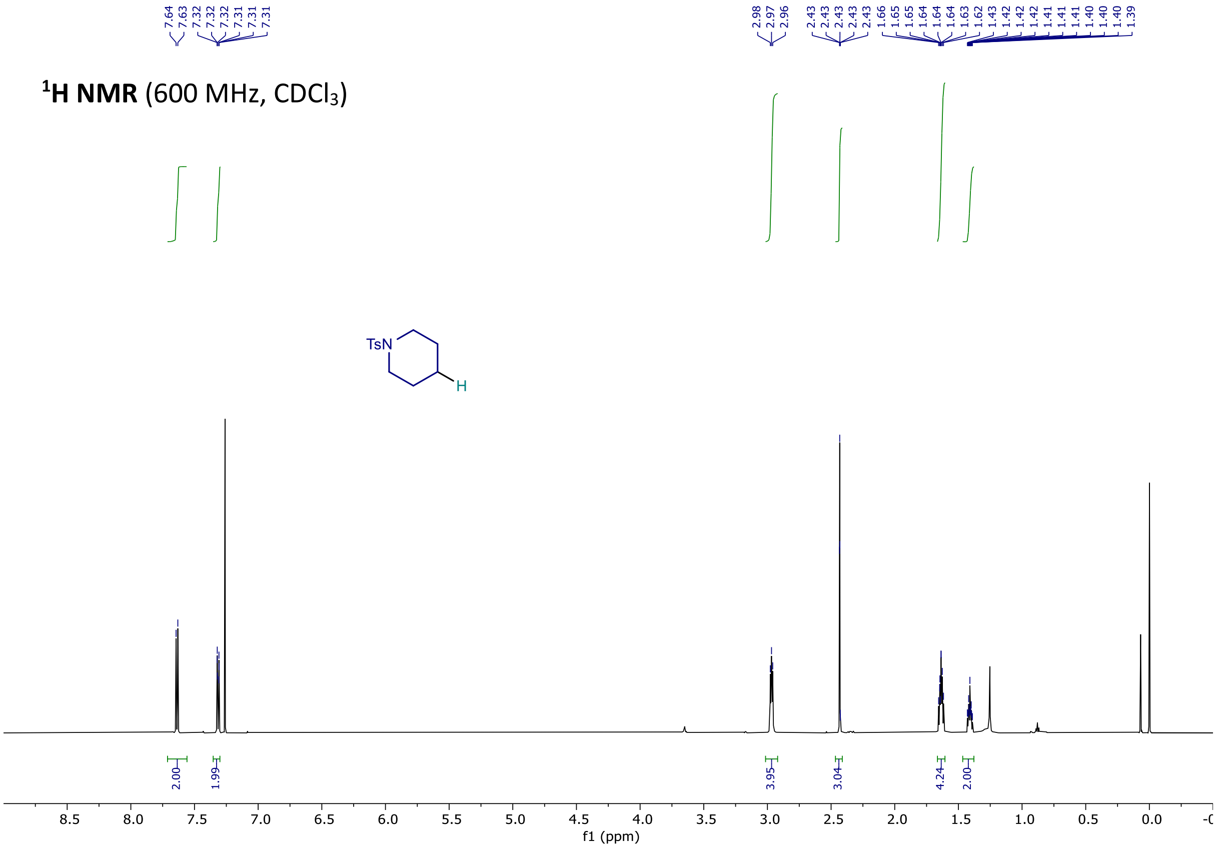

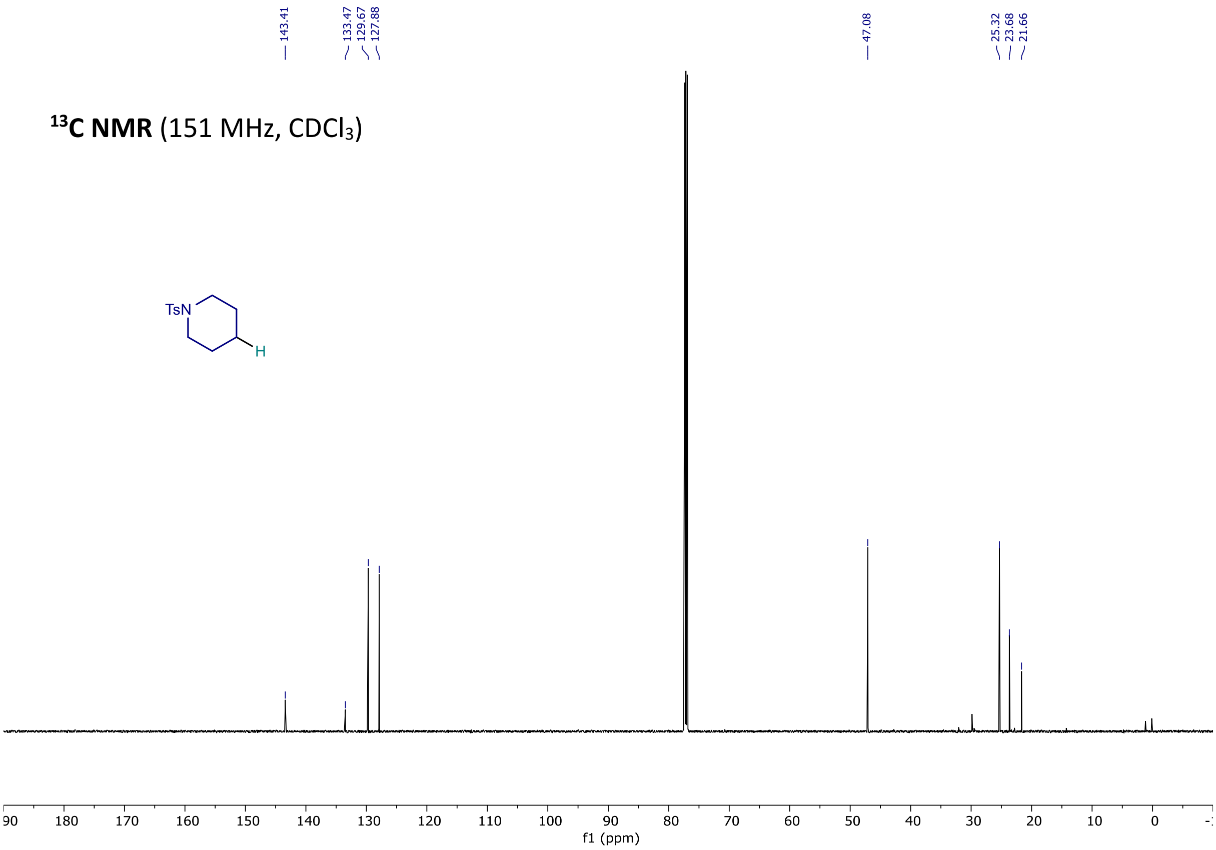

4-(2,2-Diphenylvinyl)-1-tosylpiperidine (S25)

^{1}H NMR (600 MHz, $CDCl_3$)

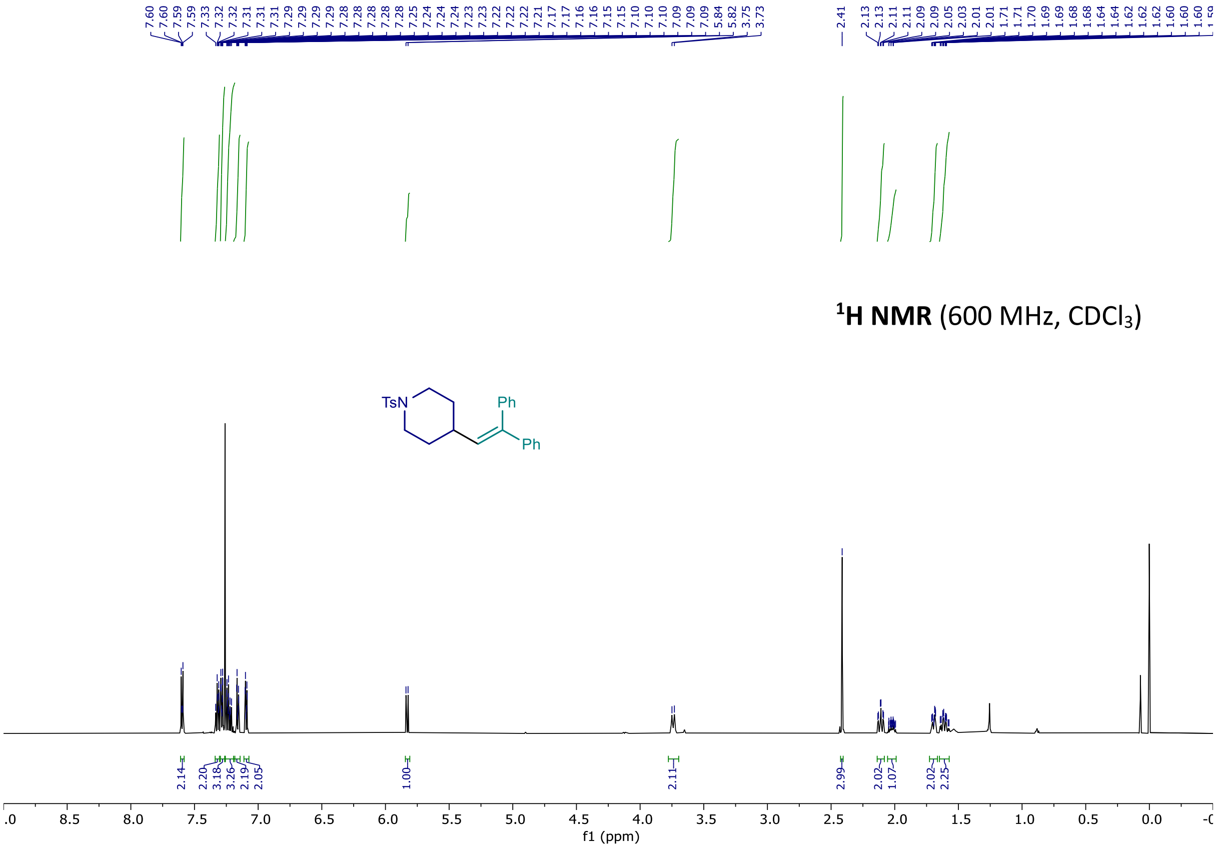

^{13}C NMR (151 MHz, $CDCl_3$)

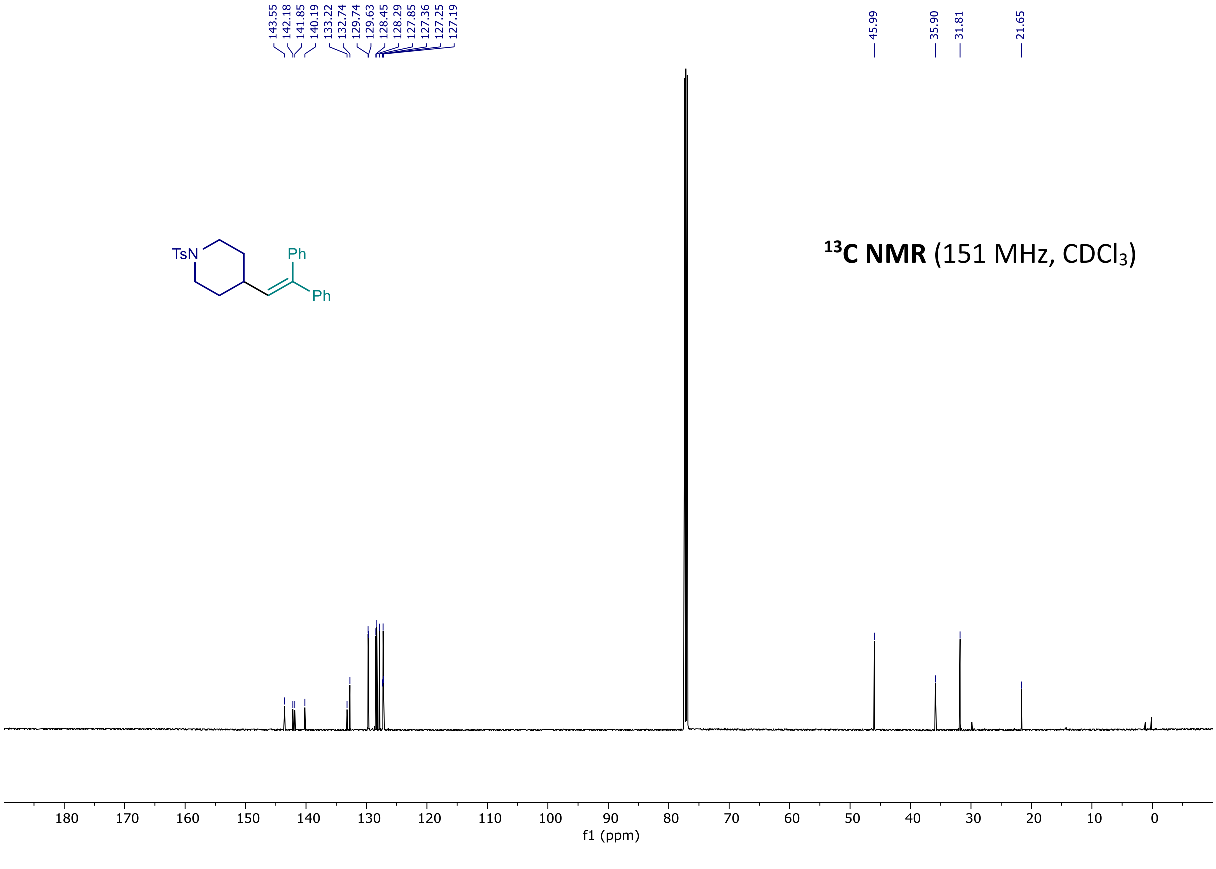

***tert*-Butyl 2-(1*H*-benzo[*d*]imidazol-1-yl)pyrrolidine-1-carboxylate (26)**

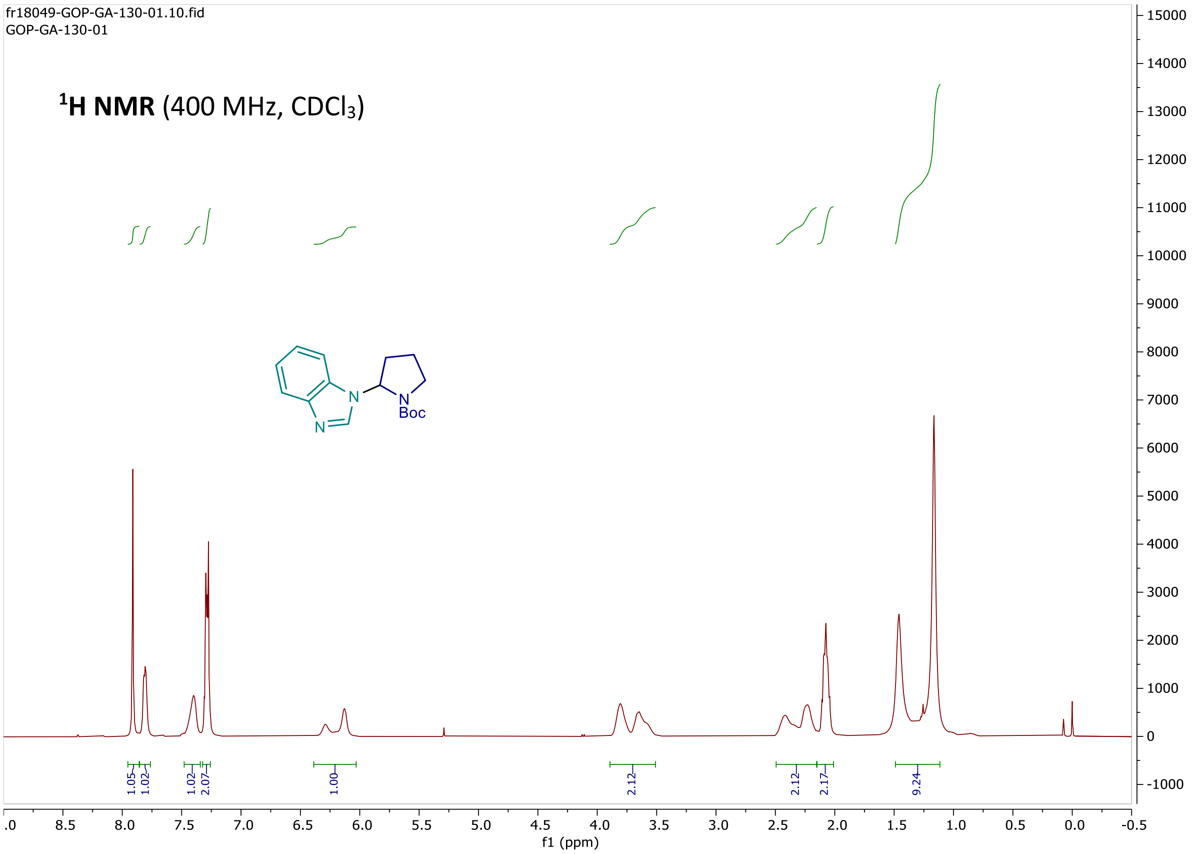

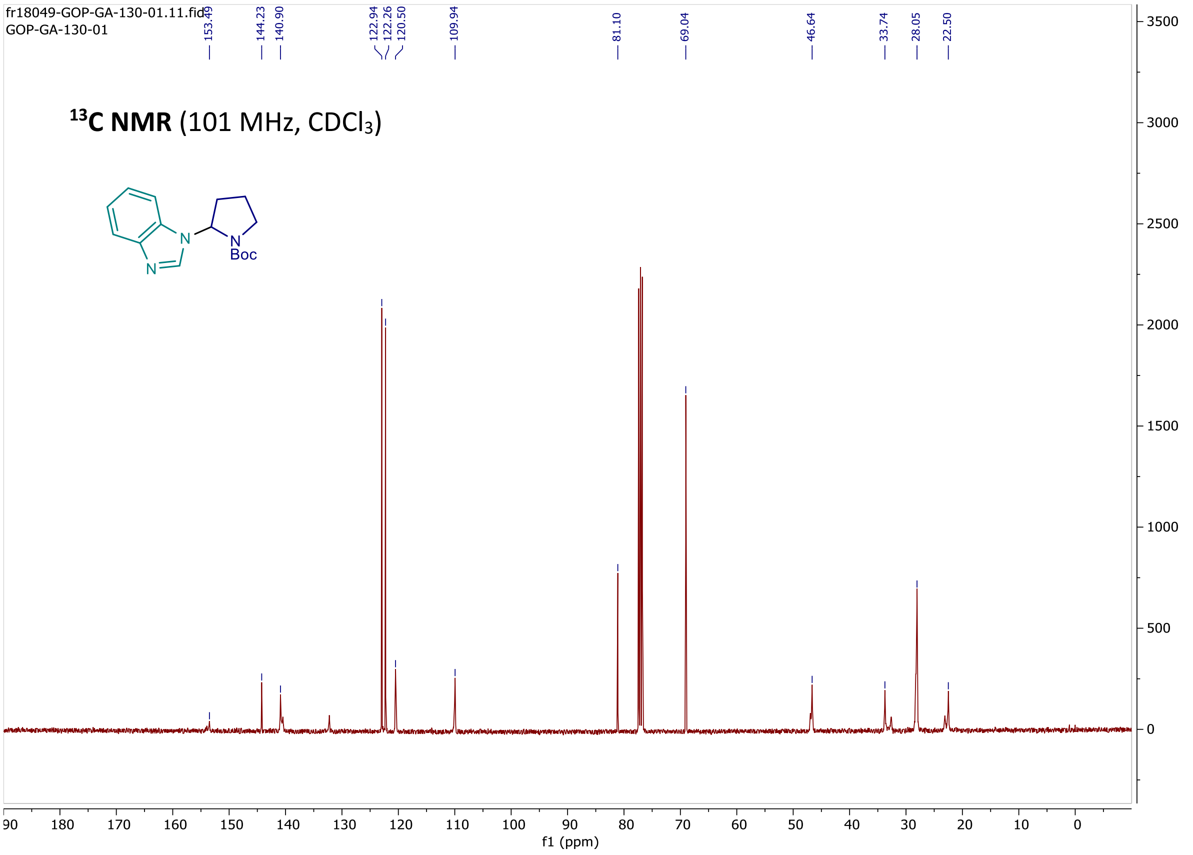

tert-Butyl 2-(N-methoxy-1_H_-benzo[_d_]imidazol-1-yl)pyrrolidine-1-carboxylate (N = 5/6 ca. 1:1, **28**)

Mixture of regioisomers, each of them has two rotamers. For simplicity in the NMR assignment, spectra were recorded at 353 K, temperature at which each pair of rotamers converges.

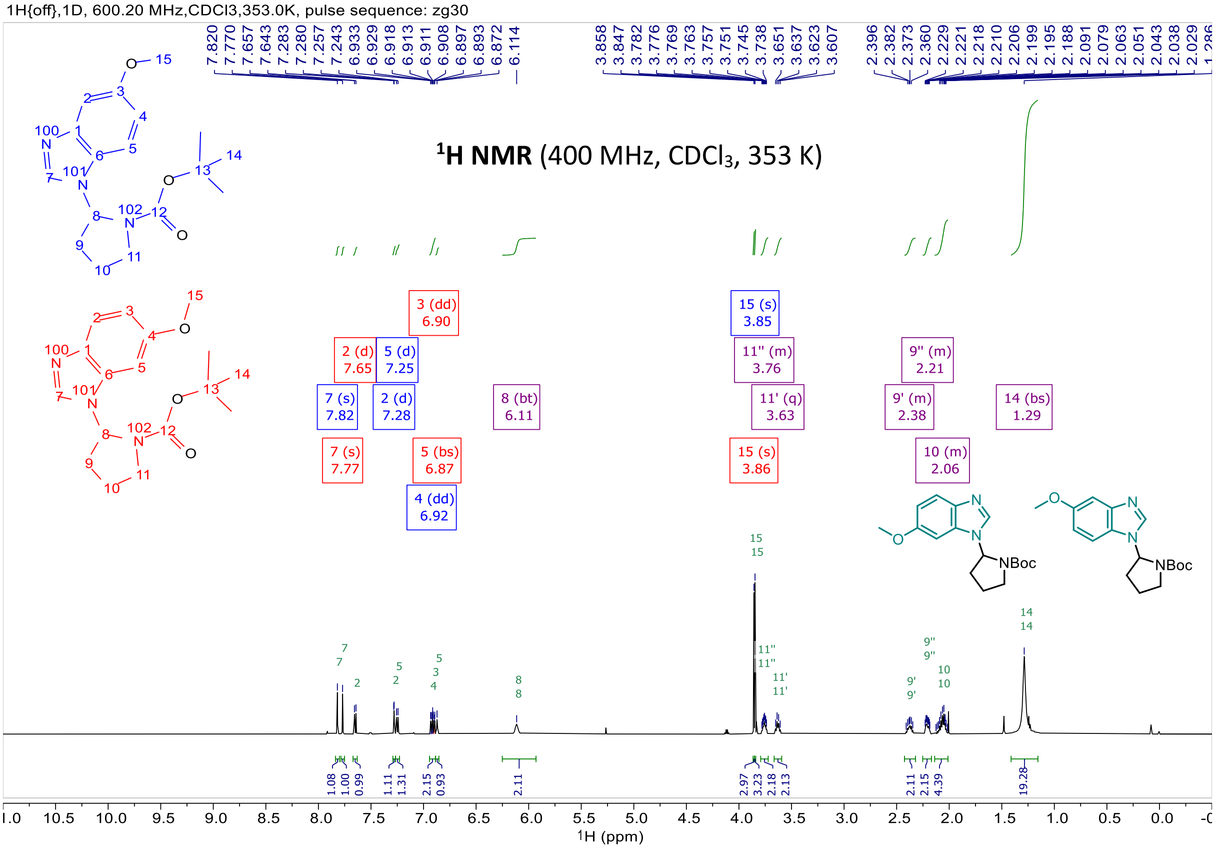

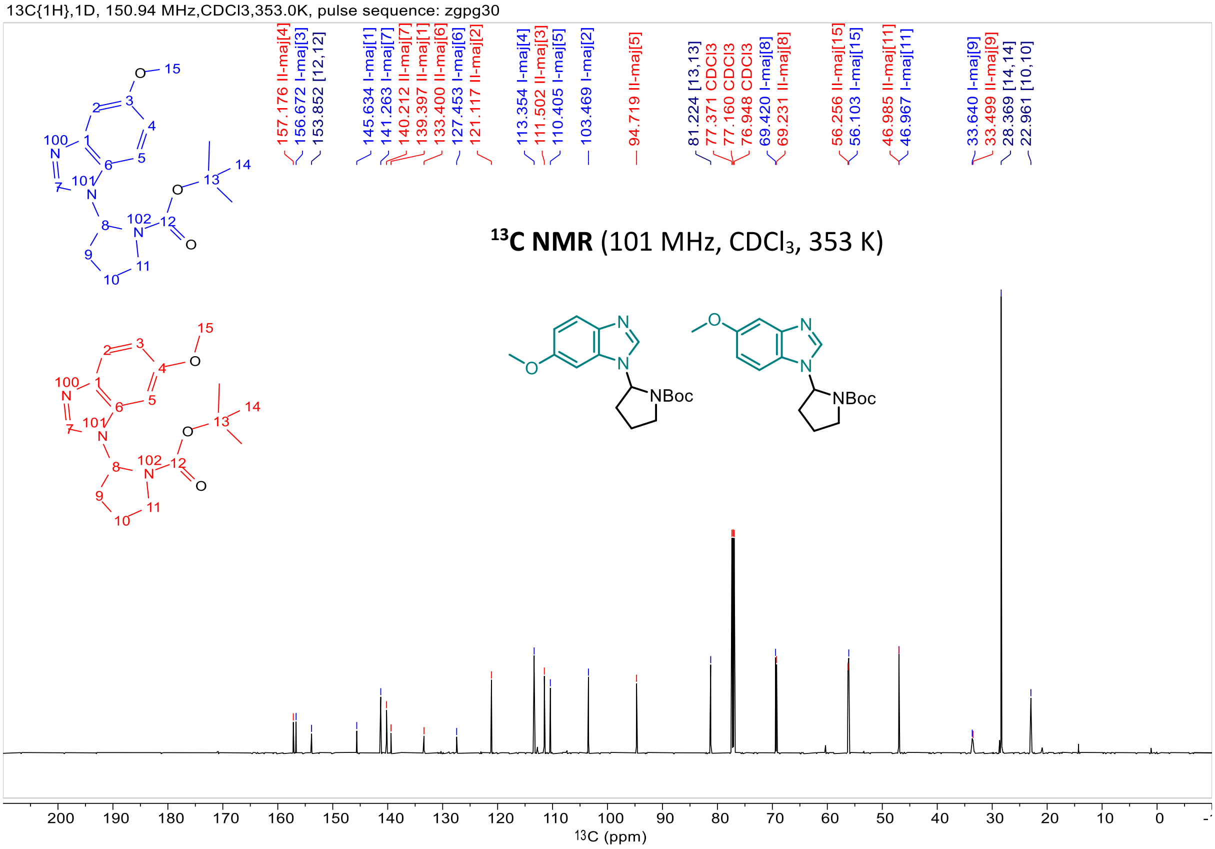

Variable-temperature NMR of **28**: rotamer signals of each regioisomers coalesce at high temperature. At lower temperatures the signals of each rotamer sharpen and can be resolved.

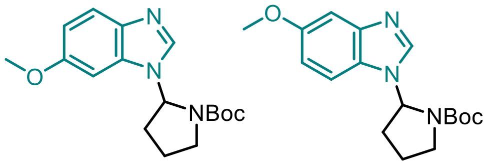

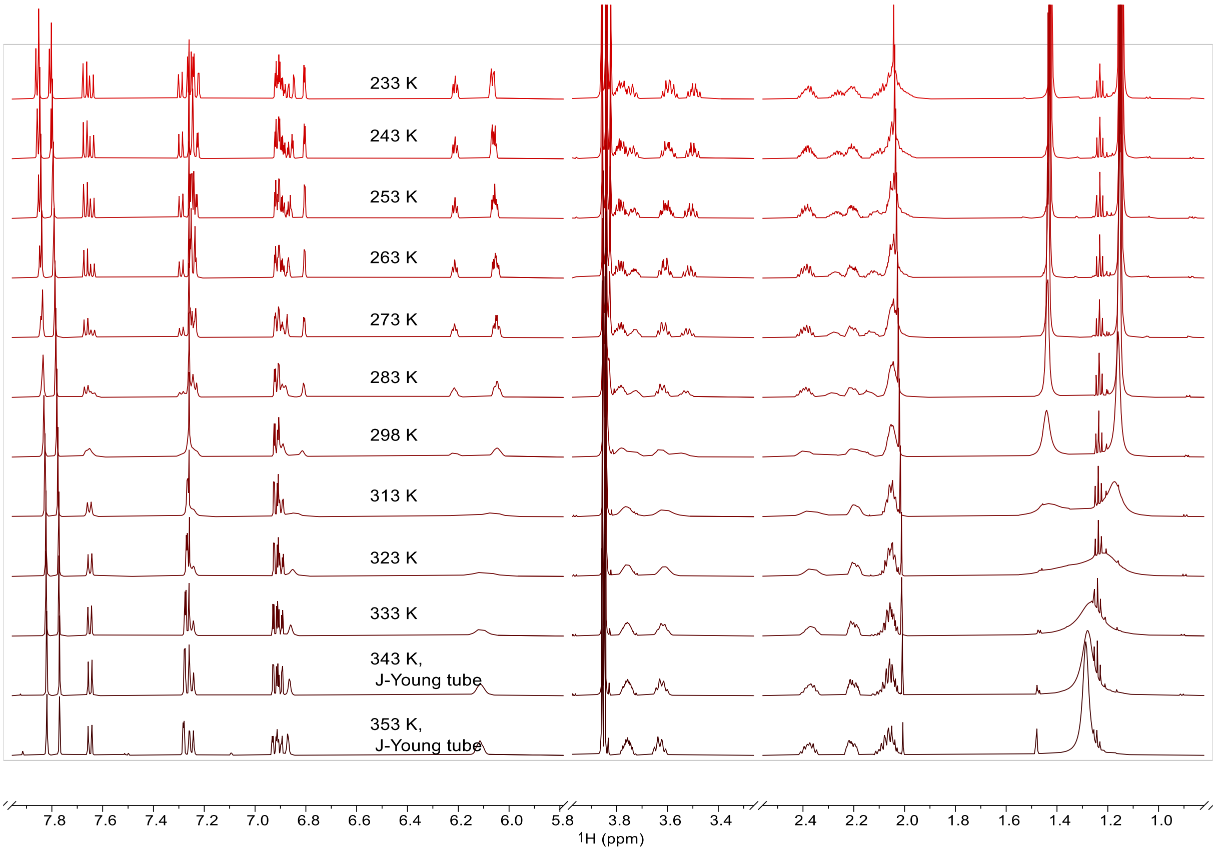

Benzyl (1-(1*H*-benzo[*d*]imidazol-1-yl)-2-phenylethyl)carbamate (29)

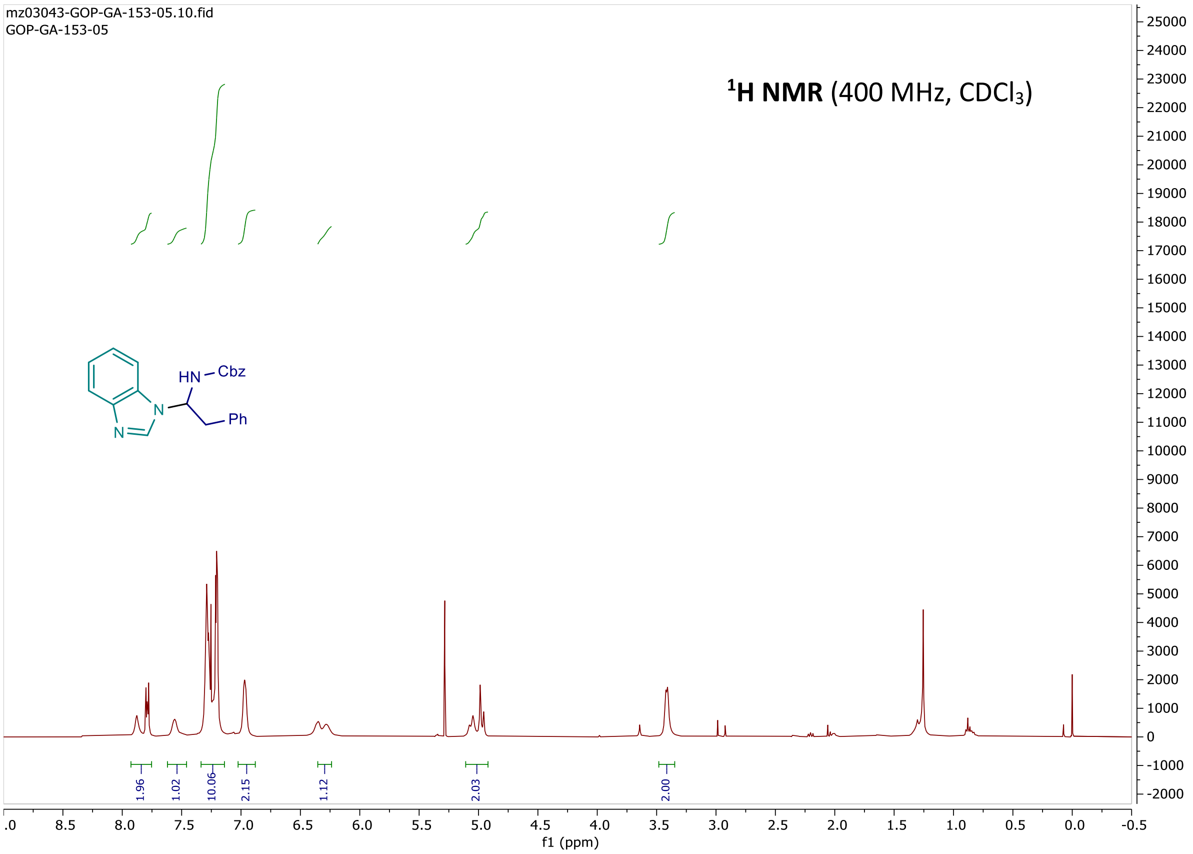

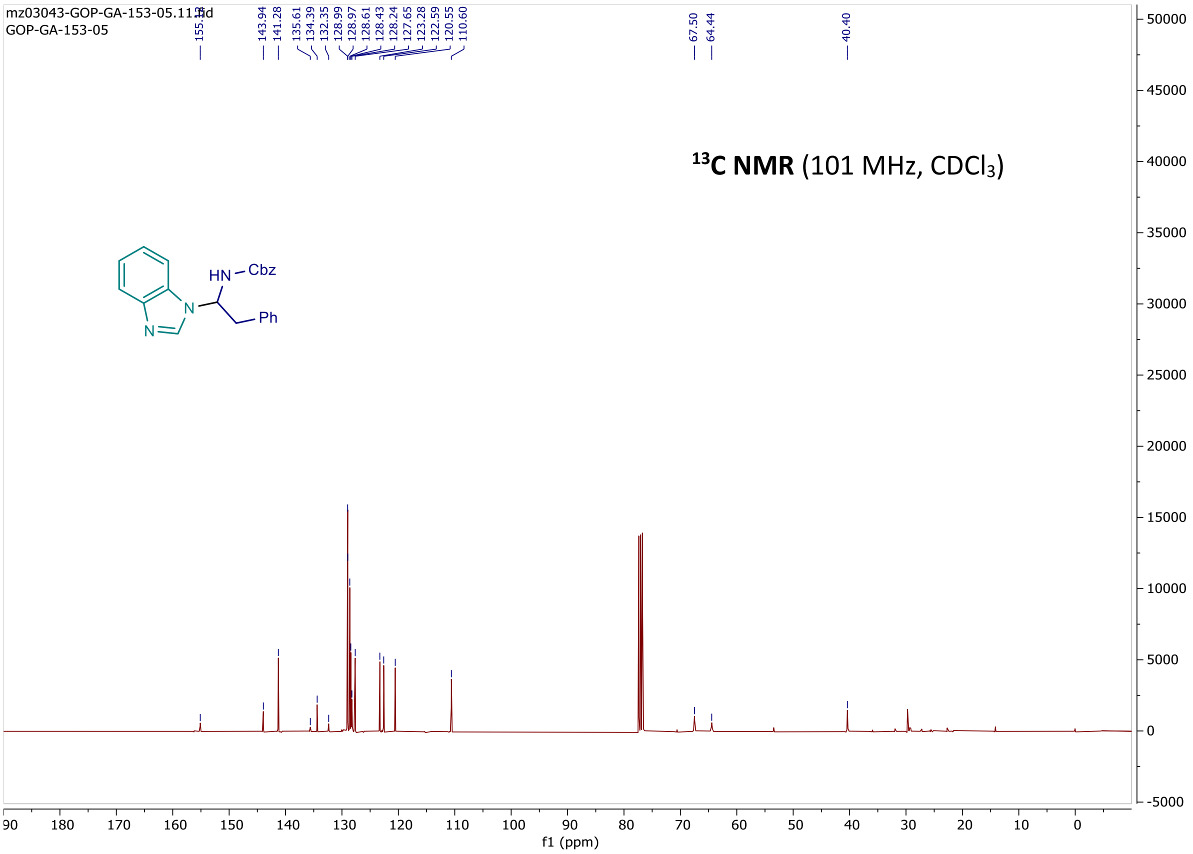

Benzyl (1-(1*H*-benzo[*d*]imidazol-1-yl)-2-methylpropyl)(methyl)carbamate (30)

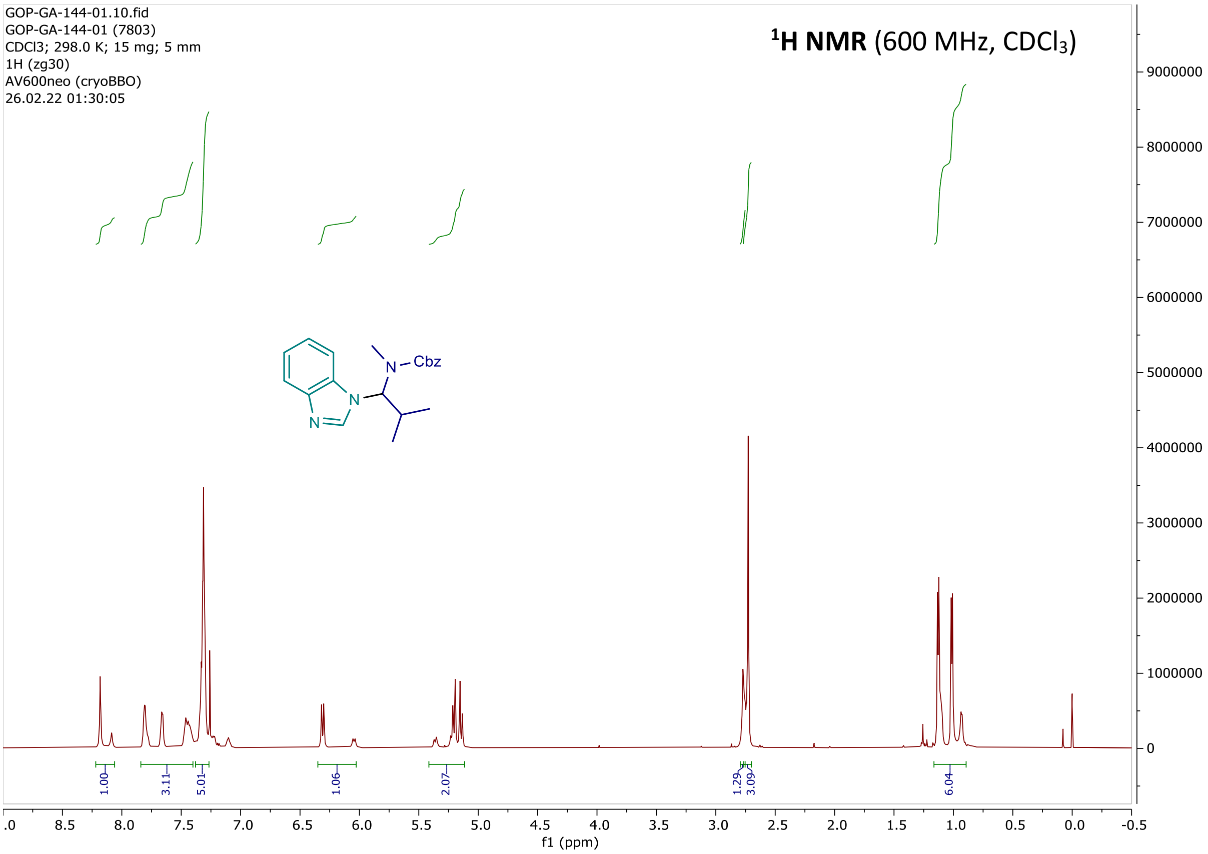

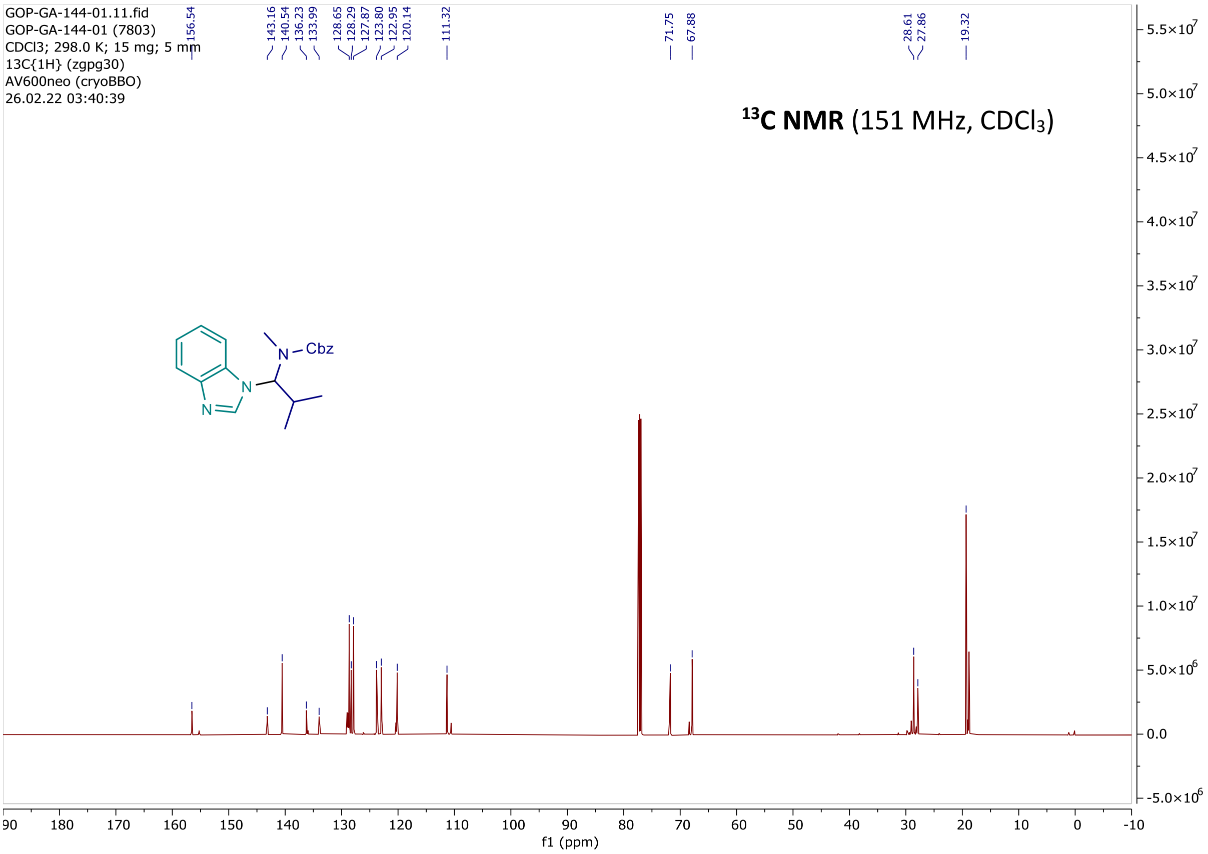

Benzyl 2-(1*H*-benzo[*d*]imidazol-1-yl)piperidine-1-carboxylate (31)

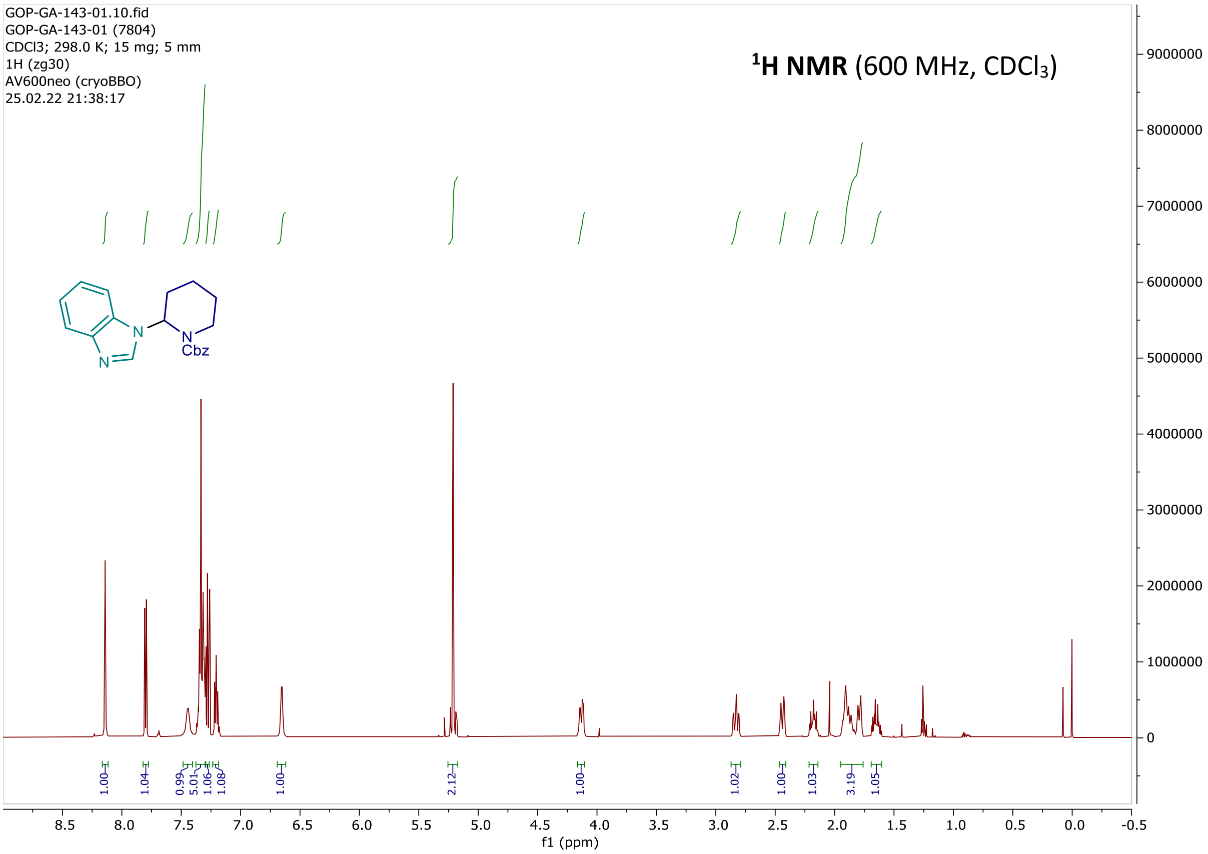

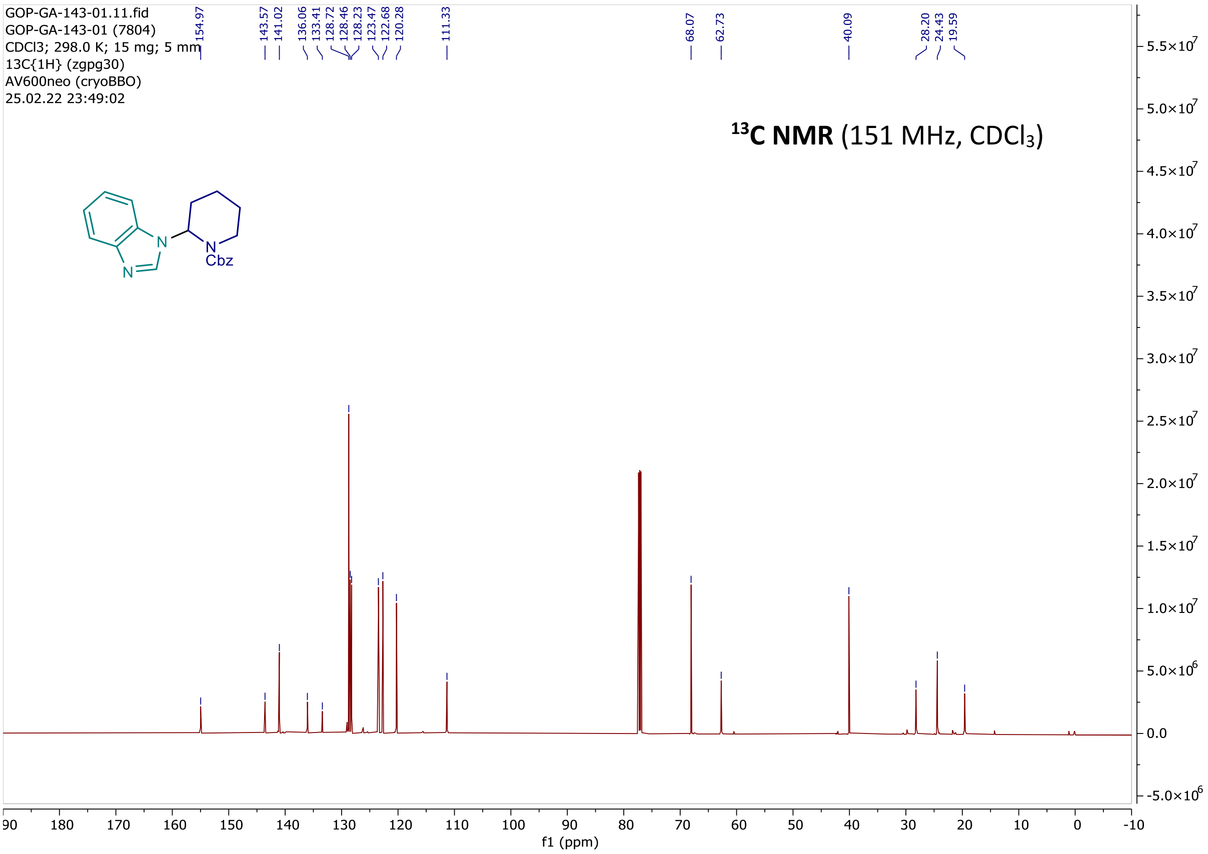

***tert*-Butyl 2-(4-(4,4,5,5-tetramethyl-1,3,2-dioxaborolan-2-yl)-1*H*-pyrazol-1-yl)pyrrolidine-1-carboxylate** (**32**)

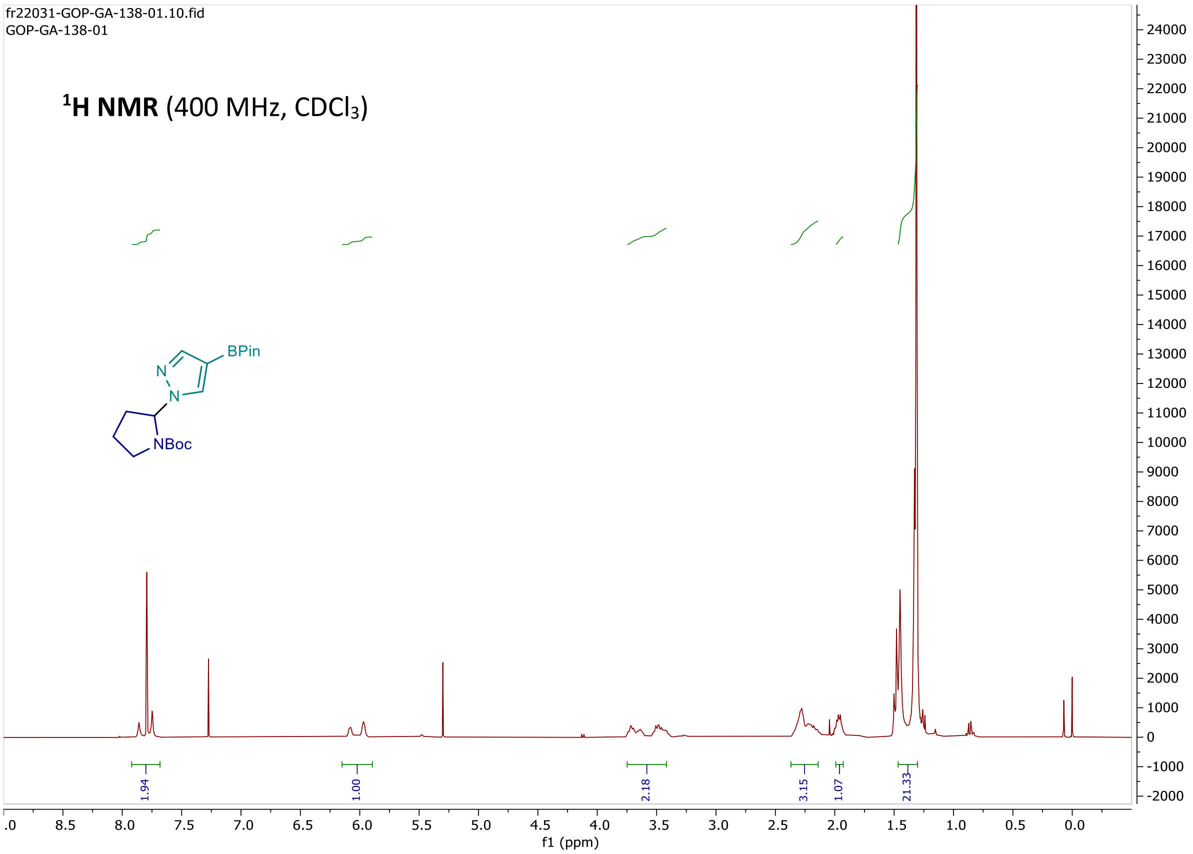

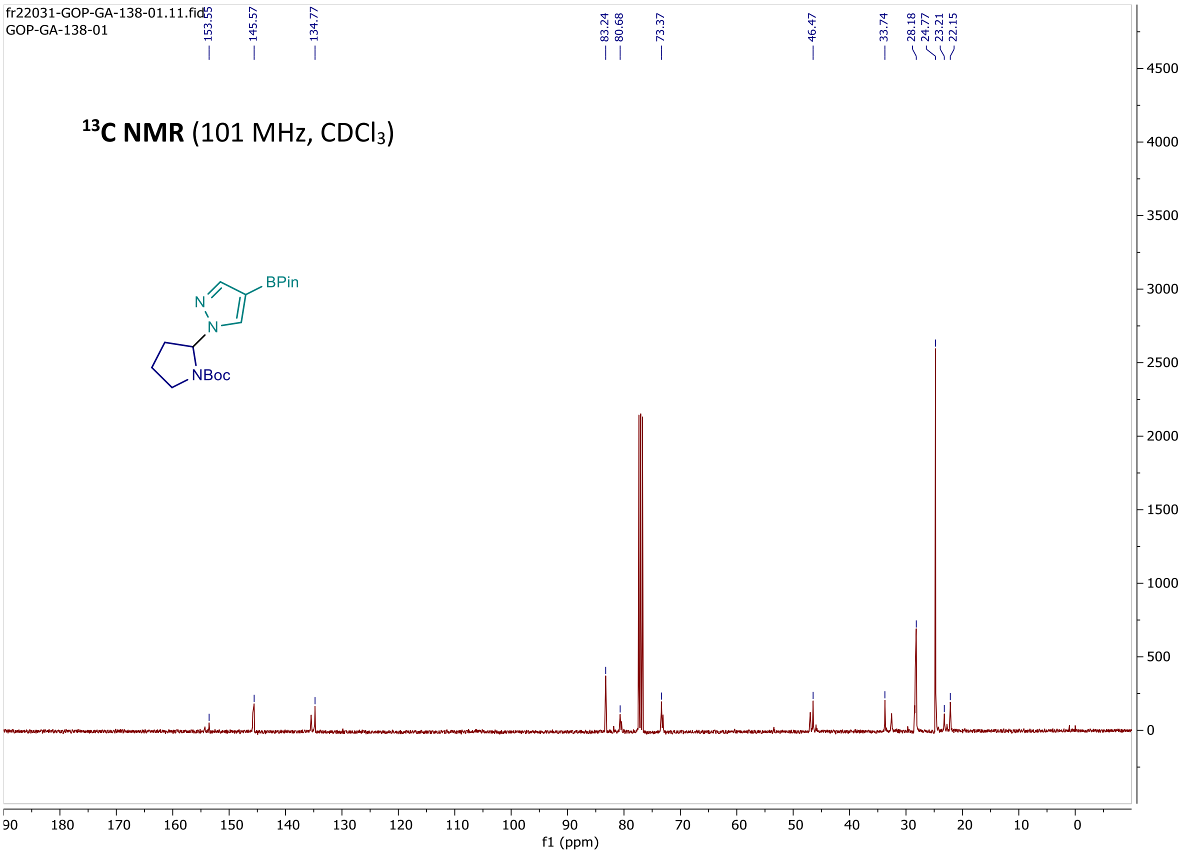

Benzyl 2-(4-(4,4,5,5-tetramethyl-1,3,2-dioxaborolan-2-yl)-1*H*-pyrazol-1-yl)piperidine-1-carboxylate (33)

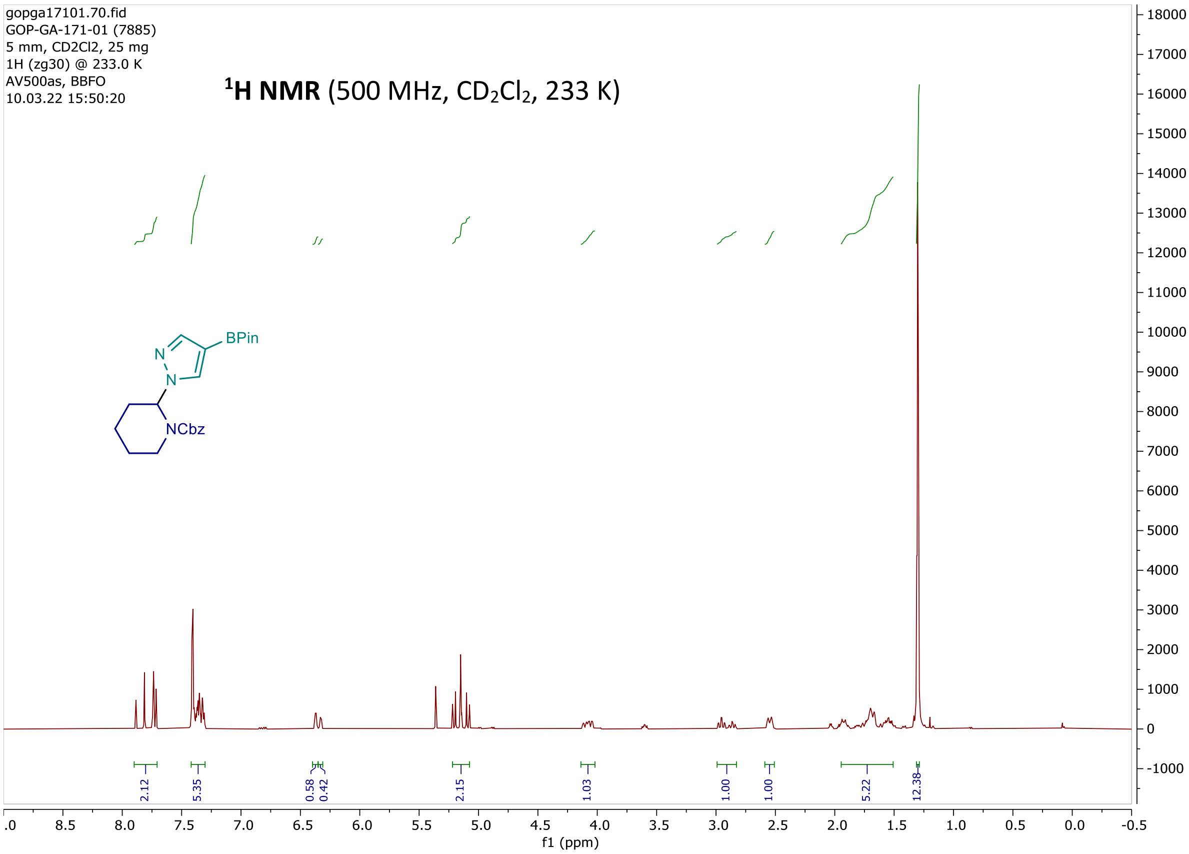

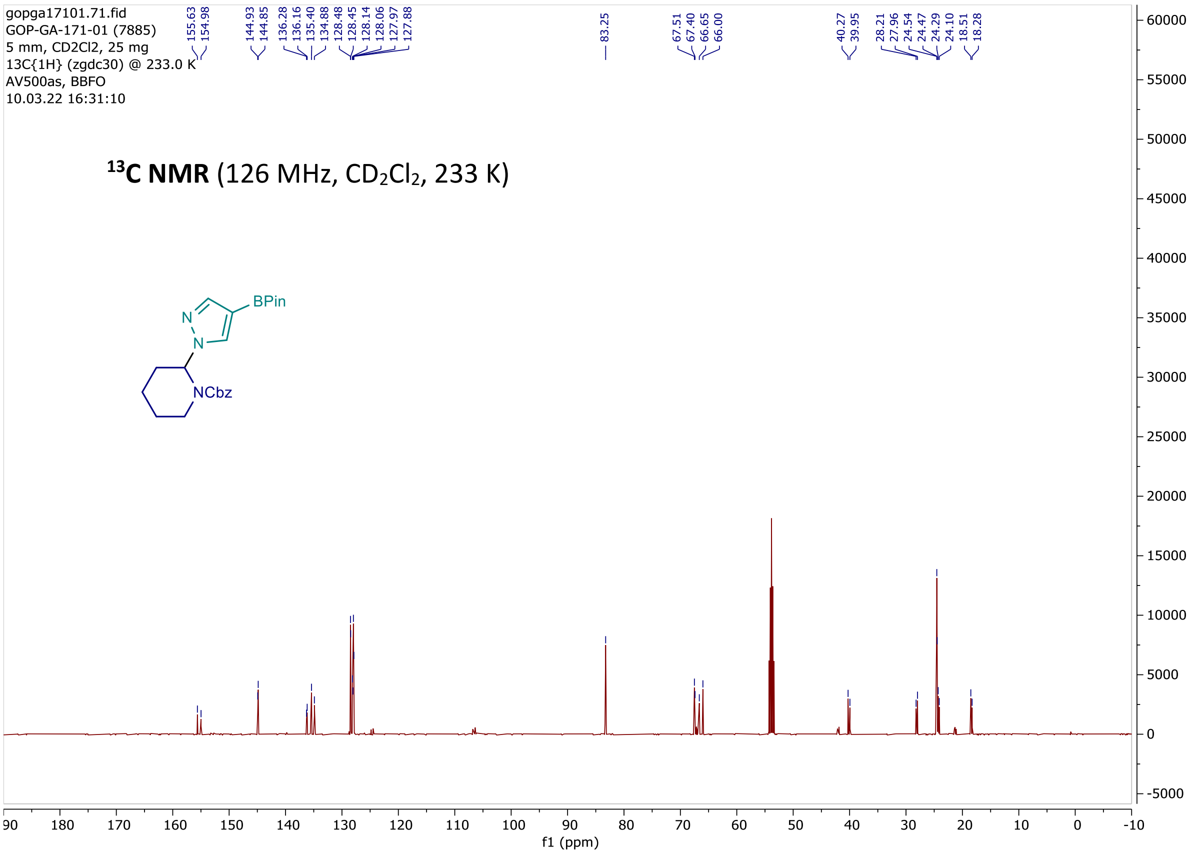

tert-Butyl (1-(1H-benzo[d]imidazol-1-yl)-2-methylpropyl)carbamate (34)

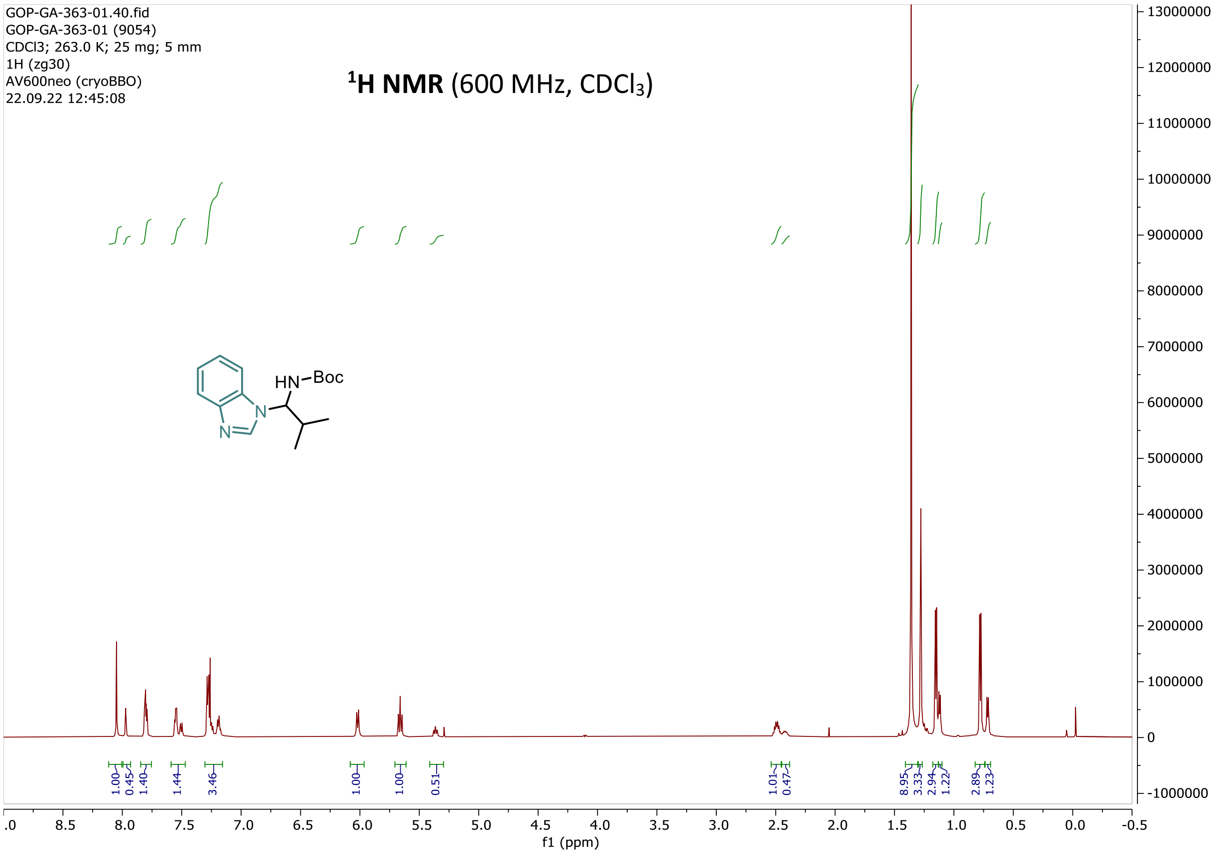

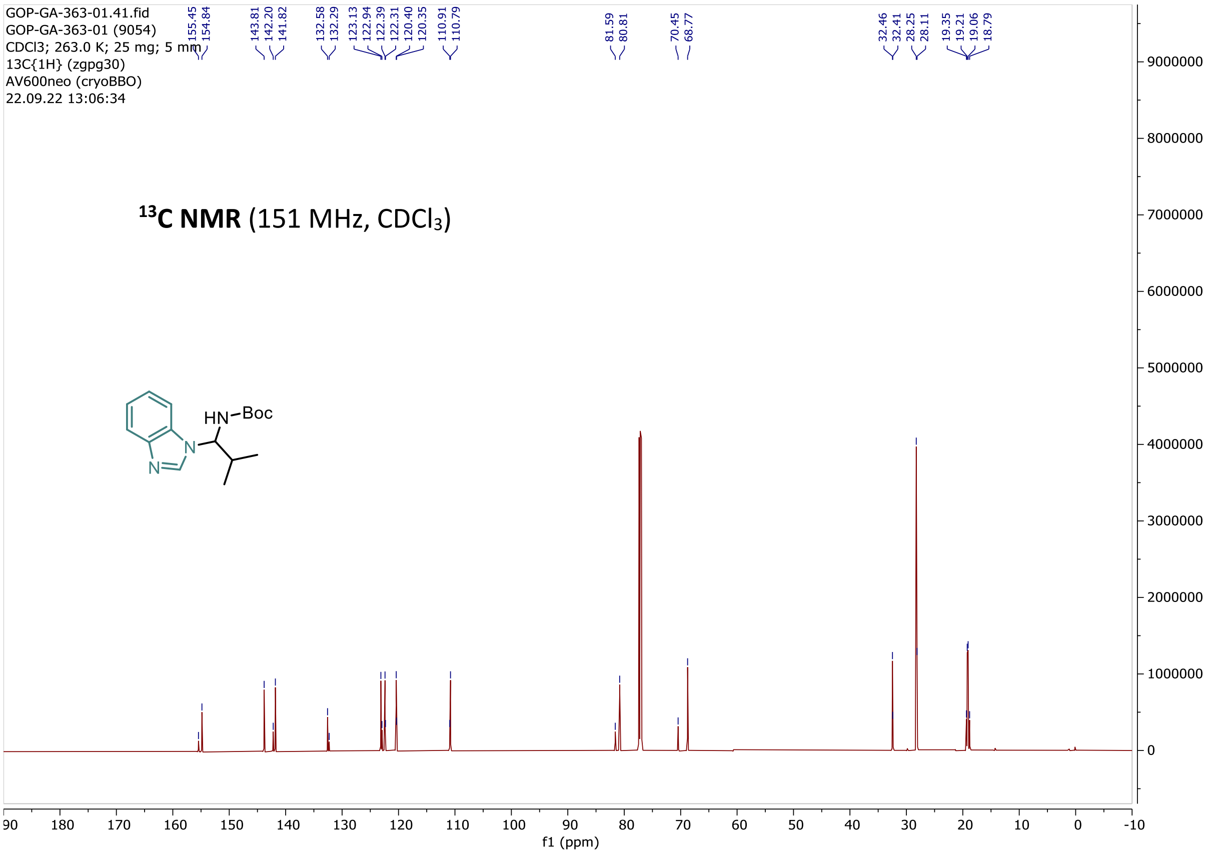

tert-Butyl (1-(1*H*-benzo[*d*]imidazol-1-yl)-3-methylbutyl)carbamate (35)

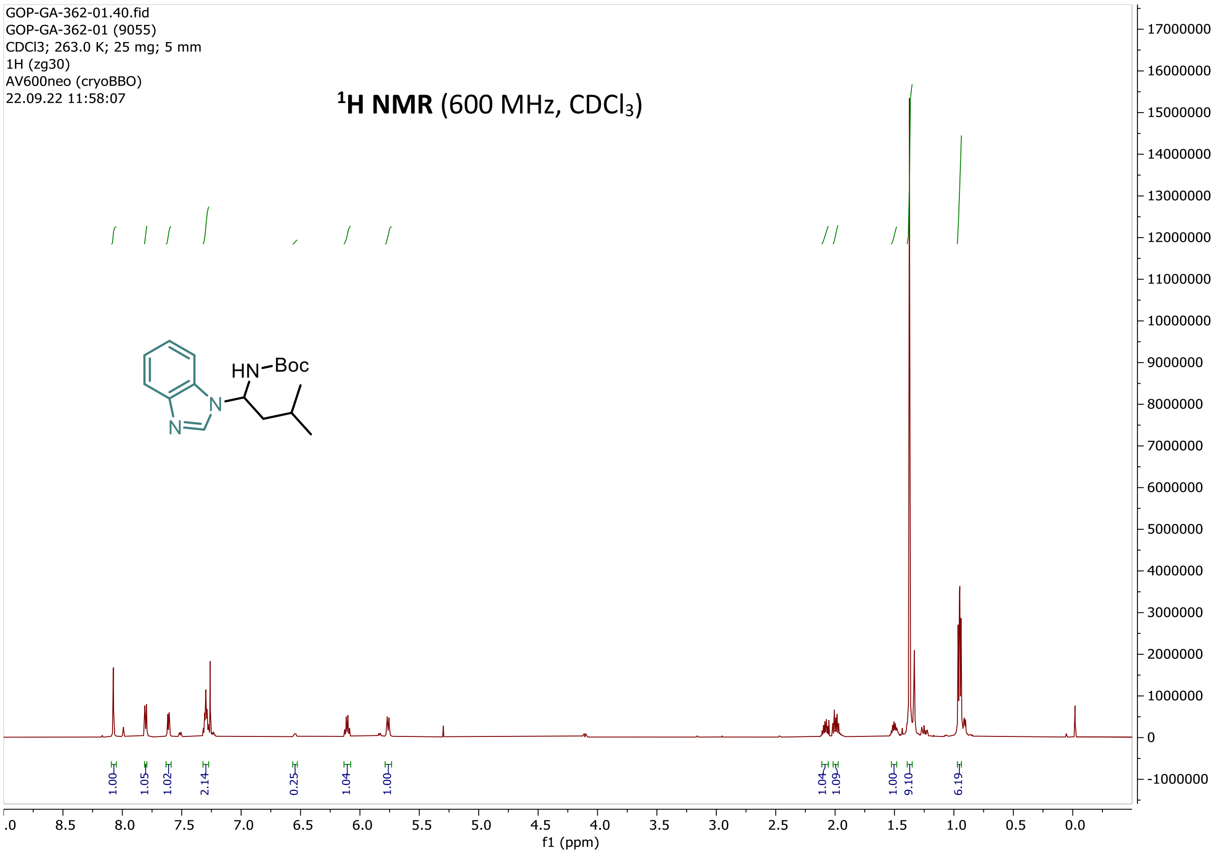

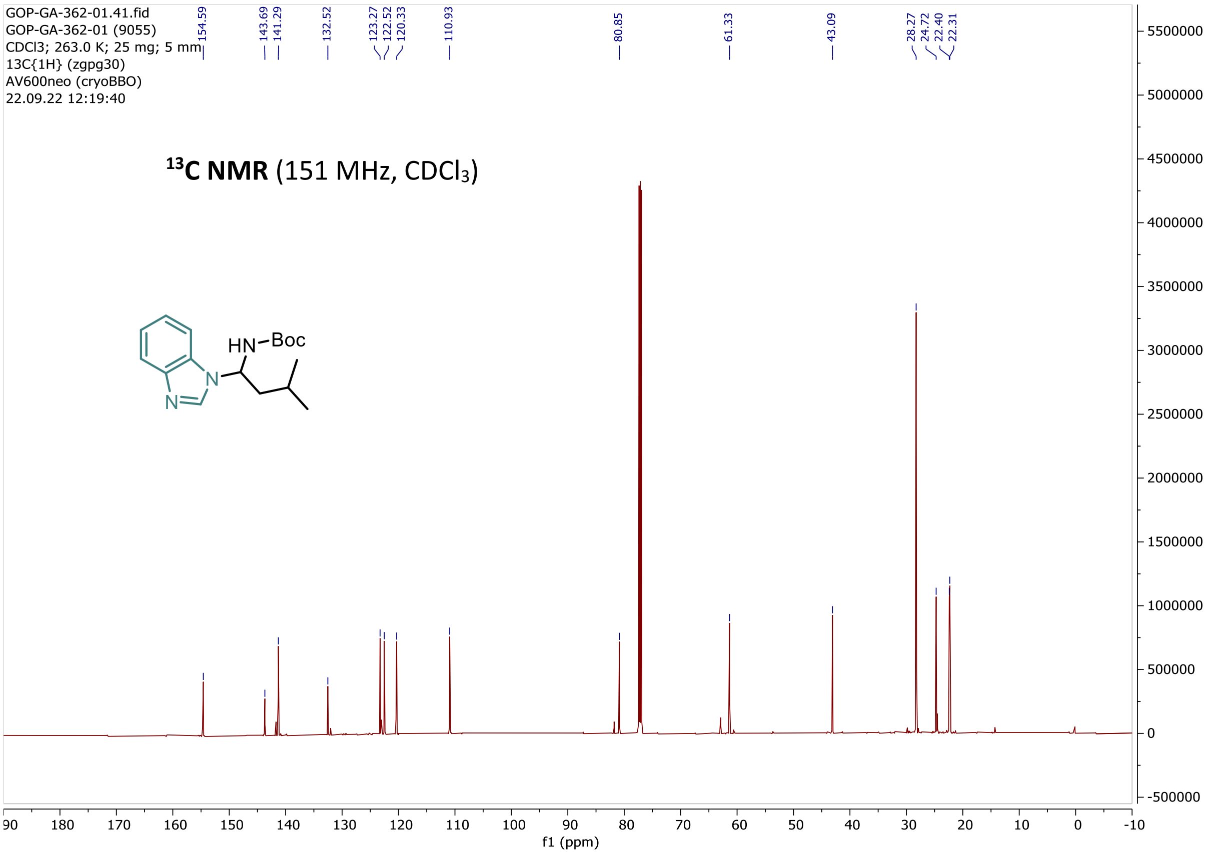

tert-Butyl 4-(1H-benzo[d]imidazol-1-yl)-4-((tert-butoxycarbonyl)amino)butanoate (36)

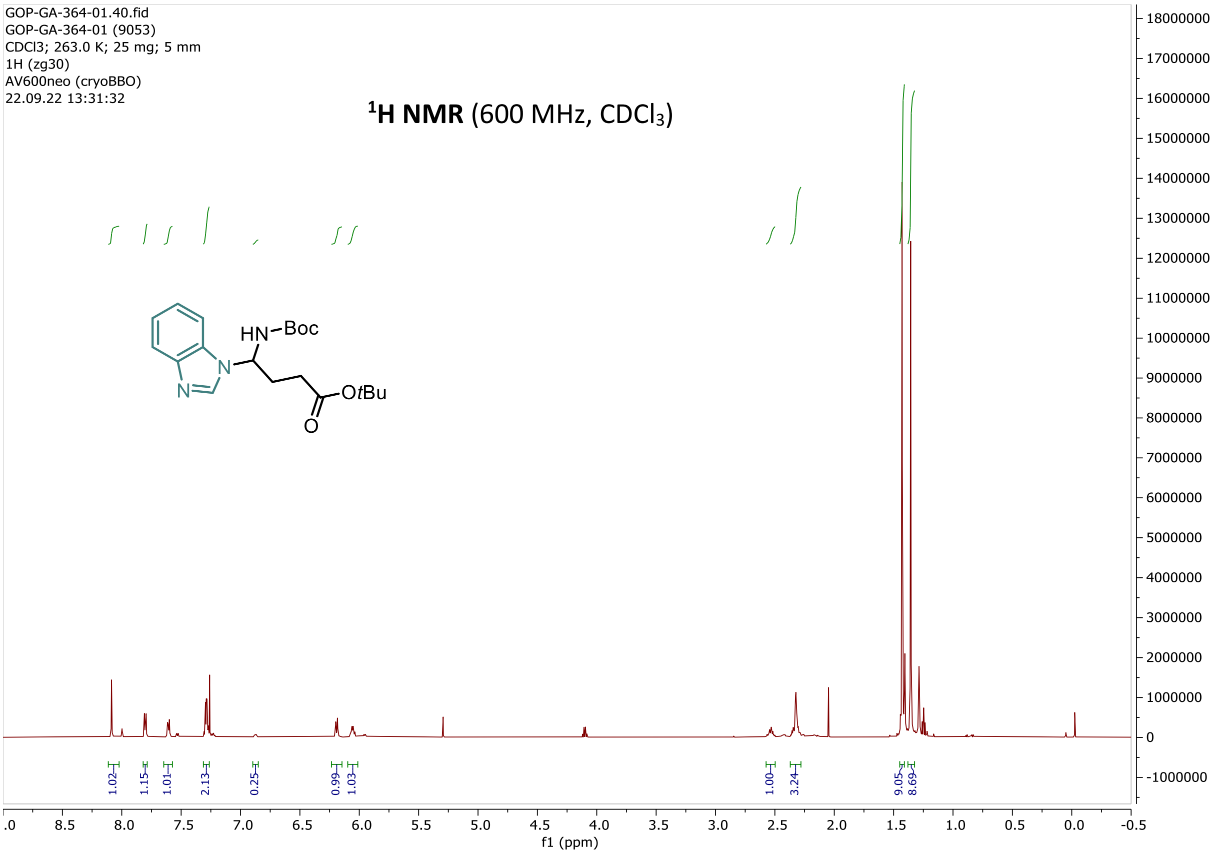

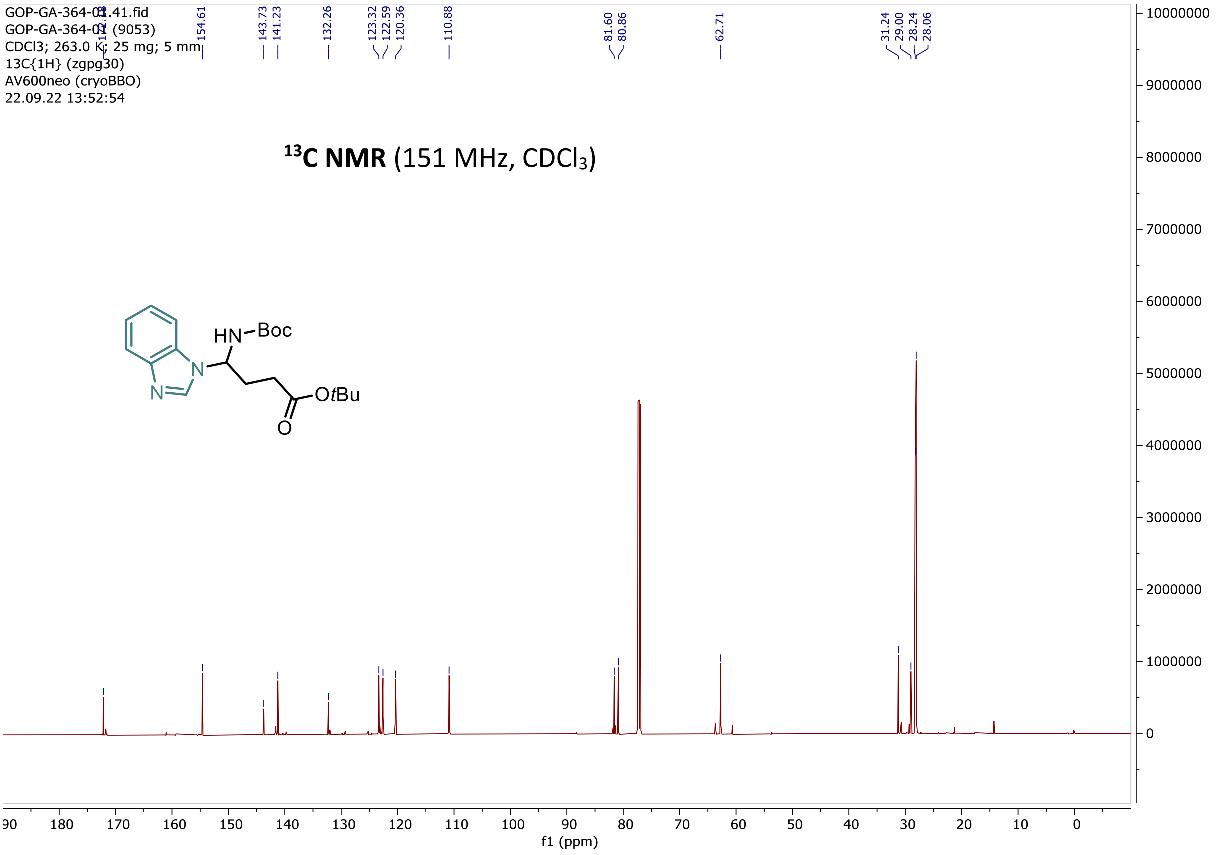

***tert*-Butyl 2-(1*H*-1,2,4-triazol-1-yl)pyrrolidine-1-carboxylate** (1:1 rotamers, **37**)

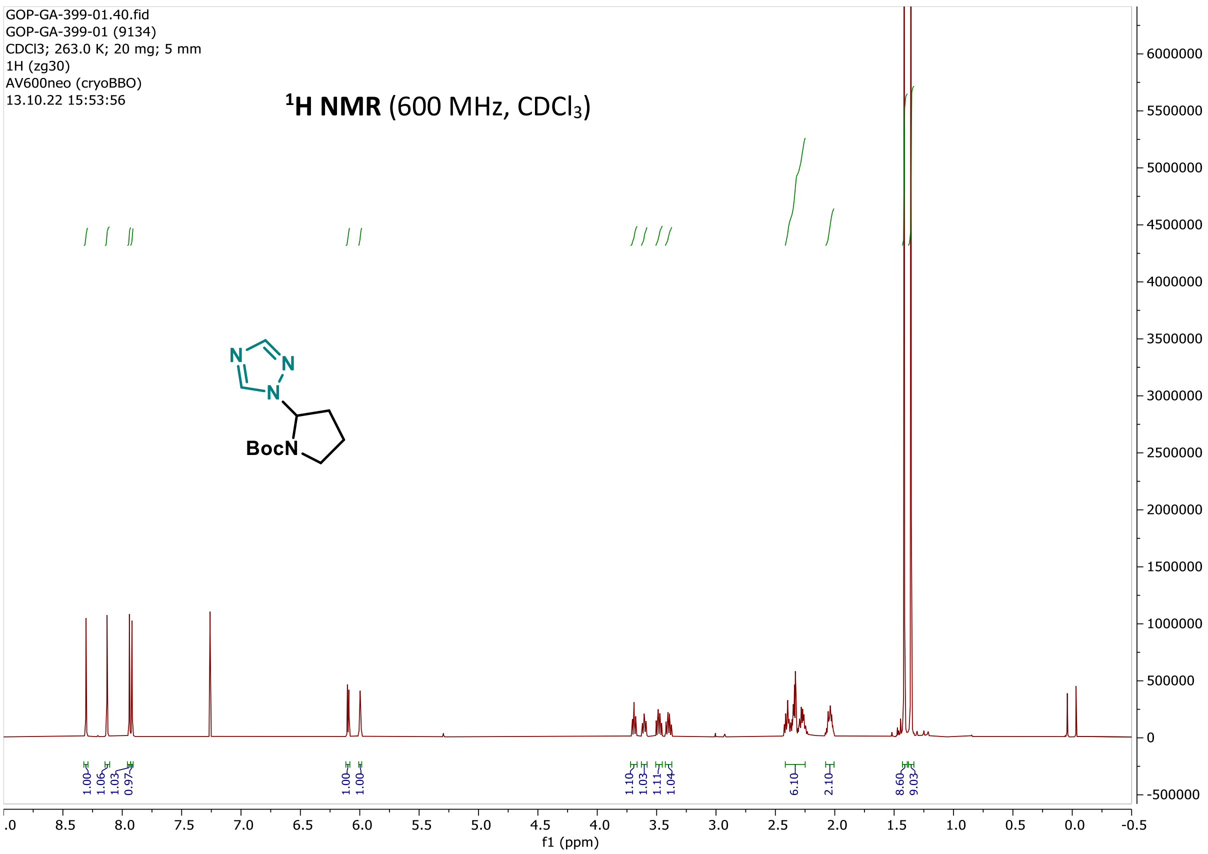

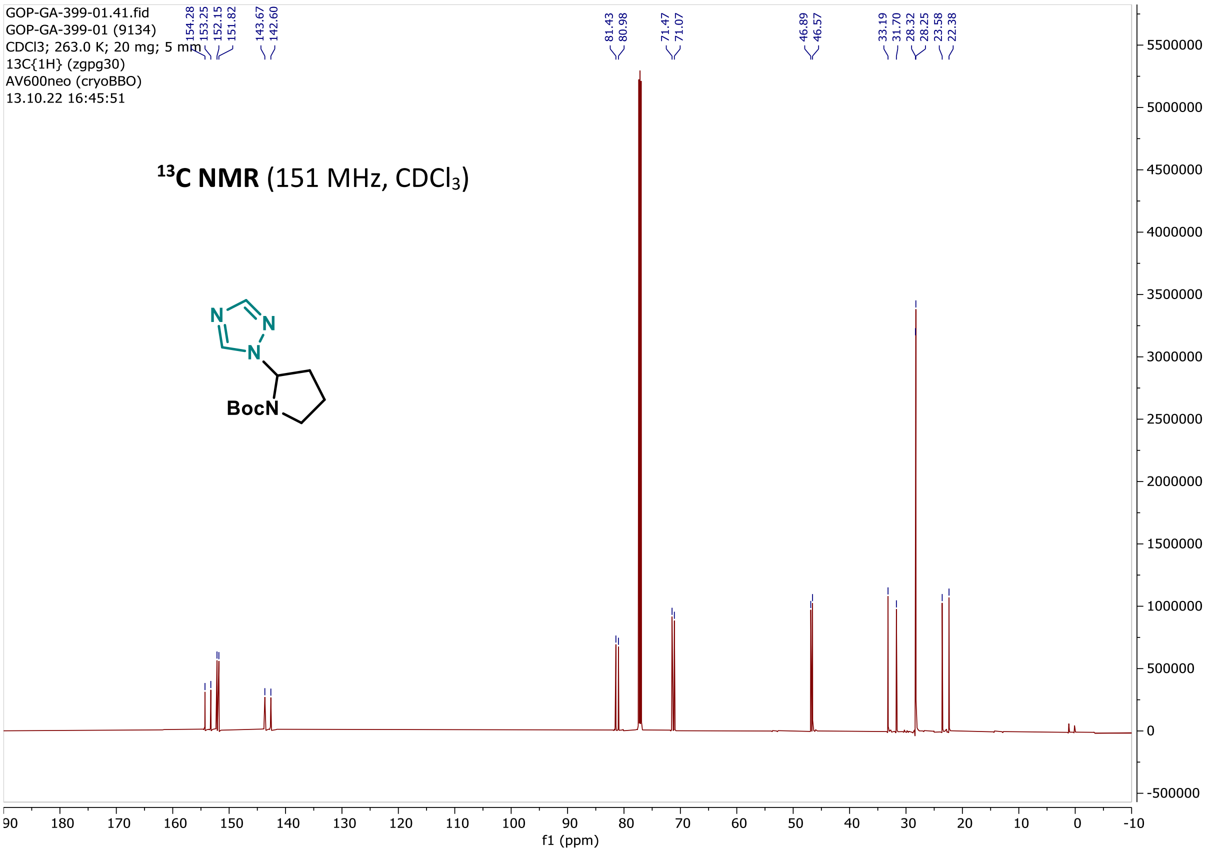

tert-Butyl 2-(4,5-diphenyl-1_H_-imidazol-1-yl)pyrrolidine-1-carboxylate (38)

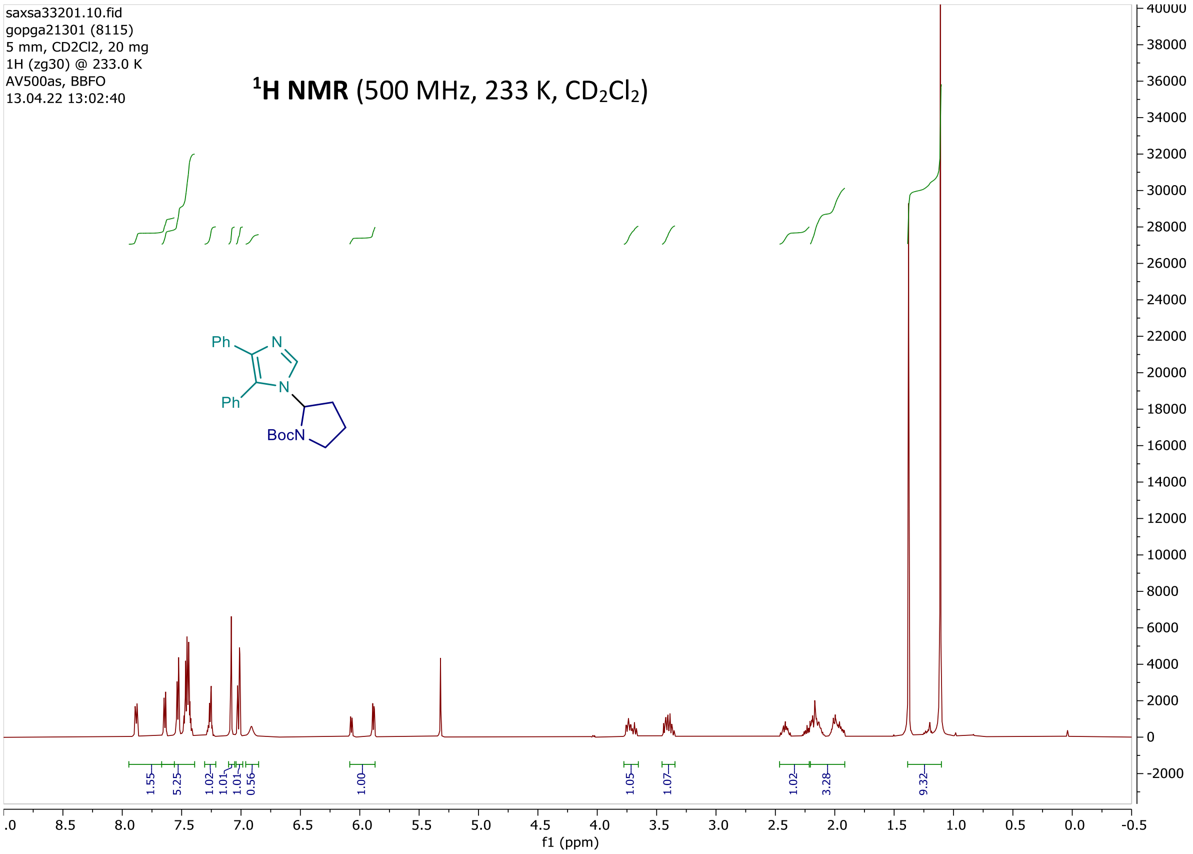

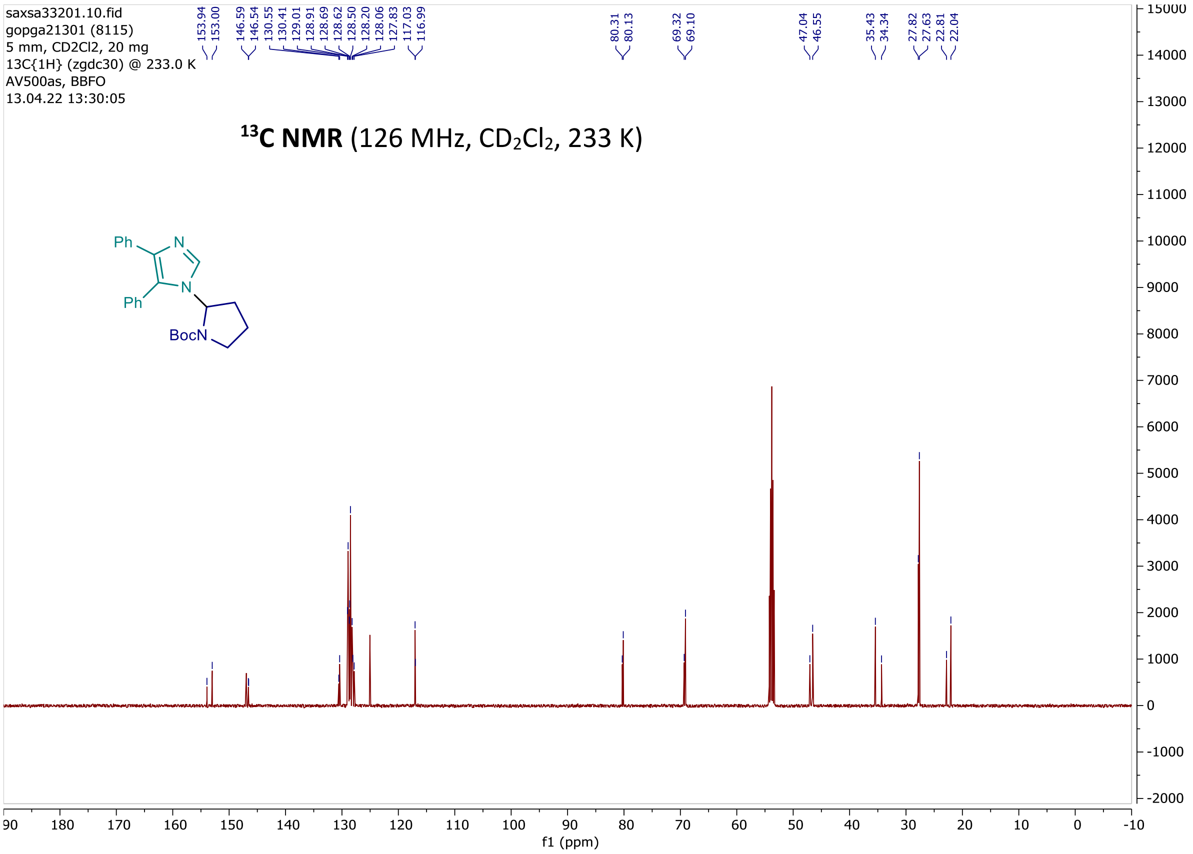

tert-Butyl 2-(1_H_-benzo[_d_][1,2,3]triazol-1-yl)pyrrolidine-1-carboxylate (39) (5:1 N1/N2 rr)

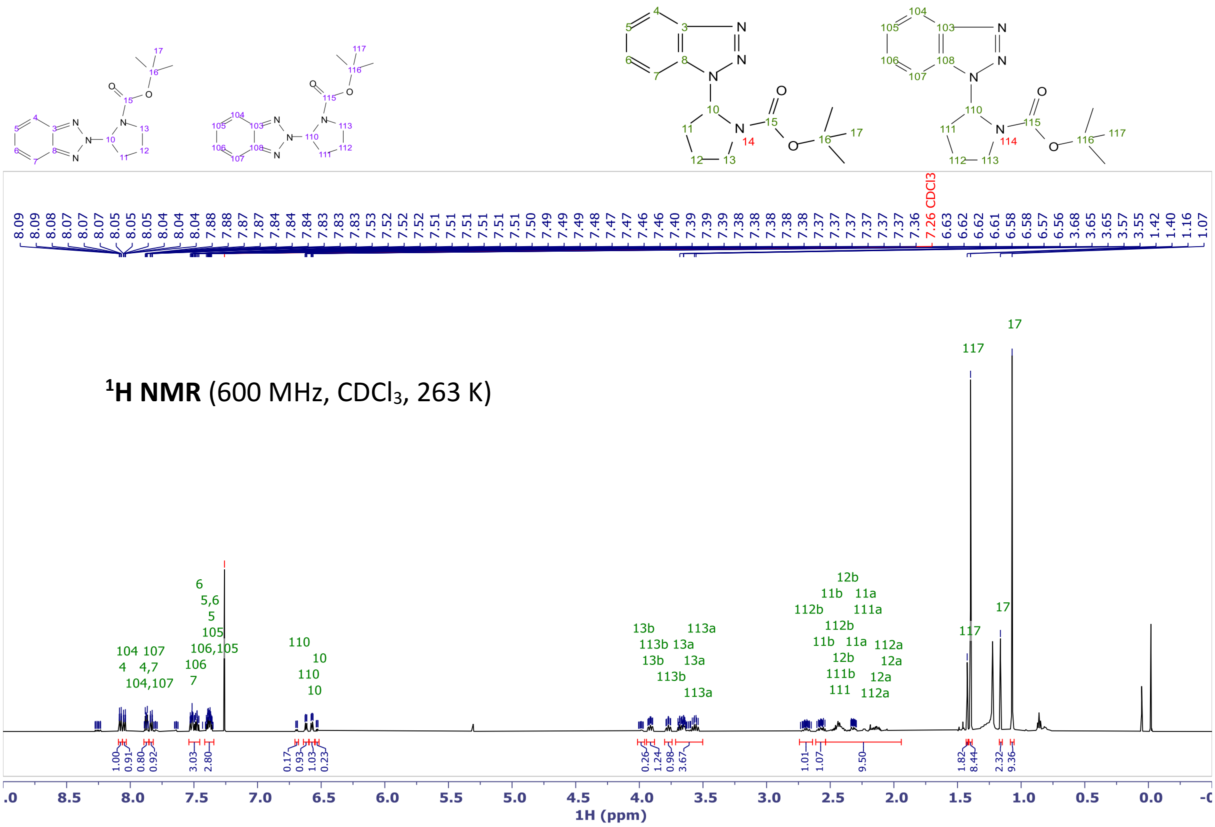

^{1}H NMR (600 MHz, $CDCl_3$, 263 K)

gopga14801.30.1.1r — gopga14801 (7830) — CDCl3; 263.0 K; 10 mg; 5 mm — 1H (zg30) — AV600neo (cryoBBO) — 02.03.22 09:27:31

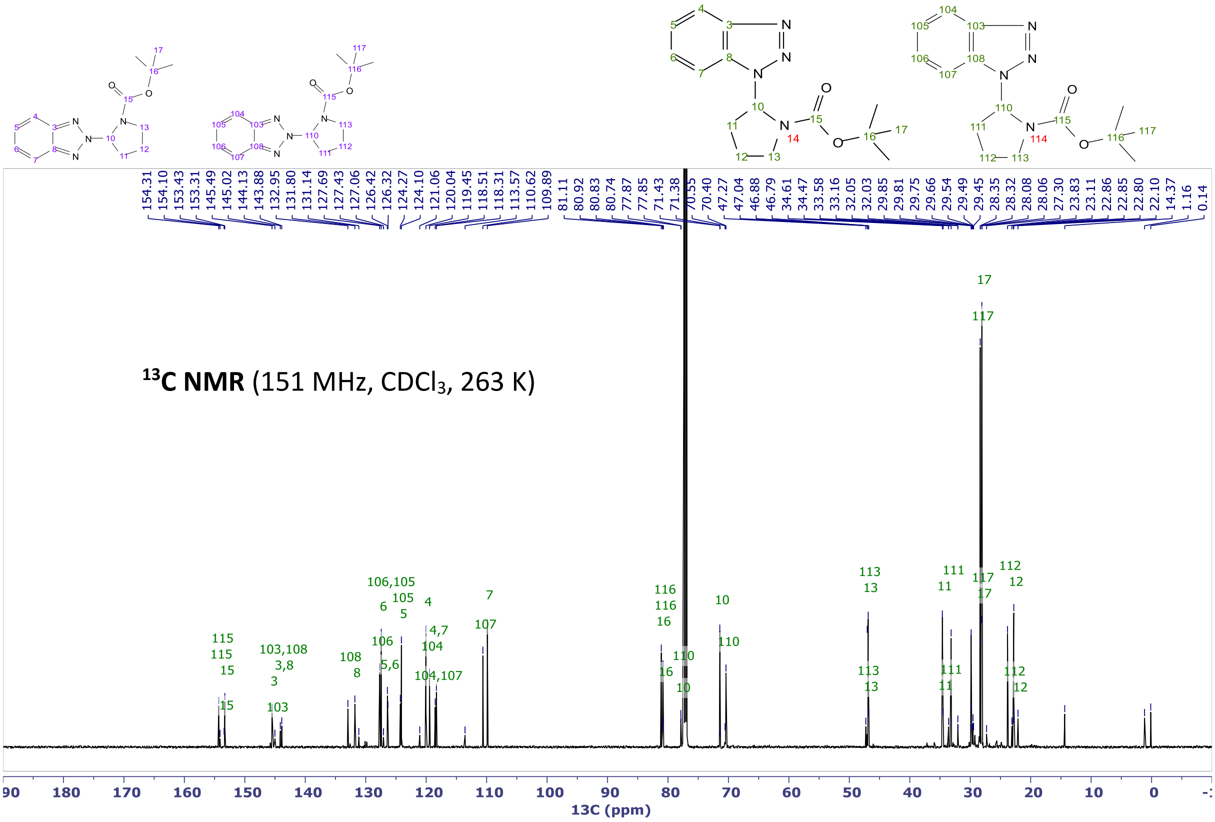

^{13}C NMR (151 MHz, $CDCl_3$, 263 K)

gopga14801.31.1.1r — gopga14801 (7830) — CDCl3; 263.0 K; 10 mg; 5 mm — 13C{1H} (zgpg30) — AV600neo (cryoBBO) — 02.03.22 10:39:55

***tert*-Butyl 2-(4-bromo-1*H*-pyrazol-1-yl)pyrrolidine-1-carboxylate (40)**

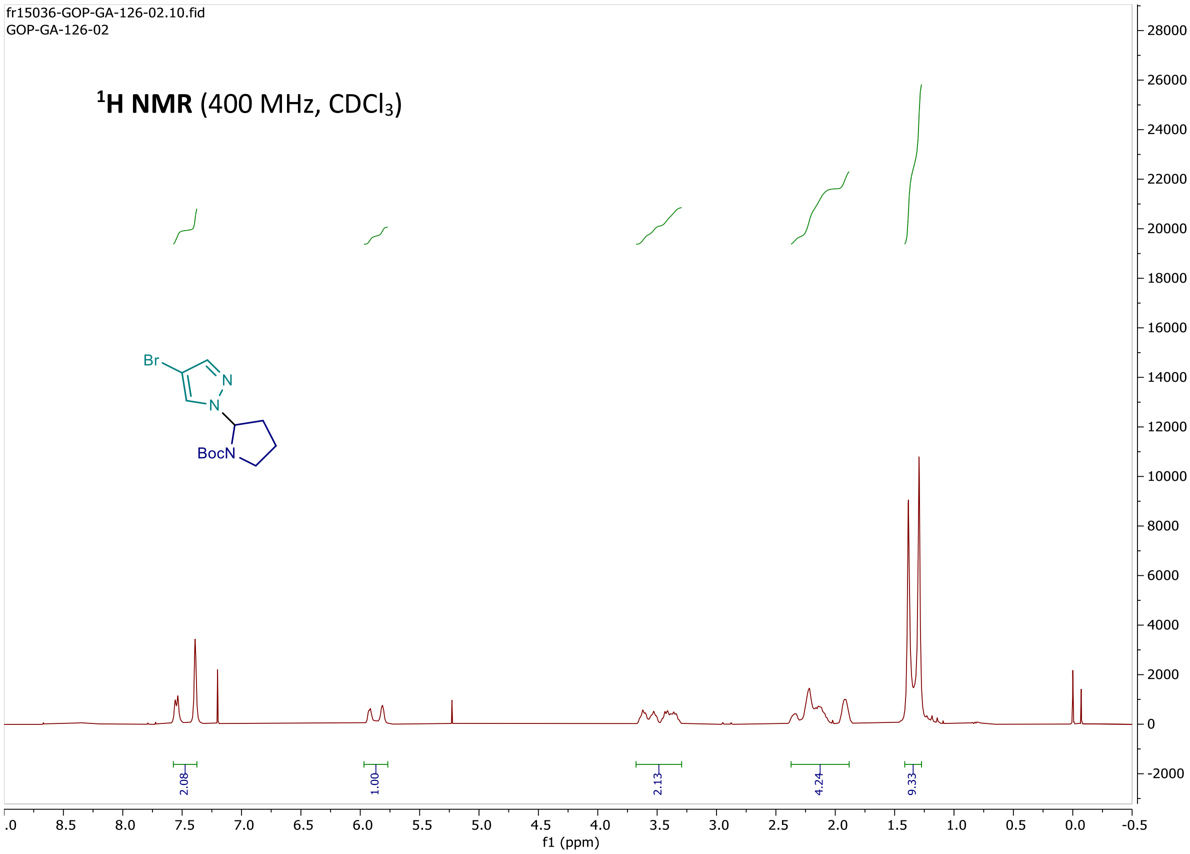

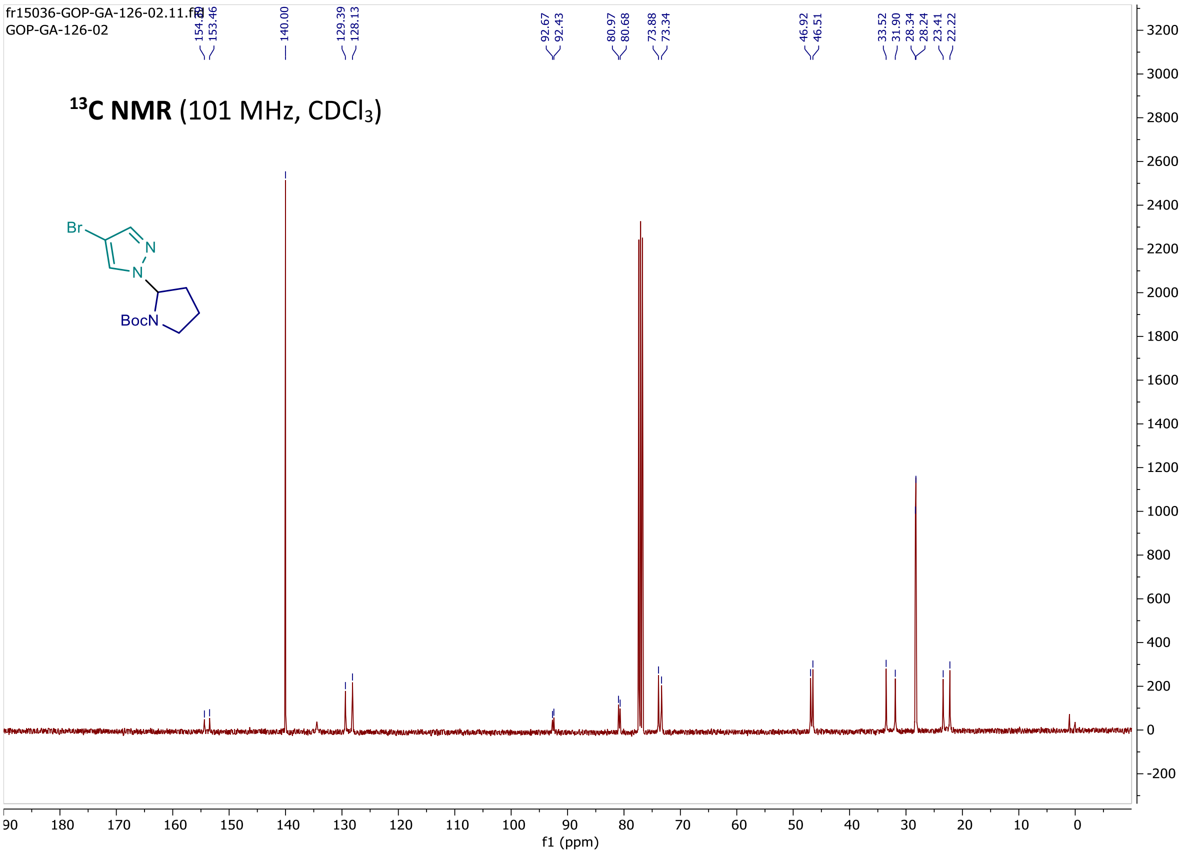

tert-Butyl 2-(4-chloro-1*H*-pyrazol-1-yl)pyrrolidine-1-carboxylate (41)

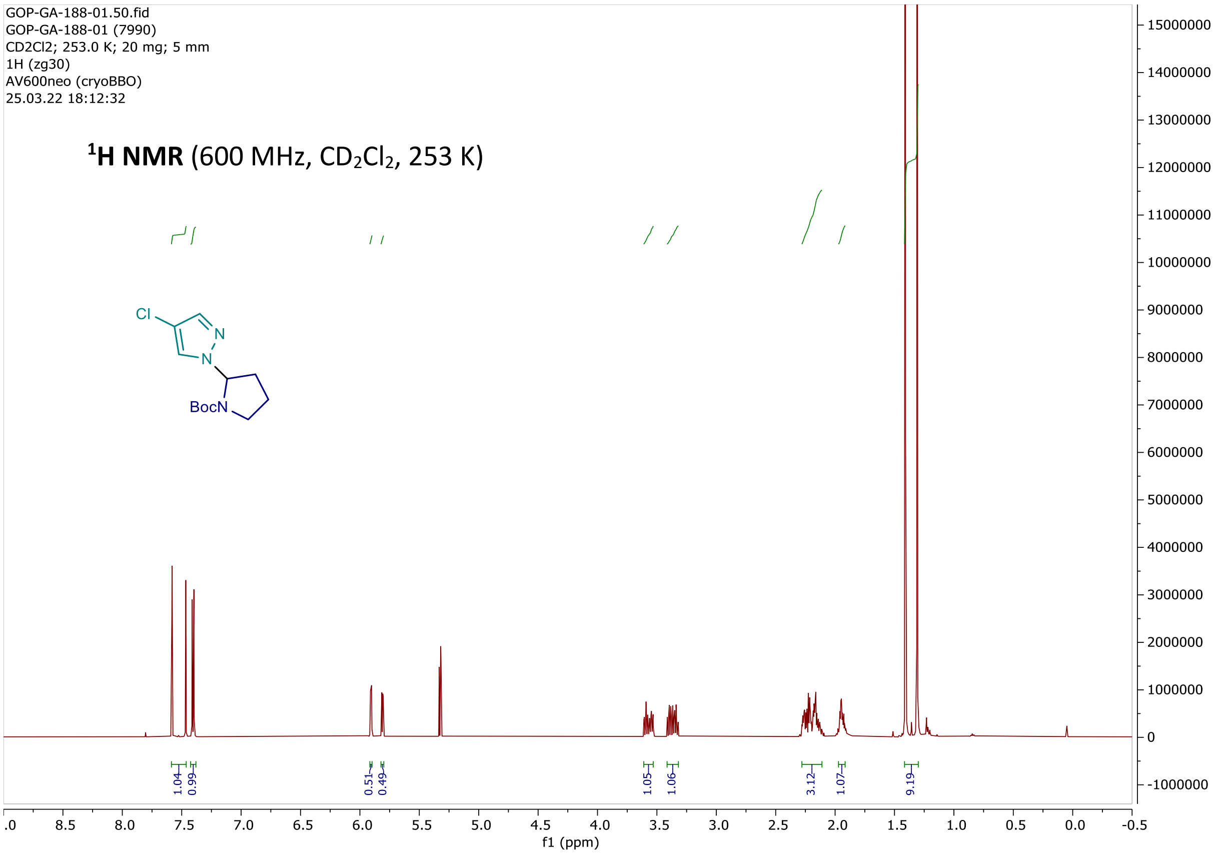

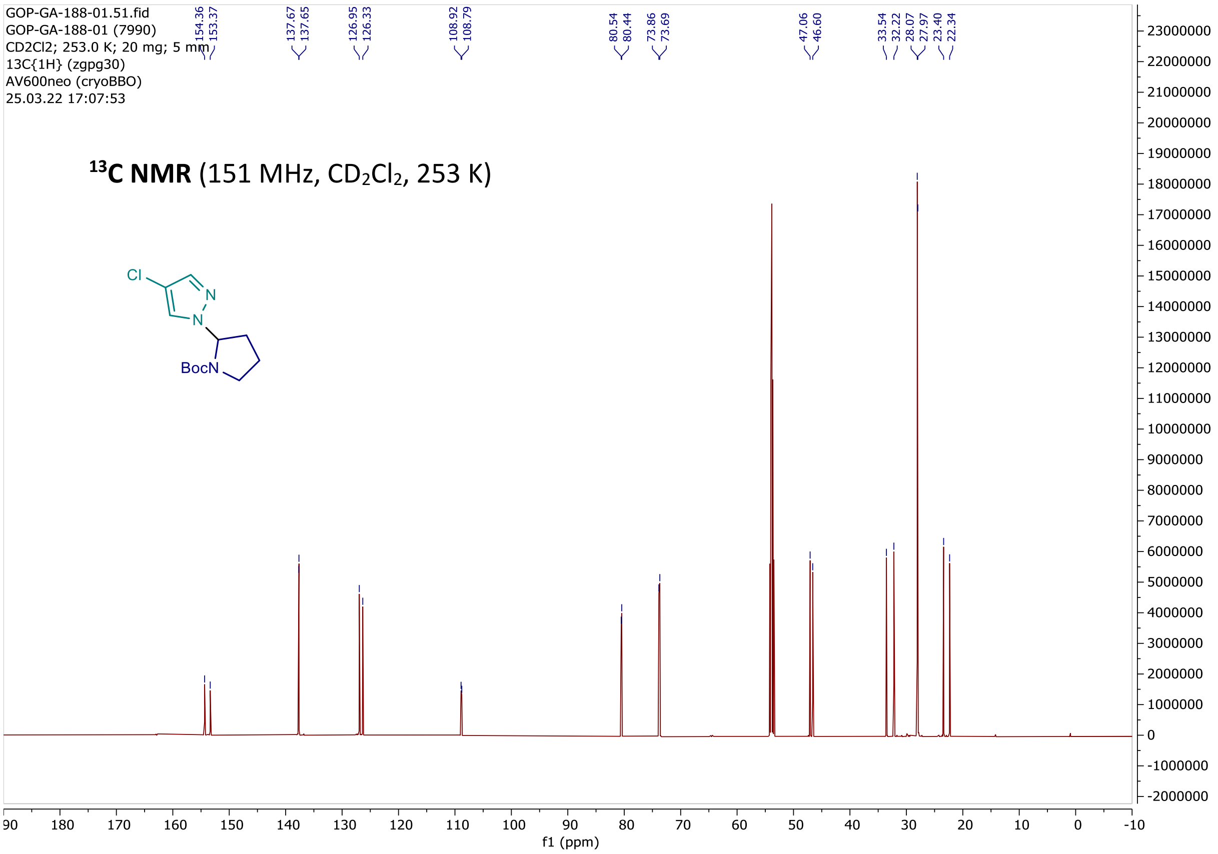

***tert*-Butyl 2-(4-bromo-3,5-dimethyl-1*H*-pyrazol-1-yl)pyrrolidine-1-carboxylate (42)**

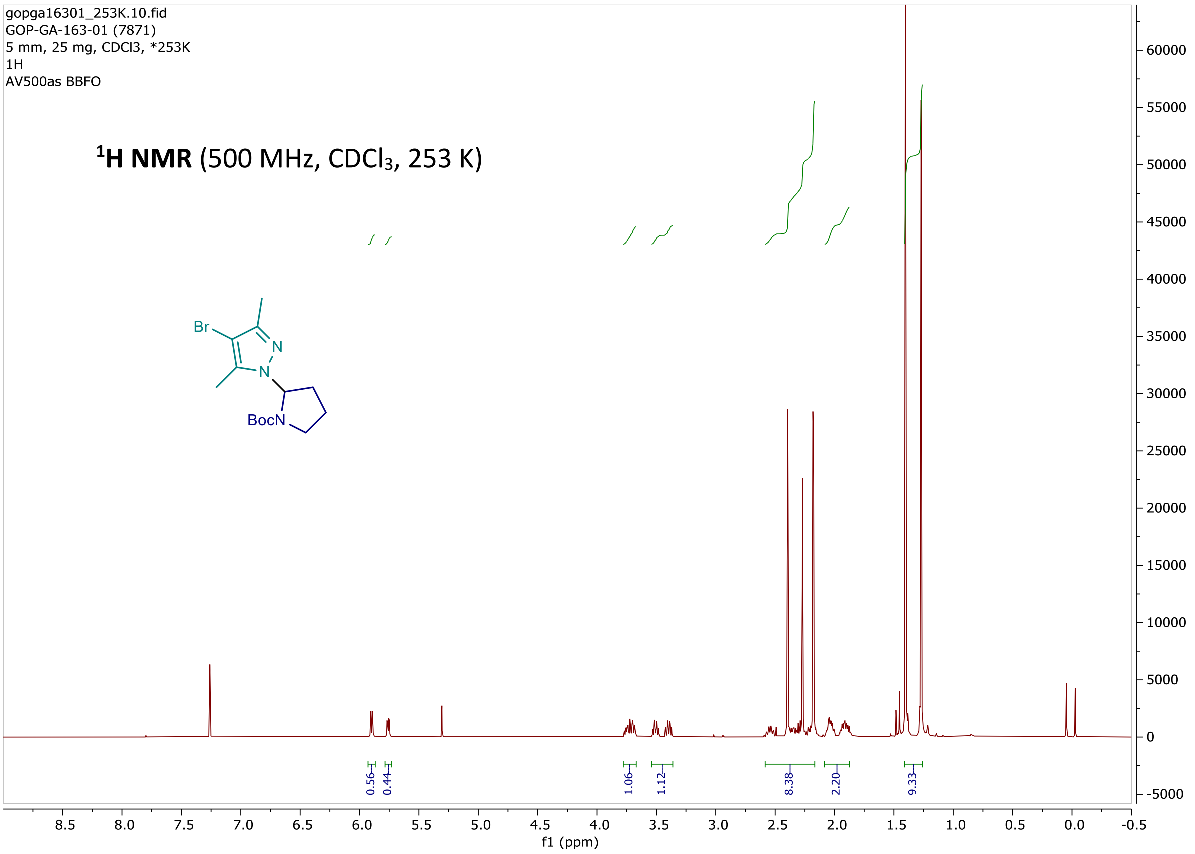

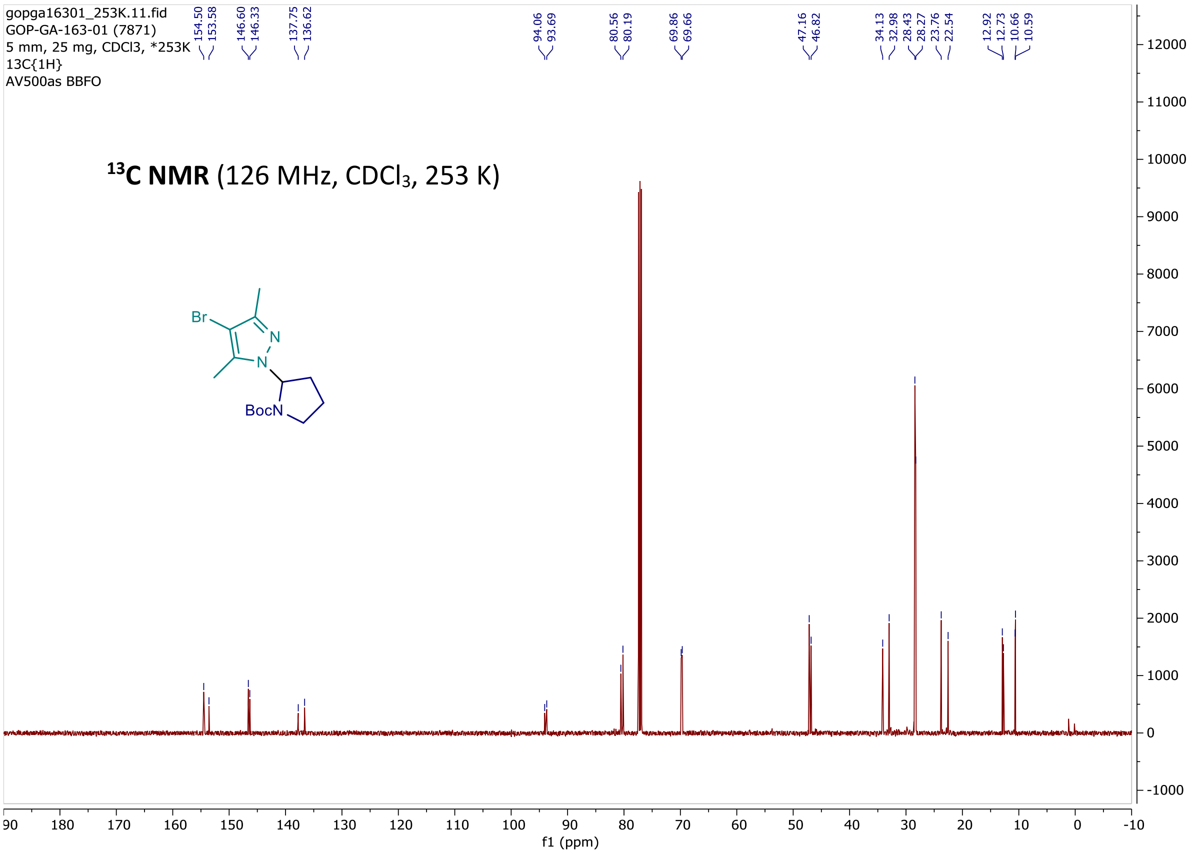

tert-Butyl 2-(3,5-bis(trifluoromethyl)-1-pyrazol-1-yl)pyrrolidine-1-carboxylate (43)

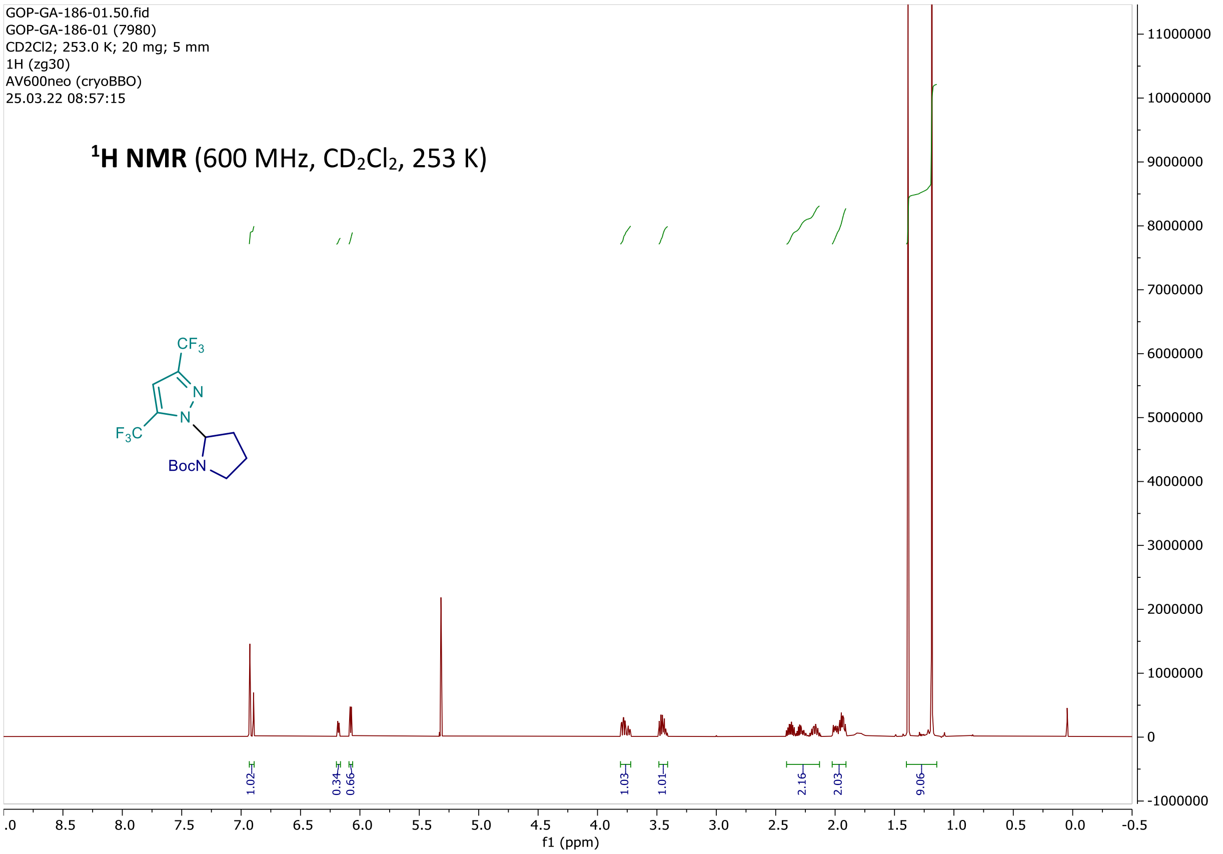

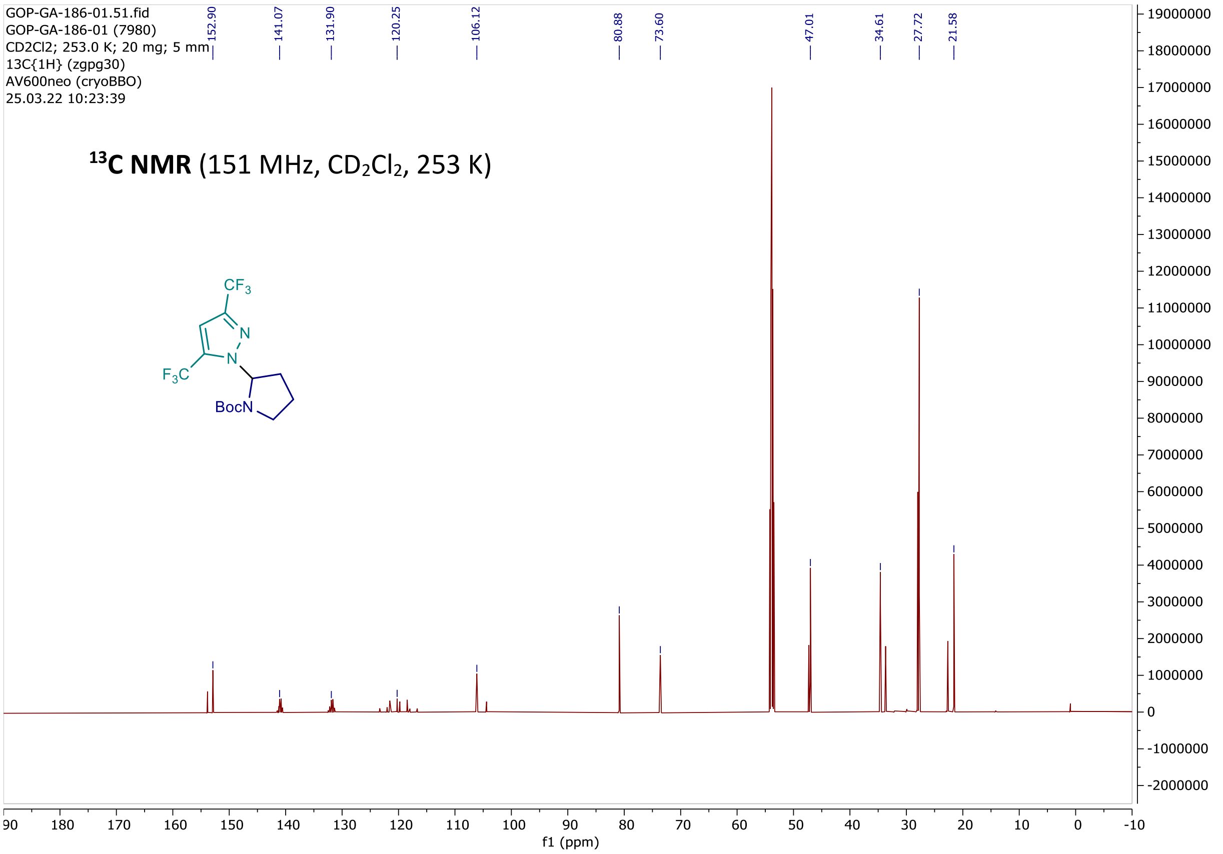

^{13}C aromatic detail of 43

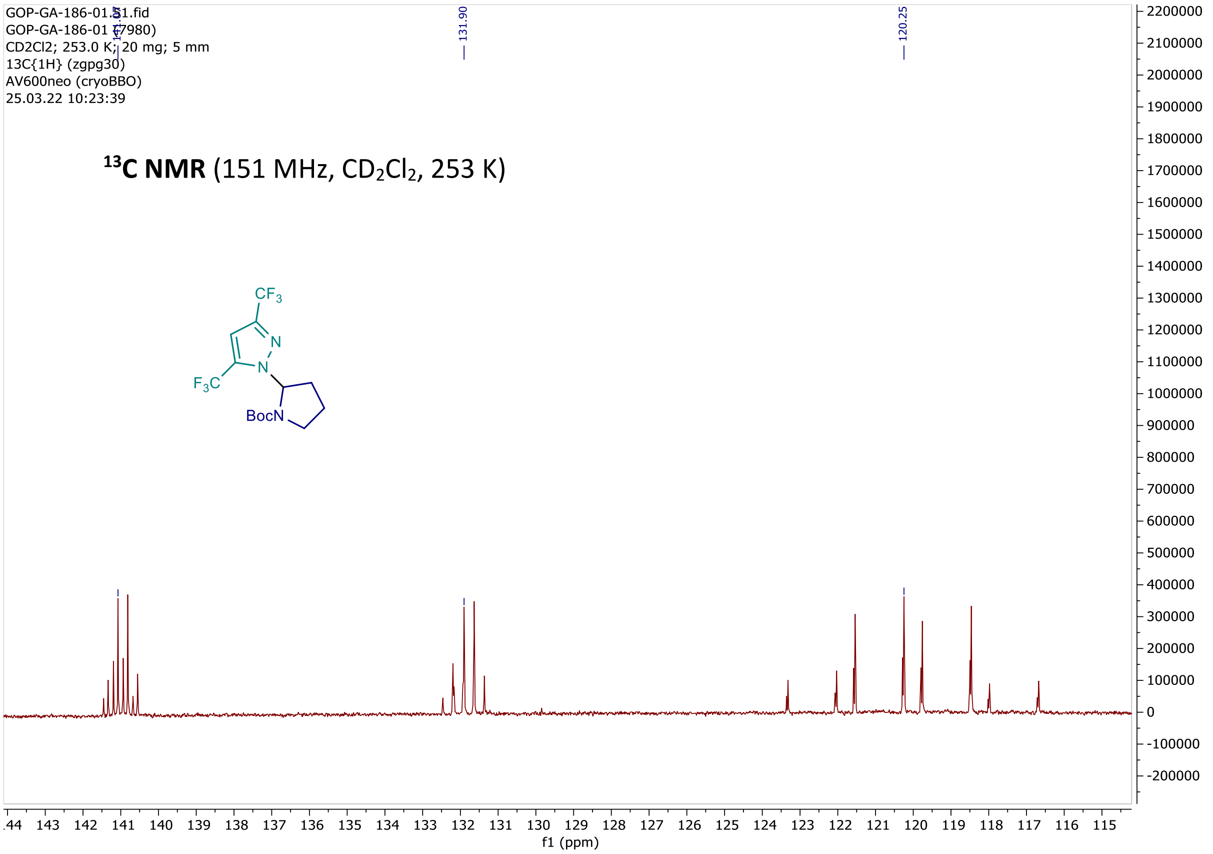

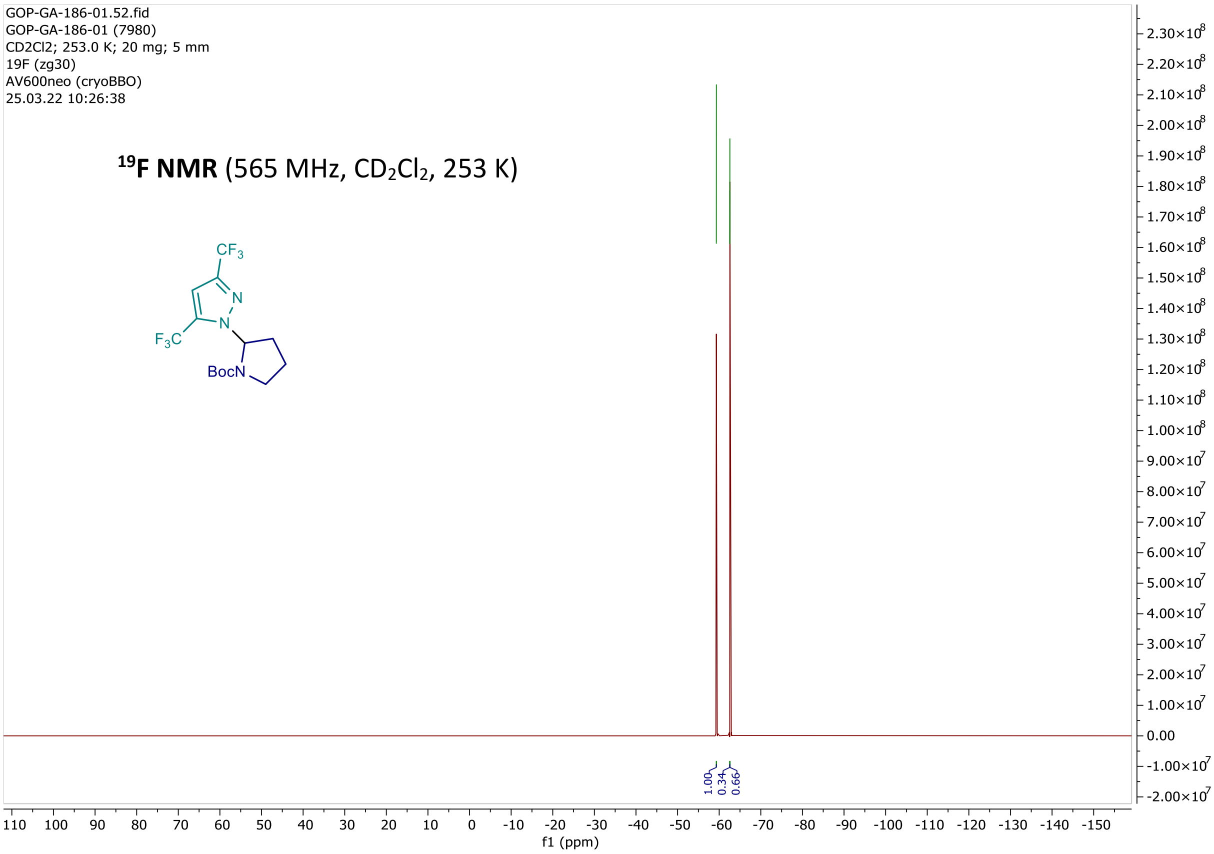

tert-Butyl 2-(2-phenyl-1H-imidazol-1-yl)pyrrolidine-1-carboxylate (44)

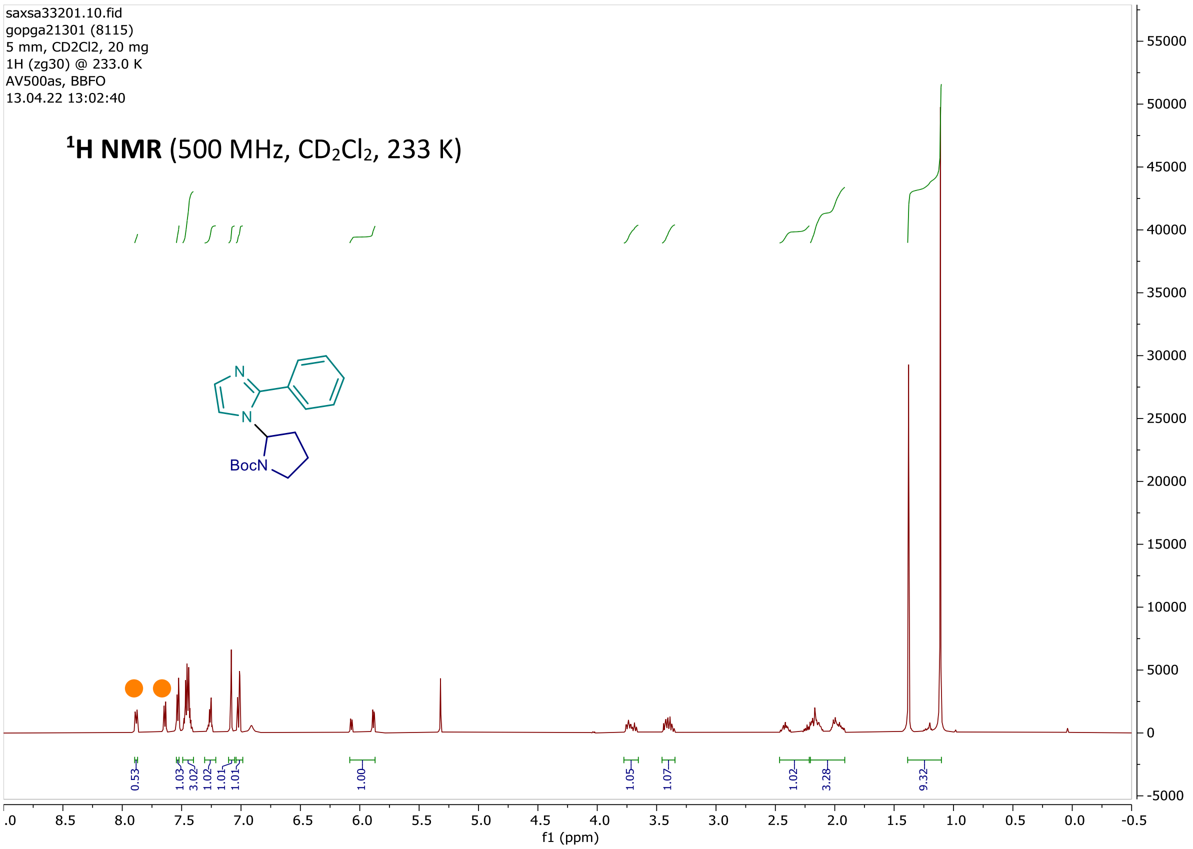

20% of starting nucleophile

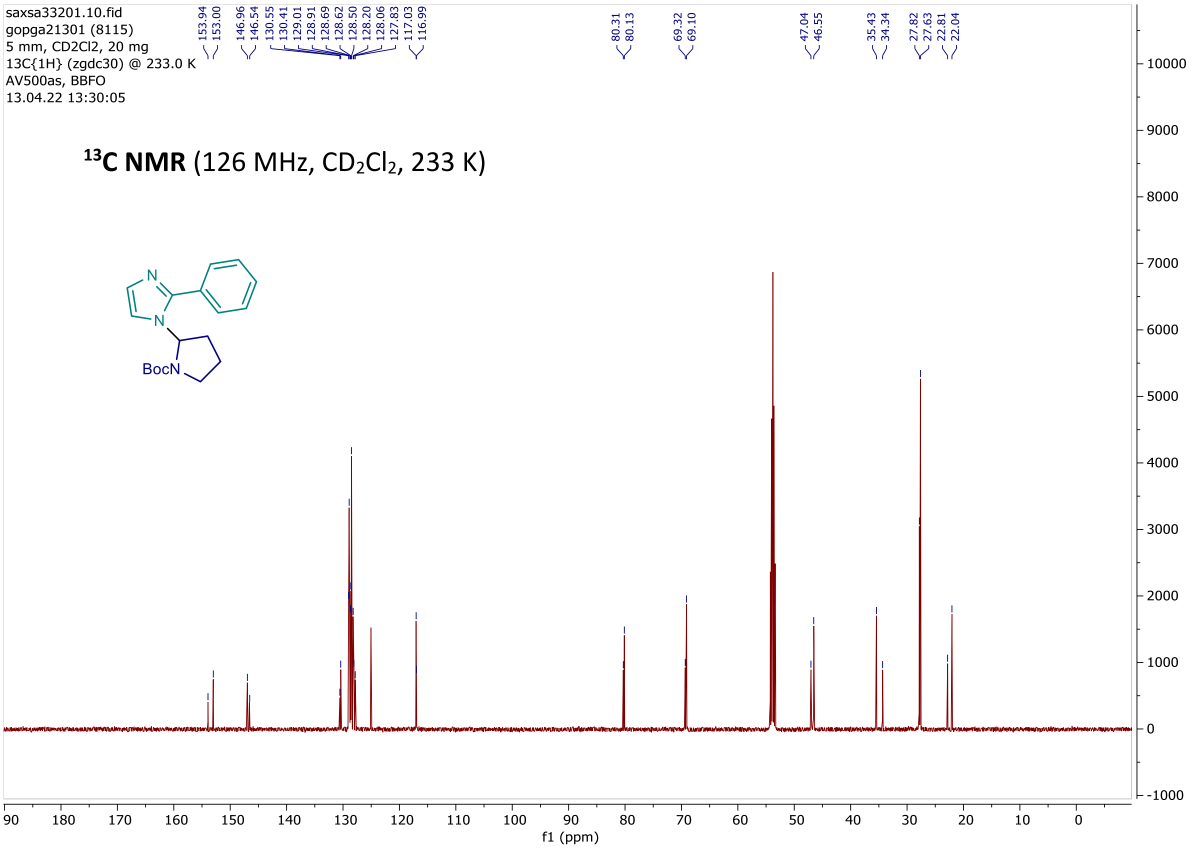

tert-Butyl 2-(2-methyl-1*H*-benzo[*d*]imidazol-1-yl)pyrrolidine-1-carboxylate (45)

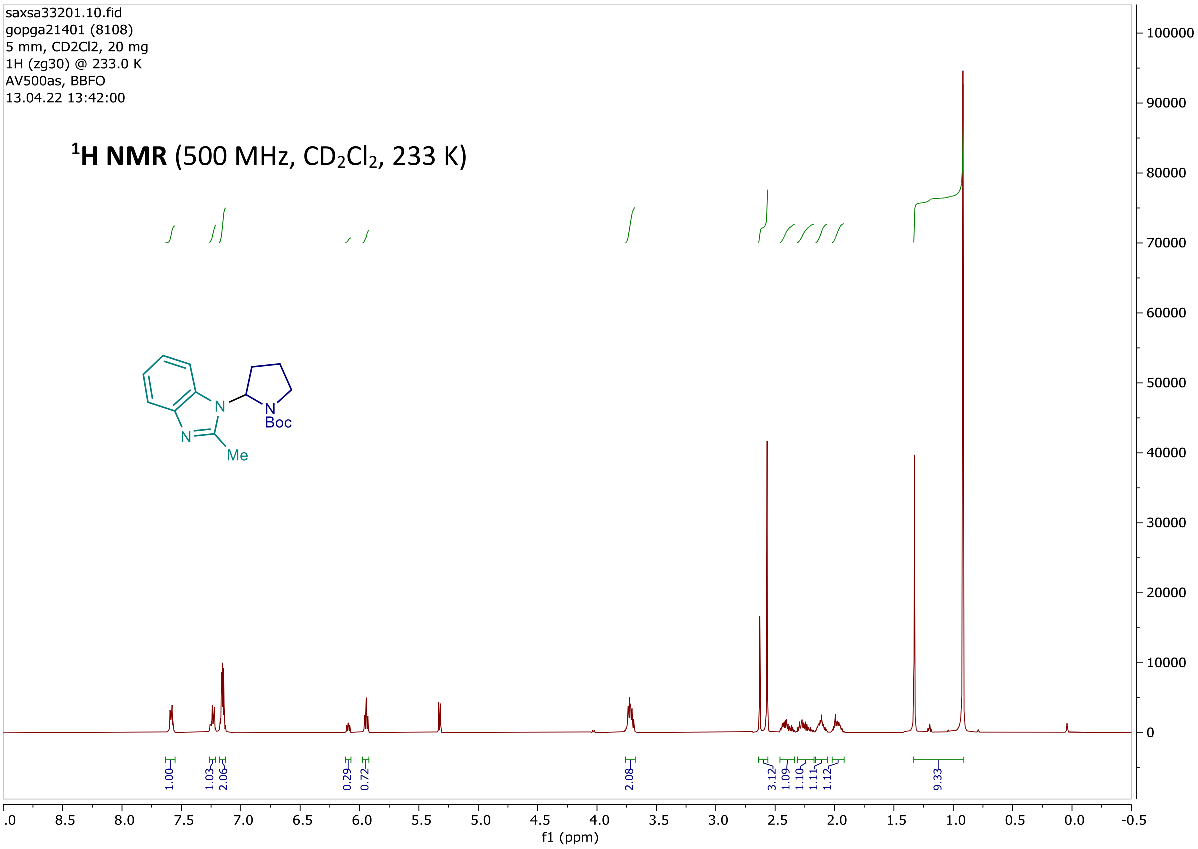

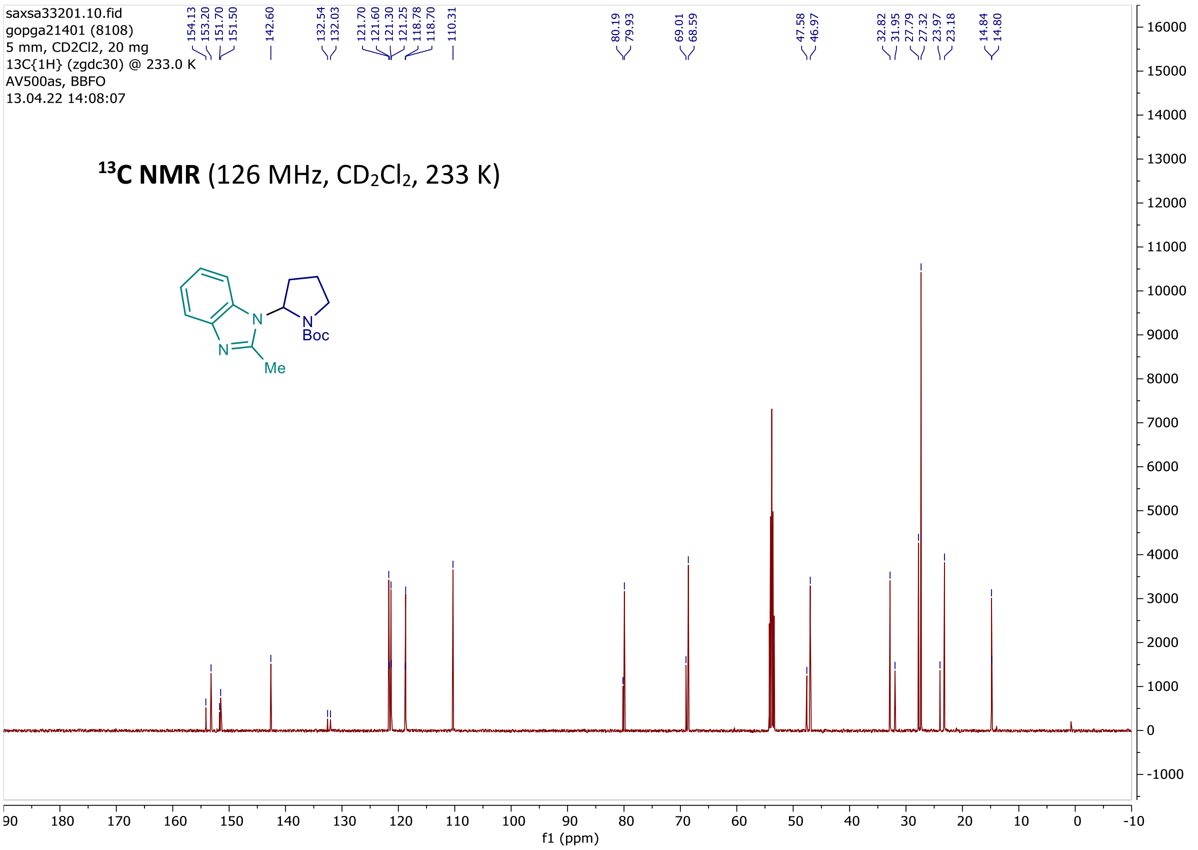

Dimethyl 1-(1-(*tert*-butoxycarbonyl)pyrrolidin-2-yl)-1*H*-imidazole-4,5-dicarboxylate (46)

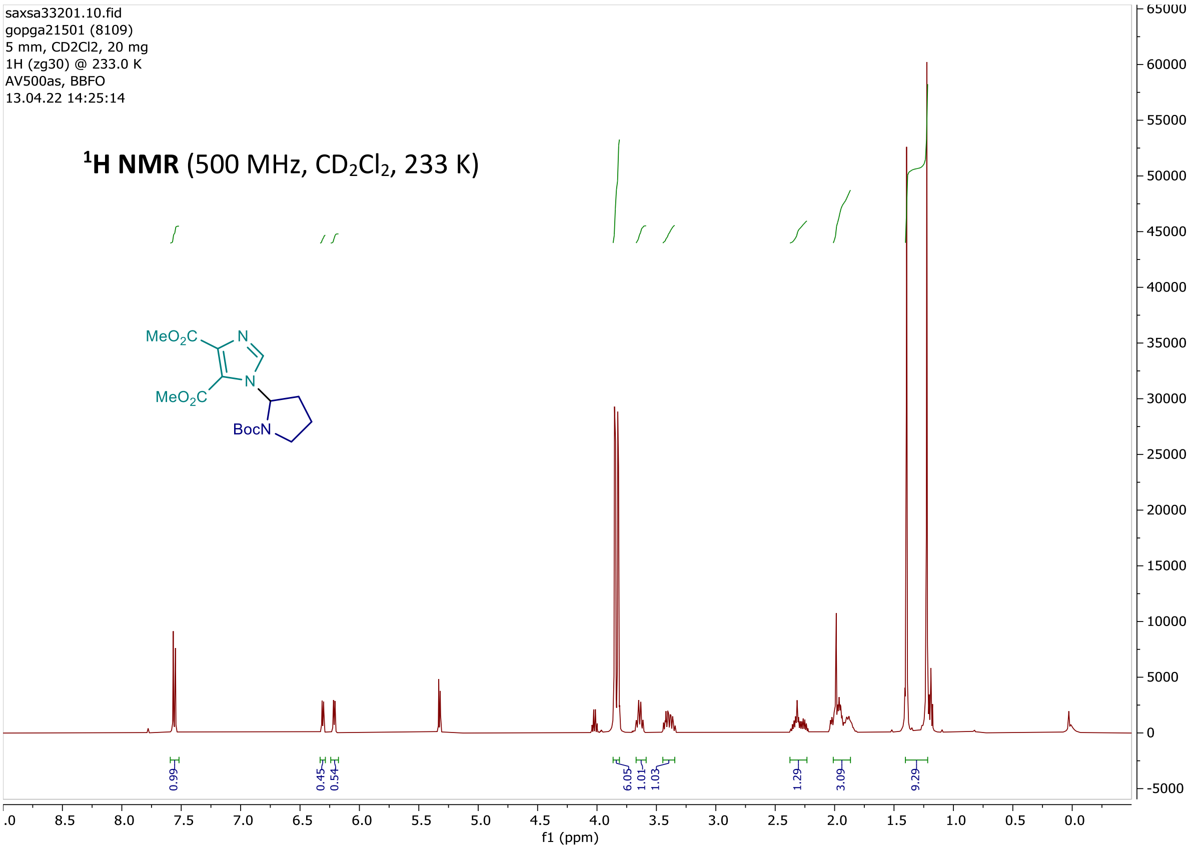

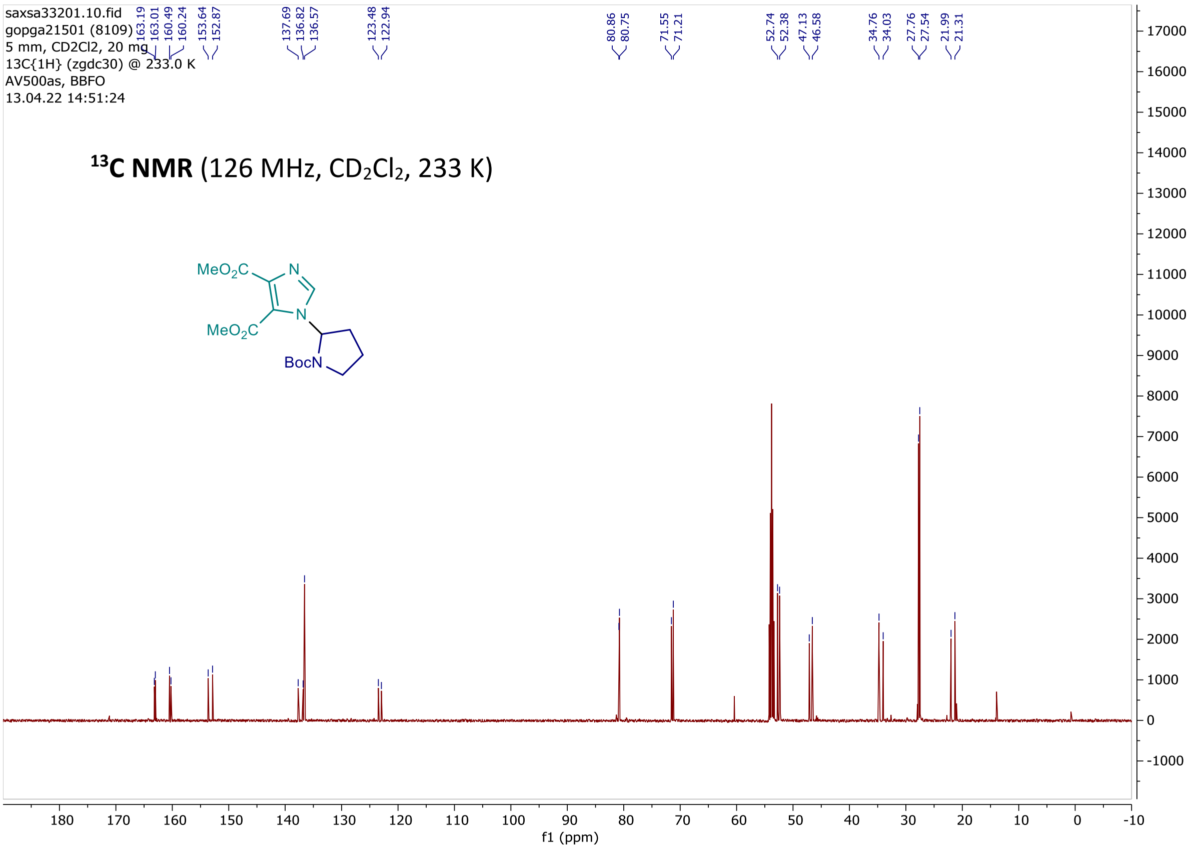

tert-Butyl 2-(1,3-dimethyl-2,6-dioxo-1,2,3,6-tetrahydro-7_H_-purin-7-yl)pyrrolidine-1-carboxylate (47)

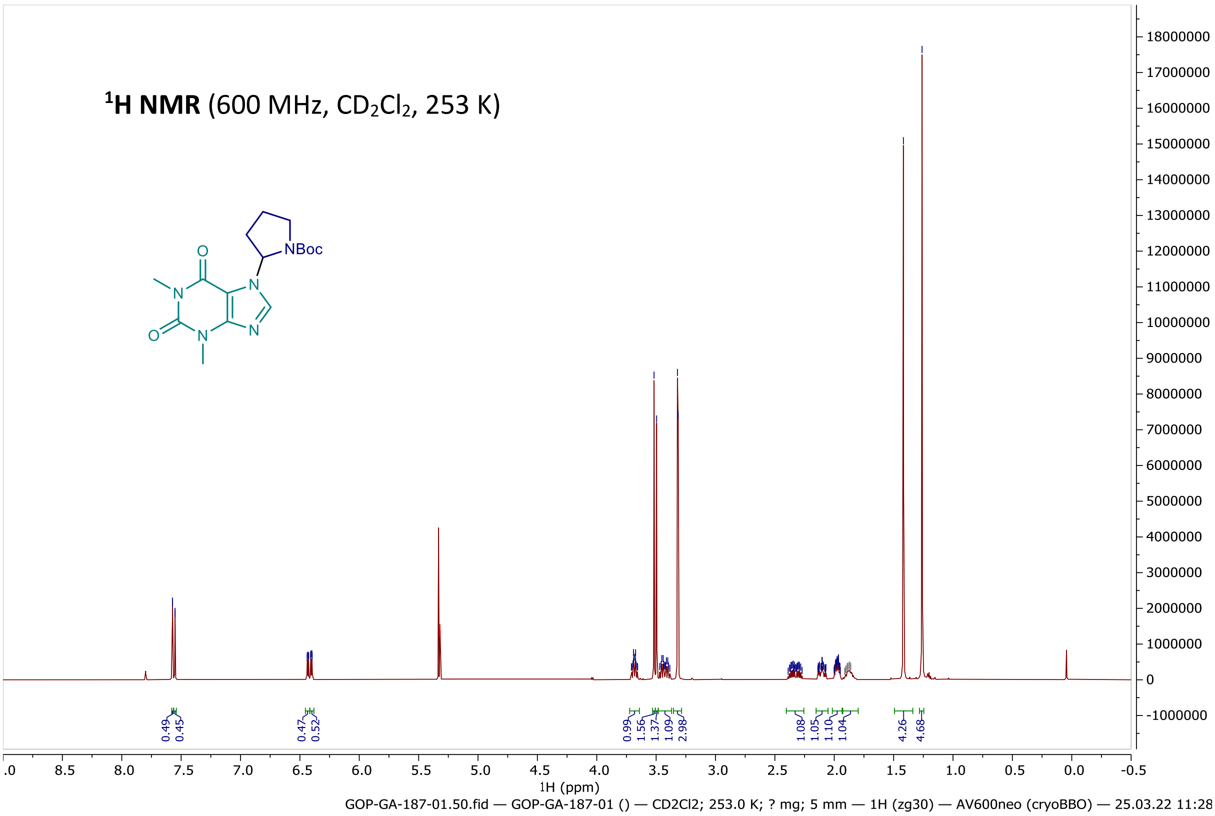

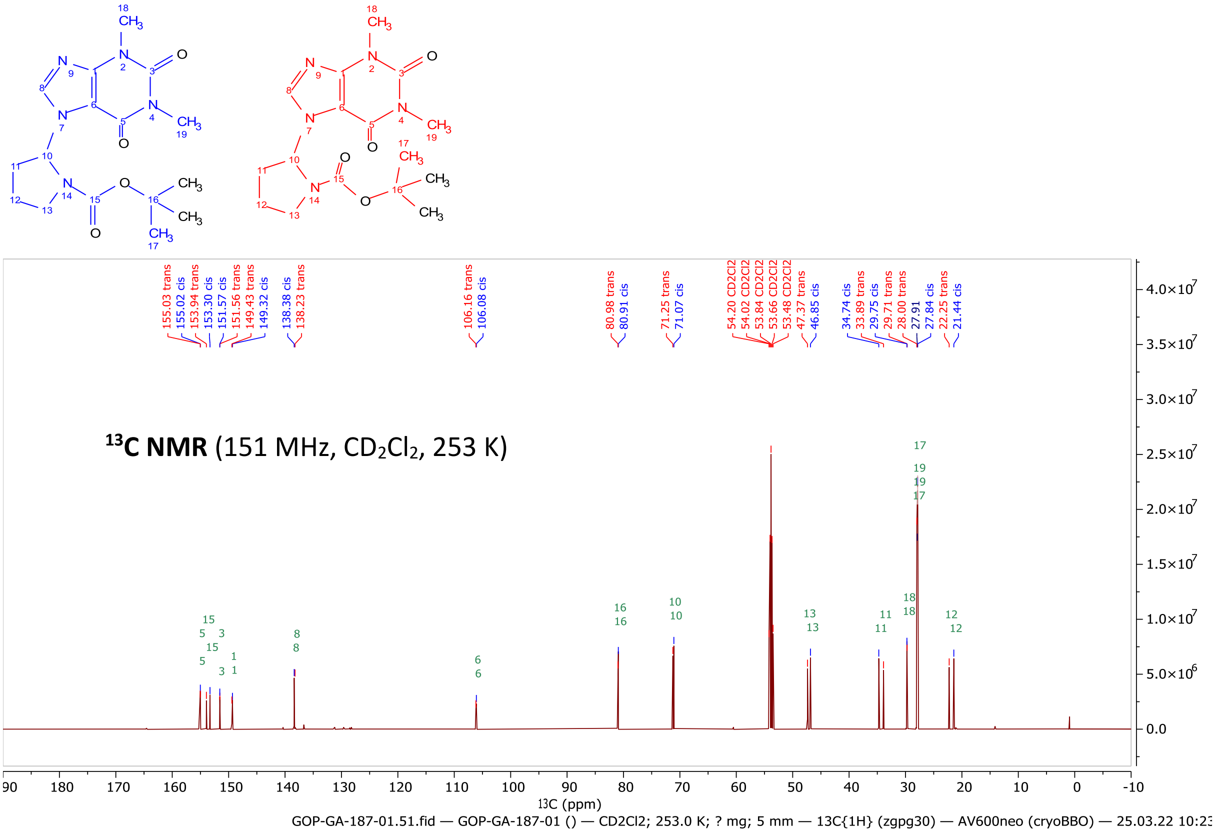

tert-Butyl 2-(1,3-dimethyl-2,6-dioxo-1,2,3,6-tetrahydro-7*H*-purin-7-yl)pyrrolidine-1-carboxylate (47)

^{1}H NOESY

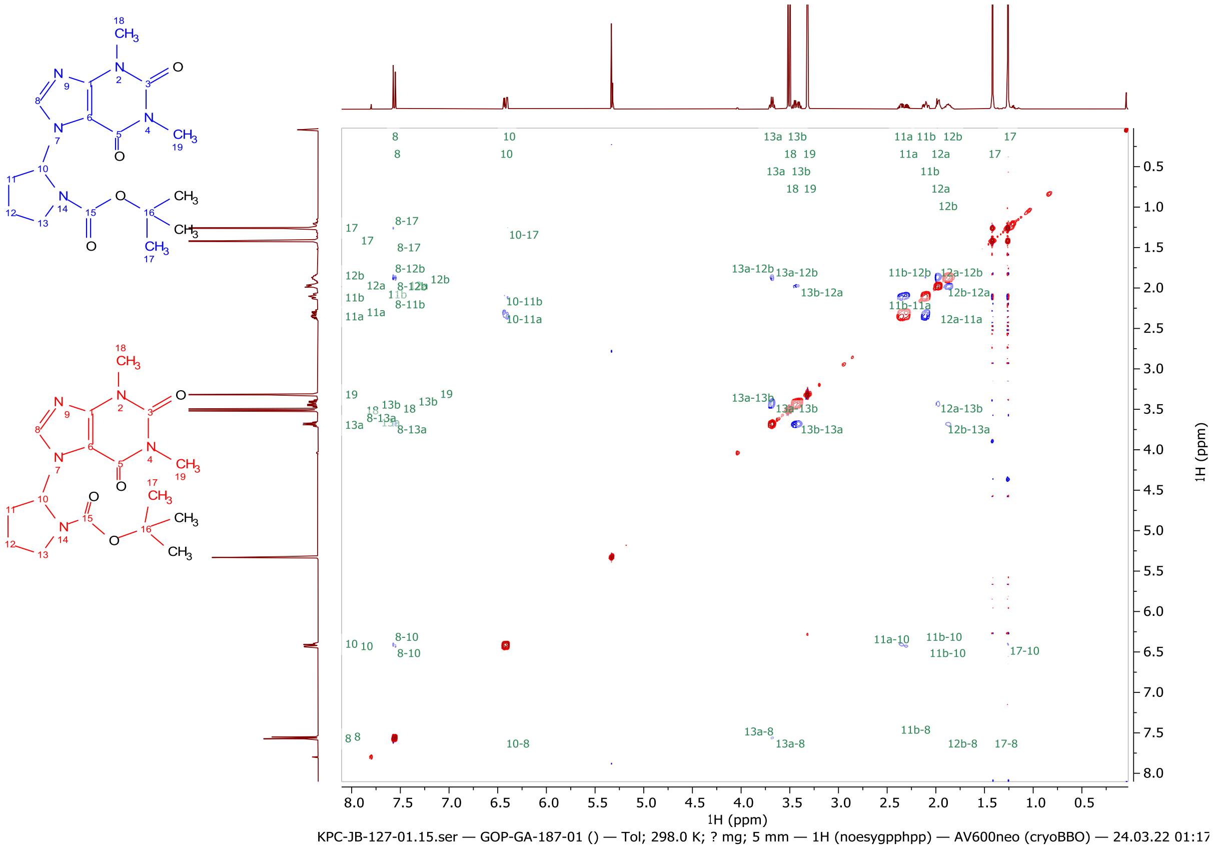

KPC-JB-127-01.15.ser — GOP-GA-187-01 () — Tol; 298.0 K; ? mg; 5 mm — 1H (noesygpphpp) — AV600neo (cryoBBO) — 24.03.22 01:17

^{1}H-^{15}N HMBC

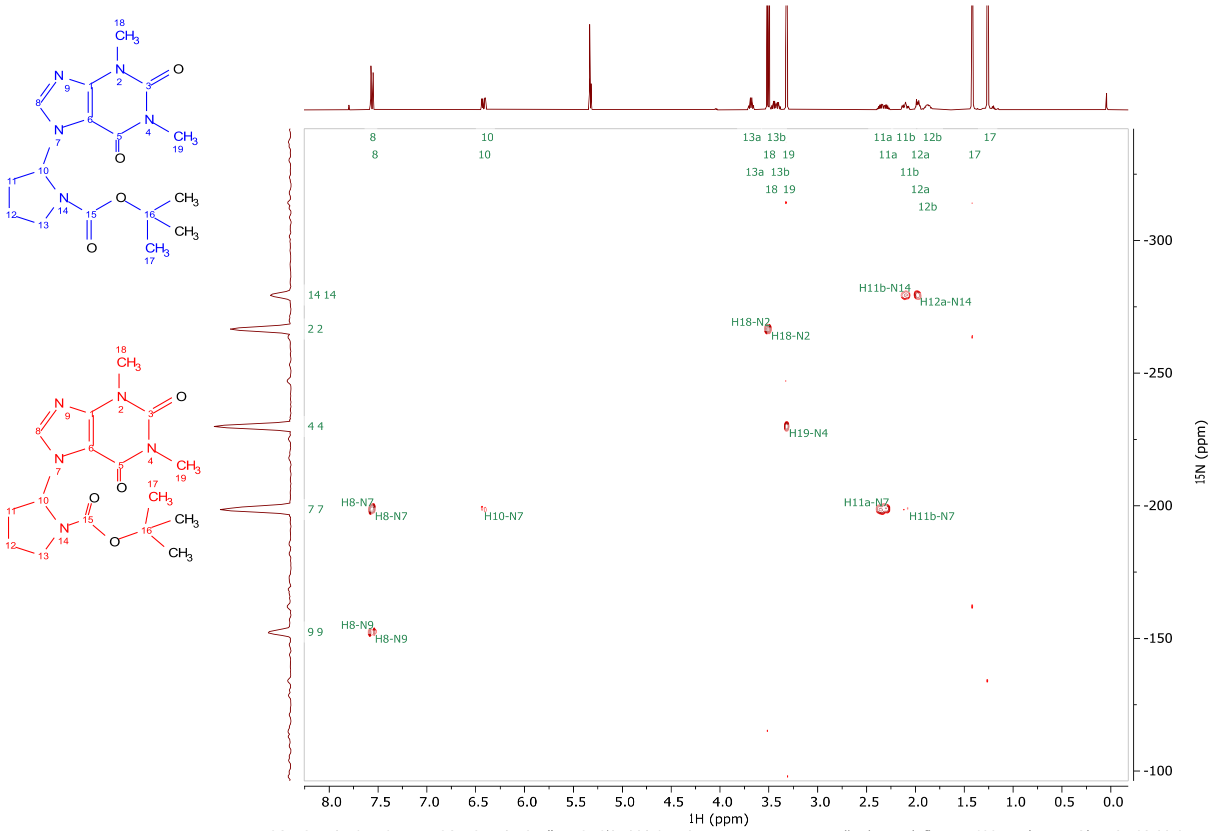

GOP-GA-187-01.56.ser — GOP-GA-187-01 () — CDCl3; 298.0 K; ? mg; 5 mm — 1H-15N (hmbcgpndqf) — AV600neo (cryoBBO) — 24.03.22 07:17

tert-Butyl 2-(2-(thiazol-4-yl)-1*H*-benzo[*d*]imidazol-1-yl)pyrrolidine-1-carboxylate (48)

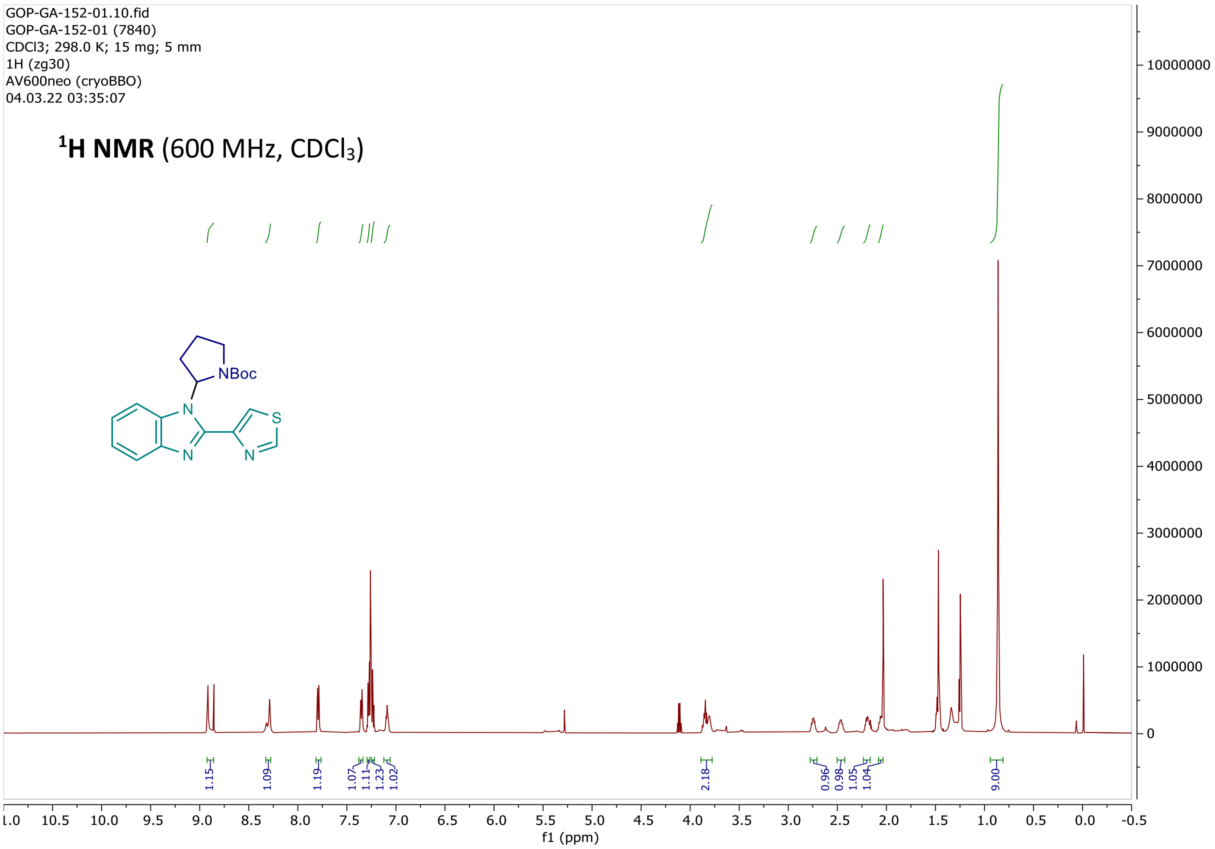

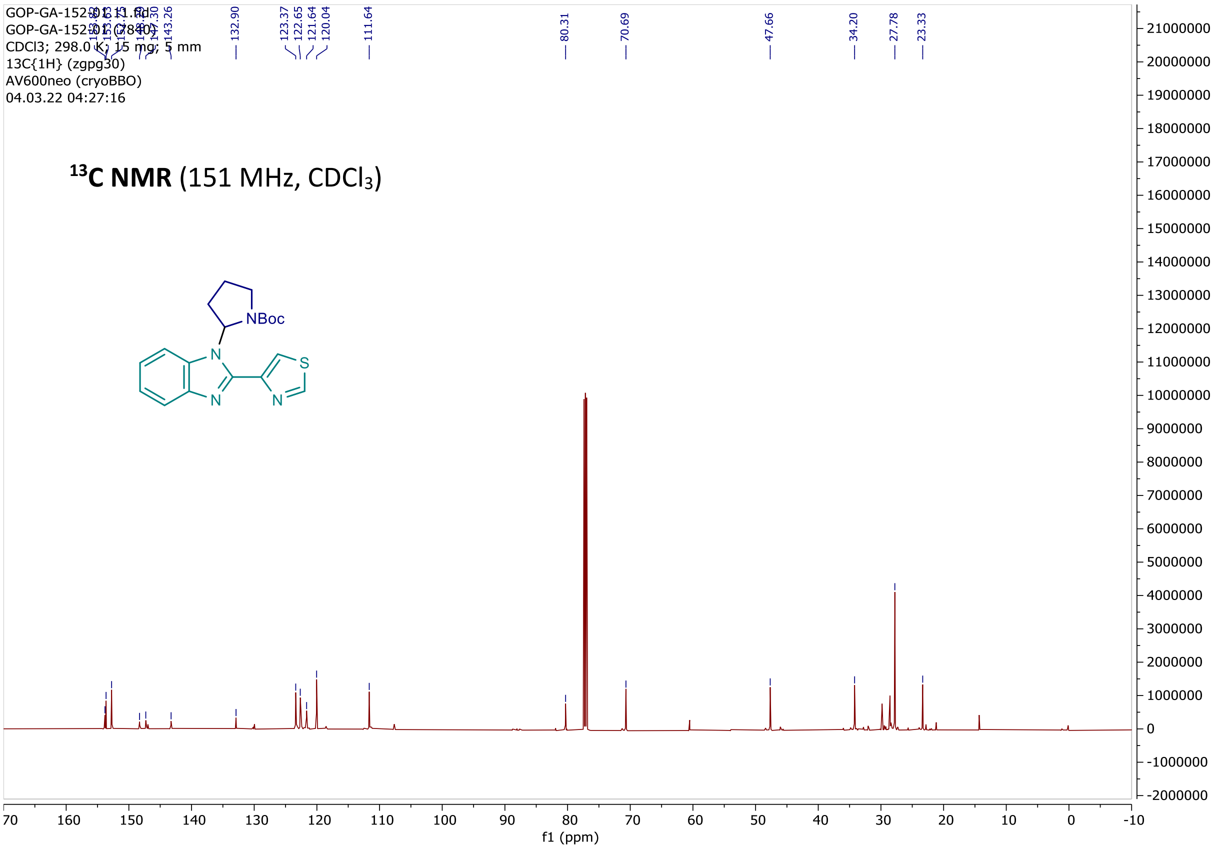

tert-Butyl 2-(2-oxo-6-(trifluoromethoxy)benzo[*d*]thiazol-3(2*H*)-yl)pyrrolidine-1-carboxylate (48)

Full assignment of ^{1}H (both rotamers)

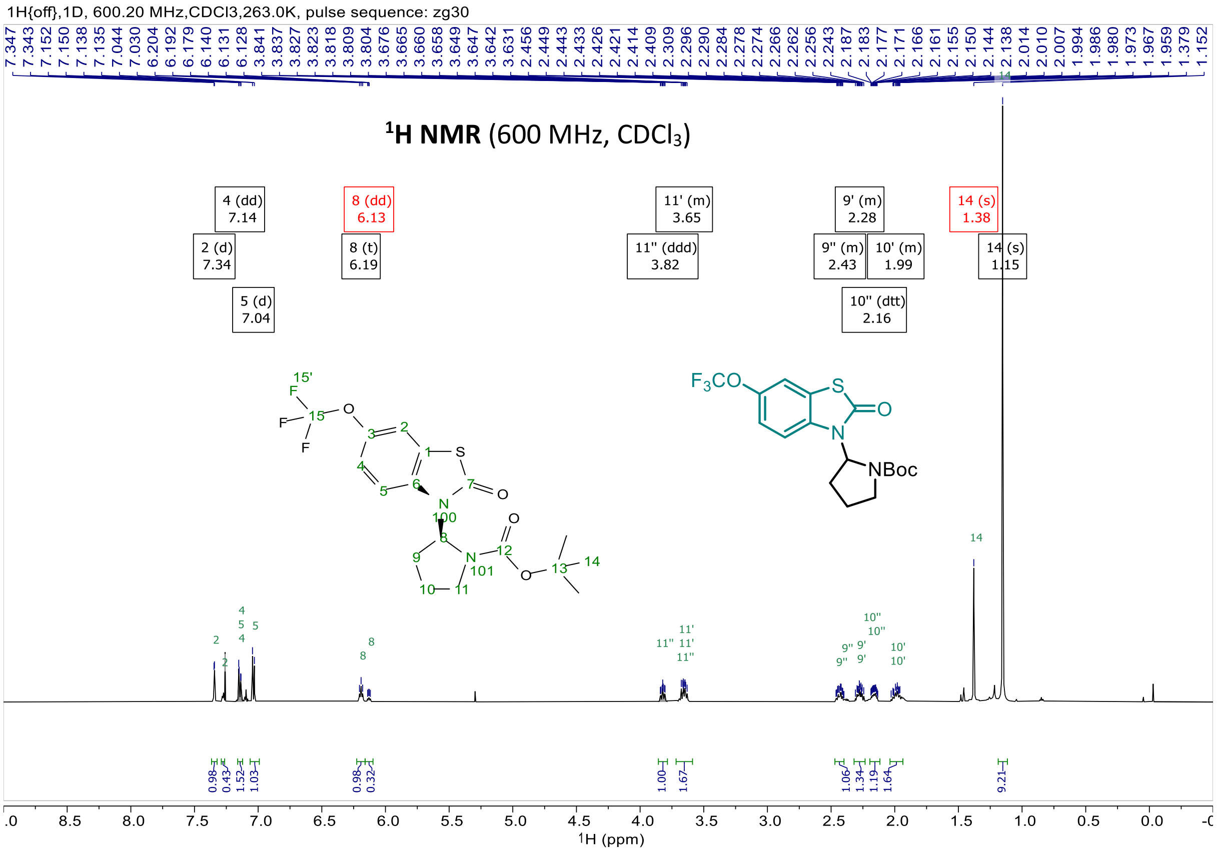

Full assignment of ^{13}C (both rotamers)

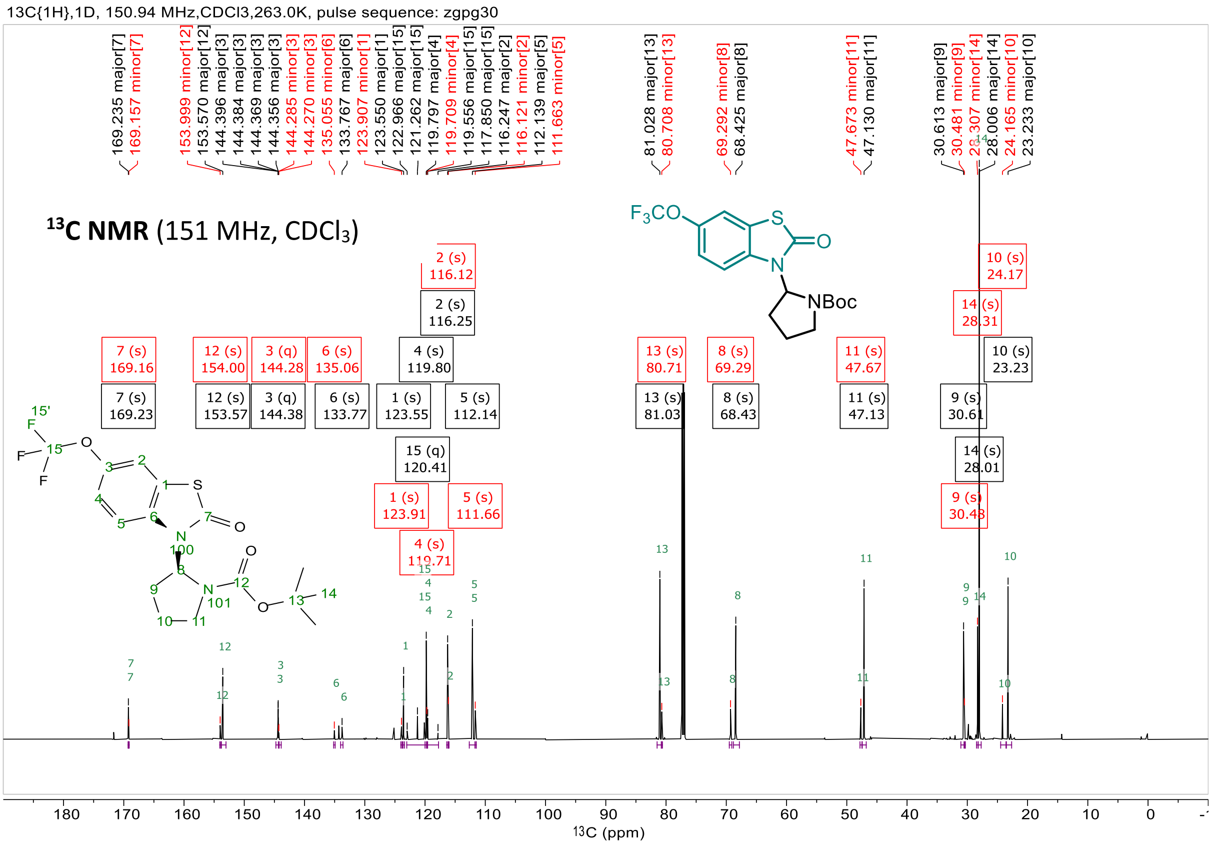

1H COSY

1H{off},COSY, 600.20 MHz,CDCl3,263.0K, pulse sequence: cosygpppqf

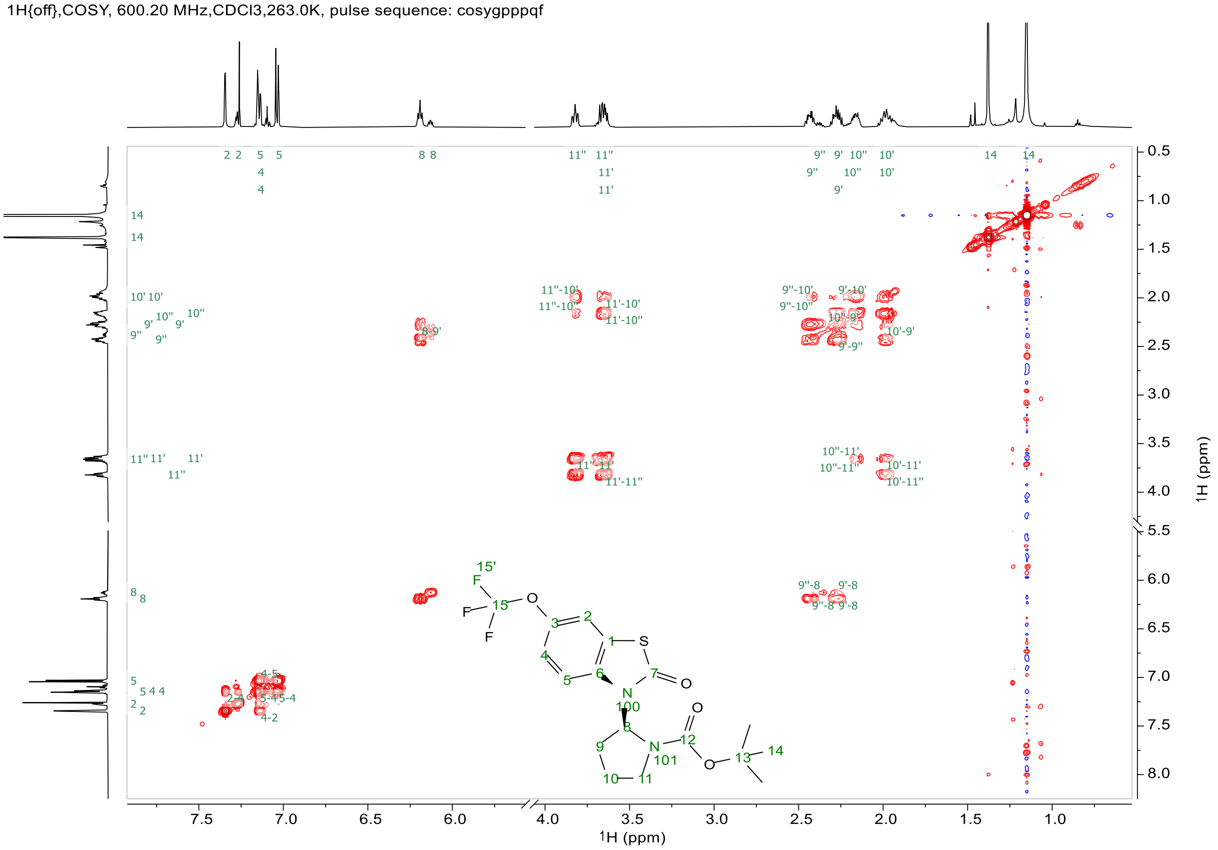

^{19}F NMR

19F{off},1D, 564.72 MHz,CDCl3,263.0K, pulse sequence: zg30

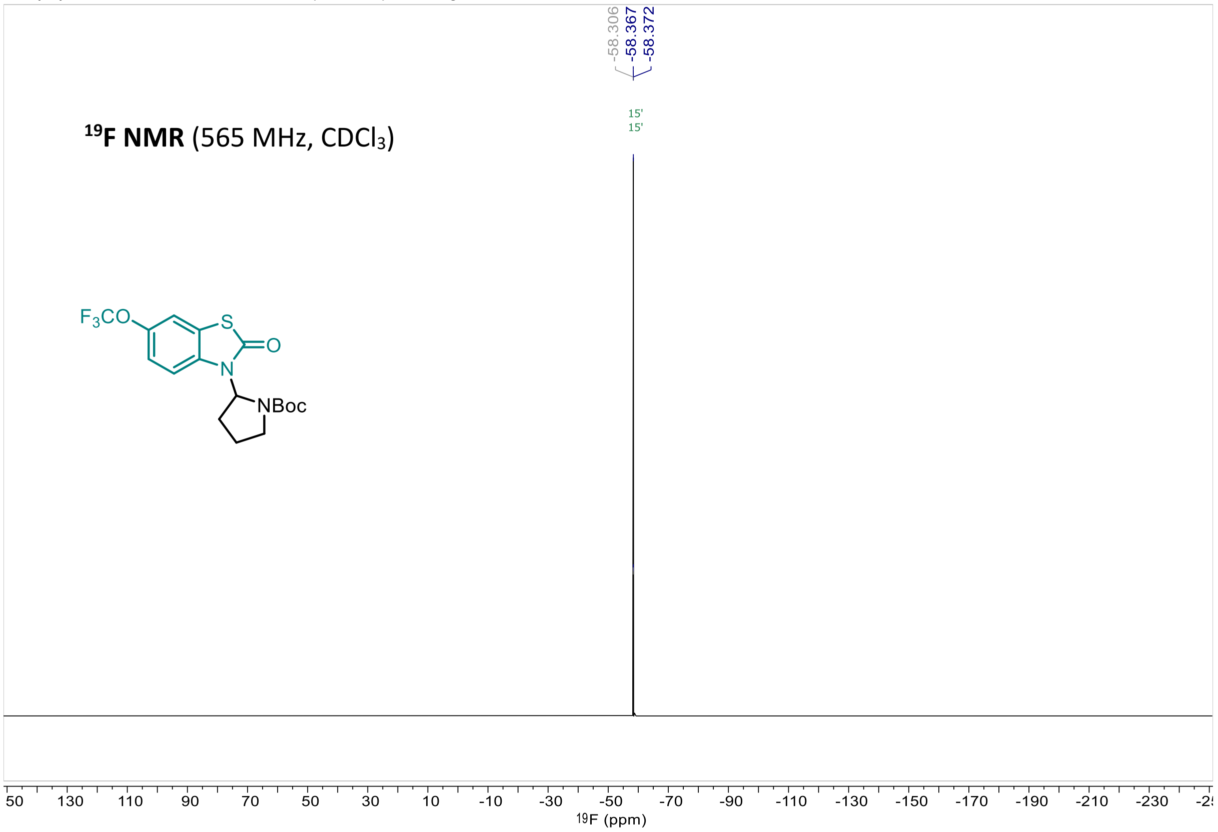

1H{13C} HSQC

1H{13C},HSQC-EDITED, 600.20 MHz,CDCl3,263.0K, pulse sequence: hsqcedetgpsisp2.3

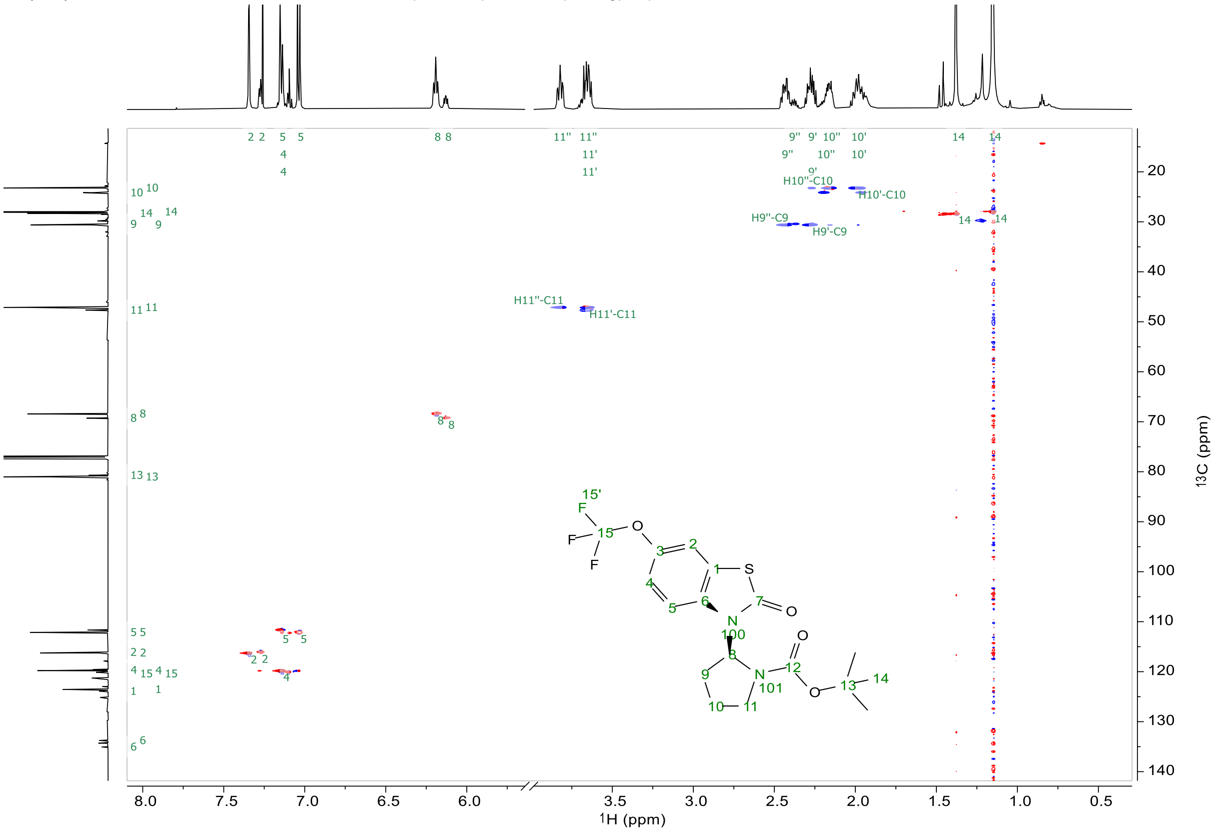

1H{13C} HMBC

1H{13C},HMBC, 600.20 MHz,CDCl3,263.0K, pulse sequence: hmbcetgpl3nd

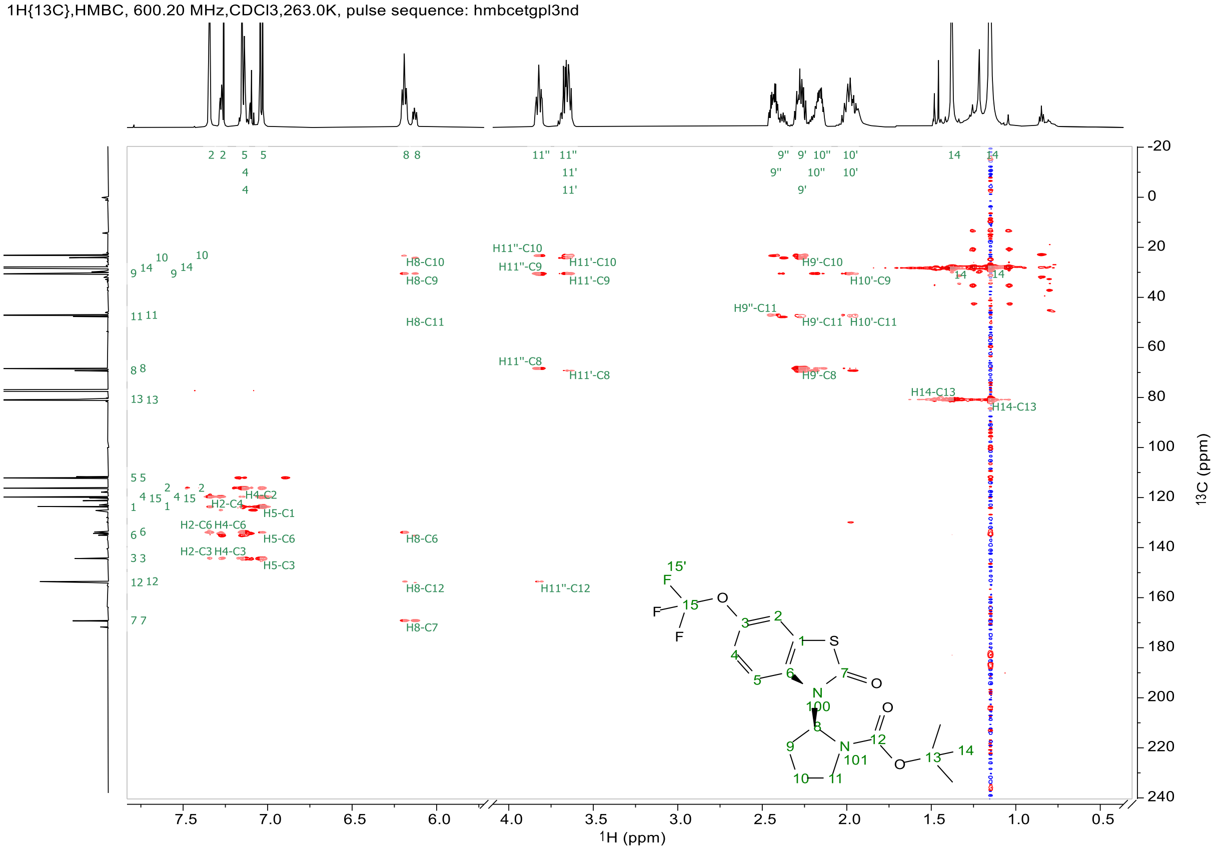

1H NOESY

1H{off},NOESY, 600.20 MHz,CDCl3,263.0K, pulse sequence: noesygpphpp

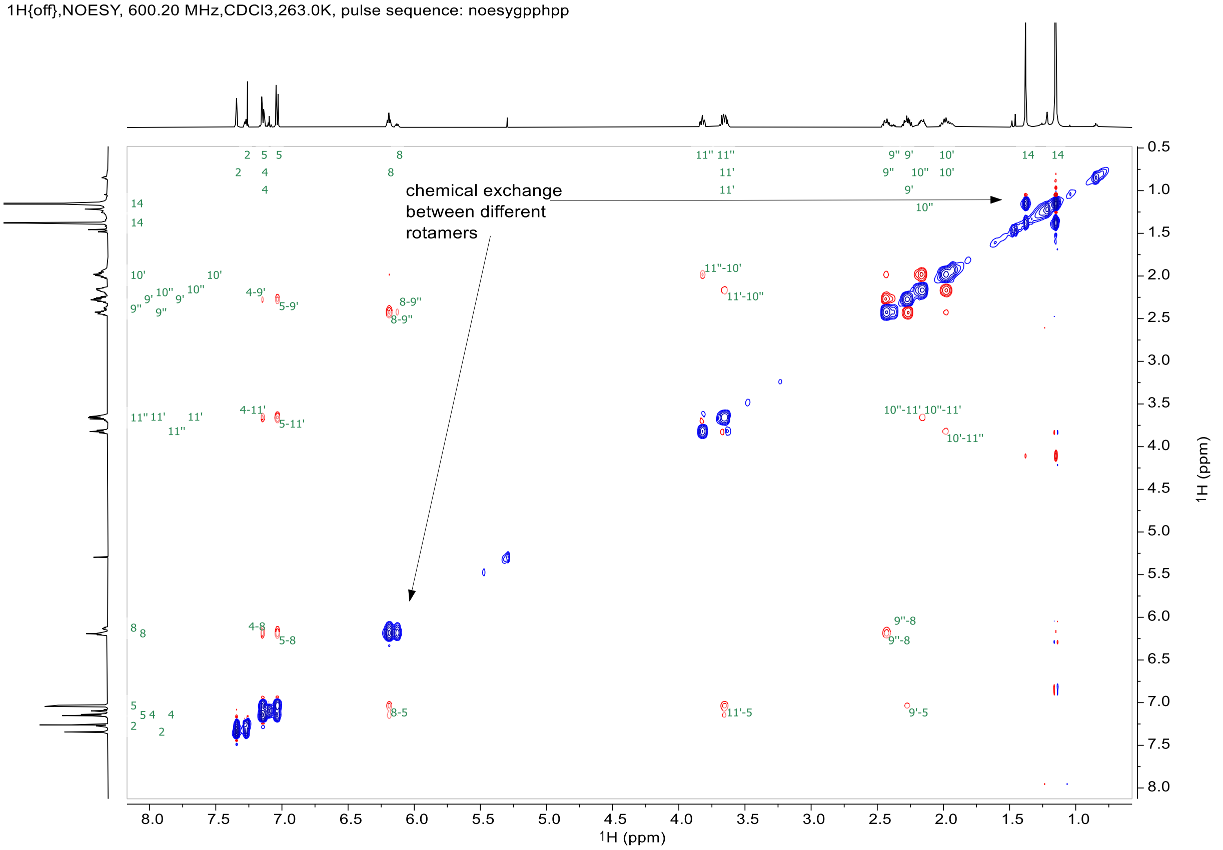

1H{15N} HMBC

1H{15N},HMBC, 600.20 MHz,CDCl3,263.0K, pulse sequence: hmbcgpndqf

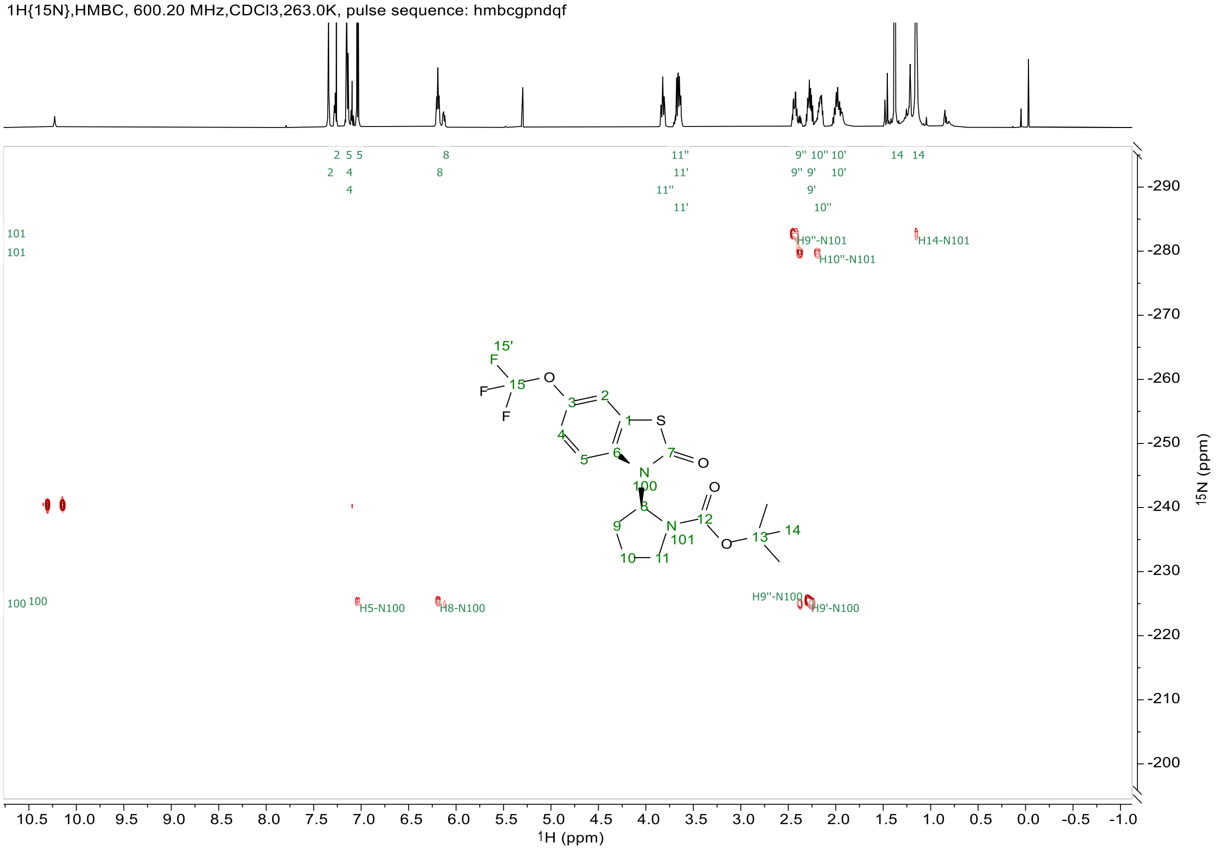

tert-Butyl 2-(4,5,6,7-tetrachloro-1,3-dioxoisoindolin-2-yl)pyrrolidine-1-carboxylate (**24**)

^{1}H NMR (600 MHz, 253 K, acetone-d_6)

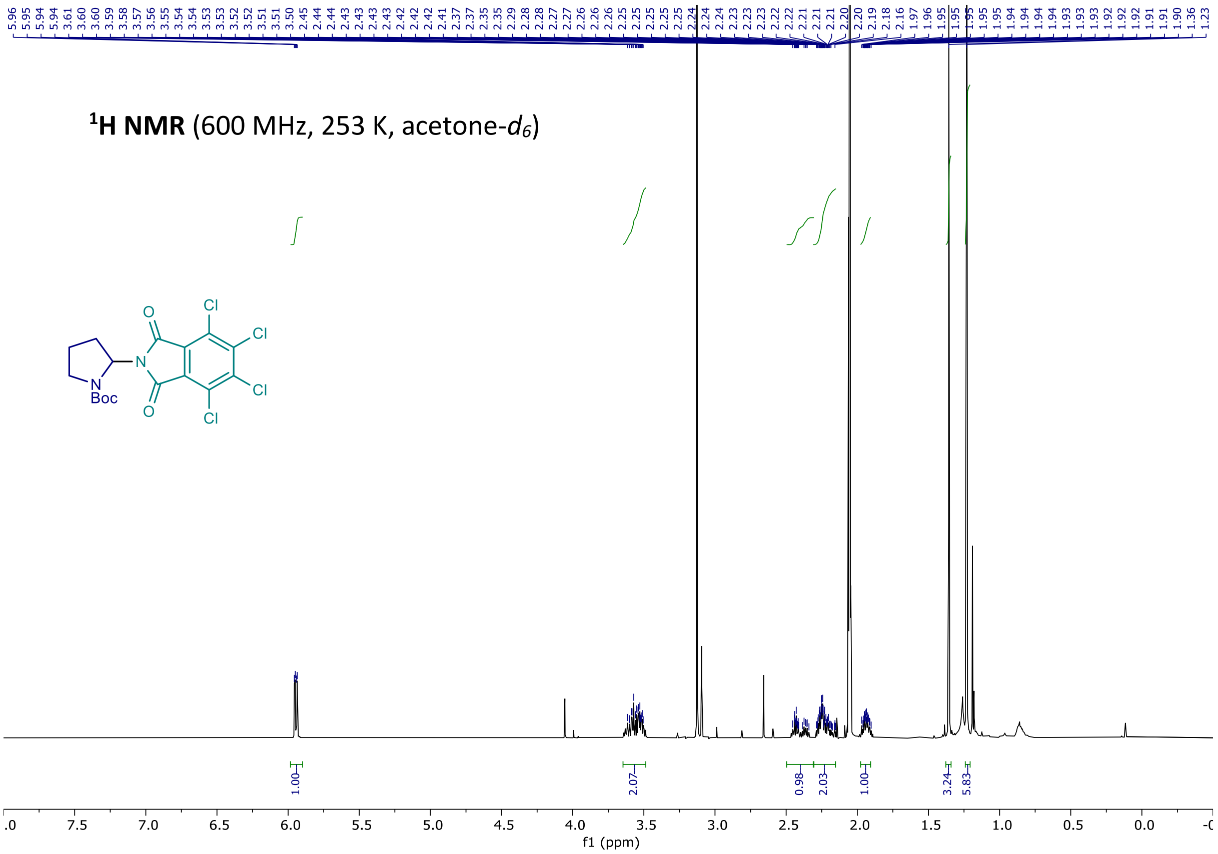

^{13}C NMR (151 MHz, 253 K, acetone-d_6)

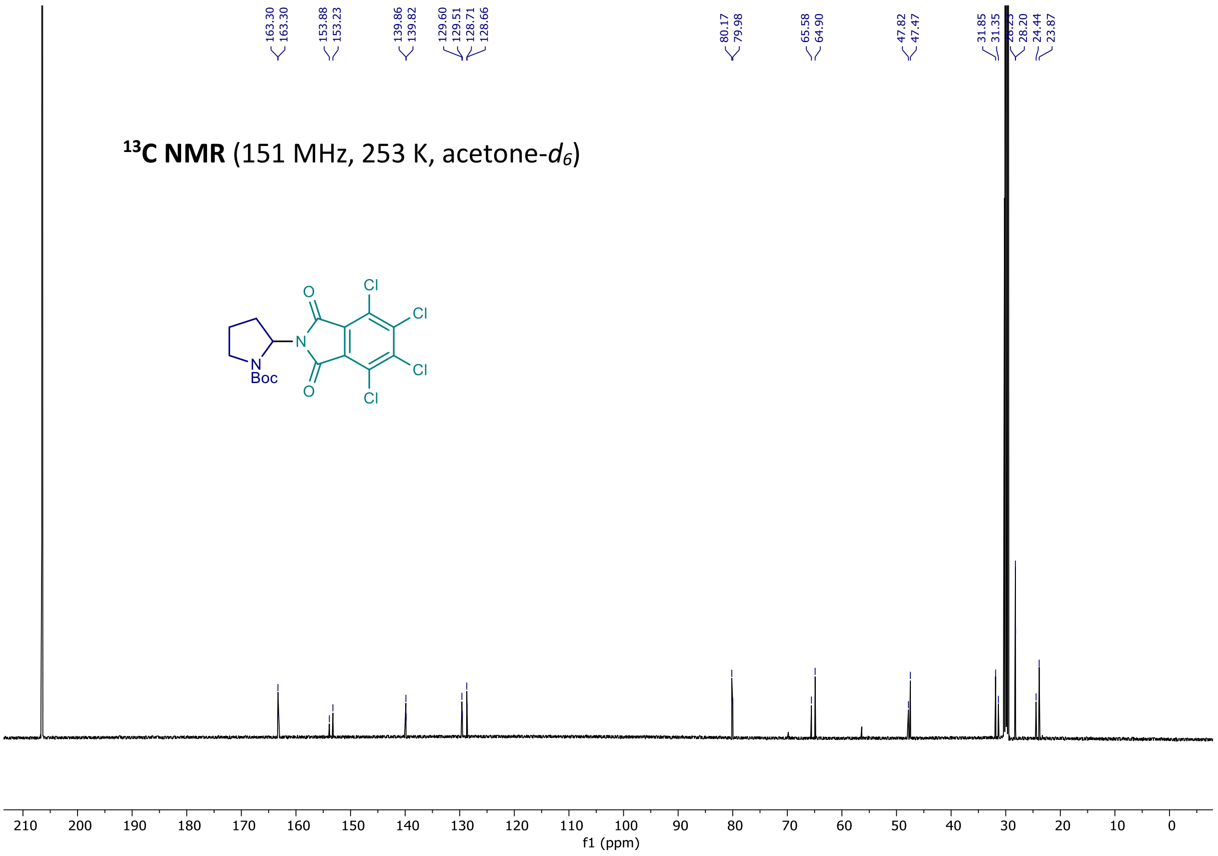

4,5,6,7-Tetrachloro-2-(2-(4-chlorophenoxy)propan-2-yl)isoindoline-1,3-dione (50)

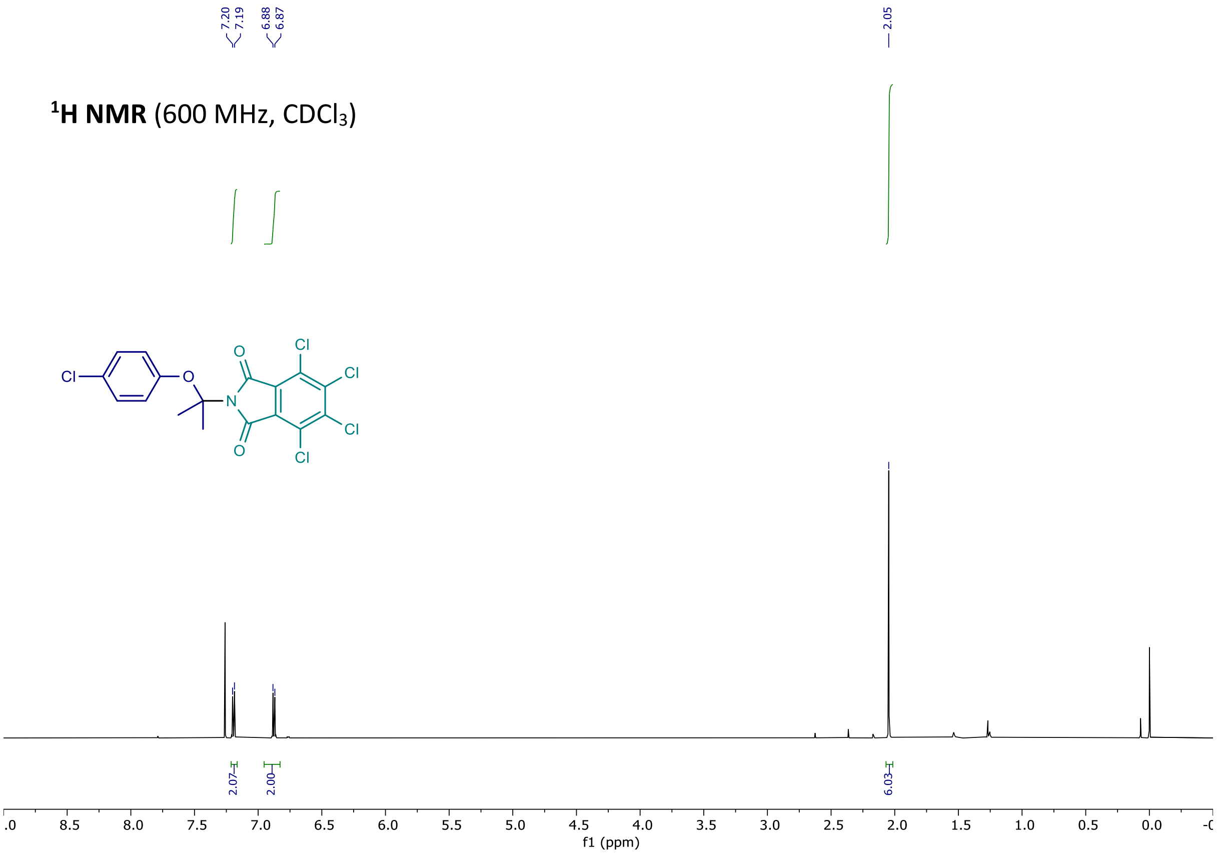

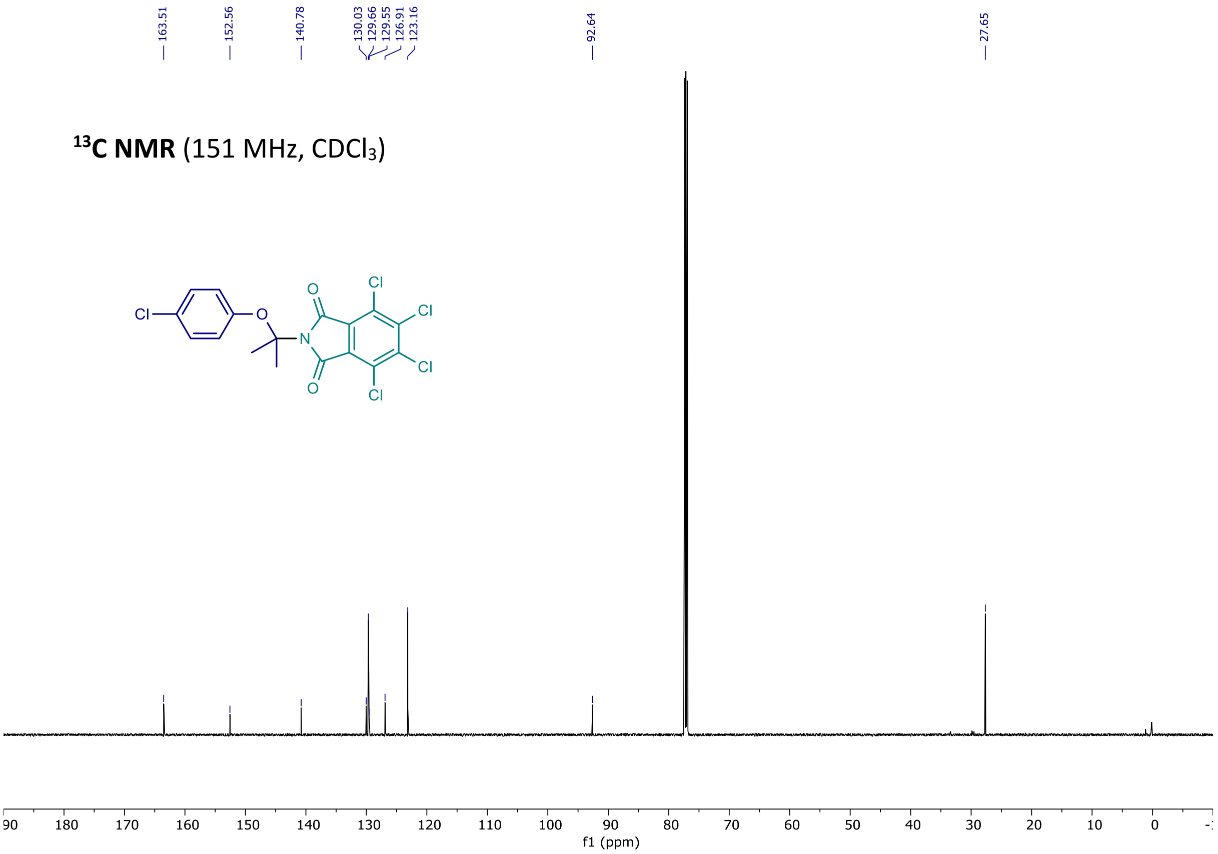

4,5,6,7-Tetrachloro-2-(2-(4-(4-chlorobenzoyl)phenoxy)propan-2-yl)isoindoline-1,3-dione (51)

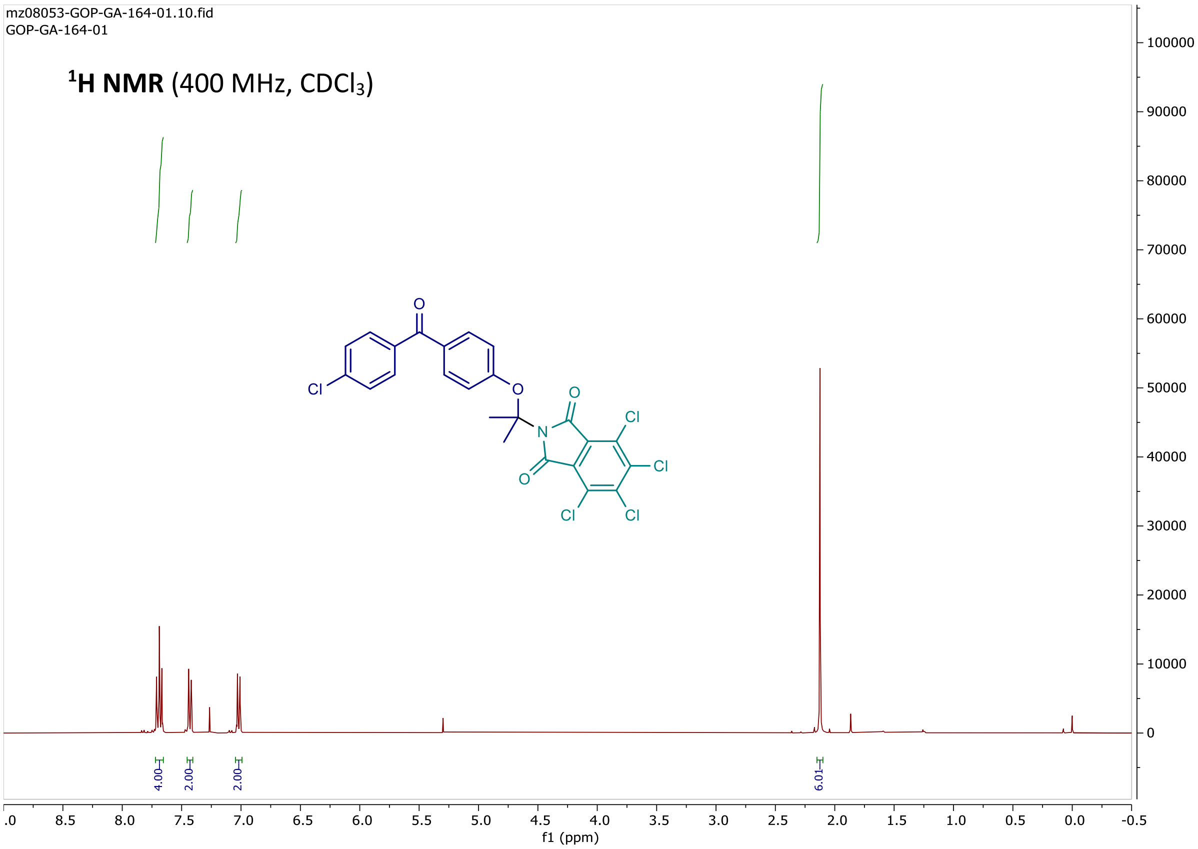

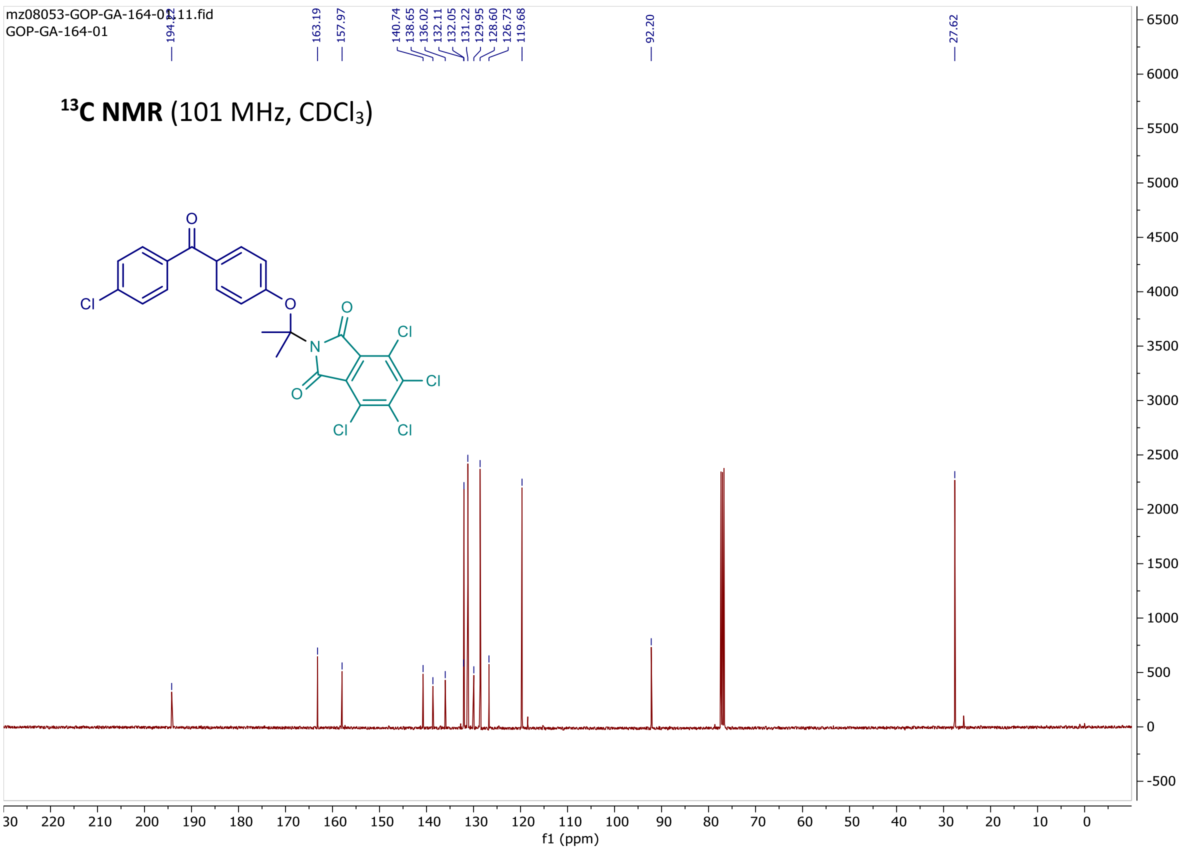

[(2,6-(*t*BuNCH)$_2$C$_6$H$_3$)Bi(*N*-Boc-pirrolid-2-yl)(tetrachlorophthalimide)] (23)

Mixture of rotamers (ca. 4:1) in DMF-d_7 at -40 °C, together with some starting Bi(I) (**1**)

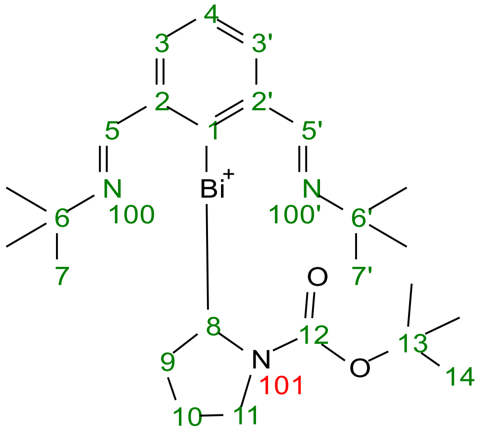

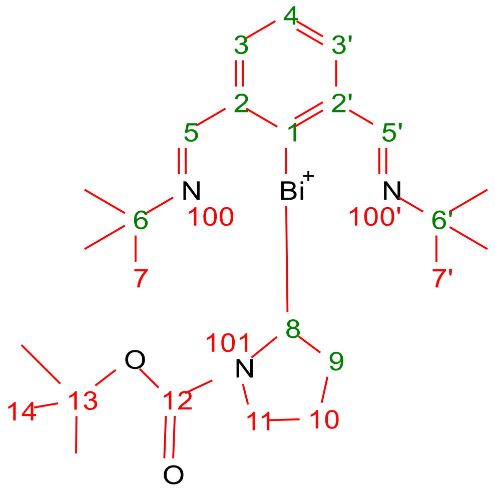

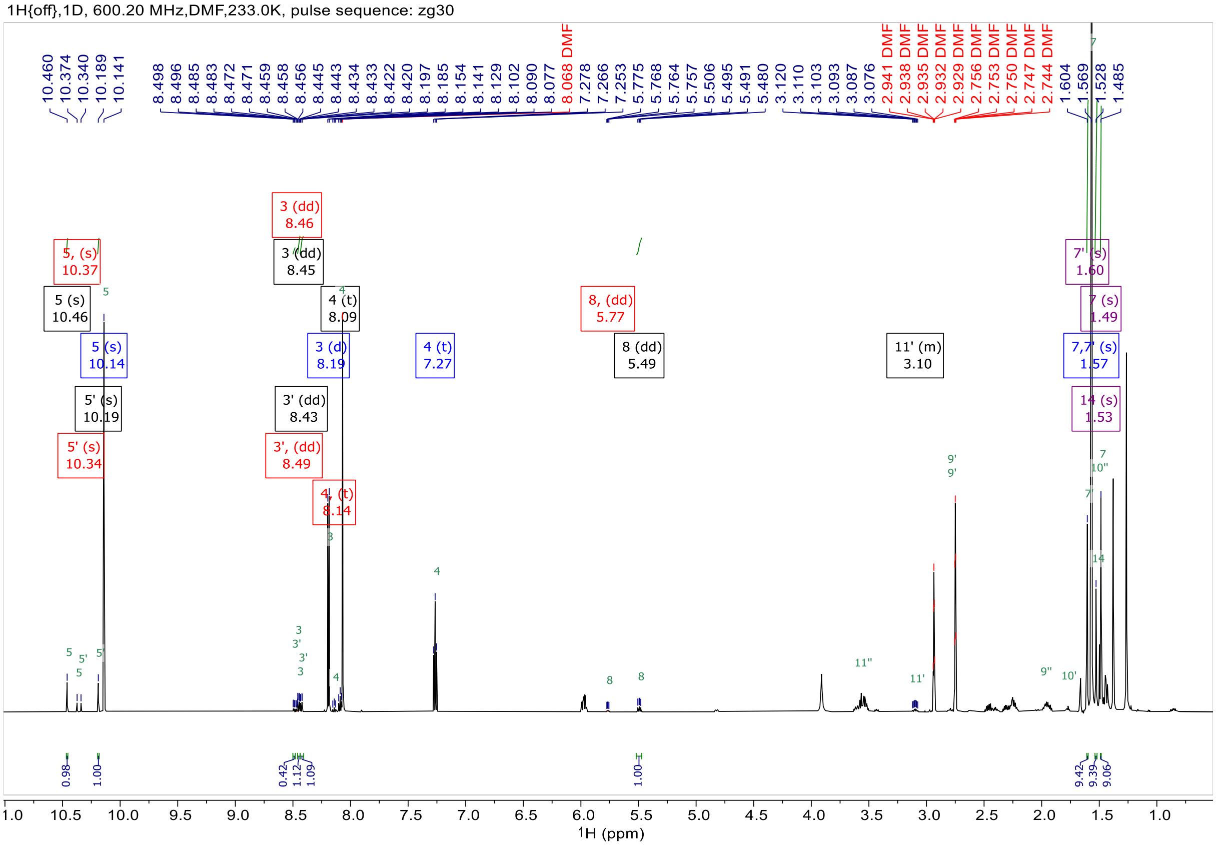

^{1}H NMR Detail of **23**

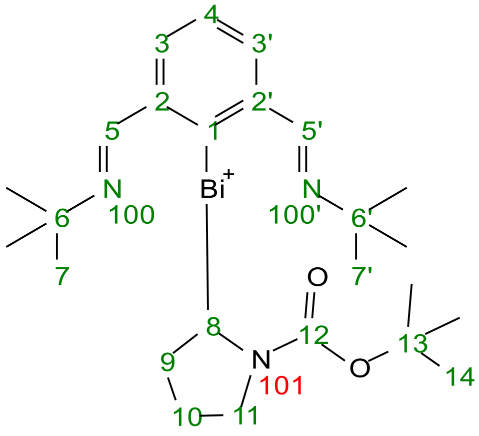

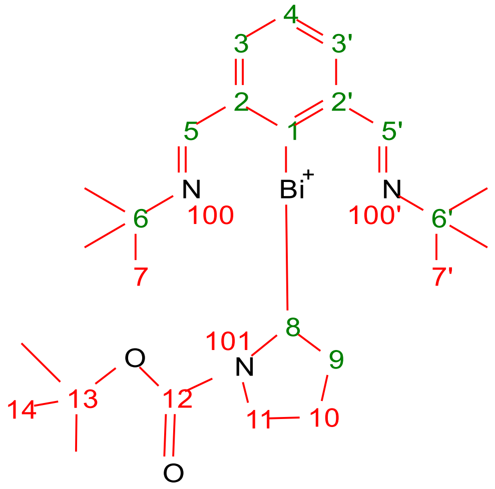

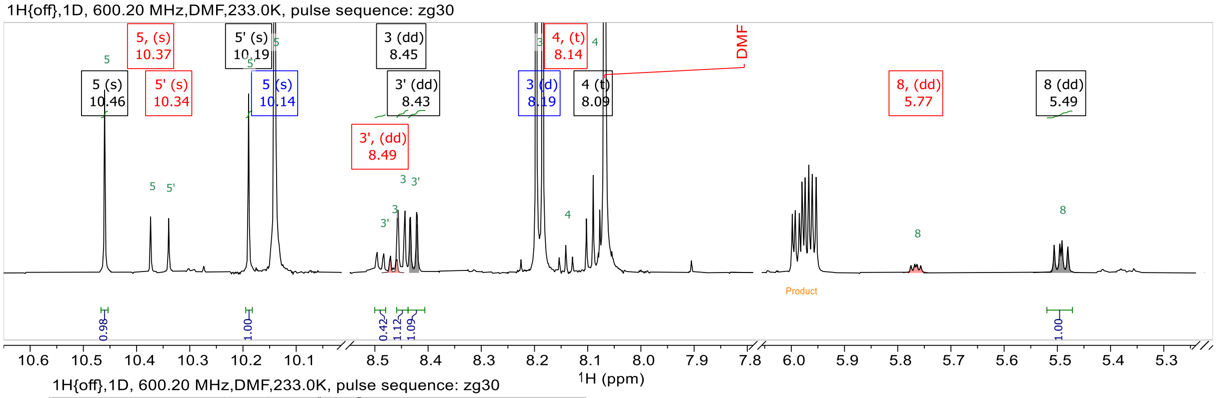

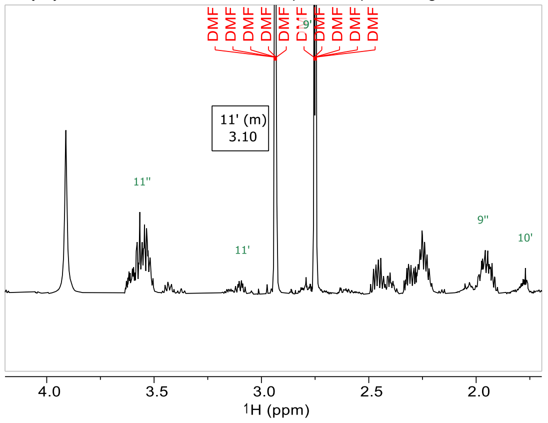

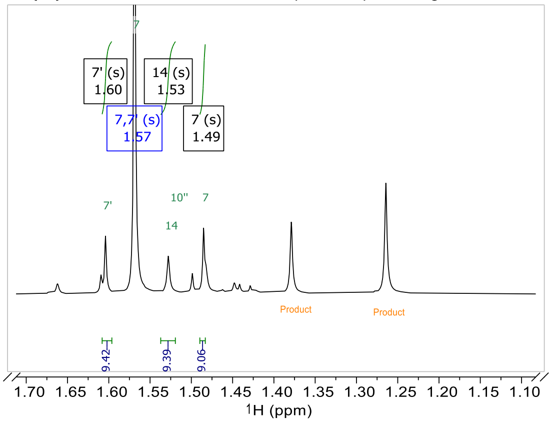

^{13}C NMR of **23**

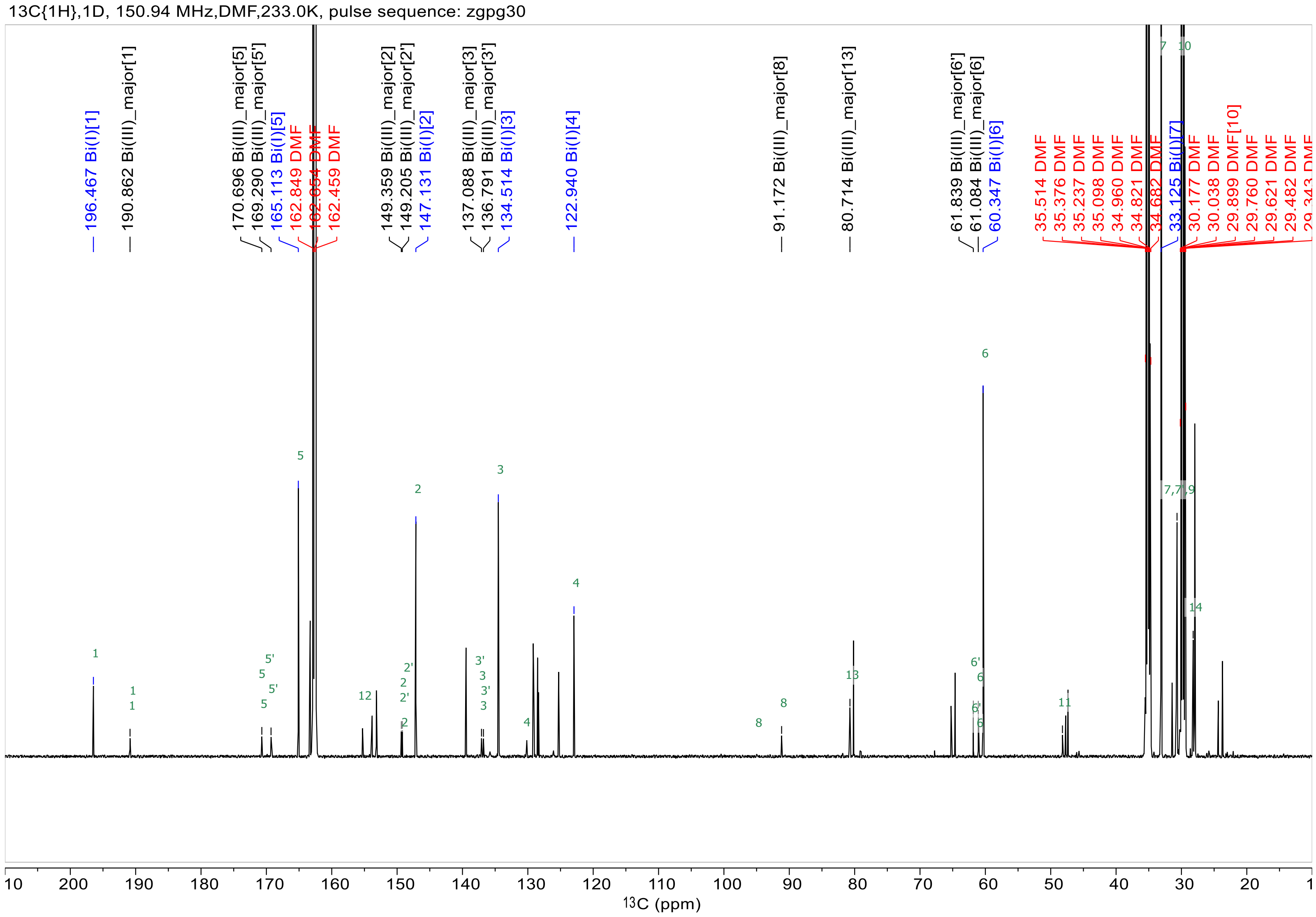
13C{1H},1D, 150.94 MHz,DMF,233.0K, pulse sequence: zgpg30
196.467 Bi(I)[1]
190.862 Bi(III)_major[1]
170.696 Bi(III)_major[5]
169.290 Bi(III)_major[5']
165.113 Bi(I)[5]
162.849 DMF
162.459 DMF
149.359 Bi(III)_major[2]
149.205 Bi(III)_major[2']
147.131 Bi(I)[2]
137.088 Bi(III)_major[3]
136.791 Bi(III)_major[3']
134.514 Bi(I)[3]
122.940 Bi(I)[4]
91.172 Bi(III)_major[8]
80.714 Bi(III)_major[13]
61.839 Bi(III)_major[6']
61.084 Bi(III)_major[6]
60.347 Bi(I)[6]
35.514 DMF
35.376 DMF
35.237 DMF
35.098 DMF
34.960 DMF
34.821 DMF
34.682 DMF
33.125 Bi(I)[7]
30.177 DMF
30.038 DMF
29.899 DMF[10]
29.760 DMF
29.621 DMF
29.482 DMF
13C (ppm)

1H{off},COSY, 600.20 MHz,DMF,233.0K, pulse sequence: cosygpppqf

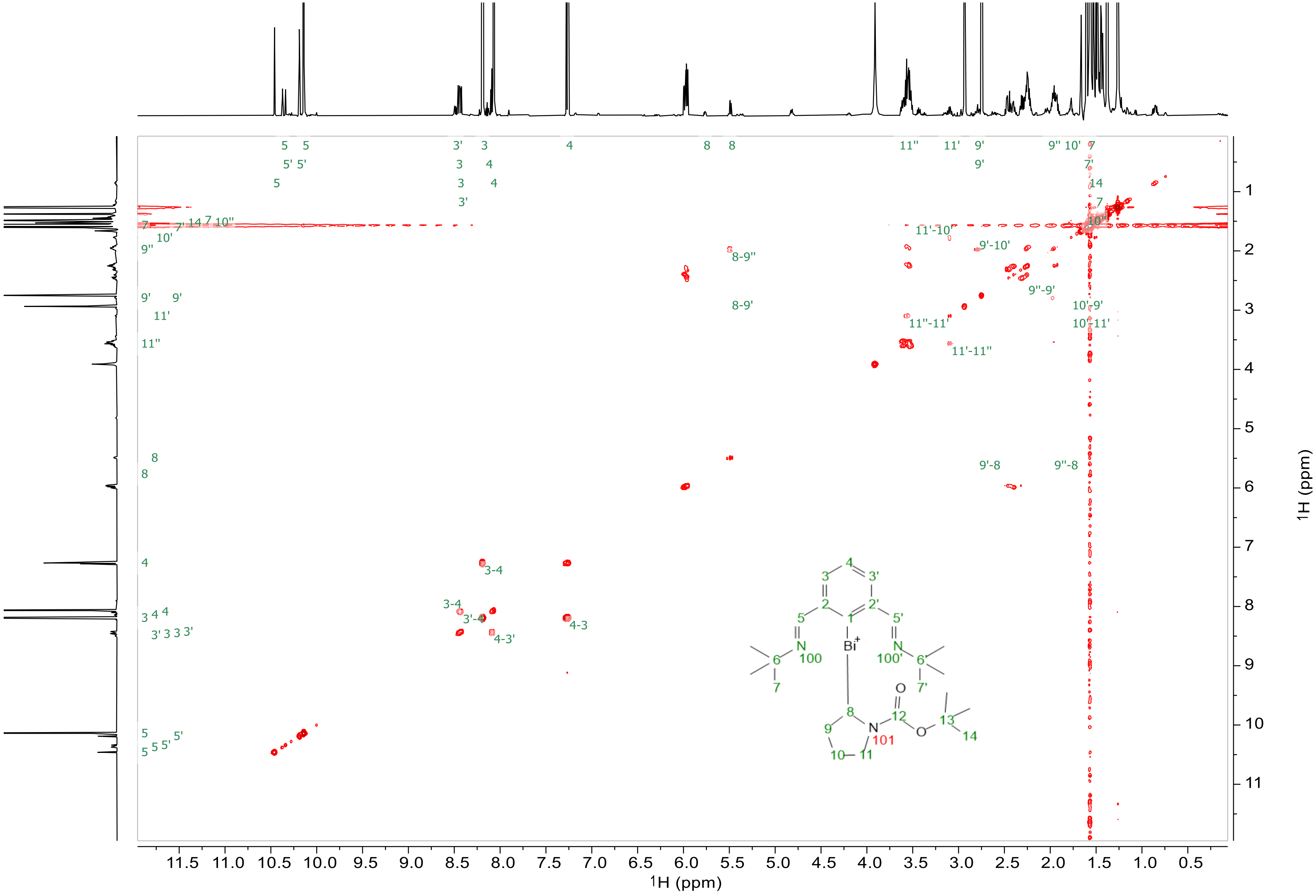

1H{13C},HSQC-EDITED, 600.20 MHz,DMF,233.0K, pulse sequence: hsqcedetgpsisp2.3

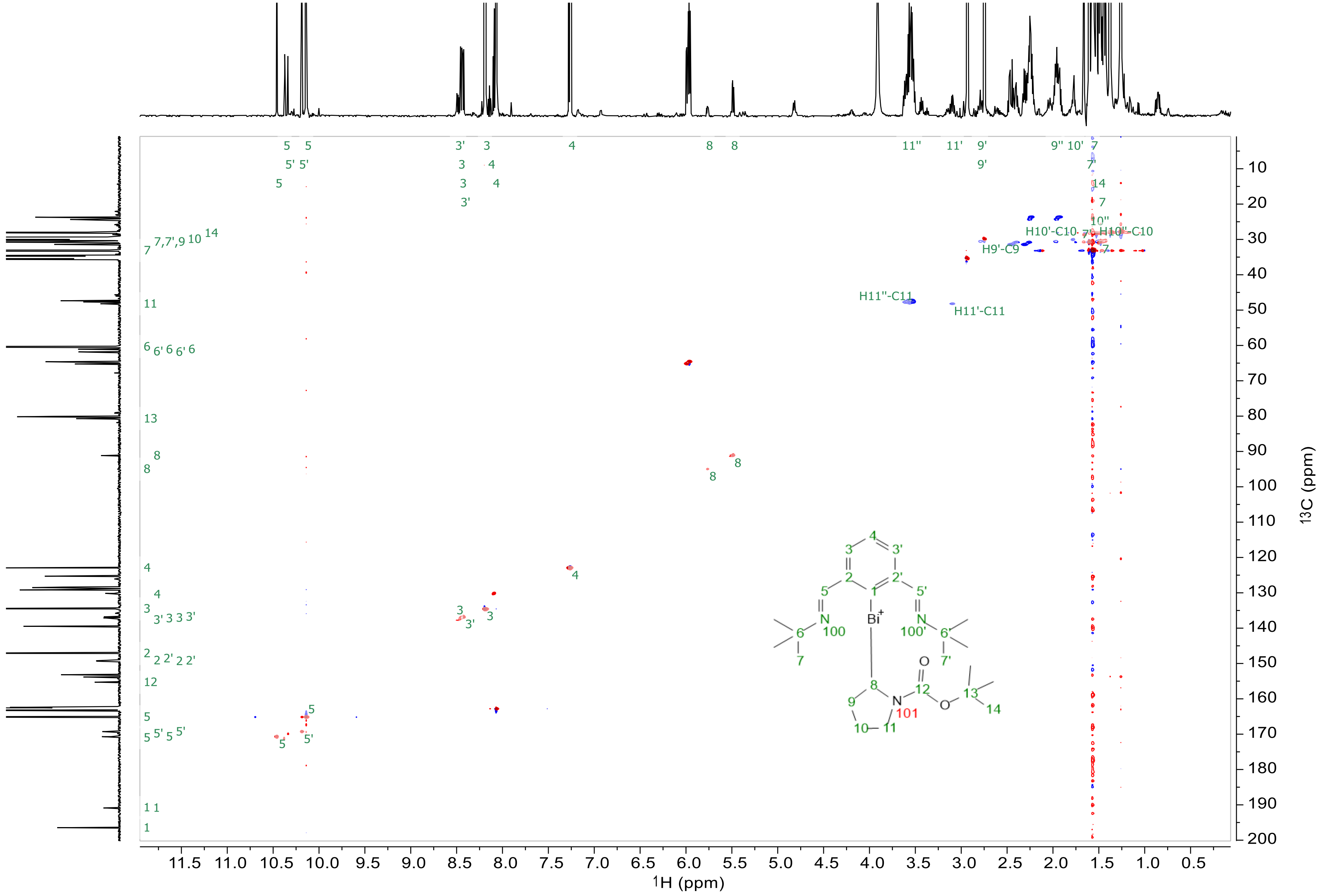

1H{13C},HMBC, 600.20 MHz,DMF,233.0K, pulse sequence: hmbcetgpl3nd

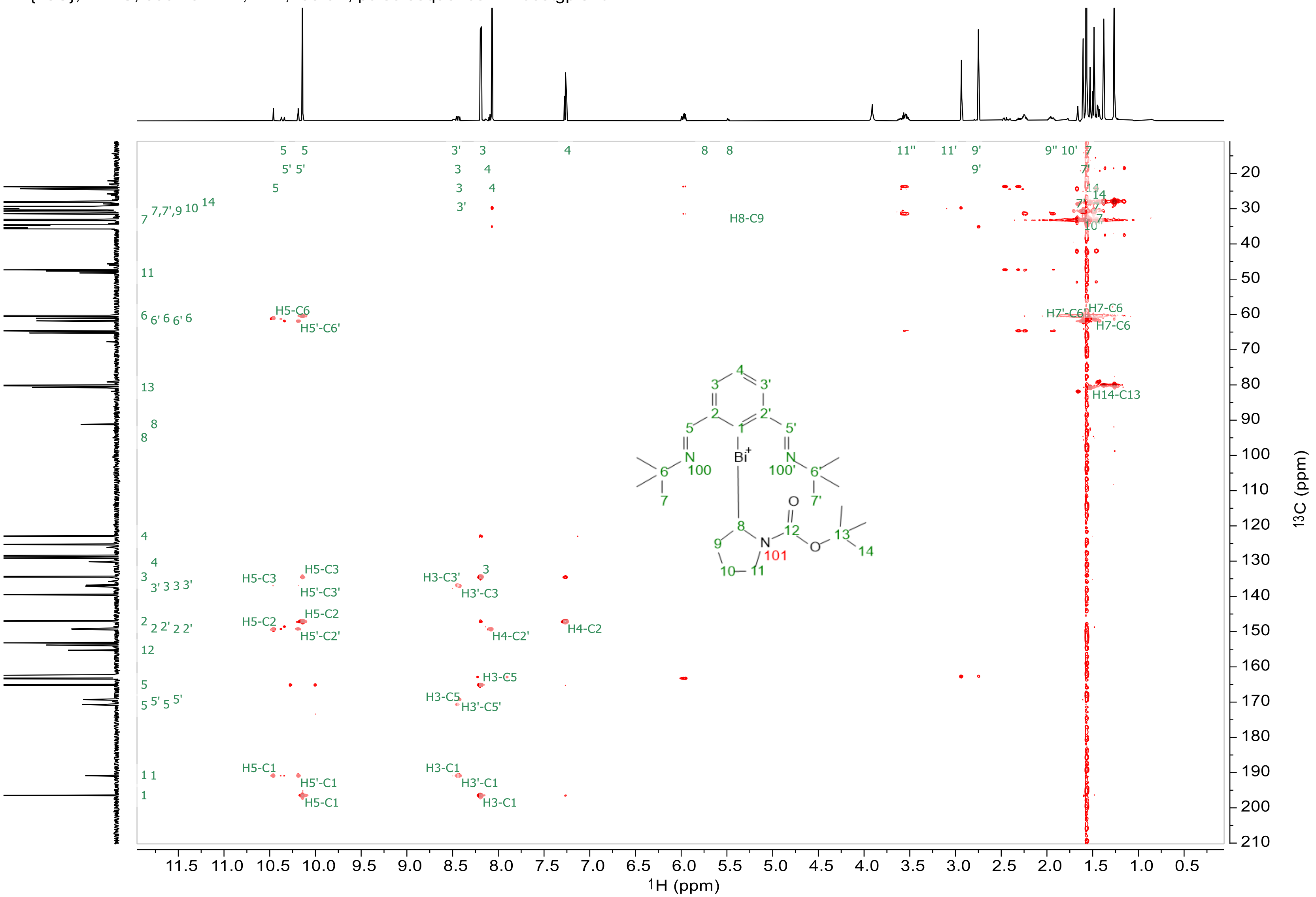

1H{13C},HMBC, 600.20 MHz,DMF,233.0K, pulse sequence: hmbcetgpl3nd

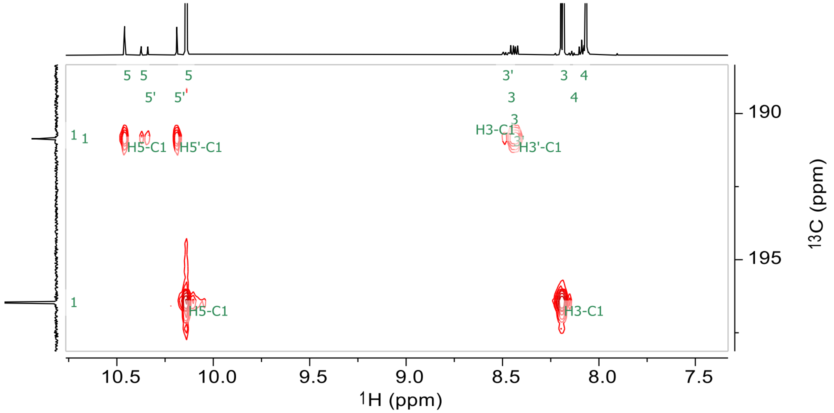

HMBC correlations to the ipso carbon (C1)

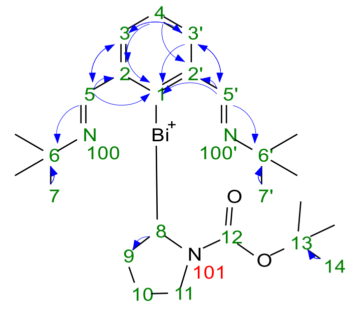

1H{15N},HMBC, 600.20 MHz,DMF,233.0K, pulse sequence: hmbcgpndqf

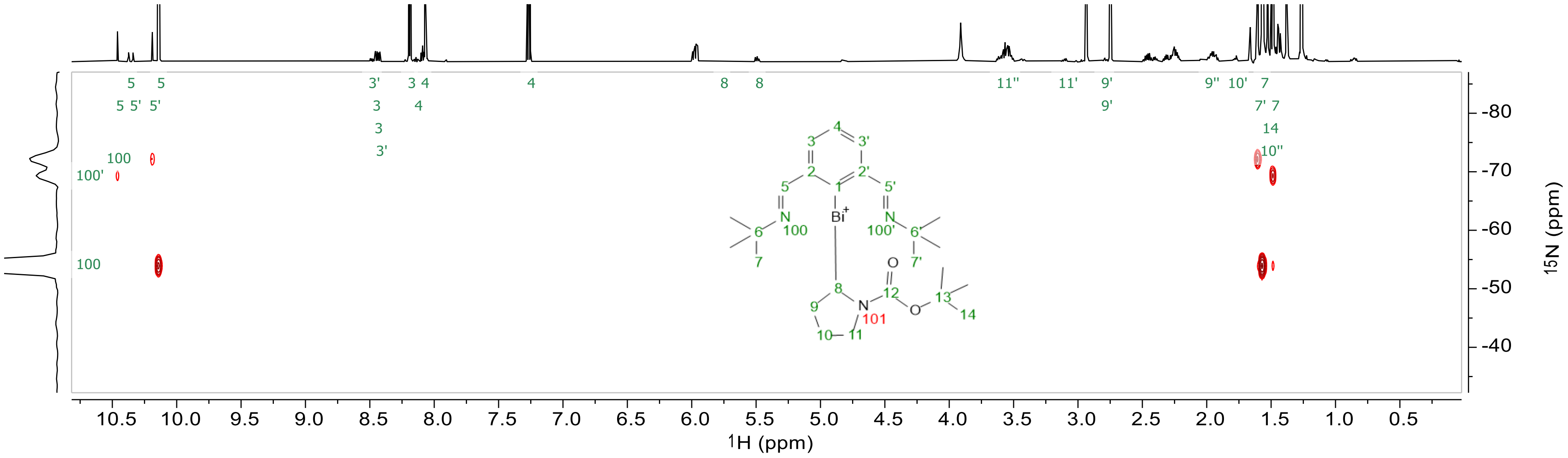

1H{off},1D, 600.20 MHz,DMF,233.0K, pulse sequence: selrogp

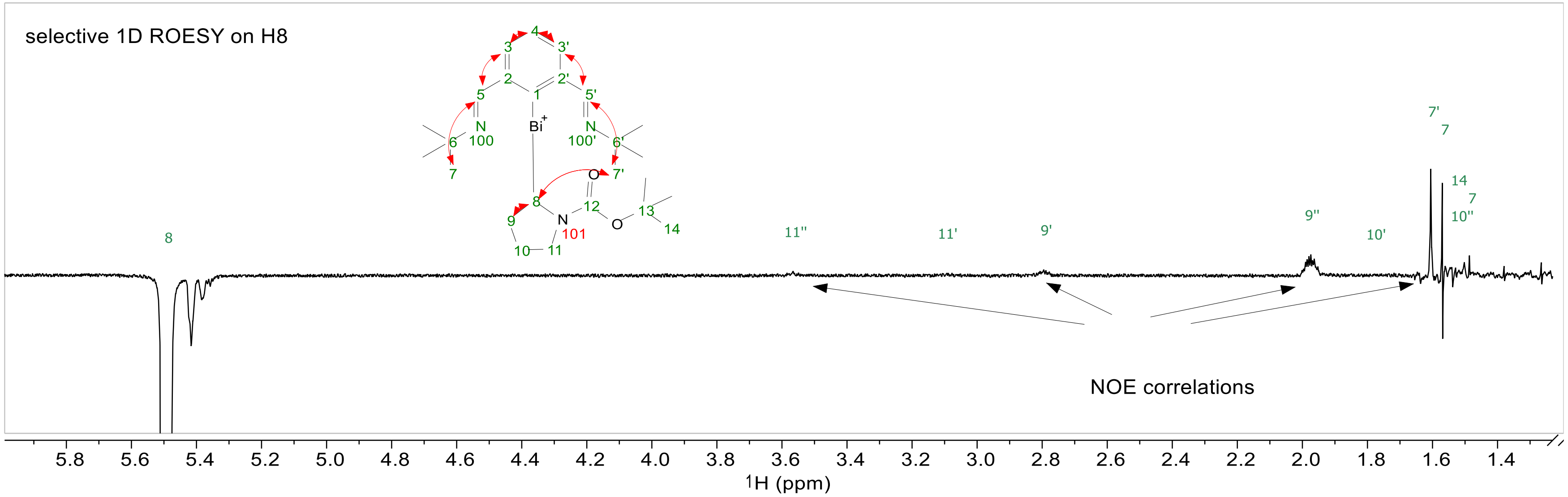

1H{off},1D, 600.20 MHz,DMF,233.0K, pulse sequence: seldigpzs

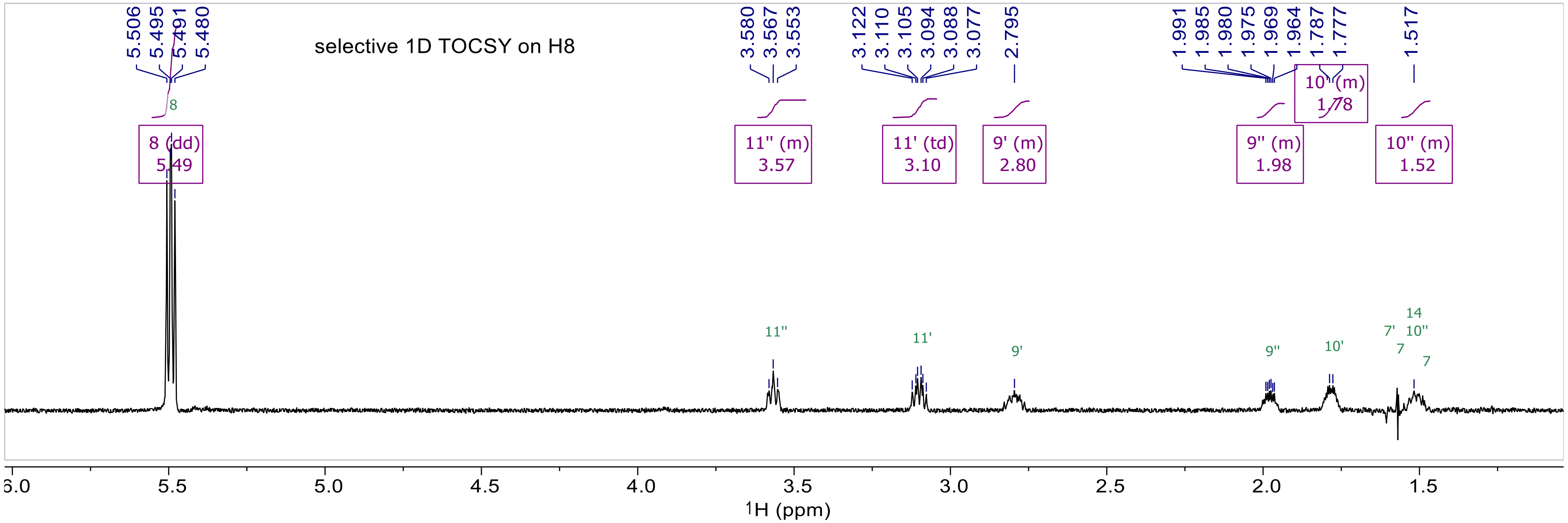

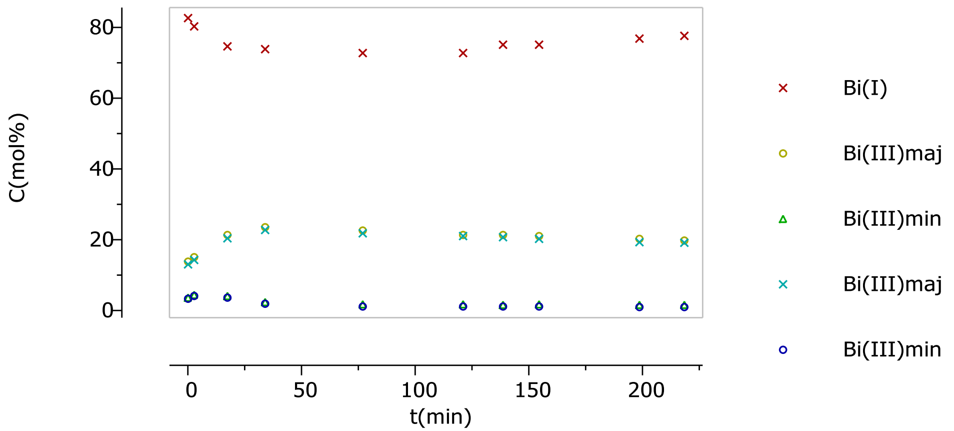

Decay of the Bi(III)-Species towards Bi(I) and reaction product observed even at -40°C

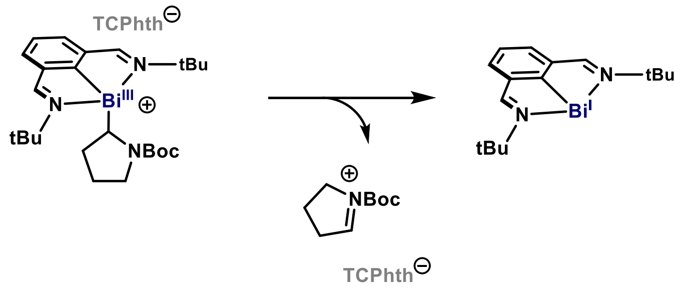

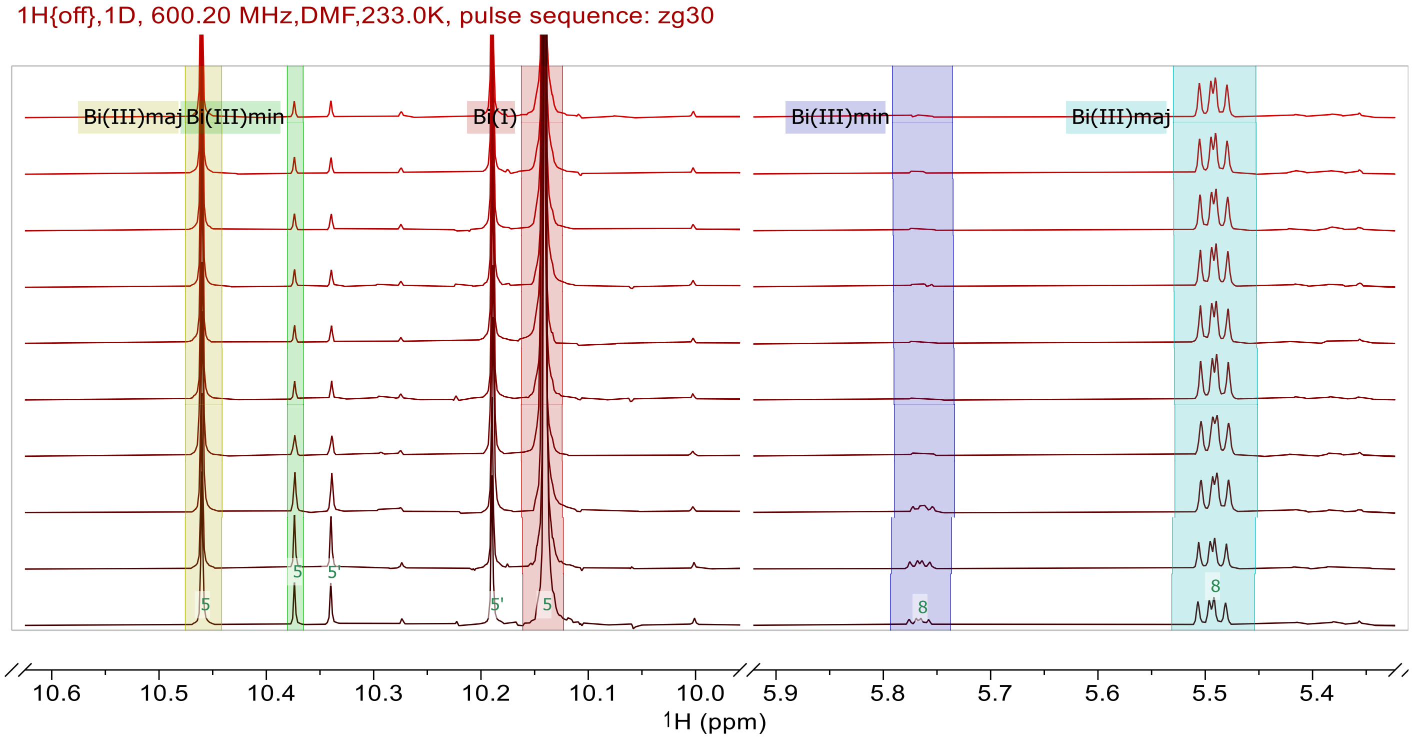

10. References

1. Vránová, I. *et al.* From Dibismuthenes to Three- and Two-Coordinated Bismuthinidenes by Fine Ligand Tuning: Evidence for Aromatic BiC_3N Rings through a Combined Experimental and Theoretical Study. *Chem. Eur. J.* **21**, 16917–16928 (2015).
2. Wang, F., Planas, O. & Cornella, J. Bi(I)-Catalyzed Transfer-Hydrogenation with Ammonia-Borane. *J. Am. Chem. Soc.* **141**, 4235–4240 (2019).
3. Pang, Y. *et al.* Catalytic Hydrodefluorination via Oxidative Addition, Ligand Metathesis, and Reductive Elimination at Bi(I)/Bi(III) Centers. *J. Am. Chem. Soc.* **143**, 12487–12493 (2021).
4. Qin, T. *et al.* A general alkyl-alkyl cross-coupling enabled by redox-active esters and alkylzinc reagents. *Science* **352**, 801–805 (2016).
5. Bosque, I. & Bach, T. 3-Acetoxyquinuclidine as Catalyst in Electron Donor–Acceptor Complex-Mediated Reactions Triggered by Visible Light. *ACS Catal.* **9**, 9103–9109 (2019).
6. Zhang, Z. & Cernak, T. The Formal Cross-Coupling of Amines and Carboxylic Acids to Form sp^3–sp^3 Carbon–Carbon Bonds. *Angew. Chem. Int. Ed.* **60**, 27293–27298 (2021).
7 A. Fawcett *et al.* Photoinduced decarboxylative borylation of carboxylic acids. *Science* **357**, 283–286 (2017).
8 Cheng, W.-M., Shang, R. & Fu, Y. Irradiation-induced palladium-catalyzed decarboxylative desaturation enabled by a dual ligand system. *Nat. Commun.* **9**, 5215 (2018).
9. Liwosz, T. W. & Chemler, S. R. Copper-Catalyzed Oxidative Heck Reactions between Alkyltrifluoroborates and Vinyl Arenes. *Org. Lett.* **15**, 3034–3037 (2013).
10. Chambers, D. R. *et al.* C–O Bond Cleavage of Alcohols via Visible Light Activation of Cobalt Alkoxycarbonyls. *Chem. Eur. J.* **23**, 10259–10263 (2017).
11. Zhang, J.-J. *et al.* Visible-Light-Mediated Dual Decarboxylative Coupling of Redox-Active Esters with α,β-Unsaturated Carboxylic Acids. *Organometallics* **38**, 4570–4577 (2019).
12. Shao, X. *et al.* Decarboxylative C_{sp3}–N Bond Formation by Electrochemical Oxidation of Amino Acids. *Org. Lett.* **21**, 9262–9267 (2019).
13 Meindl, K., Herbst-Irmer, R. & Henn, J. On the effect of neglecting anharmonic nuclear motion in charge density studies. *Acta Cryst.* **A66**, 362–371 (2010).
